# The Doryctinae (Braconidae) of Costa Rica: genera and species of the tribe Heterospilini

**DOI:** 10.3897/zookeys.347.6002

**Published:** 2013-11-06

**Authors:** Paul M. Marsh, Alexander L. Wild, James B. Whitfield

**Affiliations:** 1 P. O. Box 384, North Newton, KS 67117; 2 Department of Entomology, 320 Morrill Hall, 505 S. Goodwin Avenue, University of Illinois at Urbana-Champaign, Urbana, IL 61801

**Keywords:** Parasitoid wasps, Braconidae, Doryctinae, Heterospilini, *Heterospilus*, Costa Rica

## Abstract

A comprehensive taxonomic study is presented for the four genera and 286 species of the doryctine tribe Heterospilini occurring in Costa Rica. The tribe is represented almost entirely by the 280 species of the genus *Heterospilus* Haliday. Keys for identification of the genera and species are provided and the genera and species are described and illustrated. An interactive key to the species of *Heterospilus* also was prepared using Lucid Builder. The following new genus and species are described from Costa Rica: *Paraheterospilus* **gen. n.**, *P. ceciliaensis* **sp. n.**, *P. eumekus* **sp. n.**, *P. wilbotgardus* **sp. n.**, *Heterospilus achi* **sp. n.**, *H. achterbergi* **sp. n.**, *H. aesculapius* **sp. n.**, *H. agujas* **sp. n.**, *H. agujasensis* **sp. n.**, *H. alajuelus* **sp. n.**, *H. albocoxalis* **sp. n.**, *H. alejandroi* **sp. n.**, *H. amuzgo* **sp. n.**, *H. angelicae* **sp. n.**, *H. angustus* **sp. n.**, *H. aphrodite* **sp. n.**, *H. apollo* **sp. n.**, *H. arawak* **sp. n.**, *H. areolatus* **sp. n.**, *H. artemis* **sp. n.**, *H. athena* **sp. n.**, *H. attraholucus* **sp. n.**, *H. aubreyae* **sp. n.**, *H. austini* **sp. n.**, *H. azofeifai* **sp. n.**, *H. bacchus* **sp. n.**, *H. barbalhoae* **sp. n.**, *H. bennetti* **sp. n.**, *H. bicolor* **sp. n.**, *H. boharti* **sp. n.**, *H. borucas* **sp. n.**, *H. braeti* **sp. n.**, *H. brethesi* **sp. n.**, *H. breviarius* **sp. n.**, *H. brevicornus* **sp. n.**, *H. bribri* **sp. n.**, *H. brullei* **sp. n.**, *H. bruesi* **sp. n.**, *H. cabecares* **sp. n.**, *H. cacaoensis* **sp. n.**, *H. cachiensis* **sp. n.**, *H. cameroni* **sp. n.**, *H. cangrejaensis* **sp. n.**, *H. careonotaulus* **sp. n.**, *H. caritus* **sp. n.**, *H. carolinae* **sp. n.**, *H. cartagoensis* **sp. n.**, *H. catiensis* **sp. n.**, *H. catorce* **sp. n.**, *H. cero* **sp. n.**, *H. chaoi* **sp. n.**, *H. chilamatensis* **sp. n.**, *H. chocho* **sp. n.**, *H. chorotegus* **sp. n.**, *H. chorti* **sp. n.**, *H. cinco* **sp. n.**, *H. cocopa* **sp. n.**, *H. colliletus* **sp. n.**, *H. colonensis* **sp. n.**, *H. complanatus* **sp. n.**, *H. conservatus* **sp. n.**, *H. cora* **sp. n.**, *H. corcovado* **sp. n.**, *H. corrugatus* **sp. n.**, *H. costaricensis* **sp. n.**, *H. cressoni* **sp. n.**, *H. cuatro* **sp. n.**, *H. curtisi* **sp. n.**, *H. cushmani* **sp. n.**, *H. dani* **sp. n.**, *H. demeter* **sp. n.**, *H. dianae* **sp. n.**, *H. diecinueve* **sp. n.**, *H. dieciocho* **sp. n.**, *H. dieciseis* **sp. n.**, *H. diecisiete* **sp. n.**, *H. diez* **sp. n.**, *H. doce* **sp. n.**, *H. dos* **sp. n.**, *H. dulcus* **sp. n.**, *H. eberhardi* **sp. n.**, *H. ektorincon* **sp. n.**, *H. emilius* **sp. n.**, *H. empalmensis* **sp. n.**, *H. enderleini* **sp. n.**, *H. escazuensis* **sp. n.**, *H. fahringeri* **sp. n.**, *H. fischeri* **sp. n.**, *H. flavidus* **sp. n.**, *H. flavisoma* **sp. n.**, *H. flavostigmus* **sp. n.**, *H. foersteri* **sp. n.**, *H. fonsecai* **sp. n.**, *H. fournieri* **sp. n.**, *H. gahani* **sp. n.**, *H. garifuna* **sp. n.**, *H. gauldi* **sp. n.**, *H. golfodulcensis* **sp. n.**, *H. gouleti* **sp. n.**, *H. granulatus* **sp. n.**, *H. grisselli* **sp. n.**, *H. guanacastensis* **sp. n.**, *H. guapilensis* **sp. n.**, *H. hachaensis* **sp. n.**, *H. halidayi* **sp. n.**, *H. hansoni* **sp. n.**, *H. hansonorum* **sp. n.**, *H. haplocarinus* **sp. n.**, *H. hedqvisti* **sp. n.**, *H. hera* **sp. n.**, *H. heredius* **sp. n.**, *H. hespenheidei* **sp. n.**, *H. holleyae* **sp. n.**, *H. huddlestoni* **sp. n.**, *H. huetares* **sp. n.**, *H. hypermekus* **sp. n.**, *H. itza* **sp. n.**, *H. ixcatec* **sp. n.**, *H. ixil* **sp. n.**, *H. jabillosensis* **sp. n.**, *H. jakaltek* **sp. n.**, *H. janzeni* **sp. n.**, *H. jennieae* **sp. n.**, *H. jonmarshi* **sp. n.**, *H. jupiter* **sp. n.**, *H. kellieae* **sp. n.**, *H. kiefferi* **sp. n.**, *H. kikapu* **sp. n.**, *H. kulai* **sp. n.**, *H. kuna* **sp. n.**, *H. lapierrei* **sp. n.**, *H. lasalturus* **sp. n.**, *H. laselvus* **sp. n.**, *H. leenderti* **sp. n.**, *H. leioenopus* **sp. n.**, *H. leiponotaulus* **sp. n.**, *H. lenca* **sp. n.**, *H. levis* **sp. n.**, *H. leviscutum* **sp. n.**, *H. levitergum* **sp. n.**, *H. limonensis* **sp. n.**, *H. longinoi* **sp. n.**, *H. longisulcus* **sp. n.**, *H. longius* **sp. n.**, *H. luteogaster* **sp. n.**, *H. luteoscutum* **sp. n.**, *H. luteus* **sp. n.**, *H. macrocarinus* **sp. n.**, *H. macrocaudatus* **sp. n.**, *H. magnus* **sp. n.**, *H. malaisei* **sp. n.**, *H. mam* **sp. n.**, *H. maritzaensis* **sp. n.**, *H. mars* **sp. n.**, *H. masneri* **sp. n.**, *H. masoni* **sp. n.**, *H. mellosus* **sp. n.**, *H. menkei* **sp. n.**, *H. mercury* **sp. n.**, *H. milleri* **sp. n.**, *H. miskito* **sp. n.**, *H. mixtec* **sp. n.**, *H. monteverde* **sp. n.**, *H. mopanmaya* **sp. n.**, *H. muertensis* **sp. n.**, *H. muesebecki* **sp. n.**, *H. nahua* **sp. n.**, *H. neesi* **sp. n.**, *H. nemestrinus* **sp. n.**, *H. nephilim* **sp. n.**, *H. nephus* **sp. n.**, *H. nigracapitus* **sp. n.**, *H. nigragonatus* **sp. n.**, *H. nigricoxus* **sp. n.**, *H. nixoni* **sp. n.**, *H. noyesi* **sp. n.**, *H. nueve* **sp. n.**, *H. nunesi* **sp. n.**, *H. once* **sp. n.**, *H. orbitus* **sp. n.**, *H. orosi* **sp. n.**, *H. paloverde* **sp. n.**, *H. pappi* **sp. n.**, *H. parkeri* **sp. n.**, *H. parvus* **sp. n.**, *H. pech* **sp. n.**, *H. penosa* **sp. n.**, *H. petiolatus* **sp. n.**, *H. petralbus* **sp. n.**, *H. phaeocoxus* **sp. n.**, *H. phaeoskelus* **sp. n.**, *H. pharkidodus* **sp. n.**, *H. phytorius* **sp. n.**, *H. pitillaensis* **sp. n.**, *H. poqomchi* **sp. n.**, *H. poqomom* **sp. n.**, *H. puertoviejoensis* **sp. n.**, *H. puntarensis* **sp. n.**, *H. qanjobal* **sp. n.**, *H. quickei* **sp. n.**, *H. quitirrisi* **sp. n.**, *H. racostica* **sp. n.**, *H. rama* **sp. n.**, *H. ramirezi* **sp. n.**, *H. ratzeburgi* **sp. n.**, *H. reagani* **sp. n.**, *H. reinhardi* **sp. n.**, *H. retheospilus* **sp. n.**, *H. rhabdotus* **sp. n.**, *H. ricacosta* **sp. n.**, *H. rinconensis* **sp. n.**, *H. robbieae* **sp. n.**, *H. rohweri* **sp. n.**, *H. rojasi* **sp. n.**, *H. romani* **sp. n.**, *H. rugosus* **sp. n.**, *H. sabrinae* **sp. n.**, *H. saminae* **sp. n.**, *H. sanjosensis* **sp. n.**, *H. santarosensis* **sp. n.**, *H. sanvitoensis* **sp. n.**, *H. saturn* **sp. n.**, *H. seis* **sp. n.**, *H. sergeyi* **sp. n.**, *H. sharkeyi* **sp. n.**, *H. shawi* **sp. n.**, *H. shenefelti* **sp. n.**, *H. shonan* **sp. n.**, *H. siete* **sp. n.**, *H. similis* **sp. n.**, *H. sinuatus* **sp. n.**, *H. smithi* **sp. n.**, *H. spiloheterus* **sp. n.**, *H. staryi* **sp. n.**, *H. stelfoxi* **sp. n.**, *H. strazanaci* **sp. n.**, *H. sumo* **sp. n.**, *H. szepligeti* **sp. n.**, *H. terrabas* **sp. n.**, *H. thereospilus* **sp. n.**, *H. tobiasi* **sp. n.**, *H. tolupan* **sp. n.**, *H. townesi* **sp. n.**, *H. trece* **sp. n.**, *H. tres* **sp. n.**, *H. tricolor* **sp. n.**, *H. trienta* **sp. n.**, *H. tuberculatus* **sp. n.**, *H. turrialbaensis* **sp. n.**, *H. tzutujil* **sp. n.**, *H. ugaldei* **sp. n.**, *H. uno* **sp. n.**, *H. variabilis* **sp. n.**, *H. veinte* **sp. n.**, *H. veintidos* **sp. n.**, *H. veintitres* **sp. n.**, *H. veintiuno* **sp. n.**, *H. vierecki* **sp. n.**, *H. villegasi* **sp. n.**, *H. vittatus* **sp. n.**, *H. vulcanus* **sp. n.**, *H. wahli* **sp. n.**, *H. warreni* **sp. n.**, *H. washingtoni* **sp. n.**, *H. wesmaeli* **sp. n.**, *H. whartoni* **sp. n.**, *H. whitfieldi* **sp. n.**, *H. wildi* **sp. n.**, *H. wilkinsoni* **sp. n.**, *H. wrightae* **sp. n.**, *H. xanthus* **sp. n.**, *H. xerxes* **sp. n.**, *H. xinca* **sp. n.**, *H. yaqui* **sp. n.**, *H. ypsilon* **sp. n.**, *H. zapotec* **sp. n.**, *H. zeus* **sp. n.**, *H. zitaniae* **sp. n.**, *H. zoque* **sp. n.**, *H. zunigai* **sp. n.**, *H. zurquiensis* **sp. n.** One new combination is proposed, *Pioscelus costaricensis* (Marsh) **comb. n.**

## Introduction

This is the second part of a two-part study of the braconid subfamily Doryctinae from Costa Rica. The first part (Marsh 2002) dealt with 62 genera and 172 species excluding the genus *Heterospilus* Haliday. The present study represents a comprehensive taxonomic study of the four genera of the tribe Heterospilini. This tribe is represented almost entirely by the hyperdiverse genus *Heterospilus* which includes 280 species, and three smaller genera which include 6 species. An expanded discussion of the family Doryctinae, a brief history of biodiversity of Hymenoptera in Costa Rica and significance of these studies, can be found in Marsh (2002). In addition to the morphological study, an interactive key to the species of *Heterospilus* was prepared using Lucid Builder.

Of the 280 species of *Heterospilus* described in this study, 277 are new to science. Furthermore, nearly 25% of the new species are described from a single unique holotype.

If the numerous unplaced species in the unsorted specimens we have looked at are added to the above figures, the enormous diversity of this genus in such a small locality becomes obvious. We estimate that perhaps another 50–100 species could be added to the total.

## Materials and methods

The morphological study is based on the examination of nearly 7,000 specimens of the genus *Heterospilus* and nearly 100 specimens of the other genera of the tribe Heterospilini from Costa Rica and neighboring countries in Central America. The majority of the specimens were borrowed from the University of Wyoming and the Instituto Nacional de Biodiversidad (INBio) in Costa Rica. Additional specimens were borrowed from the American Entomological Institute, the National Museum of Natural History, Washington, Texas A&M University, the Natural History Museum, London and the University of Costa Rica. Unfortunately, the specimens from INBio are not well mounted and it is difficult to see the characters on most of them. Thus, this study is based primarily on the material from the University of Wyoming. They are mostly from a long collaboration between Scott Shaw of the University of Wyoming and Paul Hanson of the University of Costa Rica and represent specimens collected over the past 30+ years by Paul as well as by Ian Gauld and Dan Janzen.

Below are the acronyms for the collections from which specimens were borrowed and where types have been deposited. Names of the responsible curators are indicated in brackets.

AEIC American Entomological Institute, Gainesville, FL [David Wahl]

ESUW Insect Museum, Ecosystem Science, University of Wyoming, Laramie, WY [Scott Shaw]

FSAG Faculté Universitaire des Sciences Agronomiques, Gembloux, Belgium [Yves Braet]

INBC Instituto Nacional de Biodiversidad, Santo Domingo, Costa Rica (frequently abbreviated as INBio) [Manuel Solís]

LEID National Museum of Natural History, Leiden, The Netherlands [Kees van Achterberg]

MICR Museo de Insectos, University of Costa Rica, San José, Costa Rica [Paul Hanson]

NHML The Natural History Museum, London [Gavin Broad]

NMNH National Museum of Natural History, Washington, DC [Robert Kula]

TAMU Department of Entomology, Texas A&M University, College Station, TX [Robert Wharton]

The specimens in this study were examined using a Wild M5 binocular stereomicroscope and fluorescent illumination. The scanning electron micrographs were made on a Philips XL30 Environmental Scanning Electron Microscope. Most specimens were gold/palladium coated using a Denton Desk II TSC turbo-pumped sputter coater, but the numerous unique holotypes were examined uncoated. Light microscope colored images were captured with a 3 MP Leica video camera running Leica Application Suite software and mounted on a Leica MZ6 stereomicroscope. Still images from the stereomicroscope were focus-stacked using Combine ZP (Hadley 2010). Minor levels of adjustments to both SEM and light images were performed in Adobe Photoshop versions CS3 and CS4, and plates were prepared in the same program.

An interactive key to species of Heterospilus from Costa Rica was created using Lucid Builder 3.5; it can be accessed at www.lucidcentral.org. We included 49 discrete morphological characters and one continuous character, antennal segment number, that varied among species and were diagnostically useful. Where possible, ambiguities and polymorphisms in state were resolved conservatively as coded to multiple states. The Lucid character matrix was examined in a concurrent phylogenetic study where evolutionary rate of each character over a 5-locus molecular phylogeny is correlated to taxonomic utility as measured by Lucid’s “best” function (Wild et al. 2013).

Label data for holotypes and paratypes is listed exactly as indicated on the labels including misspellings, punctuation, abbreviations and absent spaces. Lines on each label are separated by a bracketed semi-colon [;].

Taxonomic decisions were the sole responsibility of PMM and authorship of all new taxa is attributed to PMM.

### *Heterospilus* Character List and Definitions of Sculpturing

During this study, 49 discrete morphological characters for the 280 species of *Heterospilus* were used both in conjunction with the interactive key prepared with Lucid Builder (Wild et al. 2013) and in preparing the descriptions of the *Heterospilus* species and other Heterospilini genera provided here. An annotated discussion of these characters is provided below including illustrations of some surface sculpturing terms used in the descriptions. Most of the surface sculpturing terms follow Harris (1979) and Marsh (2002).

### HEAD

**Color.** The head color is either entirely yellow, entirely light or medium brown, or entirely dark brown or black. Often the head is bicolored with the face and vertex of contrasting colors.

**Flagellomere color.** The antennal flagellomeres of the Costa Rican species can be divided into three distinct groups: (1) flagellomeres entirely yellow, brown or black; (2) brown with the apical 3-8 flagellomeres white, the last one sometimes dark; (3) and brown with a white annulus of 3-6 flagellomeres below the apex, with the apical 3-5 flagellomeres always dark. Although this character easily divides the species into three groups, it was not used as a major separating character in the dichotomous keys below because many species had the antennae missing. But the character was used in the descriptions and where practical in the keys.

**Flagellomere number.** Most species have longer antennae with 25 or more flagellomeres, but a few species have short antennae with fewer than 20 flagellomeres.

**Scape color.** The scape is either yellow or brown. On a number of species there is a longitudinal brown stripe along the lateral edge.

**Figure 1.**
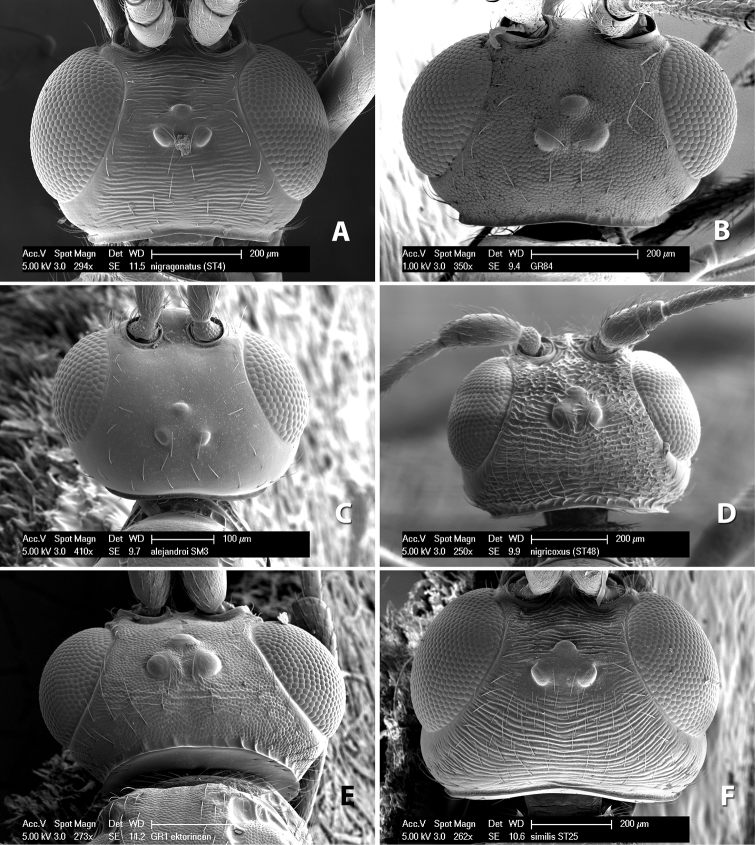
Examples of surface sculpturing in *Heterospilus* spp.

**Figure 2.**
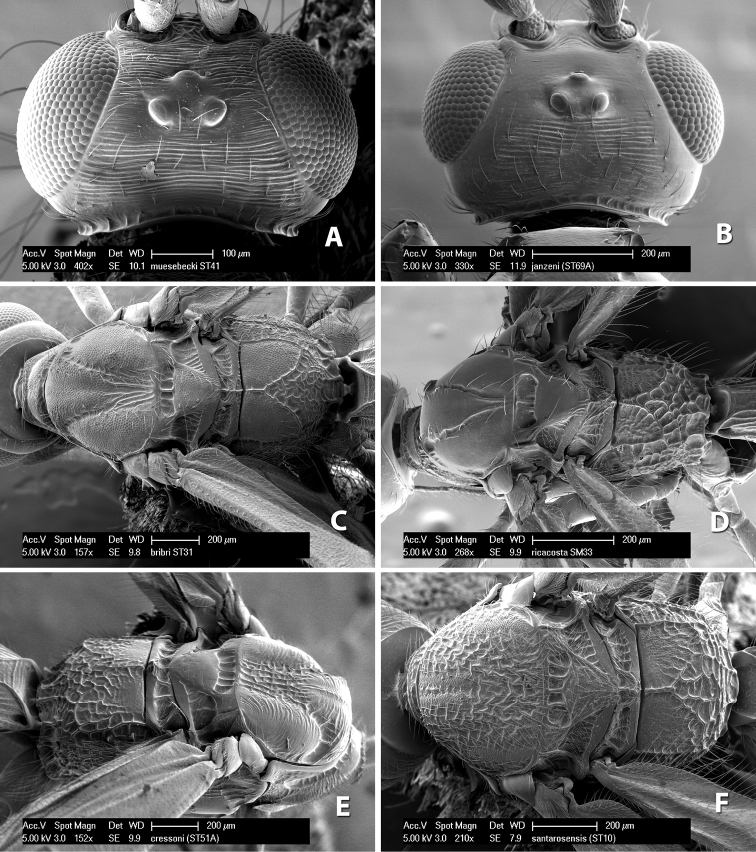
Examples of surface sculpturing in *Heterospilus* spp.

**Vertex sculpture.** The sculpture of the vertex divides the species of *Heterospilus* into three distinct groups: (1) smooth (Fig. 1C); (2) granulate (Fig. 1B); and (3) transversely striate or costate (Fig. 1A). Occasionally the sculpturing is costate-rugose (Fig. 1D) and often the granulate vertex will have more or less distinct transverse carinae behind the ocelli (Fig. 1E). While these characters are usually distinct, occasionally a smooth vertex will have a few weak carinae behind the ocelli in which case the species might be taken both ways through the keys.

**Frons sculpture.** The frons is usually sculptured the same as the vertex, smooth, costate/striate or granulate. However, occasionally the frons will be sculptured differently than the vertex, such as costate while the vertex is smooth.

**Face sculpturing.** The face is most often smooth, but can be variously sculptured striate, rugose, granulate or areolate.

**Malar space.** The malar space is the shortest distance from the base of the eye to the base of the mandible. The important character for *Heterospilus* is the distance in relation to 1/4 of the eye height - greater than, equal to or less than 1/4 of the eye height. In a few rare cases the malar space is so short that it appears to be absent, the base of the eye nearly touching the base of the mandible.

**Temple shape in dorsal view.** The temple, when viewed dorsally, is either broad and somewhat bulging behind the eye (Fig. 1F) or narrow and sharply sloping toward the occipital carina (Fig. 2A). Occasionally the temple will be broad but not bulging and thus sloping behind the eye (Fig. 2B).

**Temple width.** The width of the temple in relation to the eye width, in dorsal view, is important. The width is either greater than, equal to or less than 1/2 of the width of the eye.

**Ocell-ocular distance.** This is the shortest distance between the lateral ocellus and the edge of the eye and is compared to the diameter of the lateral ocellus. The ocell-ocular distance is 1.5 times or less, 1.5–2.5 times, or greater than 2.5 times the diameter of the lateral ocellus.

### MESOSOMA

**Color.** Most species have the mesosoma concolorous yellow, light to medium brown, or dark brown to black. Occasionally the mesosoma is bicolored, such as the mesoscutum lighter than the remainder of the mesosoma, the propodeum darker than the remainder, or the mesosoma with lateral darker stripes.

**Mesoscutal lobes sculpture.** The mesoscutal lobes are usually smooth (Fig. 2D) or granulate (Fig. 2C). Occasionally the lateral lobes are partially costate or striate (Fig. 2E) and rarely rugose along the notauli (Fig. 2F).

**Figure 3.**
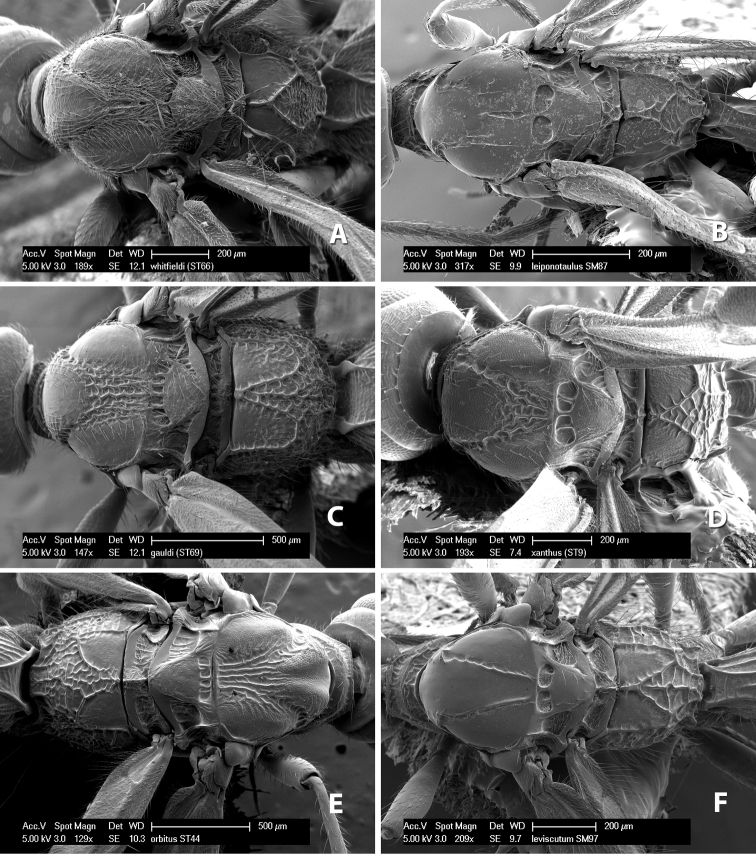
Examples of surface sculpturing in *Heterospilus* spp.

**Mesoscutal lobes pilosity.** Most species have sparse setae along the notauli (Fig. 2C), but a few have the lateral lobes entirely sparsely or densely hairy (Fig. 3A).

**Notauli sculpture.** The notauli for most species are entirely or sometimes partially scrobiculate (Fig. 2C). Occasionally they are smooth and unsculptured (Fig. 2D) or rarely weak or absent posteriorly (Fig. 3B).

**Notauli posterior meeting.** The notauli converge toward the midline of the mesoscutum and meet at the prescutellar furrow. This area where they meet is variously sculptured and is usually a triangular rugose (Fig. 3D), costate (Fig. 2C) or costate-rugose (Fig. 3E) area. Occasionally this is a wide rectangular rugose or costate area (Fig. 3C) or, rarely, an unsculptured area (Fig. 3F).

**Figure 4.**
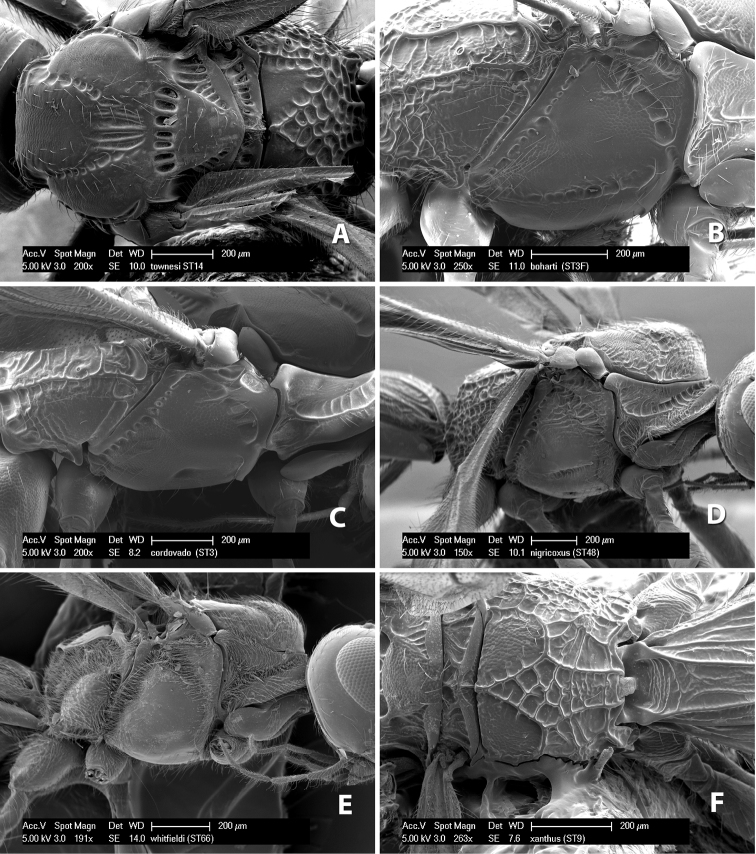
Examples of surface sculpturing in *Heterospilus* spp.

**Figure 5.**
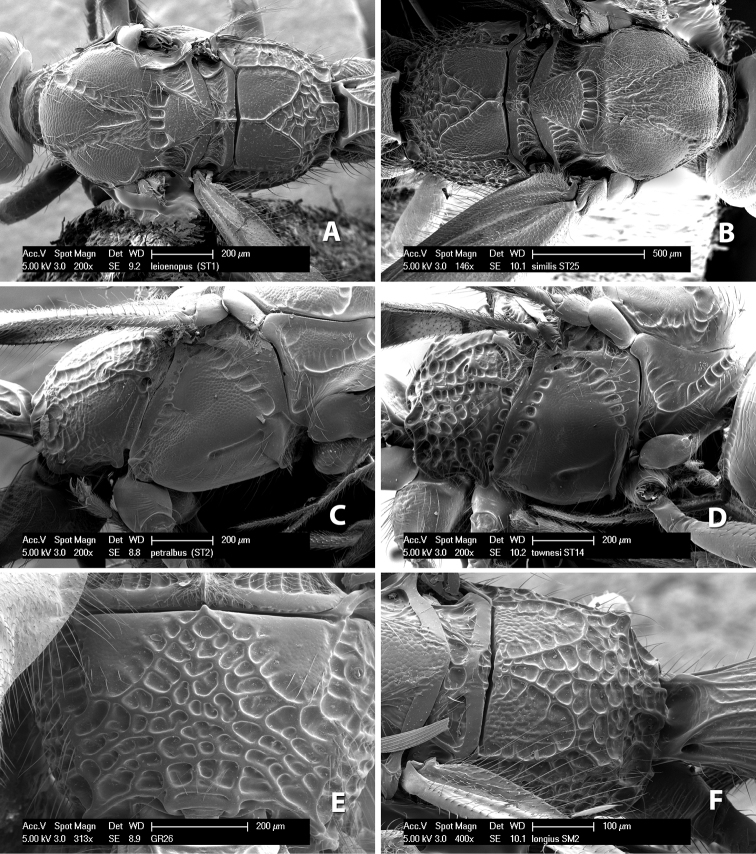
Examples of surface sculpturing in *Heterospilus* spp.

**Prescutellar furrow.** This furrow, sometimes called the scutellar sulcus (see Wharton et al. 1997: 34), is a broad transverse depression between the mesoscutum and scutellum and is divided by one median (Fig. 3B) or 3–5 (Figs 2C, 4A) cross carinae. Frequently there will be a distinct median cross carina and several weaker carinae on each side (Fig. 3F).

**Scutellum.** The scutellum is either smooth or granulate, and rarely weakly rugose.

**Mesopleuron sculpture.** In most species the mesopleuron is smooth (Fig. 4E) or granulate (Fig. 4B). In a few species it is smooth just above the precoxal sulcus and costate dorsally (Fig. 4C). Rarely the mesopleuron is mostly costate.

**Precoxal sulcus** (previously called the sternaulus; see Wharton 2006). The precoxal sulcus is always scrobiculate (Fig. 4B) or smooth (Fig. 4C) and usually shorter than the width of the mesopleuron. Occasionally it is extended to the posterior margin of the mesopleuron by a groove or carinae (Fig. 4B).

**Propodeum.** The propodeum has a distinct pattern of carinae (Fig. 4F) that is important in species identification (see Marsh 2002, fig. 5, for definitions of carinae and areas). The carinae and areas are discussed below.

**Propodeum - basal median area** . The basal median areas of the propodeum are usually distinct and entirely margined by carinae (Fig. 2C) but often the carinae are not distinct or absent (Fig. 2E). The surface sculpturing of the areas can be smooth (Fig. 3A), granulate (Fig. 2C) or rugose (Fig. 2D).

**Propodeum - basal median carina.** When this carina is present and distinct it is either long (usually longer than median carina of the prescutellar furrow) (Fig. 5A) or shorter than the prescutellar carina (Fig. 5B). Often the basal median carina is absent, thus the anterior tip of the areola meets the basal margin of the propodeum (Fig. 3C).

**Areola.** The areola is considered distinctly margined if it is enclosed anteriorly by the forked carinae at the apex of the basal median carina (sometimes referred to as the costulae) **and** posteriorly by converging carinae (Fig. 4F). It is considered not margined if the posterior carinae are absent (even if the costulae are present anteriorly) (Fig. 2C).

**Areolar sculpture.** This sculpturing is in the area within the areola, whether or not it is completely margined. The sculpturing is usually rugose (Fig. 2C) but often it is areolate (Fig. 5E) or areolate-rugose (Fig. 4A). Rarely this area is smooth (Fig. 3A).

**Propodeum - lateral area.** The propodeum laterally is usually entirely rugose (Fig. 5D) or rarely smooth. Often the lateral area is rugose posteriorly and granulate or smooth anteriorly (Fig. 5C).

**Propodeum - apical lateral corners.** Occasionally the apical-lateral corners of the propodeum are produced into a more or less distinct tubercle (Fig. 5F).

**Figure 6.**
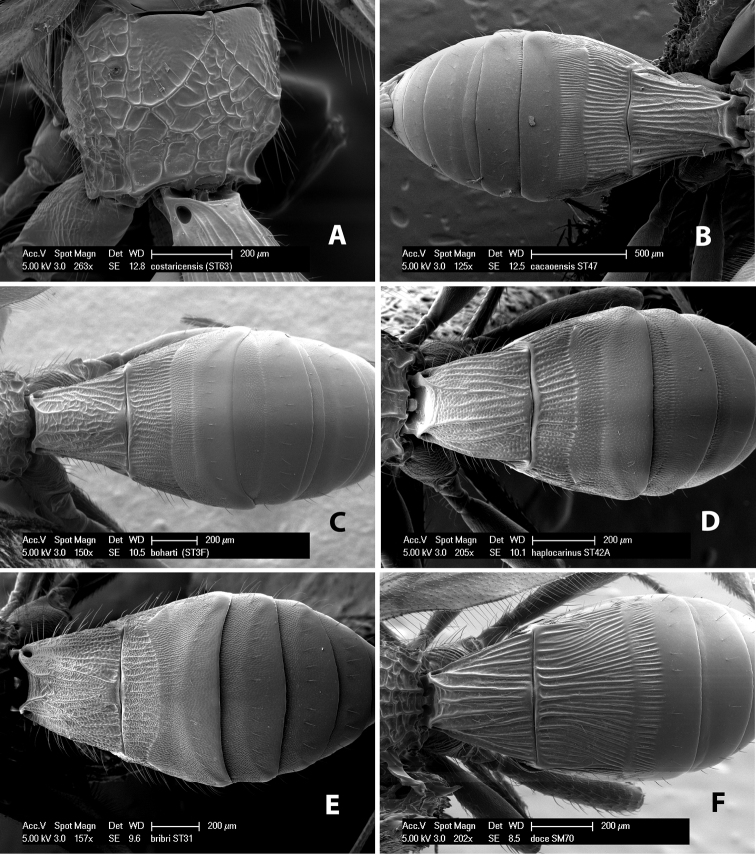
Examples of surface sculpturing in *Heterospilus* spp.

**Propodeum - area just above hind coxae.** Occasionally the carinae on the lateral-posterior area of the propodeum are produced into a raised tubercle just above the hind coxa (Fig. 6A).

**Venter of mesosoma.** The venter of the mesosoma is granulate or smooth, usually similar to the sculpturing on the mesopleuron.

### WINGS (Fig. 8A–B)

**Fore wing - relative lengths of veins r and 3RSa.** Fore wing vein r is usually shorter than vein 3RSa, but occasionally vein r is as long as or shorter than vein 3RSa.

**Fore wing - position of vein 1cu-a.** Vein 1cu-a is either interstitial with vein 1M or beyond vein 1M by a short distance.

**Stigma color.** The fore wing stigma is usually concolorous brown or yellow but is occasionally bicolored brown with yellow spot at apex and/or base.

**Stigma width.** The width of the stigma is usually greater than the length of vein r but occasionally it is equal or shorter than vein r.

**Hind wing - vein SC+R.** Vein SC+R is either present or absent. These two states are about equally divided among the species of *Heterospilus* in Costa Rica.

**Hind wing – relative lengths of veins M+CU and 1M.** These veins are usually about equal in length, but vein M+CU is occasionally shorter or longer than vein 1M.

### LEGS

**Leg color.** The legs are usually concolorous yellow, brown or black. Frequently the femur and/or tibia are bicolored and occasionally the coxae are differently colored than the femur.

### METASOMA

**First tergum (petiole) - length relative to apical width.** The length of the first tergum is usually equal to its apical width but often greater than the apical width or rarely less than the apical width.

**Figure 7.**
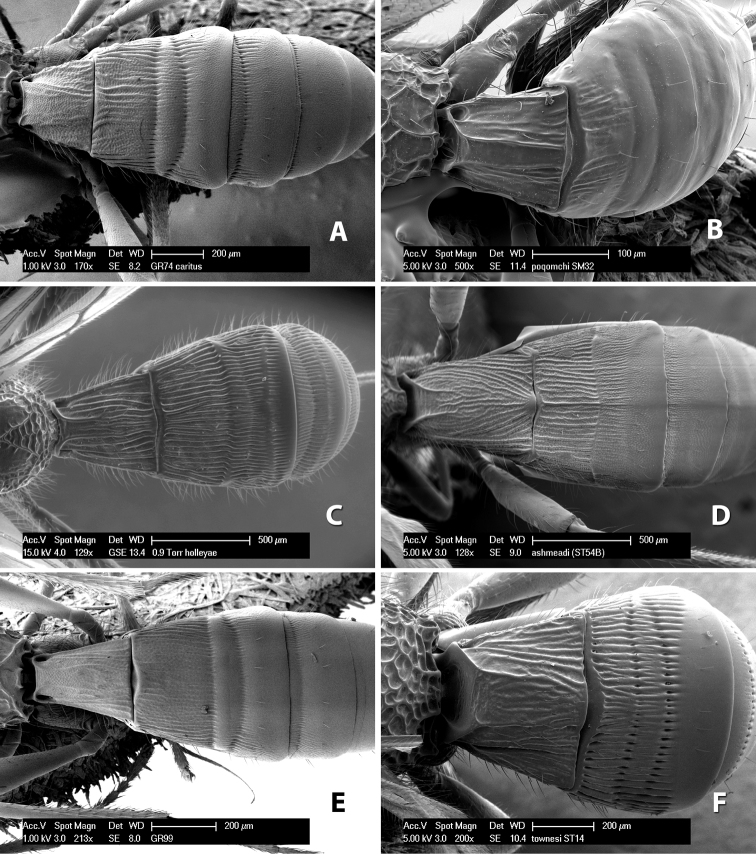
Examples of surface sculpturing in *Heterospilus* spp.

**First tergum - sculpture.** The first tergum is nearly always longitudinally costate (Fig. 6B) or porcate (Fig. 6F (porcate is defined as with fewer broad ridges than in costate and with deep broad sulcations between ridges)). Occasionally it can be more or less rugose, at least medially (Fig. 6C) and often it can be granulate (Fig. 6D) or rugose (Fig. 6E) between the costae. Rarely the tergum is entirely granulate (Fig. 7E) or partially or entirely smooth (Fig. 7A).

**Figure 8.**
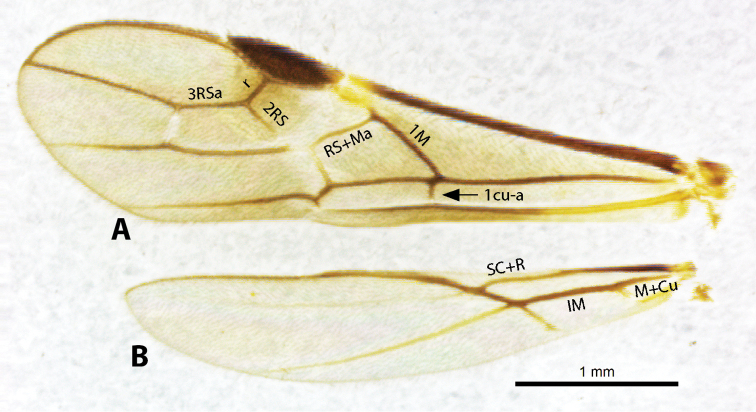
Wings of *Heterospilus* showing relevant veins.

**First tergum - color.** The first tergum is nearly always concolorous yellow, brown or black. Rarely the apical edge is lighter or darker than the remainder of the tergum.

**Second tergum - sculpture.** The second tergum is usually longitudinally costate or striate (Fig. 6F) but occasionally it is entirely granulate or, rarely, smooth.

**Second tergum - width.** The width is usually three or less times median length (Fig. 6F) but often it is narrower and four or more times median length (Fig. 6E).

**Second tergum - color.** The color is usually concolorous yellow, brown or black. Frequently the tergum is bicolored.

**Third tergum - sculpture.** The third tergum is variously sculptured as follows: longitudinally costate entirely (Fig. 7C); granulate entirely (Fig. 6E); costate at base, smooth at apex (Fig. 6B); costate at base, granulate at apex (Fig. 7D); granulate at base, smooth at apex (Fig. 6C); smooth entirely.

**Anterior and posterior transverse grooves.** In the *Heterospilus* species from Costa Rica the fused metasomal terga 2+3 always have two transverse grooves (Fig. 7A). The anterior transverse groove separates the second and third terga whereas the posterior transverse groove is on the basal third or fourth of the third tergum. Both grooves are usually costate or scrobiculate (Fig. 7E) and often deeply pitted (Fig. 7F). Rarely, one or both of the grooves is absent. The grooves are usually straight, but the anterior groove is often more or less sinuate (Fig. 6E).

**Metasomal terga 4-7 - sculpture.** These terga are nearly always granulate or smooth but occasionally terga 4 and 5 will be costate or granulate only at the base, smooth apically.

**Metasomal terga 4-7 - color.** These terga are usually concolorous with the anterior terga, but often they are lighter and distinctly contrasting in color with the anterior terga.

**Ovipositor length.** This character refers to the exposed length of the ovipositor beyond the apex of the metasoma relative to the length of the metasoma. The states of this character are: shorter than first metasomal tergum; equal to length of first tergum; equal to length of terga 1 and 2 combined; equal to 1/2 metasoma length; equal to 3/4 metasomal length; equal to length of metasoma; longer than metasoma.

## Taxonomy

### Subfamily Doryctinae

Foerster

Doryctinae Foerster, 1862: 227, 238 (as Doryctoidae).

#### Diagnosis

(taken from Marsh 2002). Cyclostome braconids with circular or oval mouth opening formed by concave apical margin of clypeus, mandibles (when closed) and concave labrum; occipital carina usually present, rarely absent; fore tibia with row or (rarely) cluster of stout, short spines along anterior edge distinct from regular setae; epicnemial carina present; double node at apex of dorsal valve of the ovipositor; flange at the apico-lateral corner of the propleuron just above the fore coxa and extending slightly over ventro-lateral corner of pronotum.

Species now included in the Doryctinae were first described by Linnaeus and Fabricius in the genus *Ichneumon*. Many of these species and subsequently described new species were transferred to the genus *Bracon* Fabricius (1804). In 1808, Spinola separated from this genus what he called the *Bracon petiolatus* group which was later described as the genus *Spathius* by Nees (1818). This is apparently the first genus to be later included in the Doryctinae.

Foerster, in his monumental study of braconid classification, divided the Braconidae into several groups which later became subfamilies, tribes and subtribes (Foerster 1862). He included doryctine genera in groups he called Doryctoidae, Euspathioidae and Hecaboloidae. Forester was the first to use the name Doryctinae (-oidae) and is considered the author of the subfamily name.

### Tribe Heterospilini

Fischer

Heterospilini Fischer, 1981: 47 (as subtribe Heterospilina).

#### Diagnosis.

Fore wing vein 2RS absent (Fig. 8A), at least partially, rarely indicated by infuscated line (nebulose vein) but not a distinct tubular vein; fore wing first subdiscal cell open at apex, vein 2cu-a absent (Fig. 8A); basal sternal plate (acrosternite) of first metasomal segment 1/4 length of tergum; hind coxa usually with antero-ventral tooth or tubercle at base (Fig. 10C), occasionally with weak tooth; hind wing of male usually with enlarged stigma near base.

The complete or near absence of the fore wing vein 2RS occurs in several other tribes of the Doryctinae. Belokobylskij (2006) presented a key to the World tribes and genera of Doryctinae with a reduced fore wing vein 2RS. A few apterous or brachypterous species of *Heterospilus* have been described including one from Costa Rica (Kula 2013) and two in the Nearctic Region (Kula 2011). In the present study of nearly 7,000 specimens, no apterous or brachypterous forms were found. Most of the specimens were collected in Malaise traps or by sweep nets but the specimens that were described by Kula were collected in pan traps. Undoubtedly, if more collecting in Costa Rica were to be done using pan traps, more specimens of wingless species would be found.

In Costa Rica, the tribe Heterospilini is composed almost entirely of the speciose genus *Heterospilus* but also includes three smaller genera which can be identified by the key below.

Very little is known about the biology of the species in the Heterospilini. The genus *Heterospilus* has the most diverse host range within the Doryctinae. Where records are known, species in *Heterospilus* parasitize a very wide range of endophytic, mostly stem-boring, hosts. In the Coleoptera, *Heterospilus* parasitizes the families Anobiidae, Bostrichidae, Bruchidae, Buprestidae, Cerambycidae, Curculionidae, Languriidae, Mordellidae and Scolytidae; in the Lepidoptera, the families Gelechiidae, Incurvariidae, Pyralidae and Tortricidae are utilized; and two Mexican species were reared from the cotton boll weevil (Marsh 2002). In Costa Rica, even less is known about the hosts of *Heterospilus* relative to the large number of species. One species, *Heterospilus microstigmi* Richards, has been reared from larvae of pemphredonine sphecid wasps of the genus *Microstigmus* (Marsh and Melo 1999); another species, *Heterospilus lapierrei* sp. n., was reared from a weevil infesting *Cecropia* (Hespenheide and LaPierre 2006); and *Heterospilus sinuatus* sp. n. has been reared from several species of bruchids (Whitehead 1975). One record of *Heterospilus* attacking streblid bat flies needs to be confirmed (Shockley and Murray 2006).

#### Key to the Genera of Heterospilini from Costa Rica

**Table:** 

1	Ovipositor curved up at apex and considerably modified (Fig. 9A)	*Neoheterospilus* Beleokobylskij
–	Ovipositor straight at apex, not modified	2
2(1)	Hind coxa without a distinct antero-ventral basal tubercle or tooth (Fig. 10D)	*Pioscelus* Muesebeck & Walkley
–	Hind coxa with a distinct antero-ventral basal tubercle or tooth Fig. 10C)	3
3(2)	Vertex sharply angled laterally near upper eye margin (Fig. 9B)	*Paraheterospilus* Marsh, gen. n.
–	Vertex not sharply angled laterally near eye	*Heterospilus* Haliday

### Genus Neoheterospilus

Belokobylskij

http://species-id.net/wiki/Neoheterospilus

Neoheterospilus Belokobylskij, 2006: 151.

#### Type species.

*Heterospilus falcatus* Marsh.

#### Diagnosis.

Small size, 2.5–3.0 mm; ovipositor curved up and modified apically with valves expanded and sickle-shaped and sheaths expanded; fore wing vein 2RS absent, vein r slightly longer than vein 3RSa, first subdiscal cell open at apex and bottom, vein 2-1A absent; hind wing vein SC+R absent, hind wing of male with stigma.

#### Distribution.

Neotropical, Palaearctic, Oriental, Australian and Afrotropical Regions.

#### Biology.

One species has been reared from Scolytidae.

#### Comments.

This genus is distinguished from all other heterospiline braconids by the unusual shape of the ovipositor that is curved up and sickle-shaped at the tip. Belokobylskij 2006 presents a key and descriptions to the world species and separates them into two subgenera. The Neotropical Region contains one described species occurring in Costa Rica (new record), Venezuela and Brazil.

### Neoheterospilus (Harpoheterospilus) falcatus

(Marsh)

http://species-id.net/wiki/Neoheterospilus_falcatus

Fig. 9A

Heterospilus falcatus Marsh, in Quicke and Marsh 1992: 563.
Neoheterospilus (Harpoheterospilus) falcatus (Marsh); Belokobylskij 2006: 175.

#### Female.

Body size: 2.5–3.0 mm. Color: body dark brown, apical metasomal terga slightly lighter; scape yellow, flagellum brown; wing veins including stigma brown; legs yellow. Head: vertex weakly granulate; frons weakly granulate; face rugose-granulate; temple in dorsal view narrow, sloping behind eye, width less than 1/4 eye width; malar space about 1/4 eye height; ocell-ocular distance 1.5 times diameter of lateral ocellus; 22–25 flagellomeres. Mesosoma: mesoscutal lobes granulate; notauli scrobiculate, meeting posteriorly in triangular rugose area; scutellum granulate; prescutellar furrow with numerous cross carinae; mesopleuron weakly granulate; precoxal sulcus scrobiculate, shorter than mesopleuron; venter weakly granulate; propodeum with basal median areas margined, granulate, basal median carina absent, areola not margined, areolar area rugose, lateral area nearly entirely granulate. Wings: fore wing vein r slightly longer than vein 3RSa, vein 1cu-a beyond vein 1M; hind wing vein SC+R absent, vein M+CU shorter than vein 1M. Metasoma: first tergum longitudinally costate, length greater than apical width; second tergum longitudinally costate on basal half and smooth apically, occasionally entirely costate; anterior transverse groove very weak or absent; posterior transverse groove absent; third and following terga smooth; ovipositor longer than metasoma.

#### Male.

Essentially as in female; hind wing with stigma.

#### Specimens examined.

Costa Rica: Alajuela [;] Est. San Ramon [;] Light trap UV/F in [;] clearing off main road [;] to station. 21.vi.1998 [;] Dadelahi & Zitani (ESUW). COSTA RICA: Alajuela [;] Chiles de Aquas [;] Zarcas Café, 300m [;] i.1990, R. Cespedes (SUW). COSTA RICA: Heredia [;] Pr: La Selva Biol. Sta. [;] 3km S. Pto. Viejo [;] 10°26'N, 84°01'W [;] 19.v.1990 [;] H.A. Hespenheide [;] on dead Citrus (ESUW). Costa Rica: Heredia [;] 3km. S. Puerto Viejo [;] OTS - La Selva, 100m [;] 16–30.IX.1992 [;] P. Hanson (ESUW). Costa Rica, Puntarenas [;] R.F. Golfo Dulce, 5km. [;] W. Piedras Blancas, 100m [;] I-1993, P. Hanson (ESUW). Costa Rica: Puntarenas [;] Pen. Osa, 5km. N. Pto. [;] Jimenez, 10m, iii-iv. [;] 1991, P. Hanson, Malaise (ESUW). PN.Manuel Antonio,80m [;] Quepos, Prov. Puntarenas [;] Costa Rica, Nov 1992, [;] G. Varela [;] L-S 370900,448800 (INBC). Playuelas, R.N.V.S. Caño Negro, Prov. [;] Alaju, COSTA RICA, 20m, 3-21 Ene 1994 [;] K. Martinez, L N 325900_454500 #2580 (INBC).

#### Biology.

Specimens of the type series from Venezuela were reared from *Xyleborus ferrugineus* (F.).

**Figure 9.**
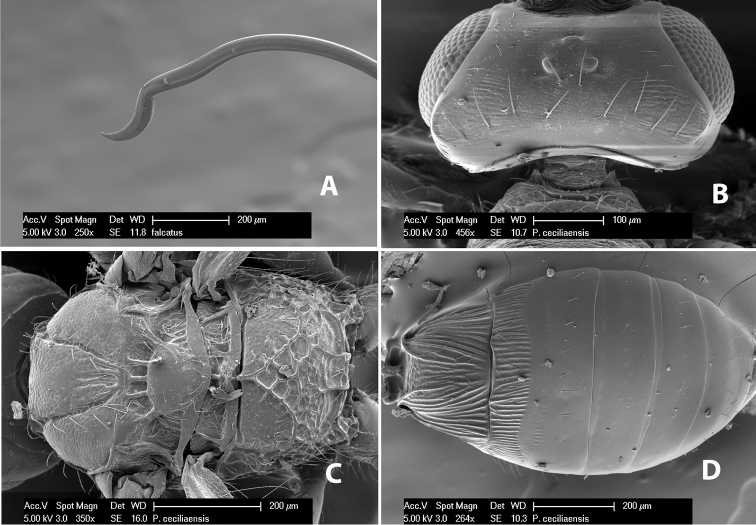
**A** apex of ovipositor, *Neoheterospilus falcatus* (Marsh) **B–D** *Paraheterospilus ceciliaensis* Marsh, sp. n.

### Genus Paraheterospilus

Marsh gen. n.

http://zoobank.org/1319B887-7BC5-4D47-84F1-FC23FB71E8CE

http://species-id.net/wiki/Paraheterospilus

#### Type species.

*Paraheterospilus ceciliaensis* Marsh, sp. n.

#### Diagnosis.

Small size, 1.5–2.0 mm; vertex sharply angled dorso-laterally near dorsal eye margin (Fig. 9B); occipital carina meeting hypostomal carina; fore tibia with single row of short spines along anterior edge; hind coxa with small but distinct antero-ventral basal tooth; basal sternal plate of metasomal segment 1 short, less than 1/4 length of tergum, laterope indistinct; fore wing vein r-m present, vein 2RS absent; hind wing vein SC+R absent.

#### Distribution.

Know only from Costa Rica.

#### Biology.

Unknown.

#### Comments.

This genus is very similar to *Heterospilus* but is distinguished from this and all other Doryctinae genera by the unusual angled dorso-lateral corners of the vertex.

#### Etymology.

The generic name is from the Greek *para*, meaning near, in reference to its similarity with the genus *Heterospilus*. Gender is masculine.

#### Key to the species of *Paraheterospilus* in Costa Rica

**Table:** 

1	Ovipositor longer than metasoma	*Paraheterospilus eumekus* Marsh, sp. n.
–	Ovipositor shorter than metasoma	2
2(1)	Width of metasomal tergum 2 about 4 times median length; propodeum sharply declivous posteriorly	*Paraheterospilus ceciliaensis* Marsh, sp. n.
–	Width of metasomal tergum 2 about 3 times median length; propodeum horizontal, not declivous posteriorly	*Paraheterospilus wilbotgardus* Marsh, sp. n.

### Paraheterospilus ceciliaensis

Marsh sp. n.

http://zoobank.org/C7CBB7C1-D844-42F5-B1D4-808D35AB2AB4

http://species-id.net/wiki/Paraheterospilus_ceciliaensis

Figure 9B–D

#### Female.

Body size: 1.5 mm. Color: body dark brown, metasoma often partially or completely lighter brown; scape and pedicel yellow, flagellum brown; legs yellow; wing veins, including stigma, brown, stigma rarely yellow at apex. Head: vertex usually smooth, often weakly transversely striate behind ocelli, dorsal-lateral corners sharply angled at upper eye margin; frons smooth; face weakly striate; temple in dorsal view narrow, sloping behind eye, width less than 1/2 eye width; malar space about 1/4 eye height; ocelli small, ocell-ocular distance about 4 times diameter of lateral ocellus; 15 flagellomeres. Mesosoma: mesoscutal lobes granulate; notauli shallow, scrobiculate, meeting posteriorly in small triangular costate area; scutellum usually smooth, occasionally weakly granulate; prescutellar furrow with 3–5 cross carinae; mesopleuron weakly granulate, often smooth just above precoxal sulcus; precoxal sulcus weakly scrobiculate or smooth, shorter than mesopleuron; venter smooth; propodeum sharply declivous posteriorly, dorsal surface short, basal median areas margined but short, granulate, basal median carina absent, areola not distinct, areolar area rugose, lateral areas entirely rugose. Wings: fore wing vein r about 1/2 length of vein 3RSa, vein 1cu-a beyond vein 1M; hind wing vein SC+R absent, vein M+CU equal to or slightly shorter than vein 1M. Metasoma: first metasomal tergum longitudinally costate, raised median area margined, declivous anteriorly and transversely costate, length of tergum about equal to apical width; second metasomal tergum longitudinally costate, width about 4 times median length; anterior transverse groove slightly sinuate; posterior transverse groove very weak or absent; third and following terga smooth; ovipositor slightly less than 1/2 length of metasoma.

#### Male.

Essentially as in female; width of second metasomal tergum about 3 times apical width; hind wing with stigma.

#### Holotype female:

Top label (white, printed) - Est. Pitilia, 700m, 9km S [;] Sta. Cecilia, P.N.Guana- [;] caste, Prov. Guan. COSTA [;] RICA, D.Garcia, 4–14 nov [;] 1991, L.N.330200–880200; second label (white, printed) - INBio bar code; third label (red, printed) - HOLOTYPE [;] *Paraheterospilus* [;] *ceciliaensis* Marsh. Deposited in INBC.

#### Paratypes.

7 ♀♀, 4 ♂♂, same data as holotype with additional collectors P.Rios and C. Moraga, and dates Ago 1991, 23 set a 14 oct 1992, 10 a 17 jun, 31 mar a 15 abr 1992, 3–18 Oct 1991 and 4-25 Nov 1991 (INBC). 1 ♀, 2 ♂♂, Costa Rica: Puntarenas [;] R.F. Golfo Dulce, 5km, [;] W. Piedras Blancas, 100m [;] vi-vii.1991, viii-ix.1991 and xi-xii.1991, P. Hanson [;] Malaise nr. second growth (ESUW). 1 ♂, Costa Rica: Guanacaste [;] P.N. Guanacaste [;] below Pitilia, 500m [;] 7–8.iii.1990, J.S. Noyes (ESUW). 1 ♀, COSTA RICA, Heredia [;] Chilamate, 75m [;] 25/III/1989 [;] col. Hanson & Godoy (ESUW). 1 ♀, PANAMA: Colon Pr. [;] Rio Guanche, 5km [;] S Portobelo, el. 100 ft. [;] 9°30.202'N, 79°39.903'W [;] 18.vii.1999, J. Wooley (TAMU).

#### Comments.

This species differs from *Paraheterospilus eumekus* and *Paraheterospilus wilbotgardus* in its shorter and squat body, the shorter metasomal tergum 2 and the shorter antenna.

#### Etymology.

Named for the locality where most of the type series was collected, Cecilia Station in Guanacaste National Park.

### Paraheterospilus eumekus

Marsh sp. n.

http://zoobank.org/5CAE39E6-B047-4F0A-8DE0-679FDC1246F1

http://species-id.net/wiki/Paraheterospilus_eumekus

#### Female.

Body size: 2.0 mm. Color: body dark brown; scape and pedicel yellow, flagellum brown; legs yellow; wing veins including stigma brown. Head: vertex smooth, dorsal-lateral corners sharply angled; frons smooth; face weakly striate or striate-granulate; temple in dorsal view narrow, sloping behind eye, with less than 1/2 eye height; malar space about 1/4 eye height; ocelli small, ocell-ocular distance nearly 4 times diameter of lateral ocellus; 16 flagellomeres. Mesosoma: mesoscutal lobes granulate; notauli shallow, scrobiculate, meeting posteriorly in small triangular costate area; scutellum usually smooth, occasionally weakly granulate; prescutellar furrow with 3–5 cross carinae; mesopleuron weakly granulate, often smooth just above precoxal sulcus; precoxal sulcus weakly scrobiculate or smooth, shorter than mesopleuron; venter smooth; propodeum sharply declivous posteriorly, dorsal surface short, basal median areas margined but short, granulate, basal median carina absent, areola not distinct, areolar area rugose, lateral areas entirely rugose. Wings: fore wing vein r shorter than vein 3RSa, vein 1cu-a interstitial with or slightly beyond vein 1M, stigma short and broad, width more than twice length of vein r; hind wing vein SC+R absent, vein M+CU equal to or slightly shorter than vein 1M. Metasoma: first tergum longitudinally costate, length slightly greater than apical width; second tergum longitudinally costate, width about 3 times median length; third tergum smooth, anterior and posterior grooves absent; terga 4–7 smooth; ovipositor longer than metasoma, often as long as body.

#### Male.

Unknown.

#### Holotype female.

Top label (white, printed) - COSTA RICA, Prov. Puntarenas, [;] Est. Rio Bonito, 2.3Km ai O. del [;] Cerro la Gamba, 110m, 9–26 MAR [;] 1996, E. Fletes. [;] L_S_293900_547075 #8308; second label - INBio bar code; third label (red, printed) - HOLOTYPE [;] *Paraheterospilus* [;] *eumekus* Marsh. Deposited in INBC.

#### Paratypes.

1 ♀, Est. Pitilia, 700m, 9km S [;] Sta. Cecilia, P.N. Guana- [;] caste, Prov. Guanacaste, [;] Costa Rica, 27 jui a 14 [;] ago 1992, P.Rios [;] L-N 830200,380200 (INBC). 1 ♀, top label - Costa Rica: Puntarenas [;] A.C.O., Golfito, Reserva [;] Forestal Golfo Dulce [;] Est. Agujas, 250–350m; second label - 2–22 October 1999 [;] J. Azofeifa, Red de Golpe [;] L-S-276750–526550 #53491 (ESUW).

#### Comments.

The very long ovipositor, often as long as the body, will distinguish this species from the others in the genus.

#### Etymology.

The specific name is from the Greek *eumekes*, meaning of great length, in reference to the long ovipositor.

### Paraheterospilus wilbotgardus

Marsh sp. n.

http://zoobank.org/1E49AB37-0E20-469B-AB38-DAE3FC8DAAA5

http://species-id.net/wiki/Paraheterospilus_wilbotgardus

#### Female.

Body size: 1.5–2.0 mm. Color: body dark brown, apical metasomal terga often lighter; scape and pedicel yellow, flagellum brown; legs yellow; wing veins including stigma light yellow. Head: vertex weakly granulate, dorsal-lateral corners sharply angled; frons weakly granulate; face granulate; temple in dorsal view somewhat broad and not sloping behind eye, width equal to 1/2 eye width; malar space about 1/4 eye height; ocell-ocular distance about 4 times diameter of lateral ocellus; 20 flagellomeres. Mesosoma: mesoscutal lobes granulate; notauli scrobiculate, meeting posteriorly in large triangular costate area; scutellum weakly granulate; prescutellar furrow with 3–5 cross carinae; mesopleuron granulate; precoxal sulcus weakly smooth, shorter than mesopleuron; venter granulate; propodeum nearly horizontal, only gradually sloping posteriorly, basal median areas distinctly margined, granulate, basal median carina present, short, areola distinct, areolar area broadly areolate-rugose, lateral areas rugose posteriorly, granulate anteriorly. Wings: fore wing vein r shorter than vein 3RSa, width of stigma slightly greater than length of vein r, vein 1cu-a beyond vein 1M; hind wing vein SC+R absent, vein M+CU shorter than vein 1M. Metasoma: first metasomal tergum longitudinally costate-granulate, length slightly greater than apical width; second tergum longitudinal costate, width about 3 times median length; anterior transverse groove present, straight; posterior transverse groove absent; third tergum costate basally, smooth apically; terga 4–7 smooth; ovipositor half as long as metasoma.

#### Male.

Unknown.

#### Holotype female.

Top label (white, printed) - Costa Rica: Puntarenas [;] San Vito, Las Cruces [;] Wilson Botanical Gardens [;] 18-22.iii.1990, 1150m [;] J.S. Noyes; second label (red, printed) - HOLOTYPE [;] *Paraheterospilus* [;] *wilbotgardus* Marsh. Deposited in (ESUW).

#### Paratypes.

1 ♀, same data as holotype (ESUW).

#### Comments.

This species is distinguished from the others in the genus by the more coarsely granulate head and mesosoma and the more horizontal propodeum.

#### Etymology.

The specific name is in reference to the type locality of the Wilson Botanical Gardens in Puntarenas Province.

### Genus Pioscelus

Muesebeck & Walkley

http://species-id.net/wiki/Pioscelus

Pioscelus Muesebeck & Walkley 1951: 180.
Amazondoryctes Barbalho & Penteado-Dias, in Barbalho et al. 1999: 142. Synonymized by Belokobylskij 2006: 150.

#### Type species.

*Hedysomus wichitus* Viereck.

#### Diagnosis

**Figure 10.**
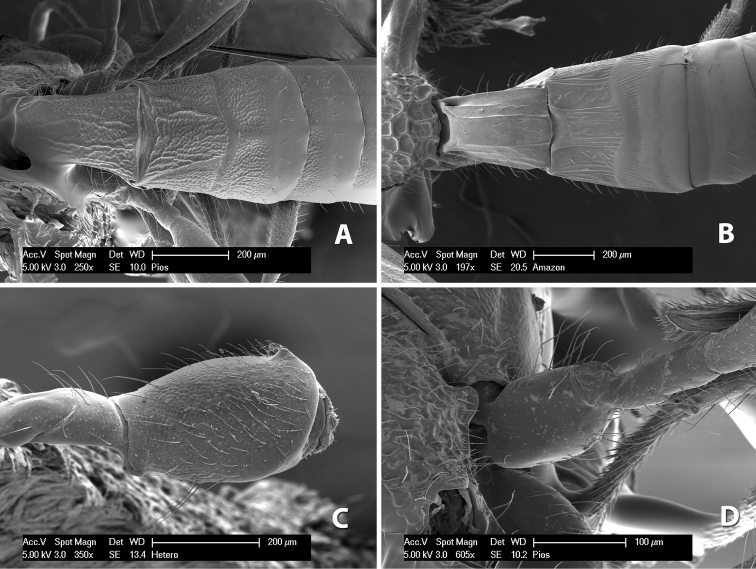
**A** *Pioscelus* sp. showing typical sculpturing on the second metasomal tergum **B** *Pioscelus* sp. showing the *Amazondoryctes* style of sculpturing on the second metasomal tergum **C** hind coxa of *Heterospilus* sp. **D** hind coxa of *Pioscelus* sp.

(taken from Marsh 2002). Body size: 2.5–4.5 mm; head cubicle; occipital carina meeting hypostomal carina; frons slightly excavated; mesoscutum declivous anteriorly; propodeum often horizontal and barely declivous posteriorly; precoxal sulcus usually as long as mesopleuron; fore tibia with single row of short spines along anterior edge; hind coxa without distinct tooth or tubercle at base (Fig. 10D) although smoothly angled; fore wing vein r-m present, vein 2RS absent or represented partially by weakly infuscate line, first subdiscal cell open at apex; hind wing vein M+CU equal to or shorter than vein 1M; first metasomal tergum longer then apical width; second metasomal tergum with either 2 converging median grooves which set off triangular area (Fig. 10A) or 2 median parallel carinae (Fig. 10B); basal sternal plate of first metasomal segment 0.25–0.33 length of tergum, dorsope distinct or weak.

#### Distribution.

Nearctic and Neotropical Regions.

#### Biology.

Unknown.

#### Comments.

The genus *Amazondoryctes* was synonymized with *Pioscelus* by Belokobylskij (2006). In the molecular analysis (Wild et al. 2013) specimens identified as *Amazondoryctes* emerged basally within *Heterospilus* but no specimens identified as *Pioscelus* were available for analysis. Thus, the exact placement of these two genera within the Heterospilini is still in question.

Two species occur in Costa Rica, *Pioscelus mesomphalus* Marsh and *Pioscelus costaricensis* (Marsh), comb. n. Descriptions for each species can be found in Marsh 2002.

### Genus Heterospilus

Haliday

http://species-id.net/wiki/Heterospilus

Heterospilus Haliday, 1836: 40.
Telebolus Marshall, 1888: 202. Synonymized by Muesebeck and Walkley 1951.
Kareba Cameron, 1904: 50. Synonymized by Marsh 1973.
Anacatostigma Enderlein, 1920(1918): 131. Synonymized by Marsh 1973.
Harpagolaccus Enderlein, 1920(1918): 138. Synonymized by Belokobylskij 1992.

#### Type species.

*Rogas (Heterospilus) quaestor* Haliday.

#### Diagnosis

(taken from Marsh 2002). Small to median size, 2.0–5.0 mm; head usually cubical, malar space varying from 1/6 to 1/3 eye height; occipital carina meeting hypostomal carina; mesoscutum usually declivous anteriorly; propodeum usually with distinct basal median areas and areola; first metasomal tergum varying from wider at apex than length to length twice apical width, basal sternal plate of first tergum 1/4 length of tergum; ovipositor varying from barely visible to longer than body; fore tibia with single row of short stout spines along anterior edge; hind coxa with small but distinct basal tubercle or tooth; fore wing vein r-m present, vein 2RS usually absent, sometimes represented by infuscate line (nebulous vein) but not a distinct tubular vein, first subdiscal cell open at apex; hind wing vein M+CU varying from shorter to longer than vein 1M; hind wing of male always with a stigma.

#### Distribution.

The genus occurs worldwide but the preponderance of species are found in the Western Hemisphere.

#### Biology.

There is very little biological information about species of *Heterospilus*, but where records exist, species are recorded as parasitoids of wood-boring beetle larvae, particularly Scolytidae, but a few species have been reared from Bruchidae and Curculionidae as well as from stem-boring sawflies and moths. Several species have been reared from nests of *Microstigmus* (Sphecidae) in Costa Rica (Marsh and Melo 1999).

#### Comments.

The identification of the genus *Heterospilus* has been firm for many years but the exact location and recognition of the type specimen has long been in question. However, van Achterberg (1997) made an extensive review of the Haliday collection of Braconidae at the Irish National Museum in Dublin, Ireland in which he attempted to “...recognize types, to label them and to re-identify the taxa described by Haliday according to modern insights.” Thus, the type specimen of *Heterospilus quaestor* Haliday was identified and re-labeled in the collection. Although van Achterberg presented a re-description of the type specimen, I have also presented a description below.

### Rogas (Heterospilus) quaestor

Haliday

http://species-id.net/wiki/Rogas_quaestor

#### Description.

Description of female lectotype designated by van Achterberg 1997.

Borrowed from Irish National Museum, June 2010.

Body length: 3.5 mm. Color: head light honey yellow, mesosoma and metasoma dark honey yellow; scape light honey yellow, flagellum brown; legs light honey yellow, apical tarsomeres brown; stigma yellow, remainder if veins light brown. Head: vertex and frons transversely striate, face and temple smooth; malar space about 1/3 eye height; temple slightly less that eye width, sloping behind eye, not bulging; ocell-ocular distance about 2.5 times diameter of lateral ocellus; 27 flagellomeres remain (incomplete). Mesosoma: propleuron smooth; pronotum with lateral groove scrobiculate and with distinct carina along lower edge, rugose below groove, granulate above groove; mesoscutal lobes granulate, notauli distinctly scrobiculate, meeting before scutellar groove in triangular rugose area; prescutellar furrow with 5 cross carinae; scutellum weakly granulate; mesopleuron smooth with weak striation dorsally, subalar groove strongly scrobiculate; episternal scrobe weak, precoxal suture (sternaulus) finely scrobiculate and about 23 length of mesopleuron; venter weakly granulate, median longitudinal groove narrow, scrobiculate; propodeum with basal median areas granulate and margined by distinct carinae, median basal carina distinct and about as long as first flagellomere, areola not distinctly margined, areola area rugose, lateral area entirely rugose. Wings: fore wing vein r about 2/3 length of vein 3RSa, vein 2RS entirely spectral except for short stub at junction of r and 3RSa, vein 1cu-a distinctly beyond vein 1M by about 1/2 its length; hind wing vein SC+R present and complete, vein M+CU shorter than vein 1M. Legs: hind coxa with distinct antero-ventral tubercle. Metasoma: first tergum with apical width about 2/3 length, longitudinally costate, raised median are distinct only at base and defined by 2 short lateral carinae; second tergum short, with width about 3 times length, longitudinally costate, anterior transverse groove distinct, scrobiculate; third tergum entirely longitudinally costate except smooth at apical edge, posterior transverse groove distinct; fourth and fifth terga costate on basal half, smooth on apical half; sixth and seventh terga smooth; ovipositor about half as long as metasoma.

Labels: first, “found in box 73, det C.v.Achterberg 1986”; second, red label “female Rogas (Heterospilus) quaestor Haliday, C. van Achterberg 1986, LECTOTYPE”; third, “Specimen figured, C.v.Achterberg 1986.” No locality labels.

Because the specimen did not have a locality label, a question remained as to how this specimen was picked as the holotype. Van Achterberg (per. comm.) stated - “It is based on circumstantial evidence; all the boxes of Haliday had a special arrangement. The *Heterospilus* was among other specimens from the same collection Haliday worked on. It was also the only *Heterospilus* in the collection and it is obviously not a European species. On the box was an indication of the origin, but this may be a later one...The locality St. Vincent is primarily based on the fit between specimen and description and the quote in the original description.” The locality indicated in Haliday’s description is “Insula Sti. Vincentii.” Thus, it seems reasonable that the specimen selected and designated as lectotype by van Achterberg is correct. I have studied several hundred specimens of *Heterospilus* from the Lesser Antilles Islands in the Caribbean but have not yet been able to find any specimens that fit the lectotype in order to select a homotype.

### Keys to and Descriptions of Costa Rican species of *Heterospilus*

The genus *Heterospilus* is one of the most speciose braconid genera in the Western Hemisphere. In the present study 280 species are described, most for the first time. The immediate challenge for such a large group is finding a character or characters that will divide the group into manageable sub-groups. In a preliminary study of the altitudinal diversity of Costa Rican *Heterospilus*, van der Ent (1999) divided the genus into three groups based on whether the antennal flagellum was entirely brown, brown with an apical white annulus or brown with a sub-apical white annulus. This has proven to be a good character but, unfortunately, at least half of the specimens studied had the flagellum broken or missing. So the character was unreliable for sorting the genus into three groups. However, a nearly perfectly reliable character was found, namely the sculpturing of the vertex, whether it is transversely costate or striate, granulate or smooth.

Presented below are keys and descriptions to the 280 species of Costa Rican *Heterospilus*. Keys and descriptions are presented separately for the three groups based on sculpturing of the vertex. Although this is a very reliable character, in a few rare instances where sculpturing may be weak, the species are taken through both keys.

The following keys to species are based on females only. As is often the case in braconid taxonomy, the sexes are more or less dimorphic. In the case of *Heterospilus*, the males are not dramatically dissimilar to the females except for the presence of a distinct stigma in the hind wing. Because the length of the ovipositor and hind wing venation are important characters to distinguish females, it is not possible to include both males and females in one key to species. Because of the number of species being dealt with from the Costa Rican fauna it was decided to include only females in the keys and descriptions. For the future it is planned to associate as many males with females as possible and present keys to the males.

The following table provides the starting page number for each group.

### Table for distinguishing species groups of Costa Rican *Heterospilus*

**Table:** 

1	Species with transversely striate or costate vertex	Page 26
2	Species with granulate vertex	Page 204
3	Species with smooth vertex	Page 330

### Key to species of Costa Rican *Heterospilus* with striate or costate vertex

**Table:** 

1	Mesopleural disc smooth, at least directly above precoxal sulcus	2
–	Mesopleural disc granulate, costate or strigate	50
2(1)	Ovipositor longer than metasoma	3
–	Ovipositor equal to or shorter than metasoma	23
3(2)	Apical width of first metasomal tergum equal to or greater than length	4
–	Apical width of first metasomal tergum less than length	11
4(3)	Face distinctly areolate	5
–	Face rugose or striate, at least below antennae, granulate or smooth	6
5(4)	Stigma of fore wing bicolored brown with basal third yellow; vertex rugose striate	*Heterospilus nigricoxus* Marsh, sp. n.
–	Stigma brown; vertex transversely costate	*Heterospilus cushmani* Marsh, sp. n.
6(4)	Lateral mesoscutal lobes transversely costate	*Heterospilus cressoni* Marsh, sp. n. (in part)
–	Lateral mesoscutal lobes granulate or smooth	7
7(6)	Anterior transverse groove on metasomal terga 2+3 sinuate	*Heterospilus rhabdotus* Marsh, sp. n. (in part)
–	Anterior transverse groove on metasomal terga 2+3 straight	8
8(7)	Stigma of fore wing yellow; mesoscutal lobes smooth	*Heterospilus fonsecai* Marsh, sp. n.
–	Stigma brown or bicolored; mesoscutal lobes granulate	9
9(8)	Stigma of fore wing entirely brown	*Heterospilus vierecki* Marsh, sp. n.
–	Stigma bicolored, brown with yellow apex	10
10(9)	Metasomal terga 3-4 nearly entirely longitudinally costate	*Heterospilus puntarensis* Marsh, sp. n.
–	Metasomal terga 3-4 smooth, costate only at base	*Heterospilus limonensis* Marsh, sp. n.
11(3)	Metasomal terga 4 and 5 granulate or costate-granulate, at least basally	12
–	Metasomal terga 4 and 5 smooth or weakly striate at extreme base	14
12(11)	Head and mesosoma (except lower portion of propleuron) honey yellow	*Heterospilus azofeifai* Marsh, sp. n.
–	Head and mesosoma dark brown or black	13
13(12)	Notauli meeting posteriorly in triangular rugose area before scutellum; metasomal tergum 2 dark brown medially; flagellum entirely brown	*Heterospilus laselvus* Marsh, sp. n.
–	Notauli meeting posteriorly in longitudinally costate are before scutellum; metasomal tergum 2 light brown or yellow medially; flagellum brown with apical 8-10 flagellomeres white	*Heterospilus kulai* Marsh, sp. n.
14(11)	Face sculptured, rugose, striate or granulate	15
–	Face more or less smooth	21
15(14)	Anterior transverse groove of metasomal tergum 2 sinuate	*Heterospilus noyesi* Marsh, sp. n.
–	Anterior transverse groove of metasomal tergum 2 straight	16
16(15)	Propodeum laterally rugose above hind coxa, smooth or weakly granulate anteriorly	*Heterospilus hansoni* Marsh, sp. n.
–	Propodeum entirely rugose laterally	17
17(16)	Prescutellar furrow with 3-5 cross carinae	18
–	Prescutellar furrow with 1 median cross carinae	19
18(17)	Scape yellow with weak lateral longitudinal brown stripe; basal median areas of propodeum smooth or weakly granulate	*Heterospilus cartagoensis* Marsh, sp. n.
–	Scape entirely yellow; basal median areas of propodeum at least partially rugose	*Heterospilus wilkinsoni* Marsh, sp. n.
19(17)	Mesoscutum lighter brown than remainder of mesosoma; basal median areas of propodeum smooth	*Heterospilus rohweri* Marsh, sp. n.
–	Mesosoma entirely black or dark brown; basal median areas of propodeum granulate	20
20(19)	Basal flagellomeres bicolored, each flagellomere brown with lighter brown or yellow at base and apex	*Heterospilus guanacastensis* Marsh, sp. n.
–	Flagellomeres uniformly brown	*Heterospilus foersteri* Marsh, sp. n.
21(14)	Mesoscutum without short transverse carinae along notauli; first metasomal tergum length distinctly longer than apical width	*Heterospilus alajuelus* Marsh, sp. n.
–	Mesoscutum with short transverse carinae along notauli; first metasomal tergum length equal or very slightly greater than apical width	22
22(21)	Mesoscutal lobes smooth	*Heterospilus menkei* Marsh, sp. n.
–	Mesoscutal lobes granulate	*Heterospilus milleri* Marsh, sp. n. (in part)
23(2)	Anterior transverse groove on metasomal terga 2-3 sinuate	24
–	Anterior transverse groove on metasomal terga 2-3 straight or only slightly bent at extreme edges	29
24(23)	Apical width of metasomal tergum 1 less than length	25
–	Apical width of metasomal tergum 1 nearly equal to or greater than length	26
25(24)	Mesoscutum black or dark brown	*Heterospilus milleri* Marsh, sp. n. (in part)
–	Mesoscutum honey yellow	*Heterospilus staryi* Marsh, sp. n.
26(24)	Body mostly yellow, often with lateral brown stripe along propodeum, upper mesopleuron and pronotum	*Heterospilus rhabdotus* Marsh, sp. n. (in part)
–	Body usually mostly dark brown or black	27
27(26)	Mesoscutal lobes smooth and polished	*Heterospilus brullei* Marsh, sp. n.
–	Mesoscutal lobes granulate	28
28(27)	Temple, in dorsal view, broad, not sloping inward behind eye, width greater than 1/2 eye width; hind femur yellow	*Heterospilus bruesi* Marsh, sp. n.
–	Temple, in dorsal view, narrow, sloping inward behind eye, width less than 1/2 eye width; hind femur brown on apical 2/3, yellow on basal 1/3	*Heterospilus stelfoxi* Marsh, sp. n.
29(23)	First metasomal tergum with apical width less than length	30
–	First metasomal tergum with apical width equal or greater than length	37
30(29)	Metasomal terga 2+3 smooth beyond anterior transverse groove, only posterior transverse groove occasionally sculptured	31
–	Metasomal terga 2+3 granulate and/or striate or costate beyond anterior transverse groove	35
31(30)	Fore wing vein r nearly on same line as vein 3RSa; area on propodeum just above hind coxa with distinct pointed tubercle	*Heterospilus costaricensis* Marsh, sp. n.
–	Fore wing vein r at distinct angle with vein 3RSa; area on propodeum just above hind coxa without distinct tubercle	32
32(31)	Head yellow; temple broad, not sloping behind eye; notauli bordered by dense long yellow setae	*Heterospilus muertensis* Marsh, sp. n.
–	Head at least partially black; temple narrow and sloping behind eye; notauli bordered by sparse short white setae	33
33(32)	Face striate	*Heterospilus reinhardi* Marsh, sp. n.
–	Face smooth	34
34(33)	Mesoscutal lobes smooth	*Heterospilus eberhardi* Marsh, sp. n.
–	Mesoscutal lobes granulate	*Heterospilus nixoni* Marsh, sp. n.
35(30)	Lateral mesoscutal lobes smooth	*Heterospilus ratzeburgi* Marsh, sp. n.
–	Lateral mesoscutal lobes granulate	36
36(35)	Precoxal sulcus extending to posterior margin of mesopleuron by carinate groove; apical width of metasomal tergum 2 about 3 times length	*Heterospilus agujas* Marsh, sp. n.
–	Precoxal sulcus shorter than mesopleuron, not extending to posterior margin of mesopleuron; apical width of metasomal tergum 2 about 4 times length	*Heterospilus zunigai* Marsh, sp. n.
37(29)	Mesoscutal lobes smooth and shining	38
–	Mesoscutal lobes granulate or striate	41
38(37)	Face punctate or rugulose; notauli meeting at scutellum in broad rectangular rugose area	*Heterospilus gauldi* Marsh, sp. n. (in part)
–	Face smooth or weakly striate; notauli meeting at scutellum in narrow triangular costate or rugose area	39
39(38)	Hind wing vein SC+R absent	*Heterospilus paloverde* Marsh, sp. n.
–	Hind wing vein SC+R present	40
40(39)	Stigma brown, basal 1/4 yellow; flagellomeres with white band on apical 1/4; sides of propodeum smooth anteriorly	*Heterospilus variabilis* Marsh, sp. n. (in part)
–	Stigma entirely brown; flagellomeres entirely brown; sides of propodeum entirely rugose	*Heterospilus janzeni* Marsh, sp. n.
41(37)	Lateral mesoscutal lobes rugose-granulate or transversely striate	42
–	Lateral mesoscutal lobes entirely granulate	43
42(41)	Face coarsely areolate; metasomal terga entirely black, tergum 3 smooth, posterior transverse groove absent	*Heterospilus areolatus* Marsh, sp. n.
–	Face weakly rugose or striate; metasomal tergum 2 brown with lateral orange stripes, tergum 3 orange medially	*Heterospilus cressoni* Marsh, sp. n. (in part)
43(41)	Prescutellar furrow with one distinct cross carina	44
–	Prescutellar furrow with 3-5 cross carinae	48
44(43)	Mesosoma with dense white hair along notauli, on scutellum, at subalar area, along posterior mesopleural groove and laterally on propodeum	*Heterospilus whitfieldi* Marsh, sp. n.
–	Mesosoma without dense hair in parts	45
45(44)	Body entirely honey yellow, propodeum occasionally light brown, stigma yellow	46
–	Body brown or black, legs yellow, stigma brown	47
46(45)	Face rugose; temple broad, not sloping behind eye; ocell-ocular distance less than twice diameter of lateral ocellus	*Heterospilus sinuatus* Marsh, sp. n.
–	Face smooth; temple narrow, sloping behind eye; ocell-ocular distance twice or more diameter of lateral ocellus	*Heterospilus fournieri* Marsh, sp. n.
47(45)	Basal median carinae of propodeum as long as half the dorsal length of propodeum	*Heterospilus macrocarinus* Marsh, sp. n.
–	Basal median carina of propodeum absent or very short	*Heterospilus cameroni* Marsh, sp. n.
48(43)	Face strongly rugose	*Heterospilus heredius* Marsh, sp. n.
–	Face smooth or weakly striate	49
49(48)	Notauli meeting before scutellum in wide rectangular rugose-costate area; metasomal tergum 2 with lateral converging yellow lines, brown medially	*Heterospilus neesi* Marsh, sp. n.
–	Notauli meeting before scutellum in triangular narrow rugose area; metasomal tergum 2 uniformly light brown or black	*Heterospilus emelius* Marsh, sp. n.
50(1)	Mesopleuron costate or strigate	*Heterospilus alajuelus* Marsh, sp. n. (in part)
–	Mesopleuron granulate	51
51(50)	Anterior transverse groove of metasomal terga 2+3 distinctly curved or sinuate, median length of tergum greater than lateral length	52
–	Anterior transverse groove of metasomal terga 2+3 straight, median length of tergum nearly equal to lateral length	72
52(51)	Apical width of first metasomal tergum less than length	53
–	Apical width of first metasomal tergum equal or greater than length	67
53(52)	Third and following metasomal terga granulate, at least at base	54
–	Third and following terga smooth, at least at apex	59
54(53)	Second metasomal tergum broad, median length more than half median length of third tergum, or nearly as long as third tergum	55
–	Second metasomal tergum narrow, median length less than half median length of third tergum	57
55(54)	Body green, sometimes marked with brown	*Heterospilus shawi* Marsh, sp. n. (in part)
–	Body dark brown or black or bicolored brown and yellow	56
56(54)	Propodeum entirely dark brown or black	*Heterospilus granulatus* Marsh, sp. n.
–	Propodeum with basal median areas and laterally yellow, areola brown	*Heterospilus vittatus* Marsh, sp. n.
57(54)	Base of hind tibia brown, remainder yellow	*Heterospilus nigragonatus* Marsh, sp. n.
–	Hind tibia entirely yellow	58
58(57)	Prescutellar furrow with one cross carina	*Heterospilus romani* Marsh, sp. n.
–	Prescutellar furrow with 3 cross carinae	*Heterospilus bribri* Marsh, sp. n.
59(53)	Length of first metasomal tergum about twice apical width	60
–	Length of first metasomal tergum about 1.5 times apical width	61
60(59)	Lateral mesoscutal lobes transversely costate	*Heterospilus gahani* Marsh, sp. n.
–	Lateral mesoscutal lobes granulate	*Heterospilus petralbus* Marsh, sp. n.
61(59)	Prescutellar furrow with one cross carina	*Heterospilus corcovado* Marsh, sp. n.
–	Prescutellar furrow with 3-5 cross carinae	62
62(61)	Fore wing vein r nearly as long as vein 3RSa, at least more than 1/2 length	63
–	Fore wing vein r at most 1/3 length of vein 3RSa	64
63(62)	Mesoscutum shining, weakly granulate; body dark brown or black	*Heterospilus lapierrei* Marsh, sp. n.
–	Mesoscutum granulate and dull; body honey yellow marked with brown	*Heterospilus flavidus* Marsh, sp. n.
64(62)	Face smooth or weakly granulate, shining	65
–	Face rugose or rugose-areolate, dull	66
65(64)	Hind tibia brown at extreme apex; flagellum entirely brown; notauli meeting at scutellum in broad rugose area	*Heterospilus wesmaeli* Marsh, sp. n.
–	Hind tibia entirely yellow; flagellum brown with apical 3-8 flagellomeres white; notauli meeting at scutellum in narrow triangular costate area	*Heterospilus monteverde* Marsh, sp. n.
66(64)	Scutellum granulate	*Heterospilus boharti* Marsh, sp. n.
–	Scutellum smooth and shining	*Heterospilus sanvitoensis* Marsh, sp. n.
67(52)	Face smooth or weakly granulate	68
–	Face rugose or striate	69
68(67)	Metasomal terga 4-6 entirely smooth	*Heterospilus leioenopus* Marsh, sp. n.
–	Metasomal terga 4-6 granulate at base	*Heterospilus nephus* Marsh, sp. n.
69(67)	Mesosoma unicolored dark brown or black	70
–	Mesoscutum lighter yellow than rest of mesosoma, or mesosoma entirely yellow or light brown	71
70(69)	Ovipositor less than 1/2 length of metasoma; flagellum entirely brown	*Heterospilus shenefelti* Marsh, sp. n.
–	Ovipositor greater than 1/2 length of metasoma; flagellum brown with apical 3-5 flagellomeres white	*Heterospilus angustus* Marsh, sp. n.
71(69)	Hind wing vein M+CU less than 1/2 length of vein 1M	*Heterospilus rhabdotus* Marsh, sp. n. (in part)
–	Hind wing vein M+CU more than 1/2 length of vein 1M	*Heterospilus similis* Marsh, sp. n.
72(51)	Face smooth and shining, occasionally weakly striate just below antennal sockets	73
–	Face sculptured striate, granulate or rugose, sometimes weakly so	90
73(72)	Prescutellar furrow with 1 cross carina	74
–	Prescutellar furrow with 3-5 cross carinae	78
74(73)	Hind wing vein SC+R absent	75
–	Hind wing vein SC+R present	76
75(74)	Ovipositor less than 1/2 length of metasoma	*Heterospilus garifuna* Marsh, sp. n.
–	Ovipositor slightly longer than metasoma	*Heterospilus itza* Marsh, sp. n.
76(74)	Metasomal tergum 2 smooth; length of tergum 1 nearly twice apical width	*Heterospilus levis* Marsh, sp. n.
–	Metasomal tergum 2 costate; length of tergum 1 nearly equal apical width	77
77(76)	Ovipositor equal to length of metasomal 1-2 combined; flagellum with apical flagellomeres white	*Heterospilus tolupan* Marsh, sp. n.
–	Ovipositor longer than metasomal terga 1-2 combined; flagellum entirely brown	*Heterospilus kuna* Marsh, sp. n.
78(73)	Hind wing vein SC+R absent	79
–	Hind wing vein SC+R present	80
79(78)	Ovipositor longer than metasoma	*Heterospilus jakaltek* Marsh, sp. n.
–	Ovipositor shorter than metasoma	*Heterospilus longius* Marsh, sp. n. (in part)
80(78)	Metasomal tergum 2 short, greatest width 4 times median length	81
–	Metasomal tergum 2 longer, greatest width 3 times or less length	84
81(80)	Median basal carina of propodeum distinct	82
–	Median basal carina of propodeum absent, areola meeting anterior edge of propodeum	83
82(81)	Body honey yellow, legs yellow, flagellomeres brown	*Heterospilus mellosus* Marsh, sp. n.
–	Body dark brown, legs yellow, apical 4-5 flagellomeres white	*Heterospilus szepligetii* Marsh, sp. n.
83(81)	Tegula yellow; metasomal terga beyond second brown or light brown	*Heterospilus masoni* Marsh, sp. n.
–	Tegula dark brown; metasomal terga entirely brown	*Heterospilus golfodulcensis* Marsh, sp. n.
84(80)	Propodeum with distinct tubercle laterally on each side of base of metasomal tergum 1, above hind coxa	85
–	Propodeum without such tubercle	86
85(84)	Lateral mesoscutal lobes entirely granulate; temple narrow, sloping behind eye; apical 8–10 flagellomeres white; stigma brown	*Heterospilus gouleti* Marsh, sp. n.
–	Lateral mesoscutal lobes partially smooth; temple broader, not sloping behind eyes; flagellum with white annulus but apical 5-6 brown; stigma yellow	*Heterospilus kiefferi* Marsh, sp. n.
86(84)	Metasomal tergum 1 with apical width 1/2 or less than length	87
–	Metasomal tergum 1 with apical width about equal to length, rarely slightly greater but at least more than 1/2 length	88
87(86)	Fore wing vein r less than 1/2 length of vein 3RSa	*Heterospilus halidayi* Marsh, sp. n.
–	Fore wing vein r greater than 1/2 length of vein 3RSa, often nearly equal	*Heterospilus cachiensis* Marsh, sp. n.
88(86)	Hind coxa granulate dorsally; apical lateral corners of propodeum produced into small tubercle	*Heterospilus fischeri* Marsh, sp. n. (in part)
–	Hind coxa costate dorsally; apical lateral corner of propodeum not produced into a tubercle	89
89(88)	Metasomal terga 4 distinctly and 5 weakly longitudinally striate at base	*Heterospilus nigrescens* Ashmead
–	Metasomal terga 4-5 entirely smooth	*Heterospilus escazuensis* Marsh, sp. n.
90(72)	Body green, sometimes marked with brown	*Heterospilus shawi* Marsh, sp. n. (in part)
–	Body dark brown or black or bicolored brown and yellow	91
91(72)	Hind wing vein SC+R absent	92
–	Hind wing vein SC+R present	99
92(91)	Apical-lateral corners of propodeum produced into blunt tubercle	93
–	Apical-lateral corners of propodeum not produced into tubercles	94
93(92)	Ovipositor about as long as metasomal terga 1-3; fore wing vein r longer than vein 3RSa	*Heterospilus longius* Marsh, sp. n. (in part)
–	Ovipositor only as long as metasomal tergum 1	*Heterospilus enderleini* Marsh, sp. n.
94(92)	Ocell-ocular distance twice or more diameter of lateral ocellus	95
–	Ocell-ocular distance at most 1.5 times diameter of lateral ocellus	96
95(94)	Prescutellar furrow with 3 cross carinae; flagellum entirely brown apically, without white flagellomeres	*Heterospilus grisselli* Marsh, sp. n.
–	Prescutellar furrow with 1 median cross carina; flagellum brown with apical 3-5 flagellomeres white	*Heterospilus quickei* Marsh, sp. n.
96(94)	Scutellum and basal median areas of propodeum rugose	*Heterospilus rugosus* Marsh, sp. n.
–	Scutellum and basal median areas of propodeum granulate	97
97(96)	Fore wing vein r longer than vein 3RSa	*Heterospilus tobiasi* Marsh, sp. n.
–	Fore wing vein r equal to or shorter than vein 3RSa	98
98(97)	Ovipositor nearly as long as metasoma	*Heterospilus ramirezi* Marsh, sp. n.
–	Ovipositor about as long as metasomal terga 2+3	*Heterospilus muesebecki* Marsh, sp. n.
99(91)	Metasomal tergum 1 narrow, apical width equal to or less than 1/2 length and only slightly greater than basal width	*Heterospilus turrialbaensis* Marsh, sp. n.
–	Metasomal tergum 1 shorter and broader, apical width greater than 1/2 length, often equal to length and nearly twice basal width	100
100(99)	Metasomal terga 3 and 4 granulate at apex	101
–	Metasomal terga 3 and 4 usually smooth at apex, occasionally costate to apex	104
101(100)	Mesoscutal lobes rugose-granulate	*Heterospilus corrugatus* Marsh, sp. n.
–	Mesoscutal lobes granulate	102
102(101)	Prescutellar furrow with one median cross carina	*Heterospilus haplocarinus* Marsh, sp. n.
–	Prescutellar furrow with 3 cross carinae	103
103(102)	Mesoscutum evenly brown; flagellum brown; basal median carina of propodeum absent, areola meeting basal margin of propodeum	*Heterospilus miskito* Marsh, sp. n.
–	Mesoscutal lobes dark brown, yellow along notauli; apical 4-6 flagellomeres white; basal median carina present, short, areola not meeting basal margin of propodeum	*Heterospilus rinconensis* Marsh, sp. n.
104(100)	Vertex and frons strongly circularly costate around ocelli	105
–	Vertex and usually frons transversely striate or costate, often weakly	106
105(104)	Vertex and frons yellow; scutellum granulate	*Heterospilus orbitus* Marsh, sp. n.
–	Vertex and frons dark brown; scutellum smooth	*Heterospilus whartoni* Marsh, sp. n.
106(104)	Mesoscutal lobes granulate, rugose at least along notauli, occasionally lateral lobes nearly entirely rugose	107
–	Mesoscutal lobes entirely granulate	108
107(106)	Apical width of metasomal tergum 1 slightly greater than length; median mesoscutal lobe with median longitudinal scrobiculate groove anteriorly	*Heterospilus santarosensis* Marsh, sp. n.
–	Apical width of metasomal tergum 1 slightly to distinctly less than length; median mesoscutal lobe with median longitudinal raised ridge anteriorly	*Heterospilus maritzaensis* Marsh, sp. n.
108(106)	Ovipositor longer than or equal to metasoma	109
–	Ovipositor shorter than metasoma	113
109(108)	Temple in dorsal view broad, not sloping behind eye, width equal to or greater than 1/2 eye width	110
–	Temple in dorsal view narrow, sloping behind eye, width less than 1/2 eye width	111
110(109)	Ovipositor longer than metasoma; length of metasomal tergum 1 greater than apical width	*Heterospilus chaoi* Marsh, sp. n.
–	Ovipositor as long as metasoma; length of metasomal tergum 1 equal to apical width	*Heterospilus jenniae* Marsh, sp. n. (in part)
111(109)	Coxae and trochanters white, remainder of legs yellow	*Heterospilus albocoxalis* Marsh, sp. n.
–	Legs entirely yellow	112
112(111)	Eye large, malar space about 1/6 eye height, ocell-ocular distance about equal to diameter of lateral ocellus; flagellomeres brown	*Heterospilus magnus* Marsh, sp. n. (in part)
–	Eye smaller, malar space about 1/4 eye height, ocell-ocular distance greater than diameter of lateral ocellus; apical flagellomeres white	*Heterospilus mixtec* Marsh, sp. n.
113(108)	Lateral ocelli large, ocell-ocular distance 1.5 times or less diameter of lateral ocellus	114
–	Lateral ocelli smaller, ocell-ocular distance at least twice diameter of lateral ocellus	115
114(113)	Body entirely yellow or honey yellow, stigma yellow	*Heterospilus xanthus* Marsh, sp. n.
–	Body dark brown or black, legs yellow, stigma brown	*Heterospilus huddlestoni* Marsh, sp. n.
115(113)	Scape yellow or light brown, with longitudinal brown stripe on lateral (outside) edge	116
–	Scape yellow, without lateral longitudinal brown stripe, or entirely brown	118
116(115)	Prescutellar furrow with one distinct median cross carina	*Heterospilus cacaoensis* Marsh, sp. n.
–	Prescutellar furrow with 3-5 cross carinae	117
117(116)	Head mostly yellow, mesosoma and metasoma brown	*Heterospilus yaqui* Marsh, sp. n.
–	Body unicolored brown or dark brown	*Heterospilus townesi* Marsh, sp. n.
118(115)	Transverse grooves on metasomal terga 2-3 absent, anterior groove rarely weakly indicated, posterior groove always absent	119
–	Both transverse grooves on metasomal terga 2-3 present, sometimes somewhat weakly so	120
119(118)	Apical flagellomeres white; coxae and trochanters often white	*Heterospilus curtisi* Marsh, sp. n.
–	Flagellomeres entirely brown; coxae and trochanters yellow	*Heterospilus fahringeri* Marsh, sp. n.
120(118)	Stigma yellow; ovipositor as long as metasomal tergum 1	*Heterospilus hedqvisti* Marsh, sp. n.
–	Stigma brown; ovipositor longer than metasomal tergum 1	121
121(120)	Body dark brown; flagellum brown, apical 5-8 flagellomeres white	*Heterospilus brethesi* Marsh, sp. n.
–	Body brown with face, mesoscutum and metasomal terga honey yellow or lighter brown; flagellum brown	*Heterospilus pappi* Marsh, sp. n.

### Heterospilus agujas

Marsh sp. n.

http://zoobank.org/63F64057-DA06-417F-BB5E-8A87A1263C3B

http://species-id.net/wiki/Heterospilus_agujas

Figure 11

#### Female.

Body size: 3.5 mm. Color: entire body dark brown, metasomal terga 4-7 lighter brown; scape yellow without lateral longitudinal brown stripe, flagellum brown with apical 5–8 flagellomeres white; legs with coxae and trochanters yellow, femora, tibiae and tarsi light brown to brown; wing veins including stigma brown. Head: vertex transversely striate; frons transversely striate; face smooth medially, striate below antennae and on malar space; temple in dorsal view narrow, sloping behind eye, width less than 1/2 eye width; malar space greater than 1/4 eye height; ocell-ocular distance twice diameter of lateral ocellus; 24 flagellomeres. Mesosoma: mesoscutal lobes granulate; notauli scrobiculate, meeting at scutellum in triangular costate-rugose area; scutellum smooth; prescutellar furrow with 3 cross carinae; mesopleuron smooth; precoxal sulcus weakly scrobiculate, extending to posterior margin of mesopleuron by carinate groove; venter smooth; propodeum with basal median areas distinctly margined and granulate, basal median carina present, short, areola not distinctly margined, areolar area rugose, lateral areas rugose posteriorly, smooth anteriorly. Wings: fore wing vein r shorter than vein 3RSa, vein 1cu-a beyond vein 1M; hind wing vein SC+R present, vein M+CU shorter than vein 1M. Metasoma: first tergum longitudinally costate, length greater than apical width; second tergum longitudinally costate, width about 3 times length; anterior transverse groove present, straight; posterior transverse groove present; third tergum granulate basally, smooth apically; terga 4-7 smooth; ovipositor equal to length of metasoma.

#### Holotype female.

Top label (white, printed) - Costa Rica: Puntarenas, ACO [;] Golfito, P.N. Corcovado [sic], 745m [;] Est. Agujas, Cerro Rincon [;] 15.v–15.vi.1999. J. Azofeifa [;] L.S. 276900-521500 #52744 [;] Malaise trap; second label (red, partially printed and hand written) - HOLOTYPE [;] Heterospilus [;] agujas [;] P. Marsh. Deposited in ESUW.

#### Paratypes.

Known only from the holotype.

#### Comments.

This species is distinguished by the precoxal sulcus being as long as the mesopleuron, extending to the posterior margin of the mesopleuron by distinct carinate groove, by the ovipositor as long as the metasoma and by the length of the first metasomal tergum nearly twice the apical width.

#### Etymology.

Named for the collecting locality of Estacion Agujas.

**Figure 11.**
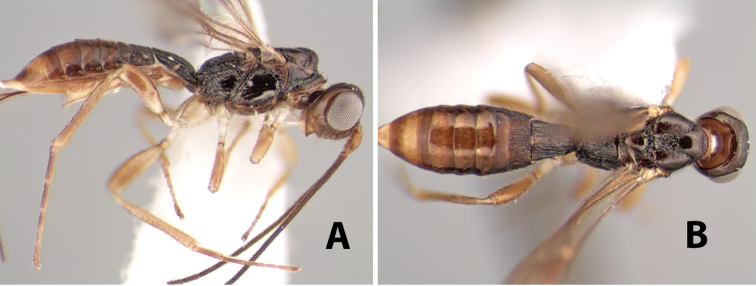
*Heterospilus agujas* Marsh, sp. n., holotype.

### Heterospilus alajuelus

Marsh sp. n.

http://zoobank.org/1F62115C-1C20-4EA6-A779-2DC93C1BF4DC

http://species-id.net/wiki/Heterospilus_alajuelus

Figure 12

#### Female.

Body size: 3.0–4.0 mm. Color: head yellow to light brown; scape yellow without lateral brown stripe, flagellum brown with apical 3–5 flagellomeres white; mesosoma dark brown with mesoscutum and venter usually yellow or honey yellow; legs yellow; wing veins including stigma brown; metasomal tergum 1 dark brown, tergum 2 brown laterally, yellow medially and apically; tergum 3 brown, yellow basally, terga 4–6 yellow, brown at basal edge. Head: vertex transversely costate; frons transversely costate; face smooth, occasionally weakly striate below eyes; temple in dorsal view somewhat broad but sloping behind eye, width equal to 1/2 eye width; malar space greater than 1/4 eye height; ocell-ocular distance about 2.5 times diameter of lateral ocellus; 22–27 flagellomeres. Mesosoma: mesoscutal lobes granulate; notauli scrobiculate, meeting posteriorly in triangular rugose area; scutellum smooth; prescutellar furrow with 3–5 cross carinae; mesopleuron smooth, occasionally costate dorsally or entirely; precoxal sulcus scrobiculate, shorter than mesopleuron; venter smooth; propodeum with basal median areas margined, smooth, basal median carina present, areola margined, areolar area broadly rugose or areolate, lateral areas entirely rugose. Wings: fore wing vein r shorter than vein 3RSa, vein 1cu-a beyond vein 1M; hind wing vein SC+R present, vein M+CU shorter than vein 1M. Metasoma: first tergum longitudinally costate, length greater than apical width; second tergum longitudinally costate; anterior transverse groove weak, sometimes absent, straight; posterior transverse groove weak or sometimes absent; third tergum costate basally, smooth apically; terga 4–7 smooth; ovipositor longer than metasoma.

#### Holotype female.

Top label (white, printed) - Costa Rica: Alajuela [;] 5km W San Ramon [;] 1200m, iv.1997 [;] O.Castro & P.Hanson; second label (red, partially printed and hand written) - HOLOTYPE [;] Heterospilus [;] alajuelus [;] P. Marsh. Deposited in ESUW.

#### Paratypes.

1 ♀, same data as holotype (ESUW). 1 ♀, Costa Rica: Alajuela [;] R. B. A. Brenes [;] San Ramon, 900m [;] ii-iii.2000, P. Hanson (ESUW). 1 ♀, Costa Rica: Guanacaste Prov. [;] Guanacaste Conservation Area [;] below Cacao, 400-600m el. [;] 3 March 1990, J.S. Noyes (ESUW). 1 ♀, Costa Rica: Limon [;] 30km N Carari, 100m [;] Sector Cocori, Malaise [;] iii.1995, E. Rojas #4524 [;] L.N. 286000-567500 (ESUW). 1 ♀, Costa Rica: Puntarenas [;] San Vito, Estac. Biol. [;] Las Alturas, 1500m [;] v.1992, Forest border, [;] Malaise, Paul Hanson (ESUW). 1 ♀, COSTA RICA, Puntarenas [;] Monteverde, 1400m [;] 30-IV-89 Col. Hanson (ESUW). 2 ♀♀, Est. Cacao,1000-1400m, [;] Lado SO Vol. Cacao, [;] P.N.G., Prov. Guan. [;] COSTA RICA, C. [;] Chaves, Abr 1991. [;] L-N-323300,375700 (INBC).

#### Comments.

The smooth face, smooth mesopleuron and white tip of the flagellum are distinctive for this species.

#### Etymology.

The specific name is from the type locality located in Alajuela Province.

**Figure 12.**
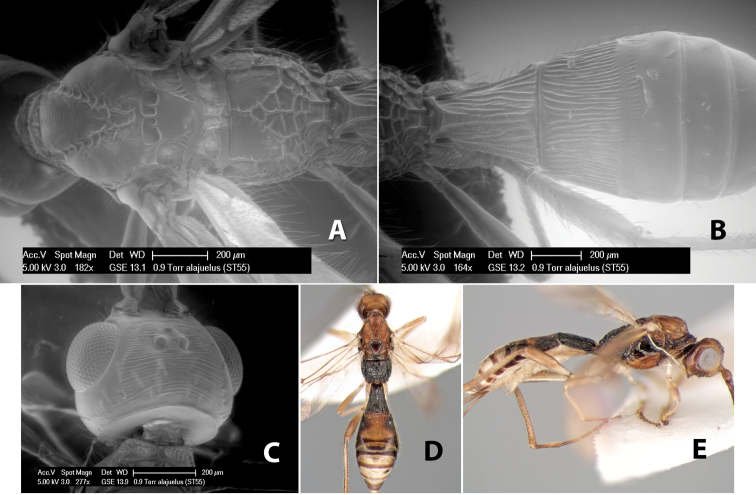
*Heterospilus alajuelus* Marsh, sp. n., holotype.

### Heterospilus albocoxalis

Marsh sp. n.

http://zoobank.org/00DE22F9-9A82-4236-90AB-ADFBCDDA2D88

http://species-id.net/wiki/Heterospilus_albocoxalis

Figure 13

#### Female.

Body size: 3.0 mm. Color: head brown; scape yellow without lateral brown stripe, flagellum brown with apical 3–5 flagellomeres white; mesosoma brown, propleuron honey yellow; metasomal tergum 1 dark brown, tergum 2 yellow, terga 3–7 brown to dark brown; wing veins including stigma brown; coxae and trochanters white, remainder of legs yellow. Head: vertex transversely striate; frons transversely striate; face striate; temple in dorsal view narrow, width less than 1/2 eye width; malar space greater than 1/4 eye height; ocell-ocular distance greater than 2.5 times diameter of lateral ocellus; 23 flagellomeres. Mesosoma: mesoscutal lobes granulate; notauli scrobiculate, meeting at scutellum in triangular rugose area; scutellum weakly granulate; prescutellar furrow with 1 cross carina; mesopleuron granulate; precoxal sulcus scrobiculate, shorter than mesopleuron; venter granulate; propodeum with basal median areas margined, granulate, basal median carina present, areola distinctly margined, areolar area rugose, lateral areas rugose posteriorly, granulate anteriorly. Wings: fore wing vein r shorter than vein 3RSa, vein 1cu-a beyond vein 1M; hind wing vein SC+R present, vein M+CU shorter than vein 1M. Metasoma: first tergum longitudinally costate, apical width less than length; second tergum granulate-rugulose; anterior transverse groove weak or absent; posterior transverse groove weak or absent; third tergum granulate at base, smooth at apex; terga 4-7 smooth; ovipositor longer than metasoma.

#### Holotype female.

Top label (white, printed) - Costa Rica: Prov. Puntarenas [;] ACO, Golfito, PN Corcovado [;] Est. Agujas, Cerro Rincon, 745m [;] 17.iv–16.v.1999, J. Azofeifa [;] L.S. 276900-521500 #52781; second label (red, partially printed and hand written) - HOLOTYPE [;] Heterospilus [;] albocoxalis [;] P. Marsh. Deposited in ESUW.

#### Paratypes.

Known only from the holotype.

#### Comments.

The white coxae and trochanters are distinctive for this species.

#### Etymology.

The specific name is from the Latin *albus* meaning white in reference to the white coxae.

**Figure 13.**
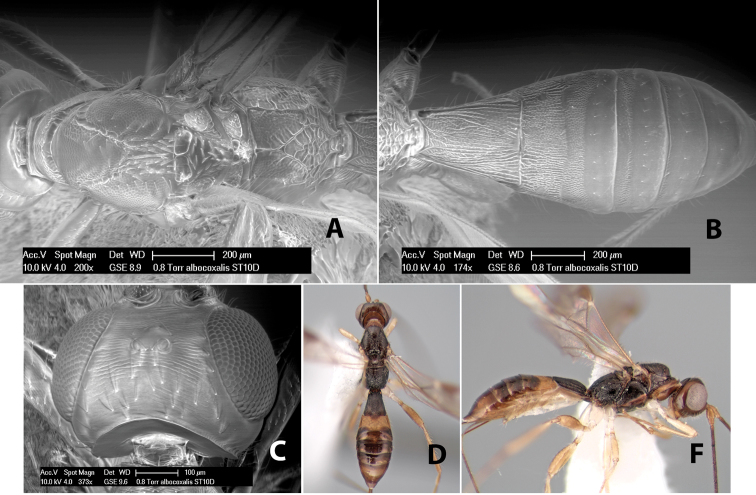
*Heterospilus albocoxalis* Marsh, sp. n., holotype.

### Heterospilus angustus

Marsh sp. n.

http://zoobank.org/F02AC25B-CAF5-4DCF-A1D2-A8D61599FEF9

http://species-id.net/wiki/Heterospilus_angustus

Figure 14

#### Female.

Body size: 2.5 mm. Color: head honey yellow; scape yellow without lateral longitudinal brown stripe, flagellum brown with apical 3–5 flagellomeres white; mesosoma brown, somewhat lighter along notauli; metasomal terga 1–4 brown, tergum 2 slightly lighter, terga 5–7 yellow; wing veins including stigma brown; legs yellow. Head: vertex costate; frons costate; face rugose; temple in dorsal view narrow, width less than 1/3 eye width; malar space equal to 1/4 eye height; ocell-ocular slightly less than 2.5 times diameter of lateral ocellus; 19 flagellomeres. Mesosoma: mesoscutal lobes granulate; notauli scrobiculate, meeting at scutellum in triangular rugose area; scutellum granulate; prescutellar furrow with 3–5 cross carinae; mesopleuron granulate; precoxal sulcus scrobiculate, shorter than mesopleuron; venter smooth; propodeum with basal median areas margined, granulate, basal median carina absent, areola not margined, areolar area rugose, lateral areas entirely rugose. Wings: fore wing vein r shorter than vein 3RSa, vein 1cu-a beyond vein 1M; hind wing vein SC+R present, vein M+CU shorter than vein 1M. Metasoma: first tergum longitudinally costate, apical width equal to length; second tergum longitudinally costate, width about 4 times length; anterior transverse groove present, sinuate; posterior transverse groove present; third tergum costate basally, smooth apically; terga 4–7 smooth; ovipositor about 3/4 length of metasoma.

#### Holotype female.

Top label (white, printed) - Costa Rica: Cartago [;] Braulio Carillo N.P. [;] 600m, 25.iii.1990 [;] J. S. Noyes, coll.; second label (red, partially printed and hand written) - HOLOTYPE [;] Heterospilus [;] angustus [;] P. Marsh. Deposited in ESUW.

#### Paratypes.

Known only from the holotype.

#### Comments.

The very narrow temple is distinctive for this species.

#### Etymology.

The specific name is from the Latin *angustus* meaning narrow in reference to the extremely narrow temple.

**Figure 14.**
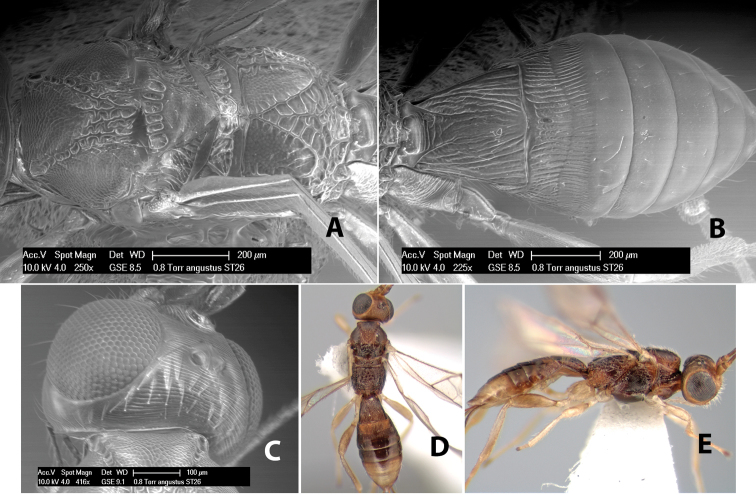
*Heterospilus angustus* Marsh, sp. n., holotype.

### Heterospilus areolatus

Marsh sp. n.

http://zoobank.org/F20B473F-2212-47AD-8277-9C6FC279D104

http://species-id.net/wiki/Heterospilus_areolatus

Figures 15 , 16

#### Female.

Body size: 3.5 mm. Color: body dark brown or black; scape yellow without lateral longitudinal brown stripe, flagellum brown; legs yellow; wing veins including stigma brown. Head: vertex transversely costate; frons transversely costate; face coarsely areolate; temple in dorsal view narrow, sloping behind eye, width equal to eye width; malar space greater than eye 1/4 eye height; ocell-ocular distance slightly greater than twice diameter of lateral ocellus; 24 flagellomeres. Mesosoma: mesoscutal lobes coarsely rugose-granulate; notauli scrobiculate, meeting at scutellum in wide rugose area; scutellum smooth; prescutellar furrow with 3 cross carinae; mesopleuron smooth; precoxal sulcus smooth, shorter than mesopleuron; venter smooth; propodeum with basal median areas not margined, rugose, basal median carina absent, areola not margined, areolar area areolate-rugose, lateral areas entirely rugose. Wings: fore wing vein r nearly as long as vein 3RSa, vein 1cu-a beyond vein 1M; hind wing vein SC+R present, vein M+CU distinctly longer than vein 1M. Metasoma: first tergum apical width slightly greater than length, longitudinally costate, raised median triangular area distinctly margined and with basal cross carinae; second tergum longitudinally costate; anterior transverse groove absent; posterior transverse groove absent; third tergum entirely smooth; terga 4–7 smooth; ovipositor equal in length to metasomal terga 1–2.

#### Holotype female.

Top label (white, printed) - Costa Rica: Puntarenas, ACO [;] Golfito, Est. Agujas, 375m [;] Res. Ftal. Golfo Dulce [;] 16.iv–16.v.1999, J. Azofeifa [;] L.S. 276551-526423 #52775 [;] Malaise trap; second label (red, partially printed and hand written) - HOLOTYPE [;] Heterospilus [;] areolatus [;] P. Marsh. Deposited in ESUW.

#### Paratypes.

Known only from the holotype.

#### Comments.

The species is distinguished by its coarsely areolate face and long hind wing vein M+CU.

#### Etymology.

The specific name is in reference to the very distinctly areolate face.

**Figure 15.**
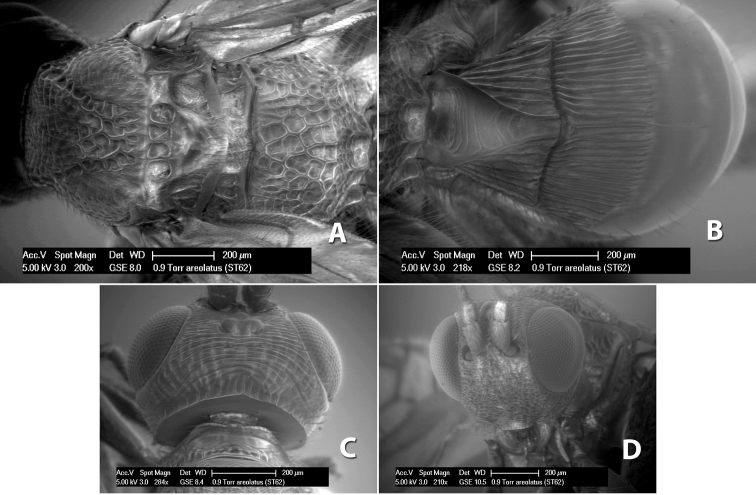
*Heterospilus areolatus* Marsh, sp. n., holotype.

**Figure 16.**
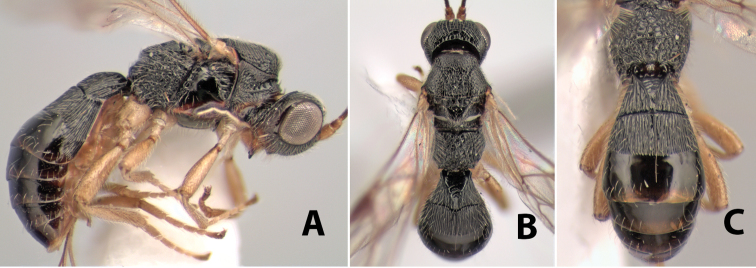
*Heterospilus areolatus* Marsh, sp. n., holotype.

### Heterospilus azofeifai

Marsh sp. n.

http://zoobank.org/4CF42C17-2D1A-4A56-B75D-458B61911DA0

http://species-id.net/wiki/Heterospilus_azofeifai

Figure 17

#### Female.

Body size: 4.5 mm. Color: head honey yellow; scape light brown without distinct lateral longitudinal brown stripe, flagellum brown (broken); mesosoma honey yellow, pronotum, mesopleuron dorsally and propodeum laterally brown; metasoma honey yellow, terga 1-2 brown laterally, terga 6-7 brown; legs yellow, base of hind tibia brown; wing veins including stigma brown. Head: vertex transversely striate; frons transversely striate; face weakly granulate; temple in dorsal view narrow and sloping behind eye, less than 1/2 eye width; malar space greater than 1/4 eye height; ocell-ocular distance about 1.5 times diameter of lateral ocellus; ? flagellomeres (broken). Mesosoma: mesoscutal lobes granulate; notauli scrobiculate, meeting at scutellum in triangular rugose area; scutellum weakly granulate; prescutellar furrow with 1 distinct median cross carinae and 2 lateral weak carinae; mesopleuron smooth; precoxal sulcus weakly scrobiculate, shorter than mesopleuron; venter smooth; propodeum with basal median areas distinctly margined, smooth, basal median carina present, areola not distinctly margined, areolar area rugose, lateral areas rugose posteriorly, smooth or granulate anteriorly. Wings: fore wing vein r shorter than vein 3RSa, vein 1cu-a beyond vein 1M; hind wing vein SC+R present, vein M+CU shorter than vein 1M. Metasoma: first tergum costate, longer than apical width; second tergum costate, narrow with width about 4 times length; anterior transverse groove present, sinuate; posterior transverse groove absent; third tergum costate at base, granulate apically; terga 4–7 granulate; ovipositor longer than metasoma.

#### Holotype female.

Top label (white, printed) - Costa Rica: Puntarenas [;] ACO, Golfito, RF Golfo Dulce [;] Est. Agujas, 250-300m [;] 3–24.vi.1999. J. Azofeifa [;] L.S. 276750-526550 #52840 [;] Red de Golpe; second label (red, partially printed and hand written) - HOLOTYPE [;] Heterospilus [;] azofeifai [;] P. Marsh. Deposited in ESUW.

#### Paratypes.

Known only from the holotype.

#### Comments.

This species is distinguished by the long ovipositor, yellow body and granulate apical metasomal terga.

#### Etymology.

Named for the collector of the holotype, J. Azofeifa.

**Figure 17.**
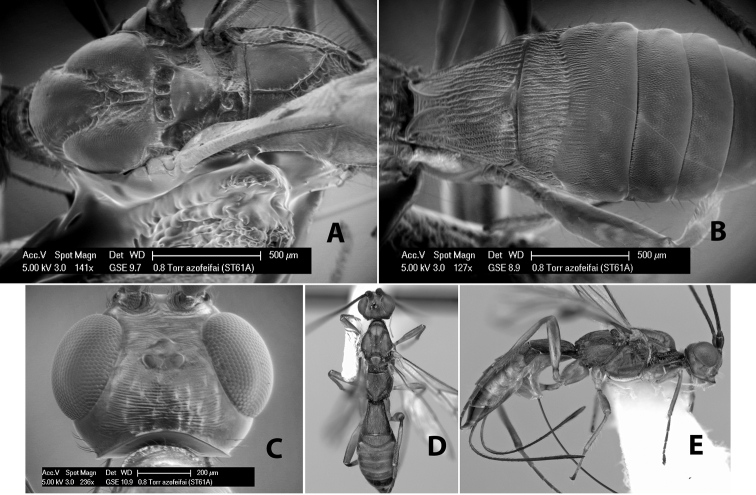
*Heterospilus azofeifai* Marsh, sp. n., holotype.

### Heterospilus boharti

Marsh sp. n.

http://zoobank.org/53A78F64-B3FC-4098-B603-34391ED4E14A

http://species-id.net/wiki/Heterospilus_boharti

Figures 18 , 19

#### Female.

Body size: 3.5–4.0 mm. Color: head dark brown, face sometimes lighter brown than vertex; scape yellow without lateral longitudinal brown stripe, flagellum brown with apical 5–8 flagellomeres white; mesosoma dark brown; metasomal terga dark brown, apical terga often lighter brown; wing veins including stigma brown; legs yellow, hind femur yellow on basal half, brown on apical half. Head: vertex transversely costate; frons transversely costate; face rugose or rugose-areolate; temple in dorsal view narrow, width less than 1/2 eye width; malar space greater than 1/4 eye height; ocell-ocular distance about 2.5 times diameter of lateral ocellus; 25–27 flagellomeres. Mesosoma: mesoscutal lobes granulate; notauli scrobiculate, meeting at scutellum in triangular rugose area; scutellum granulate; prescutellar furrow with 3 cross carinae; mesopleuron granulate; precoxal sulcus scrobiculate, extending to posterior edge of mesopleuron by costate line; venter granulate; propodeum with basal median areas margined, granulate-rugose, basal median carina absent, areola not distinctly margined, areolar area rugose, lateral areas rugose posteriorly, granulate anteriorly. Wings: fore wing vein r shorter than vein 3RSa, vein 1cu-a beyond vein 1M; hind wing vein SC+R present, vein M+CU shorter than vein 1M. Metasoma: first tergum longitudinally costate-rugose; second tergum longitudinally costate-granulate; anterior transverse groove present, slightly sinuate; posterior transverse groove present; third tergum costate basally, smooth apically; terga 4-7 smooth; ovipositor as long as metasoma.

#### Holotype female.

Top label (white, printed) - COSTA RICA, Puntar. [;] Golfo Dulce, 3Km S [;] Rincon, 10m [;] III-V/1989, Hanson; second label (red, partially printed and hand written) - HOLOTYPE [;] Heterospilus [;] boharti [;] P. Marsh. Deposited in ESUW.

#### Paratype.

1 ♀, Costa Rica: Limon [;] Sector Cocori, 100m [;] 30km N Cariari, i.1995 [;] E. Rojas, Malaise, #4526 [;] L.N. 286000-567500 (ESUW).

#### Comments.

The rugose and dull face is distinctive for this species.

#### Etymology.

Named for the late Richard M. Bohart, who guided me as a graduate student at the University of California, Davis.

**Figure 18.**
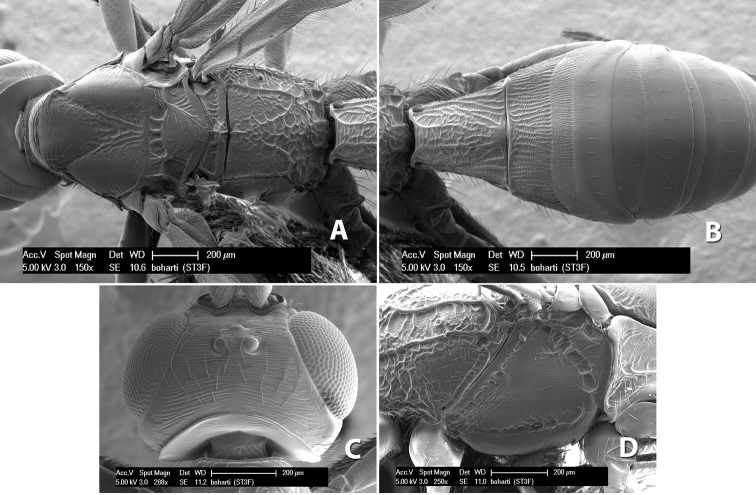
*Heterospilus boharti* Marsh, sp. n., paratype.

**Figure 19.**
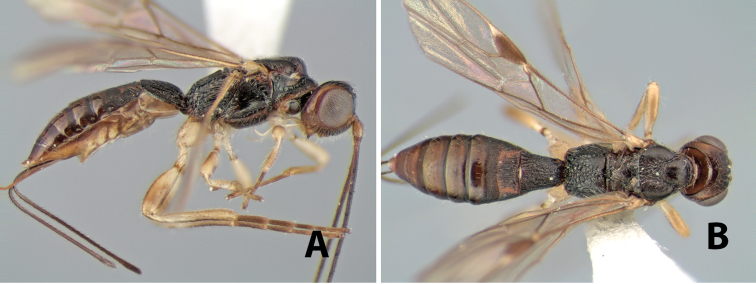
*Heterospilus boharti* Marsh, sp. n., holotype.

### Heterospilus brethesi

Marsh sp. n.

http://zoobank.org/7A6A7FF5-A96C-4AE5-9B75-3A6D946B3539

http://species-id.net/wiki/Heterospilus_brethesi

Figure 20

#### Female.

Body size: 3.0 mm. Color: head brown to light brown; scape yellow without lateral brown stripe, flagellum brown with apical 3–5 flagellomeres white; mesosoma dark brown; metasoma dark brown, apical terga slightly lighter brown; wing veins including stigma brown; legs yellow. Head: vertex transversely striate; frons transversely striate; face granulate; temple in dorsal view somewhat broad, width about 1/2 eye width; malar space greater than 1/4 eye height; ocell-ocular distance about 2.5 times diameter of lateral ocellus; 21–23 flagellomeres. Mesosoma: mesoscutal lobes granulate; notauli scrobiculate, meeting at scutellum in triangular costate rugose area; scutellum granulate; prescutellar furrow with 3 cross carinae; mesopleuron granulate; precoxal sulcus smooth, shorter than mesopleuron; venter granulate; propodeum with basal median areas margined, granulate, basal median carina absent or, if present, very short, areola distinctly margined, areolar area rugose, lateral areas entirely rugose. Wings: fore wing vein r shorter than vein 3RSa, vein 1cu-a beyond vein 1M; hind wing vein SC+R present, vein M+CU shorter than vein 1M. Metasoma: first tergum longitudinally costate, apical width equal to length; second tergum longitudinally costate; anterior transverse groove present, straight; posterior transverse groove present; third tergum costate basally, smooth apically; terga 4–7 smooth; ovipositor equal to 3/4 length of metasoma.

#### Holotype female.

Top label (white, printed) - Costa Rica: Limon [;] 30km N Cariari, 100m [;] Sector Cocori, Malaise [;] iii.1995, E. Rojas #4524 [;] L.N. 286000-567500; second label (red, partially printed and hand written) - HOLOTYPE [;] Heterospilus [;] brethesi [;] P. Marsh. Deposited in ESUW.

#### Paratype.

1 ♀, Costa Rica: Puntarenas [;] San Vito, Las Cruces [;] Wilson Botanical Gardens [;] 18-22.iii.1990, 1150m [;] J.S. Noyes (ESUW).

#### Comments.

The dark brown body and the white apical flagellomeres are distinctive for this species.

#### Etymology.

Named for the Argentinean hymenopterist, J. Brèthes.

**Figure 20.**
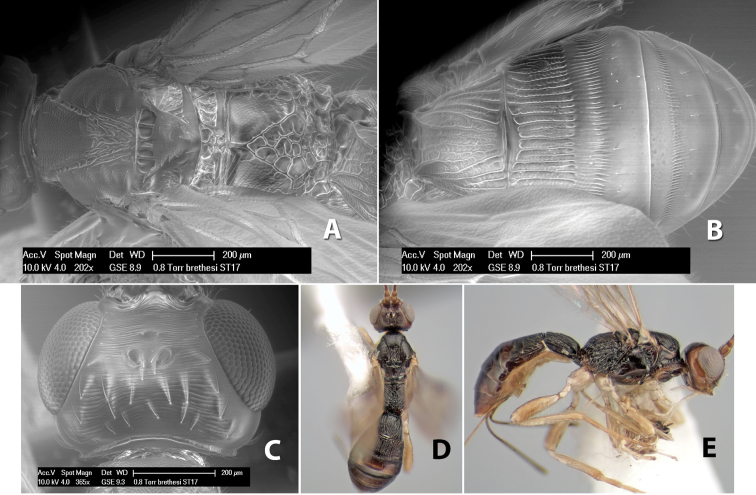
*Heterospilus brethesi* Marsh, sp. n., holotype.

### Heterospilus bribri

Marsh sp. n.

http://zoobank.org/909472AA-73A4-456B-899E-A1B4684ACAA1

http://species-id.net/wiki/Heterospilus_bribri

Figure 21

#### Female.

Body size: 2.5–3.5 mm. Color: head with vertex and frons brown, face and eye orbits yellow; scape yellow without lateral longitudinal brown stripe, flagellum brown; mesosoma brown, pronotum often light brown; metasomal tergum 1 dark brown, tergum 2 brown to honey yellow, terga 3–6 dark brown basally, yellow apically, tergum 7 yellow; wing veins brown, stigma bicolored brown with yellow apex; legs yellow. Head: vertex transversely costate; frons transversely costate; face smooth; temple in dorsal view narrow, width less than 1/2 eye width; malar space greater than 1/4 eye height; ocell-ocular distance about 2.5 times diameter of lateral ocellus; 26–31 flagellomeres. Mesosoma: mesoscutal lobes granulate; notauli scrobiculate, meeting at scutellum in triangular costate-rugose area; scutellum granulate; prescutellar furrow with 3 cross carinae; mesopleuron granulate; precoxal sulcus weakly scrobiculate or smooth, shorter than mesopleuron; venter granulate; propodeum with basal median areas margined, granulate, basal median carina present, areola distinctly margined, areolar area rugose, lateral areas entirely granulate. Wings: fore wing vein r shorter than vein 3RSa, vein 1cu-a interstitial or very slightly beyond vein 1M; hind wing vein SC+R present, vein M+CU shorter than vein 1M. Metasoma: first tergum costate, apical width less than length; second tergum costate-granulate, width about 4 times length; anterior transverse groove present, sinuate; posterior transverse groove weakly indicated or absent; third tergum entirely granulate; terga 4-7 granulate at base, smooth apically; ovipositor as long as metasoma.

#### Holotype female.

Top label (white, printed) - COSTA RICA: [;] Puntar. Golfo Dulce [;] 24km W Piedras Blancas [;] 200m, vi-viii 1989 [;] Hanson; second label (red, partially printed and hand written) - HOLOTYPE [;] Heterospilus [;] bribri [;] P. Marsh. Deposited in ESUW.

#### Paratypes.

1 ♀, Costa Rica: Puntarenas, [;] R.F. Golfo Dulce, 5km. [;] W. Piedras Blancas, 100m [;] xi–xii.1991, P. Hanson, [;] Malaise nr. second growth (ESUW). 4 ♀♀, COSTA RICA-Heredia Prov. [;] La Selva Biological Station [;] 10°26'N, 84°01'W, 100m [;] Canopy fogging 19, 31 and 32 [;] 8.x.1994, 2.xi.1994 and 3.xi.1994 [;] Project ALAS (FCK19, 31 and 32) (ESUW). 1 ♀, Costa Rica: Heredia [;] 3km. S. Puerto Viejo [;] OTS, La Selva, 100m [;] xi.1992, P. Hanson (ESUW). 1 ♀, Costa Rica: Guanacaste, ACT [;] Bagaces, P.N. Palo Verde, 212m [;] Sec. Palo Verde, Cerro Guayacan [;] 13.ix–13.x.1999, I. Jimenez, Malaise [;] L.N. 259350-389600 #53499 (ESUW). 2 ♀♀, top label - Costa Rica: Guanacaste [;] Santa Rosa Natl. Park [;] 300m, ex. Malaise trap [;] Site #: blank [;] Dates: 14.viii–6.ix.1986 and 7-28.xii.1985 [;] I.D. Gauld & D. Janzen; second label - [SE] Bosque San Emilio [;] 50yr old deciduous forest [;] [C] more or less fully [;] shaded as possible (ESUW). 1 ♀, top label - Costa Rica: Guanacaste [;] Santa Rosa Natl. Park [;] 300m, ex. Malaise trap [;] Site #: 10 [;] Dates: 26.x–16.xi.1985 [;] I.D. Gauld & D. Janzen; second label - [BH] Bosque Humedo [;] mature evergreen dry forest [;] [C] more or less fully [;] shaded as possible (ESUW). 1 ♀, Costa Rica: Guanacaste [;] Santa Rosa National Pk. [;] 300m, Malaise SE-6-C [;] Bosque San Emilio, [;] deciduous forest [;] 50yr. old, Ian Gauld [;] 5.vii.1986, full shade (ESUW). 2 ♀♀, COSTA RICA: Puntarenas [;] RF Golfo Dulce, el 200m [;] 24km W Piedras Blancas [;] P. Hanson vii and ix.1992 (TAMU). 1 ♀, COSTA RICA, Puntar. [;] Golfo Dulce, 3km [;] SW. Rincon, 10m [;] III-VI 1990, Hanson (MICR). 1 ♀, COSTA RICA, Puntar. [;] Golfo Dulce, 24km W. [;] PiedrasBlancas, 200m [;] XII.89-III.90 Hanson (MICR). 1 ♀, Sirena, Osa Pen. [;] VII.77 Cos. Rica [;] D. H. Janzen (AEIC). 5 ♀♀, S.RosaPark, Guan. [;] C. Rica. 25 Jun 77, 15 Jun 77, 16 Nov. 77, 14 Nov 77 and 20 Nov 77 [;] D.H. Janzen [;] Riparian and Dry Hill (AEIC).

#### Comments.

This species is distinguished by the 3 cross carinae in the prescutellar furrow and the granulate metasomal terga.

#### Etymology.

Named for the Bribri, an indigenous people group of Costa Rica.

**Figure 21.**
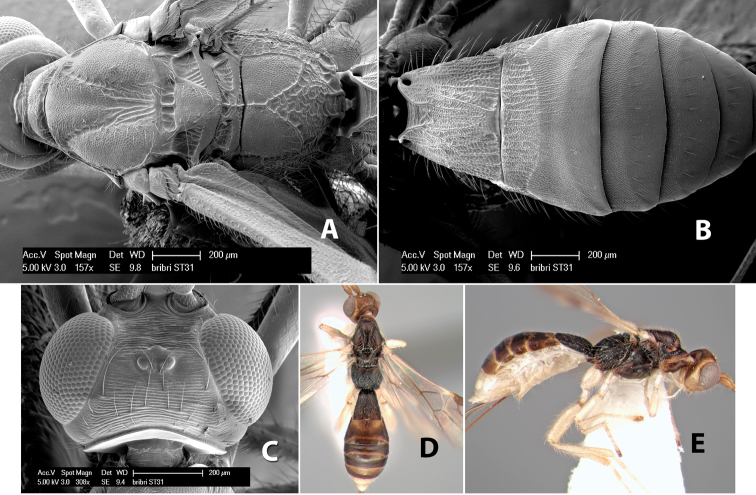
*Heterospilus bribri* Marsh, sp. n.: **A–C** paratype **D–E** holotype.

### Heterospilus bruesi

Marsh sp. n.

http://zoobank.org/D37811F8-CC38-4212-8605-445EEBD1933E

http://species-id.net/wiki/Heterospilus_bruesi

Figure 22

#### Female.

Body size: 3.0 mm. Color: head with vertex and frons brown, face and temple yellow; scape yellow without lateral longitudinal brown stripe, flagellum brown (broken); mesosoma dark brown; metasomal terga 1–4 dark brown, terga 5–7 yellow; legs yellow; wing veins including stigma brown. Head: vertex weakly transversely striate; frons transversely striate; face striate; temple in dorsal view broad, not sloping inward behind eye, width greater than 1/2 eye width; malar space less than 1/4 eye height; ocell-ocular distance slightly less than 2.5 times diameter of lateral ocellus; ? flagellomeres (broken). Mesosoma: mesoscutal lobes granulate; notauli scrobiculate, meeting at scutellum in triangular costate area; scutellum granulate; prescutellar furrow with 3 cross carinae; mesopleuron smooth; precoxal sulcus smooth, shorter than mesopleuron; venter granulate; propodeum with basal median areas granulate, distinctly margined, basal median carina absent, areola distinctly margined, areolar area rugose, lateral areas entirely rugose. Wings: fore wing vein r shorter than vein 3RSa, vein 1cu-a beyond vein 1M; hind wing vein SC+R present, vein M+CU equal in length to vein 1M. Metasoma: first tergum longitudinally costate, length equal to apical width; second tergum costate, about 4 times as wide as long; anterior transverse groove present, sinuate; posterior transverse groove absent; third tergum costate basally, smooth apically; terga 4–7 smooth; ovipositor about 3/4 length of metasoma.

#### Holotype female.

Top label (white, printed) - COSTA RICA - Heredia Prov. [;] La Selva Biological Station [;] 10°26'N, 84°01'W, 100m [;] Malaise trap 12, #397 [;] 30.vi.1995 [;] Project ALAS (M.12.397); second label (red, partially printed and hand written) - HOLOTYPE [;] Heterospilus [;] bruesi [;] P. Marsh. Deposited in ESUW.

#### Paratypes.

Known only from the holotype.

#### Comments.

The broad temple and sinuate anterior transverse groove of metasomal tergum 2 will distinguish this species.

#### Etymology.

Named for C. T. Brues who described many Braconidae in the early 1900s.

**Figure 22 A-E.**
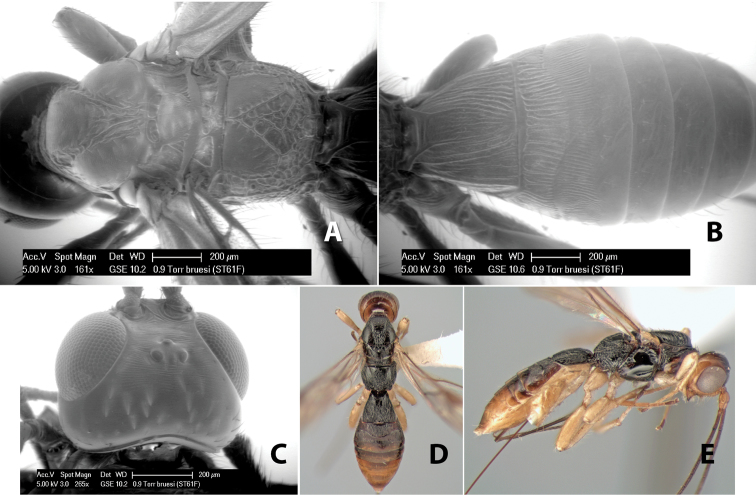
*Heterospilus bruesi* Marsh, sp. n., holotype.

### Heterospilus brullei

Marsh sp. n.

http://zoobank.org/F2F194C3-7FAB-4B2D-9729-25CB465D3B0F

http://species-id.net/wiki/Heterospilus_brullei

Figure 23

#### Female.

Body size: 3.5 mm. Color: head dark brown, face light brown; scape yellow with lateral longitudinal brown stripe, flagellum brown, apical flagellomeres white except last 3–5 brown; mesosoma entirely dark brown except propleuron occasionally lighter; metasoma dark brown to black, tergum 6 yellow, tergum 7 light brown; wing veins including stigma brown; legs yellow. Head: vertex transversely costate; frons weakly striate; face smooth; temple in dorsal view narrow, not bulging beyond eye, less than 1/2 eye width; malar space equal to 1/4 eye height; ocell-ocular distance about twice diameter of lateral ocellus; 32 flagellomeres. Mesosoma: mesoscutal lobes smooth and shining; notauli scrobiculate, meeting at scutellum in broadly triangular costate area; scutellum smooth; prescutellar furrow with 3–5 cross carinae; mesopleuron smooth; precoxal sulcus smooth, shorter than mesopleuron; venter smooth; propodeum with basal median areas distinctly margined, rugose or costate, basal median carina short but distinct, areola distinctly margined, areolar area rugose, lateral areas entirely rugose or costate. Wings: fore wing vein r only slightly shorter than vein 3RSa, vein 1cu-a beyond vein 1M; hind wing vein SC+R present, vein M+CU shorter than vein 1M. Metasoma: first tergum costate, apical width about equal to length; second tergum costate, narrow with apical width about 4 times length; anterior transverse groove present, sinuate; posterior transverse groove present; third tergum costate basally, granulate apically; terga 4–7 weakly granulate; ovipositor equal to length of metasoma.

#### Holotype female.

Top Label (white, partially printed and hand written) - Costa Rica: Guanacaste [;] Santa Rosa Natl. Park [;] 300m, ex. Malaise trap [;] Site #: (blank) [;] Dates: 14.viii–6.ix.1986 [;] I.D. Gauld & D. Janzen; second label (white, printed) - [SE] Bosque San Emilio [;] 30yr old deciduous forest [;] [C] more or less fully [;] shaded as possible; third label (red, partially printed and hand written) - HOLOTYPE [;] Heterospilus [;] brullei [;] P. Marsh. Deposited in ESUW.

#### Paratypes.

1 ♀, same data as holotype, dates of 16.xi–7.xii.1985 (ESUW). 4 ♀♀, S.RosaPark,Guan, Guan. [;] C. Rica, 6 Jul 77, 11 Jul 77, 25 Jul 77 and 4 Dec 77 [;] D.H. Janzen [;] Riparian and Dry Hill (AEIC).

#### Comments.

This species is distinguished by the smooth and shining mesoscutal lobes, the costate junction of the notauli and the rugose or costate basal median areas of the propodeum.

#### Etymology.

Named for A. Brullé who described a few Braconidae in the early 1800s.

**Figure 23.**
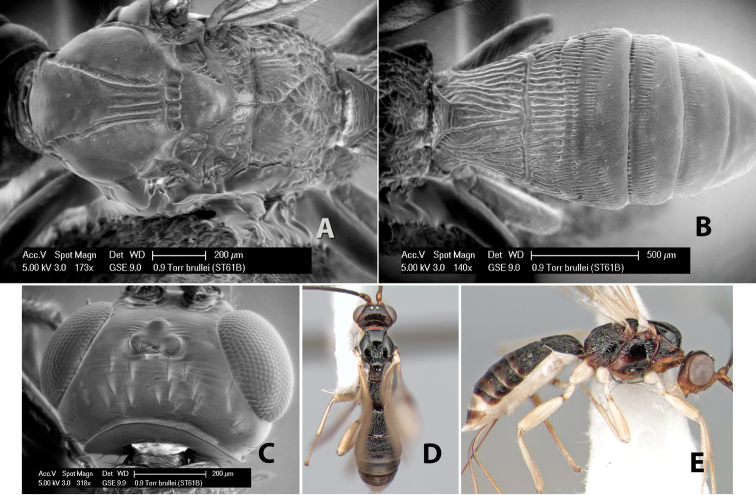
*Heterospilus brullei* Marsh, sp. n.: **A–C** paratype **D–E** holotype.

### Heterospilus cacaoensis

Marsh sp. n.

http://zoobank.org/FC8E38CA-31BE-49B4-A03B-6102546B3E30

http://species-id.net/wiki/Heterospilus_cacaoensis

Figure 24

#### Female.

Body size: 3.5–4.0 mm. Color: head dark brown, eye orbits usually and face partially marked with honey yellow; scape brown with lateral longitudinal dark brown stripe, flagellum brown with apical 8–10 flagellomeres white; mesosoma dark brown, propleuron and venter usually lighter brown; metasoma dark brown, terga 5–7 yellow; wing veins brown, stigma brown with yellow at extreme apex; legs yellow. Head: vertex transversely striate; frons transversely striate; face striate; temple in dorsal view narrow, width less than 1/2 eye width; malar space greater than 1/4 eye height; ocell-ocular distance twice diameter of lateral ocellus; 30-31 flagellomeres. Mesosoma: mesoscutal lobes granulate; notauli scrobiculate, meeting at scutellum in with 2 converging carinae; scutellum granulate; prescutellar furrow with 1 cross carina; mesopleuron granulate; precoxal sulcus scrobiculate, shorter than mesopleuron; venter granulate; propodeum with basal median areas margined, granulate, basal median carina present, short, areola distinctly margined, areolar area areolate-rugose, lateral areas rugose apically, granulate basally, small but distinct tubercle on propodeum just above hind coxa. Wings: fore wing vein r shorter than vein 3RSa, vein 1cu-a beyond vein 1M; hind wing vein SC+R present, vein M+CU shorter than vein 1M. Metasoma: first tergum longitudinally costate, length greater than apical width; second tergum longitudinally costate; anterior transverse groove present, straight; posterior transverse groove present; third tergum costate basally, smooth apically; terga 4-7 smooth; ovipositor equal to 1/2 length of metasoma.

#### Holotype female.

Top label (white, printed) - Costa Rica: Guanacaste [;] Est. Cacao, 1000–1150m [;] ix.1996, I. Villegas, Malaise [;] L.N. 323150-375500 #47559; second label (red, partially printed and hand written) - HOLOTYPE [;] Heterospilus [;] cacaoensis [;] P. Marsh. Deposited in ESUW.

#### Paratypes.

2 ♀♀, same data as holotype with dates of vii.1996, viii.1996 (ESUW). 1 ♀, COSTA RICA: Puntar [;] Golfo Dulce 24km W [;] Piedras Blancas [;] 200m, vii-ix.1990 [;] Col. Paul Hanson (ESUW). 1 ♀, Costa Rica: Cartago [;] Turrialba, CATE [;] 14-15 March 1990 [;] 700m, J.S. Noyes (ESUW). 1 ♀, top label - Costa Rica: Guanacaste [;] W. side Volcan Orosi [;] Estac. Maritza, 600m; second label - GNP Biodiversity Survey [;] 1989, Malaise trap [;] L-N-326900-373000 #6834 (ESUW).

#### Comments.

The single median cross carina in the prescutellar furrow, the white apical flagellomeres and the tubercles on the propodeum just above the hind coxa are distinctive for this species.

#### Etymology.

Named for the locality where the holotype was collected, Cacao Station in Guanacaste Province.

**Figure 24.**
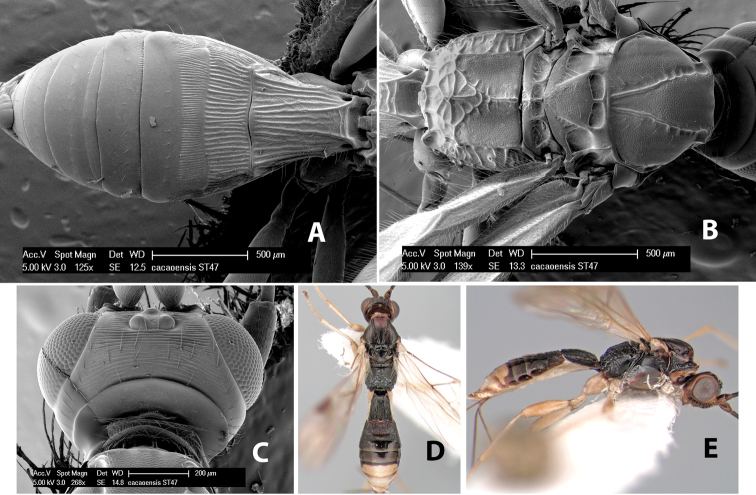
*Heterospilus cacaoensis* Marsh, sp. n.: **A–C** paratype **D–E** holotype.

### Heterospilus cachiensis

Marsh sp. n.

http://zoobank.org/47526AAC-43AA-47DC-AC5A-456FED0AEAD8

http://species-id.net/wiki/Heterospilus_cachiensis

Figure 25

#### Female.

Body size: 2.0–2.5 mm. Color: head yellow; scape yellow without lateral brown stripe, flagellum brown with apical 4-5 flagellomeres white; mesosoma dark brown except mesoscutum light brown; metasomal terga 1–2 dark brown, terga 3–4 dark brown basally, yellow apically, terga 5–7 yellow; wing veins brown, stigma brown with yellow apex and base; legs yellow. Head: vertex transversely striate medially, smooth near eyes; frons smooth; face smooth; temple in dorsal view narrow, width less than 1/2 eye width; malar space greater than 1/4 eye height; ocell-ocular distance about 2.5 times diameter of lateral ocellus; 21 flagellomeres. Mesosoma: mesoscutal lobes granulate; notauli scrobiculate, meeting at scutellum in triangular costate area; scutellum granulate; prescutellar furrow with 3 cross carinae; mesopleuron granulate; precoxal sulcus scrobiculate, usually as long as mesopleuron; venter granulate; propodeum with basal median areas margined, granulate, basal median carina present, areola distinctly margined, areolar area rugose, lateral areas entirely rugose. Wings: fore wing vein r nearly equal to vein 3RSa, vein 1cu-a beyond vein 1M; hind wing vein SC+R present, vein M+CU shorter than 1M. Metasoma: first tergum longitudinally costate, apical width 1/2 length; second tergum longitudinally costate; anterior transverse groove present, straight; posterior transverse groove present; third tergum costate basally, smooth apically; terga 4–7 smooth; ovipositor equal to length of metasomal terga 1+2 combined.

#### Holotype female.

Top label (white, printed) - Costa Rica: Cartago [;] 2km. NE Cachi [;] 1200m, vii-ix.1995 [;] P. Hanson, Malaise; second label (red, partially printed and hand written) - HOLOTYPE [;] Heterospilus [;] cachiensis [;] P. Marsh. Deposited in ESUW.

#### Paratypes.

1 ♀, same data as holotype (ESUW). 1 ♀, COSTA RICA-Heredia Prov. [;] La Selva Biological Station [;] 10°26'N, 84°01'W, 100m [;] Malaise trap 14, #288 [;] 1.xii.1993 [;] Project ALAS (M, 14 28810) (ESUW).

#### Comments.

The long precoxal sulcus, which usually extends to posterior margin of the mesopleuron, is distinctive for this species.

#### Etymology.

Named after the type locality of Cachi in Cartago Province.

**Figure 25.**
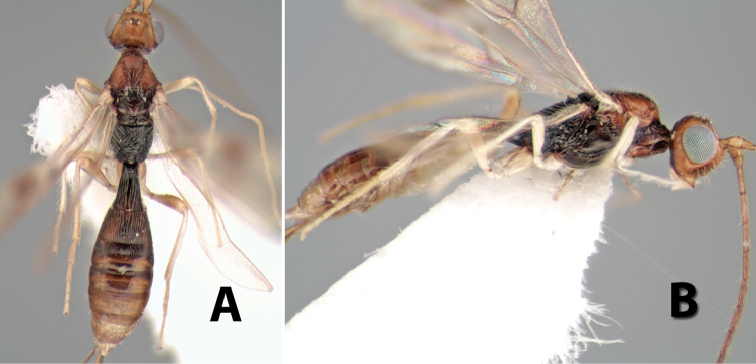
*Heterospilus cachiensis* Marsh, sp. n., holotype.

### Heterospilus cameroni

Marsh sp. n.

http://zoobank.org/449ADFBA-349B-4679-82C9-E353C1AF9AD1

http://species-id.net/wiki/Heterospilus_cameroni

Figure 26

#### Female.

Body size: 2.5–3.0 mm. Color: head with vertex and frons brown, face and eye orbits light brown; scape yellow without lateral longitudinal brown stripe, flagellum brown, basal 1–3 flagellomeres lighter; mesosoma dark brown; metasoma dark brown, apical terga 4–7 light brown; legs yellow; wing veins including stigma brown. Head: vertex transversely striate; frons transversely striate; face smooth; temple in dorsal view narrow, sloping behind eye, width less than 1/2 eye width; malar space short, equal to or less than 1/4 eye height; ocell-ocular distance about 1.5 times diameter of lateral ocellus; 21–25 flagellomeres. Mesosoma: mesoscutal lobes granulate; notauli scrobiculate, meeting at scutellum in triangular rugose area; scutellum smooth; prescutellar furrow with 1 cross carina; mesopleuron smooth; precoxal sulcus smooth or weakly scrobiculate, shorter than mesopleuron; venter smooth; propodeum with basal median areas margined, granular, basal median carina absent, areola not distinctly margined, areolar area rugose, lateral areas entirely rugose. Wings: fore wing vein r slightly shorter or equal to length of vein 3RSa, vein 1cu-a beyond vein 1M; hind wing vein SC+R present, vein M+CU equal to length of vein 1M. Metasoma: first tergum longitudinally costate, apical width equal to length; second tergum longitudinally costate; anterior transverse groove present, straight; posterior transverse groove present; third tergum costate basally, smooth apically; terga 4–7 smooth; ovipositor equal to 1/2 length of metasoma.

#### Holotype female.

Top label (white, printed) - COSTA RICA-Heredia Prov. [;] La Selva Biological Station [;] 10°26'N, 84°01'W. 100m [;] Canopy fogging 34 [;] 10.xi.1994 [;] Project ALAS (FVK34); second label (red, partially printed and hand written) - HOLOTYPE [;] Heterospilus [;] cameroni [;] P. Marsh. Deposited in ESUW.

#### Paratypes.

1 ♀, Costa Rica: Guanacaste [;] Est. Biol. Maritza, 600m [;] i.1997, C. Zuniga, Malaise [;] L.N. 326900-373000 #47557 (ESUW). 1 ♀, top label - Costa Rica: Guanacaste [;] Santa Rosa Natl. Park [;] 300m, ex. Malaise trap [;] Site #: H-1-O [;] Dates: 26.vii–14.viii.1986 [;] I.D. Gauld & D. Janzen; second label - [H] open regenerating [;] woodland <10 years old [;] [O] in clearing, fully [;] isolated part of day (ESUW). 1 ♀, Costa Rica: Limon, ACLAC [;] Central, R.B. Hitoy Cerere [;] Est. Hitoy Cerere, Send. [;] Catarata, 90m, Red de Golpe [;] 10.vii.1999, F. Umada [;] L.N. 184600-643400 #53857 (ESUW).

#### Comments.

The short malar space is distinctive for this species.

#### Etymology.

Named for P. Cameron who described many tropical Braconidae in the late 1880s and early 1900s.

**Figure 26.**
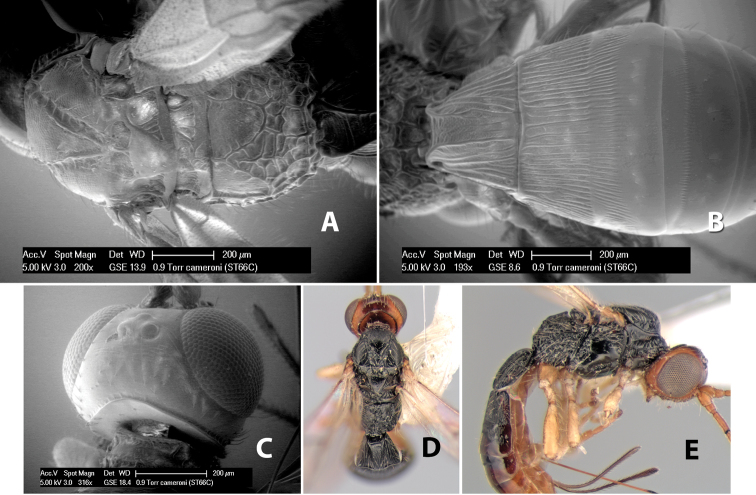
*Heterospilus cameroni* Marsh, sp. n., holotype.

### Heterospilus cartagoensis

Marsh sp. n.

http://zoobank.org/8E4BEEE1-3200-4815-B76E-E47933E35A95

http://species-id.net/wiki/Heterospilus_cartagoensis

Figure 27

#### Female.

Body size: 3.0 mm. Color: head yellow; scape light brown, with weak lateral longitudinal brown stripe, flagellum brown (broken); mesosoma with mesoscutum, mesopleuron and venter yellow, pronotum, upper portion of mesopleuron, and propodeum dark brown; metasomal tergum 1 dark brown, terga 2–5 brown medially, yellow laterally, terga 6–7 yellow; legs yellow; wing veins including stigma brown. Head: vertex transversely costate; frons transversely costate; face rugose; temple in dorsal view slightly bulging, width less than 1/2 eye width; malar space greater than 1/4 eye height; ocell-ocular distance about twice diameter of lateral ocellus; antennae broken. Mesosoma: mesoscutal lobes granulate; notauli scrobiculate, meeting at scutellum in triangular rugose area; scutellum smooth; prescutellar furrow with 3 cross carinae; mesopleuron smooth; precoxal sulcus smooth, shorter than mesopleuron; venter weakly granulate or smooth; propodeum with basal median areas granulate, distinctly margined, basal median carina distinct, areola not distinctly margined, areolar area rugose, lateral areas entirely rugose. Wings: fore wing vein r shorter than vein 3RSa, vein 1cu-a beyond vein 1M; hind wing vein SC+R present, vein M+CU shorter than vein 1M. Metasoma: first tergum costate, length longer than apical width; second tergum costate, width less than 3 times length; anterior transverse groove present, straight; posterior transverse groove very weakly indicated medially only; third tergum costate basally, smooth apically; terga 4-7 smooth; ovipositor longer than metasoma.

#### Holotype female.

Top label (white, printed) - Costa Rica: Cartago [;] Dulce Nombre, Vivero [;] Linda Vista, 1400m [;] vi-viii.1993, Hanson. Second label (red, partially printed and hand written) - HOLOTYPE [;] Heterospilus [;] cartagoensis [;] P. Marsh. Deposited in ESUW.

#### Paratypes.

Known only from the holotype.

#### Comments.

The yellow head and mesoscutum are distinctive for this species.

#### Etymology.

Named for Cartago Province where the holotype was collected.

**Figure 27.**
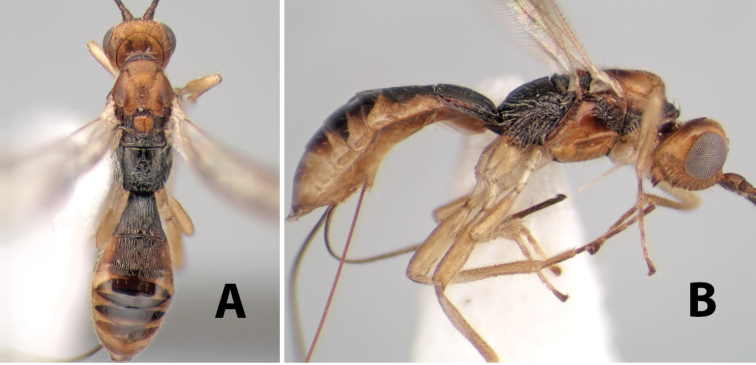
*Heterospilus cartagoensis* Marsh, sp. n., holotype.

### Heterospilus chaoi

Marsh sp. n.

http://zoobank.org/B3E3E78C-2B8F-4B24-B99F-06C1644E3E05

http://species-id.net/wiki/Heterospilus_chaoi

Figure 28

#### Female.

Body size: 3.0–3.5 mm. Color: head yellow to light brown, occasionally vertex, frons and face light brown with eye orbits and malar space yellow; scape yellow without lateral brown stripe; flagellum brown, sometimes basal flagellomeres yellowish; mesosoma usually dark brown, often with mesoscutum, propleuron and venter lighter brown or honey yellow; metasomal terga 1–3 usually dark brown, remainder of terga lighter brown or honey yellow (one paratype has the body entirely honey yellow); wing veins brown, stigma brown with yellow apex; legs yellow. Head: vertex transversely costate; frons transversely costate; face rugose; temple in dorsal view broad, not sloping behind eye, width greater than 1/2 eye width; malar space greater than 1/4 eye height; ocell-ocular distance 2.0–2.5 times diameter of lateral ocellus; 25–26 flagellomeres. Mesosoma: mesoscutal lobes granulate; notauli scrobiculate, meeting at scutellum in triangular rugose area; scutellum smooth; prescutellar furrow with 1 cross carina; mesopleuron granulate; precoxal sulcus scrobiculate, shorter than mesopleuron; venter smooth; propodeum with basal median areas margined, granulate, basal median carina present, areola not distinctly margined, areolar area rugose, lateral areas entirely rugose. Wings: fore wing vein r shorter than vein 3RSa, vein 1cu-a beyond vein 1M; hind wing vein SC+R present, vein M+CU shorter than vein 1M. Metasoma: first tergum longitudinally costate; second tergum longitudinally costate; anterior transverse groove present, straight; posterior transverse groove weak or absent; third tergum granulate or costate basally, smooth apically; terga 4–7 smooth; ovipositor longer than metasoma.

#### Holotype female.

Top label (partially printed and hand written) - Costa Rica: Guanacaste [;] Santa Rosa Natl. Park [;] 300m, ex. Malaise trap [;] Site #: (blank) [;] Dates: 7–28.xii.1985 [;] I.D. Gauld & D. Janzen; second label (white, printed) - [SE] Bosque San Emilio [;] 50yr old deciduous forest [;] [C] more or less fully [;] shaded as possible; third label (red, partially printed and hand written) - HOLOTYPE [;] Heterospilus [;] chaoi [;] P. Marsh. Deposited in ESUW.

#### Paratypes.

1 ♀, same data as holotype with date of 26.xii.85–18.i.1986 (ESUW). 1 ♀, COSTA RICA-Heredia Prov. [;] La Selva Biological Station [;] 10°26'N, 84°01'W, 100m [;] Malaise trap 12, #382 [;] 15.iii.1994 [;] Project ALAS (M, 12, 382) (ESUW). 2 ♀♀, top label - Costa Rica: Guanacaste [;] Santa Rosa National Pk. [;] 300m, Malaise, Ian Gauld [;] 18.x–8.xi.1986 and 6–27.ix.1986; second label - Bosque San Emilio [;] 50yr old deciduous [;] forest, full shade (ESUW). 1 ♀, top label - COSTA RICA, Heredia: [;] Est. Biol. La Selva, 50- [;] 150m, 10°26'N, 84°01'W [;] Apr 1998, INBio-OET; second label - 16 Abril 1998 [;] Bosque suampo [;] M/18/706 (INBC).

#### Comments.

The broad temple and long ovipositor are distinctive for this species.

#### Etymology.

Named for the late Chinese braconidologist, Hsiu-Fu Chao in remembrance of my two enjoyable visits to his laboratory in Fujien, China.

**Figure 28.**
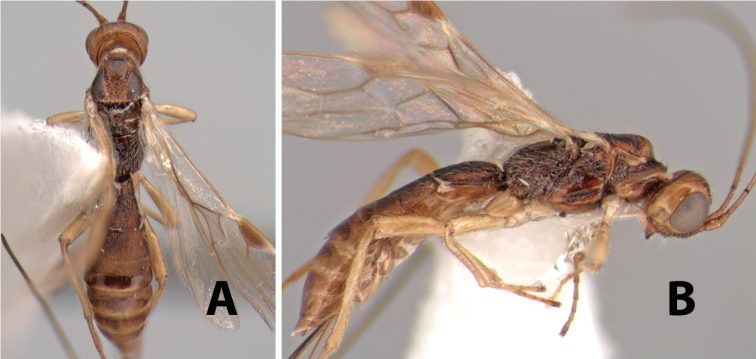
*Heterospilus chaoi* Marsh, sp. n., holotype.

### Heterospilus corcovado

Marsh sp. n.

http://zoobank.org/D3F672E9-FFAA-4907-B8EC-19DC30FA688B

http://species-id.net/wiki/Heterospilus_corcovado

Figure 29

#### Female.

Body size: 3.0–4.0 mm. Color: head brown to dark brown; scape honey yellow, usually with lateral longitudinal brown stripe, flagellum brown, apical 8–11 flagellomeres white; mesosoma usually entirely dark brown or black, occasionally mesoscutal lobes and venter lighter brown; metasomal terga 1–4 dark brown or black, terga 5–7 yellow; wing veins including stigma brown; legs yellow, occasionally femora brown on apical half. Head: vertex transversely costate; frons transversely costate; face smooth or occasionally weakly striate; temple in dorsal view narrow, width less than 1/2 eye width; malar space greater than 1/4 eye height; ocell-ocular distance slightly more than 2.5 times diameter of lateral ocellus; 29–35 flagellomeres. Mesosoma: mesoscutal lobes granulate; notauli scrobiculate, meeting at scutellum in triangular costate or costate-rugose area; scutellum granulate; prescutellar furrow with 1 cross carina; mesopleuron granulate; precoxal sulcus smooth, shorter than mesopleuron; venter granulate; propodeum with basal median areas margined, granulate, basal median carina present, areola not distinctly margined, areolar area rugose, lateral areas rugose posteriorly, granulate anteriorly, area above hind coxa with distinct tubercle. Wings: fore wing vein r shorter than vein 3RSa, vein 1cu-a beyond vein 1M; hind wing vein SC+R present, vein M+CU slightly shorter than vein 1M. Metasoma: first tergum longitudinally costate, length about 1.5 times apical width; second tergum costate, about 4 times as wide as length; anterior transverse groove present, sinuate; posterior transverse groove present, occasionally weak and partially absent; third tergum smooth entirely, rarely weakly granulate at base; terga 4-7 smooth; ovipositor as long as or longer than metasoma.

#### Holotype female.

Top label (white, printed) - Costa Rica: Puntarenas, ACO [;] Golfito, P.N. Cordovado[sic], 745m [;] Est. Agujas, Cerro Rincon [;] 15.v–15.vi.1999, J. Azofeifa [;] L.S. 276900-521500 #52744 [;] Malaise trap; second label 9 red, partially printed and hand written) - HOLOTYPE [;] Heterospilus [;] corcovado [;] P. Marsh. Deposited in ESUW.

#### Paratypes.

1 ♀, COSTA RICA: Puntar [;] Golfo Dulce 24km W [;] Piedras Blancas [;] 200m, vii-ix 1990 [;] Col. Paul Hanson (ESUW). 1 ♀, COSTA RICA, Puntarenas [;] San Vito, Jardin Bot. [;] Las Cruces, VII-VIII/89 [;] 1200m, Col. P. Hanson (ESUW). 1 ♀, COSTA RICA: Limon [;] 16km W. Guapiles [;] 400m, i-iv.1991 [;] col. Paul Hanson (ESUW). 1 ♀, Costa Rica: Limon, Sec. Cocori [;] 30Km al N> Cariari, 100m [;] xii.1994, E. Rojas, Malaise [;] L.N. 286000-567500 #4525 (ESUW). 1 ♀, COSTA RICA: Puntarenas [;] RF Golfo Dulce, el 200m [;] 24km W Piedras Blancas [;] P. Hanson xii.1992 (TAMU). 1 ♀, Est. Cuatro Esquinas, 0m, [;] P.N. Tortuguero, Prov. [;] Limon, COSTA RICA. [;] R. Delgado, Nov 1991. [;] L-N-280000,590500 (INBC).

#### Comments.

This species is distinguished by the single cross carina in the prescutellar furrow and the distinct tubercle postero-laterally on the propodeum just above the hind coxa.

#### Etymology.

Named for Corcovado National Park where the holotype was collected.

**Figure 29.**
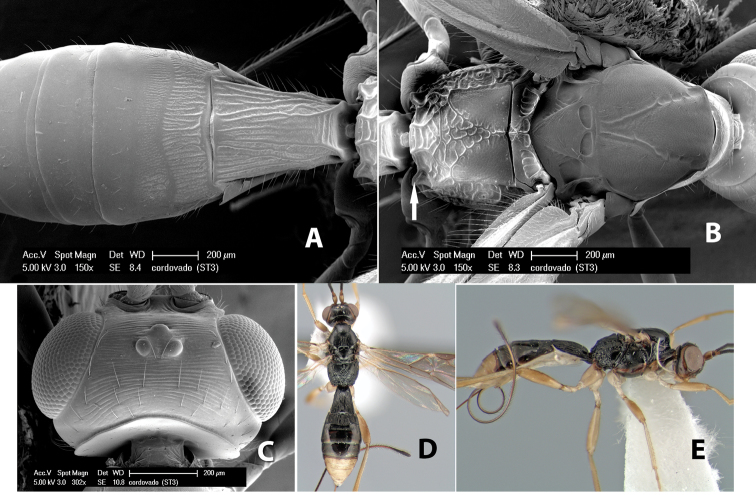
*Heterospilus corcovado* Marsh, sp. n.: **A–C** paratype **D–E** holotype.

### Heterospilus corrugatus

Marsh sp. n.

http://zoobank.org/530B045B-AD4C-4D91-A9F2-CA2794FB0B2C

http://species-id.net/wiki/Heterospilus_corrugatus

Figure 30

#### Female.

Body size: 3.0 mm. Color: head with vertex, frons and temple brown, face and eye orbits yellow; scape yellow without lateral brown stripe, flagellum yellow basally to brown apically; mesosoma dark brown, propleuron and lower half of mesopleuron honey yellow; metasoma brown to dark brown; wing veins including stigma brown, fore wing with light brown band from stigma to posterior edge of wing; legs yellow. Head: vertex transversely costate; frons transversely costate; face rugose; temple in dorsal view narrow, width less than 1/2 eye width; malar space greater than 1/4 eye height; ocell-ocular distance about 2.5 times diameter of lateral ocellus; 25 flagellomeres. Mesosoma: mesoscutal lobes strongly rugose-granulate, entirely covered with sparse short setae; notauli scrobiculate, meeting at scutellum in triangular rugose area; scutellum rugose; prescutellar furrow with 3 cross carinae; mesopleuron granulate; precoxal sulcus weakly scrobiculate, nearly smooth; venter granulate; propodeum with basal median areas margined, weakly granulate or smooth, basal median carina absent or extremely short, areola weakly margined, areolar area rugose, lateral areas entirely rugose. Wings: fore wing vein r shorter than vein 3RSa, vein 1cu-a beyond vein 1M; hind wing vein SC+R present, vein M+CU shorter than vein 1M. Metasoma: first tergum longitudinally costate-granulate length slightly greater than apical width; second tergum longitudinally costate; anterior transverse groove present, straight; posterior transverse groove weakly present; third tergum costate at base, granulate apically; terga 4-7 granulate; ovipositor as long as metasomal terga 1 and 2 combined.

#### Holotype female.

Top label (white, partially printed and hand written) - Costa Rica: Guanacaste [;] Santa Rosa Natl. Park [;] 300m, ex. Malaise trap [;] Site #:SE-7-O [;] Dates: 20.xii.86-10.i.1987 [;] I.D. Gauld & D. Janzen; second label (white, printed) - [SE] Bosque San Emilio [;] 50yr old deciduous forest [;] [O] in clearing fully [;] isolated part of day; third label (red, partially printed and hand written) - HOLOTYPE [;] Heterospilus [;] corrugatus [;] P. Marsh. Deposited in ESUW.

#### Paratypes.

3 ♀♀, S.RosaPark, Guan. [;] C. Rica 14 Sep 77, 16 Oct 77 and 19 Oct 77 [;] D.H. Janzen [;] Riparian (AEIC).

#### Comments.

The strongly rugose and granulate mesoscutum, the entirely hairy mesoscutal lobes and the granulate metasomal terga 3 and 4 are distinctive for this species.

#### Etymology.

The species name is from the Latin *corrugatus* meaning wrinkled or ridged in reference to the rugose mesoscutum.

**Figure 30.**
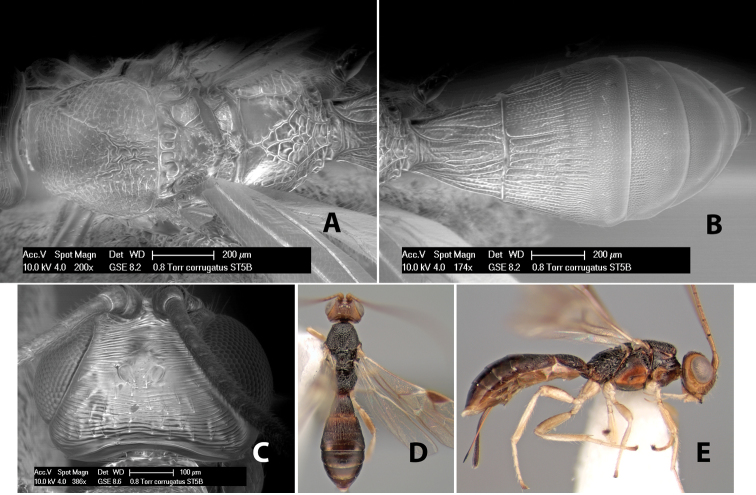
*Heterospilus corrugatus* Marsh, sp. n., holotype.

### Heterospilus costaricensis

Marsh sp. n.

http://zoobank.org/44D04373-56DD-41C2-BC94-6839ECD0BC38

http://species-id.net/wiki/Heterospilus_costaricensis

Figures 31 , 32

#### Female.

Body size: 3.0–5.0 mm. Color: head yellow, temple and lower portion of face often brown; scape yellow with lateral longitudinal brown stripe; flagellum brown with apical 10–15 flagellomeres white; propleuron bicolored brown and yellow; pronotum dark brown, often lighter along dorsal edge; mesoscutum usually yellow, lateral lobes, meeting of notauli, and scutellum often brown; mesopleuron dark brown, venter usually yellow; propodeum dark brown; metasomal terga 1–3 dark brown, tergum 2 usually yellow medially, tergum 4 brown basally and yellow apically, terga 5–7 yellow with brown laterally; legs yellow, femora brown at apex and on dorsal swelling, tarsi brown; wing veins brown, stigma bicolored brown with yellow apex and base. Head: vertex transversely costate; frons transversely costate; face striate; temple in dorsal view narrow, sloping behind eye, width less than 1/2 eye width; malar space equal to 1/4 eye height; ocell-ocular distance twice diameter of lateral ocellus; 28–35 flagellomeres. Mesosoma: mesoscutal lobes granulate; notauli scrobiculate, meeting at scutellum in rugose triangular area; scutellum smooth; prescutellar furrow with 3–5 cross carinae; mesopleuron smooth; precoxal sulcus scrobiculate, shorter than mesopleuron; venter smooth; propodeum with basal median areas distinctly margined, partially smooth and rugose, basal median carina short but distinct, areola not distinctly margined, areolar area rugose, lateral areas entirely rugose, distinct tubercle present laterally just above hind coxa. Wings: fore wing vein r nearly equal to or slightly shorter than vein 3RSA, vein r and vein 3RSa nearly on same line, not at angle to each other, vein 1cu-a beyond vein 1M; hind wing vein SC+R present, vein M+CU shorter than vein 1M. Metasoma: first tergum longitudinally costate, length greater than apical width; second tergum longitudinal costate, width less than 3 times length; anterior transverse groove present, straight; posterior transverse groove absent or very weakly indicated by shallow line; third tergum entirely smooth; terga 4-7 smooth; ovipositor equal to length of metasoma.

#### Holotype female.

Top label (white, printed) - COSTA RICA: Puntar. [;] Golfo Dulce, 3km [;] S.W. Rincon, 10m [;] IX-XI 1989. Hanson; second label (red, partially printed and hand written) - HOLOTYPE [;] Heterospilus [;] costaricensis [;] P. Marsh. Deposited in ESUW.

#### Paratypes.

6 ♀♀, Costa Rica: Puntarenas [;] R.F. Golfo Dulce, [;] 3km SW. Rincon, 10m, [;] vi.1991, Oct. 1991 and ii.1992, Paul Hanson (ESUW). 5 ♀♀, COSTA RICA: Puntar [;] Golfo Dulce 3km SW [;] Rincon [;] 10m, vii-ix 1990, xii 1989-iii 1990 and iii-v 1989 [;] Col. Paul Hanson (ESUW). 6 ♀♀, Costa Rica: Puntarenas [;] Res. Forestal Golfo Dulce [;] 3km. SW Rincon, 10m [;] xii.1992, iii.1993 and iv.1993, P. Hanson [;] Malaise, primary forest (ESUW). 3 ♀♀, COSTA RICA: [;] Puntar [;] Golfo Dulce, 3km [;] SW. Rincon, 10m [;] VI-VIII 1989, Hanson (ESUW). 1 ♀, Costa Rica: Puntarenas [;] R.F. Golfo Dulce, 3km [;] SW Rincon, 10m [;] iii.1993, P. Hanson (ESUW). 2 ♀♀, COSTA RICA: Puntar. [;] Cerro Rincon, 200m [;] S. hito, 745m, ii. [;] 1991, Hanson/Godoy (ESUW). 1 ♀, Costa Rica: Puntarenas [;] R.F.Golfo Dulce, 3km [;] SW Rincon, 10m [;] Malaise-primary forest [;] viii.1991, P. Hanson (ESUW). 2 ♀♀, COSTA RICA: Puntarenas [;] Reserva Forestal Golfo Dulce [;] 3km SW of Rincon, 10m [;] November 1992, P. Hanson [;] primary forest, Malaise trap (ESUW). 4 ♀♀, COSTA RICA: Puntarenas [;] Reserva Forestal Golfo Dulce [;] 3km southwest of Rincon [;] 10m, July 1991, P. Hanson [;] primary forest, Malaise trap (ESUW). 1 ♀, COSTA RICA: Puntarenas [;] Reserva Forestal Golfo Dulce [;] 3km SW Rincon, 10m, primary [;] forest, xii 1992, P. Hanson (ESUW). 5 ♀♀, Costa Rica: Puntarenas [;] R.F. Golfo Dulce, 24km. [;] W. Piedras Blancas, 200m [;] I.1993, VI-1991, VII-IX-1993 and ii.1993, P. Hanson (ESUW). 2 ♀♀, COSTA RICA: [;] Puntar. Golfo Dulce [;] 24km W Piedras Blancas [;] 200m, vi-viii 1989 [;] Hanson (ESUW). 1 ♀, COSTA RICA: Puntar [;] Golfo Dulce, 10km W. [;] Piedras Blancas, 100m [;] VI-VIII 1989 (ESUW). 2 ♀♀, Costa Rica, Puntarenas [;] R.F. Golfo Dulce, 5km. W. [;] Piedras Blancas, 100m [;] VI-VII-1993 and I-1993 (ESUW). 2 ♀♀, Costa Rica: Puntarenas [;] R.F. Golfo Dulce, 5km. [;] W. Piedras Blancas, 100m [;] vi-vii.1991, P. Hanson [;] Malaise, second growth (ESUW). 1 ♀, Costa Rica: Puntarenas [;] Pen. Osa, 23km. N. Pto. [;] Jimenez, La Pulma, 10m [;] VI-VIII-1993, P. Hanson (ESUW). 1 ♀, top label - Costa Rica: Puntarenas [;] Buenos Aires [;] Sendero Los Gigantes [;] Est. Altamira, 1450m; second label - 3-22 February 2000 [;] D. Rubi, Amarilla [;] LS 331700-572200 [;] #54808 (ESUW). 2 ♀♀, Costa Rica: Puntarenas [;] Pe. Osa, 5km. N. Pto. [;] Jimenez, 10m, iii-iv. [;] 1991, P. Hanson, Malaise (ESUW). 1 ♀, Costa Rica: Puntarenas [;] Buenos Aires, Est. Altamira [;] Send. Los Gigantes, 1450m [;] 3-22.ii.2000, D. Rubi, Amarilla [;] L.S.3317700-572200 #54808 (ESUW). 1 ♀, Costa Rica: Puntar. [;] P.N. Corcovado [;] Est. Sirena, 50m [;] x-xii 1990 (ESUW). 3 ♀♀, Costa Rica: Puntarenas [;] San Vito, Estac. Biol. [;] Las Alturas, 1500m [;] xi.1991, xii.1991 and i.1992, Paul Hanson (ESUW). 1 ♀, Costa Rica, Puntarenas [;] Pen. Osa, 5km. N. [;] Puerto Limenez, 10m [;] I-II-1993 P. Hanson (ESUW). 1 ♀, COSTA RICA: Puntarenas [;] San Vito, Las Cruces [;] 1200msnm, VII-IX 1988 [;] Coll. P. Hanson (ESUW). 1 ♀, Costa Rica: Puntarenas [;] Pen. Osa, Puerto [;] Jimenez, 10m [;] x.1990, Paul Hanson (ESUW). 3 ♀♀, Costa Rica: Puntarenas [;] San Vito, Las Cruces [;] Wilson Botanical Gardens [;] 18-22.iii.1990, 1150m [;] J.S. Noyes (ESUW). 4 ♀♀, COSTA RICA: San Jose [;] P.N. Braulio Carillo [;] 9.5km E tunnel, 1000m [;] vii-ix 1989, P. Hanson (ESUW). 2 ♀♀, COSTA RUCA-Heredia Prov. [;] La Selva Biological Station [;] 10°26'N, 84°01W, 100m [;] Canopy fogging 20 and 21 [;] 9.x.1994 and 10.x.1994 [;] Project ALAS (FPM20 and FOT21) (ESUW). 2 ♀♀, Costa Rica: Heredia [;] 3km. S. Puerto Viejo, [;] OTS, La Selva, 100m [;] Oct. 1992 P. Hanson [;] Malaise trap (ESUW). 1 ♀, Costa Rica, Heredia [;] 3km. S. Puerto Viejo [;])TS-La Selva, 100m [;] III-IV-1993, P. Hanson (ESUW). 1 ♀, Costa Rica: Limon, ACLAC [;] Central, R.B. Hitoy Cerere [;] Send. Espavel, 560m [;] 19.v–19.vi.1998, E. Rojas [;] L.S. 400702-570120 #52200 [;] Malaise trap (ESUW). 1 ♀, Costa Rica: Limon, ACLAC [;] Central, R.B. Hitoy Cerere [;] Est, Hitoy Cerere, Sendero [;] Bobocara, 640m, Malaise [;] 17.ix–10.x.1999, F. Umana [;] L.N. 184250-640500 #53496 (ESUW). 1 ♀, COSTA RICA: Limón [;] 16km West Guapiles [;] 400m, April 1989 [;] P. Hanson IV-V (ESUW). 1 ♀, COSTA RICA: [;] Limon [;] 4km NE Bribri [;] 50m, iv-vi 1990 [;] Col. Paul Hanson (ESUW). 1 ♀, Costa Rica: Cartago [;] La Cangreja, 1950m [;] ix-xii.1992 [;] P. Hanson (ESUW). 2 ♀♀, Costa Rica. Carthago Pr. [;] La Cangreja, 1950m [;] 1991: xi, P. Hanson (ESUW). 1 ♀, Costa Rica, Alajuela [;] Estacion Biologica [;] San Ramon, 900m [;] VII-VIII-1995 [;] P. Hanson (ESUW). 1 ♀, Costa Rica: Alajuela [;] 5km W San Ramon [;] 1200m, ii.1997 [;] O.Castro & P.Hanson (ESUW). 1 ♀, top label - Costa Rica: Guanacaste [;] Santa Rosa Natl. Park [;] 300m, ex. Malaise trap [;] Site #: BH-9-O [;] Dates: 8.ii–2.iii.1986 [;] I.D. Gauld & D. Janzen; second label - [BH] Bosque Humedo [;] mature evergreen dry forest [;] [O] in clearing, fully [;] isolated part of day (ESUW). 3 ♀♀, COSTA RICA, Puntar [;] Golfo Dulce, 3km [;] SW. Rincon, 10m [;] III-VI 1990, Hanson (MICR). 1 ♀, COSTA RICA, Guanac [;] Estac. Mengo, SW [;] Volcán Cacao, 1100m [;] 1988-1989 (MICR). 1 ♀, COSTA RICA, Puntarenas [;] R.F. Golfo Dulce, 24kmW [;] Piedras Blancas, 200m [;] IV-V.1991 col. P. Hanson (MICR). 2 ♀♀, COSTA RICA, Puntar. [;] Golfo Dulce, 24km W. [;] PiedrasBlancas, 200m [;] IX-XI 1989, Hanson (MICR). 1 ♀, Est. Sirena, 0-100m, P.N. [;] Corcovado, Prov. Punt., [;] COSTA RICA, G. Fonseca [;] Jun 1991, [;] L-S-270500,508300 (INBC). 1 ♀, top label - COSTA RICA, Heredia [;] Est. Biol. La Selva, 50- [;] 150m, 10°26'N, 84°01'W [;] Apr 1996, INBio-OET; second label - 15 Marzo 1996 [;] bosque secundario [;] M/02/049 (INBC). 2 ♀♀, COSTA RICA [;] 11 mi. from Turrialba [;] “los Esperales”, C.A.T.I.E. [;] 5-II-1995 [;] P. Stanley (TAMU). 1 ♀, COSTA RICA: Puntarenas [;] RF Golfo Dulce el 200m [;] 24km W Piedras Blancas [;] P. Hanson ix.1992 (TAMU).

#### Comments.

This species is distinct by fore wing veins r and 3RSa nearly on the same line, bicolored body, narrow temple and smooth metasomal tergum 3.

#### Etymology.

Named for the country of Costa Rica.

**Figure 31.**
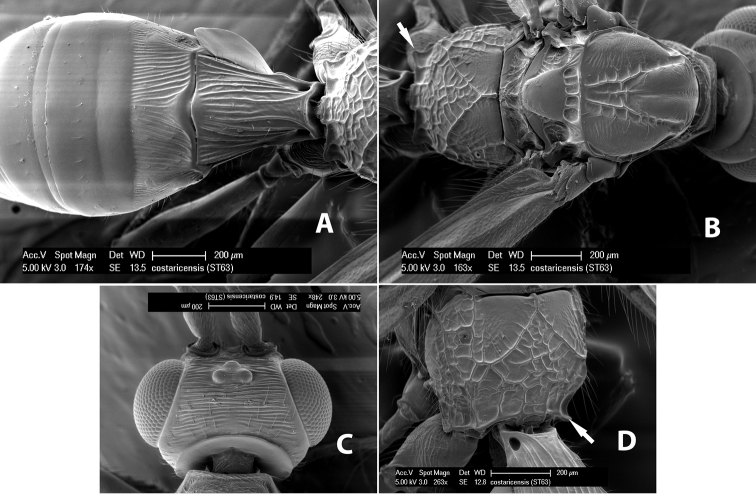
*Heterospilus costaricensis* Marsh, sp. n., paratype.

**Figure 32.**
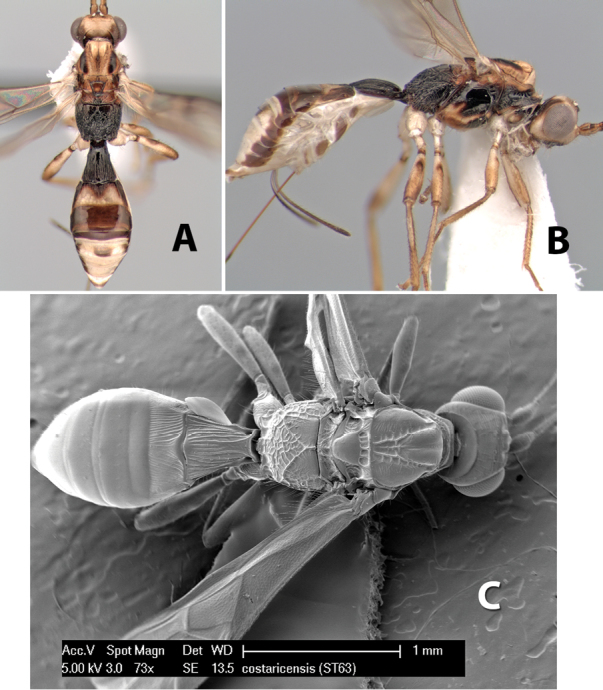
*Heterospilus costaricensis* Marsh, sp. n.: **A–B** holotype **C** paratype.

### Heterospilus cressoni

Marsh sp. n.

http://zoobank.org/B292CFE5-3FF8-4A0D-AD4A-F9590F5491C2

http://species-id.net/wiki/Heterospilus_cressoni

Figure 33

#### Female.

Body size: 3.5–4.0 mm. Color: head brown, face and eye orbits often lighter; scape and pedicel yellow, flagellum brown with apical 4–6 flagellomeres white; mesosoma brown, propodeum and mesopleuron dorsally dark brown; first metasomal tergum brown, second tergum brown medially and laterally with yellow longitudinal stripes laterally, third tergum yellow basally and medially, brown laterally, terga 4–6 yellow with anterior and posterior borders and laterally brown; wing vein brown, stigma brown with extreme base often yellow; legs with coxae and trochanters yellow, femora yellow on basal 1/4, light brown on apical 3/4, tibiae and tarsi brown. Head: vertex transversely striate, smooth near occipital carina; frons striate; temple smooth; face rugose-striate; temple in dorsal view bulging, about 1/2 eye width; malar space about 1/3 eye height; 26–31 flagellomeres. Mesosoma: mesoscutal lobes striate-granulate, middle lobe with median longitudinal raised line; notauli scrobiculate, meeting at prescutellar furrow in wide rectangular rugose area; mesopleuron smooth; precoxal sulcus smooth, about 1/2 width of mesopleuron; scutellum smooth; prescutellar furrow with 3–5 cross carinae; venter of mesopleuron smooth; propodeum nearly entirely rugose dorsally and laterally, without margined basal median areas or areola, small smooth basal median areas present. Wings: fore wing vein r 1/3 length vein 3RSa, vein 1cu-a distinctly beyond vein 1M; hind wing vein SC+R present, vein M+CU shorter than vein 1M. Metasoma: first tergum length equal to or slightly less than apical width, longitudinally costate-rugose medially, longitudinally costate laterally, granulate between costae; second tergum apical width less than 3 times median length, longitudinally costate, granulate between costae; anterior and posterior transverse groove distinct, anterior groove slightly sinuate; third tergum longitudinally costate between transverse grooves, smooth beyond posterior transverse groove; terga 4-6 smooth; ovipositor equal to or slightly longer than metasoma.

#### Holotype female.

Top label (white, partially printed and hand written) - Costa Rica: Guanacaste [;] Santa Rosa Natl. Park [;] 300m. ex. Malaise trap [;] Site # BH-12-C [;] Dates: 6-27.ix.1986 [;] I. D. Gauld & D. Janzen; second label (white printed) - [BH] Bosque Humedo [;] mature evergreen dry forest [;] [C] more or less fully [;] shaded as possible; third label (red, partially printed and hand written) - HOLOTYPE [;] Heterospilus [;] cressoni [;] P. Marsh. Deposited in ESUW.

#### Paratypes.

2 ♀♀, same data as holotype except site # of H-4-C and dates of 23.iii - 13.iv.1986 and 4-24.v.1986 (ESUW). 1 ♀, Sirena, Osa Pen. [;] VII.77 Cos. Rica [;] D. H. Janzen (AEIC).

#### Comments.

This species is distinctive by the transversely costate mesoscutal lobes.

#### Etymology.

This species is named for E. T. Cresson who described many braconids during the late 1800s.

**Figure 33.**
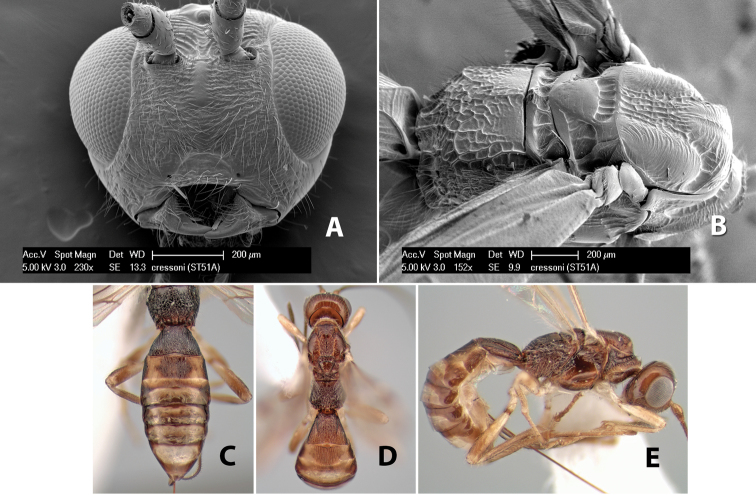
*Heterospilus cressoni* Marsh, sp. n.: **A–C** paratype **D–E** holotype.

### Heterospilus curtisi

Marsh sp. n.

http://zoobank.org/494A5B4F-BB28-4EC9-9620-6AF4B831B05E

http://species-id.net/wiki/Heterospilus_curtisi

Figure 34

#### Female.

Body size: 3.5 mm. Color: head dark brown; scape brown, flagellomeres brown with apical 5–8 flagellomeres white; mesosoma dark brown, metasoma dark brown; wing veins including stigma brown; legs light yellow or white, at least coxae and trochanters white. Head: vertex transversely striate; frons transversely striate; face rugose; temple in dorsal view broad but sloping behind eye, width equal to 1/2 eye width; malar space greater than 1/4 eye height; ocell-ocular distance 2–2.5 times diameter of lateral ocellus; 22–23 flagellomeres. Mesosoma: mesoscutal lobes granulate; notauli smooth posteriorly, weakly scrobiculate anteriorly, meeting at scutellum in triangular costate area; scutellum granulate; prescutellar furrow with 5 cross carinae; mesopleuron granulate; precoxal sulcus weakly scrobiculate, shorter than mesopleuron; venter granulate; propodeum with basal median areas margined, rugose-granulate, basal median carina absent, areola distinctly margined, areolar area rugose, lateral areas entirely rugose, propodeum with small but distinct tubercle just above hind coxa. Wings: fore wing vein r shorter than vein 3RSa, vein 1cu-a beyond vein 1M; hind wing vein SC+R present, vein M+CU shorter than vein 1M. Metasoma: first tergum longitudinally costate, length slightly greater than apical width; second tergum longitudinally costate; anterior transverse groove absent; posterior transverse groove absent; third tergum entirely smooth; terga 4-7 smooth; ovipositor equal to 1/2 length of metasoma.

#### Holotype female.

Top label (white, printed) - Costa Rica: Limon. ACLAC, 300m [;] Central Res. Biol. Hitoy Cerere [;] Est. Hitoy Cerere, Send. Bobocara [;] 8.x.1999, F. Umana, Red de Golpe [;] L.N. 184250-641800 #53498; second label (red, partially printed and hand written) - HOLOTYPE [;] Heterospilus [;] curtisi [;] P. Marsh. Deposited in ESUW.

#### Paratypes.

1 ♀, COSTA RICA: [;] Puntar. [;] Golfo Dulce, 3km [;] SW. Rincon, 10m [;] VI–VIII 1989, Hanson (ESUW). 1 ♀, Sirena, Osa Pen. [;] VII.77 Cos. Rica [;] D. H. Janzen (AEIC). 1 ♀, S.RosaPark,Guan, Guan. [;] C. Rica 4 Aug 77 [;] D.H. Janzen [;] Understory (AEIC).

#### Comments.

The white coxae and white apical flagellomeres are distinctive for this species.

#### Etymology.

Named for the British entomologist, J. Curtis.

**Figure 34.**
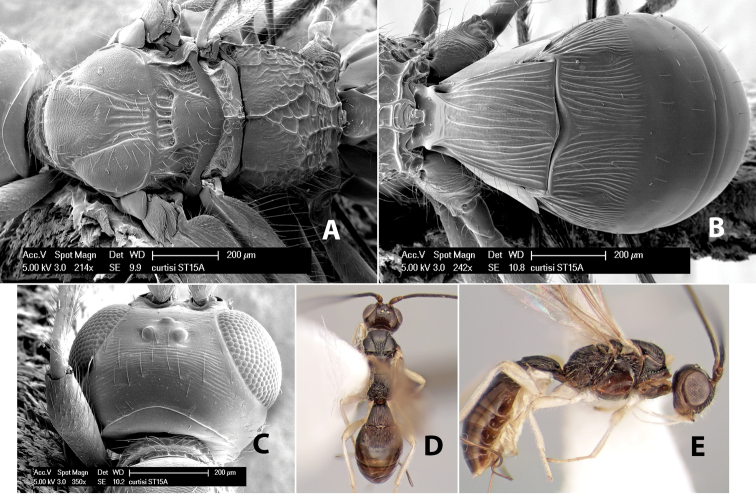
*Heterospilus curtisi* Marsh, sp. n.: **A–C** paratype **D–E** holotype.

### Heterospilus cushmani

Marsh sp. n.

http://zoobank.org/33CE172A-9FFE-4D97-A0E2-7A628F012015

http://species-id.net/wiki/Heterospilus_cushmani

Figure 35

#### Female.

Body size: 3.5 mm. Color: head dark brown, eye orbits and lower temple lighter brown; scape and pedicel yellow, flagellum yellow basally to brown apically; mesosoma dark brown; metasoma dark brown, apex of third tergum and following terga lighter brown; wing veins brown, stigma entirely brown; legs yellow. Head: vertex and frons transversely costate; face areolate or areolate-rugose; temple in dorsal view slightly less than half eye width; malar space about 1/3 eye height; ocell-ocular distance about twice diameter of lateral ocellus; 19+ flagellomeres (broken). Mesosoma: mesoscutal lobes granulate, short white setae along notauli; notauli scrobiculate, meeting before prescutellar furrow in triangular rugose area; mesopleuron smooth, weakly costate dorsally; precoxal sulcus weakly scrobiculate, about 3/4 length of mesopleuron; scutellum weakly granulate; prescutellar furrow with 5 cross carinae; venter of mesosoma smooth; propodeum with basal median areas distinct and margined, basal median areas granulate, basal median carina absent, areola meeting anterior margin of propodeum, areola margined only apically, areola and propodeum laterally rugose. Wings: fore wing vein r about 3/4 length of vein 3RSa, vein 1cu-a distinctly beyond vein 1M; hind wing vein SC+R present, vein M+CU shorter than vein 1M. Metasoma: first tergum length equal to apical width, longitudinally costate, raised basal median area slightly rugose; second tergum apical width less than 3 times length, longitudinally costate, granulate between costae; anterior transverse groove weak, posterior transverse groove very weak, only slightly indicated medially; third tergum weakly costate at extreme base, remainder smooth; terga 4-6 smooth; ovipositor longer than metasoma, as long as metasoma and half mesosoma.

#### Holotype female.

Top label (white, partially printed and hand written) - Costa Rica: Guanacaste [;] Santa Rosa Natl. Park [;] 300m. ex. Malaise trap [;] Site #: (blank) [;] Dates: 10–31.1987 (no month) [;] I. D. Gauld & D. Janzen. Second label (white, printed) - [BH] Bosque Humedo [;] mature evergreen dry forest [;] [O] in clearing, fully [;] isolate part of day. Third label (red, partially printed and hand written) - HOLOTYPE [;] Heterospilus [;] cushmani [;] P. Marsh. Deposited in ESUW.

#### Paratypes.

Known only from the holotype.

#### Comments.

This species is similar to *Heterospilus nigricoxus* but differs in having the stigma entirely brown (bicolored in *Heterospilus nigricoxus*), the legs entirely yellow (hind coxa and femur brown in *Heterospilus nigricoxus*), and granulate mesoscutal lobes without being rugose along notauli (as in *Heterospilus nigricoxus*).

#### Etymology.

This species is named after R. A. Cushman who studied Braconidae in the early 1900s.

**Figure 35.**
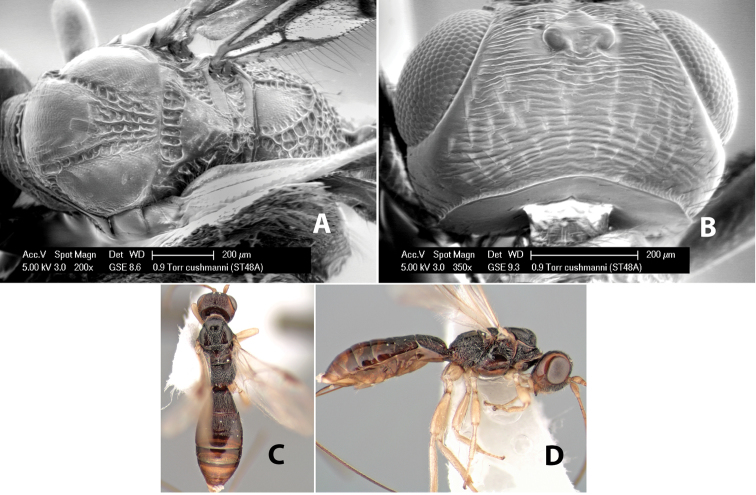
*Heterospilus cushmani* Marsh, sp. n., holotype.

### Heterospilus eberhardi

Marsh sp. n.

http://zoobank.org/E3FFD32A-FD63-49E9-ABBF-798255DD5305

http://species-id.net/wiki/Heterospilus_eberhardi

Figure 36

#### Female.

Body size: 2.5–4.0 mm. Color: head brown to dark brown; scape brown, without lateral longitudinal brown stripe, flagellum brown with apical 3–5 flagellomeres white; mesosoma dark brown; metasoma with terga 1–2 dark brown, terga 3–5 dark brown basally, yellow apically, terga 6–7 yellow; legs bicolored, coxae and trochanters yellow, joint of trochanters 2 and femora brown, femora yellow on basal 1/4, brown on apical 3/4, tibiae and tarsi brown; wing veins including stigma brown. Head: vertex weakly transversely striate; frons smooth; face smooth; temple in dorsal view narrow, slightly less than 1/2 eye width; malar space greater than 1/4 eye height; ocell-ocular distance slightly greater than 2.5 times diameter of lateral ocellus; 21–26 flagellomeres. Mesosoma: mesoscutal lobes smooth; notauli weakly and broadly scrobiculate, sometimes appearing smooth, with 2 longitudinal carinae converging toward prescutellar furrow; scutellum smooth; prescutellar furrow with 3 cross carinae; mesopleuron smooth; precoxal sulcus smooth, shorter than mesopleuron; venter smooth; propodeum with basal median areas distinctly margined, smooth, basal median carina present but short, areola usually distinctly margined, areolar area rugose, lateral areas entirely rugose. Wings: fore wing vein r shorter than vein 3RSa, vein 1cu-a beyond vein 1M; hind wing vein SC+R present, vein M+CU shorter than vein 1M. Metasoma: first tergum longitudinally costate, length greater than apical width; second tergum longitudinally costate; anterior transverse groove present, straight; posterior transverse groove present; third tergum longitudinal costate basally, smooth apically; terga 4-7 smooth; ovipositor equal to 3/4 length of metasoma.

#### Holotype female.

Top label (white, printed) - Costa Rica: San Jose [;] San Antonio de Escazu [;] 1300m, yellow pan [;] xii.1995, W. Eberhard; second label (red, partially printed and hand written) - HOLOTYPE [;] Heterospilus [;] eberhardi [;] P. Marsh. Deposited in ESUW.

#### Paratypes.

1 ♀, same data as holotype (ESUW). 1 ♀, Costa Rica: San Jose [;] San Antonio de Escazu [;] 1300m, vi.1997 (ESUW). 1 ♀, Costa Rica: San Jose [;] San Antonio de Escazu [;] 1300m, vi.1997 [;] W. Eberhard (ESUW). 1 ♀, Costa Rica: San Jose [;] San Antonio de Escazu [;] 1300m, v-vi.1995 [;] W. Eberhard & P. Hanson. 3 ♀♀, Costa Rica: Cartago [;] Braulio Carillo N.P. [;] 600m, 25.iii.1990 [;] J. S. Noyes, coll. (ESUW). 1 ♀, Costa Rica, Guanacaste [;] Tierras Morenas, Cerca de [;] Las Faldas del Volcan Tenorio [;] 1100m, 20.vii–20.viii.1996 [;] G. Rodriquez, Malaise trap [;] L.N. 289000-426500 #32630 (ESUW). 1 ♀, COSTA RICA: Puntarenas [;] San Vito, Estac. Biol. [;] Los Alturas 1500m [;] iv.1992 P. Hanson (TAMU). 1 ♀, COSTA RICA, Heredia [;] Sta. Barbara, Café [;] 1100m, 15.x.1989 [;] Col. Paul Hanson (MICR).

#### Comments.

The smooth mesoscutal lobes and two distinct longitudinal carinae before the prescutellar groove are distinctive for this species.

#### Etymology.

Named for William Eberhard who collected several specimens of the type series.

**Figure 36.**
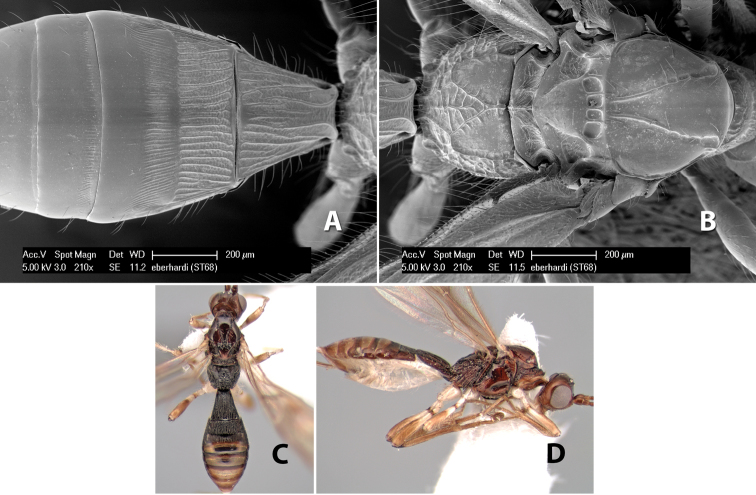
*Heterospilus eberhardi* Marsh, sp. n.: **A–B** paratype **C–D** holotype.

### Heterospilus emilius

Marsh sp. n.

http://zoobank.org/45BBE8CC-34AB-4088-82DA-275F7F196239

http://species-id.net/wiki/Heterospilus_emilius

Figure 37

#### Female.

Body size: 2.5 mm. Color: head brown; scape yellow, flagellum brown; mesosoma dark brown; metasoma brown, terga 2–3 yellow medially; legs yellow; wing veins including stigma brown. Head: vertex weakly transversely striate; frons weakly striate, nearly smooth; face smooth; temple in dorsal view narrow, sloping behind eye, width less than 1/2 eye width; malar space greater than 1/4 eye height; ocell-ocular distance about 2.5 times diameter of lateral ocellus; 17 flagellomeres. Mesosoma: mesoscutal lobes granulate; notauli scrobiculate, meeting posteriorly in triangular rugose area; scutellum granulate; prescutellar furrow with 3 cross carinae; mesopleuron smooth; precoxal sulcus smooth, shorter than mesopleuron; venter smooth; propodeum with basal median areas granulate, distinctly margined, basal median carina present, short, areola not distinctly margined, areolar area rugose, lateral areas entirely rugose. Wings: fore wing vein r shorter than vein 3RSa, vein 1cu-a beyond vein 1M; hind wing vein SC+R present, vein M+CU shorter than vein 1M. Metasoma: first tergum longitudinally costate, apical width equal to length; second tergum longitudinally striate; anterior transverse groove absent; posterior transverse groove absent; third tergum entirely smooth; terga 4-7 smooth; ovipositor length equal to length of metasomal terga 1+2.

#### Holotype female.

Top label (white, partially printed and hand written) - Costa Rica: Guanacaste [;] Santa Rosa Natl. Park [;] 300m, ex. Malaise trap [;] Site #: (blank) [;] Dates: 18.i–8.ii.1986 [;] I.D. Gauld & D. Janzen; second label (white, printed) - [SE] Bosque San Emilio [;] 50yr old deciduous forest [;] [C] more or less fully [;] shaded as possible; third label (red, partially printed and hand written) - HOLOTYPE [;] Heterospilus [;] emilius [;] P. Marsh. Deposited in ESUW.

#### Paratypes.

Known only from the holotype.

#### Comments.

The smooth mesopleuron, smooth face and short antennae are distinctive for this species.

#### Etymology.

Named for the San Emilio Forest in Santa Rosa National Park where the type was collected.

**Figure 37.**
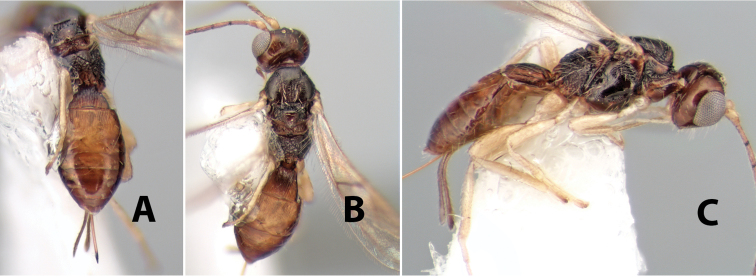
*Heterospilus emilius* Marsh, sp. n., holotype.

### Heterospilus enderleini

Marsh sp. n.

http://zoobank.org/BEC5C336-2736-4E59-B509-3A4318157412

http://species-id.net/wiki/Heterospilus_enderleini

Figure 38

#### Female.

Body size: 2.5 mm. Color: head with vertex and frons brown, face and temple honey yellow; scape yellow without lateral brown stripe, flagellum yellow basally to brown apically; mesosoma brown; metasoma brown to dark brown; wing veins including stigma brown; legs yellow. Head: vertex transversely costate; frons transversely costate; face rugose; temple in dorsal view narrow, width less than 1/2 eye width; malar space greater than 1/4 eye height; ocell-ocular distance about twice diameter of lateral ocellus; 16 flagellomeres. Mesosoma: mesoscutal lobes granulate; notauli scrobiculate, meeting at scutellum in triangular rugose area; scutellum granulate; prescutellar furrow with 5 cross carinae; mesopleuron granulate; precoxal sulcus weakly scrobiculate, shorter than mesopleuron; venter granulate; propodeum with basal median areas margined, granulate, basal median carina absent, areola weakly margined, areolar area areolate-rugose, lateral areas entirely rugose, apical-lateral corners of propodeum produced into blunt tubercle. Wings: fore wing vein r as long as vein 3RSa, vein 1cu-a beyond vein 1M; hind wing vein SC+R absent, vein M+CU shorter than vein 1M. Metasoma: first tergum longitudinally costate, apical width equal to length; second tergum longitudinally costate; anterior transverse groove present, straight; posterior transverse groove absent; third tergum costate basally, smooth apically; terga 4–7 smooth; ovipositor equal to length of metasomal tergum 1.

#### Holotype female.

Top label (white, printed) - COSTA RICA: Puntarenas [;] Reserva Forestal Golfo Dulce [;] 3km southwest of Rincon [;] 10m, July 1991, P. Hanson [;] primary forest, Malaise trap; second label (red, partially printed and hand written) - HOLOTYPE [;] Heterospilus [;] enderleini [;] P. Marsh. Deposited in ESUW.

#### Paratypes.

Known only from the holotype.

#### Comments.

The tubercles on apical-lateral corners of the propodeum, the rugose face and the absence of hind wing vein SC+R are distinctive for this species.

#### Etymology.

Named for G. Enderlein who described numerous South American braconids in the early 1900s.

**Figure 38.**
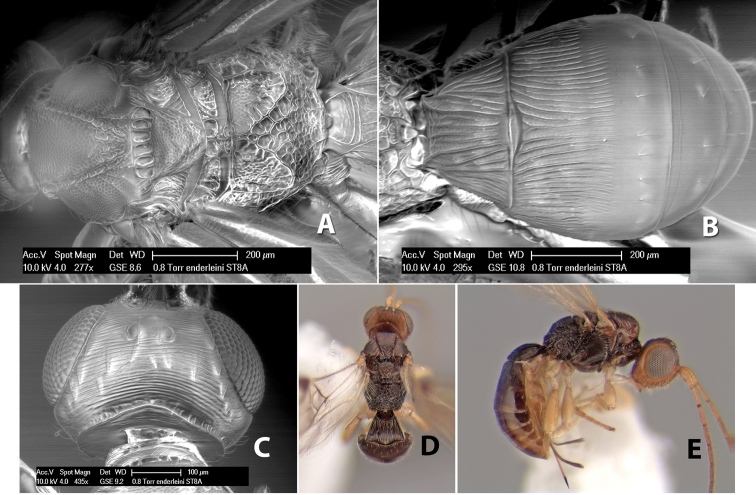
*Heterospilus enderleini* Marsh, sp. n., holotype.

### Heterospilus escazuensis

Marsh sp. n.

http://zoobank.org/A5730566-93DA-4608-B645-C831A3359146

http://species-id.net/wiki/Heterospilus_escazuensis

Figure 39

#### Female.

Body size: 2.5 mm. Color: head honey yellow; scape yellow without lateral brown stripe, flagellum yellow basally to brown apically; mesosoma brown, propodeum somewhat darker; metasomal terga 1, 2 and base of 3 brown, apex of 3 and remainder of terga yellow; wing veins light brown, stigma yellow; legs yellow. Head: vertex transversely costate, costae somewhat circular around ocelli; frons transversely costate; face smooth; temple in dorsal view narrow, sloping behind eye, less than 1/2 eye width; malar space greater than 1/4 eye height; ocell-ocular distance about 2.5 times diameter of lateral ocellus; at least 18 flagellomeres (broken). Mesosoma: mesoscutal lobes granulate; notauli scrobiculate, meeting at scutellum in triangular rugose area; scutellum granulate; prescutellar furrow with 3 cross carinae; mesopleuron granulate above precoxal sulcus, rugose-costate dorsally; precoxal sulcus scrobiculate, shorter than mesopleuron; venter granulate; propodeum with basal median areas margined, granulate-rugose, basal median carina distinct but short, areola not distinctly margined, areolar area rugose, lateral areas entirely rugose. Wings: fore wing vein r shorter than vein 3RSa, vein 1cu-a beyond vein 1M; hind wing vein SC+R present, vein M+CU shorter than vein 1M. Metasoma: first tergum longitudinally costate, apical width slightly less than length; second tergum longitudinally costate; anterior transverse groove present, straight; posterior transverse groove present; third tergum costate basally, smooth apically; terga 4-7 smooth; ovipositor as long as metasomal terga 1–2 combined.

#### Holotype female.

Top label (white, printed) - Costa Rica: San Jose [;] San Antonio de Escazu [;] 1300m, ix.1998 [;] W. Eberhard; second label (red, partially printed and hand written) - HOLOTYPE [;] Heterospilus [;] escazuensis [;] P. Marsh. Deposited in ESUW.

#### Paratypes.

1 ♀, C. Rica: Escazú [;] May 21, 1987 [;] H.&M. Townes (AEIC).

#### Comments.

The granulate mesopleuron, the costae being somewhat circular around the ocelli and the brown or honey yellow body are distinctive for this species.

#### Etymology.

Named after the type locality of San Antonio de Escazu in San Jose Province.

**Figure 39.**
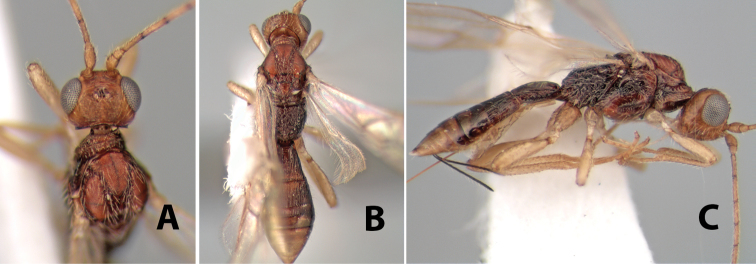
*Heterospilus escazuensis* Marsh, sp. n., holotype.

### Heterospilus fahringeri

Marsh sp. n.

http://zoobank.org/4655DB46-7474-430E-BC17-AC3E7CD9AA1E

http://species-id.net/wiki/Heterospilus_fahringeri

Figure 40

#### Female.

Body size: 2.0-2.5 mm. Color: body entirely dark brown; scape yellow without lateral brown stripe, flagellum brown; wing veins including stigma brown; legs entirely yellow. Head: vertex transversely striate; frons transversely striate; face granulate; temple in dorsal view narrow, broad but sloping behind eye, width equal to 1/2 eye width; malar space about equal to 1/4 eye height; ocell-ocular distance about 2.5 times diameter of lateral ocellus; 17 flagellomeres. Mesosoma: mesoscutal lobes granulate; notauli scrobiculate, meeting at scutellum in small rugose area; scutellum granulate; prescutellar furrow with 3 cross carinae; mesopleuron granulate; precoxal sulcus scrobiculate, shorter than mesopleuron; venter granulate; propodeum with basal median areas margined, granulate, basal median carina absent, areola not distinctly margined, areolar area rugose, lateral areas rugose posteriorly, granulate anteriorly. Wings: fore wing vein r shorter than vein 3RSa, vein 1cu-a beyond vein 1M; hind wing vein SC+R present, vein M+CU slightly longer than vein 1M. Metasoma: first tergum longitudinally costate, apical width equal to length; second tergum longitudinally costate; anterior transverse groove weak but present; posterior transverse groove absent; third tergum smooth entirely; terga 4–7 smooth; ovipositor 1/2 length of metasoma.

#### Holotype female.

Top label (white, printed) - Costa Rica: Puntarenas [;] San Vito, Estac. Biol. [;] Las Alturas, 1500m [;] xi.1991, Paul Hanson; second label (red, partially printed and hand written) - HOLOTYPE [;] Heterospilus [;] fahringeri [;] P. Marsh. Deposited in ESUW.

#### Paratypes.

1 ♀, S.RosaPark,Guan, Guan. [;] C. Rica 20.Dec.76 [;] D. H. Janzen [;] Riparian (AEIC).

#### Comments.

The granulate mesopleuron and face, the smooth third metasomal tergum and the absent posterior transverse groove on the metasoma are distinctive for this species.

#### Etymology.

Named for the German entomologist, J. Fahringer, who described many braconids in the early 1900s.

**Figure 40.**
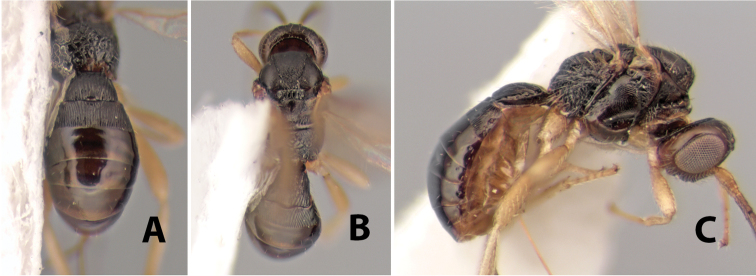
*Heterospilus fahringeri* Marsh, sp. n., holotype.

### Heterospilus fischeri

Marsh sp. n.

http://zoobank.org/B33FE58A-14A4-4F55-A8E0-4A233397FB04

http://species-id.net/wiki/Heterospilus_fischeri

Figure 41

#### Female.

Body size: 2.0–2.5 mm. Color: head with vertex and frons light brown, face and eye orbit honey yellow; scape yellow without lateral brown stripe, flagellum brown; mesosoma brown to dark brown; metasomal tergum 1 dark brown, tergum 2 light brown or yellow medially, brown laterally, terga 3–7 light brown to honey yellow; wing veins including stigma brown; legs yellow. Head: vertex weakly transversely striate, often nearly smooth; frons smooth; face smooth, sometimes with weak striations below antennae; temple in dorsal view narrow, less than 1/2 eye width; malar space greater than 1/4 eye height; ocell-ocular distance nearly 2.5 times diameter of lateral ocellus; 20–21 flagellomeres. Mesosoma: mesoscutal lobes granulate; notauli scrobiculate, meeting at scutellum in triangular or nearly rectangular rugose area; scutellum granulate; prescutellar furrow with 3–5 cross carinae; mesopleuron granulate; precoxal sulcus scrobiculate, shorter than mesopleuron; venter granulate; propodeum with basal median areas margined, granulate, basal median carina present, areola not distinctly margined, areolar area rugose, lateral areas rugose posteriorly, granulate anteriorly, apical lateral corners of propodeum produced into small tubercles. Wings: fore wing vein r shorter than vein 3RSa, vein 1cu-a beyond vein 1M; hind wing vein SC+R present, vein M+CU shorter than vein 1M. Metasoma: first tergum longitudinally costate, apical width equal to length; second tergum longitudinally costate; anterior transverse groove present, straight; posterior transverse groove present; third tergum costate at base, smooth apically; terga 4–7 smooth; ovipositor equal to length of metasomal terga 1–2 combined, rarely equal to 1/2 length of metasoma.

#### Holotype female.

Top label (white, partially printed and hand written) - Costa Rica: Guanacaste [;] Santa Rosa Natl. Park [;] 300m, ex. Malaise trap [;] Site #: BH-12-C [;] Dates: 8.ii–2.iii.1986 [;] I.D. Gauld & D. Janzen; second label (white, printed) - [BH] Bosque Humedo [;] mature evergreen dry forest [;] [C] more or less fully [;] shaded as possible; third label (red, partially printed and hand written) - HOLOTYPE [;] Heterospilus [;] fischeri [;] P. Marsh. Deposited in ESUW.

#### Paratypes.

3 ♀♀, same data as holotype except dates of 8–29.xi.1986, 18.i–8.ii.1986 and second label [BH] Bosque Humedo [;] mature evergreen dry forest [;] [O] in clearing, fully [;] isolated part of day (ESUW).

#### Comments.

The tubercles at the apical lateral corners of the propodeum are distinctive for this species.

#### Etymology.

Named for the Austrian braconidologist, Max Fischer.

**Figure 41.**
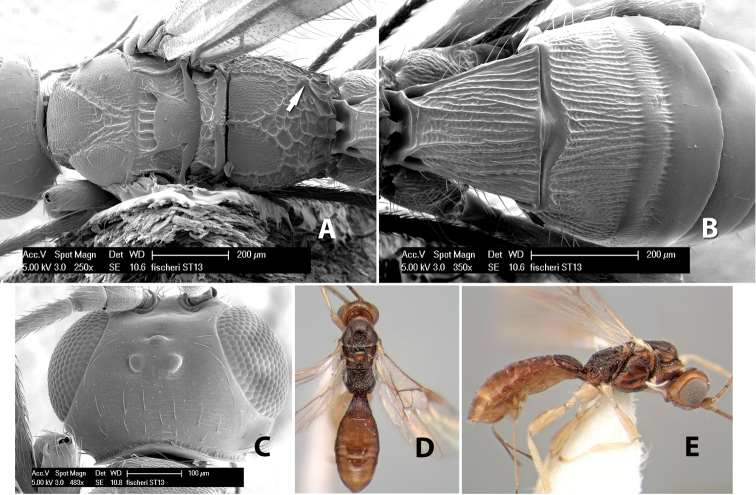
*Heterospilus fischeri* Marsh, sp. n.: **A–C** paratype **D–E** holotype.

### Heterospilus flavidus

Marsh sp. n.

http://zoobank.org/DEB2B7AD-BA1A-4D32-B918-0CE97C5A7EB6

http://species-id.net/wiki/Heterospilus_flavidus

Figure 42

#### Female.

Body size: 3.0 mm. Color: head yellow; scape yellow without lateral longitudinal brown stripe, flagellum yellow at base to brown at apex; mesoscutum and propleuron yellow, propodeum and mesopleuron darker honey yellow; metasomal tergum 1 honey yellow medially, brown laterally, terga 2 and 3 yellow medially, brown laterally, tergum 4 at base yellow medially, brown laterally and yellow apically, terga 5–7 yellow; wing veins brown, stigma brown, yellow at apex and along anterior margin; legs yellow. Head: vertex transversely costate; frons transversely costate; face rugose; temple in dorsal view narrow, width less than 1/2 eye width; malar space greater than 1/4 eye height; ocell-ocular distance about 2.5 times diameter of lateral ocellus; 26 flagellomeres. Mesosoma: mesoscutal lobes granulate and dull, lateral lobes covered with short yellow hair; notauli scrobiculate, meeting at scutellum in triangular rugose area; scutellum weakly granulate; prescutellar furrow with 3 cross carinae; mesopleuron granulate; precoxal sulcus smooth, shorter than mesopleuron; venter granulate; propodeum with basal median areas margined, granulate, basal median carina absent, areola not margined, areolar area rugose, lateral areas entirely rugose. Wings: fore wing vein r slightly shorter than vein 3RSa, vein 1cu-a beyond vein 1M; hind wing vein SC+R present, vein M+CU shorter than vein 1M. Metasoma: first tergum longitudinally costate, apical width less than length; second tergum longitudinally costate, width about 4 times length; anterior transverse groove present, sinuate; posterior transverse groove present; third tergum costate at base, smooth at apex; tergum 4 weakly costate at base, smooth apically, remainder of terga smooth; ovipositor as long as metasoma.

#### Holotype female.

Top label (white, printed) - Costa Rica: Guanacaste, Santa [;] Rosa Nat’l. Park, Bosque San [;] Emilio, trap #7 in clearing, 300m [;] II/8-III/2/1986, I Gauld; second label (white, printed) - [SE] Bosque San Emilio [;] 50yr old deciduous forest [;] [O] in clearing, fully [;] isolated part of day; third label (red, partially printed and hand written) - HOLOTYPE [;] Heterospilus [;] flavidus [;] P. Marsh. Deposited in ESUW.

#### Paratypes.

Known only from the holotype.

#### Comments.

The yellow color of this species is distinctive.

#### Etymology.

The specific name is from the Latin *flavidus* meaning yellowish in reference to the body color.

**Figure 42.**
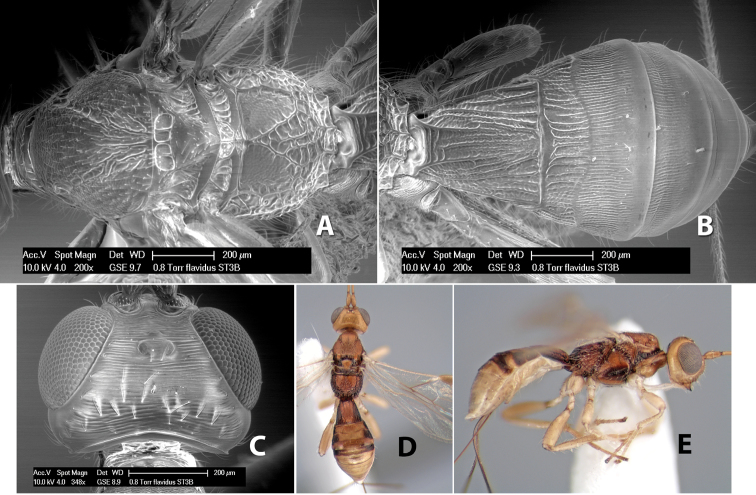
*Heterospilus flavidus* Marsh, sp. n., holotype.

### Heterospilus foersteri

Marsh sp. n.

http://zoobank.org/BE633161-596B-4AC2-B5AD-ECE3F4A59CF9

http://species-id.net/wiki/Heterospilus_foersteri

Figure 43

#### Female.

Body size: 3.0–4.0 mm. Color: body entirely dark brown, metasomal terga 4–7 usually lighter brown, face and eye orbits occasionally yellow; scape yellow, without lateral longitudinal brown stripe, flagellum brown; legs yellow, femora light brown on apical half; wing veins brown, stigma brown. Head: vertex transversely costate; frons transversely costate; face striate; temple in dorsal view broad, slightly bulging, width greater than 1/2 eye width; malar space greater than 1/4 eye height; ocell-ocular distance nearly 3 times diameter of lateral ocellus; 23–29 flagellomeres. Mesosoma: mesoscutal lobes granulate; notauli scrobiculate, meeting at scutellum in triangular costate area; scutellum smooth; prescutellar furrow with one cross carinae, rarely with weak carinae on each side; mesopleuron smooth; precoxal sulcus smooth or weakly scrobiculate, shorter than mesopleuron; venter smooth; propodeum with basal median areas distinctly margined, granulate, rugose along apical carina, basal median carina distinct, areola not distinctly margined, areolar area rugose, lateral areas entirely rugose. Wings: fore wing vein r shorter than vein 3RSa, vein 1cu-a beyond vein 1M; hind wing vein SC+R present, vein M+CU shorter than vein 1M. Metasoma: first tergum length slightly greater than apical width, longitudinally costate-granulate; second tergum costate-granulate, width less than 3 times length; anterior transverse groove present, straight; posterior transverse groove present; third tergum costate at base, smooth apically; terga 4–7 smooth; ovipositor longer than metasoma.

#### Holotype female.

Top label (white, printed) - Costa Rica: Puntarenas [;] Golfo Dulce. 24km w. [;] Piedras Blancas, 200m [;] xii.1991. Paul Hanson; second label (red, partially printed and hand written) - HOLOTYPE [;] Heterospilus [;] foersteri [;] P. Marsh. Deposited in ESUW.

#### Paratypes.

1 ♀, same data as holotype except date of ii.1993 (ESUW). 1 ♀, top label - Costa Rica: Guanacaste [;] Santa Rosa Natl. Park [;] 300m. ex. Malaise trap [;] Site #: (blank) [;] Dates: 2–23.iii.1986 [;] I.D. Gauld & D. Janzen; second label - [SE] Bosque San Emilio [;] 50yr old deciduous forest [;] [O] in clearing, fully [;] isolated part of day (ESUW). 1 ♀, top label - Costa Rica: Guanacaste [;] Santa Rosa Natl. Park [;] 300m. ex. Malaise trap [;] Site #: (blank) [;] Dates: 18.i–8.ii.1986 [;] I.D. Gauld & D. Janzen; second label - [SE] Bosque San Emilio [;] 50yr old deciduous forest [;] [C] more or less fully [;] shaded as possible (ESUW).

#### Comments.

The dark brown body, single cross carina in prescutellar groove, and the granulate basal median areas of the propodeum are characteristic for this species.

#### Etymology.

Named for A. Foerster who presented the first subfamily classification of the Braconidae.

**Figure 43.**
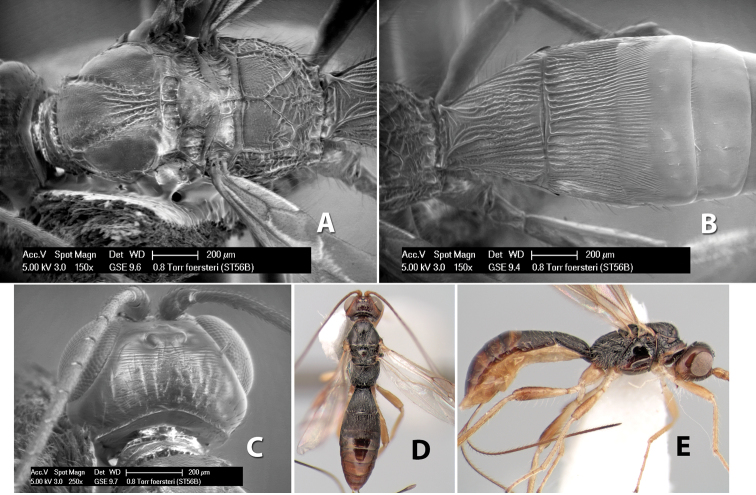
*Heterospilus foersteri* Marsh, sp. n.: **A–D** paratype **E** holotype.

### Heterospilus fonsecai

Marsh sp. n.

http://zoobank.org/FE53F57B-78DF-4D30-8F50-F8806105C5A2

http://species-id.net/wiki/Heterospilus_fonsecai

Figure 44

#### Female.

Body size: 4.0 mm. Color: head dark brown, face honey yellow; scape honey yellow, flagellum brown; mesosoma dark brown; metasoma dark brown, terga 5–7 lighter brown; wing veins brown, stigma yellow; legs yellow. Head: vertex weakly striate or entirely smooth, at least smooth near occipital carina; frons striate; face striate; temple smooth; temple in dorsal view less than 1/2 eye width, slightly bulging; malar space about 1/4 eye height; ocell-ocular distance twice diameter of lateral ocellus; 26–28 flagellomeres. Mesosoma: mesoscutal lobes smooth; notauli scrobiculate, meeting before prescutellar furrow in triangular longitudinally costate area; scutellum smooth; prescutellar furrow with 3 cross carinae; mesopleuron smooth; precoxal sulcus smooth, about 1/2 length of mesopleuron; venter smooth; propodeum with basal median areas distinctly margined, basal median areas smooth, basal median carina short but distinct, areola weakly margined, areolar area areolate-rugose, lateral areas areolate-rugose with small smooth area anteriorly. Wings: fore wing vein r about 1/2 length of vein 3RSa, vein 1cu-a beyond vein 1M by about its own length; hind wing vein SC+R present, vein M+CU slightly shorter than vein 1M. Metasoma: first tergum length about equal to apical width, longitudinally costate, granulate between costae; second tergum apical width less than 3 times median length, longitudinally costate, granulate between costae; anterior and posterior transverse grooves distinct; third tergum longitudinally costate at basal half, smooth on apical half; terga 4-7 smooth except costate at extreme base; ovipositor longer than metasoma.

#### Holotype female.

Top label (white, printed) - Costa Rica: Puntarenas [;] Est. Sirena (Parte Alta) [;] Send. Rio Claro, 1–100m [;] ix-xii.1991, G. Fonseca [;] L.S. 270500-508300 [;] #7453, Malaise trap; second label (red, partially printed and hand written) - HOLOTYPE [;] Heterospilus [;] fonsecai [;] P. Marsh. Deposited in ESUW.

#### Paratypes.

1 ♀, Costa Rica - Heredia Prov. [;] La Selva Biological Station [;] 10°26'N, 84°01'W 100m [;] Canopy fogging 32 [;] 3.xi.1994 [;] Project ALAS(FVK32) (ESUW). 1 ♀, top label -COSTA RICA, Heredia [;] Est. Biol. La Selva, 50- [;] 150m. 10°26'N, 81°01'W [;] Feb 1994, INBio-OET; second label - 1 Febrero 1994 [;] Bosque secundario [;] M/02/333 (ESUW).

#### Comments.

This species is distinct by its smooth mesoscutum and yellow stigma.

#### Etymology.

This species is named after the collector of the holotype, G. Fonseca.

**Figure 44.**
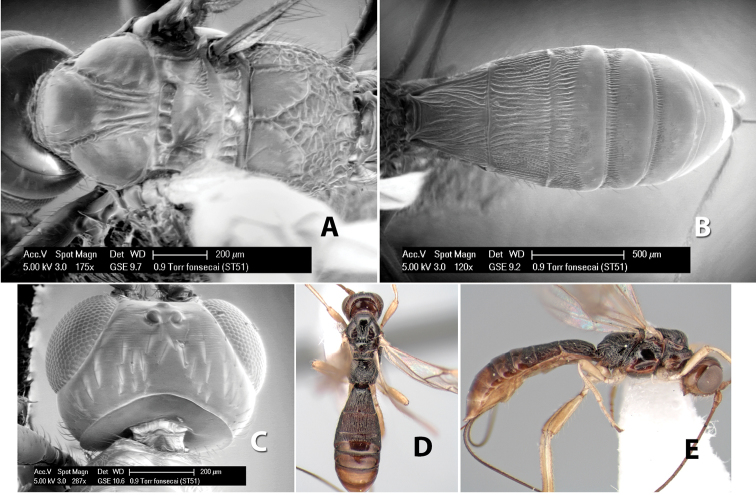
*Heterospilus fonsecai* Marsh, sp. n., holotype.

### Heterospilus fournieri

Marsh sp. n.

http://zoobank.org/4C74987C-863A-4C69-BA84-D63500CCE456

http://species-id.net/wiki/Heterospilus_fournieri

Figure 45

#### Female.

Body size: 2.0–2.5 mm. Color: body entirely honey yellow or yellow, propodeum sometimes slightly darker; scape yellow; flagellum yellow basally to brown apically; legs yellow; wing veins brown, stigma yellow. Head: vertex weakly striate; frons weakly striate; face smooth; temple in dorsal view narrow, sloping behind eye, width less than 1/2 eye width; malar space greater than 1/4 eye height; ocell-ocular distance greater than 2.5 times diameter of lateral ocellus; 11–17 flagellomeres. Mesosoma: mesoscutal lobes granulate; notauli scrobiculate, meeting at scutellum in triangular rugose area; scutellum granulate; prescutellar furrow with one distinct median cross carina, rarely with weak carinae laterally; mesopleuron smooth; precoxal sulcus smooth, shorter than mesopleuron; venter smooth; propodeum with basal median areas margined, granulate, basal median carina absent, areola not distinctly margined, areolar area rugose, lateral areas entirely rugose. Wings: fore wing vein r shorter than vein 3RSa, vein 1cu-a beyond vein 1M; hind wing vein SC+R present, vein M+CU nearly as long as vein 1M. Metasoma: first tergum longitudinally costate, apical width about equal to length; second tergum longitudinally costate; anterior transverse groove weak, straight; posterior transverse groove weak; third tergum smooth; terga 4-7 smooth; ovipositor half as long as metasoma.

#### Holotype female.

Top label (white, printed) - COSTA RICA: [;] San Jose [;] Ciudad Colon, 800m [;] xii 1989 - i 1990 [;] Luis Fournier; second label (red, partially printed and hand written) - HOLOTYPE [;] Heterospilus [;] fournieri [;] P. Marsh. Deposited in ESUW.

#### Paratypes.

1 ♀, top label, Costa Rica: Guanacaste [;] Santa Rosa National Pk. [;] 300m, Malaise, Ian Gauld [;] 31.i–21.ii.1987; second label, Open regenerating [;] woodland less than [;] 10 yrs. old, sun; third label, H-1-O [;] 31.i–21.ii.87 (ESUW). 1 ♀, S.RosaPark,Guan, Guan. [;] C. Rica 8 Mar. 77 [;] D. H. Janzen [;] Dry Hill (AEIC).

#### Comments.

The yellow or honey yellow body, smooth mesopleuron and hind wing vein SC+R being present are distinctive for this species.

#### Etymology.

Named for the collector of the holotype, Luis Fournier.

**Figure 45.**
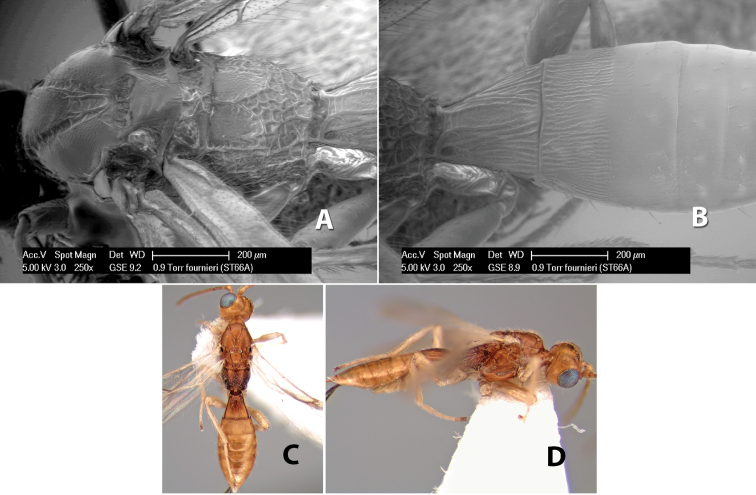
*Heterospilus fournieri* Marsh, sp. n., holotype.

### Heterospilus gahani

Marsh sp. n.

http://zoobank.org/A5B2EB1C-62E8-458B-8A43-7F9E54C0016B

http://species-id.net/wiki/Heterospilus_gahani

Figure 46

#### Female.

Body size: 4.5 mm. Color: head honey yellow; scape yellow without lateral longitudinal brown stripe, flagellum brown with apical 3–5 flagellomeres white; mesoscutum and propleuron honey yellow, propodeum, mesopleuron and venter dark brown; metasomal tergum 1 dark brown, tergum 2 dark brown with lateral converging honey yellow stripes, tergum 3 honey yellow medially, dark brown laterally, terga 4–7 honey yellow; wing veins brown, stigma yellow with central brown spot; fore and mid legs with coxae and trochanters 1 white, trochanters 2 yellow basally, brown apically, femora yellow basally and light brown apically, tibiae yellow, tarsi brown; hind coxa brown, trochanter 1 yellow, trochanter 2 yellow basally, brown apically, femur yellow on basal 1/4, brown on apical 3/4, tibia yellow medially, brown at extreme base and on apical 1/2, tarsus brown. Head: vertex transversely costate; frons transversely costate; face granulate; temple in dorsal view broad, not sloping behind eye, width equal to 1/2 eye width; malar space greater than 1/4 eye height; ocell-ocular distance slightly greater than 2.5 times diameter of lateral ocellus; 27 flagellomeres. Mesosoma: mesoscutal lobes granulate, costate along notauli; notauli scrobiculate, meeting at scutellum in rectangular rugose area; scutellum smooth; prescutellar furrow with 3–5 cross carinae; mesopleuron granular; precoxal sulcus smooth, shorter than mesopleuron; venter granulate; propodeum with basal median areas margined, rugose-granulate, basal median carina present and with attached short carinae at right angles, areola margined, areolar area rugose, lateral areas rugose posteriorly, granulate anteriorly. Wings: fore wing vein r shorter than vein 3RSa, vein 1cu-a beyond vein 1M; hind wing vein SC+R present, vein M+CU shorter than vein 1M. Metasoma: first tergum costate laterally, rugose medially, apical width 1/2 length; second tergum costate; anterior transverse groove present, sinuate; posterior transverse groove present; third tergum smooth, weakly striate antero-laterally; terga 4–7 smooth; ovipositor about 1/2 length of metasoma.

#### Holotype female.

Top label (white, printed) - Costa Rica: San Jose [;] Cerro de la Muerte [;] 2km W Empalme [;] 2300m, June 1995 [;] P. Hanson, Malaise; second label (red, partially printed and hand written) - HOLOTYPE [;] Heterospilus [;] gahani [;] P. Marsh. Deposited in ESUW.

#### Paratypes.

Known only from the holotype.

#### Comments.

This species is distinctive by the long and narrow first metasomal tergum and the costate mesoscutal lobes.

#### Etymology.

Named for the American entomologist, A. B. Gahan, who described many New World Braconidae in the early 1900s.

**Figure 46.**
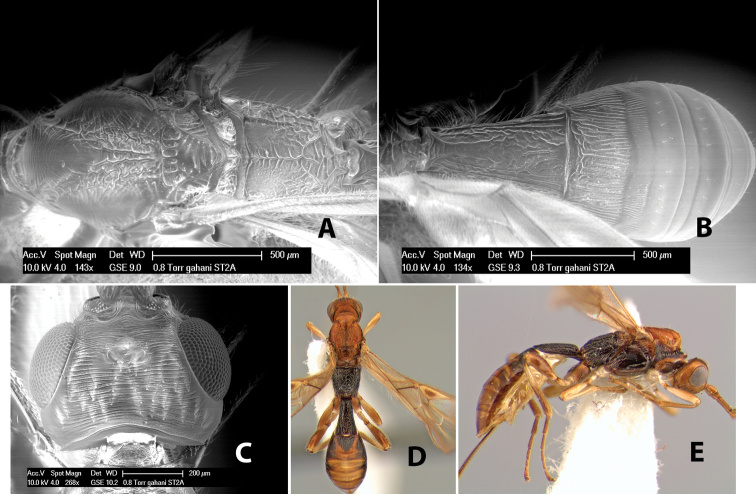
*Heterospilus gahani* Marsh, sp. n., holotype.

### Heterospilus garifuna

Marsh sp. n.

http://zoobank.org/E89463AD-6EAF-4A74-9F20-E3D2A07FDC62

http://species-id.net/wiki/Heterospilus_garifuna

Figure 47

#### Female.

Body size: 2.5 mm. Color: head with vertex and frons brown, face honey yellow; scape yellow without lateral longitudinal brown stripe, flagellum yellow at base to brown at apex; mesosoma brown; metasomal terga brown to honey yellow, tergum 2 lighter than other terga; wing veins including stigma brown; legs yellow. Head: vertex weakly striate medially, smooth near eyes; frons weakly striate near antennal bases; face smooth; temple in dorsal view narrow, sloping behind eye, width less than 1/2 eye width; malar space greater than 1/4 eye height; ocell-ocular distance about 1.5 times diameter of lateral ocellus; 20 flagellomeres. Mesosoma: mesoscutal lobes granulate; notauli scrobiculate, meeting at scutellum in triangular rugose area; scutellum granulate; prescutellar furrow with 1 cross carina; mesopleuron granulate; precoxal sulcus scrobiculate, shorter than mesopleuron; venter granulate; propodeum with basal median areas margined, granulate, basal median carina absent or extremely short, areola not distinctly margined, areolar area rugose, lateral areas entirely rugose. Wings: fore wing vein r equal to length of vein 3RSa, vein 1cu-a beyond vein 1M; hind wing vein SC+R absent, vein M+CU equal in length to vein 1M. Metasoma: first tergum longitudinally costate, apical width equal to length; second tergum longitudinally costate; anterior transverse groove present, straight; posterior transverse groove present; third tergum weakly costate at base, smooth apically; terga 4-7 smooth; ovipositor equal to length of metasomal terga 1-2 combined.

#### Holotype female.

Top label (white, partially printed and hand written) - Costa Rica: Guanacaste [;] Santa Rosa Natl. Park [;] 300m, ex. Malaise trap [;] Site #: (blank) [;] Dates: 13.iv–4.v.1986 [;] I.D. Gauld & D. Janzen; second label (white, printed) - [SE] Bosque San Emilio [;] 50yr old deciduous forest [;] [C] more or less fully [;] shaded as possible; third label (red, partially printed and hand written) - HOLOTYPE [;] Heterospilus [;] garifuna [;] P. Marsh. Deposited in ESUW.

#### Paratypes.

1 ♀, S.RosaPark, Guan. [;] C. Rica 25 Oct 77 [;] D.H. Janzen [;] Riparian (AEIC).

#### Comments.

This species is distinguished by the granulate mesopleuron, smooth face, prescutellar furrow with one cross carinae and the absent hind wing vein SC+R.

#### Etymology.

Named for the Garifuna, an indigenous people of Belize and Honduras.

**Figure 47.**
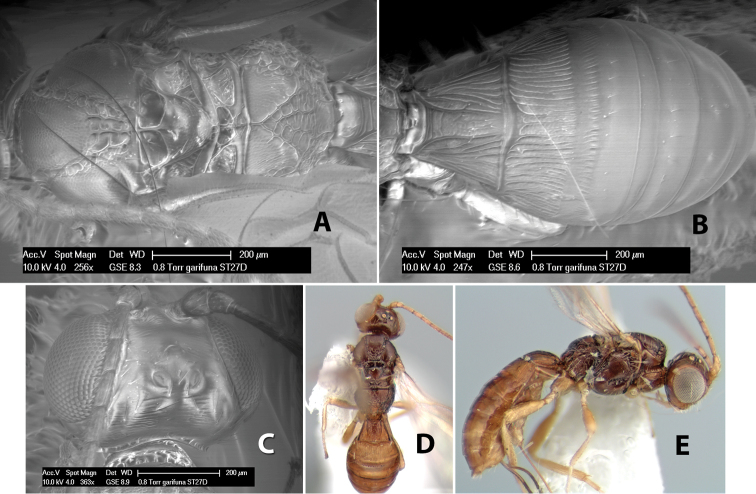
*Heterospilus garifuna* Marsh, sp. n., holotype.

### Heterospilus gauldi

Marsh sp. n.

http://zoobank.org/098257E3-539C-41F9-BBD3-FCA6F962E2FC

http://species-id.net/wiki/Heterospilus_gauldi

Figure 48

#### Female.

Body size: 3.0–4.0 mm. Color: Head with vertex and frons brown, face often and eye orbits always yellow; scape and flagellum brown; mesosoma brown or dark brown, propleuron, mesopleuron and notauli often lighter brown; metasomal tergum 1 brown, tergum 2 honey yellow, often brown medially and laterally, tergum 3 yellow medially, brown laterally, terga 4–7 honey yellow; legs yellow; wing veins brown, stigma bicolored brown with apex, base and anterior margin often yellow. Head: vertex weakly transversely striate or rarely smooth; frons weakly transversely striate; face striate; temple in dorsal view broad, not sloping behind eye, width 1/2 eye width; malar space greater than 1/4 eye height; ocell-ocular distance 2.0–2.5 times diameter of lateral ocellus; 22–28 flagellomeres. Mesosoma: mesoscutal lobes smooth; notauli scrobiculate, meeting at scutellum in wide rectangular rugose area; scutellum smooth; prescutellar furrow with 3 cross carinae; mesopleuron smooth; precoxal sulcus smooth, shorter than mesopleuron; venter smooth; propodeum with basal median areas margined, partially smooth and rugose, basal median carina absent, areola not margined, areolar area rugose or areolate-rugose, lateral areas entirely rugose. Wings: fore wing vein r nearly equal to vein 3RSa, vein 1cu-a distinctly beyond vein 1M; hind wing vein SC+R present, vein M+CU shorter than vein 1M. Metasoma: first tergum laterally longitudinally costate, medially with raised rugose area bordered by distinct converging carinae; second tergum longitudinally costate; anterior transverse groove present, straight; posterior transverse groove weakly present or absent; third tergum costate basally, smooth apically; terga 4-7 smooth; ovipositor about 1/2 length of metasoma.

#### Holotype female.

Top label (white, partially printed and hand written) - Costa Rica: Guanacaste [;] Santa Rosa Natl. Park [;] 300m, ex. Malaise trap [;] Site #: SE-8-C [;] Dates: 24.v–14.vi.1986 [;] I.D. Gauld & D. Janzen; second label (white, printed) - [SE] Bosque San Emilio [;] 50yr old deciduous forest [;] [C] more or less fully [;] shaded as possible; third label (red, partially printed and hand written) - HOLOTYPE [;] Heterospilus [;] gauldi [;] P. Marsh. Deposited in ESUW.

#### Paratypes.

4 ♀♀, same data as holotype, with dates of 31.i–21.ii.1987, 4–24.v.1986, 2–23.iii.1986 (ESUW). 5 ♀♀, same data as holotype, with dates of 13.iv–4.v.1986, 23.iii–13.iv.1986, 2–23.iii.1986, and second label - [BH] Bosque Humedo [;] mature evergreen dry forest [;] [C] more or less fully [;] shaded as possible (ESUW). 9 ♀♀, same data as holotype, with dates of 4–24.v.1986, 13.iv–4.v.1986, 24.v–14.vi.1986, and second label - [H] open regenerating [;] woodland <10 years old [;] [O] in clearing, fully [;] shaded part of day (ESUW). 6 ♀♀, same data as holotype, with dates of 12.iv–4.v.1986, 6–27.xi.1986, 24.v–14.v.1986, 13.ix–4.x.1986, and second label - [SE] Bosque San Emilio [;] 50yr old deciduous Forest [;] [O] in clearing, fully [;] isolated part of day (ESUW). 1 ♀, same data as holotype, with date of 23.iii–13.iv.1986, and second label - [BH] Bosque Humedo [;] mature evergreen dry forest [;] [O] in clearing, fully [;] isolated part of day (ESUW). 5 ♀♀, same data as holotype, with dates of 13.iv–4.v.1986, and second label - [H] open regenerating [;] woodland <10 years old [;] [C] more or less fully [;] isolated as possible (ESUW). 2 ♀♀, top label - Costa Rica: Guanacaste [;] Santa Rosa National Pk. [;] 300m, Malaise, Ian Gauld [;] 24.v–14.vi.1986; second label - Bosque San Emilio [;] 50yr old deciduous [;] forest. Sun (ESUW). 1 ♀, Costa Rica: Guanacaste, GCA [;] Sector Santa Rosa, tropical [;] dry forest, open field nr. [;] road to Playa Naranjo, [;] malaise trap, 8-18 June 1995 [;] Dadelahi, Price, Zitani (ESUW). 1 ♀, Costa Rica: Puntarenas [;] ACO, Golfito, RF Golfo Dulce [;] Est. Agujas, 250–300m [;] 2-22.x.1999, J. Azofeifa [;] L.S. 276750-526550 #53490 [;] Amarilla (ESUW). 1 ♀, Costa Rica: Puntarenas [;] Pen. Osa, 5km. N. Pto. [;] Jimenez, 10m, iii–iv.1991, P. Hanson, Malaise (ESUW). 25 ♀♀, S.RosaPark, Guan. [;] C. Rica, various dates from May 76 to May 78 [;] D.H. Janzen [;] Dry Hill and Riparian (AEIC).

#### Comments.

This species is distinguished by the short and broad metasomal tergum 1, smooth mesoscutal lobes and the rectangular area where the notauli meet before the scutellum.

#### Etymology.

Named for my friend and colleague, the late Ian Gauld, in recognition of his many years of support for the study of the biodiversity of the Hymenoptera of Costa Rica.

**Figure 48.**
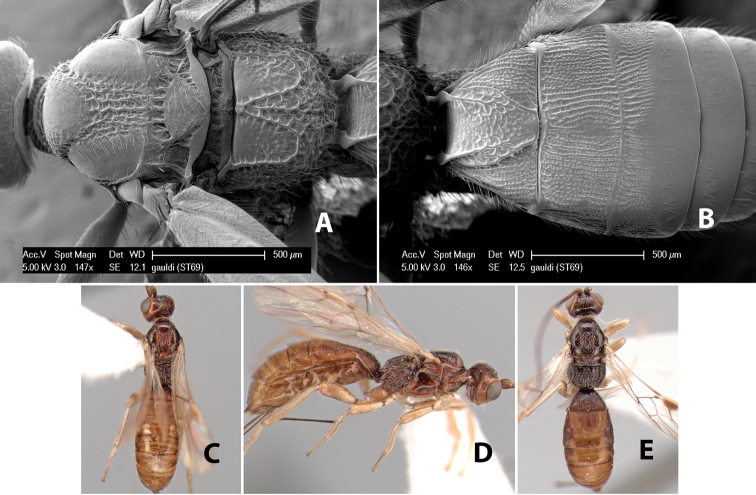
*Heterospilus gauldi* Marsh, sp. n.: **A, B, E** paratype **C, D** holotype.

### Heterospilus golfodulcensis

Marsh sp. n.

http://zoobank.org/20B982B1-2FAE-4538-8C82-E9477343E8C4

http://species-id.net/wiki/Heterospilus_golfodulcensis

Figure 49

#### Female.

Body size: 3.0 mm. Color: head, mesosoma and metasoma dark brown or black; scape yellow without lateral brown stripe, flagellum brown; wing veins including stigma brown, tegula dark brown; legs yellow. Head: vertex transversely striate; frons weakly striate; face smooth; temple in dorsal view narrow, sloping behind eye, width less than 1/2 eye width; malar space greater than 1/4 eye height; ocell-ocular distance slightly more than twice diameter of lateral ocellus; 22 flagellomeres. Mesosoma: mesoscutal lobes granulate; notauli scrobiculate, meeting at scutellum in triangular costate area; scutellum granulate; prescutellar furrow with 3 cross carinae; mesopleuron granulate; precoxal sulcus scrobiculate, shorter than mesopleuron; venter granulate; propodeum with basal median areas margined, granulate, basal median carina absent, areola weakly margined, areolar area areolate-rugose, lateral areas entirely rugose. Wings: fore wing vein r shorter than vein 3RSa, vein 1cu-a beyond vein 1M; hind wing vein SC+R present, vein M+CU about as long as vein 1M. Metasoma: first tergum longitudinally costate, apical width equal to length; second tergum longitudinally costate, width nearly 4 times median length; anterior transverse groove present, straight; posterior transverse groove present; third tergum costate basally, smooth apically; terga 4–7 smooth; ovipositor 3/4 length of metasoma.

#### Holotype female.

Top label (white, printed) - COSTA RICA: Puntarenas [;] R.F. Golfo Dulce [;] 24km W. Piedras Blancas, [;] 200m [;] Feb. 1992, Paul Hanson; second label (red, partially printed and hand written) - HOLOTYPE [;] Heterospilus [;] golfodulcensis [;] P. Marsh. Deposited in ESUW.

#### Paratypes.

Known only from the holotype.

#### Comments.

The dark brown or black metasomal terga and tegula are distinctive for this species.

#### Etymology.

Named for the locality where the type was collected, the Golfo Dulce Forest Reserve.

**Figure 49.**
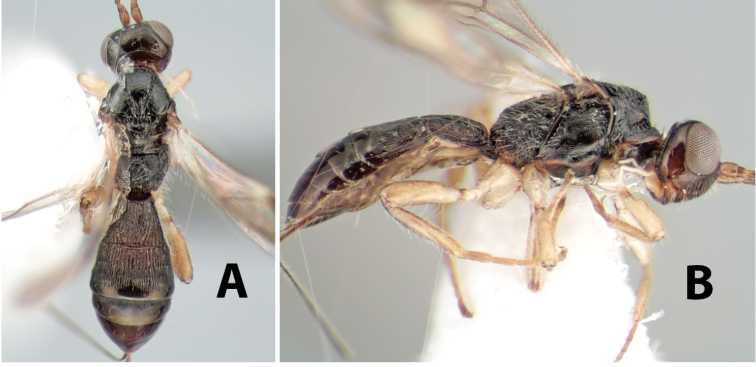
*Heterospilus golfodulcensis* Marsh, sp. n., holotype.

### Heterospilus gouleti

Marsh sp. n.

http://zoobank.org/8D890235-71ED-460D-88E4-F3B27F4E4080

http://species-id.net/wiki/Heterospilus_gouleti

Figures 50 , 51

#### Female.

Body size: 3.5-4.0 mm. Color: head brown to dark brown; scape honey yellow with lateral longitudinal brown stripe; flagellum brown with apical 10-12 flagellomeres white; mesosoma dark brown, mesoscutal lobes usually lighter brown; metasomal terga 1, 2 and base of 3 dark brown or black, apex or tergum 3, and all of terga 4-5 lighter brown, base of tergum 6 brown, apex yellow, tergum 7 yellow; wing veins including stigma brown; legs yellow, hind femur yellow on basal 1/4, brown of apical 3/4, hind tarsus often brown. Head: vertex weakly striate, often smooth near eyes; frons weakly striate or smooth; face smooth; temple in dorsal view narrow, sloping behind eye, width less than 1/2 eye wide; malar space equal to or slightly greater than 1/4 eye height; ocell-ocular distance twice diameter of lateral ocellus; 29-31 flagellomeres. Mesosoma: mesoscutal lobes granulate; notauli scrobiculate, meeting at scutellum in triangular costate area; scutellum granulate; prescutellar furrow with 1 distinct median carina and 2 weaker carinae laterally; mesopleuron granulate; precoxal sulcus smooth, shorter than mesopleuron; venter granulate; propodeum with basal median areas margined, granulate, basal median carina absent, areola not distinctly margined, areolar area rugose, lateral areas rugose posteriorly, smooth or granulate anteriorly, distinct tubercle present laterally on each side of base of metasomal tergum 1 above hind coxa. Wings: fore wing vein r shorter than vein 3RSa, vein 1cu-a slightly beyond vein 1M; hind wing vein SC+R present, vein M+CU shorter than vein 1M. Metasoma: first tergum longitudinally costate or porcate, apical width less than length; second tergum longitudinally costate or porcate; anterior transverse groove present, straight; posterior transverse groove present; third tergum costate at base, smooth apically; terga 4-7 smooth; ovipositor as long as metasoma.

#### Holotype female.

Top label (white, printed) - Costa Rica, Heredia [;] Puerto Viejo, 100m [;] OTS-La Selva [;] III-1991 P. Hanson; second label (red, partially printed and hand written) - HOLOTYPE [;] Heterospilus [;] gouleti [;] P. Marsh. Deposited in ESUW.

#### Paratypes.

1 ♀, top label - COSTA RICA: Heredia [;] Pr: La Selva Biol. Sta. [;] 3km S Pto. Viejo [;] 10°26'N, 84°01'W; second label - 27.VII.1992 [;] H.A. Hespenheide (ESUW). 1 ♀, Costa Rica-Heredia Prov. [;] Las Selva Biological Station [;] 10°26'N, 84°01'W 100m [;] Malaise trap 13, #315 [;] 3.i.1994 [;] Project ALAS (M.13.315) (ESUW). 1 ♀, Costa Rica, Puntarenas [;] R.F.Golfo Dulce, 5km. W. [;] Piedras Blancas, 100m [;] VI-VII-1993, P. Hanson (ESUW). 1 ♀, COSTA RICA, Alajuela [;] Finca La Selva [;] NE Dos Rios, 400m [;] 27/III.88, Col. Hanson (ESUW). 1 ♀, Costa Rica: Limon [;] Sector Cocori, 100m [;] 30km N Cariari, i.1995 [;] E. Rojas, Malaise #4526 [;] L.N. 268000-567500 (ESUW). 1 ♀, Costa Rica: Limon, ACLAC [;] Central, R.B. Hitoy Cerere [;] Send. Espavel, 560m [;] 19.v–19.vi.1998, E. Rojas [;] L.S. 400702-570120 #52200 [;] Malaise Trap (ESUW). 1 ♀, COSTA RICA, Guanac. [;] Estac. Pitilia, 9Km S [;] Santa Cecilia, 700m [;] IX/1988, I.Gauld (ESUW). 1 ♀, Rancho Quemado, 200m, [;] Peninsula de Osa, Prov. Punt. [;] COSTA RICA. Set 1991. F. [;] Quesada. L-S-292500-511000 (INBC). 1 ♀, COSTA RICA. Prov. Puntarenas, [;] Golfito, P.N. Corcovado, Send a [;] Sirena, 100m, 05 MAY 2001. J. Azofeifa Libre. L_S_276500_514200 (INBC).

#### Comments.

This species is distinguished by the tubercle on the propodeum just above the hind coxa, the strongly costate metasomal terga 1-2 and the brown flagellum with apical flagellomeres 10-12 being white.

#### Etymology.

Named for my friend and colleague Henri Goulet.

**Figure 50.**
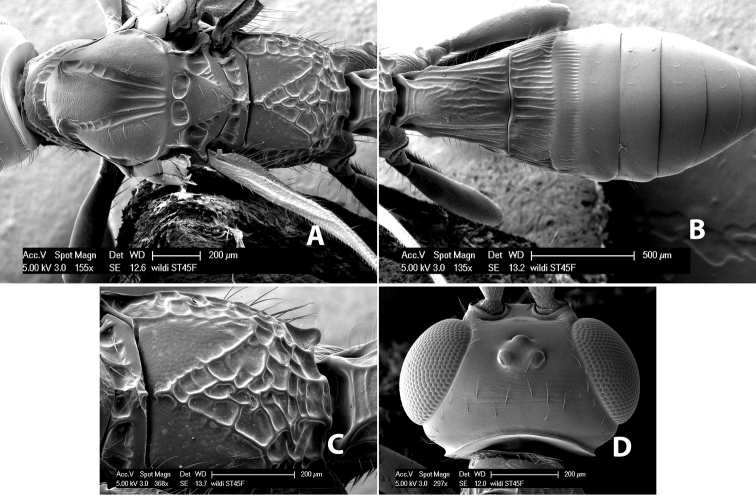
*Heterospilus gouleti* Marsh, sp. n., paratype.

**Figure 51.**
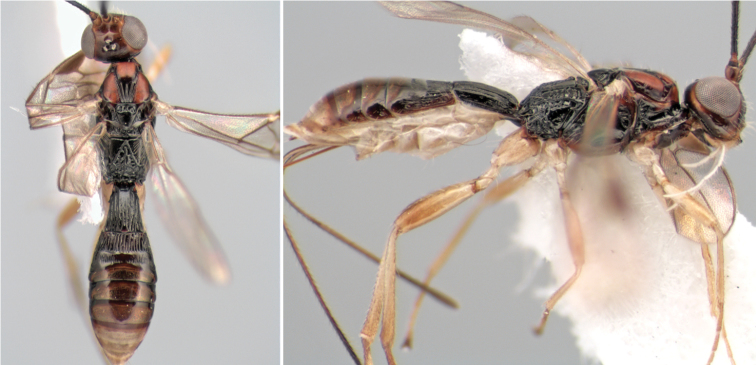
*Heterospilus gouleti* Marsh, sp. n., holotype.

### Heterospilus granulatus

Marsh sp. n.

http://zoobank.org/9ECBE257-445D-4B35-AA74-FC74016DDB3B

http://species-id.net/wiki/Heterospilus_granulatus

Figure 52

#### Female.

Body size: 4.0 mm. Color: head with vertex, frons and face brown, malar space, temple and eye orbits yellow, scape light brown or honey yellow, without lateral longitudinal brown stripe, flagellum brown; mesosoma dark brown or black, mesoscutal lobes, lower portion of mesopleuron and venter light brown or honey yellow; metasomal tergum 1 dark brown or black, tergum 2 dark brown with honey yellow stripes laterally, remaining terga dark brown basally, lighter brown apically; legs yellow, femora, especially hind femur, brown; wing veins brown, stigma brown with yellow at apex. Head: vertex transversely costate; frons transversely costate; face granulate; temple in dorsal view narrow, sloping behind eye, width less than 1/2 eye width; malar space greater than 1/4 eye height; ocell-ocular distance about 1.5 times diameter of lateral ocellus; 30–35 flagellomeres. Mesosoma: mesoscutal lobes granulate; notauli scrobiculate, meeting at scutellum in rugose triangular area; scutellum granulate; prescutellar furrow with 1 distinct median cross carina and 2 weak carinae; mesopleuron granulate; precoxal sulcus smooth, shorter than mesopleuron; venter granulate; propodeum with basal median areas distinctly margined, granulate, basal median carina present and long, areola usually not margined, areolar area rugose, lateral areas rugose posteriorly, granulate anteriorly. Wings: fore wing vein r shorter than vein 3RSa, vein 1cu-a beyond vein 1M; hind wing vein SC+R present, vein M+CU shorter than vein 1M. Metasoma: first tergum costate-granulate, apical width less than length; second tergum costate-granulate; anterior transverse groove present, sinuate; posterior transverse groove weakly indicated; third tergum entirely granulate; terga 4-7 granulate; ovipositor 1/2 length of metasoma.

#### Holotype female.

Top label (white, printed) - COSTA RICA: Puntarenas [;] R.F. Golfo Dulce, [;] 24km W. Piedras Blancas, [;] 200m, [;] Feb. 1992, Paul Hanson; second label (red, partially printed and hand written) - HOLOTYPE [;] Heterospilus [;] granulatus [;] P. Marsh. Deposited in ESUW.

#### Paratypes.

1 ♀, Costa Rica: Limon ACLAC [;] Central, R.B. Hitoy Cerere [;] Est. H. Cerere, 100-140m [;] Send. Toma de Agua, Malaise [;] 17.xi–17.xii.1999, F. Umana [;] L.N. 184600-643400 #54940 (ESUW). 1 ♀, Cerro Tortuguero, 0-120m, P.N. [;] Tortuguero, Prov. Limón, COSTA [;] RICA. Feb 1993. R. Delgado. [;] L-N-285000,588000 (INBC). 1 ♀, top label - COSTA RICA, Heredia [;] Est. Biol. La Selva, 50- [;] 150m, 10°26'N, 84°01'W [;] Sep 1993, INBio-OET; second label - 01 Setiembre 1993 [;] M/03/194 [;] Bosque primario (INBC).

#### Comments.

This species is distinguished by the granulate metasomal terga and the yellow stripes laterally on metasomal tergum 2.

#### Etymology.

The specific name is in reference to the distinctly granulate metasomal terga.

**Figure 52.**
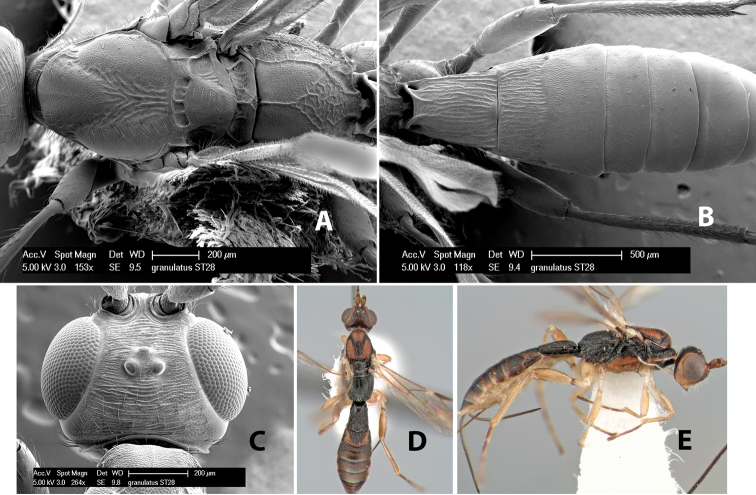
*Heterospilus granulatus* Marsh, sp. n.: **A–C** paratype **D–E** holotype.

### Heterospilus grisselli

Marsh sp. n.

http://zoobank.org/FD94D0C8-049C-42BC-B22A-267C112C15D2

http://species-id.net/wiki/Heterospilus_grisselli

Figure 53

#### Female.

Body size: 2.0–3.0 mm. Color: head with vertex and frons brown, face and eye orbits yellow; scape yellow without lateral brown stripe, flagellum yellow basally to brown apically; mesosoma and metasoma brown to dark brown; wing veins including stigma brown; legs yellow. Head: vertex transversely striate; frons transversely striate; face rugose; temple in dorsal view narrow, width less than eye width; malar space greater than 1/4 eye height; ocell-ocular distance 2-2.5 times diameter of lateral ocellus; 16-18 flagellomeres. Mesosoma: mesoscutal lobes granulate; notauli scrobiculate, meeting at scutellum in triangular costate area; scutellum granulate; prescutellar furrow with 3-5 cross carinae; mesopleuron granulate; precoxal sulcus weakly scrobiculate, shorter than mesopleuron and often with weak striae extending from sulcus to posterior margin of mesopleuron; venter granulate; propodeum with basal median areas margined, granulate, basal median carina absent, areola not distinctly margined, areolar area areolate-rugose, lateral areas entirely rugose. Wings: fore wing vein r shorter than vein 3RSa, vein 1cu-a beyond vein 1M; hind wing vein SC+R absent, vein M+CU shorter than vein 1M. Metasoma: first tergum longitudinally costate, apical width equal to length; second tergum longitudinally costate; anterior transverse groove present, straight; posterior transverse groove usually weakly present, occasionally absent; third tergum costate at extreme base, remainder smooth; terga 4-7 smooth; ovipositor as long as metasomal tergum 1.

#### Holotype female.

Top label (white, printed) - Costa Rica: Guanacaste, ACT [;] Bagaces, P. N. Palo Verde [;] Sec. P. Verde, 0-50m [;] Extremo E. Campo Aterrizaje [;] Malaise trap, #53260 [;] 17.viii–13.ix.1999, I. Jimenez [;] L.N. 260952-385020; second label (red, partially printed and hand written) - HOLOTYPE [;] Heterospilus [;] grisselli [;] P. Marsh. Deposited in ESUW.

#### Paratypes.

6 ♀♀, top label - Costa Rica: Guanacaste [;] Santa Rosa Natl. Park [;] 300m, ex. Malaise trap [;] Site #: BH-12-C and blank [;] Dates: 8.ii–2.iii.1986, 18.i–8.ii.1986 and 16.xi–7.xii.1985; second label - [BH] Bosque Humedo [;] mature evergreen dry forest [;] [C] more or less fully [;] shaded as possible (ESUW). 4 ♀♀, top label - Costa Rica: Guanacaste [;] Santa Rosa Natl. Park [;] 300m, ex. Malaise trap [;] Site #: 11, 9 and BH-9-O [;] Dates: 23.iii–13.iv.1986, 28.xii.85–18.i.1986, 8.ii–2.iii.1986 and 8–29.x.1986; second label - [BH] Bosque Humedo [;] mature evergreen dry forest [;] [O] in clearing, fully [;] isolated part of day (ESUW). 1 ♀, top label - Costa Rica: Guanacaste [;] Santa Rosa Natl. Park [;] 300m, ex. Malaise trap [;] Site #: blank [;] Dates: 10–31.i.1987; second label - [H] open regenerating [;] woodland <10 years old [;] [O] in clearing, fully [;] isolated part of day (ESUW). 2 ♀♀, top label - Costa Rica: Guanacaste [;] Santa Rosa Natl. Park [;] 300m, ex. Malaise trap [;] Site #: SE-7-O and blank [;] Dates: 8.ii–2.iii.1986 and 20.xii.86–10.i.1987; second label - [SE] Bosque San Emilio [;] 50yr old deciduous forest [;] [O] in clearing, fully [;] isolated part of day (ESUW). 2 ♀♀, top label - Costa Rica: Guanacaste [;] Santa Rosa Natl. Park [;] 300m, ex. Malaise trap [;] Site #: blank [;] Dates: 8–29.xi.1986; second label - [SE] Bosque San Emilio [;] 50yr old deciduous forest [;] [C] more or less fully [;] shaded as possible (ESUW). 1 ♀, Costa Rica: Puntarenas [;] Res. Forestal Golfo Dulce [;] 3km. SW Rincon, 10m [;] iv.1993, P. Hanson [;] Malaise, primary forest (ESUW). 2 ♀♀, Costa Rica: Puntarenas [;] R.F. Golfo Dulce, [;] 3km SW. Rincon, 10m, [;] vi.1991, Paul Hanson (ESUW). 1 ♀, Costa Rica: Puntarenas [;] R.F. Golfo Dulce [;] 3km. SW Rincon, 10m, [;] iii.1993 Paul Hanson coll. [;] Malaise, primary forest (ESUW). 2 ♀♀, S.RosaPark, Guan. [;] C. Rica 27 Oct 77 and 30 Aug 77 [;] Riparian (AEIC).

#### Comments.

The granulate mesopleuron, rugose face and short ovipositor are distinctive for this species.

#### Etymology.

Named for my friend and long time colleague at the Systematic Entomology Lab., Eric Grissell.

**Figure 53.**
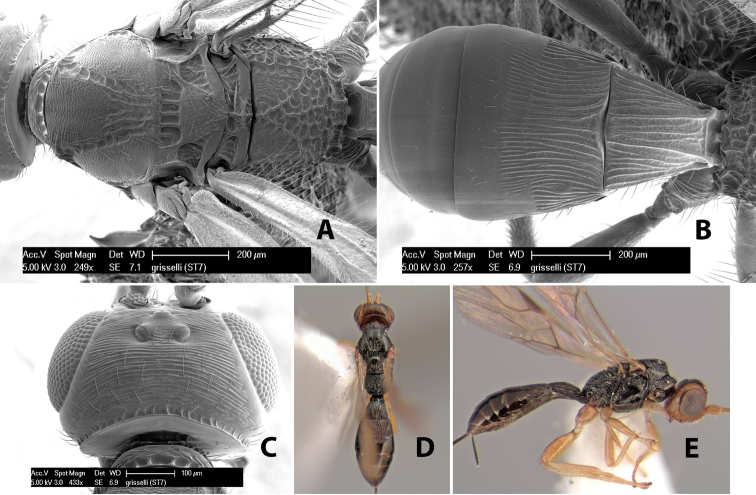
*Heterospilus grisselli* Marsh, sp. n.: **A–C** paratype **D–E** holotype.

### Heterospilus guanacastensis

Marsh sp. n.

http://zoobank.org/54795EEF-9923-4301-B4B7-59949F482072

http://species-id.net/wiki/Heterospilus_guanacastensis

Figure 54

#### Female.

Body size: 2.5–3.5 mm. Color: head with vertex and frons brown, eye orbits and face lighter brown to yellow; scape yellow, basal 2 flagellomeres yellow, next 8–10 flagellomeres bicolored brown with yellow at apex and base, remainder of flagellomeres brown except apical 3–5 white; mesosoma and metasoma dark brown; legs bicolored yellow with apical 3/4 of middle and hind femora brown; wing veins and stigma brown, wing membrane dusky along vein r. Head: vertex transversely costate; frons transversely costate; face striate at least laterally, often smooth medially; temple in dorsal view narrow, usually less than eye width, occasionally equal; malar space greater than 1/4 eye height; ocell-ocular distance about 2.5 times diameter of lateral ocellus; 15–22 flagellomeres. Mesosoma: mesoscutal lobes granulate; notauli scrobiculate, meeting at prescutellar furrow in triangular costate-rugose area; scutellum smooth; prescutellar furrow with one median distinct cross carina, occasionally weak cross carinae on each side; mesopleuron smooth; precoxal sulcus weakly scrobiculate, extending posteriorly to margin of mesopleuron by distinct carinae; venter smooth; propodeum with basal median areas smooth, distinctly margined, basal median carina absent, areola distinctly margined, areolar area rugose, lateral areas entirely rugose. Wings: fore wing vein r shorter than vein 3RSa, vein 1cu-a beyond vein 1M; hind wing vein SC+R present, vein M+CU nearly equal to vein 1M. Metasoma: first tergum longitudinally costate, length greater than apical width; second tergum longitudinally costate; anterior transverse groove present, straight; posterior transverse groove absent; third tergum costate basally, smooth apically; terga 4-7 smooth; ovipositor usually longer than metasoma, occasionally only slightly longer and appearing equal in length.

#### Holotype female.

Top label (white, partially printed and hand written) - Costa Rica: Guanacaste [;] Santa Rosa Natl. Park [;] 300m. ex. Malaise trap [;] Site #: SE-7-0 [;] Dates: 4–24.v.1986 [;] I.D. Gauld & D. Janzen; second label (white, printed) - [SE] Bosque San Emilio [;] 50yr old deciduous forest [;] [O] in clearing, fully [;] isolated part of day; third label (red, partially printed and written) - HOLOTYPE [;] Heterospilus [;] guanacastensis [;] P. Marsh. Deposited in ESUW.

#### Paratypes.

1 ♀, same data as holotype except: Site #: 13-IX; Dates: 4.x.1986 (ESUW). 8 ♀♀, same data as holotype except: Site #: SE-8-C and SE-6-C; Dates: 18.i–8.ii.1986, 14.iii.1986 and 24.v–14.vi.1986; second label - [SE] Bosque San Emilio [;] 50yr old deciduous forest [;] [C] more or less fully [;] shaded as possible (ESUW). 1 ♀, top label - Costa Rica: Guanacaste, Santa [;] Rosa Nat’l Park, Bosque San [;] Emilio, trap #5 in clearing. 300m. [;] XII/28/85–i/18/1986. I. Gauld, second label - [SE] Bosque San Emilio [;] 50yr old deciduous forest [;] [O] in clearing, fully [;] isolated part of day (ESUW). 1 ♀, top label - Costa Rica, Guanacaste Pr. [;] Guan. Conservation Area [;] Santa Rosa Hdq., 200m [;] Malaise trap 27–30 VI 1997 [;] 3x day L.J. van der Ent (ESUW). 2 ♀♀, top label - Costa Rica: Guanacaste [;] Santa Rosa National Pk. [;] 300m, Malaise trap SE-6-C [;] Bosque San Emilio, [;] deciduous forest [;] 50yr. old, Ian Gauld [;] 5.vii.1986, full shade (ESUW). 2 ♀♀, S.RosaPark, Guan. [;] C. Rica 21 May 77 and 5 Jan 77 [;] D.H. Janzen [;] Riparian (AEIC).

#### Comments.

This species is distinguished from all other species in Costa Rica by the bicolored basal flagellomeres.

#### Etymology.

Named for the province of Guanacaste where all the type series was collected.

**Figure 54.**
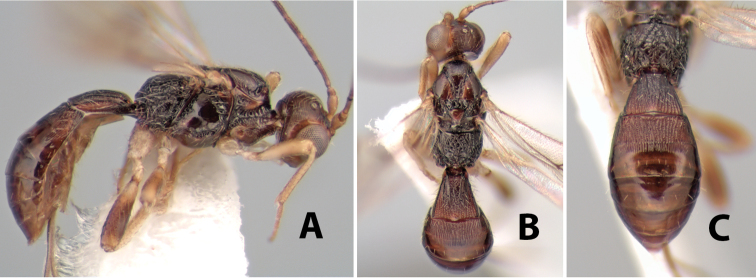
*Heterospilus guanacastensis* Marsh, sp. n., holotype.

### Heterospilus halidayi

Marsh sp. n.

http://zoobank.org/02B184B2-C86B-48BE-88AB-0CEF6ABC24F0

http://species-id.net/wiki/Heterospilus_halidayi

Figure 55

#### Female.

Body size: 3.5 mm. Color: head light honey yellow, vertex light brown behind ocelli; scape honey yellow without lateral brown stripe, flagellum brown; mesosoma dark brown except mesoscutum honey yellow; metasomal terga 1 dark brown, tergum dark brown or yellow medially and dark brown laterally, tergum 3 dark brown basally, honey yellow apically, terga 4–7 honey yellow; wing veins brown, stigma brown with yellow apex; legs yellow. Head: vertex transversely striate; frons transversely striate; face smooth; temple in dorsal view broad, equal to 1/2 eye width; malar space greater than 1/4 eye height; ocell-ocular distance slightly greater than twice diameter of lateral ocellus; 23–27 flagellomeres. Mesosoma: mesoscutal lobes granulate; notauli scrobiculate, meeting scutellum in triangular rugose area; scutellum smooth or weakly granulate; prescutellar furrow with 3 cross carinae, median one often more distinct; mesopleuron granulate; precoxal sulcus weakly scrobiculate, shorter than mesopleuron; venter granulate; propodeum with basal median areas margined, granulate, basal median carina present, areola not distinctly margined, areolar area rugose, lateral areas rugose posteriorly, granulate anteriorly. Wings: fore wing vein r less than 1/2 length vein 3RSa, vein 1cu-a beyond vein 1M; hind wing vein SC+R present, vein M+CU shorter than vein 1M. Metasoma: first tergum longitudinally costate, apical width 1/2 length; second tergum longitudinally costate; anterior transverse groove present, straight; posterior transverse groove present; third tergum costate basally, smooth apically; terga 4-7 smooth, terga 4-5 with basal transverse scrobiculate groove; ovipositor as long as or longer than metasoma.

#### Holotype female.

Top label (white, printed) - Costa Rica: San Jose [;] Cerro de la Muerte [;] 6km. N. San Gerardo [;] 2800m, IV.1992 [;] P. Hanson; second label (red, partially printed and hand written) - HOLOTYPE [;] Heterospilus [;] halidayi [;] P. Marsh. Deposited in ESUW.

#### Paratypes.

1 ♀, Costa Rica: Heredia [;] 3km. S. Puerto Viejo, [;] OTS, La Selva, 100m [;] xii.1992, P. Hanson (ESUW). 1 ♀, Costa Rica: San Jose [;] Zurqui de Moravia [;] 1600m, P. Hanson [;] ix.1995 (ESUW). 1 ♀, Costa Rica, Cartago Pr. [;] Dulce Nombre, Vivero [;] Linda Vista, 1300m [;] 1993: viii-x, P. Hanson (ESUW).

#### Comments.

The narrow metasomal tergum 1, the short fore wing vein r and the yellow mesoscutum are distinctive for this species.

#### Etymology.

Named for Irish entomologist A. H. Haliday in recognition of his pioneering work on the classification of parasitic Hymenoptera.

**Figure 55.**
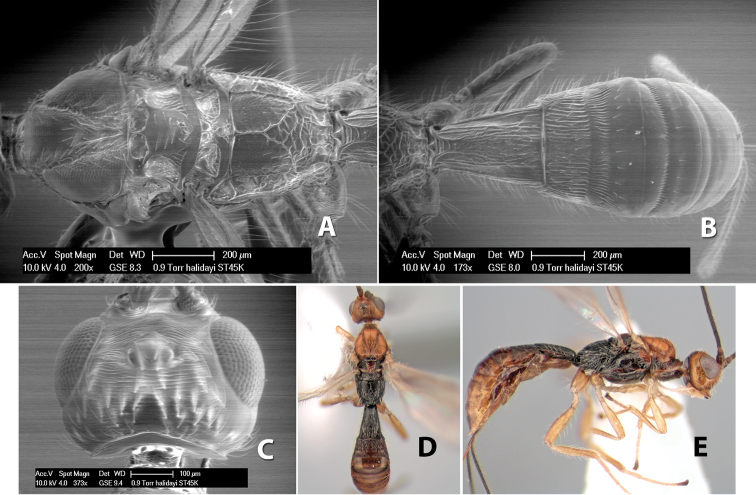
*Heterospilus halidayi* Marsh, sp. n., holotype.

### Heterospilus hansoni

Marsh sp. n.

http://zoobank.org/47363D16-71ED-4858-A4B6-07EE2C63B205

http://species-id.net/wiki/Heterospilus_hansoni

Figure 56

#### Female.

Body size: 3.0–4.0 mm. Color: entire body dark brown; scape yellow, without lateral longitudinal brown stripe, flagellum brown with apical 8–12 flagellomeres white; legs yellow; wing veins brown, stigma entirely brown. Head: vertex costate; frons costate; face rugose; temple in dorsal view slightly bulging, equal to 1/2 eye width; malar space greater than 1/4 eye height; ocell-ocular distance slightly greater than 2.5 times diameter of lateral ocellus; 26–33 flagellomeres. Mesosoma: mesoscutal lobes granulate; notauli scrobiculate, meeting at scutellum in triangular costate area; scutellum smooth; prescutellar furrow with one cross carina; mesopleuron smooth or very weakly granulate; precoxal sulcus weakly scrobiculate, shorter than mesopleuron; venter granulate; propodeum with basal median areas distinctly margined, basal median areas weakly granulate, basal median carina distinct but short, areola not distinctly margined, areolar area rugose, lateral areas rugose apically, smooth or granulate basally. Wings: fore wing vein r shorter than vein 3RSa, vein 1cu-a beyond vein 1M; hind wing vein SC+R present, vein M+CU shorter than 1M. Metasoma: first tergum longitudinally costate, length slightly greater than apical width; second tergum longitudinally costate, apical width less than 3 times length; anterior transverse groove present, straight; posterior transverse groove present, sometimes weakly indicated; third tergum costate basally, smooth apically; terga 4–7 smooth; ovipositor longer than metasoma.

#### Holotype female.

Top label (white, printed) - COSTA RICA: Puntar [;] Golfo Dulce. 10km W [;] Piedras Blancas, 100m [;] VI-VIII 1989, Hanson; second label (red, partially printed and hand written) - HOLOTYPE [;] Heterospilus [;] hansoni [;] P. Marsh. Deposited in ESUW.

#### Paratypes.

1 ♀, COSTA RICA, Puntar [;] Golfo Dulce, 3KM [;] S. W. Rincon, 10m [;] IX-XI 1989, Hanson (ESUW). 1 ♀, Costa Rica, Puntarenas [;] R.F. Golfo Dulce, [;] 3km SW. Rincon, 10m [;] vi.1991, Paul Hanson (ESUW). 1 ♀, Costa Rica: Puntarenas [;] R.F. Golfo Dulce, [;] 3km. SW. Rincon, 10m. [;] iii.1993 Paul Hanson coll. [;] Malaise, primary forest (ESUW). 3 ♀♀, Costa Rica: Limon, ACLAC [;] Central, R.B. Hitoy Cerere [;] Send. Espavel, 560m [;] 19.v–19.vi.1998, E. Rojas [;] L.S. 400702–570120 #52200 [;] Malaise Trap (ESUW). 5 ♀♀, COSTA RICA: Puntarenas [;] Reserva Forestal Golfo Dulce [;] 3km SW of Rincon, 10m [;] November 1992, July 1991 & October 1992, P. Hanson [;] primary forest, Malaise trap (ESUW). 2 ♀♀, Costa Rica: Alajuela, ACA [;] San Carlos, R.F. Arenal [;] Sendero Pilon, 600m, Malaise [;] 26.x–22.xi.1999, G. Carballo [;] L>N> 269100–457900 #54376 (ESUW). 4 ♀♀, COSTA RICA: Puntarenas [;] Rd. to Rincon, 10km W. [;] of Pan-Amer. Hwy. 100m [;] III-V 1989, Hanson & Gauld (ESUW). 1 ♀, COSTA RICA: Puntar [;] R.B. Carara, Estac. [;] Quebrada Bonita, 50m [;] viii-ix 1989, Hanson (ESUW). 1 ♀, Costa Rica: Puntarenas [;] Res. Forestal Golfo Dulce [;] 3km. SW Rincon, 10m [;] ii.1993, P. Hanson [;] Malaise, Primary forest (ESUW). 1 ♀, Costa Rica: Alajuela, 650m [;] San Carlos, R.F. Arenal, Send. [;] Pilon, 1–26.x.1999. G. Carballo [;] L.N. 269200-458050 #53929 [;] Malaise trap (ESUW). 1 ♀, COSTA RICA-Heredia Prov. [;] La Selva Biological Station [;] 10°26'N, 84°01'W, 100m [;] Malaise trap 11, #357 [;] 15.ii.1994 [;] Project ALAS (M.11.357) (ESUW). 1 ♀, Costa Rica: Limon [;] 30km N. Cariari, 100m [;] Sector Cocoon, Malaise [;] iii.1995, E. Rojas #4524 [;] L.N. 286000-567500 (ESUW). 1 ♀, Costa Rica, Heredia Prov. [;] OTS. La Selva, 100m [;] 1993 II-III P. Hanson (ESUW). 1 ♀, Costa Rica: Limon, Central [;] R.B. Hitoy Cerere, Est Hitoy [;] Cerere, Send. Toma de Agua [;] 100-140m, Malaise trap [;] 11.x–11.xi.1999, F. Umana [;] L.N. 184600-643400 #54013 (ESUW). 1 ♀, Costa Rica, San Jose [;] Zurqui De Moravia [;] 1600m. VII-1996 [;] P. Hanson (ESUW). 1 ♀, top label - Costa Rica: Limon [;] ACLAC, Central [;] Res. Biol. Hitoy Cerere [;] Est. Hitoy Cerere, 140m; second label - Sendero Toma de Agua [;] 17 Sept. - 10 Oct. 1999 [;] F. Umana, Malaise trap [;] LN 184600-643400 #53497 (ESUW). 1 ♀, Costa Rica, Puntarenas [;] R.F. Golfo Dulce, 5km [;] W. Piedras Blancas, 100m [;] I-1993, P. Hanson (ESUW). 1 ♀, COSTA RICA, Alajuela [;] Jabillos, 100m [;] 24/III/1989 [;] col. Paul Hanson (ESUW). 1 ♀, Costa Rica: Heredia [;] 3km. S. Puerto Viejo [;] OTS - La Selva, 100m [;] 16-30.IX.1992 [;] P. Hanson (ESUW). 1 ♀, Costa Rica: Heredia [;] Puerto Viejo [;] OTS, La Selva, 100m [;] iv.1991, P. Hanson (ESUW). 5 ♀♀, COSTA RICA, Heredia: [;] Est. Biol. La Selva, 50- [;] 150m, 10°26'N, 84°01W [;] Nov 1995, Apr 1996 and May 1996, INBio-OET (INBC).

#### Comments.

The entirely dark body and the partially rugose lateral area of the propodeum are distinct for this species.

#### Etymology.

Named for Paul Hanson in recognition of his years of collecting parasitic wasps in Costa Rica.

**Figure 56.**
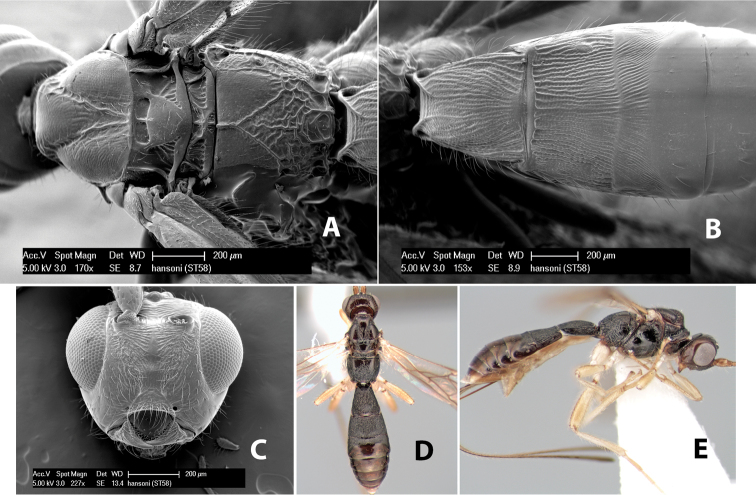
*Heterospilus hansoni* Marsh, sp. n.: **A–C** paratype **D–E** holotype.

### Heterospilus haplocarinus

Marsh sp. n.

http://zoobank.org/6912237E-D8DB-48B8-9014-AB0A5E3343EA

http://species-id.net/wiki/Heterospilus_haplocarinus

Figure 57

#### Female.

Body size: 3.0–4.0 mm. Color: head brown, face often and eye orbits always honey yellow; scape yellow with lateral longitudinal brown stripe, flagellum brown with apical 8–12 flagellomeres white; mesosoma dark brown; metasomal terga 1–4 dark brown, tergum 5 brown at base, yellow apically, terga 6-7 yellow; wing veins including stigma brown; legs yellow. Head: vertex transversely costate; frons transversely costate; face granulate; temple in dorsal view narrow, width less than 1/2 eye width; malar space equal to 1/4 eye height; ocell-ocular distance nearly 2.5 times diameter of lateral ocellus; 28–30 flagellomeres. Mesosoma: mesoscutal lobes granulate; notauli weakly scrobiculate or nearly smooth, meeting at scutellum in triangular costate area; scutellum granulate; prescutellar furrow with one median carina; mesopleuron granulate; precoxal sulcus smooth, shorter than mesopleuron; venter granulate; propodeum with basal median areas margined, granulate, basal median carina present, areola distinctly margined, areolar area rugose, lateral areas rugose posteriorly, granulate anteriorly. Area just above hind coxa with small but distinct tubercle. Wings: fore wing vein r shorter than vein 3RSa, vein 1cu-a beyond vein 1M; hind wing vein SC+R present, vein M+CU shorter than vein 1M. Metasoma: first tergum longitudinally costate-granulate, apical width slightly less than length; second tergum longitudinally costate; anterior transverse groove present, straight, rarely slightly sinuate; posterior transverse groove present; third tergum costate at base, granulate at apex; terga 4-7 granulate; ovipositor about 3/4 length of metasoma.

#### Holotype female.

Top label (white, printed) - COSTA RICA: [;] Puntar [;] Golfo Dulce, 3km [;] SW. Rincon, 10m [;] VI-VIII 1989, Hanson; second label (red, partially printed and hand written) - HOLOTYPE [;] Heterospilus [;] haplocarinus [;] P. Marsh. Deposited in ESUW.

#### Paratypes.

2 ♀♀, COSTA RICA, Guanac. [;] Estac. Pitilla. 9Km S [;] Santa Cecilia, 700m [;] IX/1988, I. Gauld (ESUW). 1 ♀, COSTA RICA: Puntar [;] Golfo Dulce 24km W [;] Piedras Blancas [;] 200m, vii-ix 1990 [;] Col. Paul Hanson (ESUW). 1 ♀, Costa Rica, Puntarenas [;] R.F. Golfo Dulce, 24km. W. [;] Piedras Blancas, 200m [;] VI-1991, P. Hanson (ESUW). 1 ♀, Costa Rica, Puntarenas [;] R.F. Golfo Dulce, 3km [;] SW Rincon, 10m [;] Malaise-primary forest [;] viii.1991, P. Hanson (ESUW).

#### Comments.

The single cross carina in the prescutellar furrow and the costate area where the notauli meet are distinctive for this species.

#### Etymology.

The specific name is from the Greek *haplos* meaning single in reference to the single cross carina in the prescutellar furrow.

**Figure 57.**
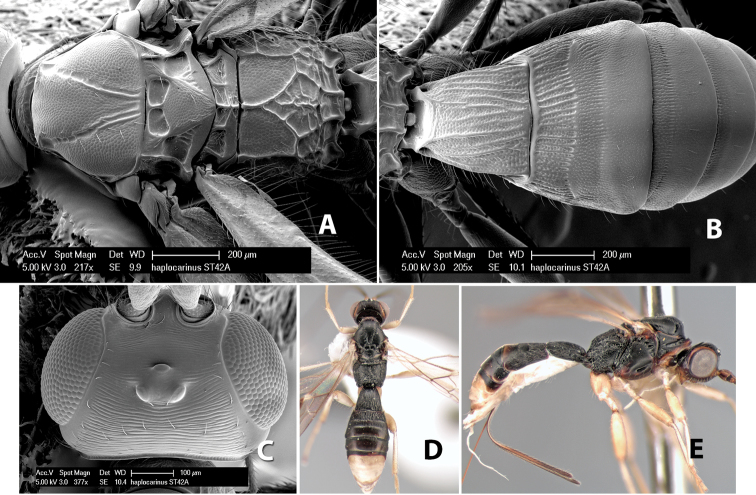
*Heterospilus haplocarinus* Marsh, sp. n.: **A–C** paratype **D–E** holotype.

### Heterospilus hedqvisti

Marsh sp. n.

http://zoobank.org/8538420B-979B-4158-8D99-7376538DD583

http://species-id.net/wiki/Heterospilus_hedqvisti

Figure 58

#### Female.

Body size: 2.5–3.0 mm. Color: head yellow; scape yellow without lateral brown stripe, flagellum brown; mesosoma dark brown, venter slightly lighter; metasoma dark brown; wing vein brown to light brown, stigma yellow; legs yellow. Head: vertex transversely striate; frons transversely striate; face striate; temple in dorsal view narrow, width less than 1/2 eye width; malar space greater than 1/4 eye height; ocell-ocular distance twice diameter of lateral ocellus; 21–23 flagellomeres. Mesosoma: somewhat flattened dorso-ventrally; mesoscutal lobes granulate; notauli scrobiculate, meeting at scutellum in wide rectangular rugose area; scutellum granulate; prescutellar furrow with 3–5 cross carinae; mesopleuron granulate; precoxal sulcus scrobiculate, shorter than mesopleuron; venter granulate; propodeum with basal median areas margined, granulate, basal median carina present, areola not distinctly margined, areolar area rugose, lateral areas entirely rugose. Wings: fore wing vein r slightly shorter than vein 3RSa, vein 1cu-a beyond vein 1M; hind wing vein SC+R present, vein M+CU as long as vein 1M. Metasoma: first tergum longitudinally costate, apical width equal to length; second tergum longitudinally costate; anterior transverse groove present, straight; posterior transverse groove present; third tergum costate basally, smooth apically; terga 4-7 smooth; ovipositor as long as metasomal tergum 1.

#### Holotype female.

Top label (white, printed) - Costa Rica: Guanacaste [;] Est. Cacao, 1000–1400m [;] 2km SW de Cerro Cacao [;] 1–9.iii.1996, A. Masis [;] L.N. 323100-375800 #7477 [;] Malaise trap; second label (red, partially printed and hand written) - HOLOTYPE [;] Heterospilus [;] hedqvisti [;] P. Marsh. Deposited in ESUW.

#### Paratypes.

1 ♀, Costa Rica: Guanacaste [;] Est. Biol. Maritza, 600m [;] i.1997, C. Zuniga, Malaise [;] L.N. 326900-373000 #47557 (ESUW).

#### Comments.

The yellow stigma and the short ovipositor are distinctive for this species.

#### Etymology.

Named for the Swedish braconidologist, Karl-Johan Hedqvist.

**Figure 58.**
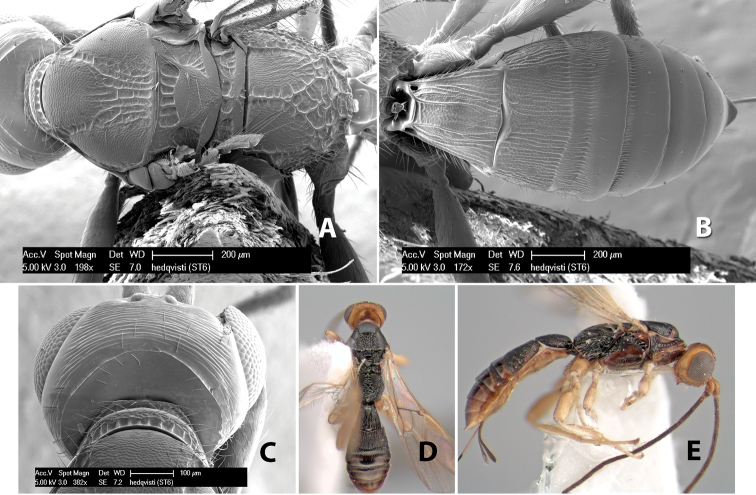
*Heterospilus hedqvisti* Marsh, sp. n.: **A–C** paratype D**–E** holotype.

### Heterospilus heredius

Marsh sp. n.

http://zoobank.org/2F19F421-6AC3-401A-8980-FB0E1F1D2B33

http://species-id.net/wiki/Heterospilus_heredius

Figure 59

#### Female.

Body size: 3.0–3.5 mm. Color: head light brown, often with vertex and frons darker brown; scape light brown, flagellum brown; mesosoma dark brown, occasionally with lighter spots laterally or along notauli; metasomal terga dark brown, apical terga slightly lighter brown; legs yellow; wing veins including stigma brown. Head: vertex transversely striate; frons weakly transversely striate; face distinctly rugose; temple in dorsal view broad, not sloping behind eye, width equal to 1/2 eye width; malar space greater than 1/4 eye height; ocell-ocular distance about twice diameter of lateral ocellus; 21–25 flagellomeres. Mesosoma: mesoscutal lobes granulate; notauli scrobiculate, meeting at scutellum in triangular rugose area; scutellum smooth; prescutellar furrow with 3 cross carinae; mesopleuron smooth; precoxal sulcus weakly scrobiculate, shorter than mesopleuron; venter smooth; propodeum with basal median areas distinctly margined, granulate, basal median carina present, areola distinctly margined, areolar area rugose, lateral areas entirely rugose. Wings: fore wing vein r shorter than vein 3RSa, vein 1cu-a beyond vein 1M; hind wing vein SC+R present, vein M+CU shorter than vein 1M. Metasoma: first tergum longitudinally costate, apical width equal to length; second tergum longitudinally costate; anterior transverse groove present, straight; posterior transverse groove absent; third tergum costate basally, smooth apically; terga 4-7 smooth; ovipositor length equal to length of terga 1+2.

#### Holotype female.

Top label (white, printed) - COSTA RICA-Heredia Prov. [;] La Selva Biological Station [;] 10°26'N, 84°01'W, 100m [;] Malaise trap 14, #260 [;] 1.xi.1993 [;] Project ALAS(M.14.260); second label (red, partially printed and hand written) - HOLOTYPE [;] Heterospilus [;] heredius [;] P. Marsh. Deposited in ESUW.

#### Paratypes.

1 ♀, same data as holotype (ESUW). 1 ♀, COSTA RICA-Heredia Prov. [;] La Selva Biological Station [;] 10°26'N, 84°01'W, 100m [;] Malaise trap 01, #334 [;] 15.ii.1994 [;] Project ALAS(M.01.334) (ESUW). 1 ♀, COSTA RICA, top label - Heredia [;] Est. Biol. La Selva, 50- [;] 150m, 10°26'N, 84°01'W [;] Oct 1993, INBio-OET; second label - 4 Octubre 1993 [;] Bosque secundario [;] M/14/232; third label - INBio bar code (ESUW).

#### Comments.

This species is distinguished by the coarsely rugose face.

#### Etymology.

Named after the Province of Heredia where the type series was collected.

**Figure 59.**
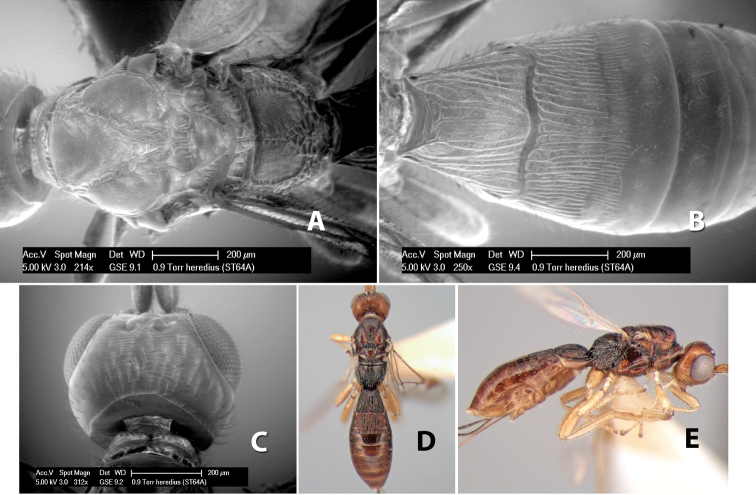
*Heterospilus heredius* Marsh, sp. n., holotype.

### Heterospilus huddlestoni

Marsh sp. n.

http://zoobank.org/4C08AFD9-EE7B-43B4-9C10-1DB7F6A5D85D

http://species-id.net/wiki/Heterospilus_huddlestoni

Figure 60

#### Female.

Body size: 3.0–3.5 mm. Color: head with vertex and frons brown, face and eye orbits honey yellow; scape yellow without lateral brown stripe, flagellum brown; metasoma dark brown; metasomal terga dark brown, terga 4–7 occasionally lighter; wing veins including stigma brown; legs yellow. Head: vertex transversely costate; frons transversely costate; face rugose; temple in dorsal view narrow, width less than 1/2 eye width; malar space equal to 1/4 eye height; ocell-ocular distance about 1.5 times diameter of lateral ocellus; 20–25 flagellomeres. Mesosoma: mesoscutal lobes granulate, median lobe often with median longitudinal groove posteriorly and ridge anteriorly; notauli scrobiculate, meeting at scutellum in wide rectangular costate area; scutellum granulate; prescutellar furrow with 3-5 cross carinae; mesopleuron granulate; precoxal sulcus scrobiculate, shorter than mesopleuron; venter granulate; propodeum with basal median areas margined, rugose or rugose-granulate, basal median carina absent, areola not distinctly margined, areolar area rugose, lateral areas entirely rugose. Wings: fore wing vein r shorter than vein 3RSa, vein 1cu-a beyond vein 1M; hind wing vein SC+R present, vein M+CU shorter than vein 1M. Metasoma: first tergum longitudinally costate, apical width equal to length; second tergum longitudinally costate; anterior transverse groove weak, often absent; posterior transverse groove absent; third tergum smooth; terga 4-7 smooth; ovipositor equal to length of metasomal terga 1 and 2 combined.

#### Holotype female.

Top label (white, printed) - COSTA RICA-Heredia Prov. [;] La Selva Biological Station [;] 10°26'N, 84°01'W, 100m [;] Malaise trap 10, #388 [;] 4.iv.1994 [;] Project ALAS (M.10.388); second label (red, partially printed and hand written) - HOLOTYPE [;] Heterospilus [;] huddlestoni [;] P. Marsh. Deposited in ESUW.

#### Paratypes.

1 ♀, Costa Rica: Limon [;] 30km N Cariari, 100m [;] Sector Cocori, Malaise [;] iii.1995, E. Rojas #4524 [;] L.N. 286000-567500 (ESUW). 1 ♀, top label - Costa Rica: Guanacaste [;] Santa Rosa National Pk. [;] 300m, Malaise, Ian Gauld [;] 10–31.i.1987; second label - Bosque Humedo [;] mature dry forest [;] high proportion [;] evergreen species [;] sun; third label - BH-11-O [;] 10-31.i.87 (ESUW).

#### Comments.

The dark brown body and the wide costate area where the notauli meet are distinctive for this species.

#### Etymology.

Named for the late British braconidologist, Tom Huddleston.

**Figure 60.**
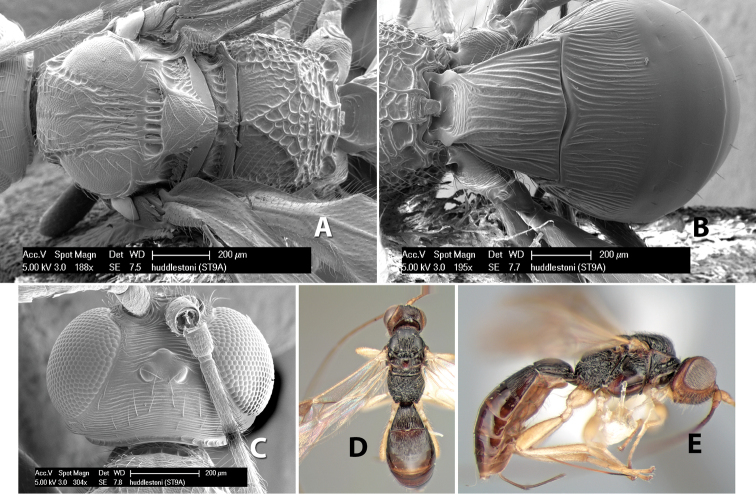
*Heterospilus huddlestoni* Marsh, sp. n.: **A–C** paratype **D–E** holotype.

### Heterospilus itza

Marsh sp. n.

http://zoobank.org/0A6F8473-2A6B-40D3-AE91-E4C15FD0CD4F

http://species-id.net/wiki/Heterospilus_itza

Figure 61

#### Female.

Body size: 2.0 mm. Color: head brown; scape yellow without lateral longitudinal brown stripe, flagellum brown, first flagellomere lighter; mesosoma and metasoma dark brown; wing veins including stigma brown; legs yellow. Head: vertex weakly striate, smooth near eyes; frons smooth; face smooth; temple in dorsal view narrow, width about 1/2 eye width; malar space slightly greater than eye height; ocell-ocular distance 2.5 times diameter of lateral ocellus; 14 flagellomeres. Mesosoma: mesoscutal lobes granulate; notauli scrobiculate, meeting at scutellum in triangular rugose area; scutellum granulate; prescutellar furrow with 1 cross carina; mesopleuron granulate; precoxal sulcus scrobiculate, shorter than mesopleuron; venter granulate; propodeum with basal median areas margined, granulate, basal median carina absent, areola not distinctly margined, areolar area rugose, lateral areas entirely rugose. Wings: fore wing vein r slightly shorter than vein 3RSa, vein 1cu-a beyond vein 1M; hind wing vein SC+R absent, vein M+CU shorter than vein 1M. Metasoma: first tergum longitudinally costate, apical width equal to length; second tergum longitudinally costate; anterior transverse groove present, straight; posterior transverse groove absent; third tergum costate at extreme base, smooth apically; terga 4-7 smooth; ovipositor slightly longer than metasoma.

#### Holotype female.

Top label (white, printed) - COSTA RICA: Puntarenas [;] Rd. to Rincon, 24km W. [;] of Pan-Amer. Hwy, 200m [;] II-III 1989, Hanson & Gauld; second label (red, partially printed and hand written) - HOLOTYPE [;] Heterospilus [;] itza [;] P. Marsh. Deposited in ESUW.

#### Paratypes.

1 ♀, S.RosaPark, Guan. [;] C. Rica 31 Oct 77 [;] D.H. Janzen [;] Riparian (AEIC).

#### Comments.

The granulate mesopleuron, single cross carina in the prescutellar furrow, smooth face, longer ovipositor and the absent hind wing vein SC+R are distinctive for this species.

#### Etymology.

Named for the Itza, an indigenous Mayan people of Guatemala.

**Figure 61.**
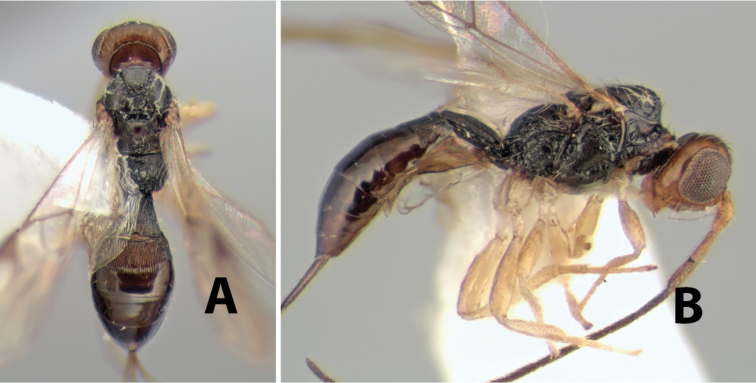
*Heterospilus itza* Marsh, sp. n., holotype.

### Heterospilus jakaltek

Marsh sp. n.

http://zoobank.org/A52E7CCA-EDDD-4825-8ADE-E0F68B3CF01C

http://species-id.net/wiki/Heterospilus_jakaltek

Figure 62

#### Female.

Body size: 2.0 mm. Color: head brown; scape yellow without lateral longitudinal brown stripe, flagellum brown with basal flagellomere yellow; mesosoma dark brown; metasomal tergum 1 dark brown, terga 2-7 light brown; wing veins including stigma brown; legs yellow. Head: vertex weakly striate medially, smooth near eyes; frons smooth; face smooth; temple in dorsal view broad, bulging behind eye, width slightly greater than 1/2 eye width; malar space equal to 1/4 eye height; ocell-ocular distance about 2.5 times diameter of lateral ocellus; 14 flagellomeres. Mesosoma: mesoscutal lobes granulate; notauli scrobiculate, meeting at scutellum in triangular rugose area; scutellum weakly granulate; prescutellar furrow with 3 cross carinae; mesopleuron weakly granulate; precoxal sulcus weakly scrobiculate, shorter than mesopleuron; venter smooth; propodeum with basal median areas margined, granulate, basal median carina present, areola not distinctly margined, areolar area rugose, lateral areas entirely rugose. Wings: fore wing vein r shorter than vein 3RSa, vein 1cu-a beyond vein 1M; hind wing vein SC+R absent, vein M+CU shorter than 1M. Metasoma: first tergum longitudinally costate, apical width equal to length; second tergum longitudinally costate; anterior transverse groove present, straight; posterior transverse groove present; third tergum costate at base, smooth apically; terga 4-7 smooth; ovipositor longer than metasoma.

#### Holotype female.

Top label (white, partially printed and hand written) - Costa Rica: Guanacaste [;] Santa Rosa Natl. Park [;] 300m, ex. Malaise trap [;] Site #: (blank) [;] Dates: 7–28.xii.1985 [;] I.D. Gauld & D. Janzen; second label (white, printed) - [SE] Bosque San Emilio [;] 50yr old deciduous forest [;] [C] more or less fully [;] shaded as possible; third label (red, partially printed and hand written) - HOLOTYPE [;] Heterospilus [;] jakaltek [;] P. Marsh. Deposited in ESUW.

#### Paratypes.

Known only from the holotype.

#### Comments.

The long ovipositor and absence of hind wing vein SC+R are distinctive for this species.

#### Etymology.

Named for the Jakaltek, an indigenous Mayan people of Guatemala.

**Figure 62.**
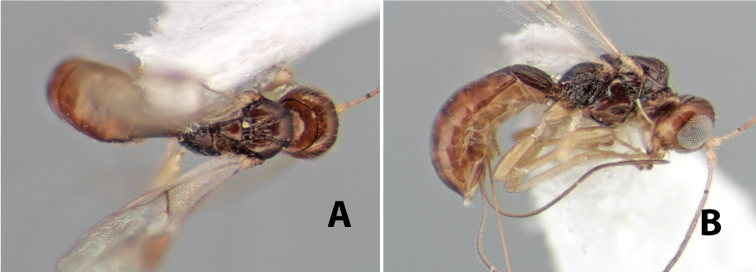
*Heterospilus jakaltek* Marsh, sp. n., holotype.

### Heterospilus janzeni

Marsh sp. n.

http://zoobank.org/D1C1E448-CC6E-4A15-AA2C-53ED3E6BB8EB

http://species-id.net/wiki/Heterospilus_janzeni

Figure 63

#### Female.

Body size: 3.0–3.5 mm. Color: head dark brown; scape brown without lateral longitudinal brown stripe, flagellum brown; mesosoma dark brown; metasoma dark brown, apical terga sometimes slightly lighter; legs yellow, apical 3/4 of femora light brown; wing veins including stigma brown. Head: vertex transversely costate; frons weakly costate, often smooth; face smooth; temple in dorsal view narrow, sloping behind eye, equal to 1/2 eye width; malar space greater than 1/4 eye height; ocell-ocular distance about twice diameter of lateral ocellus; 22–30 flagellomeres. Mesosoma: mesoscutal lobes smooth; notauli scrobiculate, meeting at scutellum in triangular costate area; scutellum smooth, rarely weakly granulate; prescutellar furrow with 3–5 cross carinae; mesopleuron smooth; precoxal sulcus scrobiculate, shorter than mesopleuron; venter smooth; propodeum with basal median areas distinctly margined, weakly rugose, basal median carina absent, rarely very short, areola not distinctly margined, areolar area rugose, lateral areas entirely rugose. Wings: fore wing vein r about equal to length of vein 3RSa, vein 1cu-a beyond vein 1M; hind wing vein SC+R present, vein M+CU shorter than vein 1M. Metasoma: first tergum longitudinally costate-rugose, apical width equal to length; second tergum longitudinally costate; anterior transverse groove present, straight; posterior transverse groove present; third tergum costate at base, smooth at apex; terga 4-7 smooth; ovipositor as long as metasoma.

#### Holotype female.

Top label (white, partially printed and hand written) - Costa Rica: Guanacaste [;] Santa Rosa Natl. Park [;] 300m, ex. Malaise trap [;] Site #: BH-12-C [;] Dates: 18.x–8.xi.1986 [;] I.D. Gauld & D. Janzen; second label (white, printed) - [BH] Bosque Humedo [;] mature evergreen dry forest [;] [C] more or less fully [;] shaded as possible; third label (red, partially printed and hand written) - HOLOTYPE [;] Heterospilus [;] janzeni [;] P. Marsh. Deposited in ESUW.

#### Paratypes.

1 ♀, top label - Costa Rica: Guanacaste [;] Santa Rosa Natl. Park [;] 300m, ex. Malaise trap [;] Site #: SE-O-5 [;] Dates: 18.x–8.xi.1986 [;] I.D. Gauld & D. Janzen; second label - [SE] Bosque San Emilio [;] 50yr old deciduous forest [;] [O] in clearing, fully [;] isolated part of day (ESUW). 1 ♀, top label - Costa Rica: Guanacaste [;] Santa Rosa Natl. Park [;] 300m, ex. Malaise trap [;] Site #: 6 [;] Dates: 6-26.x.1986 [;] I.D. Gauld & D. Janzen; second label - [SE] Bosque San Emilio [;] 50yr old deciduous forest [;] [C] more or less fully [;] shaded as possible (ESUW). 5 ♀♀, Costa Rica: Guanacaste ACT [;] Bagaces, PN Palo Verde [;] Sec. Palo Verde, Cero [;] Guaycán, 212m, Malaise [;] 18.viii–14.ix.1999 and 13.ix–13.x.1999, I. Jimenez [;] L.N. 259350-389600 #53254 and 53499 (ESUW). 3 ♀♀, top label - Costa Rica: Guanacaste [;] Santa Rosa National Pk. [;] 300m, Malaise, Ian Gauld [;] 27.ix–18.x.1986; second label - Bosque San Emilio [;] 50yr. old deciduous [;] forest, fully shade; third label, SE-8-C [;] 27.ix–18.x.86 (ESUW). 1 ♀, Costa Rica: Puntarenas [;] Pen. Osa, Puerto [;] Jimenez, 10m, December [;] 1990, P. Hanson, Malaise (ESUW). 1 ♀, S.RosaPark,Guan, Guan. [;] C. Rica 15 May, 76 [;] D. H. Janzen [;] Riparian (AEIC).

#### Comments.

This species is distinguished by the brown stigma and flagellum and the entirely rugose sides of the propodeum.

#### Etymology.

Named for Dan Janzen in recognition of his biodiversity studies in Costa Rica.

**Figure 63.**
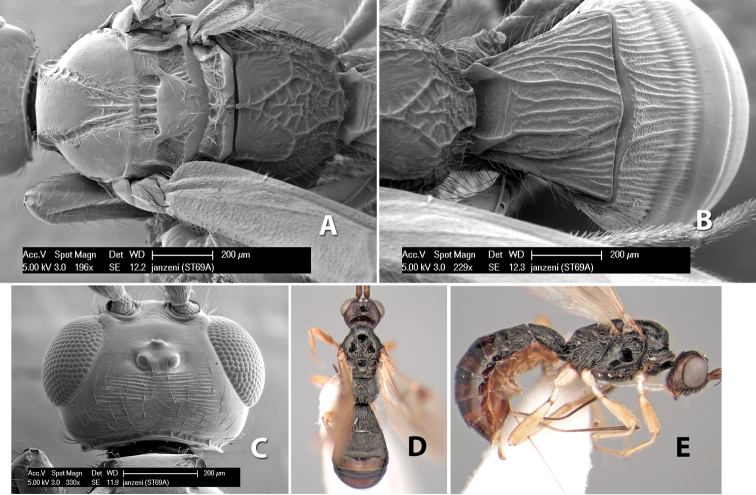
*Heterospilus janzeni* Marsh, sp. n.: **A–C** paratype **D–E** holotype.

### Heterospilus kiefferi

Marsh sp. n.

http://zoobank.org/F5BB6E4C-0D1C-4854-A24C-7F8650A286F3

http://species-id.net/wiki/Heterospilus_kiefferi

Figure 64

#### Female.

Body size: 5.0 mm. Color: head brown to dark brown; scape honey yellow with lateral longitudinal brown stripe, flagellum brown with white annulus of 4-5 flagellomeres behind apical 6 brown flagellomeres; wing veins brown, stigma yellow; legs yellow. Head: vertex weakly transversely striate; frons smooth; face smooth; temple in dorsal view brown and bulging behind eye, width equal to 1/2 eye width; malar space greater than 1/4 eye height; ocell-ocular distance about 2.5 times diameter of lateral ocellus; 28 flagellomeres. Mesosoma: mesoscutal lobes weakly granulate, lateral lobes smooth laterally; notauli scrobiculate, meeting at scutellum in triangular rugose area; scutellum smooth; prescutellar furrow with 3 cross carinae; mesopleuron weakly granulate dorsally, nearly smooth above precoxal sulcus; precoxal sulcus scrobiculate, shorter than mesopleuron; venter weakly granulate or smooth; propodeum with basal median areas margined, granulate, basal median carina present, areola not distinctly margined, areolar area rugose, lateral areas entirely rugose, distinct tubercle present laterally on each side of base of metasomal tergum 1 above hind coxa. Wings: fore wing vein r shorter than vein 3RSa, vein 1cu-a beyond vein 1M; hind wing vein SC+R present, vein M+CU shorter than vein 1M. Metasoma: first tergum longitudinally costate, length slightly greater than apical width; second tergum longitudinally costate; anterior transverse groove present, straight; posterior transverse groove present; third tergum costate basally, smooth apically; terga 4–7 smooth; ovipositor 3/4 length of metasoma.

#### Holotype female.

Top label (white, printed) - Costa Rica: Cartago [;] 4km NE Canon [;] Genesis II, 2350m [;] viii.1996, P. Hanson; second label (red, partially printed and hand written) - HOLOTYPE [;] Heterospilus [;] kiefferi [;] P. Marsh. Deposited in ESUW.

#### Paratypes.

Known only from the holotype.

#### Comments.

The tubercle on the propodeum just above the hind coxa, the partially smooth mesoscutal lobes and the broad temple distinguish this species.

#### Etymology.

Named for J. J. Kieffer who described some Braconidae in the late 1880s and early 1900s.

**Figure 64.**
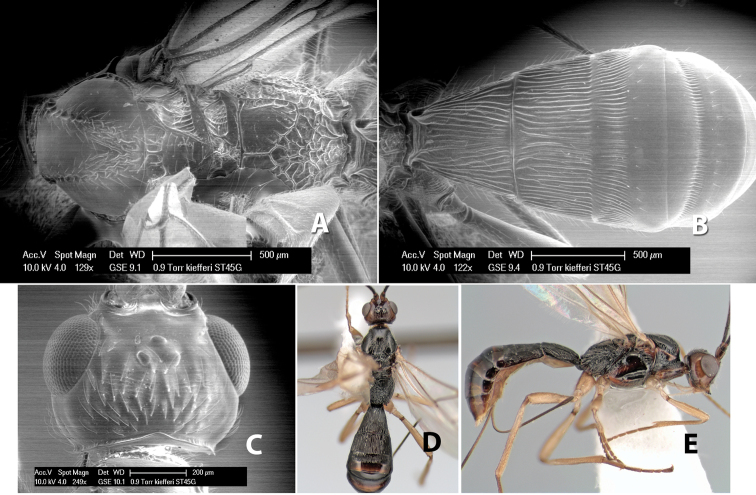
*Heterospilus kiefferi* Marsh, sp. n., holotype.

### Heterospilus kulai

Marsh sp. n.

http://zoobank.org/5126A9EE-206C-4F1D-A5A1-21CC1C6276F9

http://species-id.net/wiki/Heterospilus_kulai

Figure 65

#### Female.

Body size: 3.5–5.0 mm. Color: head dark brown; scape light brown, flagellum dark brown with apical 8-10 flagellomeres white; mesosoma dark brown; metasoma dark brown, tergum 2 yellow or light brown medially, terga 4–7 brown, often yellow laterally and/or apically on each tergum; legs bicolored yellow and brown, femora brown medially, hind tibia dark brown at base, light brown on apical half, hind tarsus brown; wing veins, including stigma, brown. Head: vertex transversely costate; frons transversely costate; face striate; temple narrow, not bulging behind eye, width equal to 1/2 eye width; malar space 1/4 eye height; ocell-ocular distance about twice diameter of lateral ocellus; 33–42 flagellomeres. Mesosoma: mesoscutal lobes granulate; notauli scrobiculate, meeting before prescutellar furrow in wide longitudinally costate area; scutellum smooth; prescutellar furrow with 3–5 cross carinae; mesopleuron smooth; precoxal sulcus smooth, shorter than mesopleuron; venter smooth; propodeum with basal median areas distinctly margined, basal median areas smooth, basal median carina present, areola not distinctly margined, areolar area rugose, lateral areas rugose with smooth area apically. Wings: fore wing vein r shorter than vein 3RSa, vein 1cu-a beyond vein 1M; hind wing vein SC+R present, vein M+CU shorter than 1M. Metasoma: first tergum length greater than apical width, longitudinally costate, rugose baso-medially, lateral costae curving toward median line at apex of tergum; second tergum longitudinally costate, apical width less than 3 times length; anterior transverse groove present, very slightly sinuate; posterior transverse groove present but indistinct; third tergum costate basally, granulate apically; terga 4–7 costate at base, granulate apically; ovipositor longer than metasoma.

#### Holotype female.

Top label (white, printed) - COSTA RICA Puntarenas [;] Reserva Forestal Golfo Dulce [;] 3km SW Rincon. 10m. primary [;] forest, xii.1992, P. Hanson; second label (red, partially printed and hand written) - HOLOTYPE [;] Heterospilus [;] kulai [;] P. Marsh. Deposited in ESUW.

#### Paratypes.

1 ♀, same data as holotype (ESUW). 1 ♀, Costa Rica: Puntarenas [;] Send. a Rio Clara, Est. Sirena [;] 1–100m, 17.vi–4.ix.1991 [;] J.C. Saborio & G. Fonseca [;] I. S. 270500-508300 #6883 (ESUW). 1 ♀, Costa Rica: Limon, ACLAC [;] Central, R.B. Hitoy Cerere [;] Est. Hitoy Cerere [;] 140m. Malaise trap [;] 17.vi–17.vii.1999. F. Umana [;] L.N. 184600-643400 #52861 (ESUW). 1 ♀, COSTA RICA-Heredia Prov. [;] La Selva Biological Station [;] 10°26'N, 84°01'W 100m [;] Malaise trap 07, #392 [;] 30.vi.1995 [;] Project ALAS (M 07 392) (ESUW). 1 ♀, Costa Rica: Puntarenas [;] R. F. Golfo Dulce, 24km. W [;] Piedras Blancas, 200m [;] III.1993. P. Hanson (ESUW). 1 ♀, COSTA RICA, Heredia [;] 3km. S. Puerto Viejo [;] OTS-La Selva, 100m [;] ii-iii.1993 P. Hanson (MICR).

#### Comments.

This species is similar to *Heterospilus laselvus* but is distinguished by the white apex of the flagellum and the costate area where the notauli meet.

#### Etymology.

Named for my good friend Robert Kula, braconidologist for the USDA’s Systematic Entomology Lab. at the Smithsonian Institution, Washington, DC, a chair that I kept warm for him for many years.

**Figure 65.**
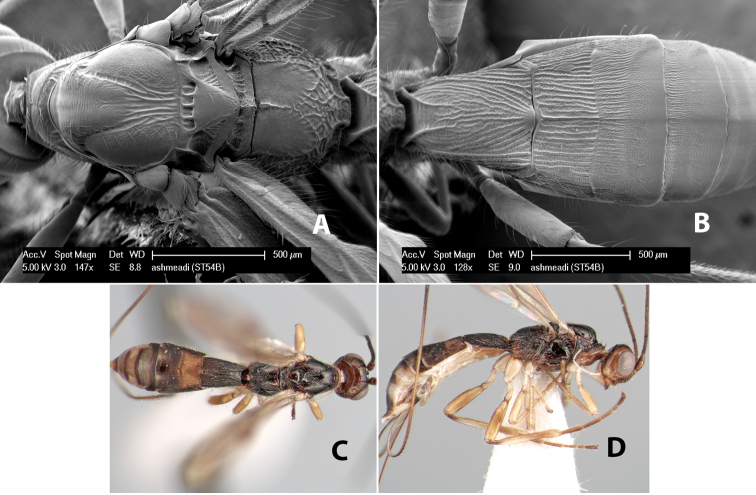
*Heterospilus kulai* Marsh, sp. n.: **A–B** paratype **C–D** holotype.

### Heterospilus kuna

Marsh sp. n.

http://zoobank.org/F77099C3-D88C-413D-88D6-1B7FDA0397EA

http://species-id.net/wiki/Heterospilus_kuna

Figure 66

#### Female.

Body size: 2.5 mm. Color: head with vertex and frons brown, face and temple honey yellow; scape yellow without lateral longitudinal brown stripe, flagellum brown; mesosoma dark brown, metasoma dark brown, apical terga slightly lighter; wing veins including stigma brown; legs yellow. Head: vertex weakly striate medially, smooth near eyes; frons smooth; face smooth; temple in dorsal view somewhat bulging, width equal to 1/2 eye width; malar space greater than 1/4 eye height; ocell-ocular distance nearly 2.5 times diameter of lateral ocellus; 18 flagellomeres. Mesosoma: mesoscutal lobes granulate; notauli scrobiculate, meeting at scutellum in triangular costate area; scutellum granulate; prescutellar furrow with 1 cross carina; mesopleuron granular; precoxal sulcus scrobiculate, shorter than mesopleuron; venter granulate; propodeum with basal median areas margined, granulate, basal median carina present, areola distinctly margined, areolar area rugose, lateral areas entirely rugose. Wings: fore wing vein r shorter than vein 3RSa, vein 1cu-a beyond vein 1M; hind wing vein SC+R present, vein M+CU shorter than vein 1M. Metasoma: first tergum longitudinally costate, apical width equal to length; second tergum longitudinally costate; anterior transverse groove present, straight; posterior transverse groove present; third tergum costate basally, smooth apically; terga 4-7 smooth; ovipositor 3/4 length of metasoma.

#### Holotype female.

Top label (white, printed) - Costa Rica: Limon [;] 30km N Cariari, 100m [;] Sector Cocori, Malaise [;] iii.1995, E. Rojas #4524 [;] L.N. 286000-567500; second label (red, partially printed and hand written) - HOLOTYPE [;] Heterospilus [;] kuna [;] P. Marsh. Deposited in ESUW.

#### Paratypes.

Known only from the holotype.

#### Comments.

The granulate mesopleuron, smooth face, single cross carina in the prescutellar furrow and the presence of hind wing vein SC+R are distinctive for this species.

#### Etymology.

Named for the Kuna, an indigenous people of Panama.

**Figure 66.**
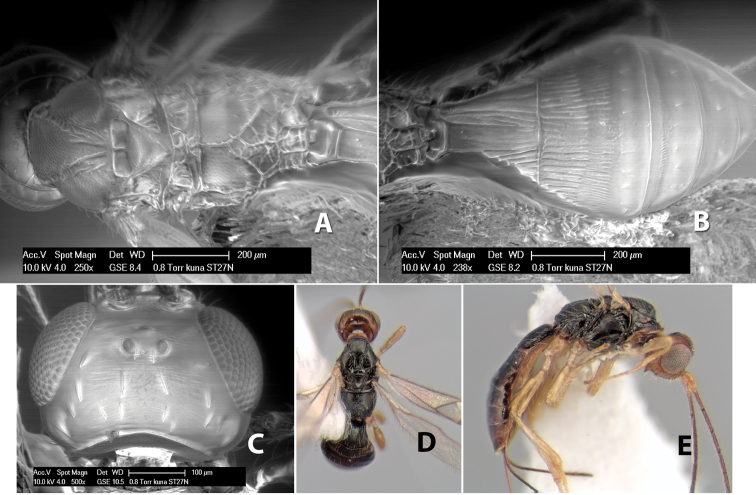
*Heterospilus kuna* Marsh, sp. n., holotype.

### Heterospilus lapierrei

Marsh sp. n.

http://zoobank.org/B38DCB8E-CDF6-4203-91A1-9C45F08BDD63

http://species-id.net/wiki/Heterospilus_lapierrei

Figure 67

#### Female.

Body size: 4.0 mm. Color: body entirely dark brown or black; scape yellow without lateral longitudinal brown stripe, flagellum brown; wing vein brown, stigma bicolored brown with yellow apex; legs yellow. Head: vertex weakly striate behind antennae, smooth near eyes; frons smooth; face smooth; temple in dorsal view narrow, width less than 1/2 eye width; malar space greater than 1/4 eye height; ocell-ocular distance greater than 2.5 times diameter of lateral ocellus; 31 flagellomeres. Mesosoma: mesoscutal lobes weakly granulate and shining, nearly smooth; notauli scrobiculate, meeting at scutellum in triangular costate area; scutellum smooth; prescutellar furrow with 3 cross carinae; mesopleuron weakly granulate; precoxal sulcus scrobiculate, shorter than mesopleuron; venter weakly granulate; propodeum with basal median areas margined, granulate, basal median carina present, areola distinctly margined, areolar area rugose, lateral areas entirely rugose. Wings: fore wing vein r nearly as long as vein 3RSa, vein 1cu-a beyond vein 1M; hind wing vein SC+R present, vein M+CU shorter than vein 1M. Metasoma: first tergum longitudinally costate, length about 1.5 times apical width; second tergum longitudinally costate; anterior transverse groove present, slightly sinuate; posterior transverse groove weakly indicated as transverse indentation; third tergum costate at base, smooth at apex; terga 4–7 smooth; ovipositor longer than metasoma.

#### Holotype female.

Top label (white, partially printed and hand written) - COSTA RICA: Heredia [;] Est. Biol. La Selva, 50–150m, 10°26'N, 84°01'W [;] X.1999; second label (white, partially printed and hand written) - Coll. L. M. LaPierre [;] Host: *Cecropia* [;] *obtusifolia*, Zyg...? (hand writing not legible) [;] ID# 0099-1097; third label (red, partially printed and hand written) - HOLOTYPE [;] Heterospilus [;] lapierrei [;] P. Marsh. Deposited in ESUW.

#### Paratypes.

Known only from the holotype.

#### Biology.

The holotype was reared from a weevil infesting *Cecropia obtusifolia* Bertol. Louis LaPierre (per. comm.) states that the host was identified as *Pseudolechriops coleyae* Hespenheide & LaPierre.

#### Comments.

This species is distinguished by the black body and nearly smooth and shining mesoscutum.

#### Etymology.

Named for the collector, L. M. LaPierre.

**Figure 67.**
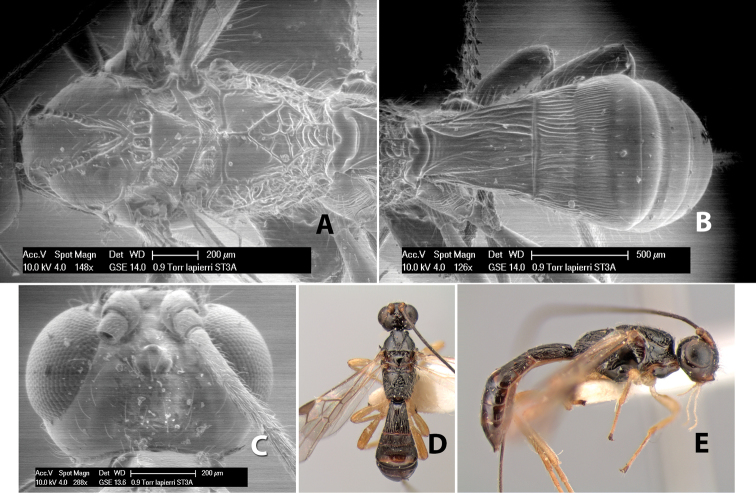
*Heterospilus lapierrei* Marsh, sp. n., holotype.

### Heterospilus laselvus

Marsh sp. n.

http://zoobank.org/76BDE515-A136-460F-A74C-F22E62B5FF06

http://species-id.net/wiki/Heterospilus_laselvus

Figure 68

#### Female.

Body size: 3.5–4.0 mm. Color: head dark brown, malar space, lower face and eye orbits usually yellow, face occasionally yellow; scape yellow without lateral longitudinal brown stripe, flagellum entirely brown; mesosoma dark brown; metasomal terga 1–2 dark brown, terga 3–6 brown at base, yellow at apex, tergum 7 brown; fore and middle legs yellow, hind coxa and trochanters yellow, hind femur yellow with middle half brown, hind tibia yellow with brown at base and on apical half, hind tarsus brown; wing veins brown, stigma entirely brown. Head: vertex transversely costate; frons transversely costate; face striate; temple narrow, not bulging behind eye, width less than 1/2 eye width; malar space greater than 1/4 eye height; ocell-ocular distance about twice diameter of lateral ocellus; 30–40 flagellomeres. Mesosoma: mesoscutal lobes weakly granulate; notauli scrobiculate, meeting at prescutellar furrow in triangular rugose area; scutellum smooth; prescutellar furrow with one cross carina; mesopleuron smooth; precoxal sulcus smooth, shorter than length of mesopleuron; venter smooth; propodeum with basal median areas distinctly margined, basal median areas weakly granulate, basal median carina distinct, areola not distinctly margined, areolar area rugose, lateral areas rugose posteriorly, smooth or weakly granulate anteriorly. Wings: fore wing vein r shorter than vein 3RSa, vein 1cu-a beyond vein 1M; hind wing vein SC+R present, vein M+CU shorter than 1M. Metasoma: first tergum rugose-costate, length greater than apical width; second tergum longitudinally costate, apical width less than 3 times length; anterior transverse groove present, straight; posterior transverse groove weak or nearly absent; third tergum costate at base, smooth at apex; terga 4–7 granulate at base, smooth at apex; ovipositor longer than metasoma.

#### Holotype female.

Top label (white, printed) - Costa Rica, Heredia [;] 3km. S. Puerto Viejo [;] OTS-LaSelva. 100m [;] IV-V-1993. P. Hanson; second label (red, partially printed and hand written) - HOLOTYPE [;] Heterospilus [;] laselvus [;] P. Marsh. Deposited in ESUW.

#### Paratypes.

1 ♀, same data as holotype except date of I-II-1993 (ESUW). 1 ♀, top label - COSTA RICA, Heredia [;] Est. Biol. La Selva, 50- [;] 150m, 10°26'N, 84°01W [;] Mar 1993, INBio-OET; second label - 02 Marzo 1993 [;] M/03/018 [;] Bosque Primario (ESUW). 1 ♀, top label - COSTA RICA: Heredia [;] Pr: La Selva Biol. Sta. [;] 3km S. Pto. Viejo [;] 10°26'N, 84°01W; second label - 23-26.V.1988 B.V. Broen [;] Malaise trap - SAT 100 [;] Secondary Forest (ESUW). 1 ♀, COSTA RICA: [;] Heredia, Chilamate [;] 75m, xii 89-iii 1990 [;] Hanson & Godoy (ESUW). 1 ♀, Costa Rica: Limon, ACLAC [;] Central, R.B. Hitoy Cerere, [;] Est. Hitoy Cerere, Send. Toma [;] de Agua, 100m, 17.iv–8.v.1999 [;] F. Umana, Malaise trap [;] L.N. 184600-643400 #52757 (ESUW). 3 ♀♀, Costa Rica: Limon, ACLAC [;] Central, R.B. Hitoy Cerere, [;] Send. Espavel, 560m [;] 19.v–16.vi.1998, E. Rojas [;] L.S. 400702-570120 #52200 [;] Malaise Trap (ESUW). 2 ♀♀, COSTA RICA, Limón [;] 4km NE Bribri [;] 50m, IX-XI 1989 [;] col. Paul Hanson (ESUW). 1 ♀, Costa Rica, Carthago Pr. [;] Dulce Nombre, Vivero [;] Linda Vista, 1300m [;] 1993:viii-x, P. Hanson (ESUW). 1 ♀, Costa Rica: Puntarenas [;] R.F. Golfo Dulce, 24km. W [;] Piedras Blancas, 200m [;] III.1993, P. Hanson (ESUW). 1 ♀, top label - Costa Rica: Guanacaste [;] Santa Rosa Natl. Park [;] 300m, ex. Malaise trap [;] Site #: BH-9-O [;] Dates: 26.vii–14.viii.1986 [;] I.D. Gauld & D. Janzen; second label - [BH] Bosque Humedo [;] mature evergreen forest [;] [O] in clearing, fully [;] isolated part of day (ESUW). 1 ♀, COSTA RICA, Guanac. [;] Estac.Pitilla,9Km S [;] Santa Cecilia, 700m [;] VI/1989, I. Gauld (MICR). 1 ♀, COSTA RICA, Limón [;] 4km NE Bribri [;] 50m, VII-IX-1990 [;] col. Paul Hanson (MICR).

#### Comments.

This species is similar to *Heterospilus ashmeadi* but is distinguished by the rugose area where the notauli meet and the dark brown flagellum.

#### Etymology.

Named after the biological station La Selva where several of the type series were collected.

**Figure 68.**
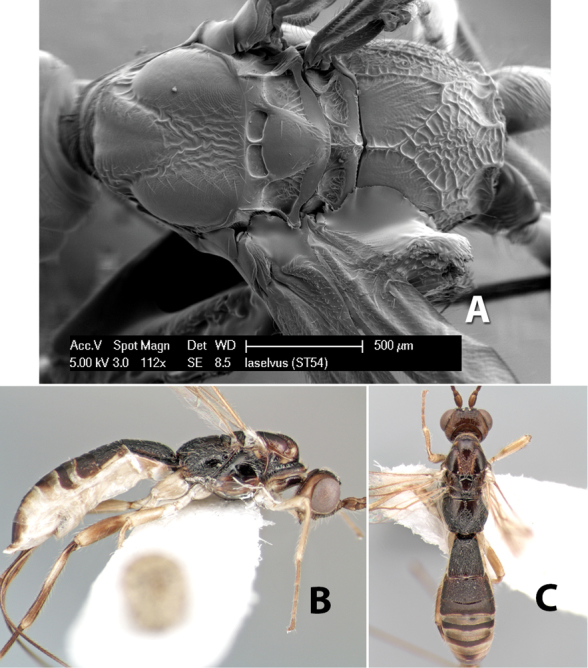
*Heterospilus laselvus* Marsh, sp. n.: **A** paratype **B–C** holotype.

### Heterospilus leioenopus

Marsh sp. n.

http://zoobank.org/3DF03D80-91C6-4C6C-B526-55E72AE9499F

http://species-id.net/wiki/Heterospilus_leioenopus

Figure 69

#### Female.

Body size: 2.5–3.5 mm. Color: head usually brown, vertex sometimes darker than face; scape honey yellow without lateral longitudinal brown stripe, flagellum entirely brown; mesosoma light brown to brown, often lighter along notauli; metasoma brown to light brown; wing veins brown, stigma bicolored brown with yellow at apex, base and along anterior edge; legs yellow. Head: vertex costate; frons costate; face smooth; temple in dorsal view narrow, width less than 1/2 eye width; malar space equal to eye height; ocell-ocular distance slightly greater than twice diameter of lateral ocellus; 21–26 flagellomeres. Mesosoma: mesoscutal lobes granulate; notauli scrobiculate, meeting at scutellum in triangular rugose area; scutellum granulate; prescutellar furrow with 3 cross carinae; mesopleuron granulate; precoxal sulcus weakly scrobiculate or smooth, shorter than mesopleuron; venter granulate; propodeum with basal median areas margined, granulate, basal median carina present, areola usually not distinctly margined, occasionally weakly margined apically, areolar area rugose, lateral areas entirely rugose. Wings: fore wing vein r slightly shorter than vein 3RSa, vein 1cu-a beyond vein 1M; hind wing vein SC+R present, vein M+CU shorter than vein 1M. Metasoma: first tergum longitudinally costate, apical width equal to length; second tergum longitudinally costate, width about 4 times length; anterior transverse groove present, sinuate; posterior transverse groove present; third tergum costate at base, smooth apically; terga 4-7 smooth; ovipositor equal in length to metasoma, rarely slightly longer.

#### Holotype female.

Top label (white, partially printed and hand written) - Costa Rica: Guanacaste [;] Santa Rosa Natl. Park [;] 300m, ex. Malaise trap [;] Site #: (blank) [;] Dates: 23.iii–13.iv.1986 [;] I.D. Gauld & D. Janzen; second label (white, printed) - [H] open regenerating [;] woodland <10 year old [;] [C] more or less fully [;] shaded as possible; third label (red, partially printed and hand written) - HOLOTYPE [;] Heterospilus [;] leioenopus [;] P. Marsh. Deposited in ESUW.

#### Paratypes.

6 ♀♀, same data as holotype with dates of 13.iv–4.v.1986, 21.ii–14.iii.1987, 18.i–8.ii.1986 and 26.vii–14.viii.1986, and second labels [H] open regenerating [;] woodland <10yr old [;] [O] in clearing, fully [;] isolated pert of day, [SE] Bosque San Emilio [;] 50yr old deciduous forest [;] [C] more or less fully [;] shaded as possible, and [SE] Bosque San Emilio [;] 0yr old deciduous forest [;] [O] in clearing, fully [;] isolated pert of day (ESUW). 2 ♀♀, COSTA RICA: [;] San Jose [;] Ciudad Colon, 800m [;] xii.1989-i.1990 [;] Luis Fournier (ESUW). 1 ♀, COSTA RICA: [;] San Jose [;] Ciudad Colon [;] 800m, iii-iv 1990 [;] Col. Luis Fournier (ESUW). 1 ♀, Costa Rica: Guanacaste [;] Est. Biol. Maritza, 600m [;] i.1997, C. Zuniga, Malaise [;] L.N. 326900-373000 #47557 (ESUW). 2 ♀♀, S.RosaPark, Guan. [;] C. Rica 3 Feb 78 and 24 Feb 78 [;] D.H. Janzen [;] Dry Hill (AEIC).

#### Comments.

The smooth face and the shorter metasomal tergum 1 are distinctive for this species.

#### Etymology.

The specific name is from the Greek *leios* meaning smooth and the Greek *enope* meaning face in reference to the smooth face.

**Figure 69 A-E.**
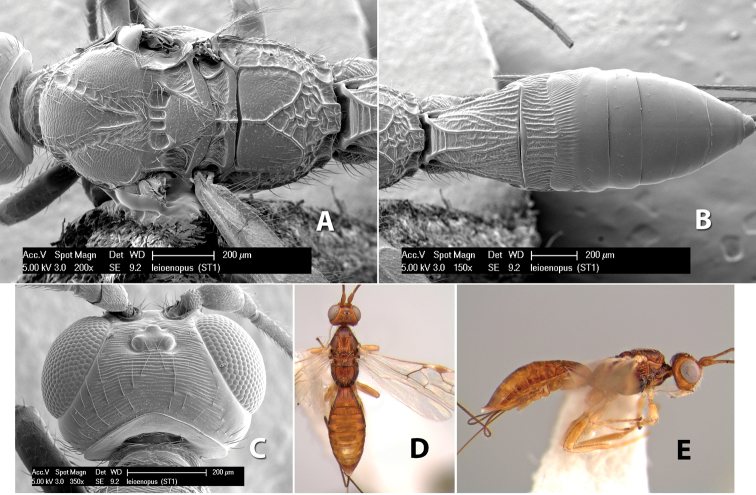
*Heterospilus leioenopus* Marsh, sp. n.: **A–C** paratype D**–E** holotype.

### Heterospilus levis

Marsh sp. n.

http://zoobank.org/8E3CFC0E-4741-40B7-8736-04AD48B28E51

http://species-id.net/wiki/Heterospilus_levis

Figure 70

#### Female.

Body size: 3.0 mm. Color: head brown; scape yellow, flagellum brown with apical 10 flagellomeres white; mesosoma dark brown; metasomal terga 1–5 dark brown, terga 6-7 yellow; wing veins including stigma brown; legs yellow. Head: vertex weakly striate behind ocelli, smooth near eyes; frons weakly striate; face smooth; temple in dorsal view narrow, width less than 1/2 eye width; malar space slightly greater than 1/4 eye height; ocell-ocular distance 2.5 times diameter of lateral ocellus; 28 flagellomeres. Mesosoma: mesoscutal lobes granulate; notauli scrobiculate, meeting at scutellum in triangular costate area; scutellum smooth; prescutellar furrow usually with 1 cross carina, sometimes with weak carinae on each side of median carina; mesopleuron granulate; precoxal sulcus smooth, shorter than mesopleuron; venter granulate; propodeum with basal median areas margined, granulate, basal median carina present, areola distinctly margined, areolar area rugose, lateral areas rugose apically, smooth basally. Wings: fore wing vein r as long as vein 3RSa, vein 1cu-a beyond vein 1M; hind wing vein SC+R present, vein M+CU shorter than vein 1M. Metasoma: first tergum longitudinally costate, apical width 1/2 length; second tergum smooth; anterior transverse groove present, straight; posterior transverse groove present; third tergum smooth; terga 4-7 smooth; ovipositor about 1/2 length of metasoma.

#### Holotype female.

Top label (white, printed) - COSTA RICA: Puntarenas [;] Reserva Forestal Golfo Dulce [;] 3km southwest of Rincon [;] 10m, July 1991. P. Hanson [;] primary forest, Malaise trap; second label (red, partially printed and hand written) - HOLOTYPE [;] Heterospilus [;] levis [;] P. Marsh. Deposited in ESUW.

#### Paratypes.

2 ♀♀, COSTA RICA: Puntar [;] Golfo Dulce, 3km [;] S.W. Rincon, 10m [;] IX-XI 1989, Hanson (ESUW). 1 ♀, COSTA RICA: Puntar [;] Golfo Dulce, 10km W [;] Piedras Blancas, 100m [;] VI-VIII 1989, Hanson (ESUW). 2 ♀♀, Costa Rica: Puntarenas, ACO [;] Golfito, P.N. Corcovado, 745m [;] Est. Agujas, Cerro Rincon [;] 15.v–15.vi.1999, J. Azofeifa [;] L.S. 276900-521500 #52744 [;] Malaise trap (ESUW). 1 ♀, Costa Rica: Heredia, ACCVC [;] Sarapiqui, Zona Prot. La Selva [;] 3km. S. de Sarapiqui, 50–100m [;] 18.iv.1988m, H. A. Hespenheide [;] manual (red, libre). #54835 [;] L.N. 268800-535300 (ESUW). 1 ♀, COSTA RICA: San Jose [;] P.N. Braulio Carillo [;] 9.5km E tunnel, 1000m [;] x-xii 1989, P. Hanson (ESUW).

#### Comments.

The smooth metasomal terga 2-7 and the narrow tergum 1 are distinctive for this species.

#### Etymology.

The specific name is from the Latin *levis* meaning smooth in reference to the smooth metasomal terga.

**Figure 70.**
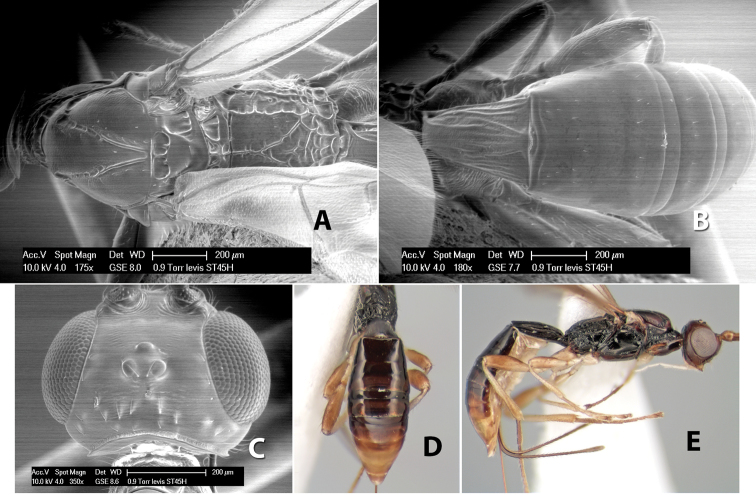
*Heterospilus levis* Marsh, sp. n., holotype.

### Heterospilus limonensis

Marsh sp. n.

http://zoobank.org/C4B80531-BE5C-4579-80B5-67F331A749C2

http://species-id.net/wiki/Heterospilus_limonensis

Figure 71

#### Female.

Body size: 3.0–4.0 mm. Color: vertex brown, frons, face, temple and eye orbits yellow or honey yellow; scape yellow without lateral longitudinal brown stripe, flagellum entirely brown; mesosoma dark brown, mesoscutal lobes and venter light brown; metasomal terga 1–2 and basal half of 3 dark brown, apical half of tergum 3 and terga 4–7 honey yellow, darker laterally; legs yellow; wing veins brown, stigma bicolored brown with yellow apex. Head: vertex transversely costate; frons transversely costate; face rugose or rugose-costate; temple in dorsal view narrow, less than 1/2 eye width; malar space greater than 1/4 eye height; ocell-ocular distance about twice diameter of lateral ocellus; 25–35 flagellomeres. Mesosoma: mesoscutal lobes granulate; notauli scrobiculate, meeting before scutellar furrow in triangular costate area; scutellum smooth; prescutellar furrow with one cross carina; mesopleuron smooth; precoxal sulcus smooth, shorter than length of mesopleuron; venter smooth; propodeum with basal median areas distinct and margined, basal median areas granulate, basal median carina distinct but short, areola not distinctly margined, areolar area rugose, lateral areas entirely rugose. Wings: fore wing vein r shorter than vein 3RSa, vein 1cu-a beyond vein 1M present; hind wing vein SC+R, vein M+CU shorter than vein 1M. Metasoma: first tergum length about equal to apical width, longitudinally costate; second tergum longitudinally costate, apical width less than 3 times length; anterior transverse groove distinct, straight; posterior transverse groove distinct; third tergum longitudinally costate at base, smooth at apex; terga 4–7 smooth, tergum 4 costate at base; ovipositor longer than metasoma.

#### Holotype female.

Top label (white, printed) - Costa Rica: Limon, ACLAC [;] Central, R. B. Hitoy Cerere [;] Est. H. Cerere, 100-140m. [;] Send. Toma de Agua, Malaise [;] 17.xi–17.xii.1999. F. Umana [;] L.N.184600-643400 #54940; second label (red, partially printed and hand written) - HOLOTYPE [;] Heterospilus [;] limonensis [;] P. Marsh. Deposited in ESUW.

#### Paratypes.

1 ♀, COSTA RICA: Limon [;] 4km NE Bribri [;] 50m. IX-XI 1989 [;] col. Paul Hanson (ESUW).

#### Comments.

This species is similar to *Heterospilus puntarensis* but differs in the single cross carina in the prescutellar furrow, the fourth metasomal tergum being mostly smooth and the bicolored mesosoma.

#### Etymology.

Named for the locality of the type specimens, Limón Province.

**Figure 71.**
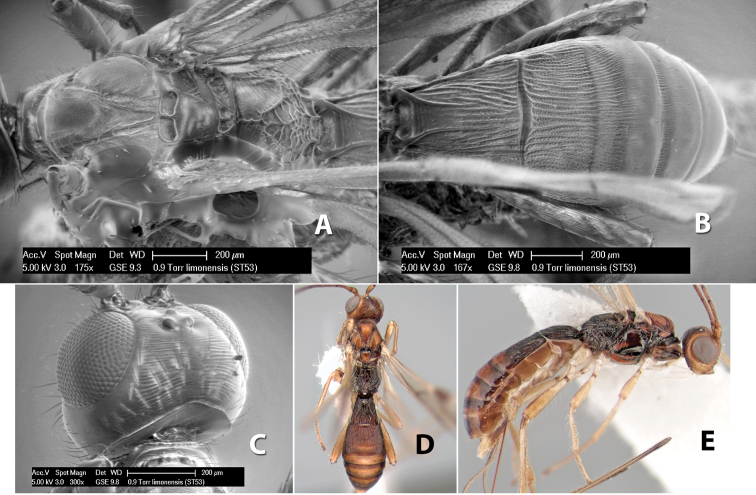
*Heterospilus limonensis* Marsh, sp. n.: **A–C E** holotype **D** paratype.

### Heterospilus macrocarinus

Marsh sp. n.

http://zoobank.org/A68AE5E6-0C17-4F25-86DA-E41B04053BD9

http://species-id.net/wiki/Heterospilus_macrocarinus

Figure 72

#### Female.

Body size: 3.0 mm. Color: head light brown or darker brown with face and eye orbits lighter; scape light brown without lateral longitudinal brown stripe, flagellum light brown basally to dark brown apically; mesosoma usually brown or dark brown, mesoscutum lighter brown, at least along notauli; metasomal tergum 1 dark brown, tergum 2 light brown medially, remainder of terga slightly lighter than tergum 1; legs yellow; wing veins including stigma brown. Head: vertex transversely striate; frons transversely striate; face weakly striate, often smooth near eyes; temple in dorsal view broad, slightly bulging behind eye, width slightly greater than 1/2 eye width; malar space greater than 1/4 eye height; ocell-ocular distance about 2.5 times diameter of lateral ocellus; 24–30 flagellomeres. Mesosoma: mesoscutal lobes granulate; notauli scrobiculate, meeting at scutellum in triangular rugose area; scutellum smooth; prescutellar furrow with 1 cross carina; mesopleuron smooth; precoxal sulcus smooth, shorter than mesopleuron; venter smooth; propodeum with basal median areas large, square, distinctly margined, rugose, basal median carina distinct, as long as half dorsal length of propodeum, areola not margined, areolar area rugose, lateral areas entirely rugose. Wings: fore wing vein r shorter than vein 3RSa, vein 1cu-a beyond vein 1M; hind wing vein SC+R present, vein M+CU shorter than vein 1M. Metasoma: first tergum longitudinally costate, apical width about equal to length; second tergum longitudinally costate; anterior transverse groove present, straight; posterior transverse groove present; third tergum smooth or weakly costate medially at base, smooth apically; terga 4–7 smooth; ovipositor as long as metasoma.

#### Holotype female.

Top label (white, partially printed and hand written) - Costa Rica: Guanacaste [;] Santa Rosa Natl. Park [;] 300m, ex. Malaise trap [;] Site #: BH-12-C [;] Dates: 29.xi–20.xii.1986 [;] I.D. Gauld & D. Janzen; second label (white, printed) - [BH] Bosque Humedo [;] mature evergreen dry forest [;] [C] more or less fully [;] shaded as possible; third label (red, partially printed and hand written) - HOLOTYPE [;] Heterospilus [;] macrocarinus [;] P. Marsh. Deposited in ESUW.

#### Paratypes.

1 ♀, Costa Rica: Puntarenas [;] R.F. Golfo Dulce, [;] 3km. SW. Rincon, 10m [;] iii.1993 Paul Hanson coll. [;] Malaise, primary forest (ESUW).

#### Comments.

This species is distinguished by the unusually long basal median carina on the propodeum.

#### Etymology.

The specific name is from the Greek *makros* meaning long in reference to the long basal median carina of the propodeum.

**Figure 72.**
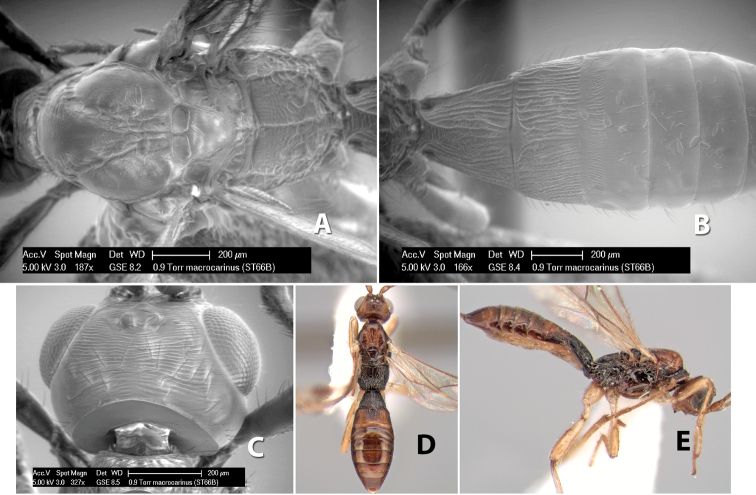
*Heterospilus macrocarinus* Marsh, sp. n.: **A–C, E** holotype **D** paratype.

### Heterospilus magnus

Marsh sp. n.

http://zoobank.org/FDBF0305-1F79-4F97-9F42-79641EBD789D

http://species-id.net/wiki/Heterospilus_magnus

Figure 73

#### Female.

Body size: 3.5–4.0 mm. Color: head with vertex usually light brown, face yellow; scape yellow without lateral brown stripe, flagellomeres brown; mesosoma dark brown; metasomal terga 1–4 dark brown to brown, terga 5–7 yellow, tergum 7 brown at apex; wing veins including stigma brown; legs yellow. Head: vertex weakly striate or occasionally smooth; frons weakly striate; face striate-granulate; temple in dorsal view narrow, width less than 1/2 eye width; malar space 1/6–1/7 eye height; ocell-ocular distance about equal to diameter of lateral ocellus; 22–26 flagellomeres. Mesosoma: mesoscutal lobes granulate; notauli scrobiculate, meeting at scutellum in triangular rugose area; scutellum granulate; prescutellar furrow usually with 1 distinct cross carina, sometimes with weak carinae on each side of median carina; mesopleuron granulate; precoxal sulcus scrobiculate, shorter than mesopleuron; venter granulate; propodeum with basal median areas margined, granulate, basal median carina absent, areola not distinctly margined, areolar area rugose, lateral areas entirely rugose. Wings: fore wing vein r shorter than vein 3RSa, vein 1cu-a beyond vein 1M; hind wing vein SC+R present, vein M+CU shorter than vein 1M. Metasoma: first tergum longitudinally costate, apical width less than length; second tergum longitudinally costate, narrow with apical width about 4 times median length; anterior transverse groove present, straight; posterior transverse groove present; third tergum costate basally, smooth apically; terga 4-7 smooth; ovipositor equal to length of metasomal 1.

#### Holotype female.

Top label (white, printed) - COSTA RICA-Heredia Prov. [;] La Selva Biological Station [;] 10°26'N, 84°01'W, 100m [;] Canopy fogging 31 [;] 2.xi.1994 [;] Project ALAS (FPM31); second label (red, partially printed and hand written) - HOLOTYPE [;] Heterospilus [;] magnus [;] P. Marsh. Deposited in ESUW.

#### Paratypes.

3 ♀♀, same data as holotype with dates of 3.xi.1994, 2.xi.1994 and 24.x.1994 (ESUW). 1 ♀, Costa Rica: Puntarenas [;] Golfo Dulce, 24km W. [;] Piedras Blancas, 200m [;] iv.1993, Paul Hanson (ESUW).

#### Comments.

The large eyes and small malar space are distinctive for this species.

#### Etymology.

The specific name is from the Latin *magnus*, meaning large, in reference to the large eyes.

**Figure 73.**
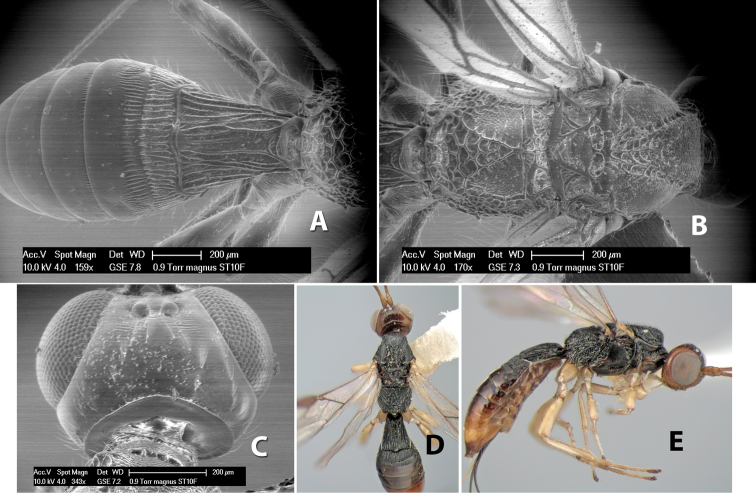
*Heterospilus magnus* Marsh, sp. n., holotype.

### Heterospilus maritzaensis

Marsh sp. n.

http://zoobank.org/9E797931-BE18-4C6E-8E65-8CC710F3A2AA

http://species-id.net/wiki/Heterospilus_maritzaensis

Figure 74

#### Female.

Body size: 3.5 mm. Color: head dark brown with yellow eye orbits and yellow spot on malar space; scape yellow without lateral brown stripe, flagellum brown; mesosoma dark brown; metasoma dark brown; wing veins including stigma brown; legs yellow. Head: vertex transversely costate; frons transversely costate; face areolate-rugose; temple in dorsal view narrow, sloping behind eye; malar space equal to 1/4 eye height; ocell-ocular distance about twice diameter of lateral ocellus; 24 flagellomeres. Mesosoma: mesoscutal lobes granulate, rugose along notauli, median lobe with median longitudinal raised ridge anteriorly; notauli scrobiculate, meeting at scutellum in triangular costate-rugose area; scutellum granulate; prescutellar furrow with 3 cross carinae; mesopleuron granulate; precoxal sulcus scrobiculate, shorter than mesopleuron; venter granulate; propodeum with basal median areas not distinctly margined, rugose, basal median carina absent, areola not distinctly margined, areolar area areolate-rugose, lateral areas entirely rugose. Wings: fore wing vein r shorter than vein 3RSa, vein 1cu-a beyond vein 1M; hind wing vein SC+R present, vein M+CU equal to vein 1M. Metasoma: first tergum longitudinally costate, length slightly greater than apical width; second tergum longitudinally costate; anterior transverse groove weak or absent; posterior transverse groove weak or absent; third tergum costate basally, smooth apically; terga 4-7 smooth; ovipositor equal to length of metasomal tergum 1.

#### Holotype female.

Top Label (white, printed) - Costa Rica: Guanacaste [;] Est. Biol. Maritza, 600m [;] i.1997, C. Zuniga, Malaise [;] L.N. 326900-373000 347557; second label (red, partially printed and hand written) - HOLOTYPE [;] Heterospilus [;] maritzaensis [;] P. Marsh. Deposited in ESUW.

#### Paratypes.

Known only from the holotype.

#### Comments.

The raised ridge on the median mesoscutal lobe and the yellow spot on the malar space are distinctive for this species.

#### Etymology.

Named for the type locality, the Maritza Biological Station in Guanacaste National Park.

**Figure 74.**
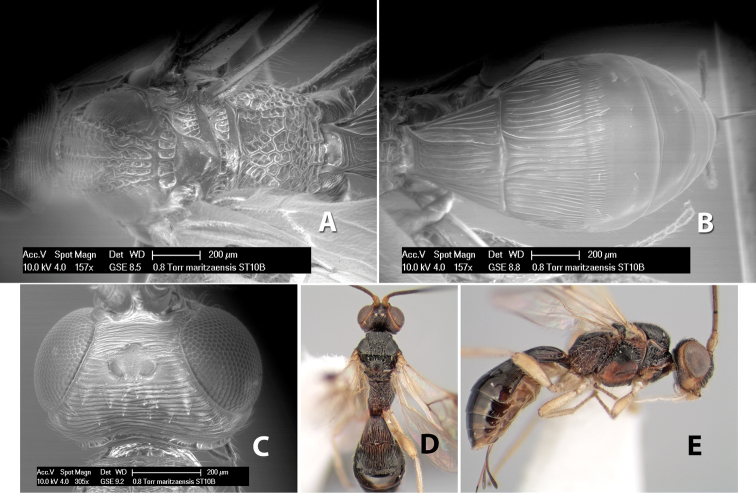
*Heterospilus maritzaensis* Marsh, sp. n., holotype.

### Heterospilus masoni

Marsh sp. n.

http://zoobank.org/4709FE39-A0F7-4F79-A8AC-16B120374C3B

http://species-id.net/wiki/Heterospilus_masoni

Figure 75

#### Female.

Body size: 2.5–3.5 mm. Color: head brown to dark brown; scape yellow without lateral brown stripe, flagellum brown; mesosoma dark brown, propodeum often lighter brown; metasomal tergum 1 dark brown, terga 2–4 yellow medially, dark brown laterally, terga 5–7 yellow; wing veins brown, stigma yellow; legs yellow. Head: vertex transversely striate; frons weakly transversely striate; face smooth; temple in dorsal view narrow, sloping behind eye, width equal to 1/2 eye width; malar space greater than 1/4 eye height; ocell-ocular distance 2.5 or more times diameter of lateral ocellus; 19–20 flagellomeres. Mesosoma: mesoscutal lobes granulate; notauli scrobiculate, meeting at scutellum in triangular costate-rugose area; scutellum granulate; prescutellar furrow with 3 cross carinae; mesopleuron granulate; precoxal sulcus smooth, shorter than mesopleuron; venter smooth; propodeum with basal median areas margined, granulate, basal median carina absent or rarely extremely short, areola not distinctly margined, areolar area rugose, lateral areas entirely rugose. Wings: fore wing vein r slightly shorter than vein 3RSa, vein 1cu-a beyond vein 1M; hind wing vein SC+R present, vein M+CU shorter than vein 1M. Metasoma: first tergum longitudinally costate, apical width as least 4 times length; second tergum longitudinally costate; anterior transverse groove present, straight; posterior transverse groove present; third tergum costate basally, smooth apically; terga 4-7 smooth; ovipositor equal to length of metasoma.

#### Holotype female.

Top label (white, partially printed and hand written) - Costa Rica: Guanacaste [;] Santa Rosa Natl. Park [;] 300m, ex. Malaise trap [;] Site #: SE-7-O [;] Dates: 4–24.v.1986 [;] I.D. Gauld & D. Janzen; second label (white, printed) - [SE] Bosque San Emilio [;] 50yr old deciduous forest [;] [O] in clearing, fully [;] isolated part of day; third label (red, partially printed and hand written) - HOLOTYPE [;] Heterospilus [;] masoni [;] P. Marsh. Deposited in ESUW.

#### Paratypes.

3 ♀♀, same data as holotype except: Site #: 10, dates of 13.ix–4.x.1986, 8–9.xi.1986, and second label [BH] Bosque Humedo [;] mature evergreen dry forest [;] [C] more or lass fully [;] shaded as possible (ESUW). 1 ♀, top label, Costa Rica: Guanacaste [;] Santa Rosa National Pk. [;] 300m, Malaise, Ian Gauld [;] 14.vi–5.vii.1986, second label, SE-7-O [;] 14.vi–5.vii.86, third label, Bosque San Emilio [;] 50yr Old deciduous [;] forest SUN (ESUW).

#### Comments.

The narrow metasomal tergum 2, light brown metasomal terga and the yellow tegula are distinctive for this species.

#### Etymology.

Named for the late Canadian braconidologist, W. R. M. (Bill) Mason in recognition of his numerous and important contributions to our knowledge of the Braconidae.

**Figure 75.**
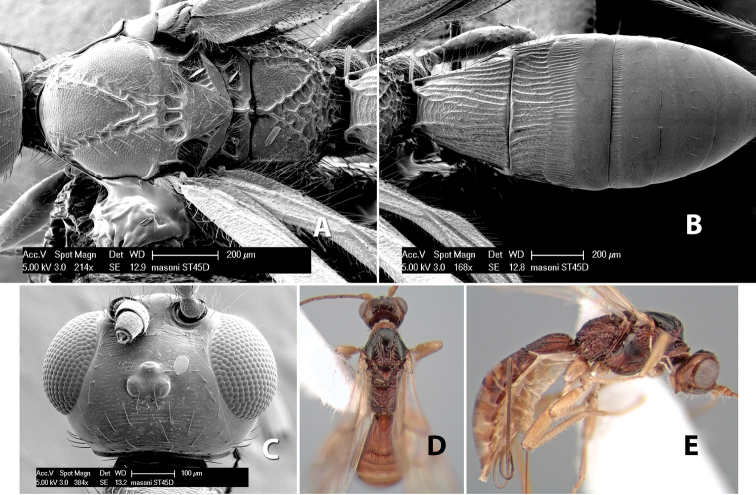
*Heterospilus masoni* Marsh, sp. n.: **A–C** paratype **D–E** holotype.

### Heterospilus mellosus

Marsh sp. n.

http://zoobank.org/7604DDB6-3057-4550-98C5-63E622442CC8

http://species-id.net/wiki/Heterospilus_mellosus

Figure 76

#### Female.

Body size: 2.0 mm. Color: head honey yellow or light brown; scape yellow without lateral longitudinal brown stripe, flagellum yellow basally to brown apically; mesosoma and metasoma honey yellow; wing veins brown, stigma yellow; legs yellow. Head: vertex weakly striate, often smooth near eyes; frons weakly striate or nearly smooth; face smooth; temple in dorsal view slightly bulging behind eye, width equal to 1/2 eye width; malar space greater than eye height; ocell-ocular distance about 2.5 times diameter of lateral ocellus; 18–19 flagellomeres. Mesosoma: mesoscutal lobes granulate; notauli scrobiculate, meeting at scutellum in triangular costate area; scutellum granulate; prescutellar furrow with 3 cross carinae; mesopleuron granulate, nearly smooth just above precoxal sulcus; precoxal sulcus smooth, shorter than mesopleuron; venter smooth; propodeum with basal median areas margined, granulate, basal median carina present, areola not distinctly margined, areolar area rugose, lateral areas entirely rugose. Wings: fore wing vein r shorter than vein 3RSa, vein 1cu-a beyond vein 1M; hind wing vein SC+R present, vein M+CU slightly shorter than vein 1M. Metasoma: first tergum longitudinally costate, apical width equal to length; second tergum longitudinally costate, greatest width about 4 times median length; anterior transverse groove weakly present and straight or partially absent; posterior transverse groove weakly present or partially absent; third tergum entirely smooth; terga 4-7 smooth; ovipositor longer than metasoma.

#### Holotype female.

Top label (white, partially printed and hand written) - Costa Rica: Guanacaste [;] Santa Rosa Natl. Park [;] 300m, ex. Malaise trap [;] Site #: (blank) [;] Dates: 31-I-21-II.1987 [;] I.D. Gauld & D. Janzen; second label (white, printed) - [SE] Bosque San Emilio [;] 50yr old deciduous forest [;] [C] more or less fully [;] shaded as possible; third label (red, partially printed and hand written) - HOLOTYPE [;] Heterospilus [;] mellosus [;] P. Marsh. Deposited in ESUW.

#### Paratypes.

1 ♀, same data as holotype, with date of 8-29.xi.1986, and second label as [BH] Bosque Humedo [;] mature evergreen dry forest [;] [C] more or less fully [;] shaded as possible (ESUW). 1 ♀, S.RosaPark, Guan. [;] C. Rica 8 Mar. 77 [;] D. H. Janzen [;] Dry Hill (AEIC).

#### Comments.

The yellow body color and the short metasomal tergum 2 are distinctive for this species.

#### Etymology.

The specific name is from the Latin *mellosus* meaning honey-colored in reference to the honey colored body.

**Figure 76.**
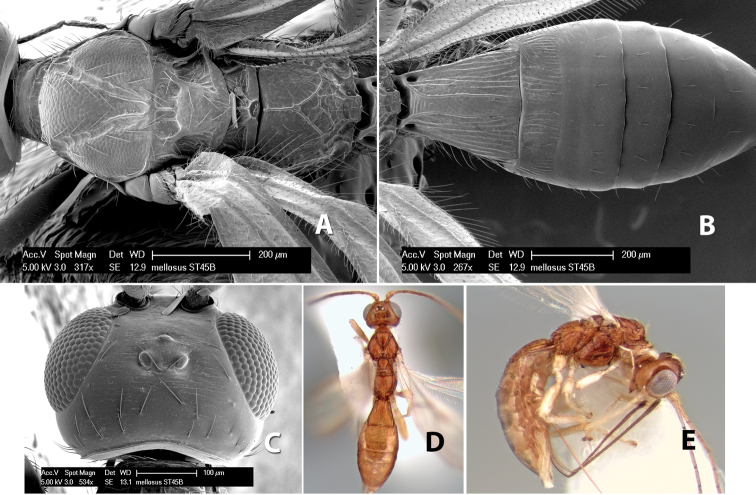
*Heterospilus mellosus* Marsh, sp. n.: **A–D** paratype **E** holotype.

### Heterospilus menkei

Marsh sp. n.

http://zoobank.org/D141884C-A4B5-4DD8-BF62-38C2EB2D4FD1

http://species-id.net/wiki/Heterospilus_menkei

Figure 77

#### Female.

Body size: 4.0 mm. Color: head dark brown to brown; scape honey yellow without lateral longitudinal brown stripe, flagellum brown with apical 8-10 flagellomeres white; mesosoma dark brown; metasoma dark brown, terga 5-7 yellow; legs yellow, hind tibia at extreme base and hind tarsus brown; wing veins including stigma brown. Head: vertex transversely costate; frons transversely costate; face smooth; temple in dorsal view narrow, not bulging behind eye, less than 1/2 eye width; malar space greater than 1/4 eye height; ocell-ocular distance about twice diameter of lateral ocellus; 29-35 flagellomeres. Mesosoma: mesoscutal lobes smooth, rugose along notauli; notauli scrobiculate, meeting at scutellum in triangular costate area; scutellum smooth; prescutellar furrow with 3 cross carinae; mesopleuron smooth; precoxal sulcus smooth, shorter than width of mesopleuron; venter smooth; propodeum with basal median areas distinct but not margined, basal median carina absent, areola not distinctly margined, areolar area areolate-rugose, lateral areas entirely rugose. Wings: fore wing vein r shorter than vein 3RSa, vein 1cu-a beyond vein 1M; hind wing vein SC+R present, vein M+CU shorter than vein 1M. Metasoma: first tergum longitudinally costate or porcate, length slightly greater than apical width; second tergum longitudinally costate or porcate, width about 4 times median length; anterior transverse groove present, straight; posterior transverse groove present; third tergum costate at base, smooth apically; terga 4-7 smooth; ovipositor longer than metasoma.

#### Holotype female.

Top label (white, printed) - COSTA RICA: Puntarenas [;] R.F. Golfo Dulce, [;] 24km W. Piedras Blancas [;] 200m [;] Feb. 1992, Paul Hanson; second label (red, partially printed and hand written) - HOLOTYPE [;] Heterospilus [;] menkei [;] P. Marsh. Deposited in ESUW.

#### Paratypes.

1 ♀, top label - COSTA RICA, Heredia [;] Est. Biol. La Selva, 50- [;] 150m, 10°26'N, 84°01'W [;] May 1993, INBio-OET; second label - 18 Mayo 1993 [;] Bosque secundario [;] M/04/099; third label - INBio bar code (ESUW).

#### Comments.

This species is distinguished by the mesoscutal lobes being rugose along the notauli and is similar to *Heterospilus milleri* but is separated by its smooth mesoscutal lobes rather than granulate in *Heterospilus milleri*.

#### Etymology.

Named for my longtime friend, colleague and college classmate Arnold Menke.

**Figure 77.**
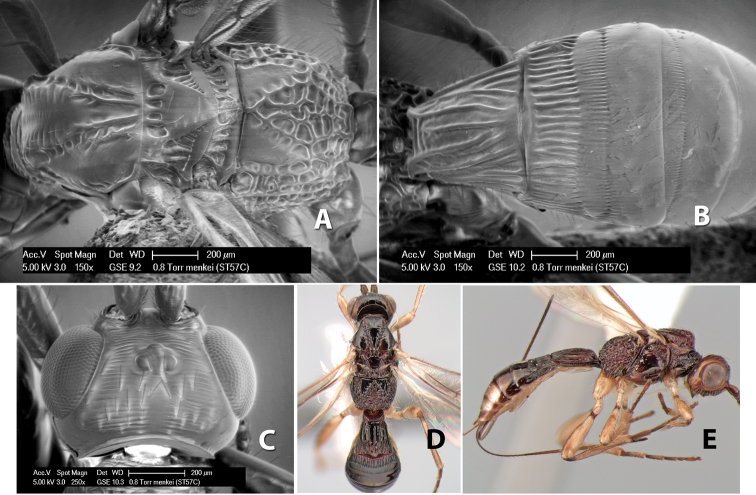
*Heterospilus menkei* Marsh, sp. n.: **A–D** holotype **E** paratype.

### Heterospilus milleri

Marsh sp. n.

http://zoobank.org/72000EF0-A7D2-4A4B-BCE1-8C7B23475671

http://species-id.net/wiki/Heterospilus_milleri

Figure 78

#### Female.

Body size: 3.5 mm. Color: head dark to light brown; scape yellow with lateral longitudinal brown stripe, flagellum brown with apical 8–10 flagellomeres white; mesosoma dark brown; metasoma dark brown, terga 5–7 yellow; legs yellow, hind tibia at extreme base and hind tarsus brown; wing veins including stigma brown. Head: vertex transversely costate, occasionally weakly so or smooth; frons transversely costate; face smooth; temple in dorsal view broad but not bulging, about equal to eye width; malar space 1/2 eye height; ocell-ocular distance slightly greater than twice diameter of lateral ocellus; 25–32 flagellomeres. Mesosoma: mesoscutal lobes granulate, weakly rugose along notauli; notauli scrobiculate, meeting at scutellum in triangular costate-rugose area; scutellum smooth; prescutellar furrow with 3 cross carinae; mesopleuron smooth; precoxal sulcus smooth, shorter than width of mesopleuron; venter smooth; propodeum with basal median areas smooth and indistinctly margined, basal median carina absent, areola not distinctly margined, areolar area areolate-rugose, lateral areas entirely rugose. Wings: fore wing vein r shorter than vein 3RSa, vein 1cu-a beyond vein 1M; hind wing vein SC+R present, vein M+CU shorter than vein 1M. Metasoma: first tergum longitudinally costate or porcate, slightly longer than apical width; second tergum costate, about 3.5 times wider than long; anterior transverse groove present, straight; posterior transverse groove present; third tergum costate at base, smooth at apex; terga 4–7 smooth; ovipositor longer or slightly shorter than metasoma.

#### Holotype female.

Top label (white, printed) - COSTA RICA: Puntarenas [;] Golfo Dulce, [;] 15km W. Piedras Blancas, [;] 100m. [;] XI.1990, Paul Hanson; second label (red, partially printed and hand written) - HOLOTYPE [;] Heterospilus [;] milleri [;] P. Marsh. Deposited in ESUW.

#### Paratypes.

1 ♀, top label - Costa Rica: Guanacaste [;] Santa Rosa Natl. Park [;] 300m, ex. Malaise trap [;] Site #: (blank) [;] Dates: 26.x–16.xi.1986 [;] I.D. Gauld & D. Janzen; second label - [BH] Bosque Humedo [;] mature evergreen dry forest [;] [C] more or less fully [;] shaded as possible (ESUW). 1 ♀, Costa Rica: Guanacaste [;] Est. Maritza, 600m [;] Malaise, ix.1996. C. Zuniga [;] L.N. 326900-373000- #47558 (ESUW). 1 ♀, COSTA RICA-Heredia Prov. [;] La Selva Biological Station [;] 10°26'N, 84°01'W, 100m [;] Canopy fogging 32 [;] 3.xi.1994 [;] Project ALAS (FVK32) (ESUW).

#### Comments.

This species is similar to *Heterospilus menkei* but is distinguished by its granulate mesoscutal lobes.

#### Etymology.

Named for my long time friend and research colleague at the USDA, Douglass Miller.

**Figure 78.**
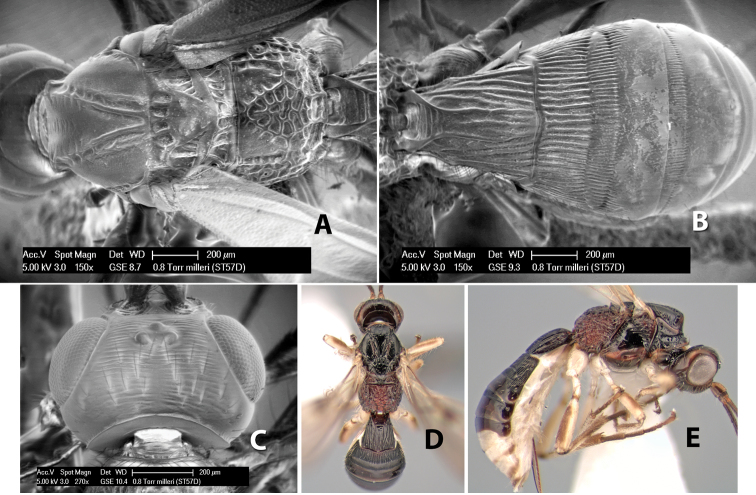
*Heterospilus milleri*, Marsh, sp. n., holotype.

### Heterospilus miskito

Marsh sp. n.

http://zoobank.org/8CDD0355-246B-4D63-BB97-7771D0D72B6C

http://species-id.net/wiki/Heterospilus_miskito

Figure 79

#### Female.

Body size: 3.0–3.5 mm. Color: head honey yellow; scape yellow without lateral brown stripe, flagellum entirely brown; mesosoma brown with mesoscutum and lower portion of mesopleuron lighter brown to honey yellow; metasomal terga 1–4 brown to dark brown, terga 5–7 yellow; wing veins including stigma brown; legs yellow. Head: vertex transversely costate; frons transversely costate; face rugose; temple in dorsal view narrow, width less than 1/2 eye width; malar space greater than 1/4 eye height; ocell-ocular distance about 2.5 times diameter of lateral ocellus; 26–27 flagellomeres. Mesosoma: mesoscutal lobes granulate; notauli scrobiculate, meeting scutellum in triangular rugose area; scutellum granulate; prescutellar furrow with 3 cross carinae; mesopleuron granulate; precoxal sulcus smooth, shorter than mesopleuron; venter granulate; propodeum with basal median areas margined, granulate, basal median carina absent, areola not distinctly margined, areolar area rugose, lateral areas rugose posteriorly, granulate anteriorly. Wings: fore wing vein r shorter than vein 3RSa, vein 1cu-a beyond vein 1M; hind wing vein SC+R present, vein M+CU shorter than vein 1M. Metasoma: first tergum longitudinally costate, apical width about equal to length; second tergum longitudinally costate-granulate; anterior transverse groove present, straight; posterior transverse groove absent; third tergum weakly costate basally, granulate apically; terga 4-5 granulate; ovipositor as long as metasoma.

#### Holotype female.

Top label (white, printed) - COSTA RICA-Heredia Prov. [;] La Selva Biological Station [;] 10°26'N, 84°01'W, 100m [;] Malaise trap 14, #260 [;] 1.xi.1993 [;] Project ALAS (M.14.260); second label (red, partially printed and hand written) - HOLOTYPE [;] Heterospilus [;] miskito [;] P. Marsh. Deposited in ESUW.

#### Paratypes.

1 ♀, Costa Rica: Guanacaste [;] P. N. Guanacaste [;] below Pitilia, 500m [;] 7–8.iii.1990, J. S. Noyes (ESUW). 1 ♀, top label - COSTA RICA, Heredia: [;] Est. Biol. La Selva, 50- [;] 150m, 10°26'N, 84°01'W [;] Mar 1996, INBio-OET; second label - 15 Marzo 1996 [;] Bosque secundario [;] M/11/600 (INBC).

#### Comments.

The evenly brown mesoscutum and the absence of the basal median carina of the propodeum are distinctive for this species.

#### Etymology.

The specific name refers to the Miskito, an indigenous people of Honduras and Nicaragua.

**Figure 79.**
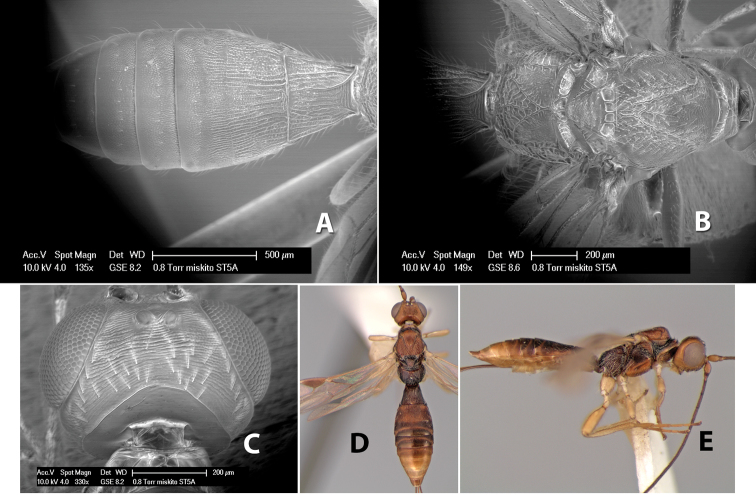
*Heterospilus miskito* Marsh, sp. n., holotype.

### Heterospilus mixtec

Marsh sp. n.

http://zoobank.org/CC84D897-7D02-4653-A8D4-9BAA383A8450

http://species-id.net/wiki/Heterospilus_mixtec

Figure 80

#### Female.

Body size: 2.5 mm. Color: head with vertex and frons brown, face and eye orbits lighter; scape yellow with lateral longitudinal brown stripe; flagellum brown with apical 5–6 flagellomeres white except apical flagellomere brown; mesosoma dark brown; metasomal terga 1–3 dark brown, remainder of terga slightly lighter; wing veins brown, stigma brown with yellow at base; legs yellow. Head: vertex weakly striate behind ocelli, smooth near eyes; frons weakly striate; face granulate; temple in dorsal view narrow, width less than 1/2 eye width; malar space greater than 1/4 eye height; ocell-ocular distance 2.5 times or greater than diameter of lateral ocellus; 20 flagellomeres. Mesosoma: mesoscutal lobes granulate; notauli scrobiculate, meeting at scutellum in triangular rugose area; scutellum granulate; prescutellar furrow with 3 cross carinae; mesopleuron granulate; precoxal sulcus scrobiculate, shorter than mesopleuron; venter granulate; propodeum with basal median areas margined, granulate, basal median carina present, short, areola usually not distinctly margined, areolar area rugose, lateral areas rugose posteriorly, granulate anteriorly. Wings: fore wing vein r shorter than vein 3RSa, vein 1cu-a beyond vein 1M; hind wing vein SC+R present, vein M+CU shorter than vein 1M. Metasoma: first tergum longitudinally costate, apical width about equal to length; second tergum longitudinally costate; anterior transverse groove present, straight; posterior transverse groove present; third tergum costate basally, smooth apically; terga 4–7 smooth; ovipositor longer than metasoma.

#### Holotype female.

Top label (white, printed) - Costa Rica, Heredia [;] 3km. S. Puerto Viejo [;] OTS-La Selva, 100m [;] IV-V-1993, P. Hanson; second label (red, partially printed and hand written) - HOLOTYPE [;] Heterospilus [;] mixtec [;] P. Marsh. Deposited in ESUW.

#### Paratypes.

1 ♀, COSTA RICA-Heredia Prov. [;] La Selva Biological Station [;] 10°26'N, 84°01'W, 100m [;] Malaise trap 05, #296 [;] 15.xii.1993 [;] Project ALAS (m.05.296) (ESUW). 1 ♀, COSTA RICA, Heredia [;] Chilamate, 75m [;] VII-VIII/1989 [;] col. Paul Hanson (ESUW). 1 ♀, top label - COSTA RICA, Heredia [;] Est. Biol. La Selva, 50- [;] 150m, 10°26'N, 84°01'W [;] Jun 1993 INBio-OET; second label - 14 Juno 1993 [;] Bosque secundario [;] M/13/135 (ESUW). 1 ♀, Costa Rica: Guanacaste [;] P.N. Guanacaste [;] below Pitilia, 500m [;] 7–8.iii.1990, J. S. Noyes (ESUW). 1 ♀, Costa Rica: Limon, Central [;] R.B. Hitoy Cerere, Est. Hitoy [;] Cerere, Send. Toma de Agua [;] 100–140m, Malaise trap [;] 11.x–11.xi.1992, F. Umana [;] L.N.184600-643400 #54013 (ESUW). 1 ♀, Costa Rica, Limon [;] Sector Cocori, 100m [;] 30km N Cariari, i.1995 [;] E. Rojas, Malaise #4526 [;] L.N. 286000-567500 (ESUW).

#### Comments.

The smaller eyes and ocelli and the white apical flagellomeres are distinctive for this species.

#### Etymology.

Named for the Mixtec, an indigenous people of Mexico.

**Figure 80.**
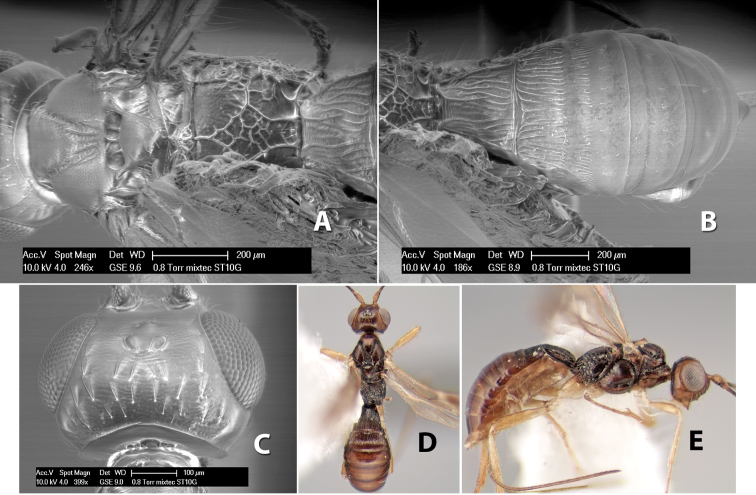
*Heterospilus mixtec* Marsh, sp. n., holotype.

### Heterospilus monteverde

Marsh sp. n.

http://zoobank.org/84BA2890-9AB1-4FAC-B6DB-B7595BFEC808

http://species-id.net/wiki/Heterospilus_monteverde

Figure 81

#### Female.

Body size: 3.5 mm. Color: head brown; scape honey yellow with lateral longitudinal brown stripe, flagellum brown with apical 5–8 flagellomeres white; mesosoma dark brown, mesoscutal lobes honey yellow anteriorly, propleuron and venter honey yellow; metasomal terga 1–4 dark brown, tergum slightly lighter, tergum 5 brown basally, yellow apically, terga 6–7 yellow basally, light brown apically; wing veins including stigma brown; legs yellow, hind femur yellow on basal 1/4, brown on apical 3/4. Head: vertex transversely costate; frons transversely costate; face smooth; temple in dorsal view narrow, less than 1/2 eye width; malar space greater than 1/4 eye height; ocell-ocular distance about 2.5 times diameter of lateral ocellus; 24–26 flagellomeres. Mesosoma: mesoscutal lobes granulate; notauli scrobiculate, meeting at scutellum in triangular costate area; scutellum granulate; prescutellar furrow with 1 median distinct cross carina plus 2 weaker carinae on each side; mesopleuron granulate; precoxal sulcus smooth, shorter than mesopleuron; venter granulate; propodeum with basal median areas margined, granulate, basal median carina present, areola not distinctly margined, areolar area rugose, lateral areas rugose posteriorly, granulate anteriorly. Wings: fore wing vein r shorter than vein 3RSa, vein 1cu-a beyond vein 1M; hind wing vein SC+R present, vein M+CU shorter than vein 1M. Metasoma: first tergum longitudinally costate-granulate; second tergum costate-granulate; anterior transverse groove present, sinuate; posterior transverse groove absent; third tergum entirely smooth; terga 4–7 smooth; ovipositor as long as metasoma.

#### Holotype female.

Top label (white, printed) - COSTA RICA: [;] Puntarenas [;] Monteverde, [;] 1400m, [;] 26–28.iii.1991, yellow [;] pan, Col. Hanson; second label (red, partially printed and hand written) - HOLOTYPE [;] Heterospilus [;] monteverde [;] P. Marsh. Deposited in ESUW.

#### Paratypes.

Known only from the holotype.

#### Comments.

The granulate mesopleuron, slightly sinuate anterior transverse groove on metasomal tergum 2 and the weak or absent posterior transverse groove of metasomal tergum 3.

#### Etymology.

The specific name is from the type locality, Monteverde in Puntarenas Province.

**Figure 81.**
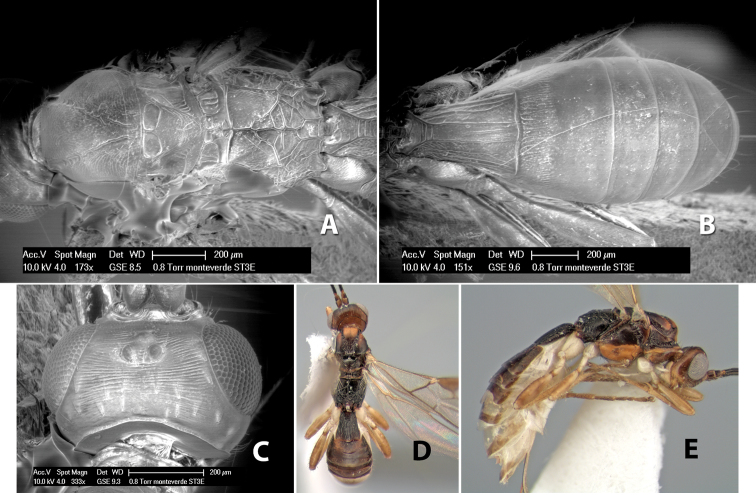
*Heterospilus monteverde* Marsh, sp. n., holotype.

### Heterospilus muertensis

Marsh sp. n.

http://zoobank.org/8E6A4D63-6B1F-498C-8E5B-5CFF975DD052

http://species-id.net/wiki/Heterospilus_muertensis

Figure 82

#### Female.

Body size: 3.0–4.0 mm. Color: head yellow; scape yellow without lateral longitudinal brown stripe, flagellum yellow basally to brown apically; mesosoma light brown, propleuron, pronotum, precoxal sulcus and propodeum laterally often darker brown; metasoma yellow or honey yellow, tergum 1 may be darker brown; legs yellow, extreme base of tibia often brown; wing veins brown, stigma somewhat bicolored, yellow basally and light brown apically. Head: vertex transversely striate-granulate; frons striate; face smooth; temple in dorsal view broad and bulging behind eye, width greater than 1/2 eye width; malar space greater than 1/4 eye height; ocell-ocular distance slightly more than 2.5 times diameter of lateral ocellus; 20-25 flagellomeres. Mesosoma: mesoscutal lobes granulate, median lobe with median groove from middle to prescutellar furrow; notauli scrobiculate, meeting at scutellum in triangular rugose area, notauli bordered by dense long yellow setae; scutellum smooth; prescutellar furrow with 3-5 cross carinae; mesopleuron smooth; precoxal sulcus smooth, shorter than mesopleuron; venter smooth; propodeum with basal median areas distinctly margined, weakly granulate or nearly smooth, basal median carina distinct and long, areola distinctly margined, areolar area rugose, lateral areas entirely rugose. Wings: fore wing vein r shorter than vein 3RSa, both vein at nearly right angled with each other, vein 1cu-a beyond vein 1M; hind wing vein SC+R present, vein M+CU slightly shorter than vein 1M. Metasoma: first tergum longitudinally costate, length greater than apical width; second tergum longitudinally costate, apical width less than 3 times length; anterior transverse groove straight, weak or nearly absent; posterior transverse groove weak or nearly absent; third tergum entirely smooth; terga 4-7 smooth; ovipositor equal to length of metasoma.

#### Holotype female.

Top label (white, printed) - COSTA RICA San Jose [;] Cerro de la Muerte, 19km [;] S, 3 W, Empalme, 2600m [;] ii-iii.1993. Paul Hanson; second label (red, partially printed and hand written) - HOLOTYPE [;] Heterospilus [;] muertensis [;] P. Marsh. Deposited in ESUW.

#### Paratypes.

1 ♀, Costa Rica: San Jose [;] Cerro de la Muerte [;] 6km. N. San Gerado [;] 2800m, November 1993 [;] P. Hanson, Malaise (ESUW). 1 ♀, Costa Rica: San Jose [;] Cerro de la Muerte [;] 19km S 3 W Empalme [;] 2600m, November 1992 [;] P. Hanson, Malaise (ESUW).

#### Comments.

This species is distinguished by the broad temple, long yellow setae along the notauli, and the generally yellow body color.

#### Etymology.

Named for Cerro de la Muerte where the type series was collected.

**Figure 82.**
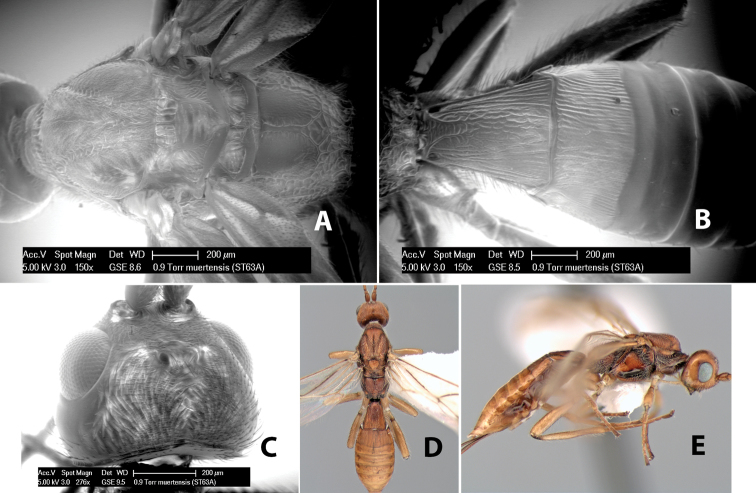
*Heterospilus muertensis* Marsh, sp. n., holotype.

### Heterospilus muesebecki

Marsh sp. n.

http://zoobank.org/8C4A8C3F-76A1-4DAD-807C-514AED1FF999

http://species-id.net/wiki/Heterospilus_muesebecki

Figure 83

#### Female.

Body size: 2.5–3.0 mm. Color: head yellow; scape yellow without lateral brown stripe, flagellum yellow basally to brown apically; mesosoma brown to dark brown; metasoma brown, terga 1 and 2 often lighter brown; wing veins including stigma brown; legs yellow. Head: vertex transversely costate; frons transversely costate; face rugose; temple in dorsal view narrow, strongly sloping behind eye, width less than 1/2 eye width; malar space equal to 1/4 eye height; ocell-ocular distance usually about 1.5 times diameter of lateral ocellus, rarely about twice diameter; 18–22 flagellomeres. Mesosoma: mesoscutal lobes coarsely granulate, median lobe often with median scrobiculate line; notauli strongly scrobiculate, meeting at scutellum in triangular rugose area; scutellum granulate; prescutellar furrow usually with 3 cross carinae, rarely with 1 distinct median carina plus 2 weaker carinae on each side; mesopleuron granulate; precoxal sulcus scrobiculate, shorter than mesopleuron but often with a carina extending from sulcus to posterior edge of mesopleuron; venter granulate; propodeum with basal median areas margined, granulate, basal median carina absent, areola not distinctly margined, areolar area rugose, lateral areas entirely rugose. Wings: fore wing vein r as long as or slightly shorter than vein 3RSa, vein 1cu-a beyond vein 1M; hind wing vein SC+R absent, vein M+CU shorter than vein 1M. Metasoma: first tergum longitudinally costate with median raised area rugose between distinct carinae, apical width equal to or slightly less than length; second tergum longitudinally costate; anterior transverse groove present, straight; posterior transverse groove present, often weakly so; third tergum costate basally, smooth apically; terga 4–7 smooth; ovipositor equal to combined length of metasomal terga 1–2.

#### Holotype female.

Top label (white, partially printed and hand written) - Costa Rica: Guanacaste [;] Santa Rosa Natl. Park [;] 300m, ex. Malaise trap [;] Site #: BH-10-C [;] Dates: 7–28.xii.1985 [;] I.D. Gauld & D. Janzen; second label (white, printed) - [BH] Bosque Humedo [;] mature evergreen dry forest [;] [C] more or less fully [;] shaded as possible; third label (red, partially printed and hand written) - HOLOTYPE [;] Heterospilus [;] muesebecki [;] P. Marsh. Deposited in ESUW.

#### Paratypes.

1 ♀, same data as holotype (ESUW). 2 ♀♀, top label - Costa Rica: Guanacaste [;] Santa Rosa Natl. Park [;] 300m, ex. Malaise trap [;] Site #: SE-7 and 5-O [;] Dates: 6–27.ix.1986 and 23.iii–13.iv.1986 [;] I.D. Gauld & D. Janzen; second label - [SE] Bosque San Emelio [;] 50yr old deciduous forest [;] [O] in clearing, fully [;] isolated part of day (ESUW). 3 ♀♀, top label - Costa Rica: Guanacaste [;] Santa Rosa Natl. Park [;] 300m, ex. Malaise trap [;] Site #: SE-6-C and blank [;] Dates: 7-28.xii.1985, 4–24.v.1986 and 3–24.viii.1985 [;] I.D. Gauld & D. Janzen; second label - [SE] Bosque San Emelio [;] 50yr old deciduous forest [;] [C] more or less fully [;] shaded as possible (ESUW). 3 ♀♀, top label - Costa Rica: Guanacaste [;] Santa Rosa Natl. Park [;] 300m, ex. Malaise trap [;] Site #: BH-10 and 12-C and blank [;] Dates: 24.v–14vi.1986 and 18.i–8.ii.1986 [;] I.D. Gauld & D. Janzen; second label - [BH] Bosque Humedo [;] mature evergreen dry forest [;] [C] more or less fully [;] shaded as possible (ESUW). 2 ♀♀, top label - Costa Rica: Guanacaste [;] Santa Rosa Natl. Park [;] 300m, ex. Malaise trap [;] Site #: BH-9-O and blank [;] Dates: 20.xi.86–10.i.1097 and 29.xi–20.xii.1986 [;] I.D. Gauld & D. Janzen; second label - [BH] Bosque Humedo [;] mature evergreen dry forest [;] [O] in clearing, fully [;] isolated part of day (ESUW). 2 ♀♀, top label - Costa Rica: Guanacaste [;] Santa Rosa Natl. Park [;] 300m, ex. Malaise trap [;] Site #: H-1-O and blank [;] Dates: 10–31.i.1987 and 20.xii.86–10.i.1987 [;] I.D. Gauld & D. Janzen; second label - [H] open regenerating [;] woodlands <10 year old [;] [O] in clearing, fully [;] isolated part of day (ESUW). 1 ♀, top label - Costa Rica: BH-10-C [;] Guanacaste Province [;] Santa Rosa Natl. Pk. [;] 300m, (dry season) [;] 10-31 January 1987; second label - Bosque Humedo, mature [;] dry forest with high [;] proportion evergreen [;] species, fully shaded [;] Townes style Malaise [;] Ian Gauld coll. (ESUW). 4 ♀♀, Costa Rica: Guanacaste [;] PN Guanacaste, 7km E HQ [;] near “small house” [;] 9.iii.1990, J. S. Noyes (ESUW). 3 ♀♀, top label - Costa Rica: Guanacaste Pr. [;] Guanacaste National Park [;] near Playa Naranja [;] 11 March 1990, J.S. Noyes; second label - PSYL#04 (ESUW). 2 ♀♀, top label - Costa Rica: Guanacaste [;] Santa Rosa National Pk. [;] 300m, Malaise, Ian Gauld [;] 10–31.i.1987; second label - mature dry forest [;] high proportion [;] evergreen species [;] Sun; third label - BH-11-O [;] 10-31.i.87 (ESUW). 1 ♀, Costa Rica: Guanacaste, ACT [;] Bagaces, P.N. Palo Verde [;] Sec. P. Verde, 0–50m [;] 2–12.xii.1999, I. Jimenez [;] L.N. 260932-385020 #54246 [;] Red de Golpe (ESUW). 1 ♀, Costa Rica: Guanacaste, ACT [;] Bagaces, P.N. Palo Verde, 212m [;] Sec. Palo Verde, Cerro Guayacan [;] 13.ix–13.x.1999, I. Jimenez, Malaise [;] L.N. 259350-389600 #53499 (ESUW). 1 ♀, Costa Rica: Puntarenas [;] Peninsula Osa [;] Puerto Jimenez, 10m [;] i–ii.1992, Paul Hanson [;] grassy, weedy site (ESUW). 1 ♀, Costa Rica, Puntarenas [;] Pen. Osa, Puerto Jimenez, [;] 10m. VIII-IX-1993 [;] P. Hanson (ESUW). 1 ♀, Costa Rica: Puntarenas [;] San Vito, Las Cruces [;] Wilson Botanical Gardens [;] 18–22.iii.1990, 1150m [;] J.S. Noyes (ESUW). 1 ♀, Costa Rica: Cartago [;] Braulio Carillo N.P. [;] 600m, 25.iii.1990 [;] J. S. Noyes, coll. (ESUW). 5 ♀♀, COSTA RICA: Puntarenas [;] RF Golfo Dulce 200m [;] 24km W Piedras Blancas [;] P. Hanson ix.1992 and vi.1993 (TAMU). 27 ♀♀, S.RosaPark, Guan. [;] C. Rica, various dates from June 1976 to January 1978 [;] D. H. Janzen [;] Riparian and Dry Hill (AEIC). 2 ♀♀, Turrialba, C.R. [;] IV-25-1957 [;] RDShenefelt [;] RDS 57-177 (AEIC). 1 ♀, COSTA RICA: *Punt-* [;] *arenas*. 7km SW Rincon [;] 31.v–7.vi.1998. B. Brown [;] & V. Berezovskiy, Mal. [;] Trp. #1; 2nd growth (AEIC).

#### Comments.

The large eyes and ocelli, strongly sculptured mesoscutum and the raised median area on metasomal tergum 1 are distinctive for this species.

#### Etymology.

Named for the great North American hymenopterist, Carl F. W. Muesebeck, in recognition for the mentoring he gave me in my early days as a budding braconidologist in Washington.

**Figure 83.**
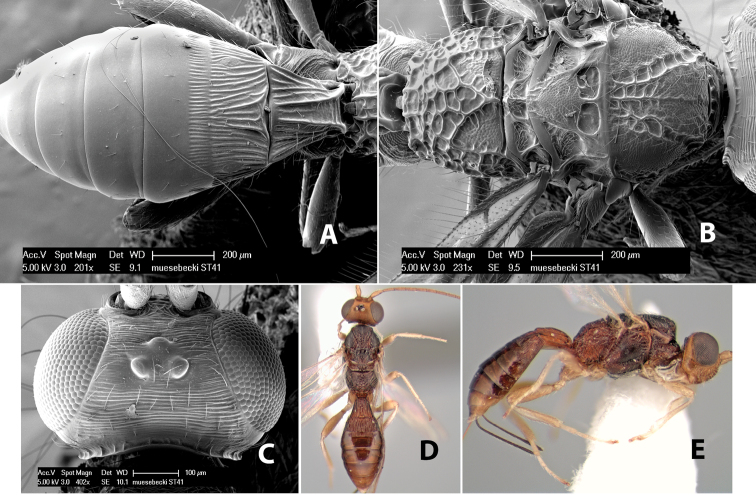
*Heterospilus muesebecki* Marsh, sp. n.: **A–C, E** paratype **D** holotype.

### Heterospilus neesi

Marsh sp. n.

http://zoobank.org/C3192579-28C3-4B57-A4DE-72D5804904B1

http://species-id.net/wiki/Heterospilus_neesi

Figure 84

#### Female.

Body size: 3.0–3.5 mm. Color: head with vertex and frons brown, face and eye orbits light brown or yellow; scape yellow, flagellum brown; mesosoma dark brown, often with lower mesopleuron lighter; metasomal tergum 1 dark brown, tergum 2 dark brown with lateral converging yellow stripes, terga 3–4 dark brown, yellow medially, terga 5-7 lighter brown than tergum 1; legs yellow, apical half of hind femur and tibia brown; wing veins including stigma brown. Head: vertex weakly striate; frons smooth or very weakly striate; face smooth except for weak striae below antennae; temple in dorsal view narrow, sloping behind eye, less than 1/2 eye width; malar space greater than 1/4 eye height; ocell-ocular distance slightly less than 2.5 times diameter of lateral ocellus; 24–30 flagellomeres. Mesosoma: mesoscutal lobes granulate; notauli scrobiculate, meeting posteriorly in wide rectangular rugose area; scutellum smooth; prescutellar furrow with 3–5 cross carinae; mesopleuron smooth; precoxal sulcus scrobiculate, shorter than mesopleuron; venter weakly granulate; propodeum with basal median areas not distinctly margined, areolate-rugose, basal median carina absent, areola not distinctly margined, areolar area areolate-rugose, lateral areas entirely rugose. Wings: fore wing vein r shorter than vein 3RSa, vein 1cu-a beyond vein 1M; hind wing vein SC+R present, vein M+CU shorter than vein 1M. Metasoma: first tergum costate-granulate; second tergum costate-granulate; anterior transverse groove present, straight; posterior transverse groove present; third tergum costate basally, smooth apically; terga 4-7 smooth; ovipositor equal to length of terga 1+2.

#### Holotype female.

Top label (white, partially printed and hand written) - Costa Rica: Guanacaste [;] Santa Rosa Natl. Park [;] 300m, ex. Malaise trap [;] Site #: (blank) [;] Dates: 24.v–14.vi.1986 [;] I.D. Gauld & D. Janzen; second label (white, printed) - [BH] Bosque Humedo [;] mature evergreen dry forest [;] [C] more or less fully [;] shaded as possible; third label (red, partially printed and hand written) - HOLOTYPE [;] Heterospilus [;] neesi [;] P. Marsh. Deposited in ESUW.

#### Paratypes.

2 ♀♀, Costa Rica: Guanacaste, ACT [;] Bagaces, P.N. Palo Verde [;] Sec. P. Verde, 150 de la Est. [;] 0-50m, 17.viii–13.ix.1999 [;] I. Jimenez, Malaise #53257 [;] L.N. 260952-385020 (ESUW).

#### Comments.

This species is distinguished by the rectangular rugose area where the notauli meet posteriorly and the yellow markings on metasomal tergum 2.

#### Etymology.

Named for C. G. Ness ab Esenbeck who was one of the earliest workers on Hymenoptera in the early 1880s.

**Figure 84.**
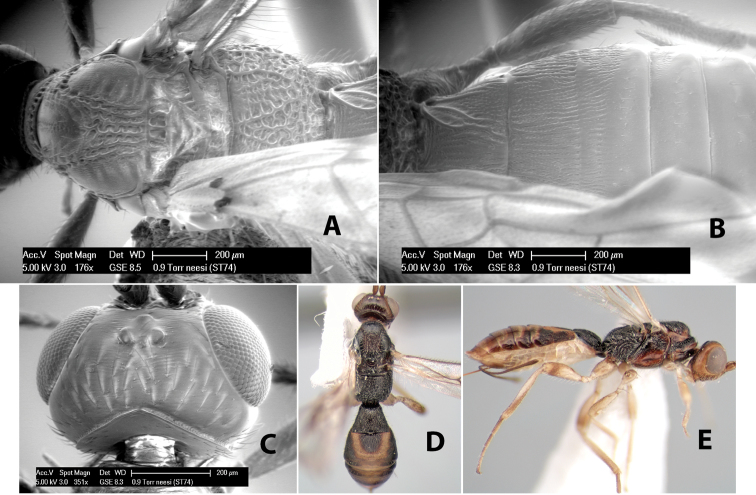
*Heterospilus neesi* Marsh, sp. n.: **A–C, E** holotype **D** paratype.

### Heterospilus nephus

Marsh sp. n.

http://zoobank.org/1CD14F70-9640-4D2D-81CB-DED9F5D83FDB

http://species-id.net/wiki/Heterospilus_nephus

Figure 85

#### Female.

Body size: 4.5–5.0 mm. Color: head entirely yellow; scape yellow without lateral longitudinal brown stripe, flagellum brown; mesosoma bicolored, mesoscutum, mesopleuron on lower half, venter and upper and lower margin of pronotum yellow, propleuron brown, pronotum medially, subalar area of mesopleuron, metanotum and propodeum dark brown; metasomal tergum 1 dark brown, tergum 2 dark brown medially, yellow laterally, tergum 3 dark brown basally, brown apically, yellow laterally, terga 4–6 dark brown basally, yellow apically and laterally, tergum 7 yellow; wing veins including stigma brown; legs yellow. Head: vertex transversely costate; frons transversely costate; face weakly granulate, often partially smooth; temple in dorsal view narrow, width less than 1/2 eye width; malar space greater than 1/4 eye height; ocell-ocular distance 1.5 times diameter of lateral ocellus; 32-35 flagellomeres. Mesosoma: mesoscutal lobes granulate; notauli scrobiculate, meeting at scutellum in triangular rugose area; scutellum granulate; prescutellar furrow with 3 cross carinae; mesopleuron granulate; precoxal sulcus weakly scrobiculate, rarely smooth, shorter than mesopleuron; venter granulate; propodeum with basal median areas margined, granulate, basal median carina absent, areola not distinctly margined, areolar area rugose, lateral areas rugose posteriorly, granulate anteriorly. Wings: fore wing vein r shorter than vein 3RSa, vein 1cu-a beyond vein 1M; hind wing vein SC+R present, vein M+CU shorter than vein 1M. Metasoma: first tergum longitudinally costate-granulate, apical width equal to length; second tergum longitudinally costate-granulate, width nearly 4 times length; anterior transverse groove present, sinuate; posterior transverse groove weakly indicated or absent; third tergum costate at base, granulate at apex; terga 4–7 granulate at base, smooth at apex; ovipositor longer than metasoma.

#### Holotype female.

Top label (white, printed) - COSTA RICA-Heredia Prov. [;] La Selva Biological Station [;] 10°26'N, 84°01'W, 100m [;] Canopy fogging 32 [;] 3.xi.1994 [;] Project ALAS(FVK32); second label (red, partially printed and hand written) - HOLOTYPE [;] Heterospilus [;] nephus [;] P. Marsh. Deposited in ESUW.

#### Paratypes.

2 ♀♀, same data as holotype (ESUW). 2 ♀♀, same data as holotype with dates of 19.x.1994, 11.xi.1994 and Canopy fogging 26, 35 (ESUW). 1 ♀, same locality data as holotype, Malaise trap 09, #327 [;] 15.i.1994 [;] Project ALAS(M.09.327) (ESUW). 1 ♀, S.RosaPark,Guan, Guan. [;] C. Rica 20 Aug 77 [;] D.H. Janzen [;] Dry Hill (AEIC). 1 ♀, COSTA RICA. Prov. Limón. R.B. [;] Hitoy Cerere, Send. Espavel, 560m, 15 [;] MAR 2003, B. Gamboa, Red de Golpe. [;] L.S. 401200 569800 #73280 (INBC).

#### Comments.

The yellow mesoscutum and the granulate base of metasomal terga 4–6 are distinctive for this species. This species is similar to *Heterospilus variegatus* Ashmead from St. Vincent, West Indies, but the ovipositor is longer than the metasoma in *Heterospilus nephus* but shorter than the metasoma in *Heterospilus variegatus*.

#### Etymology.

The specific name is from the Greek *Heterospilus nephos*, meaning cloud, in reference to the type series being collected by canopy fogging at the La Selva Biological Station.

**Figure 85.**
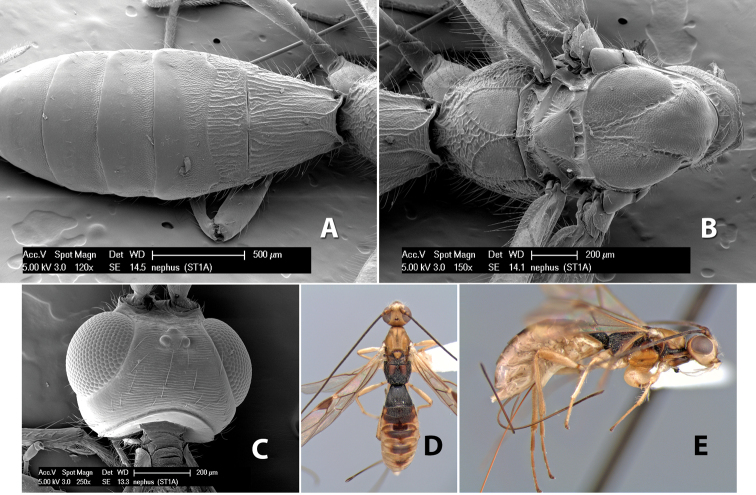
*Heterospilus nephus* Marsh, sp. n.: A–C paratype D–E holotype.

### Heterospilus nigragonatus

Marsh sp. n.

http://zoobank.org/61B209F9-69F0-4AF3-893D-F430D1F73683

http://species-id.net/wiki/Heterospilus_nigragonatus

Figure 86

#### Female.

Body size: 3.0–4.0 mm. Color: head yellow or honey yellow, temple just behind eye often brown; scape honey yellow without lateral longitudinal brown stripe, flagellum brown with apical 5–7 flagellomeres white; mesosoma dark brown, yellow along notauli, median mesoscutal lobe often light brown, mesopleuron along precoxal sulcus often light brown; metasomal tergum 1 dark brown, tergum 2 usually dark brown, often lighter, terga 3–7 dark brown basally, yellow apically; wing veins brown, stigma usually brown, often yellow at apex; legs yellow, hind tibia dark brown at base. Head: vertex transversely costate; frons transversely costate; face weakly striate or granulate; temple in dorsal view narrow, sloping behind eye, width less than 1/2 eye width; malar space greater than 1/4 eye height; ocell-ocular distance about twice diameter of lateral ocellus; 28–32 flagellomeres. Mesosoma: mesoscutal lobes granulate; notauli scrobiculate, meeting at scutellum in triangular rugose area; scutellum granulate; prescutellar furrow usually with 3 cross carinae, rarely with 1 distinct median carina and 2 weaker carinae on each side; mesopleuron granulate; precoxal sulcus weakly scrobiculate or smooth, shorter than mesopleuron; venter granulate; propodeum with basal median areas margined, granulate, basal median carina present, areola distinctly margined, areolar area rugose, lateral areas rugose posteriorly, granulate anteriorly. Wings: fore wing vein r shorter than vein 3RSa, vein 1cu-a beyond vein 1M; hind wing vein SC+R present, vein M+CU shorter than vein 1M. Metasoma: first tergum longitudinally costate, apical width less than length; second tergum longitudinally costate, nearly 4 times as wide as long; anterior transverse groove present, sinuate; posterior transverse groove weakly indicated; third tergum entirely granulate; terga 4–7 granulate; ovipositor as long as metasoma.

#### Holotype female.

Top label (white, printed) - Costa Rica: Heredia [;] Puerto Viejo [;] OTS, La Selva, 100m [;] iv.1991, P. Hanson; second label (red, partially printed and hand written) - HOLOTYPE [;] Heterospilus [;] nigragonatus [;] P. Marsh. Deposited in ESUW.

#### Paratypes.

2 ♀♀, top label - COSTA RICA, Heredia [;] Est. Biol. La Selva, 50- [;] 150m, 10°26'N, 84°01W [;] Sep 1993, INBio-CET; second labels - 01 Setiembre 1993 [;] M/03/194 [;] Bosque primario and 16 Setiembre 1993 [;] Bosque secundario [;] M/09/215 (ESUW). 1 ♀, top label - COSTA RICA, Heredia [;] Est. Biol. La Selva, 50- [;] 150m, 10°26'N, 84°01W [;] Feb 1994, INBio-CET; second label - 1 Febrero 1994 [;] Bosque secundario [;] M/09/339 (ESUW). 1 ♀, top label - COSTA RICA, Heredia [;] Est. Biol. La Selva, 50- [;] 150m, 10°26'N, 84°01W [;] Jun 1993, INBio-CET; second label - 14 Junio 1993 [;] Bosque primario [;] M/03/126 (ESUW). 1 ♀, Costa Rica: Alajuela [;] R. B. A. Brenes [;] San Ramon, 900m [;] ii–iii.2000, P. Hanson (ESUW). 1 ♀, Costa Rica: Puntarenas [;] R.F. Golfo Dulce, [;] 3km. SW. Rincon, 10m, [;] iii.1993 Paul Hanson coll. [;] Malaise, primary forest (ESUW). 1 ♀, COSTA RICA: Puntar [;] Golfo Dulce 3km SW [;] Rincon [;] 10m, vii-ix 1990 [;] Col. Paul Hanson (ESUW). 2 ♀♀, COSTA RICA: Limon [;] 16km West Guapiles [;] 400m, April 1989 and i–iv.1991 [;] P. Hanson (ESUW). 1 ♀, Costa Rica: Guanacaste [;] Est. Biol. Maritza, 600m [;] i.1997, C. Zuniga, Malaise [;] L.N. 326900-373000 #47557 (ESUW). 1 ♀, top label - Costa Rica: Guanacaste [;] W. side Volcan Orosi [;] Estac. Maritza, 600m; second label - GNP Biodiversity Survey [;] 1989, Malaise trap [;] L-N-326900-373000 #6834 (ESUW). 1 ♀, Costa Rica: Guanacaste [;] Est. Pitilia, 700m [;] 9km S Santa Cecilia [;] Malaise Sobre Tanque de Agua [;] viii.1995, C. Moraga, P. Rios [;] L>N> 329950-480450 #8542 {ESUW). 1 ♀, Costa Rica: Guanacaste [;] P. N. Guanacaste [;] below Pitilia, 500m [;] 7–8.iii.1990, J. S. Noyes (ESUW).

#### Comments.

This species is distinguished by the granulate metasomal terga, the narrow second tergum and the dark brown knee of the hind tibia.

#### Etymology.

The specific name is from the Latin *nigra* meaning black and the Greek *gonatus* meaning knee in reference to the yellow hind tibia which is black at the joint with the femur.

**Figure 86.**
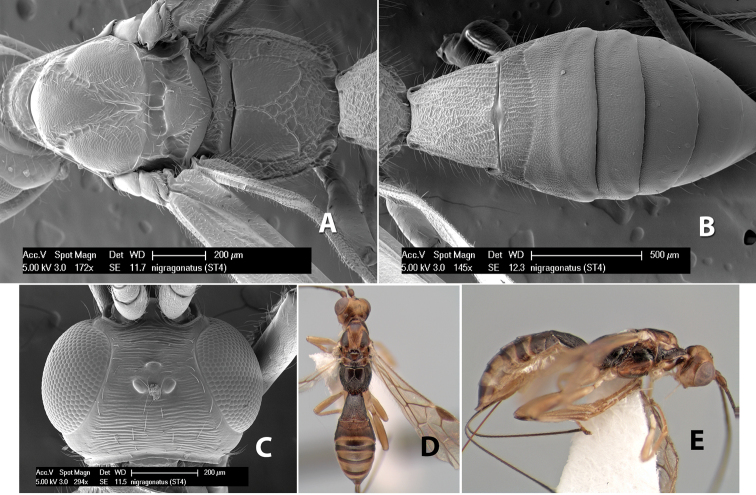
*Heterospilus nigragonatus* Marsh, sp. n.: **A–C** paratype **D–E** holotype.

### Heterospilus nigrescens

Ashmead

http://species-id.net/wiki/Heterospilus_nigrescens

Figure 87

Heterospilus nigrescens Ashmead, 1894: 120.

#### Female.

Body size: 3.0–3.5 mm. Color: entire body dark brown; scape yellow without lateral brown stripe, flagellum brown; wing veins including stigma brown; legs yellow. Head: vertex weakly striate behind ocelli, smooth near eyes; frons usually striate, sometimes weakly so and nearly smooth; face smooth, often weakly striate below antennae; temple in dorsal view broad, width slightly less than 1/2 eye width; malar space about equal to 1/4 eye height; ocell-ocular distance 2.5 or more times diameter of lateral ocellus; 21–25 flagellomeres. Mesosoma: mesoscutal lobes granulate; notauli scrobiculate, meeting at scutellum in triangular rugose area; scutellum weakly granulate to nearly smooth; prescutellar furrow with 3 cross carinae; mesopleuron granulate; precoxal sulcus weakly scrobiculate to nearly smooth, shorter than mesopleuron; venter granulate; propodeum with basal median areas margined, granulate, basal median carina present, areola not distinctly margined, areolar area rugose, lateral areas entirely rugose. Wings: fore wing vein r shorter than vein 3RSa, vein 1cu-a beyond vein 1M; hind wing vein SC+R present, vein M+CU shorter than vein 1M. Metasoma: first tergum longitudinally costate, apical width equal to or slightly shorter than length; second tergum longitudinally costate; anterior transverse groove present, straight; posterior transverse groove present; third tergum mostly longitudinally costate, smooth at extreme apex; terga 4-5 longitudinally costate on basal 1/2 to 3/4, terga 6–7 smooth; ovipositor as long as metasoma.

#### Distribution.

St. Vincent, West Indies and Costa Rica.

#### Specimens examined

. Holotype female: St. Vincent, W. I., H. H. Smith (NMNH). 1 ♀, Costa Rica: Guanacaste, ACT [;] Bagaces, P.N. Palo Verde [;] Sect. Catalina, 0–50m, do Luz [;] 8-12.xi.1999, I. Jimenez [;] L.N. 260952-85020 #53252 (ESUW). 1 ♀, Costa Rica: Puntarenas, ACT [;] Golfito, R.F. Golfo Dulce [;] Est. Agujas, 250–350m [;] 3–24.vii.1999, J. Azofeifa [;] L.S.276750-526550 #52840 [;] Red de Golpe (ESUW).

#### Comments.

The costate base of metasomal terga 4-5 is distinctive for this species.

**Figure 87.**
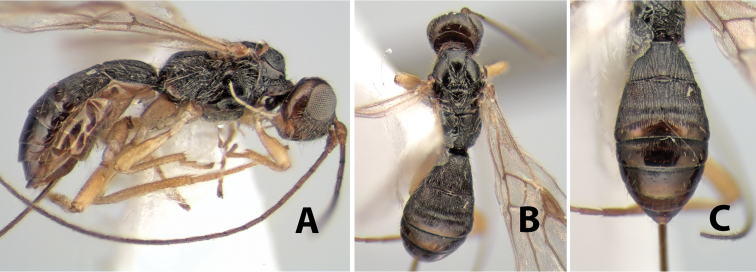
*Heterospilus nigrescens* Ashmead.

### Heterospilus nigricoxus

Marsh sp. n.

http://zoobank.org/2D6068C3-3E58-4785-B9AA-E4E5D1CFFF06

http://species-id.net/wiki/Heterospilus_nigricoxus

Figures 88 , 89

#### Female.

Body size: 2.5–3.5 mm. Color: head brown to honey yellow; scape and pedicel yellow, flagellum yellow basally to brown apically; mesosoma brown to dark brown or black, propodeum often lighter brown; metasoma brown to dark brown, apical terga often lighter than basal terga; wing veins brown, stigma bicolored, brown medially, yellow at base and apex; legs yellow, hind coxa and femur brown, hind tibia often light brown basally. Head: vertex transversely costate-rugose, frons rugose, face areolate or areolate-rugose; temple in dorsal view less than half eye width; malar space about half eye height; ocell-ocular distance about twice diameter of lateral ocellus; 18–26 flagellomeres. Mesosoma: mesoscutal lobes granulate, rugose or costate along notauli, long gold setae along notauli; notauli scrobiculate, meeting at prescutellar furrow in wide, rectangular rugose area; mesopleuron smooth above precoxal sulcus, rugose-costate dorsally; precoxal sulcus scrobiculate, about half width of mesopleuron, often with costae extending from precoxal sulcus to posterior edge of mesopleuron; scutellum smooth, prescutellar furrow with 3–5 cross carinae; venter of mesosoma smooth or partially weakly granulate; propodeum with basal median areas distinct and margined, basal median areas granulate or rugose-granulate, basal median carina absent, areola meeting anterior margin of propodeum, areola margined at least apically, areola and propodeum laterally areolate-rugose. Wings: fore wing vein r 2/3 length of vein 3RSa, vein 1cu-a distinctly beyond vein 1M; hind wing vein SC+R present, vein M+CU shorter than vein 1M. Metasoma: first tergum length equal to apical width, longitudinally costate, granulate between costae, raised basal median area distinctly margined laterally and often with cross carinae near base; second tergum apical width less than 3 times length, longitudinally costate, granulate between costae; anterior and posterior transverse grooves weakly indicated; third tergum longitudinally costate between transverse grooves, smooth beyond posterior transverse groove; terga 4–7 smooth; ovipositor longer than metasoma, often nearly as long as entire body.

#### Holotype female.

Top label (white, partially printed and hand written) - Costa Rica: Guanacaste [;] Santa Rosa Natl. Park [;] 300m, ex. Malaise trap [;] Site #: SE-8-C [;] Dates: 2–23.iii.1986 [;] I. D. Gauld & D. Janzen; second label (white, printed) - [SE] Bosque San Emilio [;] 50yr old deciduous forest [;] [C] more or less fully [;] shaded as possible; third label (red, partially printed and hand written) - HOLOTYPE [;] Heterospilus [;] nigricoxus [;] P. Marsh. Deposited in ESUW.

#### Paratypes.

1 ♀, same data as holotype (ESUW). 6 ♀♀, same data as holotype except site numbers of 6, SE.6.2, SE.7.0 and dates of 5–26.v.1985, 31.i–21-ii.1987, 2–23.iii.1986, 20.xii–10.i.1986/7, 14.vi–5.vii.1986, 10–31.i.1987 (ESUW). 2 ♀♀, same data as holotype except site number of 4, date of 10–31.i.1987 and second label [H] open regenerating [;] woodland <10 year old [;] [C] more or less fully [;] shaded as possible (ESUW). 6 ♀♀, same data as holotype except site numbers of H-3-0, H-2-0, dates of 10–31.i.1987, 18.i.1986, 31.i–21.ii.1987, 21.ii–14.iii.1987, 20.xii.1986–10.i.1987 and second label [H] open regenerating [;] woodland <10 years old [;] [O] in clearing, fully [;] isolated part of day. 1 ♀, same data as holotype except date of 20.xii.1986–10.i.1987 and second label [BH] Bosque Humedo [;] mature evergreen dry forest [;] [C] more or less fully [;] shaded as possible (ESUW). 4 ♀♀, same data as holotype except site number of SE.7.0, dates of 13.ix–4.x.1986, 10–31.i.1987, 2–23.iii.1986 and second label [SE] Bosque San Emilio [;] 30yr old deciduous forest [;] [O] in clearing, fully [;] isolated part of day (ESUW). 2 ♀♀, first label - Costa Rica: Guanacaste [;] Santa Rosa National Pa. [;] 300m. Malaise. Ian Gauld [;] 10–31.i.1987; second label - Bosque San Emilio [;] 50yr old deciduous [;] forest [;] Full shade; third label - SE-8-C [;] 10–31.i.87 (ESUW). 2 ♀♀, first label - Costa Rica: Guanacaste [;] Santa Rosa National Pk. [;] 300m. Malaise. Ian Gauld [;] 31.i–21.ii.87; second label - Bosque Humedo [;] Mature dry forest [;] high proportion [;] Evergreen species [;] Full Shade; third label - BH-10-C [;] 31.i–21.ii.87 (ESUW). 2 ♀♀, first label - Costa Rica: Guanacaste [;] Santa Rosa National Pk. [;] 300m. Malaise. Ian Gauld [;] 31.i–21.ii.87; second label - Bosque San Emilio [;] 50yr old deciduous [;] forest. Full Shade; third label - SE-8-c [;] 31.i–21.ii.87 (ESUW). 1 ♀, first label - Costa Rica: Guanacaste [;] Santa Rosa National Pk. [;] 300m. Malaise. Ian Gauld [;] 31.i–21.ii.87; second label - Open regenerating [;] woodland less than [;] 10yrs. Old. Sun; third label - H-1-0 [;] 31.i–21ii.87 (ESUW). 1 ♀, Costa Rica: Puntarenas, ACLAP [;] Coto Brus. Zona Prot. Las Tablas [;] Est. Biol. Las Alturas [;] 1550m. de Luz [;] 16–23.iii.1999, H. Mendez [;] L.S. 322800-591500 #52467 (ESUW). 5 ♀♀, S.RosaPark, Guan. [;] C. Rica 29 Jan 78 to 14 Feb 78 [;] D. H. Janzen [;] Riparian (AEIC).

#### Comments.

This species is similar to *Heterospilus cushmani* but differs by having the stigma bicolored (entirely brown in *Heterospilus cushmani*), the hind coxa and femur dark brown (yellow or very light brown in *Heterospilus cushmani*), and the mesoscutal lobes granulate but rugose along the notauli (entirely granulate in *Heterospilus cushmani*).

#### Etymology.

The specific name is from the Latin *niger* meaning dark and *coxa* meaning hip in reference to the dark colored hind coxa.

**Figure 88.**
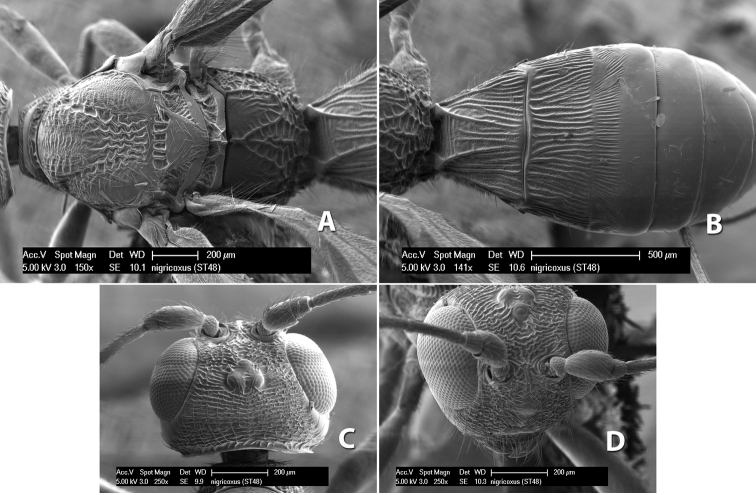
*Heterospilus nigricoxus* Marsh, sp. n., paratype.

**Figure 89.**
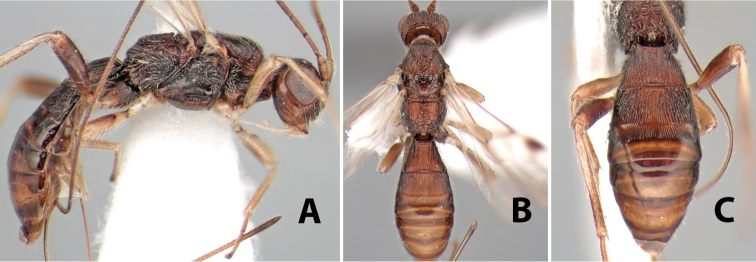
*Heterospilus nigricoxus* Marsh, sp. n.: A, C, holotype; B, paratype.

### Heterospilus nixoni

Marsh sp. n.

http://zoobank.org/327F8E8A-75B5-4829-9065-2F97570E7E14

http://species-id.net/wiki/Heterospilus_nixoni

Figure 90

#### Female.

Body size: 3.0–3.5 mm. Color: head bicolored with face and vertex usually brown and eye orbits and temple yellow or lighter brown; scape yellow or light brown, without lateral longitudinal brown stripe, flagellum brown, apical 3–5 flagellomeres white; mesosoma dark brown, often lighter along notauli and on scutellum; metasoma dark brown, apical terga 3–7 often marked with yellow; legs yellow, all tibiae at extreme base and all tarsi brown; wing veins brown, stigma bicolored brown with yellow at base and apex. Head: vertex transversely costate; frons transversely costate; face smooth; temple in dorsal view narrow, sloping behind eye, about equal to 1/2 eye width; malar space greater than 1/4 eye height; ocell-ocular distance about 2.5 times diameter of lateral ocellus; 20-26 flagellomeres. Mesosoma: mesoscutal lobes granulate; notauli scrobiculate, meeting at scutellum in triangular rugose area; scutellum smooth; prescutellar furrow with 3 cross carinae; mesopleuron smooth; precoxal sulcus weakly scrobiculate, shorter than mesopleuron; venter smooth; propodeum with basal median areas distinctly margined, smooth, basal median carina distinct, areola not distinctly margined, areolar area rugose, lateral areas entirely rugose. Wings: fore wing vein r shorter than vein 3RSa, vein 1cu-a slightly beyond or interstitial with vein 1M; hind wing vein SC+R present, vein M+CU shorter than vein 1M. Metasoma: first tergum longitudinally costate, length greater than apical width; second tergum longitudinally costate; anterior transverse groove weakly present, straight; posterior transverse groove weakly present or absent, often represented by shallow scrobiculate line; third tergum entirely smooth; terga 4–7 smooth; ovipositor equal to 3/4 length of metasoma.

#### Holotype female.

Top label (white, printed) - COSTA RICA: San Jose, [;] Cerro de la Muerte, [;] 26km N San Isidro, 2100m, [;] ii-v.1991 [;] Paul Hanson; second label (red, partially printed and hand written) - HOLOTYPE [;] Heterospilus [;] nixoni [;] P. Marsh. Deposited in ESUW.

#### Paratypes.

2 ♀♀, same data as holotype (ESUW). 8 ♀♀, Costa Rica: San Jose [;] 26km. N. San Isidro [;] just S. of Division [;] 2100m, vi–vii.1992, viii–ix.1991, iv-v.1993, ii–iv.1993 and xi.1992–i.1993 [;] P. Hanson, Malaise [;] secondary growth (ESUW). 2 ♀♀, Costa Rica: San Jose [;] Cerro de la Muerte [;] 6km. N. San Gerardo [;] 2800m, iii–iv 1993 and November 1993 [;] P. Hanson, Malaise (ESUW). 2 ♀♀, Costa Rica: San Jose [;] Cerro de la Muerte [;] 2km W Empalme [;] 2300m, June 1995 [;] P. Hanson, Malaise (ESUW). 1 ♀, COSTA RICA: San Jose [;] 16km S. Empalme [;] 2600m, III-IV 1989 [;] P. Hanson & I. Gauld (ESUW). 1 ♀, Costa Rica: Cartago [;] 4km NE Cañon [;] Genesis II, 2350m [;] vi.1996, P. Hanson (ESUW). 1 ♀, Costa Rica: Puntarenas [;] San Vito, Estac. Biol. [;] Las Alturas, 1050m [;] ix-xi.1992, Paul Hanson, [;] ex. Malaise trap (ESUW).

#### Comments.

The species is distinguished from *Heterospilus reinhardi* by the smooth face.

#### Etymology.

Named for the British entomologist Gilbert E. J. Nixon who described many Old World Braconidae during the middle 1900s.

**Figure 90.**
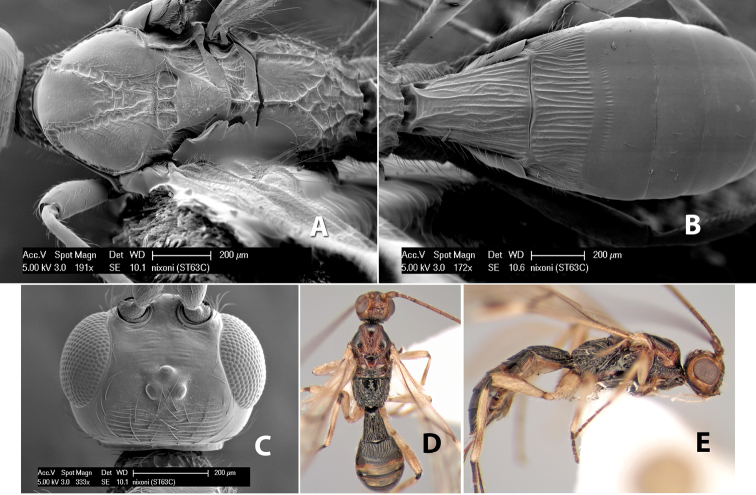
*Heterospilus nixoni* Marsh, sp. n.: **A–C** paratype **D–E** holotype.

### Heterospilus noyesi

Marsh sp. n.

http://zoobank.org/BE4C3A9E-0D41-4F4F-9361-B39C9DC6064F

http://species-id.net/wiki/Heterospilus_noyesi

Figure 91

#### Female.

Body size: 3.5–4.0 mm. Color: head honey yellow or light brown; scape yellow, without lateral longitudinal brown stripe, flagellum brown (broken); mesosoma brown, mesoscutal lobes lighter honey yellow; metasoma brown to dark brown; legs yellow, femora with brown dorsal swelling and on apical half, hind tibia brown on apical half, hind tarsus dark brown; wing veins including stigma brown. Head: vertex transversely costate; frons transversely costate; face rugose; temple in dorsal view less than 1/2 eye width; malar space greater than 1/4 eye height; ocell-ocular distance nearly 2.5 times diameter of lateral ocellus; ? flagellomeres (broken in holotype and paratype). Mesosoma: mesoscutal lobes rugose, lateral lobes costate laterally; notauli scrobiculate, meeting in triangular rugose area before scutellum; scutellum smooth; prescutellar furrow with 3-5 cross carinae; mesopleuron smooth; precoxal sulcus scrobiculate, equal to width of mesopleuron; venter smooth; propodeum with basal median areas indistinct or weakly margined, basal median areas rugose, basal median carina absent, areola not distinctly margined, areolar area areolate-rugose, lateral areas entirely rugose. Wings: fore wing vein r shorter than vein 3RSa, vein 1cu-a beyond vein 1M; hind wing vein SC+R present, vein M+CU equal in length to vein 1M. Metasoma: first tergum costate-rugose, length greater than apical width; second tergum longitudinally costate, apical width about 3 times length; anterior transverse groove present, sinuate; posterior transverse groove present but weak; third tergum costate at base, smooth at apex; terga 4-7 smooth; ovipositor longer than metasoma.

#### Holotype female.

Top label (white, printed) - Costa Rica: Puntarenas [;] San Vito, Las Cruces [;] Wilson Botanical Gardens [;] 18-22.iii.1990, 1150m [;] J.S. Noyes; second label (red, partially printed and hand written) - HOLOTYPE [;] Heterospilus [;] noyesi [;] P. Marsh. Deposited in ESUW.

#### Paratypes.

1 ♀, Costa Rica: Guanacaste [;] 2km SW de Cerro Cacao [;] Est. Cacao, 1000-1400m [;] 21-28.v.1992, Curso Biod. [;] I.N. 323300-375700 #6900 (ESUW).

#### Comments.

This species is distinctive by the rugose face and mesoscutum.

#### Etymology.

Named for John Noyes who collected the holotype specimen.

**Figure 91.**
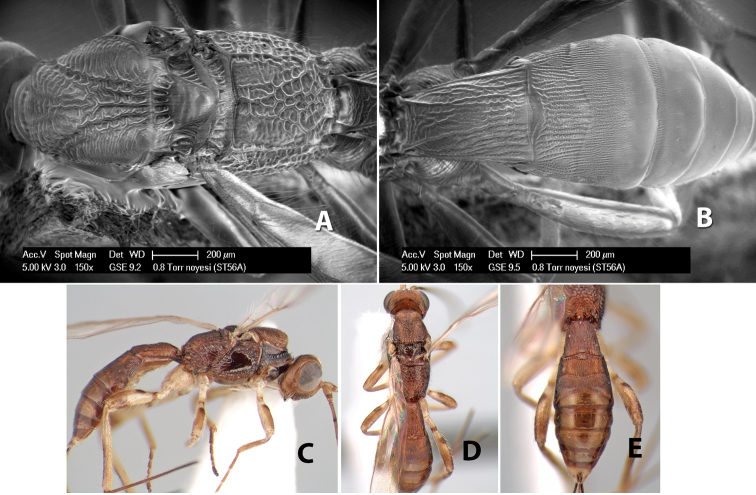
*Heterospilus noyesi* Marsh, sp. n., holotype.

### Heterospilus orbitus

Marsh sp. n.

http://zoobank.org/FAE26088-6165-4915-8C60-539F44DFEB56

http://species-id.net/wiki/Heterospilus_orbitus

Figure 92

#### Female.

Body size: 3.5–5.0 mm. Color: head with face and temple dark brown, vertex and frons yellow; scape yellow without lateral brown stripe, flagellum brown with apical 10-12 flagellomeres white; mesosoma dark brown, mesoscutum yellow, lateral lobes often partially brown; metasomal 1, 2 and base of 3 dark brown, apex of tergum 3 and remaining terga dark honey yellow; wing veins brown, stigma brown with yellow apex; legs yellow. Head: vertex and frons strongly circularly costate around ocelli; face granulate; temple in dorsal view narrow, width less than 1/2 eye width; malar space equal to 1/4 eye height; ocell-ocular distance twice diameter of lateral ocellus; 27–32 flagellomeres. Mesosoma: mesoscutal lobes granulate; notauli scrobiculate, meeting at scutellum in triangular rugose area; scutellum granulate; prescutellar furrow with 3 cross carinae; mesopleuron granulate; precoxal sulcus scrobiculate, shorter than mesopleuron; venter granulate; propodeum with basal median areas margined, weakly granular, basal median carina absent, areola not distinctly margined, areolar area rugose, lateral areas rugose posteriorly, granulate or smooth anteriorly. Wings: fore wing vein r shorter than vein 3RSa, vein 1cu-a interstitial with vein 1M, rarely slightly beyond; hind wing vein SC+R present, vein M+CU shorter than vein 1M. Metasoma: first tergum longitudinally costate, apical width equal to length; second tergum longitudinally costate, basal border with median raised smooth area; anterior transverse groove present, straight; posterior transverse groove present; third tergum costate basally, weakly granulate or smooth apically; terga 4-7 smooth; ovipositor equal to 1/2 length of metasoma.

#### Holotype female.

Top label (white, printed) - Costa Rica: Puntarenas [;] Golfo Dulce, 24km W. [;] Piedras Blancas, 200m [;] ii.1993, Paul Hanson; second label (red, partially printed and hand written) - HOLOTYPE [;] Heterospilus [;] orbitus [;] P. Marsh. Deposited in ESUW.

#### Paratypes.

1 ♀, COSTA RICA: Puntar. [;] Cerro Rincon, 200m [;] S. hito, 745m, ii. [;] 1991, Hanson/Godoy (ESUW). 2 ♀♀, Costa Rica: Puntarenas [;] Est. Sirena, 10100m [;] i-iii.1990, G. Fonesca [;] L.S. 270500-508300 [;] Malaise trap, #7450 (ESUW). 1 ♀, COSTA RICA, Guanac. [;] Estac. Pitilia, 9Km S [;] Santa Cecilia, 700m [;] IX/1988, I. Gauld (ESUW). 2 ♀♀, COSTA RICA, Puntar. [;] Golfo Dulce, 24km W. [;] Piedras Blancas, 200m [;] XII-89-I-90 Hanson (MICR). 2 ♀♀, top label - COSTA RICA, Heredia: [;] Est. Biol. La Selva, 50- [;] 150m, 10°26'N, 84°01'W [;] Nov 1995 and April 1996, INBio-OET; second label - 01 Noviembre 1995 [;] M/08/489 [;] Bosque primario and 1 Abril 1996 [;] Bosque primario [;] M/08/608 (INBC). 2 ♀♀, COSTA RICA: Puntarenas [;] San Vito, Estac. Biol. [;] Los Alturas 1500m [;] iv.1992 P. Hanson (TAMU). 1 ♀, COSTA RICA: Puntarenas [;] RF Golfo Dulce, el 200m [;] 24km W Piedras Blancas [;] P. Hanson xii.1992 (TAMU).

#### Comments.

The costae of the frons and vertex being circularly around the ocelli and the yellow mesoscutum are distinctive for this species.

#### Etymology.

The specific name is from the Latin *orbitus* meaning circular in reference to the circular costae around the ocelli.

**Figure 92.**
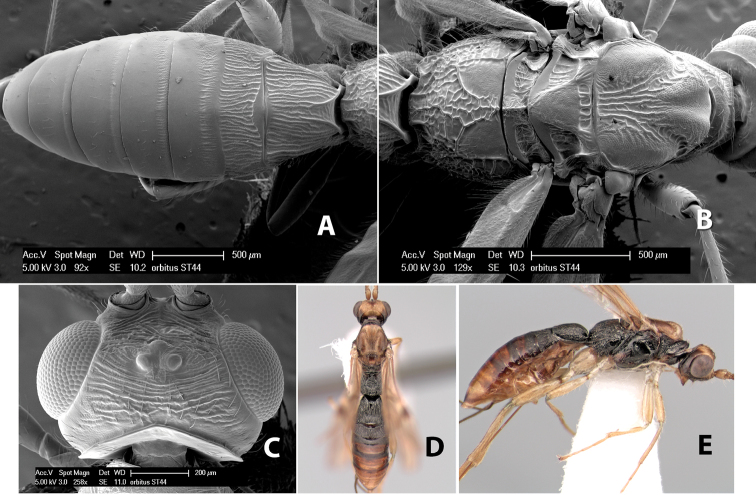
*Heterospilus orbitus* Marsh, sp. n.: **A–C** paratype **D–E** holotype.

### Heterospilus paloverde

Marsh sp. n.

http://zoobank.org/80B896FB-9D8A-4BE3-A479-98427B51DC8B

http://species-id.net/wiki/Heterospilus_paloverde

Figure 93

#### Female.

Body size: 2.5 mm. Color: head dark brown; scape yellow, flagellum brown with apical 4 flagellomeres white; mesosoma dark brown with propleuron and pronotal collar yellow; metasomal tergum 1 brown, terga 2–4 honey yellow medially, brown laterally, terga 5–7 honey yellow; legs yellow; wing vein including stigma brown. Head: vertex weakly striate medially; frons weakly striate; face smooth; temple in dorsal view narrow, sloping behind eye, width 1/2 eye width; malar space greater than 1/4 eye height; ocell-ocular distance slightly greater than 2.5 times diameter of lateral ocellus; 24 flagellomeres. Mesosoma: mesoscutal lobes smooth; notauli scrobiculate, meeting at scutellum in triangular costate area, short depression between carinae; scutellum smooth; prescutellar furrow with 1 cross carina; mesopleuron smooth; precoxal sulcus weakly scrobiculate, shorter than mesopleuron; venter smooth; propodeum with basal median areas margined, granulate, basal median carina absent, areola not distinctly margined, areolar area rugose, lateral areas entirely rugose. Wings: fore wing vein r shorter than vein 3RSa, vein 1cu-a interstitial with vein 1M; hind wing vein SC+R absent, vein M+CU shorter than 1M. Metasoma: first tergum longitudinally costate, length equal to apical width; second tergum longitudinally costate; anterior transverse groove present, straight; posterior transverse groove present; third tergum costate basally, smooth apically; terga 4-7 smooth; ovipositor equal to 3/4 length of metasoma.

#### Holotype female.

Top label (white, printed) - Costa Rica: Guanacaste, ACT [;] Bagaces, P.N. Palo Verde [;] Sector Palo Verde [;] 500 NW de la Est., 40m [;] 4.vi–6.vii.1999, I. Jimenez [;] L.N. 260952-385020 #52849 [;] Malaise trap; second label (red, partially printed and hand written) - HOLOTYPE [;] Heterospilus [;] paloverde [;] P. Marsh. Deposited in ESUW.

#### Paratypes.

Known only from the holotype.

#### Comments.

The dark mesosoma with the yellow propleuron and pronotal collar is distinctive for this species.

#### Etymology.

Named for Palo Verde National Park where the holotype was collected.

**Figure 93.**
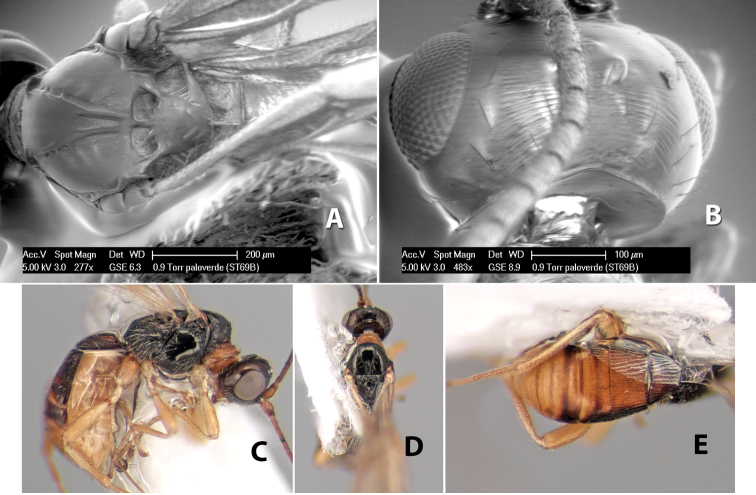
*Heterospilus paloverde* Marsh, sp. n., holotype.

### Heterospilus pappi

Marsh sp. n.

http://zoobank.org/C8022C6F-D197-421D-8CC1-725EA826E348

http://species-id.net/wiki/Heterospilus_pappi

Figure 94

#### Female.

Body size: 2.5–3.0 mm. Color: head with vertex and frons brown to light brown, face and eye orbits yellow; scape yellow without lateral brown stripe, flagellum brown; mesosoma dark brown, often mesoscutum and venter honey yellow; metasomal tergum 1 dark brown, tergum 2 and usually 3 honey yellow medially, dark brown laterally, remainder of terga light brown to brown; wing veins including stigma brown; legs yellow. Head: vertex transversely striate; frons transversely striate; face granulate; temple in dorsal view narrow, width less than 1/2 eye width; malar space equal to 1/4 eye height; ocell-ocular distance 2–2.5 times diameter of lateral ocellus; 19–25 flagellomeres. Mesosoma: mesoscutal lobes granulate; notauli scrobiculate, meeting at scutellum in wide rectangular costate-rugose area; scutellum granulate; prescutellar furrow with 3 cross carinae; mesopleuron granulate; precoxal sulcus scrobiculate, sorter than mesopleuron; venter granulate; propodeum with basal median areas margined, granulate, basal median carina present, areola not distinctly margined, areolar area rugose, lateral areas entirely rugose, apical lateral corners of propodeum produced into small points or tubercle. Wings: fore wing vein r shorter than vein 3RSa, vein 1cu-a beyond vein 1M; hind wing vein SC+R present, vein M+CU shorter than 1M. Metasoma: first tergum longitudinally costate, apical width equal to length; second tergum longitudinally costate; anterior transverse groove present, straight; posterior transverse groove present; third tergum costate basally, smooth apically; terga 4-7 smooth; ovipositor about 3/4 length of metasoma.

#### Holotype female.

Top label (white, printed) - COSTA RICA: [;] Puntar [;] Golfo Dulce, 3km [;] SW Rincon, 10m [;] VI-VIII 1989, Hanson; second label (red, partially printed and hand written) - HOLOTYPE [;] Heterospilus [;] pappi [;] P. Marsh. Deposited in ESUW.

#### Paratypes.

1 ♀, Costa Rica: Guanacaste, ACT [;] Bagaces, P.N. Palo Verde [;] Sec. P. Verde, 0–50m [;] 2–12.xii.1999, I. Jimenez [;] L.N. 260932-385020 #54246 [;] Red de Golpe (ESUW). 2 ♀♀, top label - Costa Rica: Guanacaste [;] Santa Rosa Natl. Park [;] 300m, ex. Malaise trap [;] Site #: BH-12-C and blank [;] Dates: 18.x–8.xi.1986 and 13.i–8.ii.1986 [;] I.D. Gauld & D. Janzen; second label - [BH] Bosque Humedo [;] mature evergreen dry forest [;] [O] in clearing, fully [;] isolated part of day and [BH] Bosque Humedo [;] mature evergreen dry forest [;] [C] more or less fully [;] shaded as possible (ESUW). 1 ♀, Costa Rica: Puntarenas [;] R.F. Golfo Dulce, [;] 3km SW, Rincon, 10m [;] iii.1993 Paul Hanson Coll. [;] Malaise, primary forest (ESUW). 1 ♀, Costa Rica: Puntarenas [;] R.F. Golfo Dulce, 3km [;] S.W. Rincon, 10m [;] I.1992, P. Hanson (ESUW).

#### Comments.

The bicolored body and the brown flagellum are distinctive for this species.

#### Etymology.

Named for my friend and colleague, the Hungarian braconidologist Jeno Papp.

**Figure 94.**
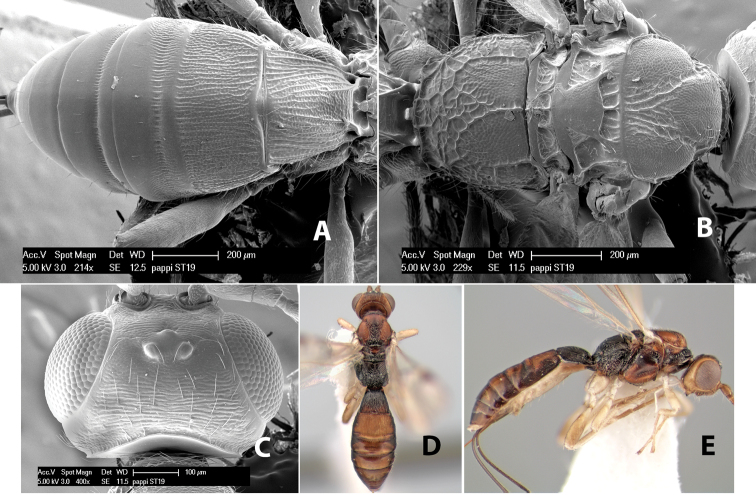
*Heterospilus pappi* Marsh, sp. n.: **A–C** paratype **D–E** holotype.

### Heterospilus petralbus

Marsh sp. n.

http://zoobank.org/4DF466C4-B626-4EAE-9FBB-710C532ECBCA

http://species-id.net/wiki/Heterospilus_petralbus

Figure 95

#### Female.

Body size: 2.0–3.5 mm. Color: head usually brown, often with face lighter; scape yellow with weak lateral longitudinal brown stripe, flagellum brown with apical 3–5 flagellomeres white; mesosoma usually dark brown, mesoscutal lobes often lighter or distinctly light brown, lower portion of mesopleuron and venter occasionally lighter brown; metasomal tergum 1 dark brown, tergum 2 usually dark brown with lateral converging yellow lines, sometimes tergum 2 nearly entirely brown, terga 3–7 brown basally, honey yellow apically; wing veins including stigma brown; legs bicolored yellow and brown, fore and mid legs yellow, hind coxa and trochanters yellow, hind femur yellow on basal 1/3, brown on apical 2/3, hind tibia brown at extreme base, yellow on basal 1/3, brown on apical 2/3, hind tarsus brown. Head: vertex transversely costate; frons transversely costate; face rugose or rugose-areolate; temple in dorsal view narrow, width less than 1/2 eye width; malar space greater than 1/4 eye height; ocell-ocular distance slightly greater than 2.5 times diameter of lateral ocellus; 22–29 flagellomeres. Mesosoma: mesoscutal lobes granulate; notauli scrobiculate, meeting at scutellum in triangular rugose-costate area; scutellum granulate; prescutellar furrow with 3 cross carinae; mesopleuron granulate; precoxal sulcus smooth, shorter than mesopleuron; venter granulate; propodeum with basal median areas margined, granulate-rugose, basal median carina present, areola not distinctly margined, areolar area rugose, lateral areas rugose posteriorly, granulate or smooth anteriorly. Wings: fore wing vein r shorter than vein 3RSa, vein 1cu-a beyond vein 1M; hind wing vein SC+R present, vein M+CU shorter than 1M. Metasoma: first tergum longitudinally costate-rugose, length greater than apical width; second tergum longitudinally costate; anterior transverse groove present, sinuate; posterior transverse groove present; third tergum smooth, often striate antero-laterally; terga 4–7 smooth; ovipositor about as long as metasoma.

#### Holotype female.

Top label (white, printed) - COSTA RICA: Puntar [;] Golfo Dulce, 10km W [;] Piedras Blancas, 100m [;] VI-VIII 1989, Hanson; second label (red, partially printed and hand written) - HOLOTYPE [;] Heterospilus [;] petralbus [;] P. Marsh. Deposited in ESUW.

#### Paratypes.

1 ♀, Costa Rica: Puntarenas [;] Golfo Dulce, 24km W. [;] Piedras Blancas, 200m [;] xii.1991, Paul Hanson (ESUW). 1 ♀, COSTA RICA: [;] 24km W Piedras Blancas [;] 200m, vi–viii 1989 [;] Hanson (ESUW). 2 ♀♀, top label - Costa Rica: Puntarenas [;] Buenos Aires [;] Sendero Los Gigantes [;] Est. Altamira, 1450m; second label - 3–22 February 2000 [;] D. Rubi, Amarilla [;] LS 331700-572200 [;] # 54808 (ESUW). 1 ♀, Costa Rica: Puntarenas [;] San Vito, Las Cruces [;] Wilson Botanical Gardens [;] 18–22.iii.1990, 1150m [;] J.S. Noyes (ESUW). 1 ♀, Costa Rica: Limon, Sec. Cocori [;] 30Km al N, Cariari, 100m [;] xii.1994, E. Rojas, Malaise [;] L.N. 286000-567500 #4525 (ESUW). 1 ♀, Costa Rica: Limon [;] 30km N Cariari, 100m [;] Sector Cocori, Malaise [;] iii.1995, E. Rojas #4524 [;] L.N. 286000-567500 (ESUW). 1 ♀, COSTA RICA-Heredia Prov. [;] La Selva Biological Station [;] 10°26'N, 84°01W, 100m [;] Malaise trap 05, #324 [;] 15.i.1994 [;] Project ALAS (M.05.324) (ESUW). 1 ♀, top label - COSTA RICA: Heredia Pr.; ] La Selva Biol. Sta. [;] 3km S Pto. Viejo [;] 10°26'N, 84°01W; second label - 10.IV.1988 [;] H.A. Hespenheide (ESUW). 1 ♀, Costa Rica: Heredia [;] Braulio Carrillo N.P. [;] 250–500m IV.10.85 [;] Henri Goulet (AEIC). 1 ♀, COSTA RICA, Puntar. [;] Golfo Dulce, 24km W. [;] PiedrasBlancas, 200m [;] XII.89–III.90 Hanson (MICR). 1 ♀, Costa Rica: Puntarenas [;] San Vito - Las Cruces [;] 5-VI-1988 1200m [;] P. Hanson (TAMU). 1 ♀, Est. Altamira, Buenos Aires, Prov. Punta. [;] COSTA RICA. 15 Set–14 Oct 1998. R. [;] Delgado, LS 572100_331700 #2370 (INBC). 1 ♀, Est. Biol. Las Alturas, [;] 1500m, Coto Brus, Prov. [;] Punt., COSTA RICA, [;] M.Zumbado, Ene 1992, [;] L-S-822500-591800 (INBC). 1 ♀, Est. Pitilia, 700m, 9km S [;] Sta. Cecilia, P.N.Guana- [;] caste, Prov.Guan. COSTA [;] RICA, C. Moraga, May [;] 1991, L-N-330200-380200 (INBC). 1 ♀, Quebrada Segundo, Tapanti, Prov. [;] Carta. COSTA RICA. 1150m. JUN [;] 1995, R.Delgado, Amarilla [;] L N 194000 559800 #5345 (INBC). 1 ♀, Est. La Casona, R.B. Monteverde, Prov. [;] Punta. COSTA RICA. 1520m. Jul 1993. N [;] Obando, L N 253250_449700 #2287 (INBC).

#### Comments.

This species is similar to *Heterospilus gahani* but is distinguished by the granulate mesoscutal lobes.

#### Etymology.

The specific name is from the Latin *petra* meaning rock and the Latin *albus* meaning white in reference to the locality of several of the type series being Piedras Blancas, meaning white stones.

**Figure 95.**
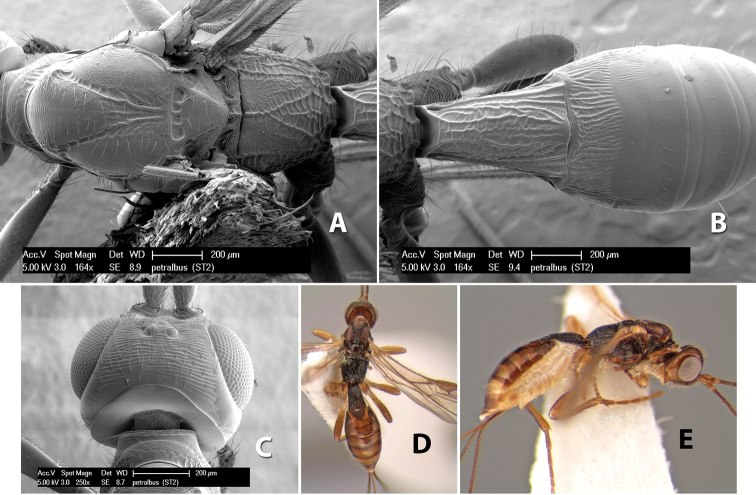
*Heterospilus petralbus* Marsh, sp. n.: **A–C** paratype **D–E** holotype.

### Heterospilus puntarensis

Marsh sp. n.

http://zoobank.org/BC17702F-D92D-49F7-8366-497A44D19A56

http://species-id.net/wiki/Heterospilus_puntarensis

Figure 96

#### Female.

Body size: 4.0 mm. Color: head with vertex brown, remainder including eye orbits yellow; scape yellow without lateral longitudinal brown stripe, flagellum light brown basally to dark brown apically; mesosoma and metasoma dark brown, apical metasomal terga lighter; legs yellow; wing veins brown, stigma bicolored, brown with yellow apex. Head: vertex transversely costate; frons transversely costate; face rugose; temple narrow, not bulging behind eye, width in dorsal view slightly less than eye width; malar space greater than 1/4 eye height; ocell-ocular distance about twice diameter of lateral ocellus; 25–35 flagellomeres. Mesosoma: mesoscutal lobes granulate; notauli scrobiculate, meeting before prescutellar furrow in triangular costate area; scutellum weakly granulate, at least along sides; prescutellar furrow with 3 cross carinae; mesopleuron smooth above precoxal sulcus, weakly striate dorsally; precoxal sulcus scrobiculate, length about half length of mesopleuron; venter granulate; propodeum with basal median areas distinct but not margined, basal median areas granulate, basal median carina distinct but short, areola not distinctly margined, areolar area rugose, lateral areas entirely rugose. Wings: fore wing vein r shorter than vein 3RSa, vein 1cu-a beyond vein 1M; hind wing vein SC+R present, vein M+CU shorter than vein 1M. Metasoma: first tergum length equals apical width, longitudinally costate or costate-rugose, few cross carinae at extreme base; second tergum longitudinally costate, apical width less than 3 times length; anterior transverse groove present, straight; posterior transverse groove distinct; third tergum longitudinally costate on basal 3/4, smooth on apical 1/4; tergum 4 longitudinally costate on basal 3/4, smooth on apical 1/4; terga 5–7 smooth; ovipositor longer than metasoma.

#### Holotype female.

Top label (white, printed) - Costa Rica: Puntarenas [;] R. F. Golfo Dulce [;] 3km. SW. Rincon. 10m. [;] iii.1993 Paul Hanson coll. [;] Malaise. primary forest; second label (red, partially printed and hand written) - HOLOTYPE [;] Heterospilus [;] puntarensis [;] P. Marsh. Deposited in ESUW.

#### Paratypes.

Known only from the holotype.

#### Comments.

The rugose propodeum with basal median areas not distinctly margined and the nearly entirely costate metasomal tergum 4 are distinctive for this species.

#### Etymology.

The specific name is from the type locality of Puntarenas Province.

**Figure 96.**
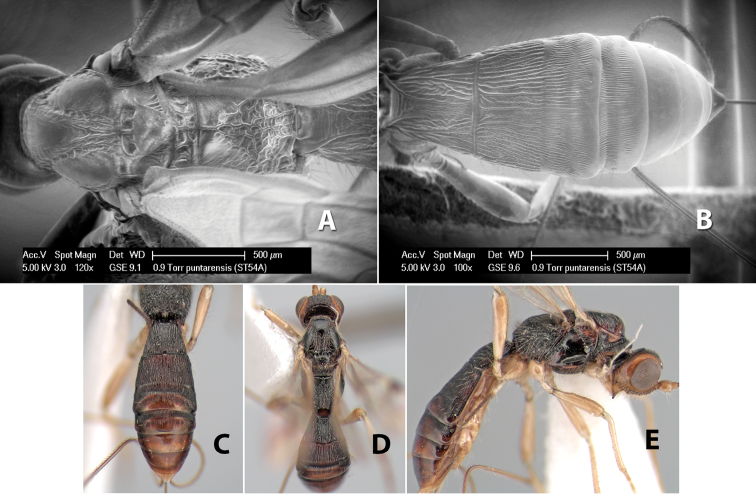
*Heterospilus puntarensis* Marsh, sp. n., holotype.

### Heterospilus quickei

Marsh sp. n.

http://zoobank.org/B8128750-C2C4-4A68-9C20-9AD1E9452855

http://species-id.net/wiki/Heterospilus_quickei

Figure 97

#### Female.

Body size: 2.5–3.0 mm. Color: head with vertex and frons brown, face and eye orbits yellow, frons sometimes yellow; scape yellow without lateral brown stripe, flagellum yellow basally to brown apically with apical 3–5 flagellomeres white; mesosoma brown, lighter brown along notauli and often on pronotum; metasomal terga dark brown, tergum 2 usually honey yellow medially, brown laterally; wing veins including stigma brown; legs yellow. Head: vertex transversely striate; frons transversely striate; face rugose; temple in dorsal view narrow, width less than 1/2 eye width; malar space greater than 1/4 eye height; ocell-ocular distance 2.0–2.5 times diameter of lateral ocellus; 16–19 flagellomeres. Mesosoma: mesoscutal lobes granulate; notauli scrobiculate, meeting at scutellum in rectangular rugose area; scutellum granulate; prescutellar furrow with 1 cross carina; mesopleuron granulate; precoxal sulcus scrobiculate, shorter than mesopleuron, occasionally with striae extending from sulcus to posterior margin of mesopleuron; venter granulate; propodeum with basal median areas margined, granulate, basal median carina absent, rarely extremely short, areola not distinctly margined, areolar area areolate-rugose, lateral areas entirely rugose. Wings: fore wing vein r shorter than vein 3RSa, vein 1cu-a beyond vein 1M; hind wing vein SC+R absent, vein M+CU shorter than vein 1M. Metasoma: first tergum longitudinally costate, apical width usually equal to length, rarely slightly less; second tergum longitudinally costate; anterior transverse groove present, straight; posterior transverse groove present; third tergum costate basally, smooth apically; terga 4–7 smooth; ovipositor as long as metasomal terga 1–2 combined.

#### Holotype female.

Top label (white, partially printed and hand written) - Costa Rica: Guanacaste [;] Santa Rosa Natl. Park [;] 300m, ex. Malaise trap [;] Site #: BH-10-C [;] Dates: 8.ii–2.iii.1986 [;] I.D. Gauld & D. Janzen; second label (white, printed) - [BH] Bosque Humedo [;] mature evergreen dry forest [;] [C] more or less fully [;] shaded as possible; third label (red, partially printed and hand written) - HOLOTYPE [;] Heterospilus [;] quickei [;] P. Marsh. Deposited in ESUW.

#### Paratypes.

3 ♀♀, same data as holotype with additional: Site #:BH-12-C; Dates: 20.xii.86–10.i.1987 and 14.viii–6.ix.1986 (ESUW). 2 ♀♀, top label - Costa Rica: Guanacaste [;] Santa Rosa Natl. Park [;] 300m, ex, Malaise trap [;] Site #: BH-11-O and blank [;] Dates: 6–27.ix.1986 and 18.x–8.xi.1986 [;] I.D. Gauld & D. Janzen; second label - [BH] Bosque Humedo [;] mature evergreen dry forest [;] [O] in clearing, fully [;] isolated part of day (ESUW). 2 ♀♀, top label - Costa Rica: Guanacaste [;] Santa Rosa Natl. Park [;] 300m, ex, Malaise trap [;] Site #: H-4-C [;] Dates: 4–24.v.1986 [;] I.D. Gauld & D. Janzen; second label - [H] open regenerating [;] woodland <10 years old [;] [C] more or less fully [;] shaded as possible (ESUW). 1 ♀, top label - Costa Rica: Guanacaste [;] Santa Rosa Natl. Park [;] 300m, ex, Malaise trap [;] Site #: H-2-O [;] Dates: 20.xii.86–10.i.1987 [;] I.D. Gauld & D. Janzen; second label - [H] open regenerating [;] woodland <10 years old [;] [O] in clearing, fully [;] isolated part of day (ESUW). 5 ♀♀, top label - Costa Rica: Guanacaste [;] Santa Rosa Natl. Park [;] 300m, ex, Malaise trap [;] Site #: SE-6-C and blank [;] Dates: 3-24.viii.1985, 14.viii–6.xi.1986 and 24.xi–20.xii.1986 [;] I.D. Gauld & D. Janzen; second label - [SE] Bosque San Emilio [;] 50yr old deciduous forest [C] more or less fully [;] shaded as possible (ESUW). 2 ♀♀, top label - Costa Rica: Guanacaste [;] Santa Rosa Natl. Park [;] 300m, ex, Malaise trap [;] Site #: SE-&-O and blank [;] Dates: 8–24.xi.1986 and 29.xi–20.xii.1986 [;] I.D. Gauld & D. Janzen; second label - [SE] Bosque San Emilio [;] 50yr old deciduous forest [O] in clearing, fully [;] isolated part of day (ESUW). 1 ♀, top label - Costa Rica: BH-10-C [;] Guanacaste Province [;] Santa Rosa Natl. Pk. [;] 300m, (dry season) [;] 10-31 January 1987; second label - Bosque Humedo, mature [;] dry forest with high [;] proportion evergreen [;] species, fully shaded [;] Townes style Malaise [;] Ian Gauld, coll. (ESUW). 1 ♀, top label - Costa Rica: Guanacaste [;] Santa Rosa National Pk. [;] 300m, Malaise, Ian Gauld [;] 10–31.i.1987; second label - Bosque Humedo [;] Mature dry forest [;] high proportion [;] Evergreen species [;] Sun; third label - BH-9-O [;] 10–31.i.87 (ESUW). 1 ♀, top label - Costa Rica: Guanacaste [;] Santa Rosa National Pk. [;] 300m, Malaise, Ian Gauld [;] 31.i–21.ii.1987; second label - Bosque Humedo [;] Mature dry forest [;] high proportion [;] Evergreen species [;] Sun (ESUW). 2 ♀♀, Costa Rica: Puntarenas [;] Res. Forestal Golfo Dulce [;] 3km. SW Rincon, 10m [;] ii and iv.1993, P. Hanson [;] Malaise, primary forest (ESW). 5 ♀♀, COSTA RICA: Puntar [;] Golfo Dulce, 3km SW [;] Rincon [;] 10m, iii-v 1989 [;] Col. Paul Hanson (ESUW). 1 ♀, COSTA RICA, Puntar. [;] Golfo Dulce, 3Km S [;] Rincón. 10m [;] III-V/1989, Hanson (ESUW). 1 ♀, Costa Rica: Limon [;] 30km N Cariari, 100m [;] Sector Cocori, Malaise [;] iii.1995, E. Rojas #4524 [;] L.N. 286000-567500 (ESUW). 3 ♀♀, S.RosaPark, Guan. [;] C. Rica 17 Oct 77, 3 Oct 77 and 13 Jun 77 [;] D.H Janzen [;] Dry Hill (AEIC).

#### Comments.

The granulate mesopleuron, rugose face and absence of hind wing vein SC+R are distinctive for this species.

#### Etymology.

Named for my colleague and friend, the British braconidologist Donald Quicke.

**Figure 97.**
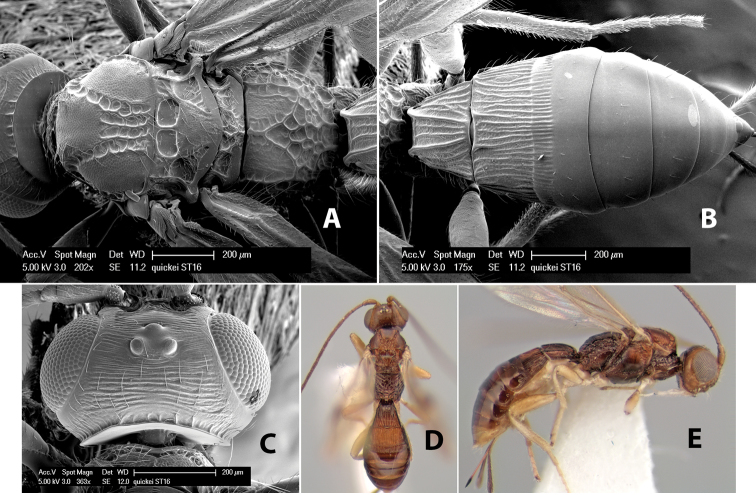
*Heterospilus quickei* Marsh, sp. n.: **A–C** paratype **D–E** holotype.

### Heterospilus ramirezi

Marsh sp. n.

http://zoobank.org/E647FD92-7AF7-43F3-BD98-F1DCE6D41C3B

http://species-id.net/wiki/Heterospilus_ramirezi

Figure 98

#### Female.

Body size: 2.5 mm. Color: head yellow; scape yellow without lateral brown stripe, flagellum yellow basally to brown apically; mesosoma brown with mesoscutum honey yellow; metasomal tergum 1 dark brown, tergum 2–4 lighter brown, tergum 2 sometimes yellow, terga 5–7 honey yellow; wing veins including stigma brown; legs yellow. Head: vertex transversely striate; frons transversely striate; face granulate; temple in dorsal view narrow, width less than 1/2 eye width; malar space equal to 1/4 eye height; ocell-ocular distance about 1.5 times diameter of lateral ocellus; 20 flagellomeres. Mesosoma: mesoscutal lobes granulate, covered by sparse short setae; notauli scrobiculate, meeting at scutellum in triangular rugose area; scutellum granulate; prescutellar furrow with 3 cross carinae; mesopleuron granulate; precoxal sulcus smooth, shorter than mesopleuron; venter granulate; propodeum with basal median areas margined, granulate, basal median carina absent, areola not distinctly margined, areolar area areolate-rugose, lateral areas entirely rugose. Wings: fore wing vein r nearly as long as vein 3RSa, vein 1cu-a beyond vein 1M; hind wing vein SC+R absent, vein M+CU shorter than vein 1M. Metasoma: first tergum longitudinally costate, apical width equal to length; second tergum longitudinally costate; anterior transverse groove present, straight; posterior transverse groove weak or absent; third tergum costate at base, smooth apically; terga 4-7 smooth; ovipositor as long as metasoma.

#### Holotype female.

Top label (white, printed) - COSTA RICA-Heredia Prov. [;] La Selva Biological Station [;] 10°26'N, 84°01'W, 100m [;] Canopy fogging 31 [;] 2.vi.1994 [;] Project ALAS (FPM31); second label (red, partially printed and hand written) - HOLOTYPE [;] Heterospilus [;] ramirezi [;] P. Marsh. Deposited in ESUW.

#### Paratypes.

1 ♀, COSTA RICA: [;] Guanacaste Prov. [;] Cerro el Hacha [;] NW Volcan Orosi [;] 300m, 1988 (ESUW).

#### Comments.

The light brown or honey yellow body color and the strongly rugose mesoscutum which is covered by sparse short hair are distinctive for this species.

#### Etymology.

Named for a good friend from Costa Rica, William Ramirez.

**Figure 98.**
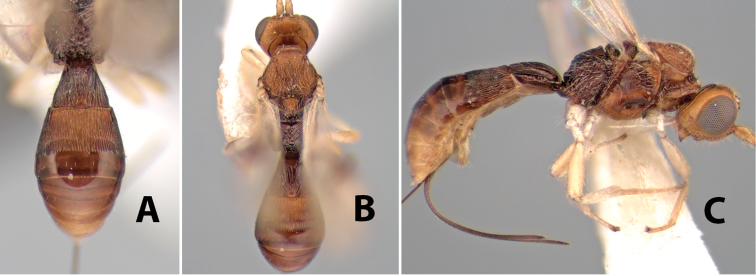
*Heterospilus ramirezi* Marsh, sp. n., holotype.

### Heterospilus ratzeburgi

Marsh sp. n.

http://zoobank.org/50719E02-0C90-4E9F-A5A7-38DB00E68B9B

http://species-id.net/wiki/Heterospilus_ratzeburgi

Figure 99

#### Female.

Body size: 4.0 mm. Color: head bicolored, vertex and frons dark brown, face and eye orbits honey yellow; scape light brown, without lateral longitudinal brown stripe, flagellum brown, apical 7 flagellomeres white; mesosoma dark brown; metasoma dark brown, terga 5–7 yellow; legs yellow, hind tibia brown at extreme apex, hind tarsus brown; wing veins brown, stigma bicolored brown with yellow base and apex. Head: vertex weakly transversely costate; frons weakly transversely costate; face smooth; temple in dorsal view narrow and sloping behind eye, width about equal to 1/2 eye width; malar space greater than 1/4 eye height; ocell-ocular distance about 3 times diameter of lateral ocellus; 30 flagellomeres. Mesosoma: mesoscutal lobes smooth, median lobe with transverse costae at apical corners; notauli scrobiculate, meeting at scutellum in triangular costate area; scutellum smooth; prescutellar furrow with 3 cross carinae; mesopleuron smooth; precoxal sulcus smooth, shorter than mesopleuron; venter smooth; propodeum with basal median areas weakly margined, smooth, basal median carina absent, areola not margined, areolar area areolate-rugose, lateral areas entirely rugose. Wings: fore wing vein r shorter than vein 3RSa, vein 1cu-a beyond vein 1M; hind wing vein SC+R present, vein M+CU shorter than vein 1M. Metasoma: first tergum longitudinally costate, length greater than apical width; second tergum longitudinally costate, width about 4 times length; anterior transverse groove present, straight; posterior transverse groove present; third tergum costate at base, smooth apically; terga 4–7 smooth; ovipositor equal in length to metasoma.

#### Holotype female.

Top label (white, printed) - Costa Rica: Puntarenas [;] R. F. Golfo Dulce, 3km [;] SW Rincon. 10m [;] Malaise-primary forest [;] viii.1991. P. Hanson; second label (red, partially printed and hand written) - HOLOTYPE [;] Heterospilus [;] ratzeburgi [;] P. Marsh. Deposited in ESUW.

#### Paratypes.

Known only from the holotype.

#### Comments.

The smooth mesoscutal lobes and narrow metasomal tergum 2 are distinctive for this species.

#### Etymology.

Named for the German entomologist, J. T. C. Ratzeburg who described many parasitic wasps in forest situations.

**Figure 99.**
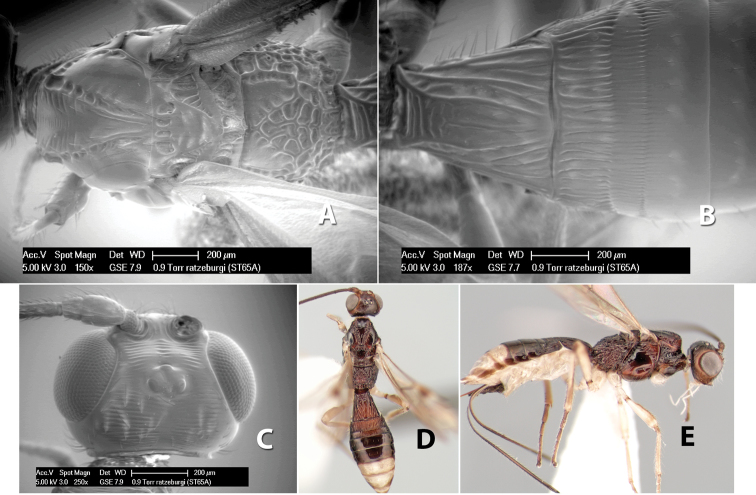
*Heterospilus ratzeburgi* Marsh, sp. n., holotype.

### Heterospilus reinhardi

Marsh sp. n.

http://zoobank.org/3704DA92-9DC3-4DD2-B4D7-6D08B35896F7

http://species-id.net/wiki/Heterospilus_reinhardi

Figure 100

#### Female.

Body size: 2.5–3.5 mm. Color: entire body brown, apical metasomal terga sometimes lighter; scape brown, without lateral longitudinal brown stripe, flagellum entirely brown; legs yellow; wing veins including stigma brown. Head: vertex weakly transversely striate; frons transversely striate; face striate; temple in dorsal view narrow, sloping behind eye, width less than 1/2 eye width; malar space greater than 1/4 eye height; ocell-ocular distance about 2.5 times diameter of lateral ocellus; 19–25 flagellomeres. Mesosoma: mesoscutal lobes granulate; notauli scrobiculate, meeting at scutellum in triangular rugose-costate area; scutellum smooth; prescutellar furrow with 3 cross carinae; mesopleuron smooth; precoxal sulcus weakly scrobiculate, shorter than mesopleuron; venter smooth; propodeum with basal median areas distinctly margined, weakly granulate, basal median carina distinct, areola distinctly margined, areolar area rugose, lateral areas rugose posteriorly, smooth or granulate anteriorly. Wings: fore wing vein r shorter than vein 3RSa, vein 1cu-a beyond vein 1M; hind wing vein SC+R present, vein M+CU nearly as long as vein 1M. Metasoma: first tergum longitudinally costate, length greater than apical width; second tergum longitudinally costate, width less than 3 times length; anterior transverse groove present, straight; posterior transverse groove weak, often nearly absent; third tergum entirely smooth; terga 4–7 smooth; ovipositor 3/4 length of metasoma.

#### Holotype female.

Top label (white, printed) - Costa Rica: Heredia [;] 3km. S. Puerto Viejo, [;] OTS, La Selva, 100m [;] Oct. 1992 P. Hanson, [;] Malaise trap; second label (red, partially printed and hand written) - HOLOTYPE [;] Heterospilus [;] reinhardi [;] P. Marsh. Deposited in ESUW.

#### Paratypes.

1 ♀, Costa Rica, Alajuela [;] Estac. Biol. San Ramon [;] 900m, X-XII-1995 [;] P. Hanson (ESUW). 1 ♀, COSTA RICA: Puntarenas [;] RF Golfo Dulce el. 200m [;] 24km W Piedras Blancas [;] P. Hanson vii.1992 (TAMU).

#### Comments.

This species is distinguished from *Heterospilus nixoni* by its striate face.

#### Etymology.

Name for the German entomologist H. Reinhard who described many braconids in the middle to late 1800s.

**Figure 100.**
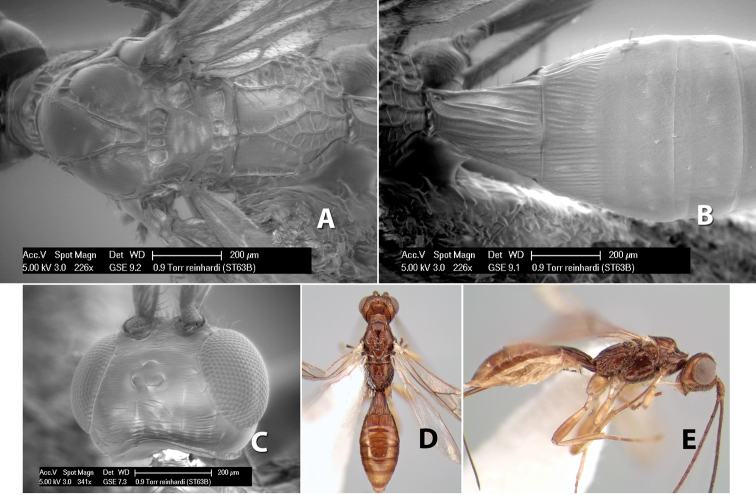
*Heterospilus reinhardi* Marsh, sp. n., holotype.

### Heterospilus rhabdotus

Marsh sp. n.

http://zoobank.org/26F739F1-4514-40B5-ABC7-2DCB0032FC57

http://species-id.net/wiki/Heterospilus_rhabdotus

Figure 101

#### Female.

Body size: 3.0–5.0 mm. Color: head yellow; scape yellow, flagellum entirely yellow or light brown; mesosoma yellow, usually with a dark brown stripe laterally from pronotum, along mesopleuron dorsally and dorso-laterally on propodeum, mesosoma occasionally entirely or mostly brown; metasoma yellow, terga 3–5 usually brown on posterior edge; wing veins brown, stigma usually bicolored yellow with brown center, occasionally entirely yellow or light brown; legs yellow. Head: vertex and frons transversely costate; face striate-granulate; temple smooth or weakly granulate; temple in dorsal view less than 1/2 eye width, narrow and sloping behind eye; malar space greater than 1/4 eye height; ocell-ocular distance 1.5–2.5 times diameter of lateral ocellus; 27–37 flagellomeres. Mesosoma: mesoscutal lobes granulate; notauli scrobiculate, meeting before prescutellar furrow in triangular rugose area; scutellum granulate; prescutellar furrow with 3–5 cross carinae; mesopleuron smooth at least just above precoxal sulcus, often costate or granulate dorsally; precoxal sulcus scrobiculate, about 1/2 length of mesopleuron; venter smooth or weakly granulate; propodeum with basal median areas distinctly margined, basal median areas granulate, basal median carina distinct but short, areola not distinctly margined, areolar area rugose, lateral areas rugose entirely. Wings: fore wing vein r about 1/2 length of vein 3RSa, vein 1cu-a distinctly beyond vein 1M; hind wing vein SC+R present, vein M+CU shorter than vein 1M. Metasoma: first tergum length equal to apical width, longitudinally costate-rugose, often granulate between costae, often with transverse costae at base; second tergum apical width slightly more than 3 times median length, longitudinally costate, often granulate between costae; anterior transverse groove distinct and sinuate, posterior groove weak or often absent; third tergum costate at base, smooth or weakly granulate at apex; terga 4-7 smooth or weakly granulate at base, smooth at apex; ovipositor equal to or longer than metasoma.

#### Holotype female.

Top label (white, partially printed and had written) - Costa Rica: Guanacaste [;] Santa Rosa Nat. Park [;] 300m, ex. Malaise trap [;] Site # BH-9-0 [;] Dates: 20.xi.86–10.i.1987 [;] I.D. Gauld & D. Janzen; second label (white, printed) - [BH] Bosque Humedo [;] mature evergreen dry forest [;] [O] in clearing, fully [;] isolated part of day; third label (red, partially printed and hand written) - HOLOTYPE [;] Heterospilus [;] rhabdotus [;] P. Marsh. Deposited in ESUW.

#### Paratypes.

10 ♀♀, same data as holotype except: Site #: BH-11-O; Dates: 18.i–8.ii.1986, 16.xi–3.xii.1985, 8.ii–2.iii.1986, 2–23.iii.1986, 29.xi–20.xii.1986 and 28.xii.85-18.i.1986 (ESUW). 8 ♀♀, top label - Costa Rica: Guanacaste [;] Santa Rosa Nat. Park [;] 300m, ex. Malaise trap [;] Site # SE-5-0, SE-7-O and blank [;] Dates: 13.ix–4.x.1986, 28.xii.85–18.i.1986, 31.i–21.ii.1987, 26.x–16.xi.1985, 10–31.i.1987 and 7–18.xii.1985 [;] I.D. Gauld & D. Janzen. second label - [SE] Bosque San Emilio [;] [O] in clearing, fully [;] isolated part of day (ESUW). 11 ♀♀, top label - Costa Rica: Guanacaste [;] Santa Rosa Nat. Park [;] 300m, ex. Malaise trap [;] Site # SE-8-C, SE-6-C and blank [;] Dates: 20.xii–10.i.1986/7, 8–29.xi.1986, 18.x–8.xi–1986, 29.xi–20.xii.1986, 2–23.iii.1986, 31.i–21.ii.1986, 7–28.xii.1985, 16.xi–7.xii.1985 and 8–26.x.1985 [;] I.D. Gauld & D. Janzen. second label - [SE] Bosque San Emilio [;] [C] more or less fully [;] shaded as possible (ESUW). 4 ♀♀, top label - Costa Rica: Guanacaste [;] Santa Rosa Nat. Park [;] 300m, ex. Malaise trap [;] Site # H-2–0 and blank [;] Dates: 28.xii.85–18.i.1986, 20.xii.86–10.i.1987, 21.ii–14.iii.1987 and 10–31.i.1987 [;] I.D. Gauld & D. Janzen. second label - [H] open regenerating [;] woodland <10 year old [O] in clearing, fully [;] isolated part of day (ESUW). 7 ♀♀, top label - Costa Rica: Guanacaste [;] Santa Rosa Nat. Park [;] 300m, ex. Malaise trap [;] Site # BH-12-C, BH-10-C and blank [;] Dates: 18.i–8.ii.1986, 16.xi–7.xii.1985 and 8.ii–2.ii.1986 [;] I.D. Gauld & D. Janzen. second label - [BH] Bosque Humedo [;] mature evergreen dry forest [;] [C] more or less fully [;] shaded as possible (ESUW). 2 ♀♀, top label - Costa Rica: Guanacaste [;] Santa Rosa Nat. Park [;] 300m, ex. Malaise trap [;] Site # H-2-C and blank [;] Dates: 18.i–8.ii.1986 and 31.i–21.ii.1987 [;] I.D. Gauld & D. Janzen. second label - [H] open regenerating [;] woodland <10 year old [;] [C] more or less fully [;] shaded as possible (ESUW). 3 ♀♀, top label - Costa Rica: Guanacaste, Santa [;] Rosa Nat'l Park, Bosque San [;] Emilio, trap #5 and 7 in clearing, 300m [;] XII/28/85-I/18/1986 and II/8-III/2/1986, I. Gauld; second label - [SE] Bosque San Emilio [;] 50yr old deciduous forest [;] [O] in clearing, fully [;] isolated part of day (ESUW). 1 ♀, top label - Costa Rica: Guanacaste, Santa [;] Rosa Nat'l Park, Bosque Humedo [;] trap #9 in clearing, 300m [;] XII/7–28/1985, I. Gauld; second label - [BH] Bosque Humedo [;] mature evergreen dry forest [;] [O] in clearing, fully [;] isolated part of day (ESUW). 2 ♀♀, top label - Costa Rica: BH-10-C [;] Guanacaste Province [;] Santa Rosa Natl. Pk. [;] 300m, (dry season) [;] 10–31 January 1987; second label - Bosque Humedo, mature [;] dry forest with high [;] proportion evergreen [;] species, fully shaded [;] Townes style Malaise [;] Ian Gauld coll. (ESUW). 8 ♀♀, top label - Costa Rica: Guanacaste [;] Santa Rosa National Pk. [;] 300m, Malaise, Ian Gauld [;] 10–31.i.1987; second label - Bosque Humedo [;] mature dry forest [;] high proportion [;] evergreen species [;] Sun; third label - BH-9-O [;] 10–31.i.87 (ESUW). 2 ♀♀, top label - Costa Rica: Guanacaste [;] Santa Rosa National Pk. [;] 300m, Malaise, Ian Gauld [;] 10–31.i.1987; second label - Bosque San Emilio [;] 50yr Old deciduous [;] Forest [;] Full Shade; third label - SE-6 and 8-C (ESUW). 1 ♀, top label - Costa Rica: Guanacaste [;] Santa Rosa National Pk. [;] 300m, Malaise, Ian Gauld [;] 31.i–21.ii.1987; second label - Bosque San Emilio [;] 50yr Old deciduous [;] Forest [;] Sun; third label - SE-7-O [;] 31.i–21.ii.87 (ESUW). 2 ♀♀, top label - Costa Rica: Guanacaste [;] Santa Rosa National Pk. [;] 300m, Malaise, Ian Gauld [;] 31.i–21.ii.1987; second label Bosque Humedo [;] mature dry forest [;] high proportion [;] evergreen species [;] Sun; third label - BH-9-O [;] 31.i–21.ii.87 (ESUW). 2 ♀♀, top label - Costa Rica: Guanacaste [;] Santa Rosa National Pk. [;] 300m, Malaise, Ian Gauld [;] 31.i–21.ii.1987; second label Bosque Humedo [;] mature dry forest [;] high proportion [;] evergreen species [;] Full Shade; third label - BH-10 and 12-C [;] 31.i–21.ii.87 (ESUW). 1 ♀, top label - Costa Rica: Guanacaste [;] Santa Rosa National Pk. [;] 300m, Malaise, Ian Gauld [;] 18.x–8.xi.1986; second label - Open regenerating [;] Woodland less than [;] 10 yrs. Old. Sun; third label - H-3-O [;] 18.x–8.xi.86 (ESUW). 1 ♀, top label - Costa Rica: Guanacaste [;] Santa Rosa National Pk. [;] 300m, Malaise, Ian Gauld [;] 18.x–8.xi.1986; second label -Bosque San Emilio [;] 50 yr. old deciduous [;] Forest. Sun; third label - SE-7-O [;] 18.i–8.ii.86 (ESUW). 1 ♀, Costa Rica: Guanacaste, ACT [;] Bagaces, P.N. Palo Verde [;] Sec. P. Verde, 0–50m [;] Extremo E Campo Aterrizaje [;] Malaise trap, #53260 [;] 17.viii–13.ix.1999. I. Jimenez [;] L.N. 560952–385020 (ESUW). 1 ♀, Costa Rica: Guanacaste [;] Santa Rosa Natl. Park [;] 300m, ex. Malaise trap [;] blank [;] Dates: 23.iii–13.iv.1986 [;] I.D. Gauld & D. Janzen (ESUW). 1 ♀, Costa Rica, Guanacaste Pr. [;] Guan. Conservation Area [;] Santa Rosa Hdq., 200m [;] Malaise trap 22–26 VII 1997 [;] 3x night L.J. van der Ent (ESUW). 1 ♀, COSTA RICA: [;] Guanacaste Prov. [;] Cerro el Hacha [;] NW Volcan Orosi 300m. 1988 (ESUW). 2 ♀♀, Costa Rica: Puntarenas [;] R.F. Golfo Dulce, 24km. W [;] Piedras Blancas, 200m [;] I.1993 and II.1993, P. Hanson (ESUW). 2 ♀♀, COSTA RICA: Puntarenas [;] Rd. to Rincon, 24km W. [;] Pan-Amer. Hwy, 200m [;] II-III 1989 and III-V 1989, Hanson & Gauld (ESUW). 3 ♀♀, COSTA RICA: [;] Puntar [;] Golfo Dulce, 3km [;] SW. Rincon, 10m [;] iii-v 1989 and VI-VIII 1989, Hanson (ESUW). 1 ♀, Costa Rica: Puntarenas [;] Pen. Osa, Puerto Jimenez [;] 10m, July 1991, full sun, [;] grassy & weedy site [;] P. Hanson, ex. Malaise (ESUW). 1 ♀, COSTA RICA: Puntar [;] Golfo Dulce, 10km W Piedras Blancas, 100m [;] VI-VIII 1989, Hanson (ESUW). 2 ♀♀, Costa Rica: Puntarenas [;] R.F. Golfo Dulce, 5km. [;] W. Piedras Blancas, 100m I-1993, P. Hanson (ESUW). 1 ♀, COSTA RICA: [;] San Jose [;] Ciudad Colon [;] 800m, iii-iv 1990 [;] Col. Luis Fournier (ESUW). 1 ♀, COSTA RICA-Heredia Prov. [;] La Selva Biological Station [;] 10°26'N, 84°01'W, 100m [;] Canopy fogging 32 [;] 3.xi.1994 [;] Project ALAS (FVK32) (ESUW). 1 ♀, Estac. Santa Rosa, 300m. [;] Guanacaste Prov. COSTA [;] RICA, Jan 1989 [;] GNP Biodiversity Survey [;] w85 37'06", N10 50'15" (INBC). 1 ♀, VicinityEstac.Murcielago [;] 8km SW Cuajiniquil, [;] Guanacaste Prov. COSTA [;] RICA, 100m, Feb 1989 [;] GNP Biodiversity Survey [;] W85 43'59", N10 54'08" (INBC). 1 ♀, Los Almendros, P. N. Guanacaste, A. C. [;] Guanacaste, Prov. Guana. COSTA RICA [;] 300m. 8 Feb–1 Mar 1993, E. E. López, LN [;] 334800_369800 #2005 (INBC). 1 ♀, top label - COSTA RICA, Heredia: [;] Est. Biol. La Selva, 50- [;] 150m, 10°26'N, 84°01W [;] Feb 1998, INBio-OET; second label - 17 Febrero 1998 [;] Bosque secundario [;] L/15/318 (INBC). 23 ♀♀, S.RosaPark, Guan. [;] C. Rica, various dates from Dec, 1976 to March 1978 [;] D. H. Janzen [;] Riparian and Dry Hill (AEIC).

#### Comments.

The longitudinal brown stripe laterally on the mesosoma is characteristic for this species.

#### Etymology.

The species name is from the Greek *rhabdotos* meaning striped in reference to the brown stripe on the mesosoma.

**Figure 101.**
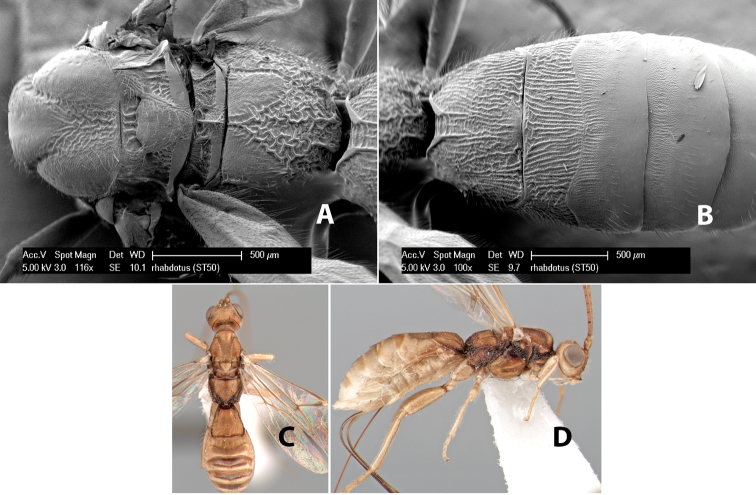
*Heterospilus rhabdotus* Marsh, sp. n.: **A, B, D** paratype **C** holotype.

### Heterospilus rinconensis

Marsh sp. n.

http://zoobank.org/781EF50E-E37A-4B32-81E0-33F204EB3ECB

http://species-id.net/wiki/Heterospilus_rinconensis

Figure 102

#### Female.

Body size: 3.0–4.0 mm. Color: head with vertex and frons yellow, face and temple brown; scape yellow with lateral longitudinal brown stripe, flagellum brown with apical 3–5 flagellomeres white; mesosoma dark brown, mesoscutum with yellow along notauli, middle lobe often partially yellow; metasomal tergum 1 dark brown, tergum 2 dark brown or yellow medially with dark brown laterally, terga 3-4 dark brown basally, yellow apically, terga 5-6 yellow laterally and brown medially, tergum 7 brown; wing veins including stigma brown; legs with coxae and trochanters light yellow or white, fore and mid femora, tibiae and tarsi yellow, hind femur light brown basally, honey yellow apically, hind tibia yellow with dark brown at extreme base, hind tarsus brown. Head: vertex transversely costate; frons transversely costate; face granulate; temple in dorsal view narrow, width less than eye width; malar space equal to 1/4 eye height; ocell-ocular distance at least 2.5 times diameter of lateral ocellus; 26–27 flagellomeres. Mesosoma: mesoscutal lobes granulate; notauli scrobiculate, meeting at scutellum in triangular rugose area; scutellum granulate; prescutellar furrow with 3 cross carinae; mesopleuron granulate; precoxal sulcus weakly scrobiculate or smooth, shorter than mesopleuron; venter granulate; propodeum with basal median areas margined, granulate, basal median carina present, areola distinctly margined, areolar area rugose, lateral areas rugose posteriorly, granulate anteriorly. Wings: fore wing vein r much shorter than vein 3RSa and nearly on same line, vein 1cu-a beyond vein 1M; hind wing vein SC+R present, vein M+CU shorter than vein 1M. Metasoma: first tergum longitudinally costate, apical width equal to length; second tergum longitudinally costate; anterior transverse groove present, straight; posterior transverse groove weak or absent; third tergum granulate; terga 4-5 granulate, terga 6-7 smooth; ovipositor 3/4 length to equal to length of metasoma.

#### Holotype female.

Top label (white, printed) - COSTA RICA: Puntarenas [;] 3km S. Rincon, 10m [;] II-III 1989 [;] P. Hanson & I. Gauld; second label (red, partially printed and hand written) - HOLOTYPE [;] Heterospilus [;] rinconensis [;] P. Marsh. Deposited in ESUW.

#### Paratypes.

1 ♀, Costa Rica: Puntarenas, [;] R.F. Golfo Dulce, 5km. [;] W. Piedras Blancas, 100m [;] viii-ix.1991, P. Hanson [;] Malaise nr. second growth (ESUW). 1 ♀, Costa Rica: Puntarenas, ACO [;] Golfito, Est. Agujas, 250-350m [;] Res. Ftal. Golfo Dulce, Amarilla [;] 3–24.vii.1999, J. Azofeifa [;] L.S. 276750-526550 #52839 (ESUW). 1 ♀, Costa Rica, Puntarenas [;] R.F. Golfo Dulce, 24km. W. [;] Piedras Blancas, 200m [;] VI-1991, P. Hanson (ESUW). 1 ♀, COSTA RICA: Puntarenas [;] R.F. Golfo Dulce, [;] 24km W. Piedras Blancas, [;] 200m [;] Feb. 1992, Paul Hanson (ESUW). 1 ♀, COSTA RICA-Heredia Prov. [;] La Selva Biological Station [;] 10°26'N, 84°01'W, 100m [;] Malaise trap 13, #303 [;] 15.xii.1993 [;] Project ALAS (M.13.303) (ESUW). 1 ♀, Costa Rica: Limon [;] 30km N Cariari, 100m [;] Sector Cocori, Malaise [;] iii.1995, E. Rojas #4524 [;] L.N. 286000-567500 (ESUW). 1 ♀, COSTA RICA: *Punt-* [;] *arenas*. 7km SW Rincon [;] 31.v–7.vi.1998; B. Brown [;] & V. Berezovskiy; Mal. [;] Trp. #5; 2nd growth (AEIC). 1 ♀, COSTA RICA: Puntarenas [;] RF Golfo Dulce, el 200m [;] 24km W Piedras Blancas [;] P. Hanson xi.1992 (TAMU). 1 ♀, Sector Cerro Cocori, Fca. de. E. [;] Rojas, 150m, Prov. Limón, [;] COSTA RICA. Mar 1993. E. Rojas [;] L-N-286000,567500 (INBC). 1 ♀, COSTA RICA. Prov. Limón, R.B. [;] Hitoy Cerere, Send. Espavel. 560m, 15 [;] MAR 2003, R. Gamboa, Red de Golpe [;] L. S. 401200 569800 #73280 (INBC). 1 ♀, top label - COSTA RICA, Heredia [;] Est. Biol. La Selva, 50- [;] 150m, 10°26'N, 84°01W [;] Apr 1996, INBio-OET; second label - 1 Abril 1996 [;] Bosque primario [;] M/07/608 (INBC).

#### Comments.

The bicolored mesoscutum is distinctive for this species.

#### Etymology.

Named for the locality of the holotype, Rincon in Puntarenas Province.

**Figure 102.**
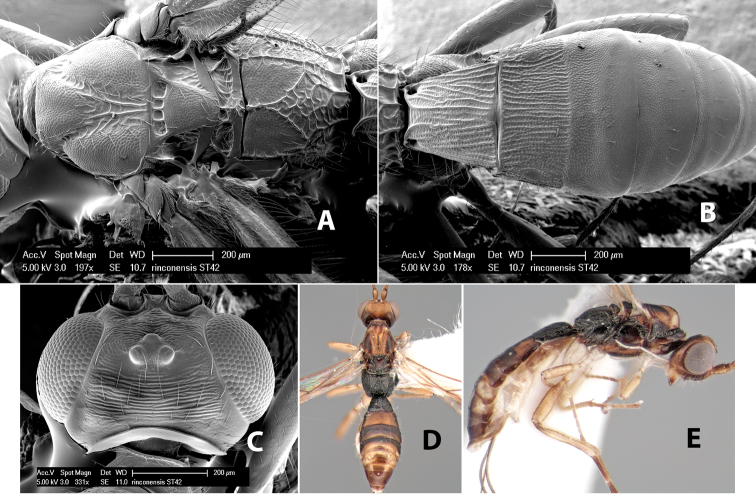
*Heterospilus rinconensis* Marsh, sp. n.: **A–C, E** paratype **D** holotype.

### Heterospilus rohweri

Marsh sp. n.

http://zoobank.org/E70B74A9-5F60-4B3C-9FB4-A8701DDB5CC4

http://species-id.net/wiki/Heterospilus_rohweri

Figure 103

#### Female.

Body size: 4.0 mm. Color: head with vertex brown, eye orbits, frons and face honey yellow, scape yellow without lateral longitudinal brown stripe, flagellum entirely brown; mesosoma dark brown except mesoscutum and scutellum lighter brown; metasoma with terga 1, 2, and base of 3 dark brown, tergum 3 apically and terga 4-7 honey yellow; legs yellow; wing veins brown, stigma brown. Head: vertex transversely costate; frons transversely costate; face rugose; temple in dorsal view narrow, not bulging, less than 1/2 eye width; malar space equal to 1/4 eye height; ocell-ocular distance about twice diameter of lateral ocellus; 31 flagellomeres. Mesosoma: mesoscutal lobes granulate; notauli scrobiculate, meeting at scutellum in triangular costate-rugose area; scutellum smooth; prescutellar furrow with one cross carina; mesopleuron smooth; precoxal sulcus smooth, shorter than mesopleuron; venter smooth; propodeum with basal median areas distinctly margined, smooth, basal median carina present, areola not distinctly margined, areolar area rugose, lateral areas entirely rugose. Wings: fore wing vein r shorter than vein 3RSa, vein 1cu-a beyond vein 1M; hind wing vein SC+R present, vein M+CU shorter than 1M. Metasoma: first tergum longitudinally costate, length greater than apical width; second tergum longitudinally costate; anterior transverse groove present, straight; posterior transverse groove present; third tergum costate basally, smooth apically; terga 4–7 smooth; ovipositor longer than metasoma.

#### Holotype female.

Top label (white, printed) - COSTA RICA, Heredia [;] Est. Biol. La Selva, 50- [;] 150m. 10°26'N, 84°01W [;] Aug 1993, INBio-OET; second label (white, printed) - 03 Agosto 1993 [;] Bosque primario [;] M/04/167; third label - INBio bar code; fourth label (red, partially printed, hand written) - HOLOTYPE [;] Heterospilus [;] rohweri [;] P. Marsh. Deposited in ESUW.

#### Paratypes.

Known only from the holotype.

#### Comments.

This species is distinguished by the lighter colored mesoscutum, longer ovipositor, and single cross carina in prescutellar furrow.

#### Etymology.

Named for S. A. Rohwer who described many Braconidae in the early 1900s.

**Figure 103.**
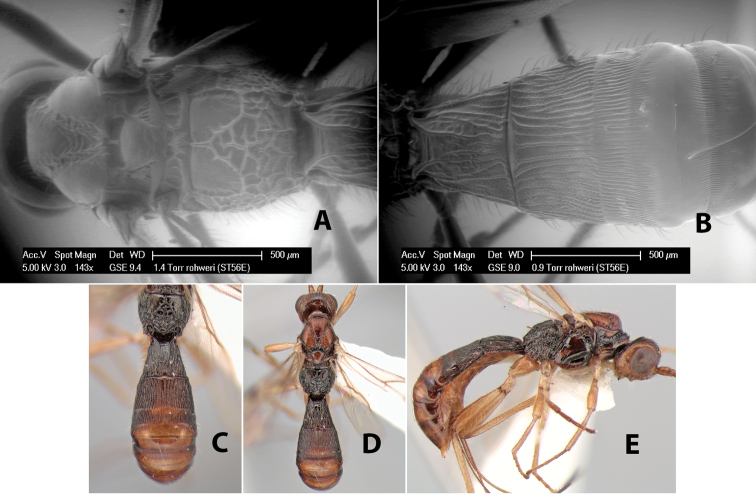
*Heterospilus rohweri* Marsh, sp. n., holotype.

### Heterospilus romani

Marsh sp. n.

http://zoobank.org/17D97598-E096-4FAE-8397-0339793C35C8

http://species-id.net/wiki/Heterospilus_romani

Figure 104

#### Female.

Body size: 2.5 mm. Color: head dark brown, scape yellow with lateral longitudinal brown stripe, flagellum brown with apical 5–8 flagellomeres white; mesosoma dark brown; metasomal 1–4 dark brown, tergum 5 brown at base, yellow at apex, terga 6–7 yellow; wing veins brown, stigma brown with small yellow spot at apex; legs yellow. Head: vertex transversely costate; frons transversely costate; face weakly granulate; temple in dorsal view narrow, sloping behind eye, width less than 1/2 eye width; malar space greater than 1/4 eye height; ocell-ocular distance about 2.5 times diameter of lateral ocellus; 27 flagellomeres. Mesosoma: mesoscutal lobes granulate; notauli scrobiculate, meeting at scutellum in triangular costate area; scutellum granulate; prescutellar furrow with 1 cross carina; mesopleuron granulate; precoxal sulcus smooth, shorter than mesopleuron; venter granulate; propodeum with basal median areas margined, granulate, basal median carina present, areola distinctly margined, areolar area rugose, lateral areas rugose posteriorly, granulate anteriorly. Wings: fore wing vein r shorter than vein 3RSa, vein 1cu-a beyond vein 1M; hind wing vein SC+R present, vein M+CU shorter than vein 1M. Metasoma: first tergum longitudinally costate-granulate, apical width less than length; second tergum longitudinally costate-granulate, about 4 times as wide as long; anterior transverse groove present, sinuate; posterior transverse groove present; third tergum granulate with smooth apex; terga 4-7 granulate at base; ovipositor about 3/4 length of metasoma.

#### Holotype female.

Top label (white, printed) - Costa Rica: Puntarenas [;] ACO, R.F. Golfo Dulce [;] Golfito, Estacion Agujas [;] La Bonanza, 495m; second label (white, printed) - 15 Sept.-15 Oct. 1999 [;] J. Azofeifa, Malaise trap [;] L.S. 276000-526550 [;] # 53487; third label (red, partially printed and hand written) - HOLOTYPE [;] Heterospilus [;] romani [;] P. Marsh. Deposited in ESUW.

#### Paratypes.

1 ♀, COSTA RICA, Puntar. [;] Golfo Dulce, 24km W. [;] PiedrasBlancas, 200m [;] III-VI-90 Hanson (MICR).

#### Comments.

The granulate metasomal terga, at least at base, and the single cross carina in the prescutellar furrow are distinctive for this species.

#### Etymology.

Named for A. Roman who described numerous Neotropical braconids in the early 1900s.

**Figure 104.**
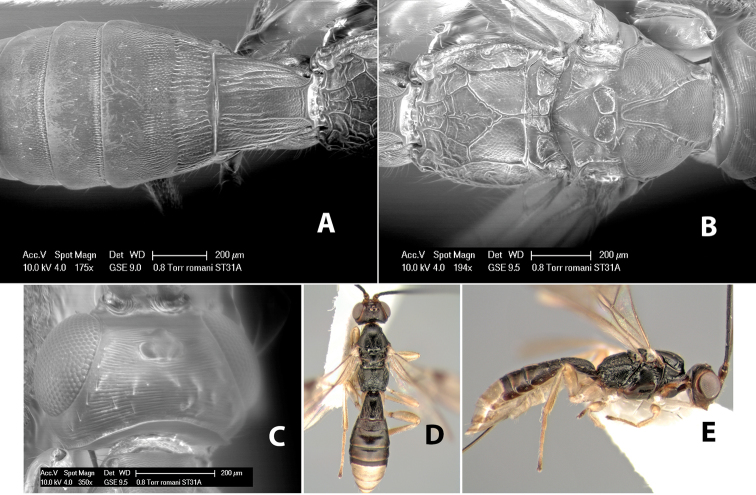
*Heterospilus romani* Marsh, sp. n., holotype.

### Heterospilus rugosus

Marsh sp. n.

http://zoobank.org/9737A752-1C90-424E-9598-FC22D9412364

http://species-id.net/wiki/Heterospilus_rugosus

Figure 105

#### Female.

Body size: 2.5 mm. Color: head honey yellow; scape yellow without lateral brown stripe, flagellum brown; mesosoma dark brown; metasomal terga dark brown, apical terga somewhat lighter; wing veins including stigma brown; legs yellow. Head: vertex transversely costate; frons transversely costate; face rugose; temple in dorsal view narrow, width less than 1/2 eye width; malar space equal to 1/4 eye height; ocell-ocular distance about 1.5 times diameter of lateral ocellus; 18 flagellomeres. Mesosoma: mesoscutal lobes granulate, rugose along notauli, lobes covered nearly entirely with sparse short yellow hair; notauli scrobiculate, meeting at scutellum in triangular rugose area; scutellum rugose; prescutellar furrow with 3–5 cross carinae; mesopleuron granulate; precoxal sulcus scrobiculate, shorter than mesopleuron; venter granulate; propodeum with basal median areas not distinctly margined, rugose or areolate-rugose, basal median carina absent, areola not margined, areolar area areolate, lateral areas entirely rugose. Wings: fore wing vein r about equal in length to vein 3RSa, vein 1cu-a beyond vein 1M; hind wing vein SC+R absent, vein M+CU about equal to vein 1M. Metasoma: first tergum longitudinally costate, median raised area prominent, apical width slightly greater than length; second tergum longitudinally costate; anterior transverse groove present, straight; posterior transverse groove absent; third tergum costate basally, smooth apically; terga 4–7 smooth; ovipositor equal to combined lengths of metasomal terga 1–2.

#### Holotype female.

Top label (white, printed) - COSTA RICA-Heredia Prov. [;] La Selva Biological Station [;] 10°26'N, 84°01'W, 100m [;] Canopy fogging 21 [;] 10.x.1994 [;] Project ALAS (FOT21); second label (red, partially printed and hand written) - HOLOTYPE [;] Heterospilus [;] rugosus [;] P. Marsh. Deposited in ESUW

#### Paratypes.

Known only from the holotype.

#### Comments.

The strongly rugose scutellum, mesoscutum and propodeum are distinctive for this species.

#### Etymology.

The specific name is from the Latin *rugosus* meaning wrinkled in reference to the rugose mesosoma.

**Figure 105.**
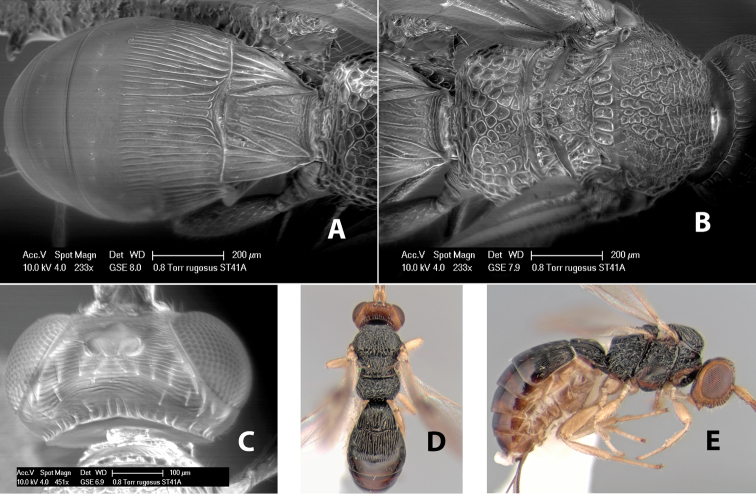
*Heterospilus rugosus* Marsh, sp. n., holotype.

### Heterospilus santarosensis

Marsh sp. n.

http://zoobank.org/FD80B555-D9A8-4498-BED7-D97B7FA0BF5C

http://species-id.net/wiki/Heterospilus_santarosensis

Figure 106

#### Female.

Body size: 3.0–4.0 mm. Color: head yellow; stigma yellow without lateral brown stripe, flagellum yellow basally to brown apically; mesosoma brown; metasomal terga 1–3 brown to light brown, terga 4–7 honey yellow; wing veins brown, stigma brown with yellow apex; legs yellow. Head: vertex transversely costate; frons transversely costate; face rugose; temple in dorsal view narrow, width slightly less than 1/2 eye width; malar space greater than 1/4 eye height; ocell-ocular distance about twice diameter of lateral ocellus; 23–26 flagellomeres. Mesosoma: mesoscutal lobes granulate, rugose along notauli, covered by sparse setae; notauli scrobiculate, meeting at scutellum in triangular rugose area; scutellum granulate or granulate-rugose; prescutellar furrow with 3–5 cross carinae; mesopleuron granulate; precoxal sulcus scrobiculate, bordered below by distinct carina; venter granulate; propodeum with basal median areas margined, granulate-rugose, basal median carina present, areola not distinctly margined, areolar area areolate-rugose, lateral areas entirely rugose. Wings: fore wing vein r shorter than vein 3RSa, vein 1cu-a beyond vein 1M; hind wing vein SC+R present, vein M+CU shorter than vein 1M. Metasoma: first tergum longitudinally costate, apical width slightly greater than length; second tergum longitudinally costate; anterior transverse groove weak or absent; posterior transverse groove weak or absent; third tergum costate basally, smooth apically; terga 4–7 smooth; ovipositor equal to length of metasomal tergum 1.

#### Holotype female.

Top label (white, partially printed and hand written) - Costa Rica: Guanacaste [;] Santa Rosa Natl. Park [;] 300m, ex. Malaise trap [;] Site #: H-3-O [;] Dates: 2–23.iii.1986 [;] I. D. Gauld & D. Janzen; second label (red, partially printed and hand written) - HOLOTYPE [;] Heterospilus [;] santarosensis [;] P. Marsh. Deposited in ESUW.

#### Paratypes.

4 ♀♀, same data as holotype, with additional dates of 23.iii–13.iv.1986, 13.ix–4.x.1986, 12.iv–4.v.1986, and second labels of [SE] Bosque San Emilio [;] 50yr old deciduous forest [;] [C] more or less fully [;] shaded as possible, [BH] Bosque Humedo [;] mature evergreen dry forest [;] [O] in clearing, fully [;] isolated part of day, [SE] Bosque San Emilio [;] 50yr old deciduous forest [;] [O] in clearing, fully [;] isolated part of day (ESUW). 1 ♀, Costa Rica: Guanacaste [;] Santa Rosa National Pk. [;] 300m, Malaise H-2-C4 [;] regenerating woodland [;] 10 yr. old. Ian Gauld [;] 4-24.v.1986, full shade (ESUW).

#### Comments.

The rugose mesoscutum and the short and broad metasomal tergum 1 are distinctive for this species.

#### Etymology.

Named for the type locality of Santa Rosa National Park in Guanacaste Province.

**Figure 106.**
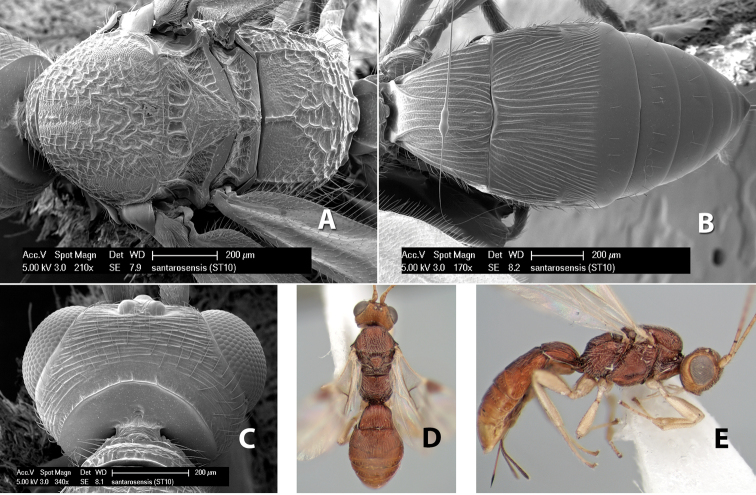
*Heterospilus santarosensis* Marsh, sp. n.: **A–C** paratype D**–E** holotype.

### Heterospilus sanvitoensis

Marsh sp. n.

http://zoobank.org/46CCF7A0-1DC6-4F04-9F08-89097BB64DA0

http://species-id.net/wiki/Heterospilus_sanvitoensis

Figure 107

#### Female.

Body size: 3.5 mm. Color: body entirely dark brown; scape honey yellow without lateral longitudinal brown stripe, flagellum brown with apical 3-5 flagellomeres white; wing veins including stigma brown; legs yellow, hind femur and tibia yellow on basal half, brown on apical half, hind tarsus brown. Head: vertex transversely costate; frons transversely costate; face granulate-areolate; temple in dorsal view narrow, width less than 1/2 eye width; malar space greater than 1/4 eye height; ocell-ocular distance 2.5 times diameter of lateral ocellus; 22 flagellomeres. Mesosoma: mesoscutal lobes granulate; notauli scrobiculate, meeting at scutellum in triangle rugose area; scutellum smooth; prescutellar furrow with 3 cross carinae; mesopleuron granulate; precoxal sulcus smooth, shorter than mesopleuron; venter granulate; propodeum with basal median areas margined, rugose, basal median carina absent, areola distinctly margined, areolar area rugose, lateral areas rugose posteriorly, granulate anteriorly, with weak apical-lateral tubercle just above hind coxa. Wings: fore wing vein r shorter than vein 3RSa, vein 1cu-a slightly beyond vein 1M; hind wing vein SC+R present, vein M+CU shorter than vein 1M. Metasoma: first tergum longitudinally costate, apical width less than length; second tergum longitudinal costate; anterior transverse groove present, sinuate; posterior transverse groove weakly indicated at least medially; third tergum smooth entirely; terga 4–7 smooth; ovipositor as long as metasoma.

#### Holotype female.

Top label (white, printed) - Costa Rica: Puntarenas [;] San Vito, Las Cruces [;] Wilson Botanical Gardens [;] 18-22.iii.1990, 1150m [;] J.S. Noyes; second label (red, partially printed and hand written) - HOLOTYPE [;] Heterospilus [;] sanvitoensis [;] P. Marsh. Deposited in ESUW.

#### Paratypes.

1 ♀, Sirena, Osa Pen. [;] VII.77 Cos. Rica [;] D. H. Janzen (AEIC).

#### Comments.

The areolate-granulate face and the smooth scutellum are distinctive for this species.

#### Etymology.

Named for the type locality of San Vito in Puntarenas Province.

**Figure 107.**
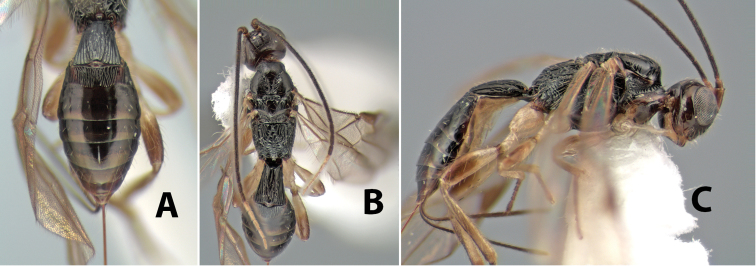
*Heterospilus sanvitoensis* Marsh, sp. n., holotype.

### Heterospilus shawi

Marsh sp. n.

http://zoobank.org/CD1E4844-51FF-4BE5-BF36-9A9F597137CD

http://species-id.net/wiki/Heterospilus_shawi

Figures 108 , 109

#### Female.

Body size: 3.5–5.0 mm. Color: head green, vertex often with longitudinal median brown stripe; scape yellow with lateral longitudinal brown stripe, flagellum brown with white annulus of 5-8 flagellomeres behind apical 3–5 brown flagellomeres; mesosoma green, often with brown longitudinal median stripe from mesoscutum to propodeum; metasoma green, terga 1 and 2 often with brown longitudinal median stripe, terga 3 and 4 usually brown basally and apically, terga 5–7 often brown medially; wing veins including stigma brown; legs green, extreme base of hind tibia (knee) brown. Head: vertex transversely costate; frons transversely costate; face rugose; temple in dorsal view broad, width about 1/2 eye width; malar space greater than 1/4 eye height; ocell-ocular distance 1.5–2.0 times diameter of lateral ocellus; 30–36 flagellomeres. Mesosoma: mesoscutal lobes granulate, often rugose along notauli, median lobe with median longitudinal scrobiculate line; notauli scrobiculate, meeting at scutellum in wide rectangular rugose area; scutellum granulate; prescutellar furrow with 3-5 cross carinae; mesopleuron granulate; precoxal sulcus smooth, shorter than mesopleuron; venter granulate; propodeum with basal median areas not distinctly margined, rugose or rugose-granulate, basal median carina absent, areola not distinctly margined, areolar area rugose, lateral areas entirely rugose. Wings: fore wing vein r shorter than vein 3RSa, vein 1cu-a beyond vein 1M; hind wing vein SC+R present, vein M+CU shorter than vein 1M. Metasoma: first tergum costate-rugose, apical width less than length; second tergum longitudinally costate; anterior transverse groove present, usually straight but often slightly sinuate; posterior transverse groove weak or absent; third tergum costate at base, granulate apically; terga 4–7 granulate; ovipositor longer than metasoma.

#### Male.

Essentially as in female; 27-28 flagellomeres, white annulus on apical 3-4 flagellomeres behind apical 2 flagellomeres.

#### Holotype female.

Top label (white, printed) - COSTA RICA-Heredia Prov. [;] La Selva Biological Station [;] 10°26'N, 84°01'W, 100m [;] Canopy fogging 36 [;] 12.xi.1994 [;] Project ALAS (F)T36; second label (red, partially printed and hand written) - HOLOTYPE [;] Heterospilus [;] shawi [;] P. Marsh. Deposited in ESUW.

#### Paratypes.

1 ♀, 3 ♂♂, same data as holotype with dates of 10.x.1994, 8.x.1994 and 2.xi.1994. 1 ♀, Costa Rica: Limon [;] Sector Cocori, 100m [;] 30km N Cariari, i.1995 [;] E. Rojas, Malaise, #4526 [;] L.N. 286000-567500 (ESUW). 1 ♀, Monumento Nacional Guayabo, A.C.A.C. [;] Amistad, Prov. Cart. COSTA RICA. 1100 [;] m. Jul 1994, G. Fonseca, LN [;] 217400_570000 #3126 (INBIO). 1 ♀, Costa Rica: Puntarenas [;] San Vito, Las Cruces [;] Wilson Botanical Gardens [;] 18-22.iii.1990, 1150m [;] J.S. Noyes (ESUW). 1 ♂, Costa Rica: Puntarenas [;] Golfo Dulce, 24km W. [;] Piedras Blancas, 200m [;] ii.1993, Paul Hanson (ESUW). 1 ♀, Costa Rica: Puntarenas [;] Pen. Osa, Rancho Quemado, [;] rio Riyito, 200m, xi-xii. [;] 1990, E. Quiros & P. Hanson, [;] ex. Malaise trap (ESUW). 1 ♀, Est Cacao, 1000-1400m, [;] Lado SO Vol. Cacao, [;] P.N.G., Prov. Guan. [;] COSTA RICA, Tp [;] Malaise, May-Jun 1991 [;] L-N-328800,375700 (INBIO). 1 ♀, COSTA RICA, Prov. Puntarenas, [;] Golfito Estacion Agujas, 300m, 08-22 [;] DIC 2000. J. Azofeifa, Manual [;] L.S._526550_276750 #61873 (INBIO). 1 ♀, COSTA RICA: Puntarenas [;] Reserva Forestal Golfo Dulce [;] 3km SW of Rincon, 10m [;] November 1992, P. Hanson [;] primary forest, Malaise trap (ESUW). 1 ♀, COSTA RICA: Puntarenas [;] RF Golfo Dulce, el 200m [;] 24km W Piedras Blancas [;] P. Hanson xii.1992 (TAMU). 2 ♀♀, PANAMA: Bocas del [;] Toro, 2km WSW [;] Chirqui Grande [;] 8°56'45"N, 82°08'13"W [;] 6.viii.1999, J. Woolley (TAMU). 1 ♀, Mexico: Veracruz [;] Los Toxilas, Darwin Trail [;] 120-130’, 19 June 1997 [;] JBWoolley, screen sweep (TAMU). 1 ♀, Guyane française, Montagne [;] de Kaw, Relais Patawa, [;] vi.2000 (Malaise) [;] A.E.I.guyane-J.Creda legs (FSAG). 1 ♂, top label - Museum Leiden [;] Canal Zone, 8km NW [;] Gamboa, Pipeline Rd. [;] 9°10'N, 79°45'W.; second label - From Luehea seemannii [;] (Tiliaecae), 4-1 [;] 26.vii.1976 [;] Y.Lubin & G. Montgomery (LEID).

#### Comments.

The unusual green color to the entire body of this species is distinctive as is the coarse rugose sculpture on the mesoscutum.

#### Etymology.

This species is named for my long time friend and colleague, Scott Shaw, in appreciation for his many years of support for this project and for the good memories of our two visits to Costa Rica and INBio.

**Figure 108.**
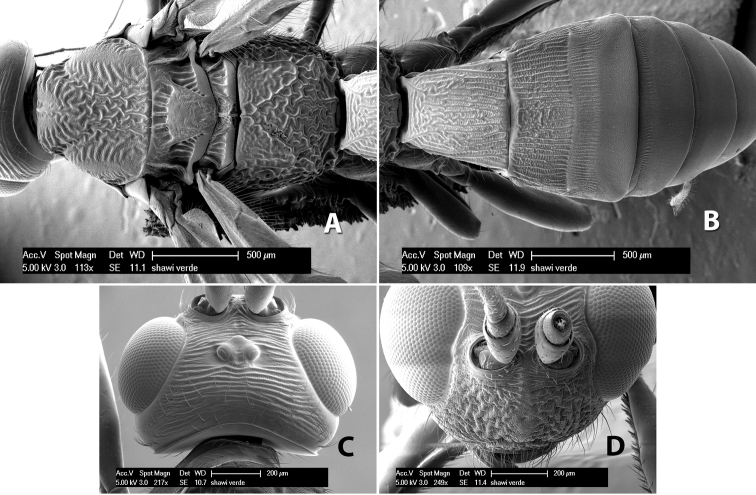
*Heterospilus shawi* Marsh, sp. n., paratype.

**Figure 109.**
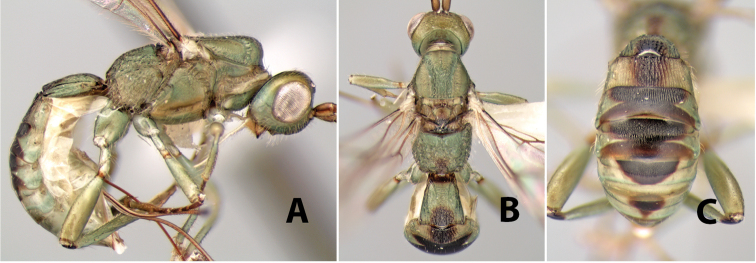
*Heterospilus shawi* Marsh, sp. n., holotype.

### Heterospilus shenefelti

Marsh sp. n.

http://zoobank.org/A2C0AB3E-C662-42BC-A28C-8C9283ABE81D

http://species-id.net/wiki/Heterospilus_shenefelti

Figure 110

#### Female.

Body size: 3.5–4.0 mm. Color: head with vertex and frons brown, face, temple and eye orbits light brown or honey yellow; scape yellow without lateral longitudinal brown stripe, flagellum brown; mesosoma dark brown; metasomal terga 1-4 dark brown, terga 5–7 yellow; wing veins including stigma brown; legs yellow. Head: vertex transversely costate; frons transversely costate; face striate; temple in dorsal view narrow, width less than 1/2 eye width; malar space greater than 1/4 eye height; ocell-ocular distance about 2.5 times diameter of lateral ocellus; 26–32 flagellomeres. Mesosoma: mesoscutal lobes granulate and dull; notauli scrobiculate, meeting at scutellum in wide rectangular rugose area; scutellum granulate; prescutellar furrow with 3 cross carinae; mesopleuron granulate; precoxal sulcus scrobiculate, shorter than mesopleuron; venter granulate; propodeum with basal median areas margined, granulate, basal median carina present, short, areola usually not distinctly margined, areolar area rugose, lateral areas entirely rugose. Wings: fore wing vein r shorter than vein 3RSa, vein 1cu-a beyond vein 1M; hind wing vein SC+R present, vein M+CU shorter than vein 1M. Metasoma: first tergum longitudinally costate, apical width equal to length; second tergum longitudinally costate, width slightly less than 4 times length; anterior transverse groove present, sinuate; posterior transverse groove present; third tergum costate basally, smooth apically; terga 4–7 smooth; ovipositor as long as length of metasomal terga 1+2.

#### Holotype female.

Top label (white, printed) - COSTA RICA: Puntarenas [;] R. F. Golfo Dulce, [;] 24km W. Piedras Blancas, [;] 200m [;] Feb. 1992, Paul Hanson; second label (red, partially printed and hand written) - HOLOTYPE [;] Heterospilus [;] shenefelti [;] P. Marsh. Deposited in ESUW.

#### Paratypes.

1 ♀, Costa Rica: Limon [;] Sector Cocori, 100m [;] 30km N Cariari, i.1995 [;] E. Rojas, Malaise #4526 [;] L.N. 286000-567500 (ESUW). 1 ♀, Costa Rica: Guanacaste [;] Est. Biol. Maritza, 600m [;] i.1997, C. Zuniga, Malaise [;] L.N. 3269000-373000 #47557 (ESUW).

#### Comments.

The striate face and short ovipositor are distinctive for this species.

#### Etymology.

Named for the late R. D. Shenefelt in recognition of his monumental work on braconid literature.

**Figure 110.**
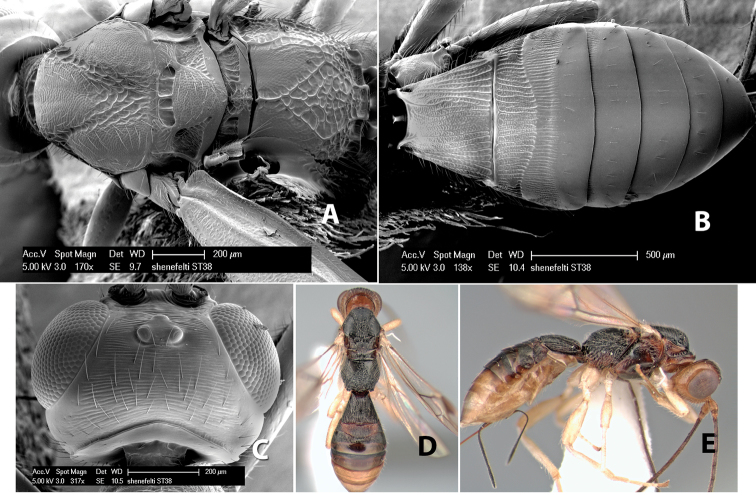
*Heterospilus shenefelti* Marsh, sp. n.: **A–C** paratype **D–E** holotype.

### Heterospilus similis

Marsh sp. n.

http://zoobank.org/52759232-1424-4191-9B86-23BE6FE57FA9

http://species-id.net/wiki/Heterospilus_similis

Figure 111

#### Female.

Body size: 2.5–4.0 mm. Color: head yellow or honey yellow; scape yellow without lateral longitudinal brown stripe; mesosoma brown to light brown, mesoscutum often lighter, sometimes with dark brown stripe along pronotum and mesopleuron; metasomal terga light brown to honey yellow; wing veins brown, stigma bicolored brown with apex and anterior edge yellow; legs yellow. Head: vertex transversely costate; frons transversely costate; face rugose; temple in dorsal view broad, not sloping behind eye, width equal to 1/2 eye width; malar space equal to 1/4 eye height; ocell-ocular distance twice diameter of lateral ocellus; 22–28 flagellomeres. Mesosoma: mesoscutal lobes granulate; notauli scrobiculate, meeting at scutellum in triangular costate area; scutellum granulate; prescutellar furrow with 3–5 cross carinae; mesopleuron granulate; precoxal sulcus scrobiculate, shorter than mesopleuron; venter granulate; propodeum with basal median areas margined, granulate, basal median carina absent or very short, areola not distinctly margined, areolar area rugose, lateral areas entirely rugose. Wings: fore wing vein r shorter than vein 3RSa, vein 1cu-a beyond vein 1M; hind wing vein SC+R present, vein M+CU greater than 1/2 vein 1M, often nearly as long. Metasoma: first tergum longitudinally costate, apical width equal to length; second tergum longitudinally costate, width about 4 times length; anterior transverse groove present, sinuate; posterior transverse groove weakly indicated or absent; third tergum costate at base, smooth apically; terga 4–7 smooth; ovipositor equal to 1/2 length of metasoma.

#### Holotype female.

Top label (white, partially printed and hand written) - Costa Rica: Guanacaste [;] Santa Rosa Natl. Park [;] 300m, ex. Malaise trap [;] Site #: (blank) [;] Dates: 18.i–8.ii.1986 [;] I.D. Gauld & D. Janzen; second label (white, printed) - [SE] Bosque San Emilio [;] 50yr old deciduous forest [;] [C] more or less fully [;] shaded as possible; third label (red, partially printed and written) - HOLOTYPE [;] Heterospilus [;] similis [;] P. Marsh. Deposited in ESUW.

#### Paratypes.

1 ♀, same data as holotype with date of 4-24.v.1986 (ESUW). 4 ♀♀, top label - Costa Rica: Guanacaste [;] Santa Rosa Natl. Park [;] 300m, ex. Malaise trap [;] Site #: SE-7-O and blank [;] Dates: 2–23.iii.1986 and 10–31.i.1987 [;] I.D. Gauld & D. Janzen; second label - [SE] Bosque San Emilio [;] 50yr old deciduous forest [;] [O] in clearing, fully [;] isolated pert of day (ESUW). 2 ♀♀, top label - Costa Rica: Guanacaste [;] Santa Rosa Natl. Park [;] 300m, ex. Malaise trap [;] Site #: 10 [;] Dates: 8–29.xi.1986 [;] I.D. Gauld & D. Janzen; second label - [BH] Bosque Humedo [;] mature evergreen dry forest [;] [C] more or less fully [;] shaded as possible (ESUW). 1 ♀, top label - Costa Rica: Guanacaste [;] Santa Rosa Natl. Park [;] 300m, ex. Malaise trap [;] Site #: H-3-O [;] Dates: 10–31.i.1987 [;] I.D. Gauld & D. Janzen; second label - [H] open regenerating [;] woodland <10 years old [;] [O] in clearing, fully [;] isolated pert of day (ESUW). 1 ♀, top label - Costa Rica: Guanacaste [;] Santa Rosa National Pk. [;] 300m, Malaise, Ian Gauld [;] 10–31.i.1987; second label - Bosque San Emilio [;] 50yr old deciduous [;] forest [;] Full Shade; third label - SE-6-C [;] 10-31.i.87 (ESUW). 1 ♀, Costa Rica, Guanacaste Pr. [;] Guan. Conservation Area [;] Santa Rosa Hdq., 200m [;] Malaise trap 22–26 VII 1997 [;] 3x night L.J. van der Ent (ESUW). 1 ♀, Costa Rica: Guanacaste [;] Est. Biol. Maritza, 600m [;] i.1997, C. Zuniga, Malaise [;] L.N. 326900-373000 #47557 (ESUW). 1 ♀, Costa Rica: Guanacaste Pr. [;] Guanacaste National Park [;] near Headquarters [;] 1-10 March 1990, J.S. Noyes (ESUW). 1 ♀, Costa Rica: Puntarenas [;] Pen. Osa, 5km. N. Pto. [;] Jimenez, 10m, iii–v. [;] 1991, P. Hanson, Malaise (ESUW). 1 ♀, Costa Rica: Puntarenas [;] Pe. Osa, Puerto Jimenez [;] 10m, July 1991, full sun, [;] grassy & weedy site [;] P. Hanson, ex. Malaise (ESUW). 1 ♀, COSTA RICA-Heredia Prov [;] La Selva Biological Station [;] 10°26'N, 84°01'W, 100m [;] Canopy fogging 31 [;] 2.xi.1994 [;] Project ALAS (FPM31) (ESUW). 4 ♀♀, S.RosaPark, Guan. [;] C. Rica 16 May, 76, 9 Mar. 77, 21 Feb. 77 and 27 Mar. 78 [;] D. H. Janzen [;] Dry Hill and Riparian (AEIC).

#### Comments.

This species is similar to *Heterospilus rhabdotus* by the color pattern but is separated by the longer hind wing vein M+SC.

#### Etymology.

The specific name is from the Latin *similis* meaning near in reference to the similarity of this species to *Heterospilus rhabdotus*.

**Figure 111.**
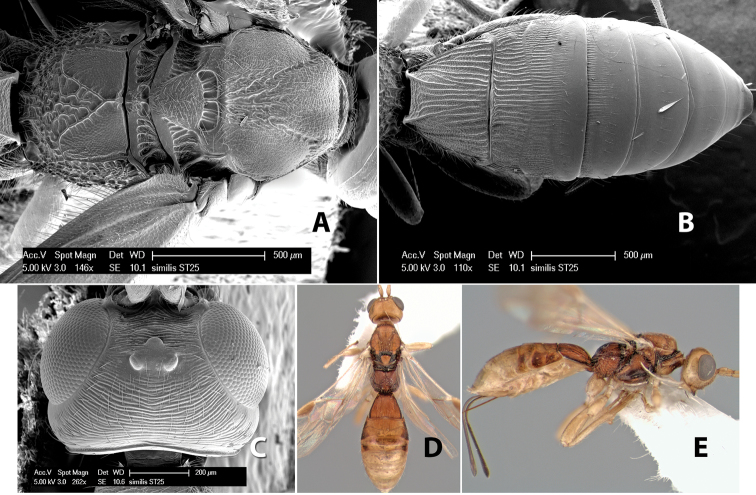
*Heterospilus similis* Marsh, sp. n.: **A–C** paratype **D–E** holotype.

### Heterospilus sinuatus

Marsh sp. n.

http://zoobank.org/20AEABE6-F88B-406E-B02A-EBB32A302D8B

http://species-id.net/wiki/Heterospilus_sinuatus

Figure 112

#### Female.

Body size: 2.5–3.5 mm. Color: body entirely honey yellow; scape yellow without lateral longitudinal brown stripe, flagellum yellow basally to brown apically; legs yellow; wing veins brown, stigma yellow. Head: vertex transversely striate; frons transversely striate; face rugose; temple in dorsal view broad, not sloping behind eye, width about 1/2 eye width; malar space less than 1/4 eye height; ocell-ocular distance about 1.5 times diameter of lateral ocellus; 19–25 flagellomeres. Mesosoma: mesoscutal lobes granulate; notauli scrobiculate, meeting at scutellum in triangular rugose area; scutellum granulate; prescutellar furrow with one distinct cross carina; mesopleuron usually smooth, often weakly granulate; precoxal sulcus smooth, shorter than mesopleuron; venter granulate; propodeum with basal median areas margined, granulate, basal median carina absent, areola not distinctly margined, areolar area rugose, lateral areas entirely rugose. Wings: fore wing vein r usually slightly shorter than vein 3RSa, occasionally equal, vein 1cu-a beyond vein 1M, vein RS+Ma distinctly curved or sinuate; hind wing vein SC+R present, vein M+CU equal in length to vein 1M. Metasoma: first tergum longitudinally costate, apical width equal to length; second tergum longitudinally costate; anterior transverse groove present, straight; posterior transverse groove present; third tergum costate basally, smooth apically; tergum 4 costate basally, smooth apically; terga 5–7 smooth; ovipositor as long as terga 1+2 combined.

#### Male.

Essentially as in female; hind wing with small stigma at base.

#### Holotype female.

Top label (white, partially printed and hand written) - Costa Rica: Guanacaste [;] Santa Rosa Natl. Park [;] 300m, ex. Malaise trap [;] Site #: (blank) [;] Dates: 4–24.v.1986 [;] I.D. Gauld & D. Janzen; second label (white, printed) - [SE] Bosque San Emilio [;] 50yr old deciduous forest [;] [C] more or less fully [;] shaded as possible; third label (red, partially printed and hand written) - HOLOTYPE [;] Heterospilus [;] sinuatus [;] P. Marsh. Deposited in ESUW.

#### Paratypes.

7 ♀♀, same data as holotype, except dates of 23.iii–13.iv.1986, 13.iv–4.v.1986, 24.v–14.vi.1986, 23.vi–13.iv.1986, 28.xii.85–18.i.1986, 14.viii–6.ix.1986, 31.i–21.ii.1987, and second labels as follows: [SE] Bosque San Emilio [;] 50yr old deciduous forest [;] [O] in clearing, fully isolated part of day; [BH] Bosque Humedo [;] mature evergreen dry forest [;] [C] more or less fully [;] shaded as possible; [BH] Bosque Humedo [;] mature evergreen dry forest [;] [O] in clearing, fully [;] isolated part of day; [H] open regenerating [;] woodland <10 years old [;] [C] more or less fully [;] shaded as possible; [H] open regenerating [;] woodland <10 year old [;] [O] in clearing, fully [;] isolated pert of day (ESUW). 1 ♀, Costa Rica, Guanacaste Pr. [;] Guan. Conservation Area [;] Santa Rosa Hdq., 200m [;] Malaise trap 26-30-VI-1997 [;] 3x night L.J. van der Ent (ESUW). 1 ♀, Costa Rica, Guanacaste Pr. [;] Guan. Conservation Area [;] Santa Rosa hdq., 200m [;] lighttrap, 7-VII-1997 [;] L.J. van der Ent (ESUW). 1 ♀, Costa Rica, Guanacaste [;] PN Guanacaste, 7km E HQ [;] near “small house” [;] 9.iii.1990, J. S. Noyes. 1 ♀, top label, Costa Rica: Guanacaste [;] Santa Rosa National Pk. [;] 300m, Malaise, Ian Gauld [;] 24.v–14.vi.1986; second label, Bosque san Emilio [;] 50yr Old deciduous [;] forest, sun (ESUW). 2 ♀♀, 2 ♂♂, top label - COSTA RICA [;] Guanacaste Prov. [;] D.H.Janzen et al. [;] #VI-20 - 1972.027 and 016; second label - Taboga, 16km. [;] SW. Canas, Savannah [;] grassland 27Jan1972 [;] Bauhinia pauletia; third label - Bruchids: [;] Gibbobruchus [;] guanacaste [;] G. cristicollis (NMNH). 1 ♀, 2 ♂♂, top label - COSTA RICA [;] Guanacaste Prov. [;] D.H.Janzen et al. [;] #VI-20 - 1972.027; second label - LaPacificaRanch ][;] 6km. N. Canas [;] Jan 20 1972; third label - Bauhinia glabra; fourth label - bruchids: [;] Caryedes [;] x-liturus [;] C. cavatus (NMNH). 1 ♀, top label - E.J.N., M. Ag.21km [;] S.Canas, Guanacaste, [;] Costa Rica, Malaise [;] Trap, VII-29-1990 (TAMU).

#### Biology.

Reared from *Bauhinia pauletia* and *Bauhinia glabra* invested with the bruchids *Gibbobruchus guanacaste*, *Gibbobruchus cristicollis*, *Caryedes x-liturus* and *Caryedes cavatus*.

#### Comments.

This species is distinguished by the distinctly curved or sinuate fore wing vein RS+Ma, the yellow body, prescutellar furrow with one cross carina and short ocell-ocular distance

#### Etymology.

The specific name is from the Latin *sinuatus*, meaning curved or bent, in reference to the distinctly curved fore wing vein RS+Ma.

**Figure 112.**
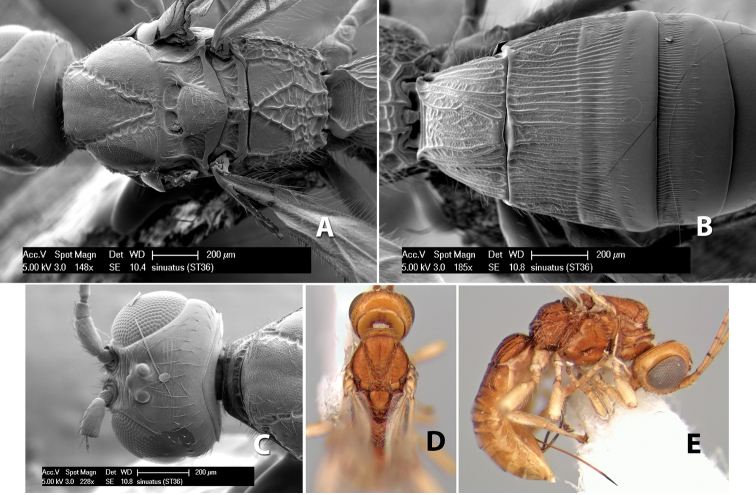
*Heterospilus sinuatus* Marsh, sp. n.: **A–C** paratype **D–E** holotype.

### Heterospilus staryi

Marsh sp. n.

http://zoobank.org/893D0F67-9F5D-450D-8B16-F38FAD952471

http://species-id.net/wiki/Heterospilus_staryi

Figure 113

#### Female.

Body size: 3.5–4.0 mm. Color: head brown, eye orbits yellow; scape yellow without lateral longitudinal brown stripe, flagellum brown with apical 5-6 flagellomeres white; mesosoma with propleuron, pronotum, mesopleuron dorsally, scutellum and propodeum dark brown, mesoscutum, mesopleuron ventrally and venter honey yellow; metasomal terga dark brown, tergum 2 with basal u-shaped yellow mark, terga 6–7 yellow; legs yellow or light brown; wing veins and stigma brown. Head: vertex transversely costate; frons transversely costate; face areolate; temple in dorsal view narrow, less than 1/2 eye width; malar space greater than 1/4 eye height; ocell-ocular distance 1.5–2 times diameter of lateral ocellus; 26–32 flagellomeres. Mesosoma: mesoscutal lobes granulate; notauli scrobiculate, meeting at scutellum in triangular costate area; scutellum smooth; prescutellar furrow with 3–5 cross carinae; mesopleuron smooth; precoxal sulcus smooth, shorter than mesopleuron; venter smooth; propodeum with basal median areas distinctly margined, areas smooth anteriorly and rugose posteriorly, basal median carina present but very short, areola not distinctly margined, areolar area rugose, lateral areas entirely rugose. Wings: fore wing vein r shorter than vein 3RSa, vein 1cu-a beyond vein 1M; hind wing vein SC+R present, vein M+CU shorter than vein 1M. Metasoma: first tergum longitudinally costate, length greater than apical width; second tergum costate, width slightly more than 3 times length; anterior transverse groove present, sinuate; posterior transverse groove absent; third tergum smooth; terga 4–7 smooth; ovipositor equal to length of metasoma.

#### Holotype female.

Top label (white, printed) - Costa Rica, Heredia [;] Puerto Viejo, 100m [;] OTS- La Selva [;] III-1991 P. Hanson; second label (red, partially printed and hand written) - HOLOTYPE [;] Heterospilus [;] staryi [;] P. Marsh. Deposited in ESUW.

#### Paratypes.

1 ♀, top label - COSTA RICA, Heredia [;] Est. Biol. La Selva, 50- [;] 150m. 10°26'N, 84°01W [;] Jun 1993 INBio-OET; second label - 2 Junio 1993 [;] Bosque secundario [;] M/16/122; third label, INBio bar code (ESUW).

#### Comments.

The species is distinguished by the u-shaped yellow mark at base of metasomal tergum 2.

#### Etymology.

Named for my colleague, Petr Stary.

**Figure 113.**
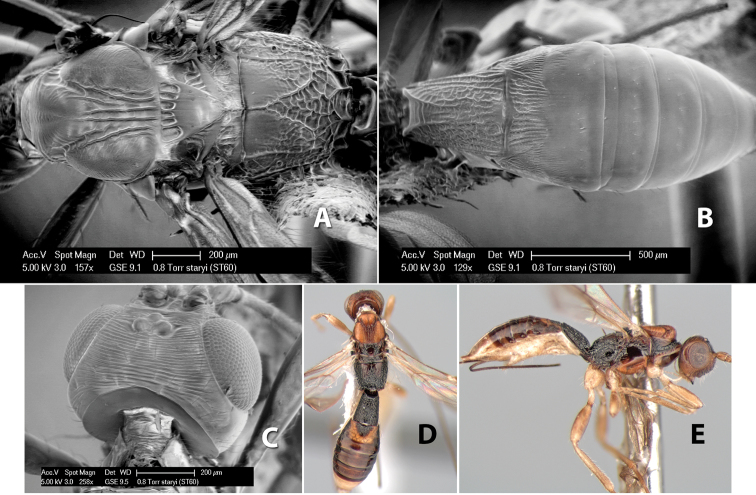
*Heterospilus staryi* Marsh, sp. n.: **A–D** holotype **E** paratype.

### Heterospilus stelfoxi

Marsh sp. n.

http://zoobank.org/967B8914-C2D3-4664-ACCA-7739DACC59C4

http://species-id.net/wiki/Heterospilus_stelfoxi

Figure 114

#### Female.

Body size: 3.0 mm. Color: head brown, face slightly lighter; scape yellow without lateral longitudinal brown stripe, flagellum entirely brown; mesosoma and metasoma dark brown or black, apical metasomal terga slightly lighter; legs yellow, hind femur yellow on basal 1/3, brown on apical 2/3; wing veins including stigma brown. Head: vertex transversely striate; frons transversely striate; face smooth; temple in dorsal view narrow, sloping behind eye, width less than 1/2 eye width; malar space greater than 1/4 eye height; ocell-ocular distance about twice diameter of lateral ocellus; 21 flagellomeres. Mesosoma: mesoscutal lobes granulate; notauli scrobiculate, meeting at scutellum in triangular rugose-costate area; scutellum smooth; prescutellar furrow with 3 cross carinae; mesopleuron smooth; precoxal sulcus scrobiculate, shorter than mesopleuron; venter weakly granulate; propodeum with basal median areas smooth, distinctly margined, basal median carina absent, areola distinctly margined, areolar area rugose, lateral areas entirely rugose. Wings: fore wing vein r shorter than vein 3RSa, vein 1cu-a beyond vein 1M; hind wing vein SC+R present, vein M+CU shorter than 1M. Metasoma: first tergum longitudinally costate, length equal to apical width; second tergum longitudinally costate, narrow, width about 4 times length; anterior transverse groove present, weakly sinuate; posterior transverse groove absent; third tergum costate at base, smooth apically; terga 4-7 smooth; ovipositor equal to length of metasoma.

#### Holotype female.

Top label (white, printed) - COSTA RICA-Heredia Prov. [;] La Selva Biological Station [;] 10°26'N, 84°01'W, 100m [;] Canopy fogging 27 [;] 20.x.1994 [;] Project ALAS (FVK27); second label (red, partially printed and hand written) - HOLOTYPE [;] Heterospilus [;] stelfoxi [;] P. Marsh. Deposited in ESUW.

#### Paratypes.

1 ♀, S.RosaPark,Guan, Guan. [;] C. Rica, 12 Jul 77 [;] D.H. Janzen [;] Dry Hill (AEIC).

#### Comments.

The species is distinguished by the sinuate anterior transverse groove on metasomal tergum 2, absence of posterior transverse groove on tergum 3, and the narrow temple.

#### Etymology.

Named for the Irish entomologist A. W. Stelfox who described many Braconidae from Ireland and England.

**Figure 114.**
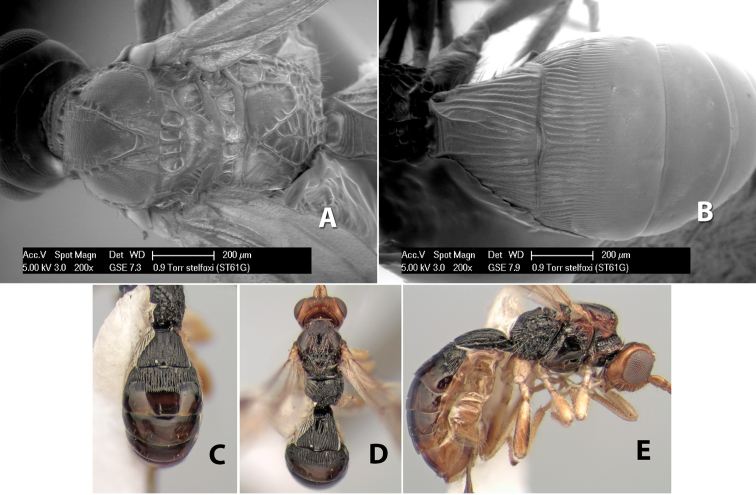
*Heterospilus stelfoxi* Marsh, sp. n., holotype.

### Heterospilus szepligetii

Marsh sp. n.

http://zoobank.org/B325741A-1349-4570-A269-464DD0A897CD

http://species-id.net/wiki/Heterospilus_szepligetii

Figure 115

#### Female.

Body size: 2.5 mm. Color: head brown; scape yellow without lateral longitudinal brown stripe, flagellum brown with apical 3–5 flagellomeres white; mesosoma dark brown; metasomal terga 1–4 dark brown, tergum 2 lighter brown, tergum 5 brown with yellow apical edge, terga 6–7 yellow; wing veins including stigma brown; legs yellow. Head: vertex transversely striate; frons transversely striate; face smooth; temple in dorsal view somewhat narrow, width slightly less than 1/2 eye width; malar space greater than eye height; ocell-ocular distance about 2.5 times diameter of lateral ocellus; 21 flagellomeres. Mesosoma: mesoscutal lobes granulate; notauli weakly scrobiculate or smooth, meeting at scutellum in triangular costate area; scutellum granulate; prescutellar furrow with 3 cross carinae; mesopleuron granulate; precoxal sulcus smooth, shorter than mesopleuron; venter granulate; propodeum with basal median areas margined, granulate, basal median carina present, areola distinctly margined, areolar area rugose, lateral areas entirely rugose. Wings: fore wing vein r shorter than vein 3RSa, vein 1cu-a beyond vein 1M; hind wing vein SC+R present, vein M+CU shorter than vein 1M. Metasoma: first tergum longitudinally costate, apical width less than length; second tergum longitudinally costate, greatest width about 4 times median length; anterior transverse groove present, straight; posterior transverse groove weakly indicated or absent; third tergum costate at base, smooth apically; terga 4–7 smooth; ovipositor as long as metasoma.

#### Holotype female.

Top label (white, printed) - Costa Rica: Cartago [;] Braulio Carillo N.P. [;] 600m, 25.iii.1990 [;] J. S. Noyes, coll.; second label (red, partially printed and hand written) - HOLOTYPE [;] Heterospilus [;] szepligetii [;] P. Marsh. Deposited in ESUW.

#### Paratypes.

1 ♀, Costa Rica: Heredia [;] BraulioCarrillo N.P. [;] 250-500m IV.10.85 [;] Henri Goulet (AEIC).

#### Comments.

The narrow metasomal tergum 2 and the white apical flagellomeres are distinctive for this species.

#### Etymology.

Named for G. V. Szépligeti who described many braconids in the early 1900s.

**Figure 115.**
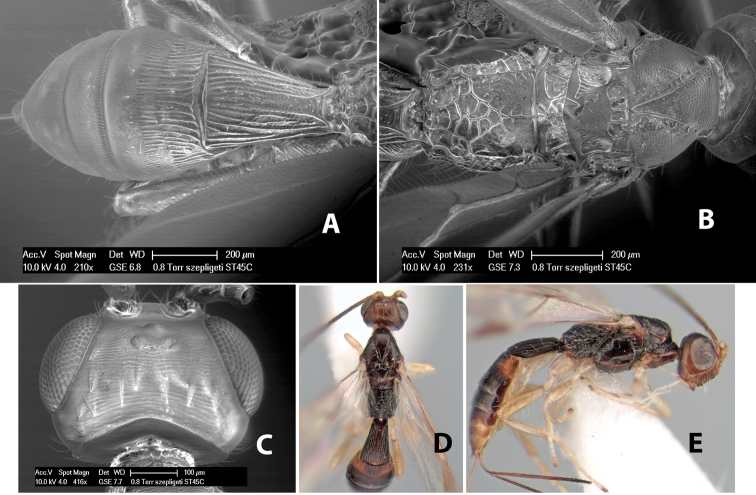
*Heterospilus szepligetii* Marsh, sp. n., holotype.

### Heterospilus tobiasi

Marsh sp. n.

http://zoobank.org/D7D420A5-440C-429E-A390-D9181DECD7B7

http://species-id.net/wiki/Heterospilus_tobiasi

Figure 116

#### Female.

Body size: 2.5–3.0 mm. Color: head yellow, vertex often brown behind ocelli; scape yellow without lateral brown stripe, flagellum yellow basally to brown apically; mesosoma brown, occasionally mesoscutum lighter brown or honey yellow; metasoma brown or dark brown, tergum 2 often lighter honey yellow medially; wing veins including stigma brown; legs yellow. Head: vertex transversely striate, sometimes weakly so only behind ocelli, smooth near eyes; frons transversely striate; face rugose or rugose-granulate; temple in dorsal view narrow, width less than 1/2 eye width; malar space equal to 1/4 eye height; ocell-ocular distance slightly less than 1.5 times diameter of lateral ocellus; 16 flagellomeres. Mesosoma: mesoscutal lobes granulate, covered with sparse short setae; notauli scrobiculate, meeting at scutellum in triangular rugose area; scutellum granulate; prescutellar furrow with 3-5 cross carinae; mesopleuron granulate; precoxal sulcus scrobiculate, shorter than mesopleuron; venter granulate; propodeum with basal median areas margined, granulate, basal median carina absent, areola not distinctly margined, areolar area areolate-rugose, lateral areas entirely rugose. Wings: fore wing vein r longer than vein 3RSa, vein 1cu-a beyond vein 1M; hind wing vein SC+R absent, vein M+CU equal in length to vein 1M. Metasoma: first tergum longitudinally costate, apical width equal to or slightly less than length; second tergum longitudinally costate; anterior transverse groove weak or absent; posterior transverse groove absent; third tergum entirely smooth; terga 4–7 smooth; ovipositor equal to combined lengths of metasomal terga 1-2.

#### Holotype female.

Top label (white, partially printed and hand written) - Costa Rica: Guanacaste [;] Santa Rosa National Pk. [;] 300m, Malaise, Ian Gauld [;] 31.i–21.ii.1987; second label (white, partially printed and hand written) - Bosque San Emilio [;] 50yr old deciduous [;] forest, full shade; third label (white, printed) - SE-8-c [;] 31.i–21.ii.87; fourth label (red, partially printed and hand written) - HOLOTYPE [;] Heterospilus [;] tobiasi [;] P. Marsh. Deposited in ESUW.

#### Paratypes.

1 ♀, top label - Costa Rica: Guanacaste [;] Santa Rosa Natl. Park [;] 300m, ex. Malaise trap [;] Site #: H-3-O [;] Dates: 21.ii–14.iii.1987 [;] I.D. Gauld & D. Janzen; second label - [H] open regenerating [;] woodland <10 years old [;] [O] in clearing, fully [;] isolated part of day (ESUW). 1 ♀, top label - Costa Rica: Guanacaste [;] Santa Rosa Natl. Park [;] 300m, ex. Malaise trap [;] Site #: (blank) [;] Dates: 10-31.i.1987 [;] I.D. Gauld & D. Janzen; second label - [H] open regenerating [;] woodland <10 years old [;] [C] more or less fully [;] shaded as possible (ESUW). 1 ♀, Costa Rica: Guanacaste Pr. [;] Guanacaste National Park [;] near Playa Naranja [;] 11 March 1990, J.S. Noyes (ESUW). 2 ♀♀, S.RosaPark, Guan. [;] C. Rica 6 June, 76 and 17 Dec. 76 [;] D. H. Janzen [;] Riparian and Dry Hill (AEIC).

#### Comments.

The fore wing vein r being longer than vein 3RSa is distinctive for this species.

#### Etymology.

Named for the late Russian braconidologist, V. I. Tobias.

**Figure 116.**
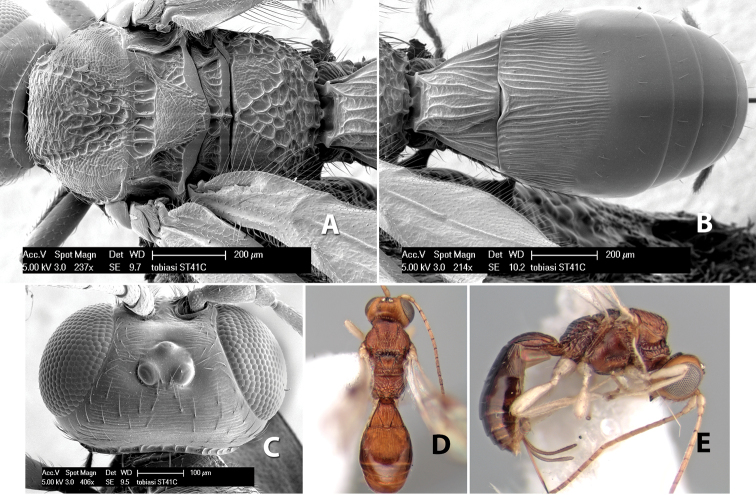
*Heterospilus tobiasi* Marsh, sp. n.: **A–D** paratype **E** holotype.

### Heterospilus tolupan

Marsh sp. n.

http://zoobank.org/85145FA9-A906-4645-84FC-9745C59E97FD

http://species-id.net/wiki/Heterospilus_tolupan

Figure 117

#### Female.

Body size: 3.5 mm. Color: head, mesosoma and metasomal terga dark brown or black, apical terga slightly lighter; scape light brown without lateral longitudinal brown stripe, flagellum brown with apical 5–7 flagellomeres white; wing veins including stigma brown; legs yellow. Head: vertex weakly striate; frons weakly striate or smooth; face smooth; temple in dorsal view narrow, width 1/2 eye width; malar space equal to eye height; ocell-ocular distance about 2.5 times diameter of lateral ocellus; 23 flagellomeres. Mesosoma: mesoscutal lobes granulate; notauli scrobiculate, meeting at scutellum in triangular costate area; scutellum granulate; prescutellar furrow with 1 cross carina; mesopleuron granulate; precoxal sulcus scrobiculate, shorter than mesopleuron; venter granulate; propodeum with basal median areas margined, granulate, basal median carina present, areola weakly margined, areolar area rugose, lateral areas entirely rugose. Wings: fore wing vein r shorter than vein 3RSa, vein 1cu-a beyond vein 1M; hind wing vein SC+R present, vein M+CU shorter than vein 1M. Metasoma: first tergum longitudinally costate, apical width about equal to length; second tergum longitudinally costate; anterior transverse groove present, straight; posterior transverse groove present; third tergum costate at base, smooth apically; terga 4–7 smooth; ovipositor as long as metasomal terga 1–2 combined.

#### Holotype female.

Top label (white, printed) - COSTA RICA, Alajuela [;] Finca San Gabriel [;] 2 0 Dos Rios, 600m [;] VIII/88, Col. Hanson; second label (red, partially printed and hand written) - HOLOTYPE [;] Heterospilus [;] tolupan [;] P. Marsh. Deposited in ESUW.

#### Paratypes.

Known only from the holotype.

#### Comments.

The granulate mesopleuron, single distinct cross carina in the prescutellar furrow, hind wing vein SC+R present and the white apical flagellomeres are distinctive for this species.

#### Etymology.

Named for the Tolupan, an indigenous people of Honduras.

**Figure 117.**
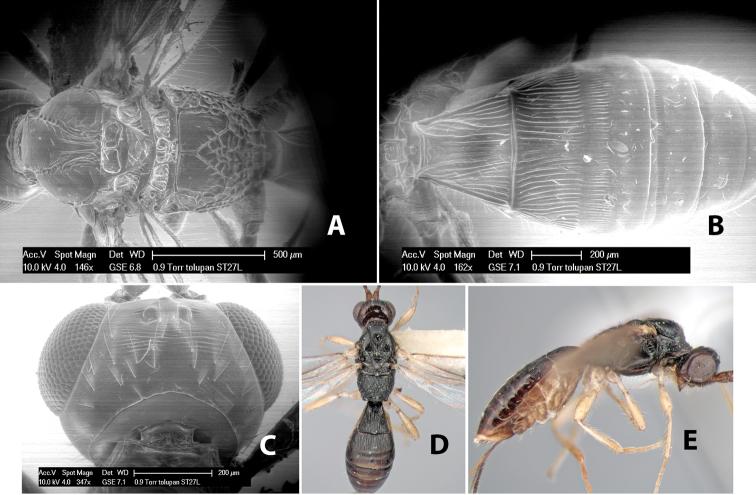
*Heterospilus tolupan* Marsh, sp. n., holotype.

### Heterospilus townesi

Marsh sp. n.

http://zoobank.org/36F02104-E682-4203-8EE7-7E56A002A12A

http://species-id.net/wiki/Heterospilus_townesi

Figure 118

#### Female.

Body size: 3.5 mm. Color: head dark brown, face and eye orbits sometimes lighter; scape honey yellow with brown lateral longitudinal stripe, flagellum brown with apical 3–4 flagellomeres before the apical 7–8 white; mesosoma dark brown, venter and propodeum often lighter; metasomal terga dark brown, terga 5–7 sometimes yellow; wing veins including stigma brown; legs yellow. Head: vertex transversely costate; frons transversely costate; face rugose; temple in dorsal view narrow, width less than 1/2 eye width; malar space equal to 1/4 eye height; ocell-ocular distance about twice diameter of lateral ocellus; 23 flagellomeres. Mesosoma: mesoscutal lobes weakly granulate and shining; notauli scrobiculate, meeting at scutellum in triangular costate area; scutellum smooth; prescutellar furrow with 3–5 cross carinae; mesopleuron weakly granulate; precoxal sulcus smooth, shorter than mesopleuron; venter weakly granulate; propodeum with basal median areas granulate and small, margined by carina and scrobiculate groove, basal median carina absent, areola not distinctly margined, areolar area areolate-rugose, lateral areas entirely rugose, propodeum with small but distinct tubercle just above hind coxa. Wings: fore wing vein r shorter than vein 3RSa, vein 1cu-a beyond vein 1M; hind wing vein SC+R present, vein M+CU shorter than vein 1M. Metasoma: first tergum longitudinally costate, apical width equal to length; second tergum nearly 4 times as wide as long, longitudinally costate; anterior and posterior transverse grooves present and indicated by row of distinct pits; third tergum costate basally, smooth apically; terga 4–7 smooth; ovipositor equal to length of metasomal terga 1 and 2 combined.

#### Holotype female.

Top label (white, printed) - COSTA RICA: Puntarenas [;] San Vito, Las Cruces [;] 1200msnm, VIII-IX 1988 [;] Coll. P. Hanson; second label (red, partially printed and hand written) - HOLOTYPE [;] Heterospilus [;] townesi [;] P. Marsh. Deposited in ESUW.

#### Paratypes.

1 ♀, Costa Rica: Guanacaste [;] Rancho Montezuma [;] 3km SE Rio Naranjo [;] 490m, Malaise trap [;] x.1994, R. G. Allen [;] L.N. 298800-418800 #5511 (ESUW).

#### Comments.

The smooth scutellum and areolate-rugose propodeum are distinctive for this species.

#### Etymology.

Named for a long time friend and world famous ichneumonologist, the late Henry Townes.

**Figure 118.**
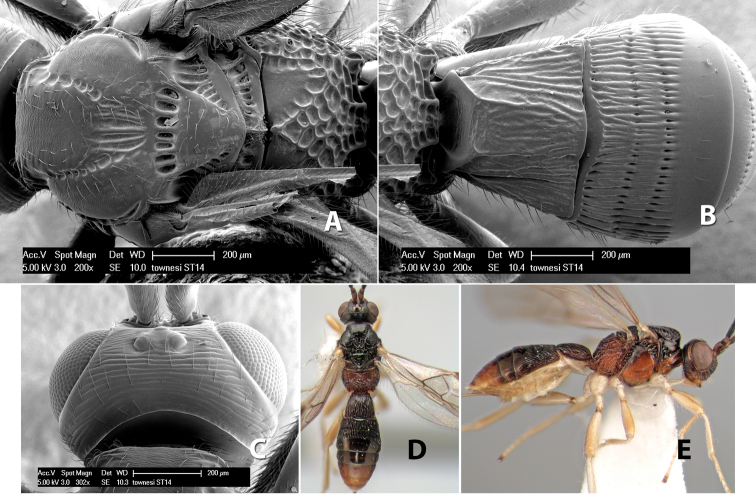
*Heterospilus townesi* Marsh, sp. n.: **A–C** paratype **D–E** holotype.

### Heterospilus turrialbaensis

Marsh sp. n.

http://zoobank.org/71213EB2-5A40-4317-8117-580912810ADA

http://species-id.net/wiki/Heterospilus_turrialbaensis

Figure 119

#### Female.

Body size: 2.0–2.5 mm. Color: head brown to dark brown; scape yellow without lateral brown stripe, flagellum yellow basally to brown apically, apical 3–5 flagellomeres white; mesosoma brown to dark brown; metasoma brown to dark brown; wing veins including stigma brown; legs yellow. Head: vertex transversely striate; frons transversely striate; face granulate; temple in dorsal view narrow, width less than 1/2 eye width; malar space greater than 1/4 eye height; ocell-ocular distance 2.5 times or greater than diameter of lateral ocellus; 20–23 flagellomeres. Mesosoma: mesoscutal lobes granulate; notauli scrobiculate, meeting at scutellum in triangular costate area; scutellum granulate; prescutellar furrow with 3 cross carinae; mesopleuron granulate; precoxal sulcus scrobiculate, shorter than mesopleuron; venter granulate; propodeum with basal median areas margined, granulate, basal median carina absent or rarely very short, areola not distinctly margined, areolar area rugose, lateral areas rugose posteriorly, granulate anteriorly. Wings: fore wing vein r shorter than vein 3RSa, vein 1cu-a beyond vein 1M; hind wing vein SC+R present, vein M+CU shorter than vein 1M. Metasoma: first tergum longitudinally costate, apical width at most equal to 1/2 length, usually less; second tergum longitudinally costate-granulate; anterior transverse groove present, straight; posterior transverse groove present; third tergum costate basally, smooth apically; terga 4–7 smooth; ovipositor equal to 1/2 length of metasoma.

#### Holotype female.

Top label (white, printed) - Costa Rica: Cartago [;] Turrialba, CATIE [;] 14-15 March 1990 [;] 700m, J.S. Noyes; second label (red, partially printed and hand written) - HOLOTYPE [;] Heterospilus [;] turrialbaensis [;] P. Marsh. Deposited in ESUW.

#### Paratypes.

1 ♀, same data as holotype (ESUW). 4 ♀♀, Costa Rica: Cartago [;] Braulio Carillo N.P. [;] 600m, 25.iii.1990 [;] J. S. Noyes, coll. (ESUW). 1 ♀, Costa Rica: Alajuela [;] Res. Biol. San Ramon [;] 800m, iv–v.1999 [;] P. Hanson, Malaise (ESUW). 1 ♀, COSTA RICA: [;] 11mi. from Turrialba [;] “Los Esperales”, C.A.T.I.E. [;] 5-II-1985 [;] P. Stansly (TAMU). 1 ♀, Costa Rica: San Jose [;] Braulio Carillo N. P. [;] 8.2km E tunnel [;] 15-V-1988 P. Hanson (TAMU).

#### Comments.

The narrow metasomal tergum 1 is distinctive for this species.

#### Etymology.

The specific name is in reference to the collection locality of Turrialba for the holotype.

**Figure 119.**
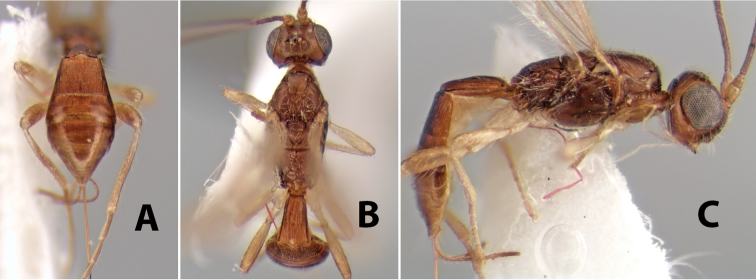
*Heterospilus turrialbaensis* Marsh, sp. n., holotype.

### Heterospilus variabilis

Marsh sp. n.

http://zoobank.org/995E5FC4-1097-4131-A26A-BC527FE55ED1

http://species-id.net/wiki/Heterospilus_variabilis

Figure 120

#### Female.

Body size: 3.5–4.0 mm. Color: head dark brown, eye orbits lighter; scape honey yellow with lateral longitudinal brown stripe, flagellum brown, apical flagellomeres white with apical 3–5 brown; mesosoma dark brown, rarely mesopleuron lighter; metasoma dark brown, apical terga sometimes lighter brown; legs bicolored with apex of femora and tarsi entirely brown; wing veins brown, stigma bicolored brown with yellow at base. Head: vertex weakly striate, often smooth; frons weakly striate, often smooth; face smooth, sometimes weakly striate below antennae; temple in dorsal view narrow, sloping behind eye, less than 1/2 eye width; malar space equal to 1/4 eye height; ocell-ocular distance about twice diameter of lateral ocellus; 23–28 flagellomeres. Mesosoma: mesoscutal lobes smooth; notauli scrobiculate, meeting at scutellum in triangular costate area; scutellum smooth; prescutellar furrow with 3 cross carinae; mesopleuron smooth; precoxal sulcus smooth, shorter than mesopleuron; venter smooth; propodeum with basal median areas distinctly margined and smooth, basal median carina absent, areola not margined, areolar area rugose, lateral areas rugose posteriorly, smooth anteriorly, area just above hind coxa with small but distinct pointed tubercle. Wings: fore wing vein r shorter than vein 3RSa, vein 1cu-a beyond vein 1M; hind wing vein SC+R present, vein M+CU shorter than vein 1M. Metasoma: first tergum longitudinally costate, apical width equal to length; second tergum longitudinally costate; anterior transverse groove present, straight; posterior transverse groove present; third tergum smooth except for costate transverse groove; terga 4–7 smooth; ovipositor equal to length of metasoma.

#### Holotype female.

Top label (white, printed) - Costa Rica: Limon [;] 30km N Cariari, 100m [;] Sector Cocori, Malaise [;] iii.1995, E. Rojas #4524 [;] L.N. 286000-567500; second label (red, partially printed and hand written) - HOLOTYPE [;] Heterospilus [;] variabilis [;] P. Marsh. Deposited in ESUW.

#### Paratypes.

1 ♀, COSTA RICA, Limon [;] 16Km W Guápiles [;] 400m, III/1989 [;] col. Paul Hanson (ESUW). 1 ♀, Costa Rica: Puntarenas [;] R.F. Golfo Dulce, [;] 3km. SW. Rincon, 10m [;] Oct. 1991, Paul Hanson (ESUW). 1 ♀, COSTA RICA: Puntar [;] Golfo Dulce, 3km [;] S.W. Rincon, 10m [;] IX-XI 1989, Hanson (ESUW). 1 ♀, Costa Rica: Puntarenas [;] ACO, Golfito, PN Corcovado [;] Est. Agujas, Charcos, 600m [;] 17.iv–16.v.1999, J. Azofeifa [;] L.S. 276350-523500 #52776 (ESUW). 1 ♀, COSTA RICA: Puntarenas [;] San Vito, Estac. Biol. [;] Los Altures 1500m [;] iv.1992 P. Hanson (TAMU).

#### Comments.

This species is distinguished by the bicolored stigma and the white band on the apical flagellomeres.

#### Etymology.

The specific name is from the Latin *variabilis* in reference to the vertex sculpture being weakly striate or smooth.

**Figure 120.**
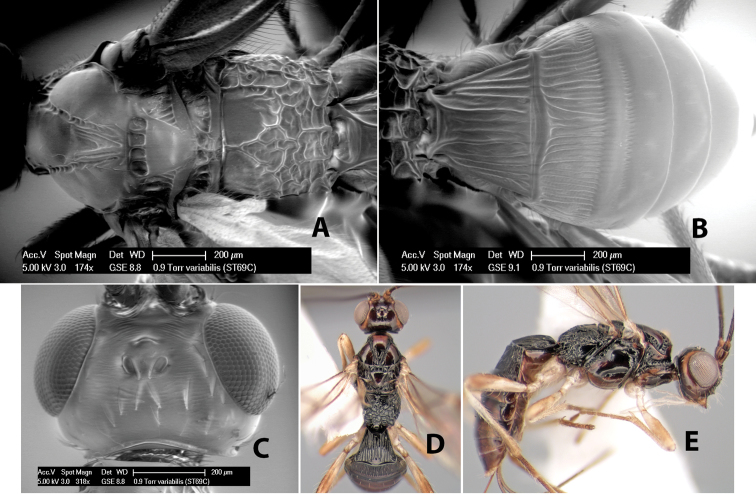
*Heterospilus variabilis* Marsh, sp. n., holotype.

### Heterospilus vierecki

Marsh sp. n.

http://zoobank.org/FC70A20C-5CEC-4CBD-9689-777D925389E3

http://species-id.net/wiki/Heterospilus_vierecki

Figure 121

#### Female.

Body size: 3.0–3.5 mm. Color: head with vertex brown, face yellow, scape yellow without lateral longitudinal brown stripe, flagellum mostly brown; mesosoma brown, middle mesoscutal lobe and propodeum lighter; metasomal terga 1 and 3 brown, 2 and 4–7 yellow; legs yellow; wing veins brown, stigma entirely brown. Head: vertex transversely costate; frons transversely costate; face striate medially, often smooth near eyes; temple in dorsal view narrow, sloping behind eye, less than 1/2 eye width; malar space greater the 1/4 eye height; ocell-ocular distance 2.5 times diameter of lateral ocellus; 19–25 flagellomeres. Mesosoma: mesoscutal lobes smooth or very weakly granulate; notauli scrobiculate, meeting before prescutellar furrow in triangular rugose area; scutellum smooth; prescutellar furrow with 3 cross carinae; mesopleuron smooth; precoxal sulcus scrobiculate, shorter than mesopleuron length; venter smooth; propodeum with basal median areas distinctly margined, basal median areas smooth or weakly granulate, basal median carina absent or very short, areola distinctly margined, areolar area rugose, lateral areas entirely rugose. Wings: fore wing vein r shorter than vein 3RSa, vein 1cu-a beyond vein 1M; hind wing vein SC+R present, vein M+CU equal to or slightly shorter than vein 1M. Metasoma: first tergum length equal to apical width, costate, sometimes rugose medially; second tergum apical width 3 or less times length; anterior transverse groove present, straight; posterior transverse groove present but often weak; third tergum costate at base, smooth at apex; terga 4–7 smooth; ovipositor longer than metasoma.

#### Holotype female.

Top label (white, partially printed and hand written - Costa Rica: Guanacaste [;] Santa Rosa Natl. Park [;] 300m, ex. Malaise trap [;] Site #: H-1-O [;] Dates: 8–29.xi.1986 [;] I.D. Gauld & D. Janzen; second label (white, printed) - [H] open regenerating [;] woodland <10 years old [;] [O] in clearing, fully [;] isolated part of day; third label (red, partially printed and hand written) - HOLOTYPE [;] Heterospilus [;] vierecki [;] P. Marsh. Deposited in ESUW.

#### Paratypes.

1 ♀, same data as holotype except, first label, site # 8H-12-C, dates of 8.ii–2.iii.1986 and second label, [BH] Bosque Humedo [;] mature evergreen dry forest [;] [C] more or less fully [;] shaded as possible (ESUW). 1 ♀, first label - Costa Rica: Guanacaste [;] Santa Rosa National Pk. [;] 300m, Malaise. Ian Gauld [;] 31.i–21ii.1987, second label - Bosque Humedo [;] mature proportion [;] evergreen species [;] Sun (ESUW). 8 ♀♀, S.RosaPark, Guan. [;] C. Rica, various date from September 77 to February 78 [;] D.H. Janzen [;] Dry Hill and Riparian (AEIC).

#### Comments.

This species is distinguished by the smooth scutellum, nearly smooth mesoscutal lobes, the ovipositor being longer than the metasoma and the brown antennae.

#### Etymology.

This species is named for H. L. Viereck who described many braconids in the early 1900s.

**Figure 121.**
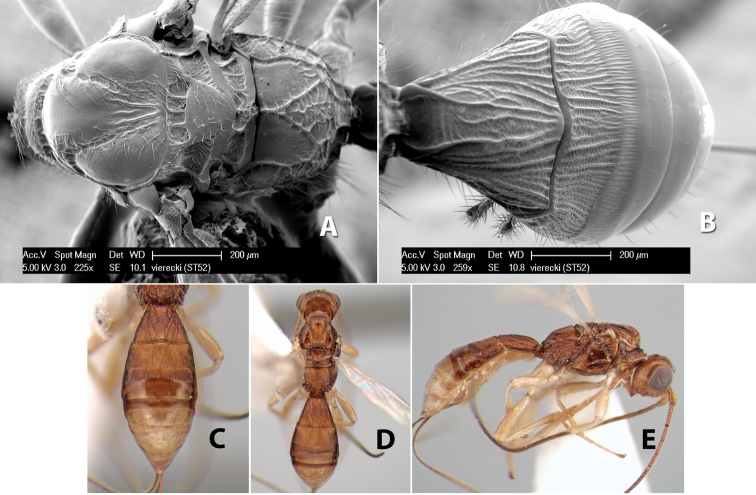
*Heterospilus vierecki* Marsh, sp. n.: **A–B** paratype **C–E** holotype.

### Heterospilus vittatus

Marsh sp. n.

http://zoobank.org/6FF49C04-93B9-4111-A872-74ECD35AFE5A

http://species-id.net/wiki/Heterospilus_vittatus

Figure 122

#### Female.

Body size: 4.5 mm. Color: head with vertex brown, frons, face, malar space and eye orbits honey yellow; scape honey yellow without lateral longitudinal brown stripe, flagellum brown; mesosoma honey yellow with brown along notauli, along pronotal groove, on scutellum and metascutum, on propodeal areola, dorsally on mesopleuron and apical-laterally on propodeum; metasoma honey yellow, terga 1 and 2 brown medially, terga 3–6 brown basally; legs yellow; wing veins including stigma brown. Head: vertex transversely costate; frons costate; face granulate; temple in dorsal view narrow, sloping behind eye, less than 1/2 eye width; malar space greater than 1/4 eye height; ocell-ocular distance about twice diameter of lateral ocellus; 33–37 flagellomeres. Mesosoma: mesoscutal lobes granulate; notauli scrobiculate, meeting at scutellum in triangular rugose area; scutellum granulate; prescutellar furrow with 3 cross carina; mesopleuron granulate; precoxal sulcus smooth, shorter than mesopleuron; venter granulate; propodeum with basal median areas margined, granulate, basal median carina present, areola not distinctly margined, areolar area rugose, lateral areas granulate. Wings: fore wing vein r shorter than vein 3RSa, vein 1cu-a beyond vein 1M; hind wing vein SC+R present, vein M+CU shorter than vein 1M. Metasoma: first tergum longitudinally costate-granulate; second tergum costate-granulate; anterior transverse groove present, sinuate; posterior transverse groove weakly indicated; third tergum entirely granulate; terga 4–7 granulate; ovipositor longer than metasoma.

#### Holotype female.

Top label (white, printed) - COSTA RICA-Heredia Prov. [;] La Selva Biological Station [;] 10°26'N, 84°01'W, 100m [;] Canopy fogging 31 [;] 2.xi.1994 [;] Project ALAS(FPM31); second label (red, partially printed and hand written) - HOLOTYPE [;] Heterospilus [;] vittatus [;] P. Marsh. Deposited in ESUW.

#### Paratypes.

Known only from the holotype.

#### Comments.

The color of this species is distinctive, honey yellow with lateral brown stripes along mesosoma and medially on metasomal terga 1–2.

#### Etymology.

The specific name is from the Latin *vittatus* meaning decorated with ribbons or stripes in reference to the brown stripes laterally on the mesosoma and medially on metasomal terga 1–2.

**Figure 122.**
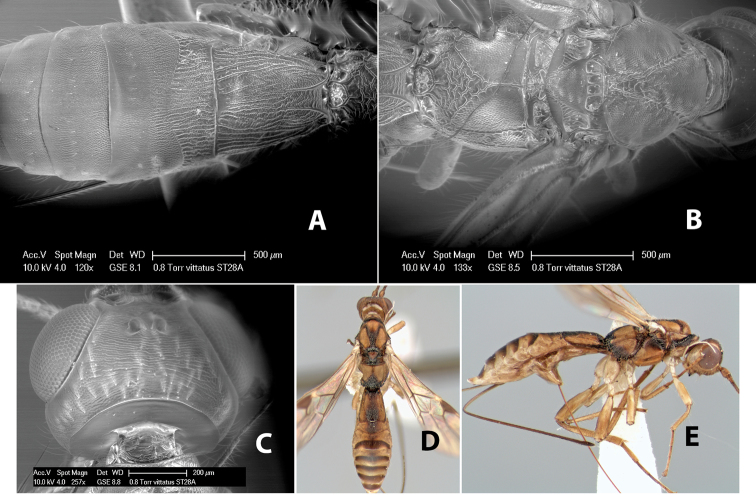
*Heterospilus vittatus* Marsh, sp. n., holotype.

### Heterospilus wesmaeli

Marsh sp. n.

http://zoobank.org/0A10F882-6486-4AC6-BD80-1633E938BB1A

http://species-id.net/wiki/Heterospilus_wesmaeli

Figure 123

#### Female.

Body size: 3.5–4.0 mm. Color: head yellow; scape yellow without lateral longitudinal brown stripe, flagellum brown; mesosoma dark brown, light brown or yellow along notauli, propleuron and precoxal sulcus; metasomal tergum 1 dark brown, tergum 2 brown medially and laterally with yellow lines medio-laterally, sometimes yellow laterally, terga 3–4 brown, sometimes yellow laterally, terga 5–7 yellow or honey yellow; wing veins including stigma brown; legs yellow, hind tibia yellow with extreme base (knee) dark brown and apical half light brown, tarsus brown. Head: vertex transversely costate; frons transversely costate; face smooth or weakly granulate; temple in dorsal view narrow, less than 1/2 eye width; malar space equal to eye height; ocell-ocular distance twice diameter of lateral ocellus; 26–28 flagellomeres. Mesosoma: mesoscutal lobes granulate; notauli scrobiculate, meeting at scutellum in wide rectangular rugose area; scutellum weakly granulate; prescutellar furrow with 3–5 cross carinae; mesopleuron weakly granulate, partially nearly smooth; precoxal sulcus smooth, shorter than mesopleuron; venter weakly granulate; propodeum with basal median areas margined, granulate, basal median carina present, areola not distinctly margined, areolar area rugose, lateral areas entirely rugose. Wings: fore wing vein r shorter than vein 3RSa, vein 1cu-a beyond vein 1M; hind wing vein SC+R present, vein M+CU equal to or longer than vein 1M. Metasoma: first tergum longitudinally costate-rugose, apical width about 3/4 length; second tergum longitudinally costate; anterior transverse groove present, sinuate; posterior transverse groove absent; third tergum costate at base, smooth at apex; terga 4–7 smooth; ovipositor as long as metasoma.

#### Holotype female.

Top label (white, printed) - Costa Rica: San Jose [;] Zurqui de Moravia [;] vi.1990, 1600m [;] Paul Hanson; second label (red, partially printed and hand written) - HOLOTYPE [;] Heterospilus [;] wesmaeli [;] P. Marsh. Deposited in ESUW.

#### Paratypes.

1 ♀, Costa Rica, Carthago Pr. [;] La Cangreja, 1960m [;] 1991:x, P. Hanson (ESUW).

#### Comments.

This species is distinguished by the nearly smooth face, brown knee on the hind tibia and the wide rugose area where the notauli meet.

#### Etymology.

Named for C. Wesmael who provided some of the early studies of Braconidae in the early 1800s, particularly for Belgium.

**Figure 123.**
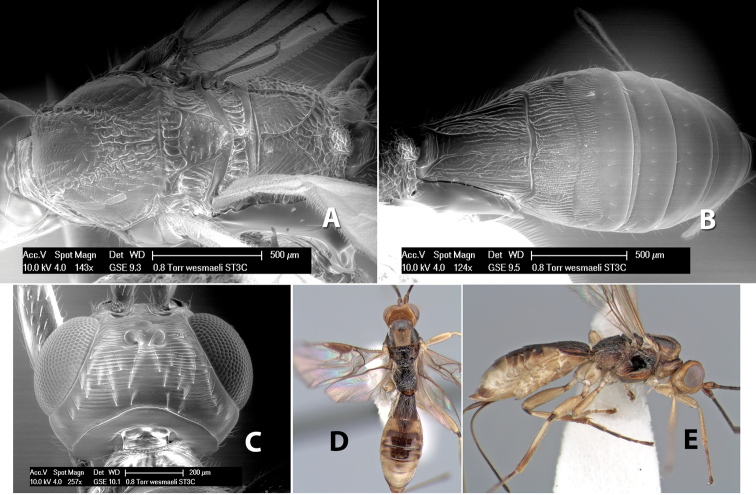
*Heterospilus wesmaeli* Marsh, sp. n., holotype.

### Heterospilus whartoni

Marsh sp. n.

http://zoobank.org/1507222C-96C3-4516-8AC0-932C06EAACC1

http://species-id.net/wiki/Heterospilus_whartoni

Figure 124

#### Female.

Body size: 3.5–4.5 mm. Color: head dark brown; scape honey yellow without lateral brown stripe, flagellum brown with apical 10–12 flagellomeres white; mesosoma dark brown, occasionally mesoscutum lighter brown medially; metasoma usually dark brown, occasionally terga 4–7 lighter; wing veins brown, stigma honey yellow; legs yellow. Head: vertex and frons strongly circularly costate around ocelli; face granulate; temple in dorsal view narrow, width less than 1/2 eye width; malar space greater than 1/4 eye height; ocell-ocular distance usually 2.5 times diameter of lateral ocellus, occasionally greater; 26–30 flagellomeres. Mesosoma: mesoscutal lobes granulate, median lobe usually with median longitudinal shallow groove; notauli scrobiculate, meeting at scutellum in triangular rugose area; scutellum smooth; prescutellar furrow with 3 cross carinae; mesopleuron weakly granular, often nearly smooth just above precoxal sulcus; precoxal sulcus scrobiculate, shorter than mesopleuron; venter granulate; propodeum with basal median areas margined, granulate, basal median carina present, areola not distinctly margined, areolar area rugose, lateral areas rugose posteriorly, granulate anteriorly. Wings: fore wing vein r shorter than vein 3RSa, vein 1cu-a beyond vein 1M; hind wing vein SC+R present, vein M+CU shorter than vein 1M. Metasoma: first tergum longitudinally costate, apical width equal to length; second tergum longitudinally costate; anterior transverse groove present, straight; posterior transverse groove present; third tergum costate at base, smooth apically; terga 4–7 smooth; ovipositor equal to 1/2 length of metasoma.

#### Holotype female.

Top label (white, printed) - Costa Rica: Puntarenas [;] San Vito, Estac. Biol. [;] Las Alturas, 1500m [;] xi.1991, Paul Hanson; second label (red, partially printed and hand written) - HOLOTYPE [;] Heterospilus [;] whartoni [;] P. Marsh. Deposited in ESUW.

#### Paratypes.

4 ♀♀, same data as holotype with additional dates of i.1992 and 15-31 Oct. 1991 (ESUW). 11 ♀♀, Costa Rica: Puntarenas [;] San Vito, Estac. Biol. [;] Las Alturas, 1500m [;] iv.1992, v.1992 and vi.1992, Forest border and in the forest [;] Malaise, Paul Hanson (ESUW, TAMU). 2 ♀♀, Costa Rica: Puntarenas [;] San Vito, Estac. Biol. [;] Las Alturas, 1500m [;] vi.1992, traps #1 + #2, [;] Malaise, Paul Hanson (ESUW). 1 ♀, Costa Rica: Puntarenas [;] San Vito, Las Cruces [;] Wilson Botanical Gardens [;] 18–22.iii.1990, 1150m [;] J.S. Noyes (ESUW). 2 ♀♀, Costa Rica: Puntarenas [;] Zona Protectora Las Tablas [;] 1Km NE de Sitio Portones [;] Camino a Tables, 1530m [;] 30.viii–5.ix.1995, M. Chinchilla [;] L.S. 320100-596800 #7458 [;] Malaise trap (ESUW). 1 ♀, Costa Rica: Puntarenas [;] Send. ac. Pittier, 1800–2000m [;] 1Km N. de la Est. Malaise [;] 13.ix–13.x.1996. A.M. Maroto [;] L.S. 331800-577400 #44868 (ESUW). 2 ♀♀, top label - Costa Rica: Guanacaste [;] W. side Volcan Orosi [;] Estac. Maritza, 600m; second label - GHP Biodiversity Survey [;] 1989, Malaise trap [;] L-N-326900-373000 #6834 (ESUW). 1 ♀, top label - Costa Rica: Puntarenas [;] Buenos Aires [;] Sendero Los Gigantes [;] Est. Altamira, 1450m; second label - 3–22 February 2000 [;] D. Rubi, Amarilla [;] LS 331700-572200 [;] # 54808 (ESUW). 1 ♀, Costa Rica: Guanacaste [;] Est. Cacao, 1000-1150m [;] ix.1996, I. Villegas, Malaise [;] L.N. 323150-375500 #47559 (ESUW). 2 ♀♀, top label - COSTA RICA, Heredia [;] Est. Biol. La Selva. 50- [;] 150m, 10°26'N, 84°01'W [;] Mar and May 1993, INBio-OET; second label - 15 Marzo and 18 Mayo 1993 [;] Bosque primario and secundario [;] M/05/100 and M/11/026 (ESUW). 1 ♀, COSTA RICA: La Selva [;] 2.ii.1994 [;] J. Longino [;] (M.14.344) (ESUW). 1 ♀, COSTA RICA, Alajuela [;] Finca San Gabriel [;] 2 0 Dos Rios, 400m [;] VIII/88, Col. Hanson (ESUW). 2 ♀♀, COSTA RICA: *Punt-* [;] *arenas*, Las Alturas, [;] 1600m, 10–13[0].vi.1998 [;] Brown & Berezovskiy: [;] Mal. Trp. #2: for. Edge (AEIC). 1 ♀, Est. Altamira, Buenos Aires, Prov. Punta. [;] COSTA RICA. 15 Set–14 Oct 1993. R. [;] Delgado, L S 572100_331700 #2370 (INBC). 1 ♀, MUSEO DE INSECTOS UNIVERSIDAD DE COSTA RICA [;] COSTA RICA PROV: Alajuela [;] Faldas Volcan [;] Tenorio. [;] 700m, 17.iv.1988 [;] Gonzales - Soto (MICR).

#### Comments.

The circularly costate vertex, the dark brown vertex and the smooth scutellum are distinctive for this species.

#### Etymology.

Named for my long time friend and fellow braconidologist, Robert Wharton.

**Figure 124.**
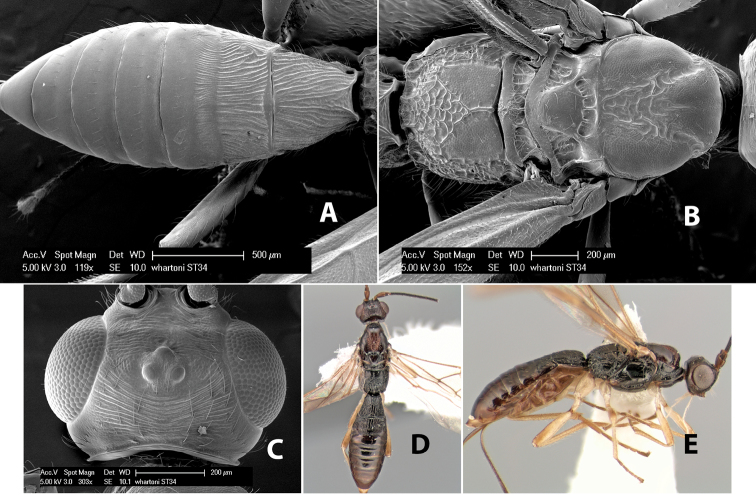
*Heterospilus whartoni* Marsh, sp. n.: **A–D** paratype; E, holotype.

### Heterospilus whitfieldi

Marsh sp. n.

http://zoobank.org/47F16954-573B-431C-BB7B-AE1608E91F06

http://species-id.net/wiki/Heterospilus_whitfieldi

Figure 125

#### Female.

Body size: 2.0–3.0 mm. Color: body usually brown to dark brown, vertex sometimes darker than face, apical metasomal terga often lighter than basal terga; scape and flagellum brown; legs yellow, hind coxa and femur brown; wing veins brown, stigma yellow. Head: vertex weakly striate, often striae present only behind ocelli; frons usually smooth, sometimes weakly striate; face smooth; temple in dorsal view broad, not sloping behind eye, width about equal to with of eye; malar space greater than 1/4 eye height; ocell-ocular distance about 2.5 times diameter of lateral ocellus; 19–25 flagellomeres. Mesosoma: mesoscutal lobes granulate, long yellow hair along notauli; notauli scrobiculate, meeting at scutellum in triangular rugose area; scutellum smooth, often hairy; prescutellar furrow with one cross carina; mesopleuron smooth, densely hair at subalar area and along posterior edge; precoxal sulcus smooth, shorter than mesopleuron; venter smooth; propodeum with basal median areas distinctly margined, smooth, basal median carina present, areola distinctly margined, areolar area weakly rugose or often unsculptured and smooth, lateral areas rugose, densely hairy. Wings: fore wing vein r slightly shorter or equal in length to vein 3RSa, vein 1cu-a beyond vein 1M; hind wing vein SC+R present, vein M+CU as long as vein 1M. Metasoma: first tergum rugose or rugose-costate, apical width slightly greater than length, median raised area distinctly margined on each side; second tergum longitudinally costate; anterior transverse groove present, straight; posterior transverse groove present; third tergum costate basally, smooth apically; terga 4–7 smooth; ovipositor equal to length of terga 1+2.

#### Holotype female.

Top label (white, partially printed and hand written) - Costa Rica: Guanacaste [;] Santa Rosa Natl. Park [;] 300m, ex. Malaise trap [;] site #: (blank) [;] Dates: 12.iv–4.v.1986 [;] I.D. Gauld & D. Janzen; second label (white, printed) - [SE] Bosque San Emilio [;] 50yr old deciduous forest [;] [O] in clearing, fully [;] isolated part of day; third label (red, partially printed and hand written) - HOLOTYPE [;] Heterospilus [;] whitfieldi [;] P. Marsh. Deposited in ESUW.

#### Paratypes.

4 ♀♀, same data as holotype with additional sites of SE-5-O and SE-7-O, and dates of 28.xii.85–18.i.1986, 6–29.ix.1986 and 7–28.xii.1985 (ESUW). 14 ♀♀, top label - Costa Rica: Guanacaste [;] Santa Rosa Natl. Park [;] 300m, ex. Malaise trap [;] Site #: SE-6-C and SE-8-C [;] Dates: 5–26.x.1985, 7–28.xii.1985, 2–23.iii.1986, 31.i–21.ii.1987, 23.iii–13.iv.1986, 26.x–16.xi.1985, 29.xi–20.xii.1986 and 18.x–8.xi.1986 [;] I.D. Gauld & D. Janzen; second label - [SE] Bosque San Emilio [;] 50yr old deciduous forest [;] [C] more or less fully [;] shaded as possible (ESUW). 13 ♀♀, top label - Costa Rica: Guanacaste [;] Santa Rosa Natl. Park [;] 300m, ex. Malaise trap [;] Site #: H-1-O, H-3-O, H-2-O and blank [;] Dates: 23.iii–13.iv.1986, 21.ii–14.iii.1987, 14.viii–6.ix.1986, 14.vi–5.vii.1986, 26.x–16.xi.1985, 20.xii.86–10.i.1987, 2–23.iii.19864–24.v.198618.x–8.xi.1986 and 13.iv–4.v.1986 [;] I.D. Gauld & D. Janzen; second label - [H] open regenerating [;] woodland <10 years old [;] [O] in clearing, fully [;] isolated part of day (ESUW). 13 ♀♀, top label - Costa Rica: Guanacaste [;] Santa Rosa Natl. Park [;] 300m, ex. Malaise trap [;] Site #: H-4-C, H-2-C and blank [;] Dates: 4–24.v.1986, 2–23.iii.1986, 18.x–8.xi.1986, 13.iv–4.v.1986, 5026.vii.1986, 14.viii–6.ix.1986, 23.iii–13.iv.1986 and 20.xii.86–10.i.1987 [;] I.D. Gauld & D. Janzen; second label - [H] open regenerating [;] woodland <10 years old [;] [C] more or less fully [;] shaded as possible (ESUW). 23 ♀♀, top label - Costa Rica: Guanacaste [;] Santa Rosa Natl. Park [;] 300m, ex. Malaise trap [;] Site #: BH-4-O, BH-9-O, BH-11-O and blank [;] Dates: 8.ii–2.iii.1986, 2–23.iii.1986, 4–24.v.1986, 18.i–8.ii.1986, 14.vi–5.vii.1986, 2–23.iii.1986, 18.i–8.11.1986, 24.v–14.vi.1986, 13.iv–4.v.1986, 29.xi–20.xii.1986 and 20.xi.86–10.i.1987 [;] I.D. Gauld & D. Janzen; second label - [BH] Bosque Humedo [;] mature evergreen dry forest [;] [O] in clearing, fully [;] isolated part of day (ESUW). 30 ♀♀, top label - Costa Rica: Guanacaste [;] Santa Rosa Natl. Park [;] 300m, ex. Malaise trap [;] Site #: BH-10-C, BH-12-C and blank [;] Dates: 24.v–14.vi.1986, 8.ii–2.iii.1986, 23.ii–13.iv.1986, 29.xi–20.xii.1986, 18.i–8.ii.1986, 18.x–8.xi.1986, 5–26.vii.1986, 14.vi–5.vii.1986, 2–23.iii.1986, 13.iv–4.v.1986, 16.xi-t.xii.1985, 6–27.ix.1986, 7–28.xii.1985 and 4–24.v.1986 [;] I.D. Gauld & D. Janzen; second label - [BH] Bosque Humedo [;] mature evergreen dry forest [C] more or less fully [;] shaded as possible (ESUW). 2 ♀♀, top label - Costa Rica: Guanacaste [;] Santa Rosa National Pk. [;] 300m, Malaise, Ian Gauld [;] 18.x–8.xi.1986; second label - Bosque San Emilio [;] 50yr Old deciduous [;] Forest [;] Full Shade; third label - SE-8-C [;] 18.x–8.xi.86 (ESUW). 1 ♀, top label - Costa Rica: Guanacaste [;] Santa Rosa National Pk. [;] 300m, Malaise, Ian Gauld [;] 6–27.ix.1986; second label - Bosque San Emilio [;] 50yr Old deciduous [;] Forest [;] Full Shade; third label - SE-8-C [;] 6–27.ix.86 (ESUW). 1 ♀, top label - Costa Rica: Guanacaste [;] Santa Rosa National Pk. [;] 300m, Malaise, Ian Gauld [;] 31.i–21.ii.1987; second label - Bosque San Emilio [;] 50yr Old deciduous [;] Forest [;] Full Shade; third label - SE-8-C [;] 31.i–21.ii.87 (ESUW). 13 ♀♀, top label - Costa Rica: Guanacaste [;] Santa Rosa National Pk. [;] 300m, Malaise, Ian Gauld [;] 31.i–21.ii.1987; second label - Bosque Humedo [;] Mature dry forest [;] high proportion [;] Evergreen species [;] Full Shade; third label - BH-10-C [;] 31.i–21.ii.87 (ESUW). 3 ♀♀, top label - Costa Rica: Guanacaste [;] Santa Rosa National Pk. [;] 300m, Malaise, Ian Gauld [;] 10–31.I.1987; second label - Bosque Humedo [;] Mature dry forest [;] high proportion [;] Evergreen species [;] Sun; third label - BH-11-O [;] 10–31.i.87 (ESUW). 1 ♀, top label - Costa Rica: BH-10-C [;] Guanacaste Province [;] Santa Rosa Natl. Pk. [;] 300m, (dry season) [;] 10–31 January 1987; second label - Bosque Humedo, mature [;] dry forest with high [;] proportion evergreen [;] species, fully shaded [;] Townes style Malaise [;] Ian Gauld coll. (ESUW). 1 ♀, Costa Rica: Guanacaste [;] Santa Rosa Natl. Park [;] 300m, ex. Malaise trap [;] Site #: 9 and SE-8-C [;] Dates: 4–24.v.1986 [;] I.D. Gauld & D. Janzen (ESUW). 2 ♀♀, Costa Rica, Guanacaste Pr. [;] Guan, Conservation Area [;] Santa Rosa Hdq., 200m [;] Malaise trap 22–26 VII 1997 and 3–8 VII 1987 [;] 3x night L.J. van der Ent (ESUW). 1 ♀, Costa Rica: Guanacaste, ACT [;] Bagaces, P.N. Palo Verde [;] Sec. P. Verde, 0–50m [;] Extremo E. Campo Aterrizaje [;] Malaise trap #53259 [;] 16.vii–17.viii.1999, I. Jimenez [;] L.N. 260952–385020 (ESUW). 1 ♀, Costa Rica: Guanacaste, ACT [;] Bagaces, P.N. Palo Verde [;] Sector Palo Verde [;] 500 NW de la Est, 40m [;] 4.vi–6.vii.1999, I. Jimenez [;] L.N. 260952–385020 #52849 [;] Malaise trap (ESUW). 2 ♀♀, COSTA RICA: [;] San Jose [;] Ciudad Colon, 800m [;] xii 1989–i.1990 [;] Luis Fournier (ESUW). 1 ♀, Costa Rica: San Jose [;] Cerro de la Muerte [;] 19km s 3 W Empalme [;] 2600m, November 1992 [;] P. Hanson, Malaise (ESUW). 2 ♀♀, Costa Rica: San Jose [;] 2km. W. Empalme [;] 2300m, July 1995 [;] P. Hanson, Malaise (ESUW). 2 ♀♀, COSTA RICA: [;] San Jose [;] Ciudad Colon [;] 800m, iii-iv 1990 [;] Col. Luis Fournier (ESUW). 1 ♀. Costa Rica: San Jose [;] San Antonio de Escazu [;] 1300m, xii.1998 [;] W. Eberhard (ESUW). 7 ♀♀, S.RosaPark, Guan. [;] C. Rica, various dates from May 1977 to May 1978 [;] D.H. Janzen [;] Dry Hill and Riparian (AEIC). 1 ♀, C. Rica: Escazú [;] May 21, 1987 [;] H. & M. Townes (AEIC).

#### Comments.

This species is distinguished by the long dense hair along the notauli, subalar area of the mesopleuron and laterally on the propodeum.

#### Etymology.

Named for my colleague and friend, Jim Whitfield, in recognition of his interest and help in this project.

**Figure 125.**
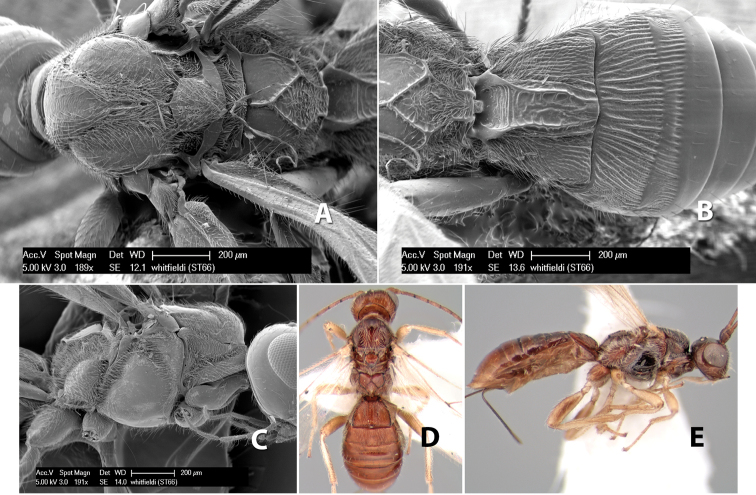
*Heterospilus whitfieldi* Marsh, sp. n.: **A–C, E** paratype **D** holotype.

### Heterospilus wilkinsoni

Marsh sp. n.

http://zoobank.org/654FF72B-BB4F-40F7-8D63-110B50102C96

http://species-id.net/wiki/Heterospilus_wilkinsoni

Figure 126

#### Female.

Body size: 4.0 mm. Color: head with vertex light brown, frons, face and eye orbits yellow; scape yellow, flagellum yellow at base to brown apically; mesosoma brown, yellow along notauli; metasoma honey yellow, tergum 1 and apex of tergum 3 light brown; legs yellow; wing veins brown, stigma bicolored brown with yellow apex. Head: vertex transversely costate; frons transversely costate; face striate; temple in dorsal view slightly bulging, width equal to 1/2 eye width; malar space equal to 1/4 eye height; ocell-ocular distance about twice diameter of lateral ocellus; antennae broken. Mesosoma: mesoscutal lobes granulate; notauli scrobiculate, meeting at scutellum in triangular rugose area; scutellum smooth; prescutellar furrow with 3 cross carina; mesopleuron smooth above precoxal sulcus, granulate and costate dorsally; precoxal sulcus scrobiculate, shorter than mesopleuron; venter smooth; propodeum with basal median areas rugose, distinctly margined, basal median carina present, areola not distinctly margined, areolar area rugose, lateral areas entirely rugose. Wings: fore wing vein r shorter than vein 3RSa, vein 1cu-a beyond vein 1M; hind wing vein SC+R present, vein M+CU shorter than vein 1M. Metasoma: first tergum costate-granulate, length greater than apical width; second tergum costate-granulate; anterior transverse groove present, straight; posterior transverse groove distinct; third tergum costate-granulate at base, smooth apically; terga 4-7 smooth; ovipositor longer than metasoma.

#### Holotype female.

Top label (white, partially printed and hand written) - Costa Rica: Guanacaste [;] Santa Rosa Natl. Park [;] 300m, ex. Malaise trap [;] Site #: BH.10.C [;] Dates: 8.ii–7.iii.1986 [;] I.D. Gauld & D. Janzen; second label (white, printed) - [BH] Bosque Humedo [;] mature evergreen dry forest [;] [C] more or less fully [;] shaded as possible; third label (red, partially printed and hand written) - HOLOTYPE [;] Heterospilus [;] wilkinsoni [;] P. Marsh. Deposited in ESUW.

#### Paratypes.

Known only from the holotype.

#### Comments.

This species is distinguished by the bicolored stigma, rugose basal median areas of the propodeum, and the honey yellow metasoma.

#### Etymology.

Named for the British entomologist, D. S. Wilkinson, who described many braconids in the early 1900s.

**Figure 126.**
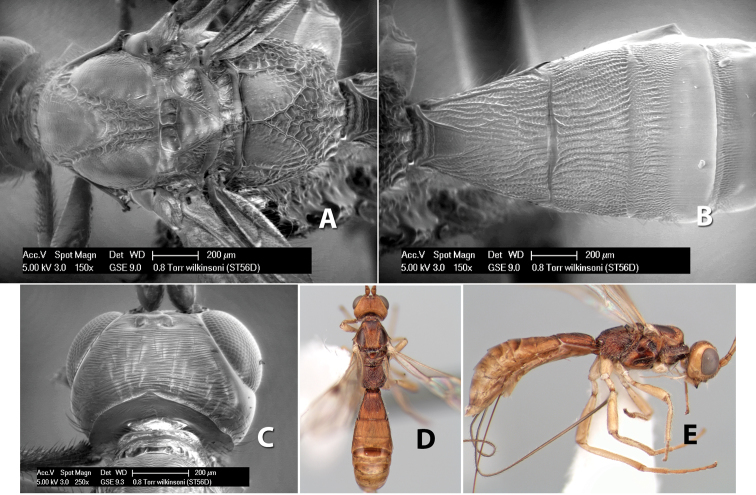
*Heterospilus wilkinsoni* Marsh, sp. n., holotype.

### Heterospilus xanthus

Marsh sp. n.

http://zoobank.org/6109E3E5-FD7D-4331-B7F5-12F700074579

http://species-id.net/wiki/Heterospilus_xanthus

Figures 127 , 128

#### Female.

Body size: 2.5–3.0 mm. Color: body entirely yellow or honey yellow; scape yellow without lateral brown stripe, flagellum yellow basally to brown apically; wing veins light brown to yellow, stigma yellow; legs yellow. Head: vertex transversely costate; frons transversely costate; face rugose; temple in dorsal view narrow, width less than 1/2 eye width; malar space less than 1/4 eye height; ocell-ocular distance equal to diameter of lateral ocellus; 17–22 flagellomeres. Mesosoma: mesoscutal lobes granulate; notauli scrobiculate, meeting at scutellum in triangular rugose area; scutellum granulate; prescutellar furrow with 3 cross carinae; mesopleuron granulate; precoxal sulcus scrobiculate, shorter than mesopleuron; venter granulate; propodeum with basal median areas margined, granulate, basal median carina absent, areola distinctly margined, areolar area rugose, lateral areas entirely rugose. Wings: fore wing vein r equal in length to vein 3RSa, vein 1cu-a beyond vein 1M; hind wing vein SC+R present, vein M+CU shorter than vein 1M. Metasoma: first tergum longitudinally costate, apical width equal to length, median raised area with distinct lateral carinae; second tergum longitudinally costate; anterior transverse groove present, straight; posterior transverse groove present; third tergum costate basally, smooth apically; terga 4–7 smooth; ovipositor equal to length of metasomal terga 1 and 2 combined.

#### Holotype female.

Top label (white, partially printed and hand written) - Costa Rica: Guanacaste [;] Santa Rosa Natl. Park [;] 300m, ex. Malaise trap [;] Site #: 8 [;] Dates: 23.iii–13.iv.1986 [;] I.D. Gauld & D. Janzen; second label (white, printed) - [SE] Bosque San Emilio [;] 50yr old deciduous forest [;] [C] more or less fully [;] shaded as possible; third label (red, partially printed and hand written) - HOLOTYPE [;] Heterospilus [;] xanthus [;] P. Marsh. Deposited in ESUW.

#### Paratypes.

4 ♀♀, same data as holotype except: dates of 13.iv–4.v.1986, 10-31.i.1987, 2–23.iii.1986; second label, [H] open regenerating [;] woodland <10 year old [;] [C] more or less fully [;] shaded as possible (ESUW). 1 ♀, Costa Rica, San Jose Pr. [;] San Pedro, university [;] sportfield, 1200m [;] lighttrap 17+20 1997 [;] L.J. van der Ent (ESUW). 1 ♀, Costa Rica, Guanacaste Pr. [;] Guan. Conservation Area [;] Santa Rosa hdq. 200m [;] lighttrap, 7-VII-1997 [;] L.J. van der Ent (ESUW). 3 ♀♀, Costa Rica: Guanacaste [;] PN Guanacaste, 7km E HQ [;] near “small house” [;] 9.iii.1990, J.S. Noyes (ESUW). 1 ♀, Costa Rica: Guanacaste Pr. [;] Guanacaste National Park [;] near Playa Naranja [;] 11 March 1990, J.S. Noyes (ESUW). 1 ♀, S.RosaPark, Guan. [;] C. Rica 27 Mar 78 [;] D. H. Janzen [;] Dry Hill (AEIC).

#### Comments.

The yellow body color and the large ocelli are distinctive for this species.

#### Etymology.

The specific name is from the Greek *xanthos*, meaning yellow, in reference to the entirely yellow body.

**Figure 127.**
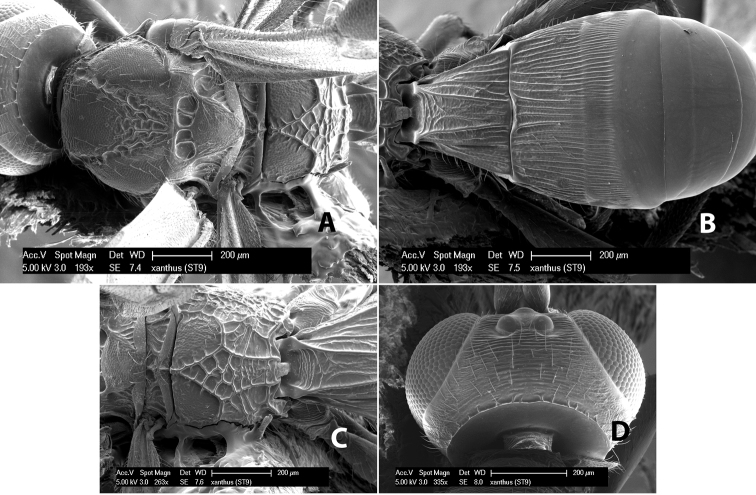
*Heterospilus xanthus* Marsh, sp. n., paratype.

**Figure 128.**
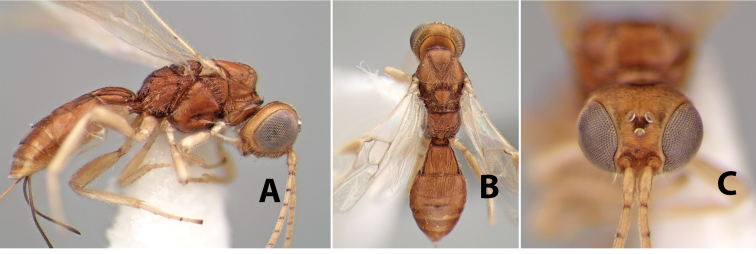
*Heterospilus xanthus* Marsh, sp. n., holotype.

### Heterospilus yaqui

Marsh sp. n.

http://zoobank.org/862EF038-AD29-40D1-9705-DE7A5A897EA4

http://species-id.net/wiki/Heterospilus_yaqui

Figure 129

#### Female.

Body size: 3.5 mm. Color: head with vertex and frons brown, face, temple and eye orbits yellow; scape yellow with lateral longitudinal brown stripe, flagellum brown; mesosoma dark brown; metasoma dark brown; wing veins light brown, stigma brown with yellow at base; legs yellow. Head: vertex weakly striate; frons weakly striate; face rugose-striate medially; temple in dorsal view broad but sloping behind eye, width equal to 1/2 eye width; malar space greater than 1/4 eye height; ocell-ocular distance about 2.5 times diameter of lateral ocellus; 23 flagellomeres. Mesosoma: mesoscutal lobes granulate; notauli scrobiculate, meeting at scutellum in triangular costate area; scutellum smooth; prescutellar furrow with 3 cross carinae; mesopleuron granulate; precoxal sulcus smooth, shorter than mesopleuron; venter granulate; propodeum with basal median areas margined, basal median carina absent, areola, not distinctly margined areolar area rugose, lateral areas rugose apically, granulate basally. Wings: fore wing vein r shorter than vein 3RSa, vein 1cu-a beyond vein 1M; hind wing vein SC+R present, vein M+CU shorter than vein 1M. Metasoma: first tergum longitudinally costate, length greater than apical width; second tergum longitudinally costate; anterior transverse groove weakly present, straight; posterior transverse groove weakly present; third tergum costate basally, smooth apically; terga 4–7 smooth; ovipositor as long as metasomal terga 1 and 2 combined.

#### Holotype female.

Top label (white, printed) - COSTA RICA: Prov. [;] Heredia, F. La Selva [;] 3km S Pto. Viejo [;] 10°26'N, 84°01W; second label (white, printed) - at fallen branch of [;] Pentaclethra macroloba; third label (white, partially printed and hand written) - 9.iv.1984 [;] H.A. Hespenheide; fourth label (red, partially printed and hand written) - HOLOTYPE [;] Heterospilus [;] yaqui [;] P. Marsh. Deposited in ESUW.

#### Paratypes.

Known only from the holotype.

#### Comments.

The partially yellow head and the brown stripe laterally on the scape are distinctive for this species.

#### Etymology.

Named for the Yaqui, an indigenous people from Sonora, Mexico.

**Figure 129.**
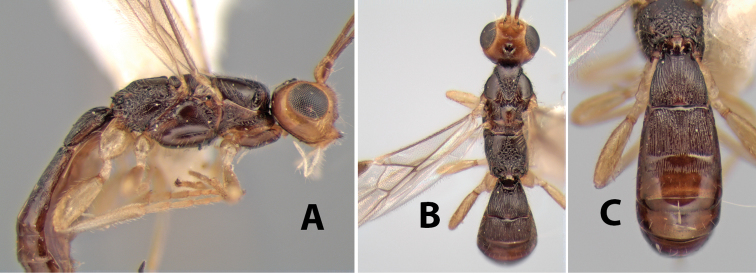
*Heterospilus yaqui* Marsh, sp. n., holotype.

### Heterospilus zunigai

Marsh sp. n.

http://zoobank.org/434482CB-CBB6-427F-BC32-C1977247FE36

http://species-id.net/wiki/Heterospilus_zunigai

Figure 130

#### Female.

Body size: 2.5 mm. Color: head with vertex and frons brown, face and eye orbits yellow or honey yellow; scape yellow without lateral longitudinal brown stripe, flagellum entirely brown; mesosoma brown, light brown along notauli, scutellum and metanotum; metasoma dark brown, terga 2 light brown, terga 5-7 yellow; wing veins brown, stigma brown medially, yellow at base, apex and along anterior margin; legs yellow. Head: vertex transversely striate; frons transversely striate; face smooth; temple in dorsal view narrow, sloping behind eye, width less than 1/2 eye width; malar space greater than 1/4 eye height; ocell-ocular distance about twice diameter of lateral ocellus; 22–27 flagellomeres. Mesosoma: mesoscutal lobes granulate; notauli scrobiculate, meeting at scutellum in triangular costate-rugose area; scutellum granulate; prescutellar furrow with 3 cross carinae; mesopleuron smooth; precoxal sulcus smooth, shorter than mesopleuron; venter smooth; propodeum with basal median areas distinctly margined, smooth, basal median carina present but short, areola not distinctly margined, areolar area rugose, lateral areas entirely rugose. Wings: fore wing vein r shorter than vein 3RSa, vein 1cu-a beyond vein 1M; hind wing vein SC+R present, vein M+CU shorter than vein 1M. Metasoma: first tergum longitudinal costate, length longer than apical width; second tergum longitudinally costate, width about 4 times length; anterior transverse groove present, straight; posterior transverse groove present; third tergum costate basally, smooth apically; terga 4–7 smooth; ovipositor about as long as metasoma.

#### Holotype female.

Top label (white, partially printed and hand written) - Costa Rica: Guanacaste [;] Santa Rosa Natl. Park [;] 300m, ex. Malaise trap [;] Site #: SE-5-O [;] Dates: 24.v–14.vi.1986 [;] I.D. Gauld & D. Janzen; second label (white, printed) - [SE] Bosque San Emilio [;] 50yr old deciduous forest [;] [O] in clearing, fully [;] isolated part of day; third label (red, partially printed and hand written )- HOLOTYPE [;] Heterospilus [;] zunigai [;] P. Marsh. Deposited in ESUW.

#### Paratypes.

1 ♀, Costa Rica: Guanacaste [;] Est. Biol. Maritza, 600m [;] i.1997. C. Zuniga. Malaise [;] L.N. 326900-373000 #47557 (ESUW).

#### Comments.

The smooth mesopleuron just above the precoxal sulcus, the granulate mesoscutum and the smooth face are distinctive for this species.

#### Etymology.

Named for C. Zuniga, one of the INBio parataxonomists.

**Figure 130.**
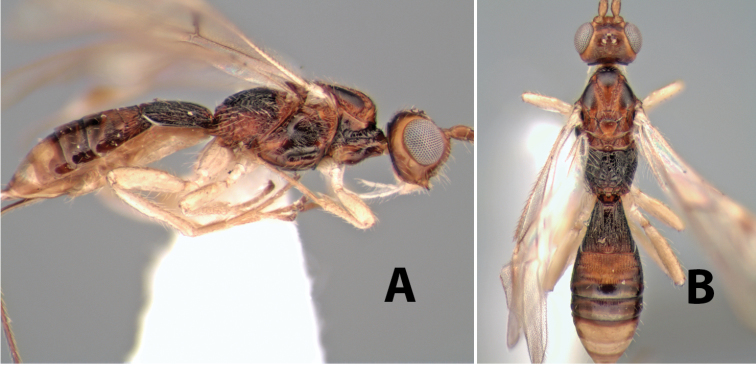
*Heterospilus zunigai* Marsh, sp. n., holotype.

### Key to species of Costa Rican *Heterospilus* with granulate vertex

**Table:** 

1	Hind wing vein SC+R present	2
–	Hind wing vein SC+R absent	42
2(1)	Metasomal terga 3-6 granulate apically, rarely smooth medially but at least granulate laterally	3
–	Metasomal terga 3-6 smooth apically	15
3(2)	Vertex granulate and usually with distinct transverse rugae behind ocelli, occasionally finely striate behind ocelli	4
–	Vertex evenly granulate behind ocelli	6
4(3)	Face granulate; temple in dorsal view narrow, sloping behind, width less than 1/2 eye width	*Heterospilus ektorincon* Marsh, sp. n.
–	Face rugose; temple in dorsal view broader, not distinctly sloping behind eye, width about 1/2 eye width	5
5(4)	Head dark brown, usually with yellow eye orbits; flagellum brown with white annulus near apex	*Heterospilus rojasi* Marsh, sp. n.
–	Head entirely brown or dark brown; flagellum entirely brown, without white annulus	*Heterospilus austini* Marsh, sp. n.
6(4)	Stigma of fore wing yellow or honey yellow	7
–	Stigma of fore wing brown or black, occasionally hyaline brown	9
7(6)	Metasoma mostly yellow or honey yellow, head and mesosoma dark brown or black	*Heterospilus flavisoma* Marsh, sp. n.
–	Metasoma with at most apical terga 6-7 lighter, remainder of terga dark brown, concolorous with mesosoma	8
8(7)	Scape yellow with lateral longitudinal brown stripe	*Heterospilus achi* Marsh, sp. n.
–	Scape entirely dark brown or black	*Heterospilus dianae* Marsh, sp. n.
9(6)	Ovipositor at most as long as metasomal tergum 1, usually shorter	10
–	Ovipositor longer than metasomal tergum 1	12
10(9)	Metasomal tergum 1 distinctly wider at apex than length; flagellum entirely brown with white annulus near tip	*Heterospilus cora* Marsh, sp. n.
–	Metasomal tergum 1 width equal to or less than length; flagellum with white annulus near tip	11
11(10)	Body entirely black or dark brown	*Heterospilus barbalhoae* Marsh, sp. n.
–	Head, mesoscutum, metasomal tergum 2 dorsally and apical metasomal terga honey yellow, remainder of body brown	*Heterospilus quitirrisi* Marsh, sp. n.
12(9)	Metasomal tergum 1 length equal to apical width	13
–	Metasomal tergum 1 length greater than width	14
13(12).	Propodeum in dorsal view with small but distinct tubercles just above hind coxae; flagellum with white annulus near tip	*Heterospilus chorotegus* Marsh, sp. n.
–	Propodeum without tubercles above hind coxae; flagellum entirely brown, without white annulus	*Heterospilus wrightae* Marsh, sp. n.
14(12)	Metasomal terga entirely brown	*Heterospilus carolinae* Marsh, sp. n. (in part)
–	Metasomal terga 5-7 yellow, terga 1-4 brown	*Heterospilus zapotec* Marsh, sp. n. (in part)
15(2)	Body, including head, flattened dorso-ventrally	*Heterospilus complanatus* Marsh, sp. n.
–	Body not flattened dorso-ventrally	16
16(15)	Prescutellar furrow with one cross carina	17
–	Prescutellar furrow with 3-5 cross carinae	18
17(16)	Hind coxa brown, fore and mid coxae yellow	*Heterospilus phaeocoxus* Marsh, sp. n.
–	All coxae unicolorous yellow or light brown	*Heterospilus kikapu* Marsh, sp. n.
18(16)	Propodeum with median basal carina distinct and long, at least as long as median cross carina of prescutellar furrow, usually longer	19
–	Propodeum with median basal carina absent, if rarely a short carina apparently present, it is shorter than median cross carinae of prescutellar furrow, costulae of areola usually meeting at base of propodeum	24
19(18)	Scape yellow or brown, without lateral longitudinal brown stripe	20
–	Scape yellow, with lateral longitudinal brown stripe	21
20(19)	Fore wing vein r slightly less than 1/2 length of vein 3RSa; flagellum entirely brown; ocell-ocular distance 1.5 times diameter of lateral ocellus	*Heterospilus amuzgo* Marsh, sp. n.
–	Fore wing vein r nearly as long as vein 3RSa; flagellum brown with apical flagellomeres white; ocell-ocular distance 2.5 times diameter of lateral ocellus	*Heterospilus zitaniae* Marsh, sp. n.
21(19)	Metasomal tergum 2 yellow or lighter than remainder of terga	*Heterospilus bicolor* Marsh, sp. n.
–	Metasomal tergum 2 brown, concolorous with remainder of terga	22
22(21)	Mesoscutum light brown or honey yellow	*Heterospilus cangrejaensis* Marsh, sp. n.
–	Mesoscutum dark brown	23
23(22)	Ovipositor as long as metasomal tergum 1	*Heterospilus guapilensis* Marsh, sp. n.
–	Ovipositor 3/4 length of metasoma	*Heterospilus chocho* Marsh, sp. n.
24(18)	Face smooth	25
–	Face sculptured, striate, granulate or areolate	26
25(24)	Scutellum smooth	*Heterospilus pitillaensis* Marsh, sp. n.
–	Scutellum granulate	*Heterospilus orosi* Marsh, sp. n.
26(24)	Notauli meeting posteriorly in triangular rugose area	27
–	Notauli meeting posteriorly in triangular costate or costate rugose area	32
27(26)	Basal median areas of propodeum distinctly margined	*Heterospilus cocopa* Marsh, sp. n.
–	Basal median areas of propodeum not margined, often small or absent	28
28(27)	Ocell-ocular distance 2.5-3 times diameter of lateral ocellus	29
–	Ocell-ocular distance 2 times or less diameter of lateral ocellus	30
29(28)	Fore wing vein r usually as long as, or rarely very slightly shorter than, vein 3RSa	*Heterospilus jonmarshi* Marsh, sp. n.
–	Fore wing vein r distinctly shorter than vein 3RSa	*Heterospilus ixcatec* Marsh, sp. n.
30(28)	Propodeum with distinct tubercle or projection apico-laterally just above hind coxae	*Heterospilus sumo* Marsh, sp. n.
–	Propodeum without tubercle or projection above hind coxae	31
31(30)	Legs bicolored, hind femur yellow on basal half, brown on apical half; flagellum brown with white annulus near apex	*Heterospilus villegasi* Marsh, sp. n.
–	Legs entirely yellow; flagellum entirely brown	*Heterospilus nemestrinus* Marsh, sp. n.
32(26)	Basal median areas of propodeum not distinctly margined	33
–	Basal median areas of propodeum distinctly margined	34
33(32)	Body light brown to honey yellow; flagellum entirely brown	*Heterospilus achterbergi* Marsh, sp. n.
–	Head and mesosoma dark brown, metasoma bicolored dark brown and yellow; flagellum brown with weak apical white annulus	*Heterospilus pech* Marsh, sp. n.
34(32)	Vertex with more or less distinct transverse rugae or costae behind ocelli	35
–	Vertex entirely granulate	38
35(34)	Scape entirely brown or dark brown	*Heterospilus sharkeyi* Marsh, sp. n.
–	Scape yellow with lateral longitudinal brown stripe	36
36(35)	Head yellow	*Heterospilus ugaldei* Marsh, sp. n.
–	Head dark brown	37
37(36)	Mesoscutal lobes brown to light brown, lighter than remainder of mesosoma	*Heterospilus jennieae* Marsh, sp. n. (in part)
–	Mesosoma entirely dark brown	*Heterospilus arawak* Marsh, sp. n.
38(34)	Scape brown or dark brown	39
–	Scape yellow with lateral longitudinal brown stripe	40
39(38)	Stigma yellow	*Heterospilus puertoviejoensis* Marsh, sp. n.
–	Stigma brown	*Heterospilus chilamatensis* Marsh, sp. n.
40(38)	Apical metasomal terga dark brown, concolorous with anterior terga	*Heterospilus carolinae* Marsh, sp. n. (in part)
–	Apical metasomal terga yellow, remainder of terga dark brown, tergum 2 often with yellow spots laterally	41
41(40)	Metasomal tergum 2 entirely dark brown	*Heterospilus zapotec* Marsh, sp. n. (in part)
–	Metasomal tergum 2 brown with yellow spots laterally	*Heterospilus zoque* Marsh, sp. n.
42(1)	Metasomal terga 3-6 smooth apically	43
–	Metasomal terga 3-6 granulate apically	60
43(42)	Ovipositor as long as or shorter than metasomal tergum 1	44
–	Ovipositor longer than metasomal tergum 1	51
44(43)	Scape yellow or brown without lateral brown stripe	45
–	Scape yellow with more or less distinct lateral longitudinal brown stripe	46
45(44)	Length of metasomal tergum 1 nearly twice apical width; mesoscutal lobes weakly granulate and shining	*Heterospilus nahua* Marsh, sp. n.
–	Length of metasomal tergum 1 equal to apical width; mesoscutal lobes distinctly granulate and dull	*Heterospilus poqomam* Marsh, sp. n.
46(44)	Basal median areas of propodeum distinctly margined	47
–	Basal median area of propodeum not distinctly margined	49
47(46)	Mesoscutal lobes weakly granulate and shining	*Heterospilus mam* Marsh, sp. n.
–	Mesoscutal lobes distinctly granulate and dull	48
48(47)	Length of metasomal tergum 1 at least 1.5 times apical width, often nearly twice as long	*Heterospilus strazanaci* Marsh, sp. n.
–	Length of metasomal tergum 1 about equal to apical width	*Heterospilus aubreyae* Marsh, sp. n.
49(46)	Propodeum with distinct tubercle (raised carina) laterally just above hind coxa	*Heterospilus vulcanus* Marsh, sp. n.
–	Propodeum without distinct tubercle laterally above hind coxa	50
50(49)	Prescutellar furrow with 3 cross carinae; mesoscutal lobes weakly granulate and shining	*Heterospilus jabillosensis* Marsh, sp. n.
–	Prescutellar furrow with 1 cross carina; mesoscutal lobes distinctly granulate and dull	*Heterospilus parkeri* Marsh, sp. n.
51(43)	Scape yellow without lateral longitudinal brown stripe; flagellum entirely brown	52
–	Scape yellow with lateral longitudinal brown stripe; flagellum with apical white annulus	54
52(51)	Fore wing vein r at most half as long as vein 3RSa	*Heterospilus ixil* Marsh, sp. n.
–	Fore wing vein r nearly as long as or equal to vein 3RSa	53
53(52)	Body entirely yellow or honey yellow	*Heterospilus warreni* Marsh, sp. n.
–	Body entirely dark brown	*Heterospilus longisulcus* Marsh, sp. n.
54(51)	Areola on propodeum with numerous distinct cross carinae or costae	*Heterospilus tzutujil* Marsh, sp. n.
–	Areola on propodeum rugose or areolate-rugose	55
55(54)	Mesosoma entirely dark brown or black	56
–	Mesosoma light brown or bicolored, at least mesoscutum partially light brown or yellow	58
56(55)	Vertex granulate with weak but distinctive transverse rugae behind ocelli	*Heterospilus sergeyi* Marsh, sp. n.
–	Vertex entirely granulate	57
57(56)	Metasomal tergum 2 dark brown, light brown or honey yellow medially; ocell-ocular distance usually more than twice diameter of lateral ocellus	*Heterospilus bennetti* Marsh, sp. n.
–	Metasomal tergum 2 entirely dark brown; ocell-ocular distance usually twice or less diameter of lateral ocellus	*Heterospilus spiloheterus* Marsh, sp. n.
58(55)	Notauli meeting at prescutellar furrow in unsculptured area	*Heterospilus lasalturus* Marsh, sp. n.
–	Notauli meeting at prescutellar furrow in small triangular costate area	59
59(58)	Temple in dorsal view strongly sloping behind eye	*Heterospilus robbieae* Marsh, sp. n.
–	Temple somewhat bulging, not strongly sloping behind eye	*Heterospilus mopanmaya* Marsh, sp. n.
60(42)	Ovipositor as long as or shorter than metasomal tergum 1	61
–	Ovipositor longer than metasomal tergum 1	72
61(60)	Metasomal tergum 2 bicolored brown or dark brown with median yellow or honey yellow spot	62
–	Metasomal tergum brown or yellow, concolorous with anterior and/or posterior terga	67
62(61)	Mesoscutal lobes lighter in color than remainder of mesosoma	*Heterospilus tricolor* Marsh, sp. n.
–	Mesosoma entirely brown, dark brown or black	63
63(62)	Face smooth, rarely partially very weakly granulate	64
–	Face granulate or striate	65
64(63)	Length of metasomal tergum 1 greater than apical width	*Heterospilus saminae* Marsh, sp. n. (in part)
–	Length of metasomal tergum 1 equal to apical width	*Heterospilus dani* Marsh, sp. n.
65(63)	Mesoscutal lobes weakly granulate, shining	*Heterospilus aesculapius* Marsh, sp. n.
–	Mesoscutal lobes distinctly granulate, dull	66
66(65)	Propodeum with distinct tubercle laterally just above hind coxa	*Heterospilus sabrinae* Marsh, sp. n. (in part)
–	Propodeum without lateral tubercle above hind coxa	*Heterospilus bacchus* Marsh, sp. n.
67(61)	Notauli weak or absent posteriorly	*Heterospilus caritus* Marsh, sp. n.
–	Notauli complete and distinct posteriorly	68
68(67)	Notauli meeting at prescutellar furrow in broadly rugose area	*Heterospilus longinoi* Marsh, sp. n.
–	Notauli meeting at prescutellar in costate or unsculptured area	69
69(68)	Face more or less smooth and shining	*Heterospilus saminae* Marsh, sp. n. (in part)
–	Face granulate and dull	70
70(69)	Apical metasomal terga bright yellow, distinctly contrasting with the dark brown anterior terga	*Heterospilus kellieae* Marsh, sp. n.
–	Apical metasomal terga usually concolorous with anterior terga, rarely slightly lighter brown	71
71(70)	Notauli deeply scrobiculate anteriorly, meeting at prescutellar furrow in deeply costate-rugose area	*Heterospilus agujasensis* Marsh, sp. n.
–	Notauli shallow and weakly scrobiculate anteriorly, meeting at prescutellar furrow in weakly costate area	*Heterospilus sabrinae* Marsh, sp. n. (in part)
72(60)	Body entirely yellow, apical metasomal terga rarely darker	*Heterospilus microstigmi* Richards
–	Body entirely brown or dark brown	73
73(72)	Ovipositor longer than metasoma; flagellum entirely brown	*Heterospilus richardsi* Marsh & Mello
–	Ovipositor shorter than metasoma; flagellum brown with apical white annulus	74
74(73)	Metasomal tergum 2 entirely brown or dark brown	75
–	Metasomal tergum 2 bicolored brown with yellow area medially	76
75(74)	Mesoscutal lobes weakly granulate, shining	*Heterospilus lenca* Marsh, sp. n.
–	Mesoscutal lobes granulate and dull	*Heterospilus rama* Marsh, sp. n.
76(74)	Scape yellow without lateral longitudinal brown stripe	*Heterospilus braeti* Marsh, sp. n.
–	Scape yellow with distinct lateral longitudinal brown stripe	77
77(76)	Vertex granulate with weak transverse striae or rugae behind ocelli	*Heterospilus leenderti* Marsh, sp. n.
–	Vertex entirely granulate	78
78(77)	Mesosoma entirely dark brown, concolorous with head	*Heterospilus phytorius* Marsh, sp. n.
–	Mesosoma partially or entirely brown or light brown or bicolored, usually lighter in color than head	*Heterospilus xinca* Marsh, sp. n.

### Heterospilus achi

Marsh sp. n.

http://zoobank.org/13CB5BF9-0DEC-4640-8A5E-6D77B60D317B

http://species-id.net/wiki/Heterospilus_achi

Figure 131

#### Female.

Body size: 3.0 mm. Color: head dark brown; scape yellow with lateral longitudinal brown stripe, flagellum brown with apical white band, apical most 3–5 flagellomeres brown; mesosoma dark brown; metasoma dark brown, metasomal terga 5–7 yellow or light brown; wing veins brown, stigma yellow; legs yellow. Head: vertex granulate; frons granulate; face granulate; temple in dorsal view narrow, width slightly less than 1/2 eye width; malar space about 1/4 eye height; ocell-ocular distance slightly greater than 2.5 times diameter of lateral ocellus; 24–25 flagellomeres. Mesosoma: mesoscutal lobes granulate, median lobe with median longitudinal groove posteriorly; notauli scrobiculate, meeting at scutellum in triangular costate area; scutellum granulate; prescutellar furrow with 3–5 cross carinae; mesopleuron granulate; precoxal sulcus weakly scrobiculate or smooth, shorter than mesopleuron; venter granulate; propodeum with basal median areas margined, granulate, basal median carina absent, areola not distinctly margined, areolar area areolate-rugose, lateral areas entirely rugose. Wings: fore wing vein r shorter than vein 3RSa, vein 1cu-a beyond vein 1M; hind wing vein SC+R present, vein M+CU shorter than vein 1M. Metasoma: first tergum longitudinally costate, apical width equal to length; second tergum longitudinally costate; anterior transverse groove present, straight; posterior transverse groove present; third tergum costate basally, granulate apically; terga 4–7 granulate; ovipositor equal in length to metasomal terga 1 and 2 combined.

#### Holotype female.

Top label (white, partially printed and hand written) - Costa Rica: Guanacaste [;] Santa Rosa National Pk. [;] 300m, Malaise, Ian Gauld [;] 10-31.i.1987; second label (white, partially printed and hand written) - Bosque San Emilio [;] 59 yr old deciduous [;] forest [;] Full Shade; third label (white, printed) - SE-6-C [;] 10-31.i.87; fourth label (red, partially printed and hand written) - HOLOTYPE [;] Heterospilus [;] achi [;] P. Marsh. Deposited in ESUW.

#### Paratypes.

1 ♀, top label - Costa Rica: Guanacaste [;] Santa Rosa National Pk. [;] 300m, Malaise, Ian Gauld [;] 31.i–21.ii.1987; second label - Bosque Humedo [;] mature dry forest [;] high proportion [;] evergreen species [;] Sun; third label - BH-9-O [;] 31.i–21.ii.87 (ESUW). 3 ♀♀, top label - Costa Rica: Guanacaste [;] Santa Rosa National Pk. [;] 300m, ex. Malaise trap [;] Site #: BH-12-C and blank [;] Dates: 8.ii–2.iii.1986, 8–29.xi.1986 and 16.xi–2.xii.1985 [;] I.D. Gauld & D. Janzen; second label - [BH] Bosque Humedo [;] mature evergreen dry forest [;] [C] more or less fully [;] shaded as possible and [SE] Bosque San Emilio [;] 50yr old deciduous forest [;] [C] more or less fully [;] shaded as possible (ESUW). 15 ♀♀, S.RosaPark, Guan. [;] C. Rica, dates 5 Dec 76 to 4 Nov 77 [;] D.H. Janzen [;] Riparian (AEIC).

#### Comments.

The yellow stigma and the brown lateral stripe on the scape are distinctive for this species.

#### Etymology.

Named for the Achi, a Mayan people of Guatemala.

**Figure 131.**
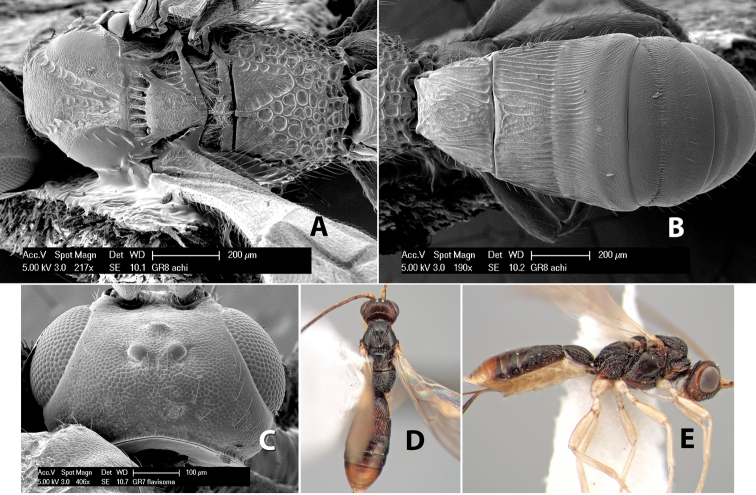
*Heterospilus achi* Marsh, sp. n.: **A–C** paratype **D–E** holotype.

### Heterospilus achterbergi

Marsh sp. n.

http://zoobank.org/72ED5CB1-1658-4303-AAAC-888CF2C3B7F4

http://species-id.net/wiki/Heterospilus_achterbergi

Figure 132

#### Female.

Body size: 2.5–3.0 mm. Color: body entirely light brown to brown; scape yellow without lateral brown stripe, flagellum yellow at base to brown at apex; wing veins including stigma brown; legs yellow. Head: vertex granulate, often with weak transverse rugae behind ocelli; frons granulate; face granulate-rugose; temple in dorsal view broad, slightly bulging behind eye, width equal to 1/2 eye width; malar space greater than 1/4 eye height; ocell-ocular distance 2.5 times diameter of lateral ocellus; 19–22 flagellomeres. Mesosoma: mesoscutal lobes granulate; notauli scrobiculate, meeting at scutellum in triangular costate area; scutellum granulate; prescutellar furrow with 3–5 cross carinae; mesopleuron granulate; precoxal sulcus smooth, shorter than mesopleuron; venter smooth or weakly granulate; propodeum with basal median areas distinct but not distinctly margined, granulate, basal median carina absent, areola not margined, areolar area areolate-rugose, lateral areas entirely rugose. Wings: fore wing vein r slightly shorter or nearly equal to vein 3RSa, vein 1cu-a beyond vein 1M; hind wing vein SC+R present, vein M+CU shorter than vein 1M. Metasoma: first tergum longitudinally costate-rugose, length equal to apical width; second tergum longitudinally costate; anterior transverse groove present, straight; posterior transverse groove present; third tergum costate anteriorly, smooth posteriorly; terga 4–7 smooth; ovipositor as long as 1/2 metasoma.

#### Holotype female.

Top label (white, printed) - Costa Rica: San Jose [;] 2km. W. Empalme [;] 2300m, July 1995 [;] P. Hanson, Malaise; second label (red, partially printed and hand written) - HOLOTYPE [;] Heterospilus [;] achterbergi [;] P. Marsh. Deposited in ESUW.

#### Paratypes.

27 ♀♀, same data as holotype (ESUW). 39 ♀♀, top label - Costa Rica: Guanacaste [;] Santa Rosa Natl. Park [;] 300m, ex. Malaise trap [;] Site #: BH-9-O, BH-11-O and blank [;] Dates: 2–23.iii.1986, 13.iv–4.v.1986, 23.iii–13.iv.1986, 8.ii–2.iii.1986 and 18.i–8.ii.1986 [;] I.D. Gauld & D. Janzen; second label - [BH] Bosque Humedo [;] mature evergreen dry forest [;] [O] in clearing, fully [;] isolated part of day (ESUW). 66 ♀♀, top label - Costa Rica: Guanacaste [;] Santa Rosa Natl. Park [;] 300m, ex. Malaise trap [;] Site #: BH-10-C, BH-12-C and blank [;] Dates: 4–24.v.1986, 13.iv–4.v.1986, 8.ii–2.iii.1986, 26.vii–14.viii.1986, 23.iii–13.iv.1986, 8.i–2.ii.1986, 6–27.ix.1986 and 2–23.iii.1986 [;] I.D. Gauld & D. Janzen; second label - [BH] Bosque Humedo [;] mature evergreen dry forest [;] [C] more or less fully [;] shaded as possible (ESUW). 15 ♀♀, top label - Costa Rica: Guanacaste [;] Santa Rosa Natl. Park [;] 300m, ex. Malaise trap [;] Site #: H-4-C [;] Dates: 4–24.v.1986 and 2–23.iii.1986 [;] I.D. Gauld & D. Janzen; second label - [H] open regenerating [;] woodland <10 years old [;] [C] more or less fully [;] shaded as possible (ESUW). 1 ♀, top label - Costa Rica: Guanacaste [;] Santa Rosa Natl. Park [;] 300m, ex. Malaise trap [;] Site #: H-1-O [;] Dates: 13.iv–4.v.1986 [;] I.D. Gauld & D. Janzen; second label - [H] open regenerating [;] woodland <10 years old [;] [O] in clearing, fully [;] isolated part of day (ESUW). 5 ♀♀, top label - Costa Rica: Guanacaste [;] Santa Rosa Natl. Park [;] 300m, ex. Malaise trap [;] Site #: SE-6-C [;] Dates: 2–23.iii.1986, 13.iv–4.v.1986 and 23.iii–13.iv.1986 [;] I.D. Gauld & D. Janzen; second label - [SE] Bosque San Emelio [;] 50yr old deciduous forest [;] [C] more or less fully [;] shaded as possible (ESUW). 4 ♀♀, Costa Rica: San Jose [;] Cerro de la Muerte [;] 19km S 3 W Empalme [;] 2600m, November 1992 [;] P. Hanson, Malaise (ESUW). 1 ♀, S.RosaPark, Guan. [;] C. Rica 26 Feb. 77 [;] D. H. Janzen [;] Riparian (AEIC).

#### Comments.

The light brown body color and the unmargined basal median areas of the propodeum are distinctive for this species.

#### Etymology.

Named for my long time friend and colleague Kees van Achterberg.

**Figure 132.**
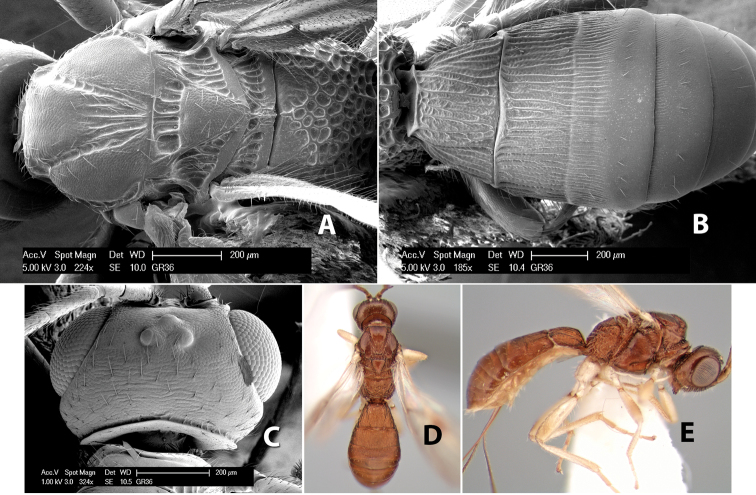
*Heterospilus achterbergi* Marsh, sp. n.: **A–C** paratype **D–E** holotype.

### Heterospilus aesculapius

Marsh sp. n.

http://zoobank.org/F746F293-5497-4057-827A-4C4A224E144A

http://species-id.net/wiki/Heterospilus_aesculapius

Figure 133

#### Female.

Body size: 2.5 mm. Color: body dark brown or black, metasomal tergum 1 entirely and tergum 2 medially at base yellow; scape yellow with lateral longitudinal brown stripe, flagellum brown with apical whit annulus, apical 3–5 flagellomeres brown; wing veins including stigma brown; legs yellow. Head: vertex weakly granulate and partially smooth; frons weakly granulate or smooth; face granulate; temple in dorsal view broad but sloping behind eye, width equal to 1/2 eye width; malar space greater than 1/4 eye height; ocell-ocular distance twice diameter of lateral ocellus; 26–27 flagellomeres. Mesosoma: mesoscutal lobes weakly granulate and partially smooth; notauli weakly scrobiculate or smooth, junction with prescutellar furrow unsculptured and represented by longitudinal dimple; scutellum smooth; prescutellar furrow with 3–5 cross carinae; mesopleuron granulate; precoxal sulcus scrobiculate, shorter than mesopleuron; venter granulate; propodeum with basal median areas margined, granulate, basal median carina absent, areola not margined, areolar area areolate-rugose, lateral areas entirely rugose. Wings: fore wing vein r shorter than vein 3RSa, vein 1cu-a interstitial with vein 1M; hind wing vein SC+R absent, vein M+CU shorter than vein 1M. Metasoma: first tergum longitudinally costate, length equal to apical width; second tergum longitudinally costate-granulate; anterior transverse groove present, straight; posterior transverse groove present; third tergum either entirely granulate except for costate transverse groove or costate-granulate at base and granulate at apex; terga 4–7 granulate; ovipositor slightly shorter than metasomal tergum 1.

#### Holotype female.

Top label (white, printed) - COSTA RICA: Puntarenas [;] Golfo Dulce, [;] 15km W. Piedras Blancas, [;] 100m [;] xi 1990, Paul Hanson; second label (red, partially printed and hand written) - HOLOTYPE [;] Heterospilus [;] aesculapius [;] P. Marsh. Deposited in ESUW.

#### Paratypes.

1 ♀, Costa Rica, Puntarenas [;] R.F. Golfo Dulce, 5km W. [;] Piedras Blancas, 100m [;] VI-VII-1993, P. Hanson (ESUW).

#### Comments.

The yellow markings on metasomal terga 1 and 2, the nearly smooth mesoscutum and granulate metasomal terga 3-7 are distinctive for this species.

#### Etymology.

Named for Aesculapius, the Roman god of health and medicine.

**Figure 133.**
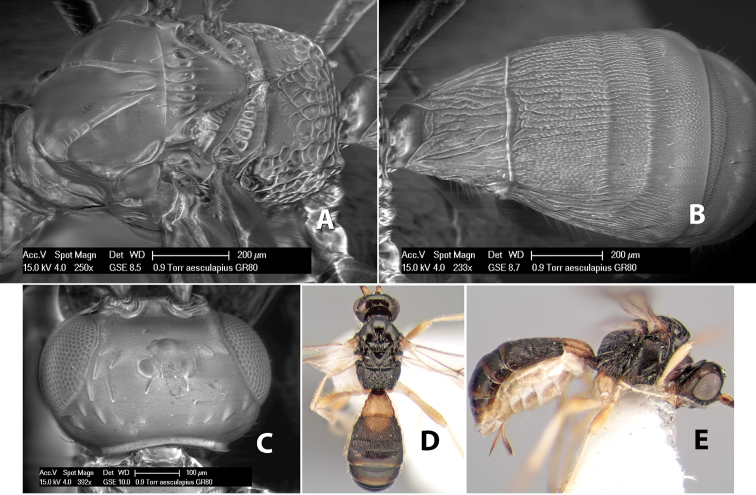
*Heterospilus aesculapius* Marsh, sp. n., holotype.

### Heterospilus agujasensis

Marsh sp. n.

http://zoobank.org/2CE4B724-3BDB-42B8-B250-FD7272B5762A

http://species-id.net/wiki/Heterospilus_agujasensis

Figure 134

#### Female.

Body size: 2.5 mm. Color: body dark brown, apical metasomal terga yellow; scape yellow without lateral brown stripe, flagellum brown with apical white annulus, apical 3–5 flagellomeres brown; wing veins including stigma brown; legs yellow. Head: vertex granulate; frons granulate; face granulate; temple in dorsal view narrow, sloping behind eye, width less than 1/2 eye width; malar space greater than 1/4 eye height; ocell-ocular distance about twice diameter of lateral ocellus; 19 flagellomeres. Mesosoma: mesoscutal lobes granulate; notauli deeply scrobiculate, meeting at scutellum in triangular costate-rugose area, mesoscutum along notauli rugose; scutellum granulate; prescutellar furrow with 3 cross carinae; mesopleuron granulate; precoxal sulcus scrobiculate, more or less circular, shorter than mesopleuron; venter granulate; propodeum with basal median areas margined, granulate, basal median carina absent, areola not margined, areolar area rugose, lateral areas entirely rugose, propodeum with small but distinct tubercle above hind coxa. Wings: fore wing vein r shorter than vein 3RSa, vein 1cu-a beyond vein 1M; hind wing vein SC+R absent, vein M+CU shorter than vein 1M. Metasoma: first tergum longitudinally costate, length equal to apical width; second tergum longitudinally costate; anterior transverse groove present, straight; posterior transverse groove present; third tergum entirely granulate except for costate transverse groove; terga 4–7 granulate; ovipositor shorter than metasomal tergum 1.

#### Holotype female.

Top label (white, printed) - Costa Rica: Prov. Puntarenas [;] ACO, Golfito, PN Corcovado [;] Est. Agujas, Cerro Rincon, 745m [;] 17.iv–16.v.1999, J. Azofeifa [;] L.S. 276900-521500 #52781; second label (red, partially printed and hand written) - HOLOTYPE [;] Heterospilus [;] agujasensis [;] P. Marsh. Deposited in ESUW.

#### Paratypes.

Known only from the holotype.

#### Comments.

The mesoscutum being rugose along the notauli, the granulate metasomal terga 3–7 and the white annulus on the flagellum are distinctive for this species.

#### Etymology.

Named for the Agujas Biological Station where the holotype was collected.

**Figure 134.**
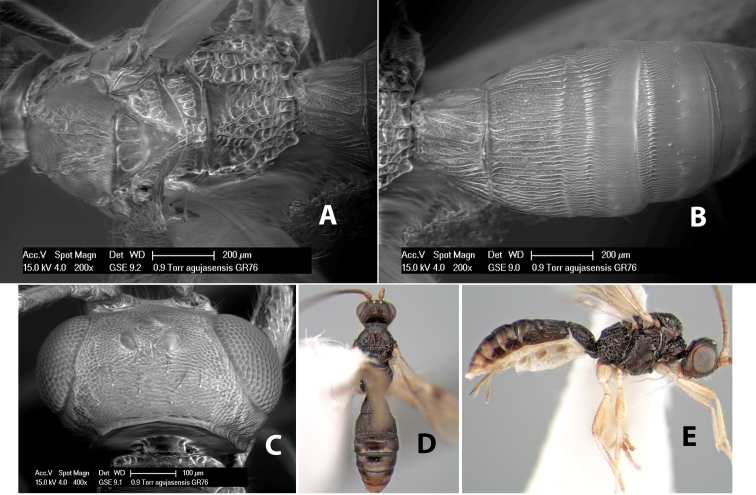
*Heterospilus agujasensis* Marsh, sp. n., holotype.

### Heterospilus amuzgo

Marsh sp. n.

http://zoobank.org/C649BE5B-1366-4D3A-83FD-18E86AE1E7F6

http://species-id.net/wiki/Heterospilus_amuzgo

Figure 135

#### Female.

Body size: 3.0 mm. Color: head honey yellow, vertex somewhat darker; scape yellow without lateral brown stripe, flagellum brown; mesosoma dark brown; metasomal terga 1 and 2 dark brown, tergum 3 yellow basally, brown apically, terga 4–7 honey yellow; wing veins including stigma brown; legs yellow. Head: vertex weakly granulate, smooth near eyes; frons weakly granulate; face weakly granulate, smooth near eyes; temple in dorsal view narrow, sloping behind eyes, width less than 1/2 eye width; malar space greater than 1/4 eye height; ocell-ocular distance about 1.5 times diameter of lateral ocellus; 22–24 flagellomeres. Mesosoma: mesoscutal lobes granulate; notauli scrobiculate, meeting at scutellum in triangular costate-rugose area; scutellum granulate; prescutellar furrow with 3 cross carinae; mesopleuron granulate; precoxal sulcus scrobiculate, shorter than mesopleuron; venter granulate; propodeum with basal median areas margined, granulate, basal median carina present, areola not distinctly margined, areolar area rugose, lateral areas entirely rugose. Wings: fore wing vein r shorter than vein 3RSa, vein 1cu-a beyond vein 1M; hind wing vein SC+R present, vein M+CU shorter than vein 1M. Metasoma: first tergum longitudinally costate, length about equal to apical width; second tergum longitudinally costate; anterior transverse groove present, straight; posterior transverse groove present; third tergum entirely smooth except for costate posterior transverse groove; terga 4-7 smooth; ovipositor as long as metasomal terga 1 and 2 combined.

#### Holotype female.

Top label (white, printed) - Costa Rica: San Jose [;] San Antonio de Escazu [;] 1300m, iii-iv.1998 [;] W. Eberhard & P. Hanson; second label (red, partially printed and hand written) - HOLOTYPE [;] Heterospilus [;] amuzgo [;] P. Marsh. Deposited in ESUW.

#### Paratypes.

Known only from the holotype.

#### Comments.

The brown flagellum and larger ocelli are distinctive for this species.

#### Etymology.

Named for the Amuzgo, an indigenous people of Mexico.

**Figure 135.**
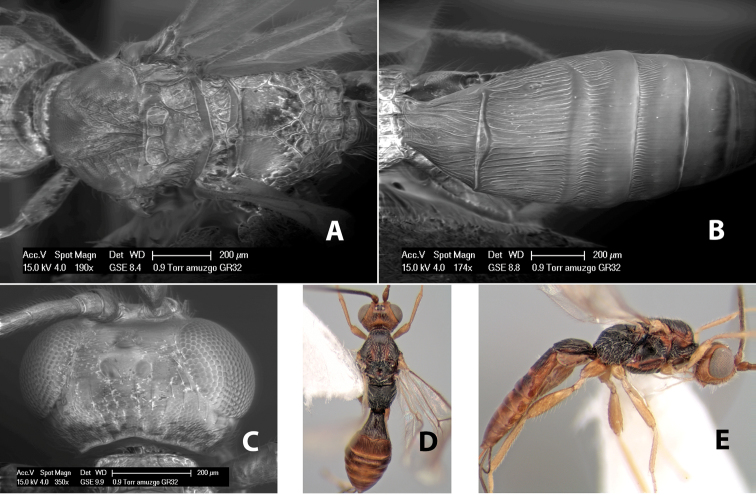
*Heterospilus amuzgo* Marsh, sp. n., holotype.

### Heterospilus arawak

Marsh sp. n.

http://zoobank.org/16B28CCC-D6BA-4587-AC30-8E1A02A3899E

http://species-id.net/wiki/Heterospilus_arawak

Figure 136

#### Female.

Body size: 3.5–4.0 mm. Color: body dark brown, head with eye orbits often lighter brown; scape yellow with lateral longitudinal brown stripe, flagellum brown with apical white annulus, apical 3–5 flagellomeres brown; wing veins brown, stigma bicolored brown with yellow at base; legs yellow. Head: vertex granulate; frons granulate-striate; face granulate-striate; temple in dorsal view broad but sloping behind eye, width equal to 1/2 eye width; malar space greater than 1/4 eye height; ocell-ocular distance 2.0–2.5 times diameter of lateral ocellus; 25–28 flagellomeres. Mesosoma: mesoscutal lobes granulate, shining; notauli scrobiculate, meeting at scutellum in triangular costate area; scutellum granulate, shining; prescutellar furrow with 3 cross carinae; mesopleuron granulate; precoxal sulcus weakly scrobiculate or smooth; venter granulate; propodeum with basal median areas distinctly margined, granulate, basal median carina absent, areola not distinctly margined, areolar area rugose, lateral areas rugose posteriorly, granulate anteriorly, propodeum with small but distinct tubercle above hind coxa. Wings: fore wing vein r shorter than vein 3RSa, vein 1cu-a beyond vein 1M; hind wing vein SC+R present, vein M+CU shorter than vein 1M. Metasoma: first tergum longitudinally costate, length equal to apical width; second tergum longitudinally costate; anterior transverse groove present, straight; posterior transverse groove present; third tergum costate basally, smooth apically; terga 4–7 smooth; ovipositor 1/2–3/4 length of metasoma.

#### Holotype female.

Top label (white, printed) - Costa Rica: Heredia [;] 3km. S. Puerto Viejo, [;] OTS, La Selva, 100m [;] xii.1992, P. Hanson; second label (red, partially printed and hand written) - HOLOTYPE [;] Heterospilus [;] arawak [;] P. Marsh. Deposited in ESUW.

#### Paratypes.

1 ♀, Costa Rica: Guanacaste [;] Est. Biol. Maritza, 600m [;] i.1997, C. Zuniga, Malaise [;] L.N. 326900-373000 #47557 (ESUW). 1 ♀, Costa Rica: Guanacaste, ACT [;] Bagaces, P.N. Palo Verde [;] Sect. Catalina, 0–50m, de Luz [;] 8–12.xi.1999, I. Jimenez [;] L.N. 260952-385020 #53252 (ESUW).

#### Comments.

The dark brown body, granulate but shining mesoscutum and white annulus on the flagellum are distinctive for this species.

#### Etymology.

Named for the Arawak, an indigenous people of Surinam.

**Figure 136.**
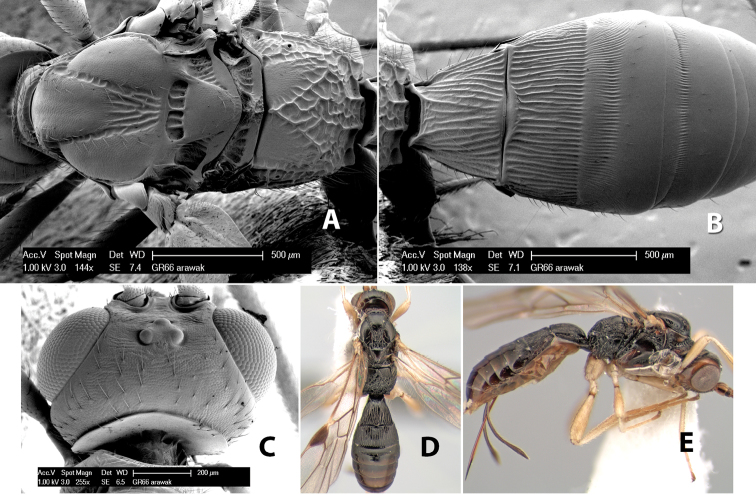
*Heterospilus arawak* Marsh, sp. n.: **A–C** paratype **D–E** holotype.

### Heterospilus aubreyae

Marsh sp. n.

http://zoobank.org/3AEFC103-669F-400B-8EF6-1BCBAC98BABA

http://species-id.net/wiki/Heterospilus_aubreyae

Figure 137

#### Female.

Body size: 2.5–3.0 mm. Color: body dark brown, apical metasomal terga usually lighter brown; scape yellow, lateral brown stripe very weakly indicated or absent, flagellum brown, apical 5–7 flagellomeres white, apical most one often brown; wing veins including stigma brown; legs yellow, apical 3/4 of femora brown. Head: vertex granulate, sometimes weakly so; frons granulate; face granulate; temple in dorsal view narrow, sloping behind eye, width less than 1/2 eye width; malar space equal to or slightly greater than 1/4 eye height; ocell-ocular distance about 2.5 times diameter of lateral ocellus; 18–24 flagellomeres. Mesosoma: mesoscutal lobes weakly granulate and shining; notauli smooth, area where they meet usually unsculptured; scutellum granulate; prescutellar furrow usually with 1 distinct median cross carina, occasionally with weaker carinae on each side; mesopleuron granulate; precoxal sulcus smooth, shorter than mesopleuron; venter granulate; propodeum with basal median areas margined, granulate, basal median carina absent, areola indistinctly margined, areolar area rugose, lateral areas entirely rugose. Wings: fore wing vein r shorter than vein 3RSa, vein 1cu-a beyond vein 1M; hind wing vein SC+R absent, vein M+CU shorter than vein 1M. Metasoma: first tergum longitudinally costate, length equal to or greater than apical width; second tergum longitudinally costate; anterior transverse groove present, straight; posterior transverse groove present; third tergum entirely smooth; terga 4–7 smooth; ovipositor about as long a metasomal tergum 1.

#### Holotype female.

Top label (white, printed) - COSTA RICA-Heredia Prov. [;] La Selva Biological Station [;] 10°26'N, 84°01'W, 100m [;] Canopy fogging 19 [;] 8.x.1994 [;] Project ALAS (FVK19); second label (red, partially printed and hand written) - HOLOTYPE [;] Heterospilus [;] aubreyae [;] P. Marsh. Deposited in ESUW.

#### Paratypes.

9 ♀♀, same data as holotype with additional dates of 24.x.1994, 20.x.1994, 15.x.1994, 10.x.1994 and 17.x.1994 (ESUW). 5 ♀♀, - COSTA RICA-Heredia Prov. [;] La Selva Biological Station [;] 10°26'N, 84°01'W, 100m [;] Malaise trap 07, #281 and 309 and 04, #390 [;] 1.xii.1993, 3.i.1994 and 30.vi.1995 [;] Project ALAS (M.07.281, 309 and M.04.390) (ESUW). 1 ♀, COSTA RICA: Puntarenas [;] Rd. to Rincon, 10km W. [;] of Pan-Amer. Hwy, 100m [;] III-V 1989, Hanson & Gauld (ESUW). 1 ♀, COSTA RICA: Puntar [;] Golfo Dulce, 3km [;] S.W. Rincon, 10m [;] IX-XI 1989, Hanson (ESUW). 3 ♀♀, COSTA RICA: [;] Puntar. Golfo Dulce [;] 24km W Piedras Blancas [;] 200m, vi–viii 1989 [;] Hanson (ESUW). 1 ♀, Costa Rica, Puntarenas [;] Pen. Osa, 27km. s. Pto. [;] Jimenez, Rio Piro, 75m [;] XI-1990 P. Hanson (ESUW). 2 ♀♀, COSTA RICA: Puntarenas [;] Reserva Forestal Golfo Dulce [;] 3km southwest of Rincon [;] 10m, July 1991, P. Hanson [;] primary forest, Malaise trap (ESUW). 3 ♀♀, COSTA RICA: Puntar [;] Golfo Dulce, 10km W [;] Piedras Blancas, 100m [;] VI-VIII 1989, Hanson (ESUW). 1 ♀, Costa Rica: Puntarenas [;] R.F. Golfo Dulce, 24km. [;] W. Piedras Blancas, 200m [;] I.1993, P. Hanson (ESUW). 4 ♀♀, Costa Rica: Puntarenas, ACO [;] Golfito, P.N. Corcovado, 745m [;] Est. Agujas, Cerro Rincon [;] 15.v–15.vi.1999, J. Azofeifa [;] L.S. 276900–521500 #52744 [;] Malaise trap (ESUW). 1 ♀, Costa Rica: Prov. Puntarenas [;] ACO, Golfito, PN Corcovado [;] Est. Agujas, Cerro Rincon, 745m [;] 17.iv–16.v.1999, J. Azofeifa [;] L.S. 276900–521500 #52781 (ESUW). 1 ♀, Costa Rica: Puntarenas [;] San Vito, Estac. Biol. Las Alturas, 1500m [;] xii.1991, Paul Hanson (ESUW). 2 ♀♀, COSTA RICA: Puntar [;] Golfo Dulce 3km SW [;] Rincon [;] 10m, xii 1989-iii 1990 [;] Col. Paul Hanson (ESUW). 11 ♀♀, Costa Rica: Puntar. [;] P.N. Corcovado [;] Est. Sirena, 50m [;] x-xii 1990 (ESUW). 1 ♀, Costa Rica: Puntarenas [;] Res. Forestal Golfo Dulce [;] 3km. SW Rincon, 10m [;] iv.1993, P. Hanson [;] Malaise, primary forest (ESUW). 1 ♀, COSTA RICA: Puntar [;] Golfo Dulce 24km W. [;] Piedras Blancas [;] 200m, xii 89-iii 1990 [;] Col. Paul Hanson (ESUW). 1 ♀, COSTA RICA: [;] Heredia, Chilamate [;] 75m, xi 1989 [;] Hanson & Godoy (ESUW). 1 ♀, Costa Rica, San Jose [;] Zurqui De Moravia [;] 1600m, VII-1996 [;] P. Hanson (ESUW). 1 ♀, Costa Rica, San Jose [;] Zurqui De Moravia [;] 1600m, February 1996 [;] P. Hanson, Malaise (ESUW). 2 ♀♀, COSTA RICA: San José [;] P.N. Braulio Carillo [;] 9.5km E tunnel, 1000m [;] 1.iii 1990 and VI/1989 P. Hanson (ESUW). 9 ♀♀, COSTA RICA, Limon [;] 16km W Guapiles [;] 400m, II/1989, April 1989, III 1989 and viii-x 1990 [;] col. Paul Hanson (ESUW). 1 ♀, Costa Rica: Limon [;] 30km N Cariari, 100m [;] Sector Cocori, Malaise [;] iii.1995, E. Rojas #4524 [;] L.N. 286000–567500 (ESUW). 2 ♀♀, COSTA RICA: Limon [;] P.N. Tortuguero [;] Est. 4-esquinas, 0m [;] VI-VIII 1989 and iv-v 1989, J. Solano (ESUW). 1 ♀, Costa Rica: Cartago [;] Braulio Carillo N.P. [;] 600m, 25.iii.1990 [;] J. S. Noyes, coll. (ESUW). 1 ♀, Costa Rica: Alajuela, ACA [;] San Carlos, R.F. Arenal [;] Sebdero Pilon, 600m, Malaise [;] 14.x–3.xii.1998, G. Carballo [;] L.N. 269100–457900 #53365 (ESUW). 1 ♀, top label - Costa Rica: Guanacaste [;] Santa Rosa Natl. Park [;] 300m, ex. Malaise trap [;] Site #: SE-6-C [;] Dates; 18.x–8.xi.1986 [;] I.D. Gauld & D. Janzen; second label - [SE] Bosque San Emilio [;] 50yr old deciduous forest [;] [C] more or less fully [;] shaded as possible (ESUW). 1 ♀, Costa Rica: Guanacaste [;] P.N. Guanacaste [;] below Pitilia, 500m [;] 7–8.iii.1990 (ESUW). 3 ♀♀, top label - Costa Rica: Guanacaste [;] Santa Rosa National Pk. [;] 300m, Malaise, Ian Gauld [;] 27.ix–18.x.1986 and 5–26.vii.1986; second label - Bosque San Emilio [;] 50yr old deciduous [;] forest. Sun; third label - SE-7-O [;] 27.ix–18.x.86 and 5–26.vii.86 (ESUW). 1 ♀, top label - Costa Rica: Guanacaste [;] Santa Rosa National Pk. [;] 300m, Malaise, Ian Gauld [;] 10–31.i.1987; second label - Bosque San Emilio [;] 50yr old deciduous [;] forest. Full Shade; third label - SE-6-C [;] 10–31.i.87 (ESUW). 1 ♀, top label - Costa Rica: Guanacaste [;] 9km S. Santa Cecilia [;] Estacion Pitilia, 700m [;] vi.1996, Malaise trap; second label - C. Moraga & P. Rios [;] L.N. 330200–380200 [;] #47562 (ESUW). 1 ♀, top label - COSTA RICA, Heredia: [;] Est. Biol. La Selva, 50- [;] 150m, 10°26'N, 84°01'W [;] Mar. 1996, INBio-OET; second label - 15 Marzo 1996 [;] Bosque primario [;] M/03/593 (INBC).

#### Comments.

The smooth notauli and the unsculptured area where they meet and the bicolored legs are distinctive for this species.

#### Etymology.

Named for my grand-daughter, Aubrey Holoski.

**Figure 137.**
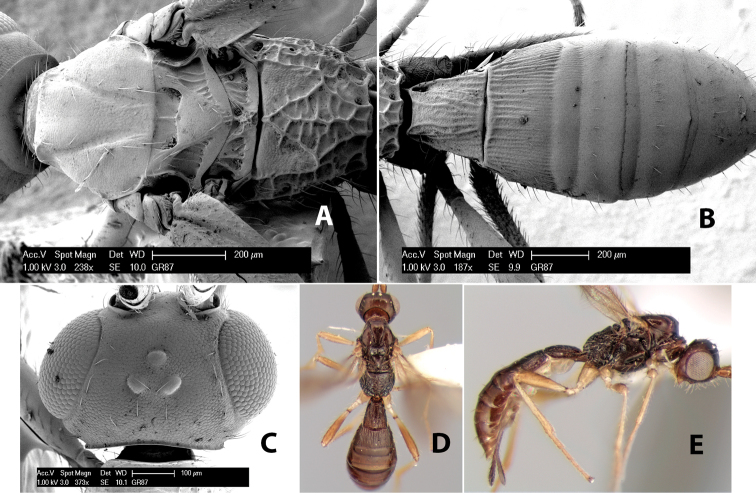
*Heterospilus aubreyae* Marsh, sp. n.: **A–C** paratype **D–E** holotype.

### Heterospilus austini

Marsh sp. n.

http://zoobank.org/DC4B8BC3-B584-4AEB-93AE-772069B7400C

http://species-id.net/wiki/Heterospilus_austini

Figure 138

#### Female.

Body size: 3.0–3.5 mm. Color: head brown; scape yellow with weak lateral longitudinal brown stripe, this stripe often absent, flagellum brown; mesosoma brown; metasoma brown, apical terga sometimes lighter; wing veins brown, stigma bicolored brown with yellow at base and apex and often along anterior edge; legs yellow. Head: vertex granulate, usually with weak striations or rugae behind ocelli; frons granulate; face rugose; temple in dorsal view broad but not bulging behind eye, width about equal to eye width; malar space greater than 1/4 eye height; ocell-ocular distance greater than 2.5 times diameter of lateral ocellus; 21–24 flagellomeres. Mesosoma: mesoscutal lobes granulate; notauli scrobiculate, meeting at scutellum in triangular costate-rugose area; scutellum granulate; prescutellar furrow with 3–5 cross carinae; mesopleuron granulate; precoxal sulcus weakly scrobiculate or smooth, shorter than mesopleuron; venter granulate; propodeum with basal median areas not distinctly margined, granulate, basal median carina absent, areola not distinctly margined, areolar area areolate, lateral areas entirely rugose. Wings: fore wing vein r shorter than vein 3RSa, vein 1cu-a beyond vein 1M; hind wing vein SC+R present, vein M+CU equal in length to vein 1M. Metasoma: first tergum longitudinally costate, medially costate-rugose; second tergum longitudinally costate; anterior transverse groove present, straight; posterior transverse groove present; third tergum costate basally, granulate apically; terga 4–7 weakly granulate; ovipositor equal to 1/2 length of metasoma.

#### Holotype female.

Top label (white, partially printed and hand written) - Costa Rica: Guanacaste [;] Santa Rosa Natl. Park [;] 300m, ex. Malaise trap [;] Site #: BH-10-C [;] Dates: 4-24.v.1986 [;] I.D. Gauld & D. Janzen; second label (white, printed) - [BH] Bosque Humedo [;] mature evergreen dry forest [;] [C] more or less fully [;] shaded as possible; third label (red, partially printed and hand written) - HOLOTYPE [;] Heterospilus [;] austini [;] P. Marsh. Deposited in ESUW.

#### Paratypes.

14 ♀♀, same data as holotype except: dates of 13.iv.1986, 8.ii–2.iii.1986, 9–26.x.1985, 2–23.iii.1986, 13.iv–4.v.1986 and 23.iii–13.iv.1986; second labels of [BH] Bosque Humedo [;] mature evergreen dry forest [;] [O] in clearing, fully [;] isolated part of day, and [H] open regenerating [;] woodland <10 years old [;] [C] more or less fully [;] shaded as possible (ESUW). 1 ♀, Costa Rica: San Jose [;] Cerro de la Muerte [;] 19km S 3 W Empalme [;] 2600m, November 1992 [;] P. Hanson, Malaise (ESUW). 1 ♀, Costa Rica: San Jose [;] 2km W. Empalme [;] 2300m, July 1995 [;] P. Hanson, Malaise (ESUW). 1 ♀, Costa Rica: Guanacaste, ACT [;] Bagaces, P.N. Palo Verde [;] Sec. P. Verde, 200 NE Est. [;] Extremo E de Campo de [;] Aterrizaje, 0–50m, Malaise [;] 8.xi–9.xii.1999, I. Jimenez [;] L.N. 260952-385020 #54241 (ESUW). 2 ♀♀, S.RosaPark. Guan. [;] C. Rica 6 and 22 Feb 78 [;] D.H. Janzen [;] Riparian (AEIC).

#### Comments.

The rugose face, weak striations behind ocelli and the brown body are distinctive for this species. This species is very similar to *Heterospilus faustinus* Marsh from Venezuela which differs in having the apical metasomal terga distinctly granulate and dull (weakly granulate and shining in *Heterospilus austini*) and the second metasomal tergum yellow and distinctly lighter than the other terga (metasoma is entirely brown in *Heterospilus austini*).

#### Etymology.

Named for my colleague and friend from “down under”, Andy Austin.

**Figure 138.**
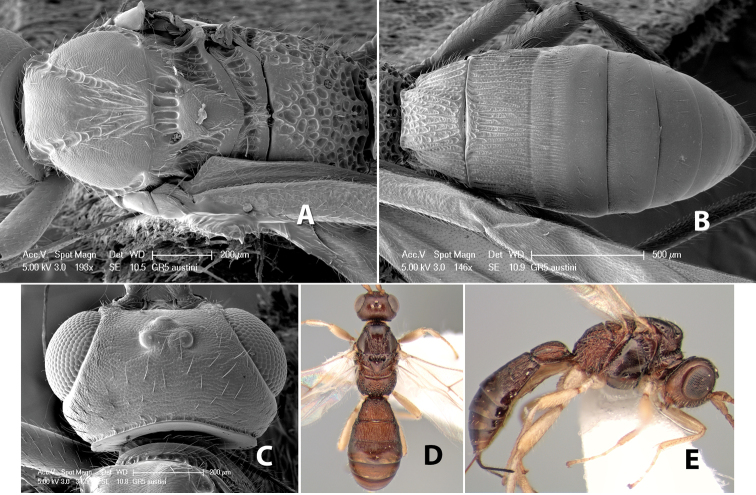
*Heterospilus austini* Marsh, sp. n.: **A–C, E** paratype **D** holotype.

### Heterospilus bacchus

Marsh sp. n.

http://zoobank.org/B1CB7546-D8F5-4336-B3AF-D0D8B189A12F

http://species-id.net/wiki/Heterospilus_bacchus

Figure 139

#### Female.

Body size: 3.0 mm. Color: body dark brown, metasomal tergum 2 yellow medially, tergum 5 at apex and terga 6–7 entirely yellow; scape yellow with lateral longitudinal brown stripe, flagellum brown with apical white annulus, apical 5–7 flagellomeres brown; wing veins including stigma brown; legs yellow. Head: vertex granulate; frons granulate; face granulate; temple in dorsal view narrow, sloping behind eye, width less than 1/2 eye width; malar space slightly greater than 1/4 eye height; ocell-ocular distance slightly greater than 2.5 times diameter of lateral ocellus; 21–24 flagellomeres. Mesosoma: mesoscutal lobes granulate; notauli weakly scrobiculate, meeting at scutellum in weak small costate area, occasionally nearly absent posteriorly; scutellum granulate; prescutellar furrow with 5 cross carinae; mesopleuron granulate; precoxal sulcus smooth, shorter than mesopleuron; venter granulate; propodeum with basal median areas margined, granulate, basal median carina absent, areola not margined, areolar area rugose, lateral areas rugose with small granulate area anteriorly. Wings: fore wing vein r shorter than vein 3RSa, vein 1cu-a slightly beyond vein 1M; hind wing vein SC+R absent, vein M+CU shorter than vein 1M. Metasoma: first tergum longitudinal costate-granulate laterally, granulate medially, length equal to apical width; second tergum longitudinally costate-granulate; anterior transverse groove present, straight; posterior transverse groove present; third tergum costate basally, granulate apically; terga 4–7 granulate; ovipositor equal to or shorter than metasomal tergum 1.

#### Holotype female.

Top label (white, printed) - COSTA RICA: Puntarenas [;] Reserva Forestal Golfo Dulce [;] 3km SW of Rincon, 10m [;] Mar-April 1992, P. Hanson [;] primary forest, Malaise trap; second label (red, partially printed and hand written) - HOLOTYPE [;] Heterospilus [;] bacchus [;] P. Marsh. Deposited in ESUW.

#### Paratypes.

3 ♀♀, same data as holotype (some lines and/or words in different order), additional dates of July 1991, viii.1991 and iii.1993 (ESUW). 1 ♀, Costa Rica: Heredia [;] Braulio Carrillo N.P. [;] 250–500m IV.10.85 [;] Henri Goulet (AEIC). 2 ♀♀, COSTA RICA: *Punt-* [;] *arenas*. 7km SW Rincon [;] 31.v–7.vi.1998. B. Brown [;] & V. Berezovskiy. Mal. [;] Trp. #3, 1° forest and #5, 2nd growth (AEIC).

#### Comments.

The granulate metasomal tergum 1 and the short ovipositor are distinctive for this species.

#### Etymology.

Named for Bacchus, the Roman god of wine, sensual pleasure and truth.

**Figure 139.**
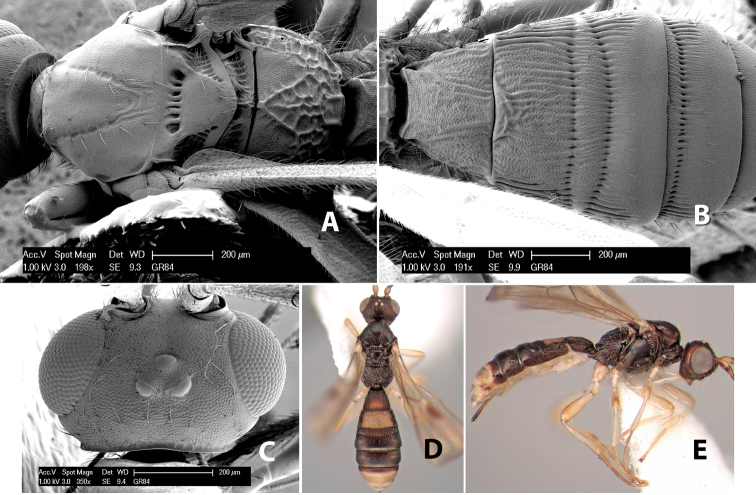
*Heterospilus bacchus* Marsh, sp. n.: **A–C** paratype **D–E** holotype.

### Heterospilus barbalhoae

Marsh sp. n.

http://zoobank.org/527CF5D8-5056-4AB2-AD39-29A740727F25

http://species-id.net/wiki/Heterospilus_barbalhoae

Figure 140

#### Female.

Body size: 2.0–2.5 mm. Color: head dark brown, face usually lighter brown; scape yellow with lateral longitudinal brown stripe, flagellum brown with white annulus apically, apical 2–5 flagellomeres brown; mesosoma and metasoma dark brown; wing veins including stigma brown; legs yellow. Head: vertex granular; frons granular; face granular; temple in dorsal view narrow but not distinctly sloping behind eye, width less than 1/2 eye width; malar space greater than 1/4 eye height; ocell-ocular distance about twice diameter of lateral ocellus; 20–22 flagellomeres. Mesosoma: mesoscutal lobes granulate; notauli scrobiculate, meeting posteriorly in small triangular rugose or costate area; scutellum granulate; prescutellar furrow with 3–5 cross carinae; mesopleuron granulate; precoxal sulcus smooth, shorter than mesopleuron; venter granulate; propodeum with basal median areas distinct but usually not distinctly margined, granulate, basal median carina absent, areola not distinctly margined, areolar area areolate-rugose, lateral areas entirely rugose. Wings: fore wing vein r shorter than vein 3RSa, vein 1cu-a beyond vein 1M; hind wing vein SC+R present, vein M+CU shorter than vein 1M. Metasoma: first tergum longitudinally costate, often rugose medially, apical width equal to or slightly less than length; second tergum longitudinally costate; anterior transverse groove present, straight; posterior transverse groove present; third tergum costate basally, granulate apically; terga 4-7 granulate; ovipositor shorter than or often equal to length of metasomal tergum 1.

#### Holotype female.

Top label (white, printed) - COSTA RICA: Puntar [;] Golfo Dulce 24km W [;] Piedras Blancas [;] 200m, ix–xi.1989 [;] Col. Paul Hanson; second label (red, partially printed and hand written) - HOLOTYPE [;] Heterospilus [;] barbalhoae [;] P. Marsh. Deposited in ESUW.

#### Paratypes.

10 ♀♀, same data as holotype with additional dates of xii.89-iii.1990, Feb. 1992, I.1993, ii.1993 and III.1993 (ESUW). 1 ♀, Costa Rica: Prov. Puntarenas [;] ACO, Golfito, PN Corcovado [;] Est. Agujas, Cerro Rincon, 745m [;] 17.iv–16.v.1999, J. Azofeifa [;] L.S. 276900-521500 #52781 (ESUW). 1 ♀, COSTA RICA: Puntarenas [;] Reserva Forestal Golfo Dulce [;] 3km southwest of Rincon [;] 10m, July 1991, P. Hanson [;] primary forest, Malaise trap (ESUW). 1 ♀, Costa Rica, Puntarenas [;] Res. Forestal Golfo Dulce [;] 3km SW Rincon, 10m [;] ii.1993, P. Hanson [;] Malaise, primary forest (ESUW). 2 ♀♀, Costa Rica: Puntarenas [;] R.F. Golfo Dulce, [;] 3km. SW. Rincon, 10m. [;] iii.1993 Paul Hanson coll. [;] Malaise, primary forest (ESUW). 1 ♀, Costa Rica: Puntarenas [;] R.F.Golfo Dulce, [;] 3km. SW. Rincon, 10m, [;] ii.1992, Paul Hanson (ESUW). 1 ♀, Costa Rica: Puntarenas [;] San Vito, Estac. Biol. [;] Las Alturas, 1500m [;] ii.1992, Paul Hanson (ESUW). 1 ♀, Costa Rica: Puntar. [;] P.N. Corcovado [;] Est. Sirena, 50m [;] x-xii 1990 (ESUW). 1 ♀, Costa Rica: Limon, Sec. Cocori [;] 30Km al N. Cariari, 100m [;] xii.1994, E. Rojas, Malaise [;] L.N. 286000-567500 #4525 (ESUW). 1 ♀, COSTA RICA: Limón [;] 16km W. Guápiles [;] 400m, iii–v 1990 [;] col. Paul Hanson (ESUW). 1 ♀, Costa Rica: Limon, ACLAC [;] Central Res. Biol. Hitoy Cerere [;] Est. Hitoy Cerere, Send. Espavel [;] 300m, 17.iv–17.v.1990, F. Umana [;] L.S. 401500-570200 #52777 Mal. (ESUW). Costa Rica: Limon, ACLAC [;] Central, R.B. Hitoy Cerere [;] Send, Espavel, 560m [;] 19.v–19.vi.1998, E. Rojas [;] L.S. 400702-570120 #52200 [;] Malaise trap (ESUW). 1 ♀, COSTA RICA-Heredia Prov. [;] La Selva Biological Station [;] 10°26'N, 84°01'W, 100m [;] Malaise trap 01, #376 [;] 15.iii.1994 [;] Project ALAS (M.01.376) (ESUW). 1 ♀, top label - COSTA RICA: Heredia [;] Est. Biol. La Selva, 50- [;] 150m, 10°26'N, 84°01'W [;] Apr. 1993, INBio-OET; second label - 2 Abril 1993 [;] Bosque secundario [;] M/14/061; third label - INBio bar code (ESUW). 1 ♀, Costa Rica, Heredia [;] Puerto Viejo, 100m [;] OTS-La Selva [;] III-1991 P. Hanson (ESUW). 1 ♀, Costa Rica: Cartago [;] Turrialba, CATIE [;] 14-15 March 1990 [;] 700m, J.S. Noyes (ESUW). 1 ♀, top label - Costa Rica: Guanacaste [;] Santa Rosa Natl. Park [;] 300m, ex. Malaise trap [;] Site #H-1-O [;] Dates: 8-29.xi.1986 [;] I.D. Gauld & D. Janzen; second label - [H] open regenerating [;] woodland, 10 years old [;] [O] in clearing, fully [;] isolated part of day (ESUW). 1 ♀, top label - COSTA RICA, Heredia: [;] Est. Biol. La Selva, 50- [;] 150m, 10°26'N, 84°01'W [;] Mar 1998, INBio-OET; second label - 19 Marzo 1998 [;] Borde suampo [;] M. 18/18/704 (INBC). 1 ♀, top label - COSTA RICA, Heredia: [;] Est. Biol. La Selva, 50- [;] 150m, 10°26'N, 84°01'W [;] Sep 1998, INBio-OET; second label - 03 Setiembre [;] Borde Suampo [;] M/18/716 (INBC). 2 ♀♀, Costa Rica: Puntarenas [;] RF Golfo Dulce el 200m [;] 24km W Piedras Blancas [;] P. Hanson ix.1992 and vi.1993 (TAMU).

#### Comments.

The dark brown body and the flagellum with white annulus near tip are distinctive for this species.

#### Etymology.

Named for my friend, colleague and former student of the Doryctinae, Sandra Barbalho.

**Figure 140.**
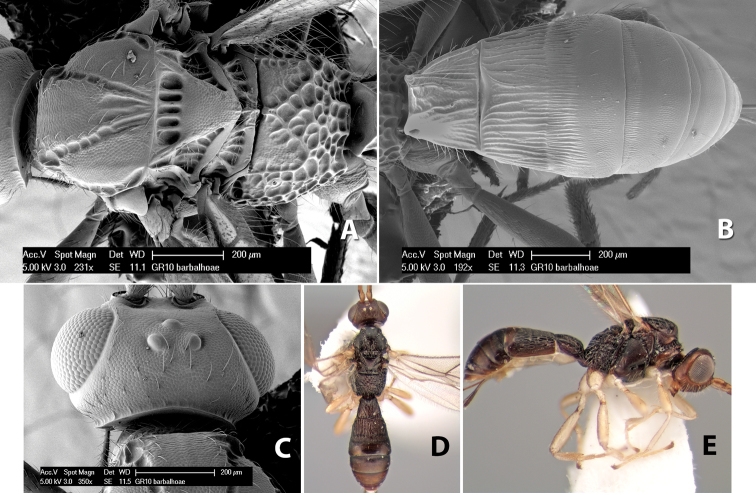
*Heterospilus barbalhoae* Marsh, sp. n.: **A–D** paratype **E** holotype.

### Heterospilus bennetti

Marsh sp. n.

http://zoobank.org/DA4EA397-EF2B-4F68-9F04-2AE9061E11B1

http://species-id.net/wiki/Heterospilus_bennetti

Figure 141

#### Female.

Body size: 2.5–3.0 mm. Color: body dark brown, metasomal tergum 2 usually yellow medially; scape yellow with lateral longitudinal brown stripe; flagellum brown with apical white annulus, apical 5–7 flagellomeres brown; wing veins brown, stigma usually brown, rarely honey yellow of bicolored brown with yellow at base, apex and along apical margin; legs yellow. Head: vertex granulate; frons granulate; face granulate; temple in dorsal view narrow, sloping behind eye, width less than 1/2 eye width; malar space equal to 1/4 eye height; ocell-ocular distance slightly greater than 2.5 timed diameter of lateral ocellus; flagellomeres. Mesosoma: mesoscutal lobes weakly granulate and shining; notauli scrobiculate, meeting at scutellum in triangular costate area, median longitudinal dimple-like depression present; scutellum smooth; prescutellar furrow with 5 cross carinae; mesopleuron granulate; precoxal sulcus smooth, shorter than mesopleuron; venter smooth; propodeum with basal median areas margined, granulate, basal median carina absent, areola usually weakly but distinctly margined, often not margined, areolar area areolate-rugose, lateral areas entirely rugose, propodeum with distinct tubercle above hind coxa. Wings: fore wing vein r shorter than vein 3RSa, vein 1cu-a beyond vein 1M; hind wing vein SC+R absent, vein M+CU shorter than vein 1M. Metasoma: first tergum longitudinally costate, length equal to apical width; second tergum longitudinally costate, lateral costae angled toward midline, raised smooth area medially at base of tergum; anterior transverse groove present, straight; posterior transverse groove present; third tergum costate basally, smooth apically; terga 4–7 smooth; ovipositor equal to length of metasomal terga 1 and 2 combined.

#### Holotype female.

Top label (white, printed) - COSTA RICA: San Jose [;] Ciudad Colon [;] 800m, vi–vii 1990 [;] Col. Luis Fournier; second label (red, partially printed and hand written) - HOLOTYPE [;] Heterospilus [;] bennetti [;] P. Marsh. Deposited in ESUW.

#### Paratypes.

11 ♀♀, Costa Rica, Cartago [;] Turrialba, La Isabel [;] 650m, Café, IV-1994 [;] M. Cerda & P. Hanson (ESUW). 1 ♀, Costa Rica: Puntarenas [;] San Vito, Estac. Biol [;] Las Alturas, 1500m [;] vi.1992, traps #1 + #2, [;] Malaise, Paul Hanson (ESUW). 1 ♀, Costa Rica: Puntarenas [;] San Vito, Las Cruces [;] Wilson Botanical Gardens [;] 18–22.iii.1990, 1150m [;] J.S. Noyes (ESUW). 1 ♀, COSTA RICA: Puntarenas [;] Reserva Forestal Golf Dulce [;] 3km southwest of Rincon [;] 10m, July 1991, P. Hanson [;] primary forest, Malaise trap (ESUW). 4 ♀♀, top label - Costa Rica: Guanacaste [;] Santa Rosa Natl. Park [;] 300m, ex. Malaise trap [;] Site #: BH-10-C and blank [;] Dates: 6–27.ix.1986, 13.iv–4.v.1986, 7–28.xii.1985 and 18.i–8.ii.1986 [;] I.D. Gauld & D. Janzen; second label - [BH] Bosque Humedo [;] mature evergreen dry forest [;] [C] more or less fully [;] shaded as possible (ESUW). 2 ♀♀, top label - Costa Rica: Guanacaste [;] Santa Rosa Natl. Park [;] 300m, ex. Malaise trap [;] Site #: H-4-C and #2 [;] Dates: 4–24.v.1986 and 13.iv–4.v.1986 [;] I.D. Gauld & D. Janzen; second label - [H] open regenerating [;] woodland <10 years old [;] [C] more or less fully [;] shaded as possible (ESUW). 3 ♀♀, top label - Costa Rica: Guanacaste [;] Santa Rosa Natl. Park [;] 300m, ex. Malaise trap [;] Site #: H-1-O and H-3-O [;] Dates: 20.xii.86–10.i.1987, 14.vi–5.vii.1986 and 10–31.i.1987 [;] I.D. Gauld & D. Janzen; second label - [H] open regenerating [;] woodland <10 years old [;] [O] in clearing fully [;] isolated part of day (ESUW). 4 ♀♀, top label - Costa Rica: Guanacaste [;] Santa Rosa Natl. Park [;] 300m, ex. Malaise trap [;] Site #: BH-9-O and blank [;] Dates: 16.xi–7.xii.1985, 2–23.iii.1986 and 8–29.vi.1986 [;] I.D. Gauld & D. Janzen; second label - [BH] Bosque Humedo [;] mature evergreen dry forest [O] in clearing fully [;] isolated part of day (ESUW). 3 ♀♀, top label - Costa Rica: Guanacaste [;] Santa Rosa Natl. Park [;] 300m, ex. Malaise trap [;] Site #: BH-10-C and 6 [;] Dates: 8.ii–2.iii.1986 and 16.ix–7.xii.1985 [;] I.D. Gauld & D. Janzen; second label - [SE] Bosque San Emilio [;] 50yr old deciduous forest [;] [C] more or less fully [;] shaded as possible (ESUW). 4 ♀♀, top label - Costa Rica: Guanacaste [;] Santa Rosa Natl. Park [;] 300m, ex. Malaise trap [;] Site #: SE-O-5 and blank [;] Dates: 18.x–8.xi.1986 and 13.ix–4.x.1986 [;] I.D. Gauld & D. Janzen; second label - [SE] Bosque San Emilio [;] 50yr old deciduous forest [;] [C] more or less fully [;] shaded as possible (ESUW). 1 ♀, top label - Costa Rica: Puntarenas [;] Santa Rosa National Pk. [;] 300m, Malaise, Ian Gauld [;] 27.ix–18.x.1986; second label - Bosque San Emilio [;] 50 yr Old deciduous [;] Forest, Full Shade; third label - SE-8-C [;] 27.ix–18.x.86 (ESUW). 3 ♀♀, Costa Rica: Guanacaste [;] P.N. Guanacaste [;] below Pitilia, 500m [;] 7-8.iii.1990, J. S. Noyes (ESUW). 1 ♀, Costa Rica: Guanacaste [;] Est. Biol. Maritza, 600m [;] i.1997, C. Zuniga, Malaise [;] L.N. 326900-373000 #47557 (ESUW). 1 ♀, Costa Rica: Guanacaste, ACT [;] Bagaces, P.N. Palo Verde [;] Sec. P. Verde, 150 de la Est. [;] 0-50m, 17.viii–13.ix.1999 [;] I. Jimenez, Malaise #53257 [;] L.N.260952-385020 (ESUW). 1 ♀, COSTA RICA, Puntar. [;] Golfo Dulce, 24km W. [;] PiedrasBlancas, 200m [;] XII-89-III 1990 Hanson (MICR). 1 ♀, COSTA RICA, SanJosé [;] Ciudad Colon, 800m [;] II 1990 [;] col. Luis Fournier (MICR). 3 ♀♀, Costa Rica: Puntarenas [;] San Vito - Las Cruces [;] 5-VI-1988 1200m [;] P. Hanson (TAMU). 4 ♀♀, Costa Rica [;] San Vito, Puntarenas [;] 19 December 1974 and 1–5 January 1975 [;] M. Palmer (TSMU). 2 ♀♀, COSTA RICA: Puntarenas [;] RF Golfo Dulce el 200m [;] 24km W Piedras Blancas [;] P. Hanson x.1992 (TAMU).

#### Comments.

The raised area medially at the base of metasomal tergum 2, the costae angled toward the midline of tergum 2 and the dimple-like depression at the junction of the notauli are distinctive for this species.

#### Etymology.

Named for my grand-son, Bennett Holoski.

**Figure 141.**
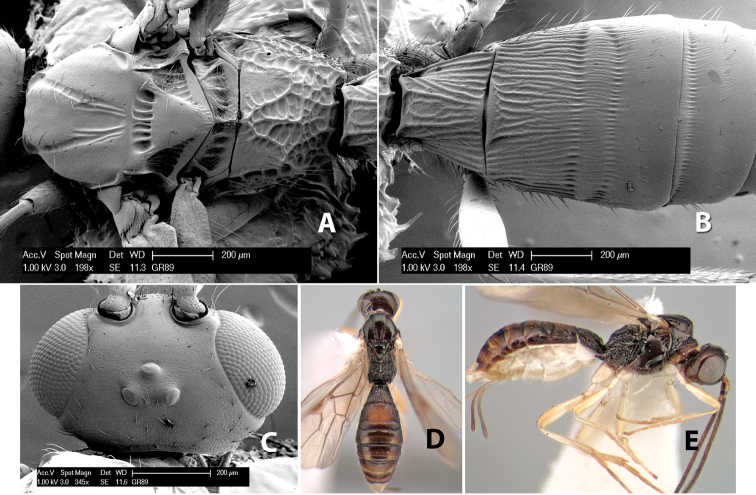
*Heterospilus bennetti* Marsh, sp. n.: **A–C** paratype **D–E** holotype.

### Heterospilus bicolor

Marsh sp. n.

http://zoobank.org/7507A6A1-1674-49A5-9B2A-6DD0B5850100

http://species-id.net/wiki/Heterospilus_bicolor

Figure 142

#### Female.

Body size: 3.0–4.0 mm. Color: head dark brown, face honey yellow, malar space and lower temple yellow; scape yellow with lateral longitudinal brown stripe, flagellum brown with white annulus near apex, apical 3–5 flagellomere brown; mesosoma dark brown; metasomal terga 1, 3 and 4 dark brown, tergum 2 yellow medially, brown laterally, terga 5–7 yellow; wing veins brown, stigma brown with yellow at apex; legs yellow. Head: vertex granulate with distinct transverse rugae behind ocellus; frons granulate or granulate-costate; face striate; temple in dorsal view narrow, sloping behind eye, width less than 1/2 eye width; malar space greater than 1/4 eye height; ocell-ocular distance about 2.5 times diameter of lateral ocellus; 23–27 flagellomeres. Mesosoma: mesoscutal lobes granulate; notauli scrobiculate, meeting at scutellum in triangular rugose or costate-rugose area; scutellum granulate; prescutellar furrow usually with 3 cross carinae, rarely with 1 distinct median carina; mesopleuron granulate; precoxal sulcus scrobiculate, shorter than mesopleuron; venter granulate; propodeum with basal median areas margined, granulate, basal median carina present, areola not margined, areolar area rugose, lateral areas rugose apically, granulate basally, propodeum with distinct tubercle above hind coxae. Wings: fore wing vein r shorter than vein 3RSa, vein 1cu-a beyond vein 1M; hind wing vein SC+R present, vein M+CU shorter than vein 1M. Metasoma: first tergum longitudinally costate, length longer than apical width; second tergum longitudinally costate; anterior transverse groove present, straight; posterior transverse groove present; third tergum costate basally, smooth apically; terga 4–7 smooth; ovipositor as long as metasoma.

#### Holotype female.

Top label (white, printed) - Costa Rica: Prov. Puntarenas [;] ACO, Golfito, PN Corcovado [;] Est. Agujas, Cerro Rincon, 745m [;] 17.iv–16.v.1999, J. Azofeifa [;] L.S. 276900-521500 #52781; second label (red, partially printed and hand written) - HOLOTYPE [;] Heterospilus [;] bicolor [;] P. Marsh. Deposited in ESUW.

#### Paratypes.

1 ♀, COSTA RICA: Puntar. [;] P.N. Corcovado, Est. [;] Sirena, 50m [;] IV-VII 1989 (ESUW). 3 ♀♀, Costa Rica: Alajuela [;] 5km. W San Ramon [;] 1200m, April 1997 [;] O. Castro & P. Hanson (ESUW). 1 ♀, Costa Rica: Puntarenas [;] Res Forestal Golfito Dulce [;] 3km, SW Rincon, 10m [;] ii.1993, P. Hanson [;] Malaise, primary forest (ESUW). 1 ♀, COSTA RICA: Limon [;] P.N. Tortuguero [;] Est. 4-esquinas, 0m [;] VI-VIII 1989, Solano (ESUW).

#### Comments.

The bicolored brown and yellow body and costate-granulate vertex are distinctive for the species

#### Etymology.

The specific name is in reference to the bicolored antennae, head, metasomal terga and stigma.

**Figure 142.**
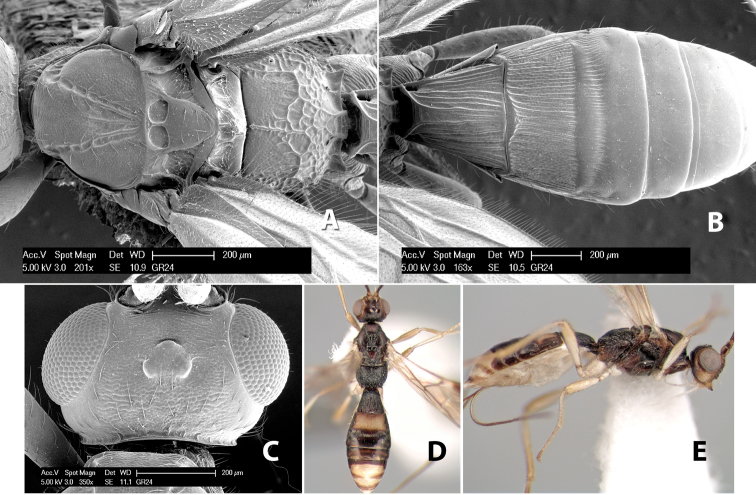
*Heterospilus bicolor* Marsh, sp. n.: **A–D** paratype **E** holotype.

### Heterospilus braeti

Marsh sp. n.

http://zoobank.org/C63F4D1C-7C13-4E6E-BC3E-664A67BC1AF3

http://species-id.net/wiki/Heterospilus_braeti

Figure 143

#### Female.

Body size: 2.0–2.5 mm. Color: head and mesosoma dark brown, metasoma terga mostly honey yellow or brown, usually dark brown laterally, terga 4-7 often darker than anterior terga; scape yellow without lateral brown stripe, flagellum brown with apical white annulus, apical 3–5 flagellomeres brown; wing veins brown, stigma usually light brown, often yellow; legs yellow. Head: vertex granulate; frons granulate; face granulate; temple in dorsal view sloping behind eye, width equal to 1/2 eye width; malar space greater than 1/4 eye height; ocell-ocular distance greater than 2.5 times diameter of later ocellus; 16–19 flagellomeres. Mesosoma: mesoscutal lobes granulate; notauli weakly scrobiculate or partially smooth, meeting at scutellum in small triangular costate area; scutellum granulate; prescutellar furrow with 3 cross carinae; mesopleuron granulate; precoxal sulcus smooth, shorter than mesopleuron; venter granulate; propodeum with basal median areas small and not margined, granulate, basal median carina absent, areola not margined, areolar area rugose, lateral areas entirely rugose. Wings: fore wing vein r shorter than vein 3RSa, vein 1cu-a beyond vein 1M; hind wing vein SC+R absent, vein M+CU shorter than vein 1M. Metasoma: first tergum longitudinally costate, length equal to apical width; second tergum longitudinally costate; anterior transverse groove present, straight; posterior transverse groove present; third tergum costate basally, weakly granulate apically; terga 4–7 weakly granulate; ovipositor equal to length of metasomal terga 1 and 2 combined.

#### Holotype female.

Top label (white, partially printed and hand written) - Costa Rica: Guanacaste [;] Santa Rosa Natl. Park [;] 300m, ex. Malaise trap [;] Site #: 11 [;] Dates: 13.iv–4.v.1986 [;] I.D. Gauld & D. Janzen; second label (white, printed) - [BH] Bosque Humedo [;] mature evergreen dry forest [;] [O] in clearing, fully [;] isolated part of day; third label (red, partially printed and hand written) - HOLOTYPE [;] Heterospilus [;] braeti [;] P. Marsh. Deposited in ESUW.

#### Paratypes.

2 ♀♀, same data as holotype with additional dates of 29.xi–20.xii.1986 and 28.xii.85–18.i.1986 (ESUW). 4 ♀♀, top label - Costa Rica: Guanacaste [;] Santa Rosa Natl. Park [;] 300m, ex. Malaise trap [;] Site #: 3 and blank [;] Dates: 10–31.i.1987 and 14.viii–6.ix.1986 [;] I.D. Gauld & D. Janzen; second label - [H] open regenerating woodland <10 years old [;] [O] in clearing, fully [;] isolated part of day (ESUW). 3 ♀♀, top label - Costa Rica: Guanacaste [;] Santa Rosa Natl. Park [;] 300m, ex. Malaise trap [;] Site #: SE-6-C and 6 [;] Dates: 28.xii.85–18.i.1986, 2–23.iii.1986 and 8.ii–2.iii.1986 [;] I.D. Gauld & D. Janzen; second label - [SE] Bosque San Emilio [;] 50yr old deciduous forest [;] [C] more or less fully [;] shaded as possible (ESUW). 5 ♀♀, top label - Costa Rica: Guanacaste [;] Santa Rosa Natl. Park [;] 300m, ex. Malaise trap [;] Site #: all blank [;] Dates: 28.xii.85–18.i.1986, 2–23.iii.1986 and 31.i–21.ii.1987 [;] I.D. Gauld & D. Janzen; second label - [SE] Bosque San Emilio [;] 50yr old deciduous forest [;] [O] in clearing, fully [;] isolated part of day (ESUW). 2 ♀♀, top label - Costa Rica: Guanacaste [;] Santa Rosa Natl. Park [;] 300m, ex. Malaise trap [;] Site #: BH-0-C and 10 [;] Dates: 4–24.v.1986 and 8–29.xi.1986 [;] I.D. Gauld & D. Janzen; second label - [BH] Bosque Humedo [;] mature evergreen dry forest [;] [C] more or less fully [;] shaded as possible (ESUW). 1 ♀, top label - Costa Rica: Guanacaste [;] Santa Rosa Natl. Park [;] 300m, ex. Malaise trap [;] Site #: H-2-C and 10 [;] Dates: 29.xi–20.xii.1986 [;] I.D. Gauld & D. Janzen; second label - [;] [H] open regenerating woodland <10 years old [C] more or less fully [;] shaded as possible (ESUW). 2 ♀♀, top label - Costa Rica: Guanacaste, Santa [;] Rosa Nat’l Park, Bosque San [;] Emilio, trap #5 in clearing, 300m [;] XI/18–29/1986, I. Gauld; second label - [SE] Bosque San Emilio [;] 50yr old deciduous forest [;] [O] in clearing, fully [;] isolated part of day (ESUW). 3 ♀♀, top label - Costa Rica: Guanacaste [;] Santa Rosa National Pk. [;] 300m, Malaise, Ian Gauld [;] 10–31.i.1987; second label - Bosque San Emilio [;] 50 yr. Old deciduous [;] Forest [;] Full Shade; third label - SE-8-C [;] 10–31.i.87 (ESUW). 1 ♀, top label - Costa Rica: Guanacaste [;] Santa Rosa National Pk. [;] 300m, Malaise, Ian Gauld [;] 24.v–14.vi.1986; second label - Bosque San Emilio [;] 50 yr. Old deciduous [;] Forest [;] Sun; third label - SE-7-O [;] 24.v–14.vi.86 (ESUW). 1 ♀, top label - Costa Rica: Guanacaste [;] Santa Rosa National Pk. [;] 300m, Malaise, Ian Gauld [;] 14.vi.1986; second label - Bosque Humedo [;] mature dry forest [;] high proportion [;] evergreen species [;] Sun.; third label - BH-11-O [;] 14.Vi.86 (ESUW). 1 ♀, Costa Rica, Puntarenas [;] R.F. Golfo Dulce, 24km. W. [;] Piedras Blancas, 200m [;] VI-1991, P. Hanson (ESUW). 1 ♀, Costa Rica: Puntarenas [;] Peninsula Osa, Puerto [;] Jimenez, 10m, x–xi.1991 [;] P. Hanson, Malaise trap [;] grassy, disturbed site (ESUW). 1 ♀, Costa Rica: Puntarenas [;] R.F. Golfo Dulce, 3km. SW. Rincon, 10m. [;] ii.1992, Paul Hanson (ESUW). 2 ♀♀, COSTA RICA: [;] San Jose [;] Ciudad Colon [;] 800m, iii-iv 1990 [;] Col. Luis Fournier (ESUW). 1 ♀, COSTA RICA, Alajuela [;] Inst.Tec.SantaClara [;] 150m, 24/III/1989 [;] col. Hanson & Godoy (ESUW). 1 ♀, COSTA RICA, San Jose [;] San Antonio de Escazu [;] 1300m, VI/1988 [;] Col. W. Eberhard (MICR).

#### Comments.

The short antennae, small body size and honey yellow metasoma contrasting with the dark brown mesosoma are distinctive for this species.

#### Etymology.

Named for my colleague and fellow braconidologist, Yves Braet.

**Figure 143.**
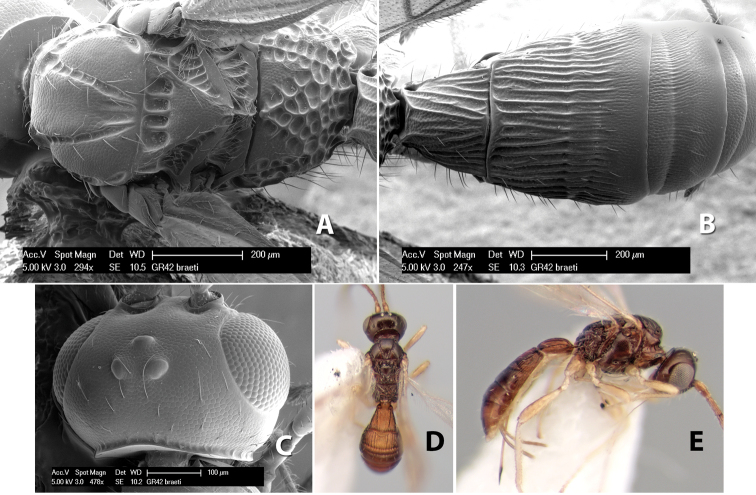
*Heterospilus braeti* Marsh, sp. n.: **A–C** paratype **D–E** holotype.

### Heterospilus cangrejaensis

Marsh sp. n.

http://zoobank.org/17984DD4-D409-4354-A4DB-92BCBD30175E

http://species-id.net/wiki/Heterospilus_cangrejaensis

Figure 144

#### Female.

Body size: 3.0–4.0 mm. Color: head dark brown, scape yellow with lateral longitudinal brown stripe, flagellum brown with white annulus near tip but apical 3–5 flagellomere brown; mesosoma dark brown, mesoscutal lobes, propleuron and venter usually lighter brown; metasomal terga dark brown, apical terga usually yellow; wing veins including stigma brown; legs yellow. Head: vertex granulate; frons weakly granulate or smooth; face weakly granulate or smooth; temple in dorsal view broad but not bulging behind eye, width equal to 1/2 eye width; malar space greater than 1/4 eye height; ocell-ocular distance at least 2.5 times diameter of lateral ocellus; 20–24 flagellomeres. Mesosoma: mesoscutal lobes granulate; notauli scrobiculate, meeting at scutellum in triangular rugose area; scutellum granulate; prescutellar furrow with 3 cross carinae; mesopleuron granulate; precoxal sulcus weakly scrobiculate, shorter than mesopleuron; venter granulate; propodeum with basal median areas margined, granulate, basal median carina present, areola not distinctly margined, areolar area rugose, lateral areas rugose posteriorly, granulate anteriorly. Wings: fore wing vein r shorter than vein 3RSa, vein 1cu-a beyond vein 1M; hind wing vein SC+R present, vein M+CU about equal to vein 1M. Metasoma: first tergum longitudinally costate, length greater than apical width; second tergum longitudinally costate; anterior transverse groove present, straight; posterior transverse groove present; third tergum costate basally, smooth apically; terga 4–7 smooth; ovipositor as long as metasomal tergum 1.

#### Holotype female.

Top label (white, printed) - Costa Rica, Cart[h]ago Pr. [;] La Cangreja, 1950m [;] 1991:x, P Hanson; second label (red, partially printed and hand written) - HOLOTYPE [;] Heterospilus [;] cangrejaensis [;] P. Marsh. Deposited in ESUW.

#### Paratypes.

11 ♀♀, same data as holotype with additional dates of xi.1991, vii.1991, ix–xii.1992 (ESUW). 1 ♀, COSTA RICA: Limon [;] 16km W. Guapiles [;] 400m, May 1989 [;] Coll. P. Hanson (ESUW). 1 ♀, top label - COSTA RICA, Heredia [;] Est. Biol. La Selva, 50- [;] 150m, 10°26'N, 84°01'W [;] Mar 1993, INBio-OET; second label - 02 Marzo 1993 [;] Bosque Primario [;] M/04/019; third label - INBio bar code (ESUW).

#### Comments.

The lighter colored mesoscutum is distinctive for this species.

#### Etymology.

The specific name is from the locality, La Cangreja in Cartago Province, where most of the type series was collected.

**Figure 144.**
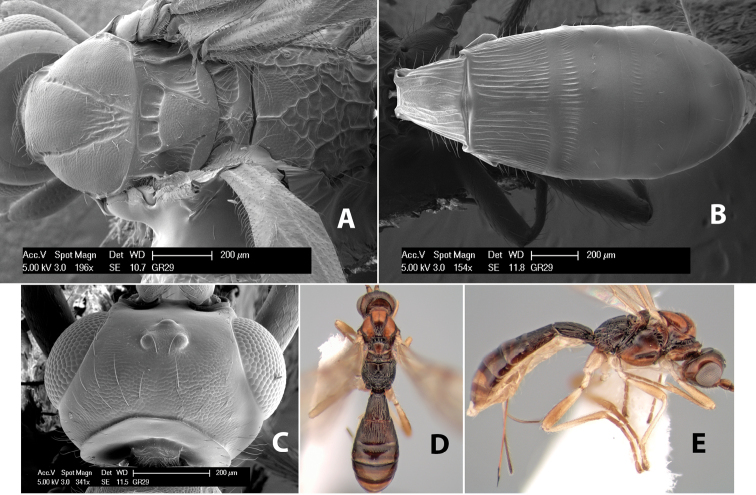
*Heterospilus cangrejaensis* Marsh, sp. n.: **A–C** paratype **D–E** holotype.

### Heterospilus caritus

Marsh sp. n.

http://zoobank.org/A8277E6D-4C43-46EC-89F7-7717C9152BE0

http://species-id.net/wiki/Heterospilus_caritus

Figure 145

#### Female.

Body size: 2.0–2.5 mm. Color: body dark brown, apical metasomal terga yellow; scape yellow with lateral longitudinal brown stripe, flagellum brown with apical white annulus, apical 3–5 flagellomeres brown; wing veins including stigma brown, legs yellow. Head: vertex granulate; frons granulate; face granulate; temple in dorsal view narrow, sloping behind eye, width less than 1/2 eye width; malar space slightly greater than 1/4 eye height; ocell-ocular distance slightly greater than 2.5 times diameter of lateral ocellus; 19–21 flagellomeres. Mesosoma: mesoscutal lobes granulate; notauli weakly scrobiculate or smooth anteriorly, absent or weak posteriorly; scutellum granulate; prescutellar furrow with 5 cross carinae; mesopleuron granulate; precoxal sulcus smooth, shorter than mesopleuron; venter granulate; propodeum with basal median areas margined, granulate, basal median carina absent, areola usually indistinct, rarely weakly indicated, areolar area rugose, lateral areas rugose apically, small granulate area basally. Wings: fore wing vein r shorter than vein 3RSa, vein 1cu-a beyond vein 1M; hind wing vein SC+R absent, vein M+CU shorter than vein 1M. Metasoma: first tergum longitudinally costate-granulate, often granulate medially, length equal to or longer than apical width; second tergum longitudinally costate-granulate; anterior and posterior transverse grooves present and indicated by row of distinct pits; third tergum granulate except for costate transverse groove; terga 4–7 granulate; ovipositor as long as or slightly shorter than metasomal tergum 1.

#### Holotype female.

Top label (white, printed) - Costa Rica: Puntarenas, ACO [;] Golfito, Est. Agujas, 250–350m [;] 15.viii–15.ix.1999, J. Azofeifa [;] L.S. 276750-526550 #53264 [;] Malaise trap; second label (red, partially printed and hand written) - HOLOTYPE [;] Heterospilus [;] caritus [;] P. Marsh. Deposited in ESUW.

#### Paratypes.

1 ♀, Costa Rica: Puntarenas, ACO [;] Golfito, R.F. Golfo Dulce [;] Est. Agujas, 250–350m [;] 4–22.v.1999, J. Azofeifa [;] L.S.276750-526550 #52779 [;] Red de Golpe (ESUW). 1 ♀, Costa Rica: Puntarenas, ACO [;] Golfito, R.F. Golfo Dulce [;] Est. Agujas, Cerro Rincon [;] 600–745m, Malaise trap [;] 15.viii–15.ix.1999, J. Azofeifa [;] L.S.275500-521950 #53268 (ESUW). 1 ♀, Costa Rica: Puntarenas [;] ACO, Golfito, RF Golfo Dulce [;] Est. Agujas, 250–300m [;] 2–22.x.1999, J. Azofeifa [;] L.S. 276750-526550 #53490 [;] Amarilla (ESUW). 1 ♀, Costa Rica: Puntarenas [;] R.F.Golfo Dulce, 3km [;] SW Rincon, 10m [;] Malaise-primary forest [;] viii.1991, P. Hanson (ESUW). 1 ♀, Costa Rica: Puntarenas [;] Res. Forestal Golfo Dulce [;] 3km. SW Rincon, 10m [;] xii.1992, P. Hanson [;] Malaise, primary forest (ESUW). 5 ♀♀, Costa Rica: Puntarenas [;] R.F. Golfo Dulce, [;] 3km.SW. Rincon, 10m [;] ii.1992, vi.1991 and xii.1992, Paul Hanson (ESUW). 1 ♀, COSTA RICA, Puntar [;] Golfo Dulce, 10km W [;] Piedras Blancas, 100m [;] VI-VII 1989, Hanson (ESUW). 1 ♀, COSTA RICA: Puntar [;] Golfo Dulce 3km SW [;] Rincon [;] 10m, xii 1989-iii 1990 [;] Col. Paul Hanson (ESUW). 1 ♀, Costa Rica: Puntarenas [;] Pen. Osa, Cerro Rincon [;] 200 meters S. del hito [;] 745m el., virgin forest [;] i.1991, Hanson & Quiros [;] ex. Malaise trap (ESUW). 1 ♀, Costa Rica: Puntarenas [;] R.F. Golfo Dulce, 24km. W [;] Piedras Blancas, 200m [;] III.1993, P. Hanson (ESUW). 1 ♀, Costa Rica: Puntar. [;] P.N. Corcovado [;] Est. Sirena, 50m [;] x-xii 1990 (ESUW). 2 ♀♀, Costa Rica: Limon, ACLAC [;] Central, R.B. Hitoy Cerere [;] Send. Espavel, 560m [;] 19.v–19.vi.1998, E. Rojas [;] L.S. 400702-570120 #52200 [;] Malaise trap (ESUW). 1 ♀, COSTA RICA: Limon [;] 4km NE Bribri [;] 50m, IX-XI 1989 [;] col. Paul Hanson (ESUW). 1 ♀, COSTA RICA: [;] Limon [;] 7km SW Bribri [;] 50m, xi 1989 [;] Col. Paul Hanson (ESUW). 1 ♀, Costa Rica: Guanacaste [;] Est. Cacao, 1000–1150m [;] ix.1996, I. Villegas, Malaise [;] L.N. 323150-375500 #47559 (ESUW). 1 ♀, COSTA RICA: Heredia, Est. [;] Biol. La Selva OTS [;] M.03.389, 30.VI.1995 (ESUW). 1 ♀, COSTA RICA, Alajuela [;] Jabillos, 100m [;] 24/III/1989 [;] col. Paul Hanson (ESUW).

#### Comments.

The weak or absent notauli posteriorly, the short ovipositor and the granulate metasomal terga 4–7 are distinctive for this species.

#### Etymology.

The specific name is from the Latin *caritus*, meaning lacking or devoid of, in reference to the absent notauli posteriorly.

**Figure 145.**
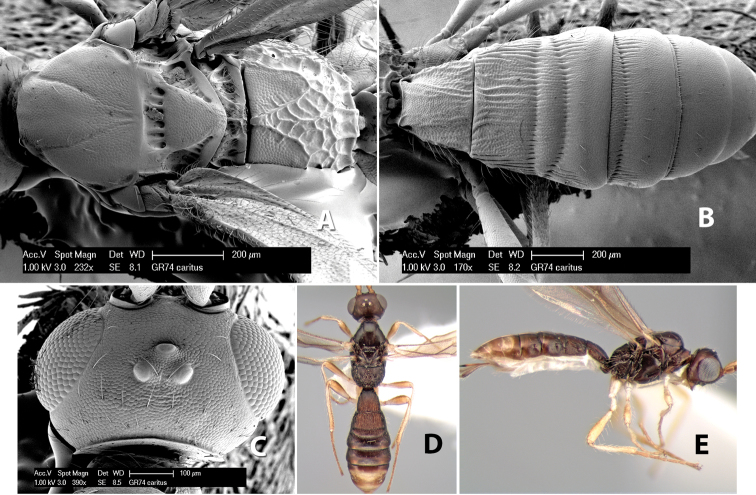
*Heterospilus caritus* Marsh, sp. n.: **A–C, E** paratype **D** holotype.

### Heterospilus carolinae

Marsh sp. n.

http://zoobank.org/587FA10C-908C-4968-924C-986E14FED65C

http://species-id.net/wiki/Heterospilus_carolinae

Figure 146

#### Female.

Body size: 3.5–4.0 mm. Color: body entirely dark brown; scape yellow with lateral longitudinal brown stripe, flagellum brown with apical white annulus, apical 5–7 flagellomeres brown; wings including stigma brown; legs yellow. Head: vertex granulate, often with transverse rugae behind ocelli; frons granulate; face granulate-rugose; temple in dorsal view narrow, sloping behind eye, width equal to 1/2 eye width; malar space 1/4 eye height; ocell-ocular distance about twice diameter of lateral ocellus; 24–28 flagellomeres. Mesosoma: mesoscutal lobes granulate; notauli weakly scrobiculate, meeting at scutellum in triangular weakly costate-rugose area; scutellum granulate; prescutellar furrow with 5 cross carinae; mesopleuron granulate; precoxal sulcus smooth, shorter than mesopleuron; venter granulate; propodeum with basal median areas margined, granulate, basal median carina absent, areola not margined, areolar area areolate-rugose, lateral areas entirely rugose. Wings: fore wing vein r shorter than vein 3RSa, vein 1cu-a beyond vein 1M; hind wing vein SC+R present, vein M+CU shorter than vein 1M. Metasoma: first tergum longitudinally costate, length greater than apical width; second tergum longitudinally costate; anterior transverse groove present, straight, posterior transverse groove present, both transverse grooves costate with row of deep pits; third tergum costate basally, weakly granular or smooth apically; terga 4–7 weakly granulate or smooth; ovipositor equal to length of metasomal terga 1 and 2 combined.

#### Holotype female.

Top label (white, printed) - Costa Rica: Puntarenas [;] R. F. Golfo Dulce, 24km. [;] W. Piedras Blancas, 200m [;] I.1993. P. Hanson; second label (red, partially printed and hand written) - HOLOTYPE [;] Heterospilus [;] carolinae [;] P. Marsh. Deposited in ESUW.

#### Paratypes.

1 ♀, same data as holotype with additional date of Feb. 1992 (ESUW). 1 ♀, Costa Rica: Guanacaste [;] Est. Pitilia, 700m [;] 9km. S de Santa Cecilia [;] viii-ix.1996, P. Rios & [;] C. Moraga, Malaise [;] L.N. 329950-380450 #47563 (ESUW). 3 ♀♀, COSTA RICA: [;] Heredia, Chilamate [;] 75m, xii 89-iii 1990, xi 1989 and IX-X 1989 [;] Hanson & Godoy (ESUW). 1 ♀, COSTA RICA: [;] Heredia, Chilamate [;] 75m, May 1989 [;] Coll. P. Hanson (ESUW). 1 ♀, top label - COSTA RICA, Heredia [;] Est. Biol. La Selva, 50- [;] 150m, 10°26'N, 84°01'W [;] Mar 1993, INBio-OET; second label - 02 Marzo 1993 [;] M/03/018 [;] Bosque primario; third label - INBio bar code (ESUW). 1 ♀, Costa Rica: Heredia [;] 3km. S. Puerto Viejo, [;] OTS, La Selva, 100m [;] xi.1992 P. Hanson (ESUW). 1 ♀, top label - COSTA RICA, Heredia: [;] Est. Biol. La Selva, 50- [;] 150m, 10°26'N, 84°01'W [;] Sep 1998, INBio-OET; second label - 03 Setiembre 1998 [;] Borde Suampo [;] M/18/716 (INBC). 2 ♀♀, COSTA RICA: Puntarenas [;] RF Golfo Dulce el 200m [;] 24km W Piedras Blancas [;] p. Hanson xi.1992 and xii.1992 (TAMU).

#### Comments.

This species is similar to *Heterospilus zapotec* but is distinguished by the entirely brown metasomal terga.

#### Etymology.

Named for Carolina Godoy who, along with her husband, Paul Hanson, has collected many braconids, and in appreciation for her and Paul’s hospitality during my visits to Costa Rica.

**Figure 146.**
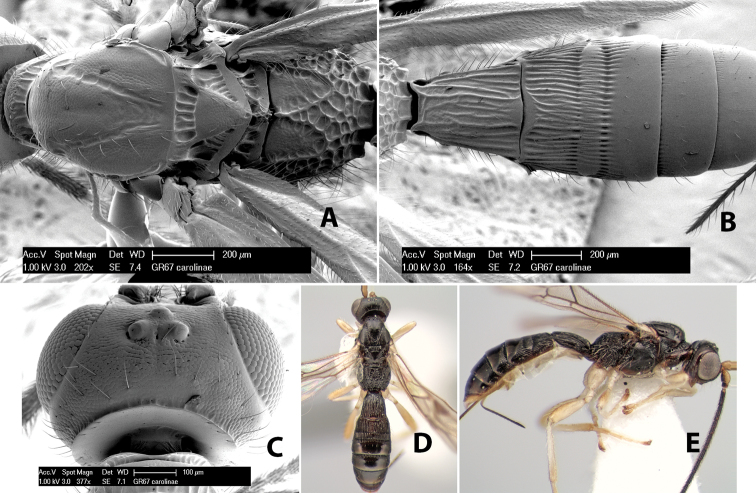
*Heterospilus carolinae* Marsh, sp. n.: **A–C** paratype **D–E** holotype.

### Heterospilus chilamatensis

Marsh sp. n.

http://zoobank.org/01E0E081-9F1E-4E43-8B31-B617B20780A3

http://species-id.net/wiki/Heterospilus_chilamatensis

Figure 147

#### Female.

Body size: 2.5–3.0 mm. Color: body dark brown, apical terga usually yellow; scape brown without lateral brown stripe; flagellum brown with white apical annulus, apical 3–5 flagellomeres brown; wing veins including stigma brown; legs yellow, tarsi usually brown. Head: vertex granulate; frons granulate; face granulate; temple in dorsal view narrow, sloping behind eye, width less than 1/2 eye width; malar space greater than 1/4 eye height; ocell-ocular distance slightly greater than twice diameter of lateral ocellus; 24–26 flagellomeres. Mesosoma: mesoscutal lobes granulate; notauli weakly scrobiculate, meeting at scutellum in triangular costate area; scutellum smooth; prescutellar furrow with 5 cross carinae; mesopleuron granulate; precoxal sulcus smooth, shorter than mesopleuron; venter granulate; propodeum with basal median areas distinctly margined, rarely indistinctly margined, sculpture granulate, basal median carina absent, areola not distinct, areolar area areolate-rugose, lateral areas entirely rugose, propodeum with small but distinct tubercle above hind coxa at base of petiole. Wings: fore wing vein r shorter than vein 3RSa, vein 1cu-a slightly beyond vein 1M, rarely interstitial; hind wing vein SC+R present, vein M+CU shorter than vein 1M. Metasoma: first tergum longitudinally costate, length equal to apical width; second tergum longitudinally costate; anterior transverse groove present, straight; posterior transverse groove present; third tergum costate at base, smooth at apex; terga 4–7 smooth; ovipositor half as long as metasoma.

#### Holotype female.

Top label (white, partially printed and hand written) - COSTA RICA, Heredia [;] Chilamate, 75m [;] 25.iii.1989 [;] Hanson & Godoy; second label (red, partially printed and hand written) - HOLOTYPE [;] Heterospilus [;] chilamatensis [;] P. Marsh. Deposited in ESUW.

#### Paratypes.

1 ♀, COSTA RICA-Heredia Prov. [;] La Selva Biological Station [;] 10°26'N, 84°01'W, 100m [;] Malaise trap 01, #332 [;] 1,2.i.1994 [;] Project ALAS(M.01.332) (ESUW). 2 ♀♀, COSTA RICA: Limon [;] 4km NE Bribri [;] 50m, IX-XI 1989 [;] col. Paul Hanson (ESUW). 1 ♀, S.RosaPark, Guan. [;] C. Rica 7 Dec., 76 [;] D. H. Janzen [;] Riparian (AEIC).

#### Comments.

The presence of hind wing vein SC+R, the smooth metasomal terga 4–6 and the dark brown scape are distinctive for this species.

#### Etymology.

Named for the locality of the holotype, Chilamate in Heredia Province.

**Figure 147.**
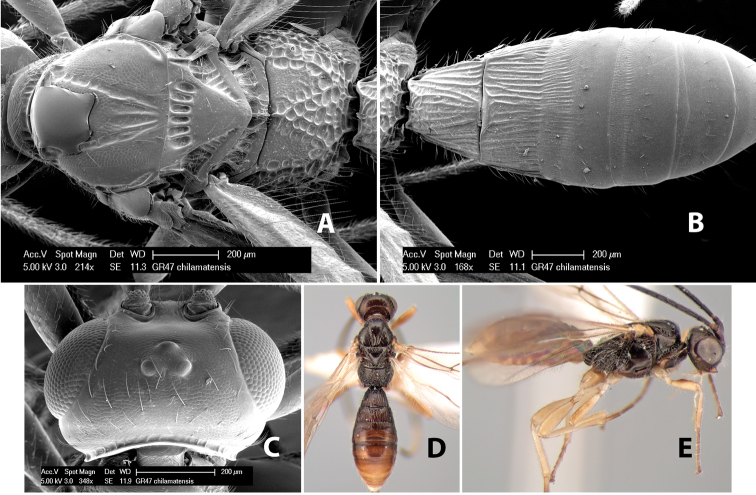
*Heterospilus chilamatensis* Marsh, sp. n.: **A–D** paratype **E** holotype.

### Heterospilus chocho

Marsh sp. n.

http://zoobank.org/980A519E-9359-462F-A4FC-52FA72263C5C

http://species-id.net/wiki/Heterospilus_chocho

Figure 148

#### Female.

Body size: 2.5–3.0 mm. Color: body dark brown, apical metasomal terga yellow; scape yellow with lateral longitudinal brown stripe, flagellum brown, apical 3–6 flagellomeres white except apical one darker; wing veins brown, stigma bicolored brown with yellow at apex and base; legs yellow. Head: vertex granulate; frons weakly granulate or smooth; face weakly granulate or smooth; temple in dorsal view narrow, sloping behind eye, width slightly less than 1/2 eye width; malar space greater than 1/4 eye height; ocell-ocular distance at least 2.5 times diameter of lateral ocellus; 22–24 flagellomeres. Mesosoma: mesoscutal lobes granulate; notauli scrobiculate, meeting at scutellum in triangular costate area; scutellum granulate; prescutellar furrow with 3 cross carinae, rarely only median carina distinct; mesopleuron granulate; precoxal sulcus weakly scrobiculate, shorter than mesopleuron; venter granulate; propodeum with basal median areas margined, granulate, basal median carina present, areola usually weakly margined, areolar area rugose, lateral areas rugose posteriorly, granulate anteriorly, propodeum with weak but distinct tubercles above hind coxae. Wings: fore wing vein r shorter than vein 3RSa, vein 1cu-a beyond vein 1M; hind wing vein SC+R present, vein M+CU slightly shorter than vein 1M. Metasoma: first tergum longitudinally costate, length slightly greater than apical width; second tergum longitudinally costate; anterior transverse groove present, straight; posterior transverse groove present; third tergum costate basally, smooth apically; terga 4–7 smooth; ovipositor about 3/4 length of metasoma.

#### Holotype female.

Top label (white, printed) - COSTA RICA: Limon [;] 16km West Guapiles [;] 400m, April 1989 [;] P. Hanson; second label (red, partially printed and hand written) - HOLOTYPE [;] Heterospilus [;] chocho [;] P. Marsh. Deposited in ESUW.

#### Paratypes.

1 ♀, same data as holotype with date of i–iv.1991 (ESUW). 1 ♀, Costa Rica: Alajuela, San Carlos [;] R.F.Arenal, Sector Cerro Chaio [;] 1100m, Malaise trap [;] 25.ix–22.x.1999, G. Carballo [;] L.N.269500-460900 #53935 (ESUW). 1 ♀, Costa Rica: Puntarenas, ACO [;] Golfito, R.F. Golfo Dulce [;] Est. Agujas, 250–350m [;] 4–22.v.1999, J. Azofeifa [;] L.S.276750-526550 #52779 [;] Red de Golpe (ESUW). 1 ♀, Costa Rica: Cartago [;] Braulio Carillo N.P. [;] 600m, 25.iii.1990 [;] J. S. Noyes, coll. (ESUW). 1 ♀, COSTA RICA, Guanac. [;] Estac. Pitilla, 9Km S [;] Santa Cecilia, 700m [;] VI/1989, I. Gauld (MICR).

#### Comments.

This species is very similar to *Heterospilus guapilensis* bur differs in the longer ovipositor, the bicolored stigma and the usually distinctly margined areola on the propodeum.

#### Etymology.

Named for the Chocho, an indigenous people of Mexico.

**Figure 148.**
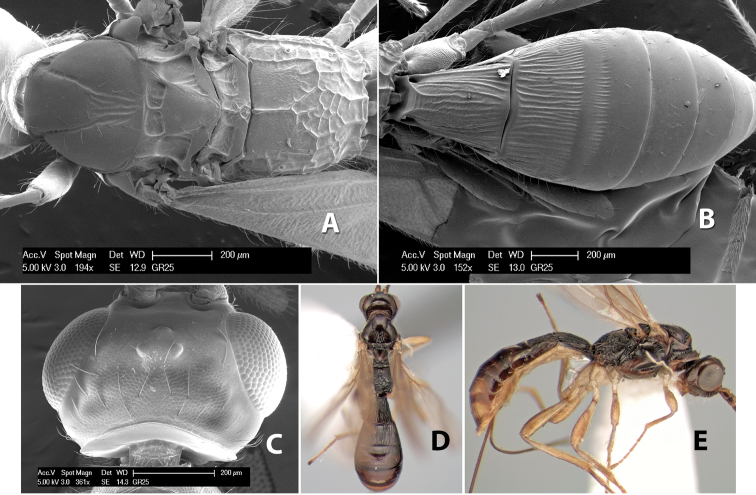
*Heterospilus chocho* Marsh, sp. n.: **A–C** paratype **D–E** holotype.

### Heterospilus chorotegus

Marsh sp. n.

http://zoobank.org/5A24836C-5837-4EFB-9712-5A4082DF090A

http://species-id.net/wiki/Heterospilus_chorotegus

Figure 149

#### Female.

Body size: 2.5–3.0 mm. Color: body entirely dark brown, apical metasomal terga rarely slightly lighter; scape brown, flagellum brown with white annulus near apex, apical 3–5 flagellomeres brown; wing veins including stigma brown; legs yellow. Head: vertex granulate, occasionally smooth behind ocelli; frons granulate, rarely partially smooth; face granulate; temple in dorsal view broad but sloping behind eye; malar space equal to 1/4 eye height, width equal to 1/2 eye width; ocell-ocular distance slightly greater than 2.5 times diameter of lateral ocellus; 22–26 flagellomeres. Mesosoma: mesoscutal lobes granulate; notauli scrobiculate, meeting posteriorly in triangular costate area; scutellum granulate, occasionally smooth; prescutellar furrow with 3 cross carinae; mesopleuron granulate; precoxal sulcus smooth, shorter than mesopleuron; venter smooth; propodeum with basal median areas weakly margined, granulate, basal median carina absent, areola not margined, areolar area areolate-rugose, lateral areas entirely rugose, propodeum with small but distinct tubercle just above hind coxa. Wings: fore wing vein r shorter than vein 3RSa, vein 1cu-a beyond vein 1M; hind wing vein SC+R present, vein M+CU shorter than vein 1M. Metasoma: first tergum longitudinally costate, sometimes rugose medially, apical width equal to length; second tergum longitudinally costate; anterior transverse groove present, straight; posterior transverse groove present; third tergum costate anteriorly, granulate posteriorly; terga 4–7 granulate, tergum 4 occasionally costate basally; ovipositor equal in length to metasomal terga 1 and 2 combined.

#### Holotype female.

Top label (white, partially printed and hand written) - Costa Rica: Guanacaste [;] Santa Rosa Natl. Park [;] 300m, ex. Malaise trap [;] Site #: H-1-O [;] Dates: 2o.vii.86–10.i.1987 [;] I.D. Gauld & D. Janzen; second label (white, printed) - [H] open regenerating [;] woodland 10 years old [;] [O] in clearing, fully [;] isolated part of day; third label (red, partially printed and hand written) - HOLOTYPE [;] Heterospilus [;] chorotegus [;] P. Marsh. Deposited on ESUW.

#### Paratypes.

7 ♀♀, same data as holotype except: top label, additional site numbers H-3-O, H-2-C and H-4-C, and additional dates 21.ii–14.iii.1987, 31.i–21.ii.1987, 6–27.ix.1986, 4–24.v.1986 and 16.xi–7.xii.1985; second label, additional third and fourth lines [C] more or less fully [;] shaded as possible (ESUW). 1 ♀, Costa Rica: Guanacaste, ACT [;] Bagaces, P.N. Palo Verde [;] Sec. P. Verde, 200 NE Est. Extremo E de Campo de [;] Aterrizaje, 0–50m, Malaise [;] 8.xi–8.xii.1999, I. Jimenez [;] L.N.260952-385020 #54241 (ESUW). 1 ♀, top label - Costa Rica: Guanacaste [;] Santa Rosa National Pk. [;] 300m, Malaise, Ian Gauld [;] 31.i–21.ii 1987; second label - Open regenerating [;] woodland less than [;] 19 yrs. old Sun; third label - H-1-O [;] 31.i–21.ii.87 (ESUW). 2 ♀♀, Costa Rica: San Jose [;] San Antonio de Escazu [;] 1300m, vi-vii and iii-iv.1998 [;] W. Eberhard & P. Hanson (ESUW). 1 ♀, Costa Rica, Puntarenas [;] Pen. Osa, Puerto Jimenez [;] 10m, VI-1993, P. Hanson (ESUW). 1 ♀, Costa Rica: Puntarenas [;] Pen. Osa, Puerto Jimenez [;] 10m, January 1991, full sun, [;] grassy & weedy site [;] P. Hanson, ex. Malaise (ESUW). 1 ♀, Costa Rica: Puntarenas [;] Pen. Osa, Puerto [;] Jimenez, 10m, December [;] 1990, P. Hanson, Malaise (ESUW). 2 ♀♀, Costa Rica: Puntarenas [;] Golfo Dulce, 24km W. [;] Piedras Blancas, 200m [;] ii and iv.1993, Paul Hanson (ESUW). 1 ♀, Costa Rica: Puntarenas [;] Golfito [;] 8–13 January 1988 [;] P. Hanson (TAMU).

#### Comments.

The small tubercle on the propodeum just above the hind coxa and the white annulus on the flagellum are distinctive for this species

#### Etymology.

Named for the Chorotega, an indigenous people of Costa Rica.

**Figure 149.**
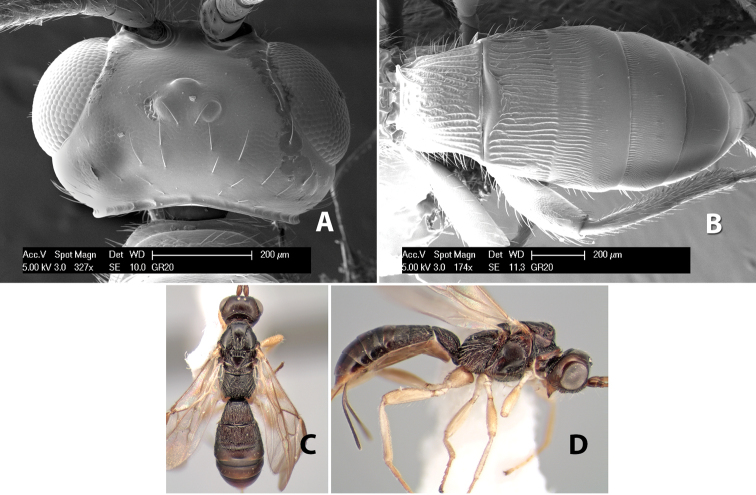
*Heterospilus chorotegus* Marsh, sp. n.: **A, B, D** paratype **C** holotype.

### Heterospilus cocopa

Marsh sp. n.

http://zoobank.org/A4604DA6-59F3-4BAA-90FE-D56FB154E4D0

http://species-id.net/wiki/Heterospilus_cocopa

Figure 150

#### Female.

Body size: 3.5 mm. Color: head bicolored, vertex and frons dark brown, face, temple and eye orbits honey yellow; scape yellow with lateral longitudinal brown stripe, flagellum honey yellow basally to dark brown apically without white annulus; mesosoma dark brown; metasomal terga dark brown, tergum 2 honey yellow medially, terga 5–7 honey yellow; wing veins brown, stigma bicolored brown with yellow at base; legs yellow. Head: vertex granulate; frons granulate; face granulate-rugose; temple in dorsal view narrow, sloping behind eye, width less than 1/2 eye width; malar space greater than 1/4 eye height; ocell-ocular distance slightly greater than 2.5 times diameter of lateral ocellus; 24 flagellomeres (broken in type series). Mesosoma: mesoscutal lobes granulate; notauli scrobiculate, meeting at scutellum in triangular rugose area; scutellum granulate; prescutellar furrow with 3–5 cross carinae; mesopleuron granulate; precoxal sulcus scrobiculate, shorter than mesopleuron; venter granulate; propodeum with basal median areas distinctly margined, granulate, basal median carina absent, areola not distinctly margined, areolar area rugose, lateral areas rugose posteriorly, granulate anteriorly. Wings: fore wing vein r about equal to vein 3RSa, vein 1cu-a beyond vein 1M; hind wing vein SC+R present, vein M+CU shorter than vein 1M. Metasoma: first tergum longitudinally costate, length equal to apical width; second tergum longitudinally costate; anterior transverse groove present, straight; posterior transverse groove present; third tergum costate basally, smooth apically; terga 4–7 smooth; ovipositor half as long as metasoma.

#### Holotype female.

Top label (white, printed) - Costa Rica: Guanacaste [;] Est. Biol. Maritza, 600m [;] i.1997, C. Zuniga, Malaise [;] L.N. 326900-373000 #47557; second label (red, partially printed and hand written) - HOLOTYPE [;] Heterospilus [;] cocopa [;] P. Marsh. Deposited in ESUW.

#### Paratypes.

1 ♀, same data as holotype (ESUW).

#### Comments.

The honey yellow basal flagellomeres and the bicolored head and metasoma are distinctive for this species.

#### Etymology.

Named for the Cocopa, an indigenous people of Baja California, Mexico.

**Figure 150.**
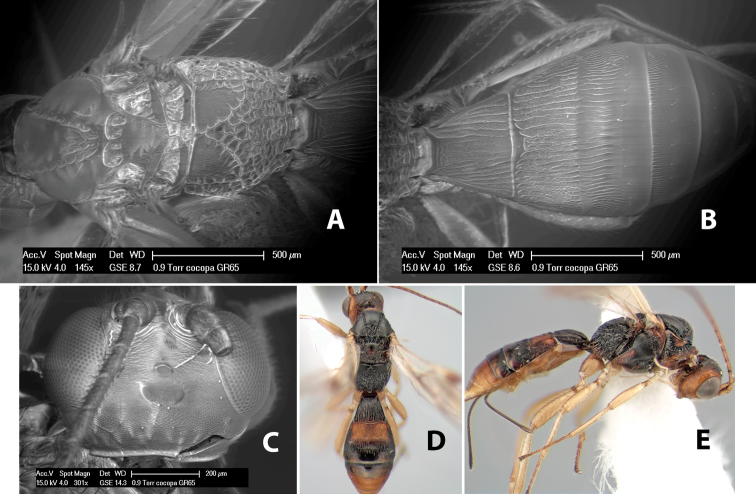
*Heterospilus cocopa* Marsh, sp. n., holotype.

### Heterospilus complanatus

Marsh sp. n.

http://zoobank.org/B869E2F6-B602-43F1-81A7-D188CA2F999C

http://species-id.net/wiki/Heterospilus_complanatus

Figure 151

#### Female.

Body size: 2.5 mm. Color: head with vertex dark brown, face lighter brown; scape brown, flagellum brown with apical white annulus, apical 3–5 flagellomeres brown; mesosoma bicolored, mesoscutum dark brown except light brown medially and along notauli, propleuron dark brown, pronotum lighter brown, mesopleuron and venter dark brown, propodeum light brown; metasomal terga brown, tergum 1 marked with dark brown at base and laterally, terga 2–4 dark brown laterally; wing veins light brown, stigma yellow; legs yellow. Head: flattened dorso-ventrally; vertex granulate; frons granulate; face granulate; temple in dorsal view broad but sloping behind eye, width equal to 1/2 eye width; malar space equal to 1/4 eye height; ocell-ocular distance greater than 2.5 times diameter of lateral ocellus; 21 flagellomeres. Mesosoma: flattened dorso-ventrally; mesoscutal lobes granulate; notauli scrobiculate, meeting posteriorly in wide rugose area; scutellum granulate; prescutellar furrow with 5 cross carinae; mesopleuron granulate; precoxal sulcus weakly scrobiculate, shorter than mesopleuron; venter granulate; propodeum with basal median areas very narrow and not distinctly margined, weakly granulate, basal median carina absent, areola not distinctly margined, areolar area areolate, lateral areas entirely rugose or areolate. Wings: fore wing vein r shorter than vein 3RSa, vein 1cu-a beyond vein 1M; hind wing vein SC+R present, vein M+CU shorter than vein 1M. Metasoma: first tergum longitudinally costate, apical width less than length; second tergum longitudinally costate; anterior transverse groove present, straight; posterior transverse groove present, curved anteriorly at sides; third tergum costate at base, smooth at apex; terga 4–7 smooth, tergum 4 costate at base; ovipositor shorter than metasomal tergum 1.

#### Holotype female.

Top label (white, printed) - Costa Rica: Guanacaste [;] Est. Biol. Maritza, 600m [;] i.1997, C. Zuniga, Malaise [;] L.N. 326900-373000 #47557; second label (red, partially printed and hand written) - HOLOTYPE [;] Heterospilus [;] complanatus [;] P. Marsh. Deposited in ESUW.

#### Paratypes.

Known only from the holotype.

#### Comments.

The flattened body is distinctive for this species.

#### Etymology.

The specific name is from the Latin *complanatus* meaning flattened in reference to the flat body.

**Figure 151.**
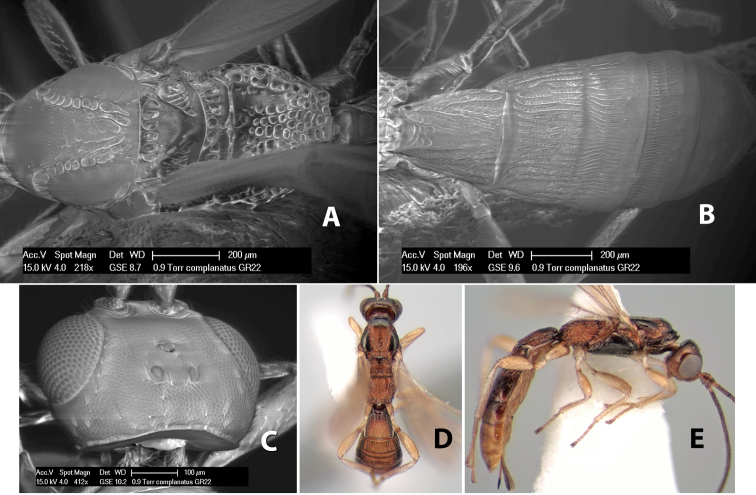
*Heterospilus complanatus* Marsh, sp. n., holotype.

### Heterospilus cora

Marsh sp. n.

http://zoobank.org/05582597-527B-4EBB-A824-9343C1646DC8

http://species-id.net/wiki/Heterospilus_cora

Figure 152

#### Female.

Body size: 2.5 mm. Color: head dark brown; scape honey yellow without lateral brown stripe; flagellum brown; mesosoma brown to dark brown; metasomal terga dark brown, tergum 1 at apex and tergum 2 medially yellow, apical terga slightly lighter; wing veins including stigma brown; legs yellow. Head: vertex granulate; frons granulate; face granulate; temple in dorsal view broad but sloping behind eye, width equal to 1/2 eye width; malar space greater than 1/4 eye height; ocell-ocular distance greater than 2.5 times diameter of lateral ocellus; 22 flagellomeres. Mesosoma: mesoscutal lobes granulate; notauli scrobiculate, meeting at scutellum in small triangular rugose area; scutellum granulate; prescutellar furrow with 3–5 cross carinae; mesopleuron granulate; precoxal sulcus smooth, shorter than mesopleuron; venter granulate; propodeum with basal median areas distinct but not distinctly margined, granulate, basal median carina absent, areola not distinctly margined, areolar area areolate, lateral areas entirely rugose. Wings: fore wing vein r shorter than vein 3RSa, vein 1cu-a beyond vein 1M; hind wing vein SC+R present, vein M+CU shorter than vein 1M. Metasoma: first tergum longitudinally costate, apical width slightly greater than length; second tergum longitudinally costate; anterior transverse groove present, straight; posterior transverse groove present; third tergum costate basally, granulate apically; terga 4–7 granulate; ovipositor slightly shorter than metasomal tergum 1.

#### Holotype female.

Top label (white, partially printed and hand written) - Costa Rica: Guanacaste [;] Santa Rosa Natl. Park [;] 300m, ex. Malaise trap [;] Site #: BH-9-O [;] Dates: 2–23.iii.1986 [;] I.D. Gauld & D. Janzen; second label (white, printed) - [SE] Bosque San Emilio [;] 50yr old deciduous forest [;] [C] more or less fully [;] shaded as possible; third label (red, partially printed and hand written) - HOLOTYPE [;] Heterospilus [;] cora [;] P. Marsh. Deposited in ESUW.

#### Paratypes.

Known only from the holotype.

#### Comments.

The short ovipositor, short metasomal tergum 1 and the brown flagellum are distinctive for this species.

#### Etymology.

Named for the Cora, an indigenous people of Jalisco and Nayarit, Mexico.

**Figure 152.**
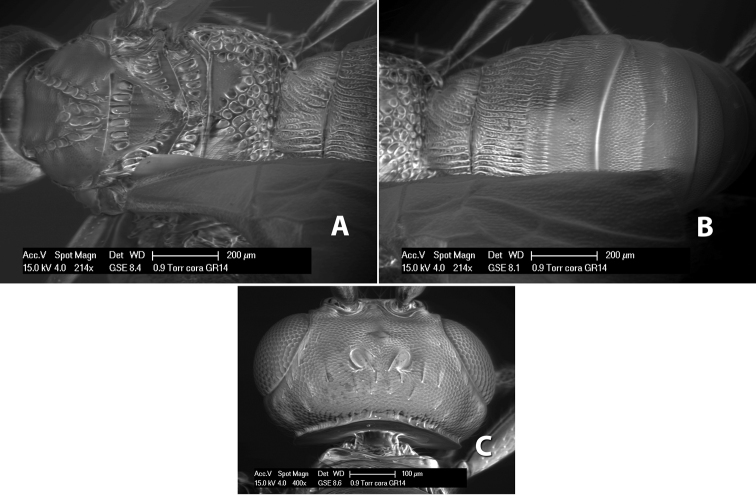
*Heterospilus cora* Marsh, sp. n., paratype.

### Heterospilus dani

Marsh sp. n.

http://zoobank.org/08B95033-D574-4B2D-A5E2-19E92FF72863

http://species-id.net/wiki/Heterospilus_dani

Figure 153

#### Female.

Body size: 2.0–2.5 mm. Color: body dark brown, metasomal tergum 1 sometimes, terga 2 and 5–7 always yellow; scape yellow, usually with lateral longitudinal brown stripe, occasionally this stripe weak or absent; flagellum brown with apical white annulus, apical 3–5 flagellomeres brown; wing veins including stigma brown; legs yellow. Head: vertex weakly granulate; frons weakly granulate; face weakly granulate or partially smooth; temple in dorsal view narrow, sloping behind eye, width less than 1/2 eye width; malar space slightly greater than 1/4 eye height; ocell-ocular distance greater than 2.5 times diameter of lateral ocellus; 17–21 flagellomeres. Mesosoma: mesoscutal lobes granulate; notauli scrobiculate, meeting at scutellum in very small weakly costate area; scutellum granulate; prescutellar furrow with 3–5 cross carinae; mesopleuron granulate; precoxal sulcus smooth, shorter than mesopleuron; venter granulate; propodeum with basal median areas margined, granulate, basal median carina absent, areola not margined, areolar area rugose, lateral areas entirely rugose. Wings: fore wing vein r shorter than vein 3RSa, vein 1cu-a interstitial with vein 1M; hind wing vein SC+R absent, vein M+CU shorter than vein 1M. Metasoma: first tergum longitudinally costate-granulate, length equal to apical width; second tergum longitudinally costate-granulate; anterior transverse groove present, straight; posterior transverse groove present; third tergum costate basally, granulate apically; terga 4–7 granulate; ovipositor shorter than metasomal tergum 1.

#### Holotype female.

Top label (white, printed) - Costa Rica: Puntarenas [;] R.F. Golfo Dulce, [;] 3km. SW. Rincon, 10m [;] ii.1992, Paul Hanson; second label (red, partially printed and hand written) - HOLOTYPE [;] Heterospilus [;] dani [;] P. Marsh. Deposited in ESUW.

#### Paratypes.

10 ♀♀, same data as holotype with additional dates of xii.1992 and vi.1991 (ESUW). 1 ♀, COSTA RICA: [;] Puntar [;] Golfo Dulce, 3km [;] SW, Rincon, 10m [;] VI-VIII 1989, Hanson (ESUW). 1 ♀, Costa Rica: Puntarenas [;] R.F.Golfo Dulce, 3km [;] SW Rincon, 10m [;] Malaise-primary forest [;] viii.1991, P. Hanson (ESUW). 1 ♀, Costa Rica: Puntarenas [;] San Vito, Estac. Biol. [;] Las Alturas, 1750m [;] IX-XI.1992, P. Hanson (ESUW). 1 ♀, Costa Rica: Puntarenas [;] Res. Forestal Golfo Dulce [;] 3km. SW Rincon, 10m [;] xii.1992, P. Hanson [;] Malaise, primary forest (ESUW). 1 ♀, COSTA RICA: Puntarenas [;] Rd. to Rincon, 24km W. [;] of Pan-Amer. Hwy. 200m [;] II-III 1989, Hanson & Gauld (ESUW). 2 ♀♀, COSTA RICA: [;] Puntar, Golfo Dulce [;] 24km W Piedras Blancas [;] 200m, vi-viii 1989 [;] Hanson (ESUW). 2 ♀♀, Costa Rica: Puntarenas [;] R.F. Golfo Dulce, 24km. W [;] Piedras Blancas, 200m [;] III.1993, P. Hanson (ESUW). 2 ♀♀, Costa Rica: Puntarenas [;] R.F. Golfo Dulce, 3km. [;] S.W. Rincon, 10m [;] I.1992, P. Hanson (ESUW). 3 ♀♀, top label - Costa Rica: Guanacaste [;] Santa Rosa Natl. Park [;] 300m, ex. Malaise trap [;] Site: H-2-C and H-4-C [;] Dates: 27.xi–18.x.1986, 24.v–14.vi.1986 and 8–29.x.1986; second label - [H] open regenerating [;] woodland <10 years old [;] [C] more or less fully [;] shaded as possible (ESUW). 8 ♀♀, top label - Costa Rica: Guanacaste [;] Santa Rosa Natl. Park [;] 300m, ex. Malaise trap [;] Site: H-1-O, H-3-O and blank [;] Dates; 26.x–16.xi.1985, 29.xi–20.xii.1986, 10–31.i.1987, 27.ix–16.x.1986, 18.x–8.xi.1986, 14.viii–6.x.1986 and 16.xi–7.xii.1985; second label - [H] open regenerating [;] woodland <10 years old [;] [O] in clearing, fully [;] isolated part of day (ESUW). 2 ♀♀, top label - Costa Rica: Guanacaste [;] Santa Rosa Natl. Park [;] 300m, ex. Malaise trap [;] Site: SE-8-C and blank [;] Dates; 8–29.xi.1986 and 20.xii–10.i.1986/7; second label - [SE] Bosque San Emilio [;] 50yr old deciduous forest [;] [C] more or less fully [;] shaded as possible (ESUW). 1 ♀, top label - Costa Rica: Guanacaste [;] Santa Rosa Natl. Park [;] 300m, ex. Malaise trap [;] Site: SE-5-O [;] Dates; 6–23.ix.1986; second label - [SE] Bosque San Emilio [;] 50yr old deciduous forest [;] [O] in clearing, fully [;] isolated part of day (ESUW). 1 ♀, top label - Costa Rica: Guanacaste [;] Santa Rosa Natl. Park [;] 300m, ex. Malaise trap [;] Site: blank [;] Dates; 24.v–14.vi.1986; second label - [BH] Bosque Humedo [;] mature evergreen dry forest [;] [O] in clearing, fully [;] isolated part of day (ESUW). 1 ♀, top label - Costa Rica: BH-10-C [;] Guanacaste Province [;] Santa Rosa Natl. Pk. [;] 300m, (dry season) [;] 10–31 January 1987; second label - Bosque Humedo, mature [;] dry forest with high [;] proportion evergreen [;] species, fully shaded [;] Townes style Malaise [;] Ian Gauld coll. (ESUW). 1 ♀, Costa Rica: Guanacaste [;] Santa Rosa National Pk. [;] 300m, Malaise H-3-O [;] regenerating woodland [;] <10 yr. old, Ian Gauld [;] 5–26.vii.2986, clearing (ESUW). 1 ♀ Costa Rica: Guanacaste [;] Santa Rosa Natl. Park [;] regenerating woodland [;] >10yr. old, 300 metres [;] 6–27.ix.1986, I.D.Gauld [;] ex. Townes Malaise H3-O [;] direct sun daily, wet (ESUW). 1 ♀, top label - Costa Rica: Guanacaste [;] Santa Rosa National Pk. [;] 300m, Malaise, Ian Gauld [;] 31.i–21.ii.1987; second label - Bosque Humedo [;] Mature dry forest [;] high proportion [;] Evergreen species [;] Full Shade; third label - BH-10-C [;] 31.i–21.ii.87 (ESUW). 1 ♀, top label - Costa Rica: Guanacaste [;] W. side Volcan Orosi [;] Estac. Maritza, 600m; second label - GNP Biodiversity Survey [;] 1989, Malaise trap [;] L-N-326900-373000 #6834 (ESUW). 1 ♀, COSTA RICA: Puntarenas [;] Reserva Forestal Golfo Dulce [;] 3km SW of Rincon, 10m [;] Mar-April 1992, P. Hanson [;] primary forest, Malaise trap (ESUW). 3 ♀♀, COSTA RICA: [;] San Jose [;] Ciudad Colon [;] 800m, iii-iv 1990 [;] Col. Luis Fournier (ESUW). 1 ♀, COSTA RICA: Alajuela [;] San Pedro de la [;] Tigra Cacao, 200m [;] iii-iv 1990, R. Cespedes (ESUW). 1 ♀, Costa Rica, Carthago Pr. [;] Dulce Nombre, Vivero [;] Linda Vista, 1300m [;] 1994: v-vi, P. Hanson (ESUW). 1 ♀, Costa Rica, Cartago [;] Turrialba, La Isabel [;] 650m, Café, IV-1994 [;] M. Cerda & P. Hanson (ESUW). 1 ♀, COSTA RICA: Puntarenas [;] RF Golfo Dulce el 200m [;] 24km W Piedras Blancas [;] P. Hanson ix.1992 (TAMU).

#### Comments.

The weakly sculptured area where the notauli meet and the bicolored metasomal terga are distinctive for this species.

#### Etymology.

Named for my son-in-law, Dan Holoski.

**Figure 153.**
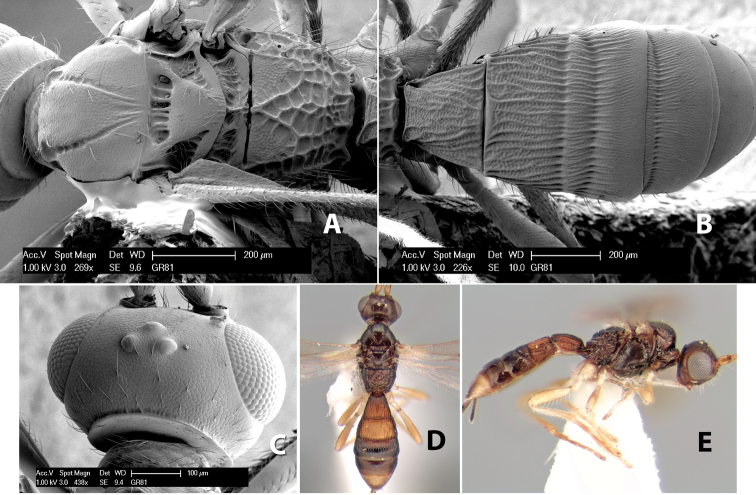
*Heterospilus dani* Marsh, sp. n.: **A–C** paratype **D–E** holotype.

### Heterospilus dianae

Marsh sp. n.

http://zoobank.org/45F03603-A019-4C07-953D-F9FEDA79AE78

http://species-id.net/wiki/Heterospilus_dianae

Figure 154

#### Female.

Body size: 2.5 mm. Color: body dark brown, apical metasomal terga lighter brown; scape brown, flagellum brown with 3–5 flagellomeres white near apex, apical most 3–5 flagellomeres brown; wing veins brown, stigma honey yellow; legs yellow. Head: vertex weakly granulate; frons weakly granulate; face granulate; temple in dorsal view broad but not bulging behind eye, width about 1/2 eye width; malar space greater than 1/4 eye height; ocell-ocular distance greater than 2.5 times diameter of lateral ocellus; 23–24 flagellomeres. Mesosoma: mesoscutal lobes granulate, often weakly so; notauli scrobiculate, meeting at scutellum in triangular costate area; scutellum granulate; prescutellar furrow with 3–5 cross carinae; mesopleuron granulate; precoxal sulcus smooth, shorter than mesopleuron; venter granulate; propodeum with basal median areas margined, granulate, basal median carina absent, areola not distinctly margined, areolar area areolate-rugose, lateral areas entirely rugose. Wings: fore wing vein r slightly shorter than vein 3RSa, vein 1cu-a slightly beyond vein 1M; hind wing vein SC+R present, vein M+CU shorter than vein 1M. Metasoma: first tergum longitudinally costate, apical width equal to length; second tergum longitudinally costate; anterior transverse groove present, straight; posterior transverse groove present; third tergum costate basally, weakly granulate apically; terga 4–7 weakly granulate; ovipositor equal to combined length of metasomal terga 1 and 2.

#### Holotype female.

Top label (partially printed and hand written) - Costa Rica: Guanacaste [;] Santa Rosa Natl. Park [;] 300m, ex. Malaise trap [;] Site #: H-1-O [;] Dates: 8–29.xi.1986 [;] I.D. Gauld & D. Janzen; second label (white, printed) - [H] open regenerating [;] woodland <10 years old [;] [O] in clearing, fully [;] isolated part of day; third label (red, partially printed and hand written) - HOLOTYPE [;] Heterospilus [;] dianae [;] P. Marsh. Deposited in ESUW.

#### Paratypes.

2 ♀♀, same data as holotype with additional date of 20.xii.86–10.i.198(7) (ESUW). 1 ♀, Costa Rica: Puntarenas [;] San Vito - Las Cruces [;] 22-IV to 5-V-1988 [;] P. Hanson (TAMU).

#### Comments.

The dark brown scape and body and honey yellow stigma are distinctive for this species.

#### Etymology.

Named for the Roman goddess Diana.

**Figure 154.**
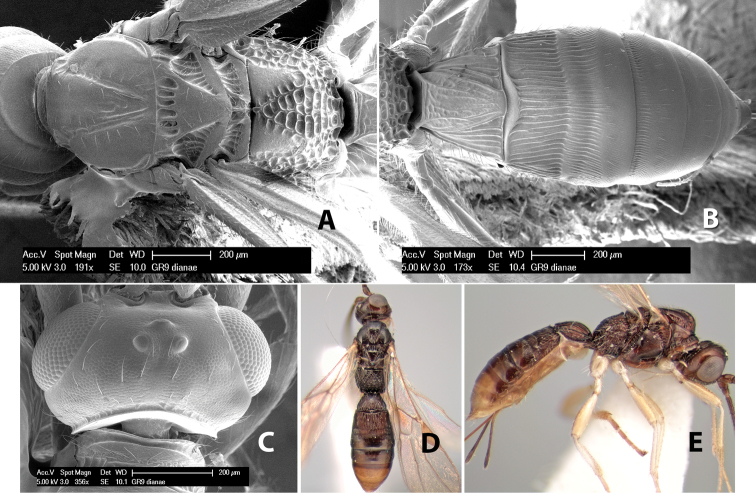
*Heterospilus dianae* Marsh, sp. n.: **A–C, E** paratype **D** holotype.

### Heterospilus ektorincon

Marsh sp. n.

http://zoobank.org/82149565-F212-48CB-A7AC-7B62D89984DA

http://species-id.net/wiki/Heterospilus_ektorincon

Figure 155

#### Female.

Body size: 3.0 mm. Color: head dark brown; scape yellow with lateral longitudinal brown stripe, flagellum brown with apical 5–6 flagellomeres below apical-most 5–8 flagellomeres white; mesosoma dark brown; metasomal tergum 1 dark brown, tergum 2 and usually 3 yellow medially, dark brown laterally, terga 4 and 5 dark brown, remainder of terga lighter brown; wing veins including stigma brown; legs yellow. Head: vertex granulate, usually with distinct transverse rugae behind ocelli; frons granulate; face granulate; temple in dorsal view narrow, sloping behind eye, width less than 1/2 eye width; malar space equal to 1/4 eye height; ocell-ocular distance 1.5–2.0 times diameter of lateral ocellus; 24–28 flagellomeres. Mesosoma: mesoscutal lobes granulate; notauli scrobiculate, meeting at scutellum in short triangular costate area; scutellum granulate; prescutellar furrow with 3–5 cross carinae; mesopleuron granulate; precoxal sulcus weakly scrobiculate, shorter than mesopleuron; venter granulate; propodeum with basal median areas indistinctly margined, granulate but margined by areolate groove, basal median carina absent, areola not distinctly margined, areolar area areolate, lateral areas entirely rugose, propodeum with distinct tubercle just above hind coxae. Wings: fore wing vein r shorter than vein 3RSa, vein 1cu-a beyond vein 1M; hind wing vein SC+R present, vein M+CU shorter than vein 1M. Metasoma: first tergum longitudinally costate, somewhat rugose medially, length equal to or slightly greater than apical width; second tergum longitudinally costate; anterior transverse groove present, straight; posterior transverse groove present; third tergum costate basally, granulate apically; terga 4–7 granulate; ovipositor equal to length of metasomal terga 1 and 2 combined.

#### Holotype female.

Top label (white, printed) - Costa Rica: Puntarenas [;] Res. Forestal Golfo Dulce [;] 3km. SW Rincon, 10m [;] iv.1993, P. Hanson [;] Malaise, primary forest; second label (red, partially printed and hand written) - HOLOTYPE [;] Heterospilus [;] ektorincon [;] P. Marsh. Deposited in ESUW.

#### Paratypes.

14 ♀♀, same data as holotype with additional dates of iii.1993, xii.1992 and ii.1993 (ESUW). 3 ♀♀, Costa Rica: Puntarenas [;] R.F. Golfo Dulce [;] 3km. SW. Rincon, 10m. [;] vi.1991 and ii.1992, Paul Hanson (ESUW). 5 ♀♀, COSTA RICA: Puntarenas [;] Rd. to Rincon, 10km W. [;] of Pan-Amer. Hwy, 100m [;] III-V 1989, Hanson & Gauld (ESUW). 1 ♀, COSTA RICA: Puntar [;] Golfo Dulce, 10km W [;] Piedras Blancas, 100m [;] VI-VIII 1989, Hanson (ESUW). 2 ♀♀, Costa Rica: Puntarenas [;] R.F. Golfo Dulce, 24km. W [;] Piedras Blancas, 200m [;] III.1993 and vi.1991, P. Hanson (ESUW). 1 ♀, COSTA RICA: Puntar. [;] Cerro Rincon, 200m [;] S. hito, 745m, ii. [;] 1991, Hanson/Godoy (ESUW). 2 ♀♀, COSTA RICA: Puntar [;] P.N. Corcovado, Est [;] Sirena, 50m [;] IV-VIII 1989 (ESUW). 1 ♀, Costa Rica: Puntar. [;] P.N. Corcovado [;] Est. Sirena, 50m [;] x-xii 1990 (ESUW). 1 ♀, COSTA RICA: Puntar [;] Golfo Dulce, 3km SW [;] Rincon [;] 10m, xii 1989-iii 1990 [;] Col. Paul Hanson (ESUW). 1 ♀, Costa Rica: Puntarenas [;] San Vito, Las Cruces [;] Wilson Botanical Gardens [;] 18–22.iii.1990, 1150m [;] J.S. Noyes (ESUW). 1 ♀, COSTA RICA: Puntarenas [;] Reserva Forestal Golfo Dulce [;] 3km SW of Rincon, 10m [;] October 1992, P. Hanson [;] primary forest, Malaise trap (ESUW). 1 ♀, Costa Rica: Puntarenas [;] Pen. Osa, 23km. N. Pto. [;] Jimenez, La Palma, 10m [;] viii-ix.1991, P. Hanson [;] Malaise, in large trees (ESUW). 1 ♀. Costa Rica: Puntarenas [;] Peninsula Osa, 10m [;] 3km SW Rincon [;] ix–1992, P. Hanson (ESUW). 1 ♀, Costa Rica: Heredia [;] 3km. S. Puerto Viejo, [;] OTS, La Selva, 100m [;] xii.1992, P. Hanson (ESUW). 1 ♀, Costa Rica: Limon [;] 30km N Cariari, 100m [;] Sector Cocori, Malaise [;] iii.1995, E. Rojas #4524 [;] L.N. 286000-567500 (ESUW). 1 ♀, COSTA RICA: Limon [;] 16km W. Guapiles [;] 400m, i-iv.1991 [;] col. Paul Hanson (ESUW). 1 ♀, COSTA RICA, Guanac. [;] Estac. Pitilla, 9Km S [;] Santa Cecilia, 700m [;] IV/1989, I Gauld (ESUW). 1 ♀, Costa Rica: Cartago [;] Braulio Carrillo N.P. [;] 400m IV.10–11.85 [;] Henri Goulet (AEIC). 1 ♀, top label - COSTA RICA, Heredia: [;] Est. Biol. La Selva, 50- [;] 150m, 10°26'N, 84°01'W [;] Sep 1995, INBio-OET; second label - 29 Setiembre 1995 [;] M/05/463 [;] Bosque primario (INBC). 1 ♀, top label - COSTA RICA, Heredia: [;] Est. Biol. La Selva, 50- [;] 150m, 10°26'N, 84°01'W [;] Mar 1996, INBio-OET; second label - 5 Marzo 1966 [;] Borde Suampo [;] M/18.703 (INBC). 1 ♀, top label - COSTA RICA, Heredia: [;] Est. Biol. La Selva, 50- [;] 150m, 10°26'N, 84°01'W [;] Feb 1998, INBio-OET; second label - 19 Febrero 1998 [;] M/18/702 [;] Borde suampo (INBC). 2 ♀♀, COSTA RICA, Puntar. [;] Golfo Dulce, 24km W. [;] PiedrasBlancas, 200m [;] XII-89-III-90 and III-VI-90 Hanson (MICR). 1 ♀, COSTA RICA, Puntarenas [;] San Vito, Jardin Bot. [;] Las Cruces, XII/1988 [;] 1200m, Col. P. Hanson (MICR). 1 ♀, COSTA RICA: [;] 11mi. from Turrialba [;] “Los Esperales”, C.A.T.I.E. [;] 5-II-1985 [;] P. Stansly (TAMU).

#### Comments.

The rugae on the vertex and the granulate face and metasomal terga 4–6 are distinctive for this species.

#### Etymology.

The specific name is from the Greek prefix *ekto-*, meaning out of or from, in reference to most of the type series being collected near the town of Rincon in Puntarenas Province.

**Figure 155.**
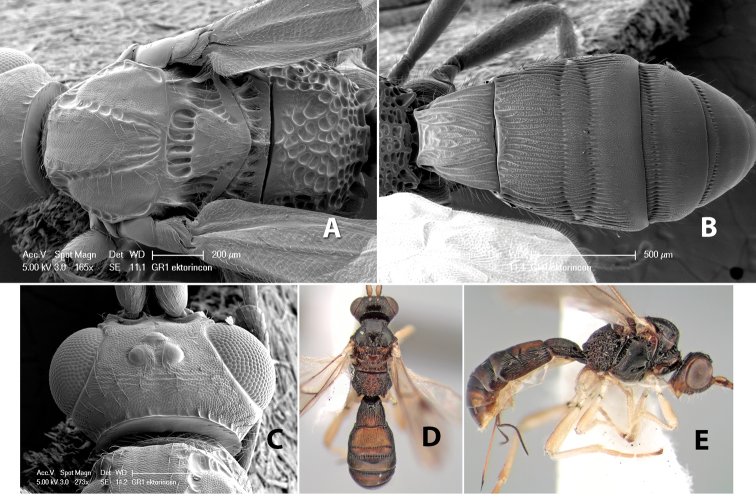
*Heterospilus ektorincon* Marsh, sp. n. **A–C** paratype **D–E** holotype.

### Heterospilus flavisoma

Marsh sp. n.

http://zoobank.org/90F27D2E-5CC8-48F9-9148-A0239C6F0E11

http://species-id.net/wiki/Heterospilus_flavisoma

Figure 156

#### Female.

Body size: 2.5 mm. Color: head dark brown; scape yellow with weak lateral longitudinal brown stripe, sometimes absent, apical 3–5 flagellomeres brown, nest 3–5 white, remainder brown; mesosoma dark brown; metasoma usually entirely yellow or honey yellow, terga 1–3 often marked with brown; wing veins brown, stigma yellow; legs yellow. Head: vertex granulate; frons granulate; face granulate; temple in dorsal view broad, width equal or greater than 1/2 eye width; malar space greater than 1/4 eye height; ocell-ocular distance about 2.5 times diameter of lateral ocellus; 23–26 flagellomeres. Mesosoma: mesoscutal lobes granulate; notauli scrobiculate, meeting at scutellum in triangular costate area; scutellum granulate; prescutellar furrow with 3–5 cross carinae; mesopleuron granulate; precoxal sulcus scrobiculate, shorter than mesopleuron; venter granulate; propodeum with basal median areas distinct but not distinctly margined, granulate, basal median carina absent, areola not distinctly margined, areolar area areolate, lateral areas entirely rugose. Wings: fore wing vein r shorter than vein 3RSa, vein 1cu-a beyond vein 1M; hind wing vein SC+R present, vein M+CU shorter than vein 1M. Metasoma: first tergum longitudinally costate, apical width equal to length; second tergum longitudinally costate; anterior transverse groove present, straight; posterior transverse groove present; third tergum costate at base, granulate at apex; terga 4–7 weakly granulate, sometimes appearing smooth; ovipositor equal to length of metasomal terga 1 and 2 combined.

#### Holotype female.

Top label (white, printed) - COSTA RICA: [;] Guanacaste Prov. [;] Cerro el Hacha [;] NW Volcan Orosi [;] 300m, 1988; second label (red, partially printed and hand written) - HOLOTYPE [;] Heterospilus [;] flavisoma [;] P. Marsh. Deposited in ESUW.

#### Paratypes.

3 ♀♀, same data as holotype (ESUW). 6 ♀♀, top label - Costa Rica: Guanacaste [;] Santa Rosa Natl. Park [;] 300m, ex. Malaise trap [;] Site # BH-9-O, H-1-O and H-3-O [;] Dates: 29.xi–20.xii.1986, 26.vii–14.viii.1986, 18.x–8.xi.1986, 28.xii.86–10.i.1987 and 10–31.i.1987 [;] I.D. Gauld & D. Janzen; second label, [BH] Bosque Humedo [;] mature evergreen dry forest [;] [O] in clearing, fully [;] isolated part of day, [H] open regenerating [;] woodland, 10 years old [;] [O] in clearing, fully [;] isolated part of day (ESUW). 1 ♀, S.RosaPark. Guan. [;] C. Rica 5 Jan 78 [;] D.H. Janzen [;] Riparian (AEIC).

#### Comments.

The yellow metasoma and yellow stigma are distinctive for this species.

#### Etymology.

The specific name is from the Latin *flavus*, meaning yellow, in reference to the yellow metasoma.

**Figure 156.**
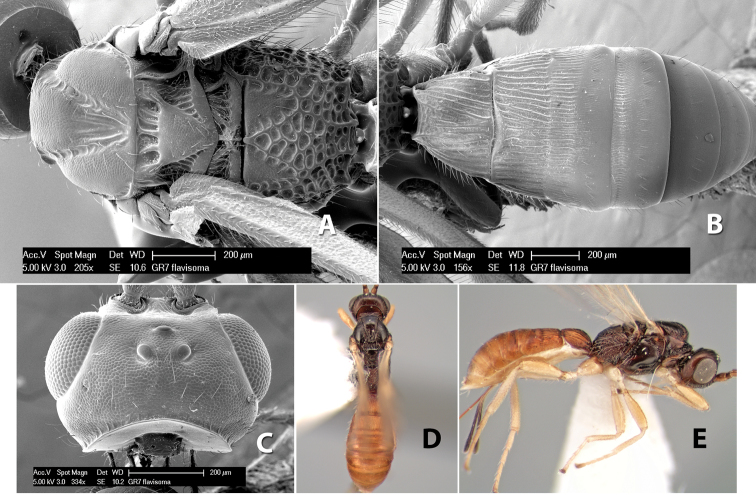
*Heterospilus flavisoma* Marsh, sp. n.: **A–C** paratype **D–E** holotype.

### Heterospilus guapilensis

Marsh sp. n.

http://zoobank.org/BFA4256F-3BFB-481F-A21B-F12072F87099

http://species-id.net/wiki/Heterospilus_guapilensis

Figure 157

#### Female.

Body size: 2.5–3.0 mm. Color: body dark brown, apical metasomal terga yellow; scape yellow with lateral longitudinal brown stripe, flagellum brown with apical 4–6 flagellomeres white, apical one darker; wing veins including stigma brown; legs yellow. Head: vertex granulate; frons weakly granulate or smooth; face granulate; temple in dorsal view narrow, sloping behind eye, width less than 1/2 eye width; malar space greater than 1/4 eye height; ocell-ocular distance about 2.5 times diameter of lateral ocellus; 22–24 flagellomeres. Mesosoma: mesoscutal lobes granulate; notauli scrobiculate, meeting at scutellum in triangular costate area; scutellum granulate; prescutellar furrow with 3 cross carinae; mesopleuron granulate; precoxal sulcus weakly scrobiculate, shorter than mesopleuron; venter granulate; propodeum with basal median areas margined, granulate, basal median carina present, areola not distinctly margined, areolar area rugose, lateral areas rugose posteriorly, granulate anteriorly, propodeum with weak but distinct tubercles above hind coxae. Wings: fore wing vein r shorter than vein 3RSa, vein 1cu-a beyond vein 1M; hind wing vein SC+R present, vein M+CU slightly shorter than vein 1M. Metasoma: first tergum longitudinally costate, length slightly greater than apical width; second tergum longitudinally costate; anterior transverse groove present, straight; posterior transverse groove present; third tergum costate basally, smooth apically; terga 4–7 smooth; ovipositor as long as metasomal tergum 1.

#### Holotype female.

Top label (white, printed) - COSTA RICA: Limon [;] 16km West Guapiles [;] 400m, April 1989 [;] P. Hanson; second label (red, partially printed and hand written) - HOLOTYPE [;] Heterospilus [;] guapilensis [;] P. Marsh. Deposited in ESUW.

#### Paratypes.

1 ♀, COSTA RICA: [;] Heredia, Chilamate [;] 75m, xii 89-iii 1990 [;] Hanson & Godoy (ESUW). 1 ♀, Costa Rica, Puntarenas [;] Pen. Osa, 5km. N. [;] Puerto Jimenez, 10m [;] I-II-1993 P. Hanson (ESUW). 1 ♀, Costa Rica: San Jose [;] Zurqui de Moravia [;] 1600m, xi.1995 [;] P. Hanson (ESUW). 1 ♀, COSTA RICA-Heredia Prov. [;] La Selva Biological Station [;] 10°26'N, 84°01'W, 100m [;] Malaise trap 04, #351 [;] 15.ii.1994 [;] Project ALAS (M.04.351) (ESUW).

#### Comments.

This species is similar to *Heterospilus chocho* but differs in the shorter ovipositor, the brown stigma and the indistinctly margined areola of the propodeum.

#### Etymology.

The specific name is from the locality of the holotype, Guapiles, in Limon Province.

**Figure 157.**
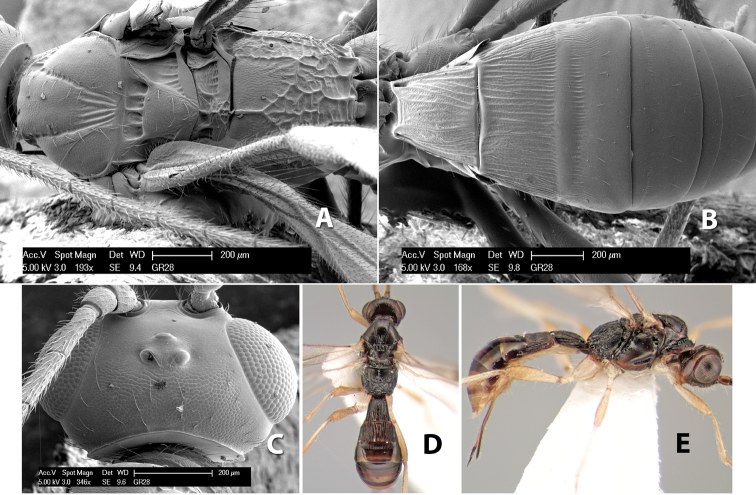
*Heterospilus guapilensis* Marsh, sp. n.: **A–C** paratype **D–E** holotype.

### Heterospilus ixcatec

Marsh sp. n.

http://zoobank.org/C47119CB-E259-400E-A6A6-0A8FA37FDD82

http://species-id.net/wiki/Heterospilus_ixcatec

Figures 158 , 159

#### Female.

Body size: 2.0 mm. Color: body dark brown, metasomal tergum 1 apically, tergum 2 medially and tergum 7 yellow; scape yellow without lateral brown stripe, flagellum brown; legs yellow. Head: vertex granulate; frons granulate; face granulate-rugose; temple in dorsal view narrow, sloping behind eye, width equal to 1/2 eye width; malar space greater than 1/4 eye height; ocell-ocular distance about 2.5 times diameter of lateral ocellus; 23–24 flagellomeres. Mesosoma: mesoscutal lobes granulate; notauli scrobiculate, meeting at scutellum in triangular rugose area; scutellum granulate; prescutellar furrow with 3–5 cross carinae; mesopleuron granulate; precoxal sulcus scrobiculate, shorter than mesopleuron; venter granulate; propodeum with basal median areas small and indistinct, not margined, granulate, basal median carina absent, areola not margined, areolar area areolate-rugose, lateral areas entirely rugose. Wings: fore wing vein r shorter than vein 3RSa, vein 1cu-a beyond vein 1M; hind wing vein SC+R present, vein M+CU shorter than vein 1M. Metasoma: first tergum longitudinally costate, length equal to apical width; second tergum longitudinally costate; anterior transverse groove present, straight; posterior transverse groove weakly present or absent; third tergum costate at extreme base, remainder smooth; terga 4–7 smooth; ovipositor as long as metasomal terga 1 and 2 combined.

#### Holotype female.

Top label (white, partially printed and hand written) - Costa Rica: Guanacaste [;] Santa Rosa Natl. Park [;] 500m, ex. Malaise trap [;] Site #: BH-11-O [;] Dates: 8.ii–2.iii.1986 [;] I.D. Gauld & D. Janzen; second label (white, printed) - [BH] Bosque Humedo [;] mature evergreen dry forest [;] [O] in clearing, fully [;] isolated part of day; third label (red, partially printed and hand written) - HOLOTYPE [;] Heterospilus [;] ixcatec [;] P. Marsh. Deposited in ESUW.

#### Paratypes.

2 ♀♀, same data as holotype except: Site #: BH-9-O and BH-12-C; Dates: 13.iv–4.v.1986 and 2–23.iii.1986; second label - [BH] Bosque Humedo [;] mature evergreen dry forest [;] [C] more or less fully [;] shaded as possible (ESUW). 1 ♀, top label - Costa Rica: Guanacaste [;] Santa Rosa National Pk. [;] 300m, Malaise, Ian Gauld [;] 31.i–21.ii.1987; second label - Bosque Humedo [;] Mature dry forest [;] high proportion [;] Evergreen species [;] Sun (ESUW). 1 ♀, Costa Rica: Guanacaste [;] P. N. Guanacaste [;] below Pitilla, 500m [;] 7–8.iii.1990, J. S. Noyes (ESUW).

#### Comments.

The short squat body, the rugose area where the notauli meet and the bicolored metasomal terga are distinctive for this species.

#### Etymology.

Named for the Ixcatec, an indigenous people of Mexico.

**Figure 158.**
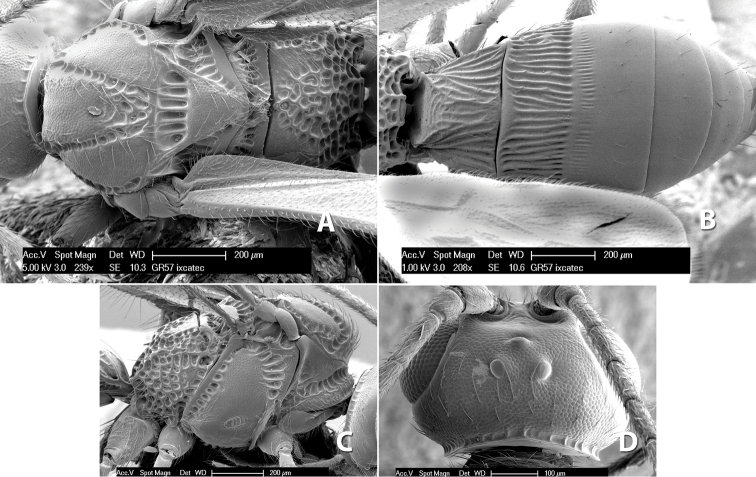
*Heterospilus ixcatec* Marsh, sp. n., paratype.

**Figure 159.**
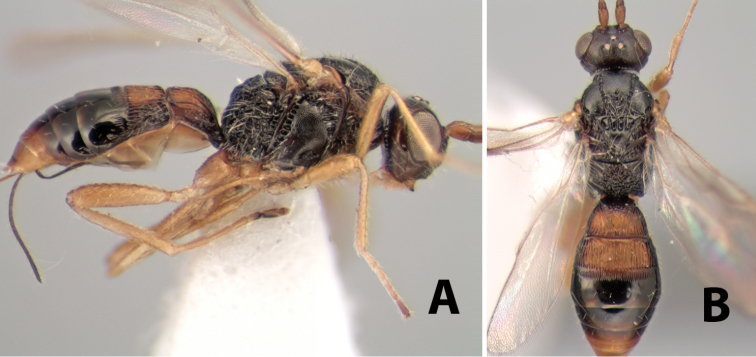
*Heterospilus ixcatec* Marsh, sp. n., holotype.

### Heterospilus ixil

Marsh sp. n.

http://zoobank.org/6017D5DD-0AE1-4CBA-ACEF-C874508AFA29

http://species-id.net/wiki/Heterospilus_ixil

Figure 160

#### Female.

Body size: 2.0 mm. Color: head and mesosoma dark brown, metasoma lighter brown with tergum 1 apically, tergum 2 medially and terga 5–7 yellow; scape yellow without lateral brown stripe, flagellum entirely brown; wing veins including stigma brown; legs yellow. Head: vertex granulate; frons granulate; face granulate; temple in dorsal view broad but only slightly bulging behind eye, width equal to 1/2 eye width; malar space slightly greater than 1/4 eye height; ocell-ocular distance about 2.5 times diameter of lateral ocellus; 18–20 flagellomeres. Mesosoma: mesoscutal lobes weakly granulate, lateral lobes partially smooth; notauli scrobiculate, meeting at scutellum in small rugose area; scutellum weakly granulate; prescutellar furrow with 3 cross carinae; mesopleuron granulate; precoxal sulcus scrobiculate, shorter than mesopleuron; venter smooth; propodeum with basal median areas small and distinct but not margined, granulate, basal median carina absent, areola not margined, areolar area areolate-rugose, lateral areas entirely rugose. Wings: fore wing vein r shorter than vein 3RSa, vein 1cu-a slightly beyond vein 1M; hind wing vein SC+R absent, vein M+CU shorter than vein 1M. Metasoma: first tergum longitudinally costate, length equal to apical width; second tergum longitudinally costate; anterior transverse groove present, straight; posterior transverse groove very weak or absent; third tergum costate basally, smooth apically; terga 4–7 smooth; ovipositor half as long as metasoma.

#### Holotype female.

Top label (white, partially printed and hand written) - Costa Rica: Guanacaste [;] Santa Rosa Natl. Park [;] 300m, ex. Malaise trap [;] Site #: H-1-O [;] Dates: 14.vi–5.vii.1986 [;] I.D. Gauld & D. Janzen; second label (white, printed) - [H] open regenerating [;] woodland <10 years old [;] [O] in clearing, fully [;] isolated part of day; third label (red, partially printed and hand written) - HOLOTYPE [;] Heterospilus [;] ixil [;] P. Marsh. Deposited in ESUW.

#### Paratypes.

1 ♀, top label - Costa Rica: Guanacaste [;] Santa Rosa Natl. Park [;] 300m, ex. Malaise trap [;] Site #: (blank) [;] Dates: 4-24-V 1986 [;] I.D. Gauld & D. Janzen; second label - [BH] Bosque Humedo [;] mature evergreen dry forest [;] [C] more or less fully [;] shaded as possible (ESUW).

#### Comments.

The longer ovipositor, brown flagellum and partially smooth mesoscutal lobes are distinctive for this species.

#### Etymology.

Named for the Ixil, an indigenous people of Guatemala.

**Figure 160.**
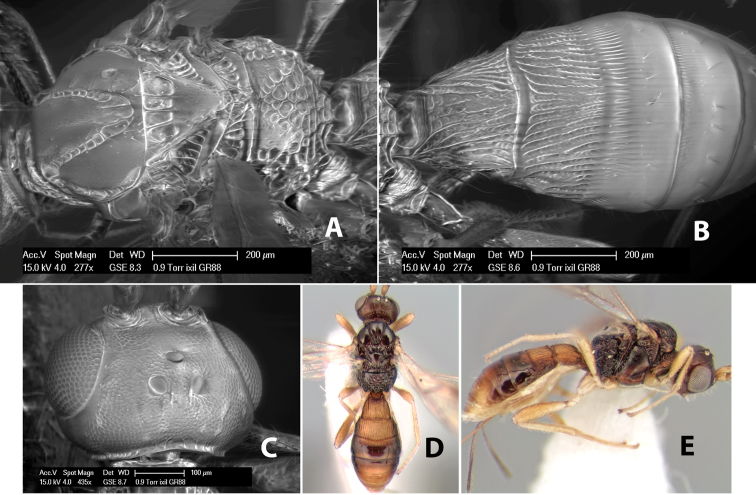
*Heterospilus ixil* Marsh, sp. n., holotype.

### Heterospilus jabillosensis

Marsh sp. n.

http://zoobank.org/0C5D5E56-C4BD-47FA-91D0-BEBCF8100C63

http://species-id.net/wiki/Heterospilus_jabillosensis

Figure 161

#### Female.

Body size: 2.0 mm. Color: head dark brown; scape yellow with lateral longitudinal brown stripe, flagellum brown with apical white annulus, apical 3–5 flagellomeres brown; mesosoma dark brown, propleuron and pronotal collar often lighter; metasomal terga light brown to brown, terga 1 apically and 2 medially often lighter yellow, apical terga yellow; wing veins including stigma brown; legs yellow. Head: vertex weakly granulate; frons smooth; face smooth, sometimes weakly striate below antennae; temple in dorsal view narrow, sloping behind eye, width less than 1/2 eye width; malar space greater than 1/4 eye height; ocell-ocular distance greater than 2.5 times diameter of lateral ocellus; 15–18 flagellomeres. Mesosoma: mesoscutal lobes smooth; notauli smooth, meeting at scutellum in unsculptured area; scutellum smooth; prescutellar furrow with 3–5 cross carinae; mesopleuron granulate; precoxal sulcus smooth, shorter than mesopleuron; venter smooth; propodeum with basal median areas distinct, not or weakly margined, basal median carina absent, areola not margined, areolar area rugose, lateral areas entirely rugose. Wings: fore wing vein r shorter than vein 3RSa, vein 1cu-a interstitial with vein 1M; hind wing vein SC+R absent, vein M+CU shorter than vein 1M. Metasoma: first tergum longitudinally costate, often granulate medially, length equal to or slightly greater than apical width; second tergum longitudinally costate; anterior transverse groove present, straight; posterior transverse groove present; third tergum entirely smooth except for costate transverse groove; terga 4–7 smooth; ovipositor as long as metasomal tergum 1.

#### Holotype female.

Top label (white, printed) - COSTA RICA, Alajuela [;] Jabillos, 100m [;] 24/III/1989 [;] col. Paul Hanson; second label (red, partially printed and hand written) - HOLOLTYPE [;] Heterospilus [;] jabillosensis [;] P. Marsh. Deposited in ESUW.

#### Paratypes.

3 ♀♀, same data as holotype (ESUW). 1 ♀, Costa Rica, Puntarenas [;] R.F. Golfo Dulce, 5km. [;] W. Piedras Blancas, 100m [;] I-1993, P. Hanson (ESUW). 1 ♀, COSTA RICA, Heredia [;] Chilamate, 75m [;] 25.III.1989 [;] Col. P. Hanson (ESUW). 1 ♀, COSTA RICA: Puntarenas [;] Reserva Forestal Golfo Dulce [;] 3km SW of Rincon, 10m [;] Mar-April 1992, P. Hanson [;] primary forest, Malaise trap (ESUW). 2 ♀♀, top label - Costa Rica: Guanacaste [;] Santa Rosa Natl. Park [;] 300m, ex. Malaise trap [;] Site #: (blank) [;] Dates: 16.xi–7.xii.1985 and 27.ix–18.x.1986 [;] I.D. Gauld & D. Janzen; second label - [H] open regenerating [;] woodland <10 years old [;] [O] on clearing, fully [;] isolated part of day (ESUW). 1 ♀, Costa Rica: Limon, Central [;] R.B. Hitoy Cerere, Est. Hitoy [;] Cerere, Send. Toma de Agua, [;] 100–140m, Malaise trap [;] 11.x–11.xi.1999, F. Umana [;] L.N. 184600-643400 #54013 (ESUW). 2 ♀♀, COSTA RICA: Puntarenas [;] RF Golfo Dulce el 200m [;] 24km W Piedras Blancas [;] P. Hanson vii.1992 and x.1992 (TAMU).

#### Comments.

The small body, short antennae and smooth mesoscutum are distinctive for this species.

#### Etymology.

Named for Jabillos, Alajuela Province, where part of the type series was collected.

**Figure 161.**
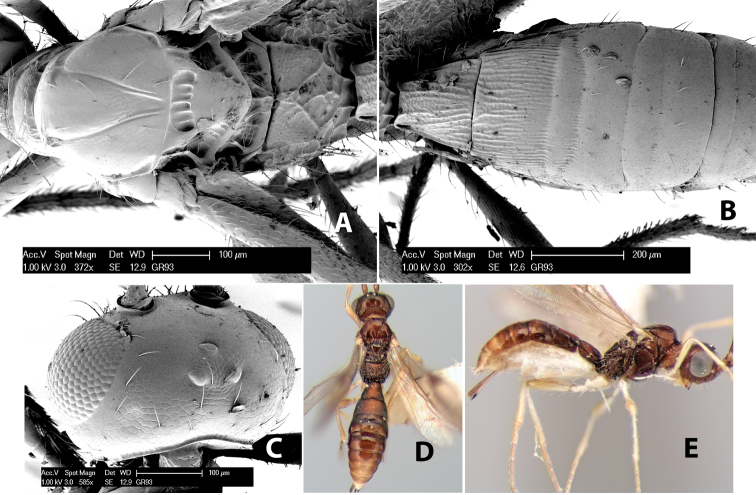
*Heterospilus jabillosensis* Marsh, sp. n.: **A–C, E** paratype **D** holotype.

### Heterospilus jennieae

Marsh sp. n.

http://zoobank.org/0CC8B43E-A212-4761-A3E2-2B5B26C85F7B

http://species-id.net/wiki/Heterospilus_jennieae

Figure 162

#### Female.

Body size: 3.5–4.0 mm. Color: head dark brown; scape yellow with lateral longitudinal brown stripe, flagellum brown with apical 8–10 flagellomeres white, apical flagellomere often brown; mesosoma dark brown, mesoscutal lobes lighter brown; metasomal terga dark brown, terga 4–7 usually lighter; wing veins brown, stigma bicolored brown with yellow at base; legs yellow. Head: vertex granulate, area behind ocelli granulate-striate; frons transversely striate; face striate; temple in dorsal view broad, not sloping behind eye, width equal to 1/2 eye width; malar space greater than 1/4 eye height; ocell-ocular distance 2.5 or more times diameter of lateral ocellus; 26–30 flagellomeres. Mesosoma: mesoscutal lobes granulate; notauli scrobiculate, meeting at scutellum in triangular rugose area; scutellum granulate; prescutellar furrow with 3 cross carinae; mesopleuron granulate; precoxal sulcus smooth, shorter than mesopleuron; venter granulate; propodeum with basal median areas margined, granulate, basal median carina absent, areola not margined, areolar area rugose, lateral areas rugose posteriorly, granulate or smooth anteriorly, propodeum with small but distinct tubercle above hind coxa. Wings: fore wing vein r shorter than vein 3RSa, vein 1cu-a beyond vein 1M; hind wing vein SC+R present, vein M+CU shorter than vein 1M. Metasoma: first tergum longitudinally costate, length equal to or slightly greater than apical width; second tergum longitudinally costate; anterior transverse groove present, straight; posterior transverse groove present; third tergum costate basally, smooth apically; terga 4–7 smooth; ovipositor as long as metasoma.

#### Holotype female.

Top label (white, printed) - COSTA RICA, Heredia [;] Est. Biol. La Selva, 50- [;] 150m, 10°26'N, 84°01'W [;] Mar 1993, INBio-OET; second label (white, printed) - 02 Marzo 1993 [;] M/05/020 [;] Bosque primario; third label - INBio bar code label; fourth label (red, partially printed and hand written) - HOLOTYPE [;] Heterospilus [;] jennieae [;] P. Marsh. Deposited in ESUW.

#### Paratypes.

4 ♀♀, same data as holotype with additional date of Apr 1993 (ESUW). 1 ♀, Costa Rica, Heredia Prov. [;] OTS. La Selva, 100m [;] 1993 II-III P. Hanson (ESUW). 2 ♀♀, Costa Rica, Heredia [;] Puerto Viejo, 100m [;] OTS-La Selva [;] III-1991 P. Hanson (ESUW). 2 ♀♀, Costa Rica, Heredia [;] 3km. S. Puerto Viejo [;] OTS-La Selva [;] III-IV-1993, P. Hanson (ESUW). 1 ♀, COSTA RICA-Heredia Prov. [;] La Selva Biological Station [;] 10°26'N, 84°01W 100m [;] Malaise trap 06,#347 [;] 3.ii.1994 [;] Project ALAS (M.06.347) (ESUW). 1 ♀, top label - COSTA RICA: Heredia [;] Pr: La Selva Biol. Sta. [;] 3km S. Pto. Viejo [;] 10°26'N, 84°01'W; second label - 22.vi.1991 [;] H.A. Hespenheide (ESUW). 1 ♀, top label - COSTA RICA: Prov. [;] Heredia, F. La Selva [;] 3km S Pto. Viejo [;] 10°26'N, 84°01W; second label - 12.xi.19?? MT#2 [;] T.W. Sherry (ESUW). 1 ♀, Costa Rica: Limon, Sec. Cocori [;] 30Km al N. Cariari, 100m [;] xii.1994, E. Rojas, Malaise [;] L.N. 286000-567500 #4525 (ESUW). 1 ♀, Costa Rica: Limon [;] Sector Cocori, 100m [;] 30km N Carari, i.1995 [;] E. Rojas, Malaise, #4526 [;] L.N. 286000-567500 #4525 (ESUW). 1 ♀, Costa Rica: Limon [;] 30km N Carari, 100m [;] Sector Cocori, Malaise [;] iii.1995, E. Rojas #4524 [;] L.N. 286000-567500 #4525 (ESUW). 1 ♀, Costa Rica: Limon, ACLAC [;] Central, R.B. Hitoy Cerere, [;] Est Hitoy Cerere, Sect. Toma [;] de Agua, 100m, 17.iv–8.v.1999. [;] F. Umana, Malaise trap [;] L.N. 184600-643400 #52757 (ESUW). 1 ♀, COSTA RICA: [;] Limon [;] 4km NE Bribri [;] 50m, iv-vi 1990 [;] Col. Paul Hanson (ESUW). 1 ♀, Costa Rica: Puntarenas [;] San Vito, Las Cruces [;] Wilson Botanical Gardens [;] 18–22.iii.1990, 1150m [;] J.S. Noyes (ESUW). 1 ♀, Costa Rica: Alajuela [;] ACA, R.B.San Ramon [;] 875m, Malaise near [;] river, 10–26.viii.1998 [;] L.J. van der Ent (ESUW). 7 ♀♀, top label - COSTA RICA, Heredia: [;] Est. Biol. La Selva, 50- [;] 150m, 10°26'N, 84°01'W [;] Feb 1998, INBio-OET; second label - 5 and 19 Febrero 1998 [;] M/18/702 and 701 [;] Borde Suampo (INBC). 12 ♀♀, top label - COSTA RICA, Heredia: [;] Est. Biol. La Selva, 50- [;] 150m, 10°26'N, 84°01'W [;] Mar 1998, INBio-OET; second label - 5 and 19 Marzo 1998 [;] M/18/703 and 704 [;] Borde Suampo (INBC). 8 ♀♀, top label - COSTA RICA, Heredia: [;] Est. Biol. La Selva, 50- [;] 150m, 10°26'N, 84°01'W [;] Apr 1998, INBio-OET; second label - 2, 16 and 19 Febrero 1998 [;] M/18/705, 706 and 707 [;] Borde Suampo (INBC). 1 ♀, top label - COSTA RICA, Heredia: [;] Est. Biol. La Selva, 50- [;] 150m, 10°26'N, 84°01'W [;] Apr 1996, INBio-OET; second label - 1 Abril 1996 [;] Bosque primario [;] M/12/613 (INBC).

#### Comments.

The lighter colored mesoscutal lobes, the granulate-striate vertex and the ovipositor length equal to length of metasoma are distinctive for this species. It is similar to *Heterospilus mixtec* but is distinguished by the lighter covered mesoscutal lobes.

#### Etymology.

Named for my loving wife and friend of nearly 50 years, Jennie.

**Figure 162.**
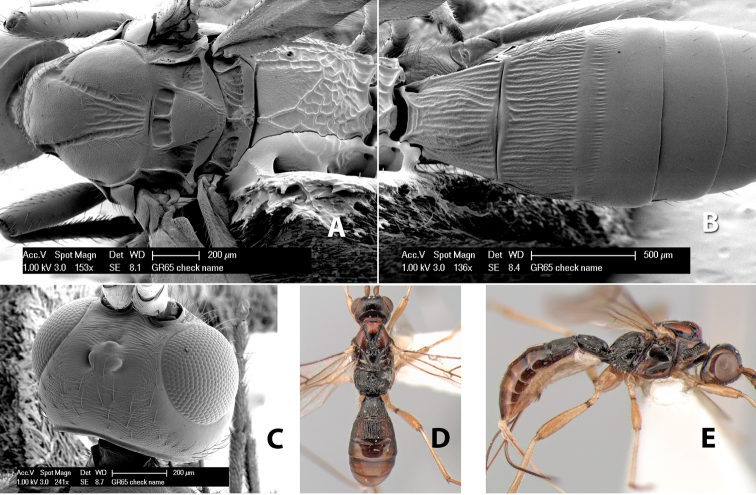
*Heterospilus jennieae* Marsh, sp. n.: **A–C** paratype **D–E** holotype.

### Heterospilus jonmarshi

Marsh sp. n.

http://zoobank.org/0C31C247-F5CB-47DB-9CA9-F6FA0CDADAEB

http://species-id.net/wiki/Heterospilus_jonmarshi

Figure 163

#### Female.

Body size: 3.0 mm. Color: body medium to dark brown, apical metasomal terga often lighter brown than anterior terga, rarely all terga lighter brown than mesosoma; scape light brown or yellow, usually with lateral longitudinal brown stripe, rarely stripe weak or absent, flagellum entirely brown; wing veins brown, stigma yellow; legs yellow. Head: vertex granulate; frons granulate; face granulate; temple in dorsal view broad but not bulging behind eye, width greater than 1/2 eye height; malar space greater than 1/4 eye height; ocell-ocular distance 2.5 times diameter of lateral ocellus; 21–25 flagellomeres. Mesosoma: mesoscutal lobes granulate and shining, rarely partially smooth; notauli scrobiculate, meeting at scutellum in triangular rugose area, often with median longitudinal rugose groove extending anteriorly on median mesoscutal lobe; scutellum weakly granulate, often smooth; prescutellar furrow with 3 cross; mesopleuron granulate; precoxal sulcus scrobiculate, shorter than mesopleuron; venter granulate; propodeum with basal median areas small and not margined, granulate, basal median carina absent, areola not distinct, areolar area areolate-rugose, lateral areas entirely rugose. Wings: fore wing vein r slightly shorter or equal to vein 3RSa, vein 1cu-a beyond vein 1M; hind wing vein SC+R present, vein M+CU shorter than vein 1M. Metasoma: first tergum longitudinally costate laterally, rugose medially, length slightly less than apical width; second tergum longitudinally costate-rugose; anterior transverse groove present, straight; posterior transverse groove present but usually weakly indicated; third tergum costate basally, smooth apically; terga 4–7 smooth; ovipositor equal to half length of metasoma.

#### Holotype female.

Top label (white, partially printed and hand written) - Costa Rica: Guanacaste [;] Santa Rosa Natl. Park [;] 300m, ex. Malaise trap [;] Site #: H-3-O [;] Dates: 2–23.iii.1986 [;] I.D. Gauld & D. Janzen; second label (white, printed) - [H] open regenerating [;] woodland, 10 years old [;] [O] in clearing, fully [;] isolated part of day; third label (red, partially printed and hand written) - HOLOTYPE [;] Heterospilus [;] jonmarshi [;] P. Marsh. Deposited in ESUW.

#### Paratypes.

12 ♀♀, same data as holotype except: Site: H-1-O; Dates: 23.iii–19.iv.1986, 21.ii–14.iii.1987, 20.xii.86–10.i.1987, 18.x–8.xi.1986, 6–27.ix.1986, 26.vii–14.viii.1986, 14.viii–6.ix.1986, 14.iv–4.v.1986 and 31.i–21.ii.1987 (ESUW). 4 ♀♀, top label - Costa Rica: Guanacaste [;] Santa Rosa Natl. Park [;] 300m, ex. Malaise trap [;] Site #: BH-11-O and BH-9-O [;] Dates: 4–24.v.1986, 18.i–8.ii.1986, 23.iii–13.iv.1986 and 13.iv–4.v.1986 [;] I.D. Gauld & D. Janzen; second label - [BH] Bosque Humedo [;] mature evergreen dry forest [;] [O] in clearing, fully [;] isolated part of day (ESUW). 4 ♀♀, top label - Costa Rica: Guanacaste [;] Santa Rosa Natl. Park [;] 300m, ex. Malaise trap [;] Site #: SE-6-C [;] Dates: 16.xi–7.xii.1985, 8.ii–2.iii.1986, 26.vii–14.viii.1986 and 20.xii.86–10.i.1987 [;] I.D. Gauld & D. Janzen; second label - [SE] Bosque San Emilio [;] 50yr old deciduous forest [;] [C] more or less fully [;] shaded as possible (ESUW). 3 ♀♀, top label - Costa Rica: Guanacaste [;] Santa Rosa Natl. Park [;] 300m, ex. Malaise trap [;] Site #: 7 [;] Dates: 6–27.xi.1986 and 14.viii–6.ix.1986 [;] I.D. Gauld & D. Janzen; second label - [SE] Bosque San Emilio [;] 50yr old deciduous forest [O] in clearing, fully [;] isolated part of day (ESUW). 3 ♀♀, top label - Costa Rica: Guanacaste [;] Santa Rosa Natl. Park [;] 300m, ex. Malaise trap [;] Site #: H-2-C and H-4-C [;] Dates: 29.xi–20.xii.1986, 31.i–21.ii.1987 and 4–24.v.1986 [;] I.D. Gauld & D. Janzen; second label - [H] open regenerating [;] woodland, 10 years old [;] [C] more or less fully [;] shaded as possible (ESUW). 1 ♀, top label - Costa Rica: Guanacaste [;] Santa Rosa Natl. Park [;] 300m, ex. Malaise trap [;] Site #: BH-10-C [;] Dates: 2–23.iii.1986 [;] I.D. Gauld & D. Janzen; second label - [BH] Bosque Humedo [;] mature evergreen dry forest [;] [C] more or less fully [;] shaded as possible (ESUW). 1 ♀, top label - Costa Rica: Guanacaste [;] Santa Rosa National Pk. [;] 300m, Malaise, Ian Gauld [;] 18.x–8.xi.1986; second label - Open regenerating [;] Woodland less than [;] 10 yrs. Old. Sun; third label - H-3-O [;] 18.x–8.xi.86 (ESUW). 1 ♀, top label - Costa Rica: Guanacaste [;] Santa Rosa National Pk. [;] 300m, Malaise, Ian Gauld [;] 10–31.i.1987; second label - Bosque San Emilio [;] 50 yr. Old deciduous [;] Forest [;] Full Shade; third label - SE-6-C [;] 10–31.i.87 (ESUW). 3 ♀♀, C.R.: Guanacaste [;] P.N. Santa Rosa [;] 200m, January 1991 [;] Col. Paul Hanson (ESUW). 2 ♀♀, Costa Rica: Guanacaste, Bagaces [;] Pque. Ntl. Palo Verde, Sct. P. Verde [;] Cerro, Guayacan, 212m, Malaise [;] 13.x–11.xi.1999, I. Jimenez [;] L.N. 259350-389600 #54006 (ESUW). 1 ♀, Costa Rica: Guanacaste [;] Santa Rosa National Pk. [;] 300m, Malaise H-2-C4 [;] regenerating woodland [;] <10 yr. old, Ian Gauld [;] 4–24.v.1986, full shade (ESUW). 1 ♀, S.RosaPark. Guan. [;] C. Rica 17 Jan 78 [;] D.H. Janzen [;] Dry Hill (AEIC).

#### Comments.

The median longitudinal rugose line on the mesoscutum, the short and broad metasomal tergum 1 and the yellow stigma are distinctive for this species.

#### Etymology.

Named for my son, Jon Marsh.

**Figure 163.**
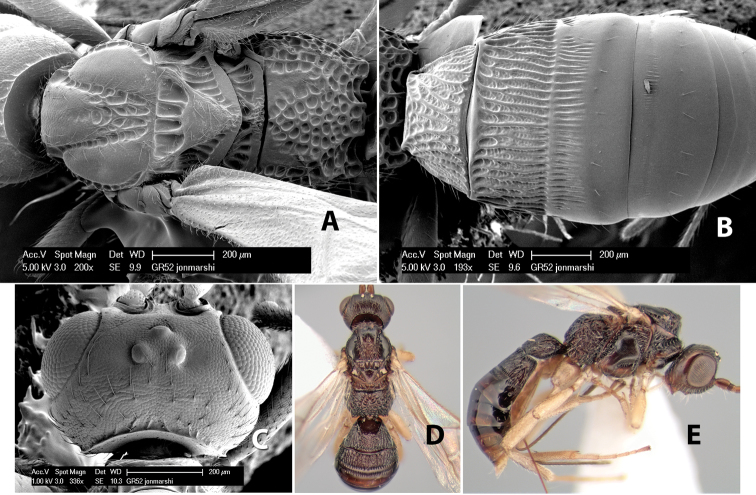
*Heterospilus jonmarshi* Marsh, sp. n.: **A–C** paratype **D–E** holotype.

### Heterospilus kellieae

Marsh sp. n.

http://zoobank.org/A3CB32DB-98B6-4DEB-B5C8-D445B9934E73

http://species-id.net/wiki/Heterospilus_kellieae

Figure 164

#### Female.

Body size: 2.5–3.0 mm. Color: body dark brown; scape yellow with lateral longitudinal brown stripe, flagellum brown with apical white annulus, apical 3–5 flagellomeres brown; wing veins including stigma brown; legs yellow. Head: vertex granulate; frons granulate; face granulate; temple in dorsal view narrow, sloping behind eye, width less than 1/2 eye width; malar space greater than 1/4 eye height; ocell-ocular distance about 2.5 times diameter of later ocellus; 20–23 flagellomeres. Mesosoma: mesoscutal lobes granulate; notauli weakly scrobiculate or smooth, meeting at scutellum in small costate or rarely smooth area; scutellum granulate; prescutellar furrow with 5 cross carinae; mesopleuron granulate; precoxal sulcus weakly scrobiculate or smooth, shorter than mesopleuron; venter granulate; propodeum with basal median areas margined, granulate, basal median carina absent, areola not distinct, areolar area rugose, lateral areas mostly rugose with small granulate area at base, propodeum usually with weak but distinct tubercle above hind coxa. Wings: fore wing vein r shorter than vein 3RSa, vein 1cu-a beyond vein 1M; hind wing vein SC+R absent, vein M+CU shorter than vein 1M. Metasoma: first tergum longitudinally costate-granulate, length equal to or slightly greater than apical width; second tergum longitudinally costate-granulate; anterior transverse groove present, straight; posterior transverse groove present and wide; third tergum entirely granulate except for costate transverse groove; terga 4–7 granulate; ovipositor slightly shorter than metasomal tergum 1.

#### Holotype female.

Top label (white, printed) - Costa Rica: Puntarenas, [;] R.F. Golfo Dulce, 5km. [;] W. Piedras Blancas, 100m [;] xi-xii.1991, P. Hanson [;] Malaise nr. second growth; second label (red, partially printed and hand written) - HOLOTYPE [;] Heterospilus [;] kellieae [;] P. Marsh. Deposited in ESUW.

#### Paratypes.

12 ♀♀, Costa Rica: Puntarenas [;] Golfo Dulce, 24km W. [;] Piedras Blancas, 200m [;] ii.1993, xii.1993 and iv.1993, Paul Hanson (ESUW). 3 ♀♀, COSTA RICA: Puntarenas [;] R.F. Golfo Dulce, [;] 24km W. Piedras Blancas, [;] 200m [;] Feb. 1992, Paul Hanson (ESUW). 12 ♀♀, COSTA RICA: [;] Puntar. Golfo Dulce [;] 24km W Piedras Blancas [;] 200m, vi-viii 1989 [;] Hanson (ESUW). 6 ♀♀, Costa Rica: Puntarenas [;] R.F. Golfo Dulce, 24km. W [;] Piedras Blancas, 200m [;] III.1993, P. Hanson (ESUW). 3 ♀♀, COSTA RICA: Puntar [;] Golfo Dulce 24km W [;] Piedras Blancas [;] 200m, vii-ix 1990 [;] Col. Paul Hanson (ESUW). 1 ♀, Costa Rica: Puntarenas [;] R.F. Golf Dulce, 24km. [;] W. Piedras Blancas, 200m [;] I.1993, P. Hanson (ESUW). 5 ♀♀, COSTA RICA: Puntar [;] Golfo Dulce, 10km W [;] Piedras Blancas, 100m [;] VI-VIII 1989, Hanson (ESUW). 9 ♀♀, Costa Rica: Puntarenas [;] R.F. Golfo Dulce, 5km. W. [;] Piedras Blancas, 100m [;] VI-VII-1993, I-1993, VIII-IX-1993 and IV-V-1993, P. Hanson (ESUW). 1 ♀, Costa Rica: Puntarenas [;] Res. Forestal Golfo Dulce [;] 24km. W. Piedras Blancas, 200m [;] xi.1990, P. Hanson [;] Malaise, primary forest (ESUW). 15 ♀♀, COSTA RICA: Puntarenas [;] Rd. to Rincon, 24km W. [;] Pan-Amer. Hwy, 200m [;] III-V 1989, Hanson & Gauld (ESUW). 2 ♀♀, COSTA RICA: Puntarenas [;] Rd. to Rincon, 10km W. [;] of Pan-Amer. Hwy, 100m [;] III-V 1989, Hanson & Gauld (ESUW). 2 ♀♀, COSTA RICA: Puntarenas [;] Rd. to Rincon, 24km W. [;] of Pan-Amer. Hwy, 200m [;] II-III 1989, Hanson & Gauld (ESUW). 1 ♀, Costa Rica: Puntarenas [;] R.F. Golfo Dulce, [;] 3km SW. Rincon, 10m, [;] vi.1991, Paul Hanson (ESUW). 1 ♀, COSTA RICA: Puntarenas [;] Reserva Forestal Golfo Dulce [;] 3km SW of Rincon, 10m [;] Mar-April 1992 and November 1992, P. Hanson [;] primary forest, Malaise trap (ESUW). 2 ♀♀, Costa Rica: Puntarenas [;] Res. Forestal Golfo Dulce [;] 3km. SW Rincon, 10m [;] xii.1992, P. Hanson [;] Malaise, primary forest (ESUW). 1 ♀, COSTA RICA: Puntar [;] Golfo Dulce 3km SW [;] Rincon [;] 10m, iii-v 1989 [;] Col. Paul Hanson (ESUW). 1 ♀, Costa Rica: Puntarenas [;] R.F. Golfo Dulce [;] 3km. SW. Rincon, 10m [;] Oct. 1991, Paul Hanson (ESUW). 2 ♀♀, Costa Rica: Puntarenas [;] R.F.Golfo Dulce, 3km [;] SW Rincon, 10m [;] Malaise-primary forest [;] viii.1991, P. Hanson (ESUW). 1 ♀, COSTA RICA: Puntarenas [;] Reserva Forestal Golfo Dulce [;] 3km south-west of Rincon [;] 10m, July 1991, P. Hanson [;] primary forest, Malaise trap (ESUW). 6 ♀♀, Costa Rica: Puntarenas [;] San Vito, Las Cruces [;] Wilson Botanical Gardens [;] 18–22.iii.1990, 1150m [;] J.S. Noyes (ESUW). 1 ♀, Costa Rica: Puntarenas, ACO [;] Golfito, PN Corcovado [;] Est. Agujas, Quebraditas [;] 640m, 15.ix–15.x.1999 [;] J. Azofeifa, Malaise, #53492 [;] L.S. 275200-520100 (ESUW). 1 ♀, Costa Rica: Puntarenas, ACO [;] Golfito, R.F. Golfo Dulce [;] Est. Agujas, 250–350m [;] 1–11.xi.1991, J. Azofeifa [;] Red de Golpe #54023 [;] L.S. 2767500-526550 (ESUW). 1 ♀, Costa Rica: Puntarenas, ACO [;] Golfito, Est. Agujas, 250–350m [;] Res. Ftal. Golfo Dulce, Amarilla [;] 3–24.vii.1999, J. Azofeifa [;] L.S.276750-526550 #52839 (ESUW). 1 ♀, top label - Costa Rica: Puntarenas [;] ACO, R.F. Golfo Dulce [;] Golfito, Estacion Agujas [;] La Bonanza, 495m; second label - 15 Sept.-15 Oct. 1999 [;] J. Azofeifa, Malaise trap [;] L.S. 276000-526550 [;] #53487 (ESUW). 1 ♀, Costa Rica, Puntarenas [;] San Vito, 1200m, café [;] III-IV-1996, P. Hanson (ESUW). 1 ♀, Costa Rica: San Jose [;] Ciudad Colon [;] 800m, iv-v 1990 [;] Col. Luis Fournier (ESUW). 2 ♀♀, Costa Rica: Heredia [;] Est. Biol. La Selva [;] 50–150m, 10.26 N [;] 84.01 W, Aug.1992 (ESUW). 1 ♀, COSTA RICA-Heredia Prov. [;] La Selva Biological Station [;] 10°26'N, 84°01'W, 100m [;] Malaise trap 01, #304 [;] 3.1.1994 [;] Project ALAS (M.01.304) (ESUW). 1 ♀, COSTA RICA, Heredia [;] Chilamate, 75m [;] VII-VIII/1989 [;] col. Paul Hanson (ESUW). 1 ♀, COSTA RICA: [;] Heredia, Chilamate [;] 75m, iv-vi 1990 [;] Hanson & Godoy (ESUW). 14 ♀♀, COSTA RICA: Limon [;] 16km W. Guapiles [;] 400m, III-V 1990, I-III 1990, III 1989, II 1989 and April 1989 [;] col. Paul Hanson (ESUW). 1 ♀, COSTA RICA, Limon [;] sur de Iriquois [;] 300m, 22/V/1987 [;] Col. Paul Hanson (ESUW). 1 ♀, COSTA RICA, Alajuela [;] Jabillos, 100m [;] 24/III/1989 [;] col. Paul Hanson (ESUW). 1 ♀, Costa Rica: Alajuela [;] 5km. W San Ramon [;] 1200m, November 1996 [;] O. Castro & P. Hanson (ESUW). 1 ♀, Costa Rica, Alajuela [;] Estac. Biol. San Ramon [;] 900m, X-XII-1995 [;] P. Hanson (ESUW). 1 ♀, Costa Rica: Alajuela [;] San Carlos, R.F. Arenal [;] Send Pilon, 600m, Malaise [;] 9.ix–1.x.1999, G. Carballo [;] L.N. 269100-467900 #53937 (ESUW). 1 ♀, top label - Costa Rica: Guanacaste [;] Santa Rosa Natl. Park [;] 300m, ex Malaise trap [;] Site #: H-1-O [;] Dates: 18.x–8.xi.1986 [;] I.D. Gauld & D. Janzen; second label - [H] open regenerating [;] woodland <10 years old [;] [O] in clearing, fully [;] isolated part of day (ESUW). 1 ♀, - Costa Rica: Guanacaste [;] Santa Rosa Natl. Park [;] 300m, ex Malaise trap [;] Site #: blank [;] Dates: 16.xi–7.xii.1985 [;] I.D. Gauld & D. Janzen; second label - [BH] Bosque Humedo [;] mature evergreen dry forest [;] [C] more or less fully [;] shaded as possible (ESUW). 1 ♀, top label - Costa Rica: Guanacaste [;] Santa Rosa National Pk. [;] 300m, Malaise, Ian Gauld [;] 18.x–8.xi.1986; second label - Bosque San Emilio [;] 50 yr. old deciduous [;] forest [;] Full Shade; third label - SE-8-C [;] 18.x–8.xi.86 (ESUW). 1 ♀, top label - Costa Rica: Guanacaste [;] 9km S. Santa Cecilia [;] Estacion Pitilia, 700m [;] vi.1996, Malaise trap; second label - C. Moraga & P. Rios [;] L.N. 330200-380200 [;] 47562 (ESUW). 2 ♀♀, Costa Rica: Guanacaste [;] P.N. Guanacaste [;] below Pitilia, 500m [;] 7–8.iii.1990, J. s. Noyes (ESUW). 5 ♀♀, COSTA RICA, Guanac. [;] Estac. Pitilia, 9Km S [;] Santa Cecilia, 700m [;] IV/1989, V/1989 and VI/1989, I. Gauld (ESUW). 1 ♀, Costa Rica: Guanacaste [;] Arenales, W. side of [;] Volcan Cacao, 900m [;] xi-xii 1990, P. Hanson (ESUW). 1 ♀, Costa Rica, Guanacaste Pr. [;] Guanac. Conserv. Area [;] Estacion Pitilia, 680m [;] M.trap, 11–20 vii 1997 [;] 2x day, L.J. van der Ent (ESUW). 1 ♀, Costa Rica: Guanacaste, ACT [;] Bagaces, P.N. Palo Verde [;] Sector Palo Verde, Cerro [;] Guayacan, 212m, Malaise [;] 15.vii–18.viii.1999, I. Jimenez [;] L.N. 259350-389600 #53256 (ESUW). 1 ♀, Costa Rica: Guanacaste, Bagaces [;] Pque. Ntl. Palo Verde, Sct. P. Verde [;] Cerro, Guayacan, 212m, Malaise [;] 13.x–11.xi.1999, I. Jimenez [;] L.N. 259350-389600 #54006 (ESUW). 1 ♀, Sirena, Osa Pen. [;] VII 77 Cos. Rica [;] D. H. Janzen (AEIC). 2 ♀♀, Costa Rica: Heredia [;] Braulio Carillo N.P. [;] 250–500m IV.10.85 [;] Henri Goulet (AEIC). 1 ♀, COSTA RICA: *Punt-* [;] *arenas*. 7km SW Rincon [;] 31.v–7.vi.1998; B. Brown [;] & V. Berezovskiy; Mal. [;] Trp. #3: 1° forest (AEIC). 1 ♀, top label - COSTA RICA, Heredia: [;] Est. Biol. La Selva, 50- [;] 150m, 10°26'N, 84°01'W [;] May 1996, INBio-OET; second label - 2 Mayo 1996 [;] Bosque secundario [;] M/02/628 (INBC). 1 ♀, top label - COSTA RICA, Heredia: [;] Est. Biol. La Selva, 50- [;] 150m, 10°26'N, 84°01'W [;] Apr 1998, INBio-OET; second label - 30 Abril 1998 [;] Boide Suampo [;] M/18/707 (INBC). 1 ♀, top label - COSTA RICA, Heredia: [;] Est. Biol. La Selva, 50- [;] 150m, 10°26'N, 84°01'W [;] Mar 1998, INBio-OET; second label - 19 Marzo 1998 [;] Boide Suampo [;] M/18/704 (INBC). 2 ♀♀, COSTA RICA, Guanac. [;] Estac. Pitilla, 9Km S [;] Santa Cecilia, 700m [;] VI/1989, I. Gauld (MICR). 1 ♀, COSTA RICA, San José [;] P.N.BraulioCarrillo [;] p.5Km E tunnel, 1000m [;] VI/1989, col. Hanson (MICR). 1 ♀, COSTA RICA, Heredia [;] Rio Frio, Banano [;] 100m, XI-XII 1989 [;] col. Edgar Quirós (MICR). 3 ♀♀, COSTA RICA, Puntar. [;] Golfo Dulce, 24km W. [;] PiedrasBlancas, 200m [;] III-VI-90 Hanson (MICR). 1 ♀, COSTA RICA, Puntarenas [;] San Vito, Jardin Bot. [;] Las Cruces, VI-VII/89 [;] 1200m, Col. P. Hanson (MICR). 10 ♀♀, COSTA RICA: Puntarenas [;] RF Golfo Dulce el 200m [;] 24km W Piedras Blancas [;] P. Hanson vii.1992, ix.1992, xi.1992, xii.1992 and vi.1993 (TAMU).

#### Comments.

The short ovipositor, granulate metasomal terga 4–7 and the nearly unsculptured area where the notauli meet are distinctive for this species.

#### Etymology.

Named for my daughter, Kellie Holoski.

**Figure 164.**
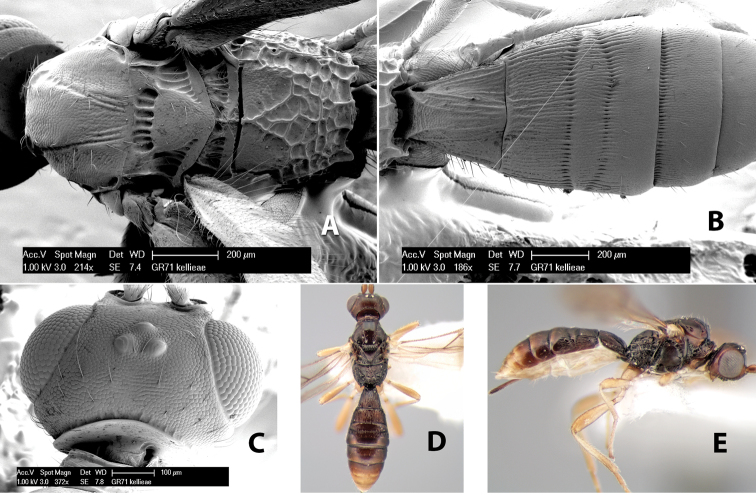
*Heterospilus kellieae* Marsh, sp. n.: **A–C** paratype **D–E** holotype.

### Heterospilus kikapu

Marsh sp. n.

http://zoobank.org/E2DB76B2-4EC3-4107-993E-9AB657E34B49

http://species-id.net/wiki/Heterospilus_kikapu

Figure 165

#### Female.

Body size: 2.5–3.0 mm. Color: entire body light brown or honey yellow; scape yellow without lateral brown stripe, flagellum brown with apical 5–6 flagellomeres white, apical one sometimes brown; legs yellow; wing veins including stigma brown. Head: vertex granulate; frons granulate; face striate; temple in dorsal view broad, equal to 1/2 eye width; malar space greater than 1/4 eye height; ocell-ocular distance about 2.5 times diameter of lateral ocellus; 21–22 flagellomeres. Mesosoma: mesoscutal lobes weakly granulate, nearly smooth; notauli weakly scrobiculate or smooth, meeting at scutellum in triangular costate area; scutellum weakly granulate or smooth; prescutellar furrow with one cross carina; mesopleuron weakly granulate; precoxal sulcus smooth, shorter than mesopleuron; venter granulate; propodeum with basal median areas margined, granulate, basal median carina absent, areola distinctly margined, areolar area rugose, lateral areas rugose posteriorly, granulate anteriorly, propodeum with small but distinct tubercle just above each hind coxae. Wings: fore wing vein r shorter than vein 3RSa, vein 1cu-a beyond vein 1M; hind wing vein SC+R present, vein M+CU slightly shorter than vein 1M. Metasoma: first tergum longitudinally costate, length slightly greater than apical width; second tergum longitudinally costate; anterior transverse groove present, straight; posterior transverse groove present; third tergum costate basally, smooth apically; terga 4–7 smooth; ovipositor as long as metasoma.

#### Holotype female.

Top label (white, printed) - COSTA RICA, Heredia [;] Est. Biol. La Selva, 50- [;] 150m, 10°26'N, 84°01'W [;] May 1993. INBio-OET; second label (white, printed) - 18 Mayo 1993 [;] Bosque secundario [;] M/04/099; third label - INBio bar code; fourth label (red, partially printed and hand written) - HOLOTYPE [;] Heterospilus [;] kikapu [;] P. Marsh. Deposited in ESUW.

#### Paratypes.

1 ♀, Costa Rica, Heredia Prov. [;] OTS. La Selva, 100m [;] 1993 II-III P. Hanson (ESUW). 1 ♀, COSTA RICA, Puntarenas [;] San Vito, Jardin Bot. [;] Las Cruces, XII 1988 [;] 1200m, Col. P. Hanson (MICR).

#### Comments.

The single cross carina in the prescutellar furrow and the light brown body are distinctive for this species.

#### Etymology.

Named for the Kikapú, an indigenous people of Mexico.

**Figure 165.**
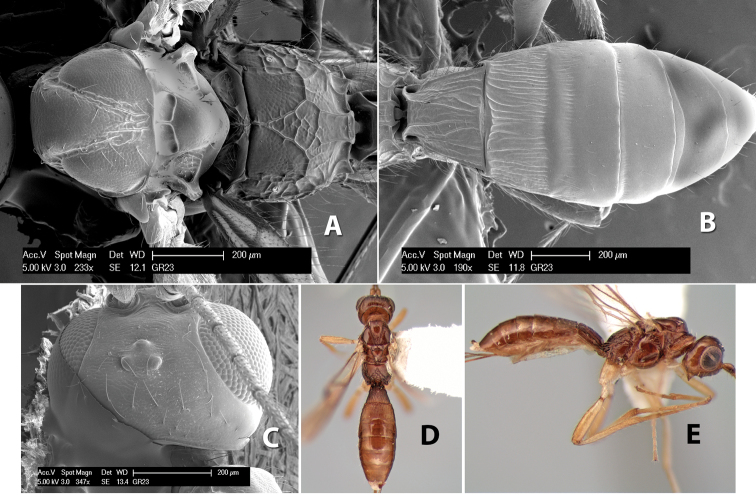
*Heterospilus kikapu* Marsh, sp. n.: **A–E** paratype **D–E** holotype.

### Heterospilus lasalturus

Marsh sp. n.

http://zoobank.org/0EB756B8-437D-4319-918D-CFC9FCD60976

http://species-id.net/wiki/Heterospilus_lasalturus

Figure 166

#### Female.

Body size: 2.5–3.0 mm. Color: head bicolored, vertex dark brown, face honey yellow; scape yellow with lateral longitudinal brown stripe, flagellum brown with apical white annulus, apical 3–5 flagellomeres brown; mesosoma light brown; metasoma brown, meddle terga usually somewhat darker; wing veins brown, stigma yellow; legs yellow. Head: vertex granulate; frons granulate; face smooth; temple in dorsal view broad but sloping behind eye, width equal to 1/2 eye width; malar space greater than 1/4 eye height; ocell-ocular distance 2.5 times diameter of lateral ocellus; 21–23 flagellomeres. Mesosoma: mesoscutal lobes granulate; notauli scrobiculate, meeting at scutellum in unsculptured area; scutellum granulate; prescutellar furrow with 5 cross carinae; mesopleuron granulate; precoxal sulcus weakly scrobiculate or smooth, shorter than mesopleuron; venter granulate; propodeum with basal median areas margined, granulate, basal median carina absent, areola not margined, areolar area rugose, lateral areas entirely rugose. Wings: fore wing vein r shorter than vein 3RSa, vein 1cu-a beyond vein 1M; hind wing vein SC+R absent, vein M+CU slightly shorter than vein 1M. Metasoma: first tergum longitudinally costate, length greater than apical width; second tergum longitudinally costate; anterior transverse groove present, straight; posterior transverse groove present; third tergum costate basally, smooth apically; terga 4–7 smooth; ovipositor as long as metasomal terga 1 and 2 combined.

#### Holotype female.

Top label (white, printed) - Costa Rica: Puntarenas [;] San Vito, Estac. Biol. [;] Las Alturas, 2050m [;] ix-xi.1992, Paul Hanson [;] ex. Malaise trap; second label (red, partially printed and hand written) - HOLOTYPE [;] Heterospilus [;] lasalturus [;] P. Marsh. Deposited in ESUW.

#### Paratypes.

5 ♀♀, same data as holotype (ESUW). 5 ♀♀, top label - Costa Rica: Guanacaste [;] Santa Rosa Natl. Park [;] 300m, ex. Malaise trap [;] Site #: BH-12-C, SE-5-O and H-1-O [;] Dates: 19.xii.85–18.i.1986, 5–26.vii.1986, 7–28.xii.1985, 10–23.ix.1986 and 29.xi–20.xii.1986 [;] I.D. Gauld & D Janzen; second labels - [BH] Bosque Humedo [;] mature evergreen dry forest [;] [C] more or less fully [;] shaded as possible, [BH] Bosque Humedo [;] Mature evergreen dry forest [;] [O] in clearing, fully [;] isolated part of day, [SE] Bosque San Emilio [;] 50yr old deciduous forest [O] in clearing, fully [;] isolated part of day and [H] open regenerating [;] woodland <10 years old [;] [O] in clearing, fully isolated part of day (ESUW). 1 ♀, top label - Costa Rica: Guanacaste [;] Santa Rosa National Pk. [;] 300m, Malaise, Ian Gauld [;] 31.i–22.ii.1987; second label - Bosque Humedo [;] Mature dry forest [;] high proportion [;] Evergreen species [;] Sun (ESUW).

#### Comments.

The medium brown body color and the unsculptured area where the notauli meet are distinctive for this species.

#### Etymology.

Named for the Las Alturas Biological Station where most of the type series was collected.

**Figure 166.**
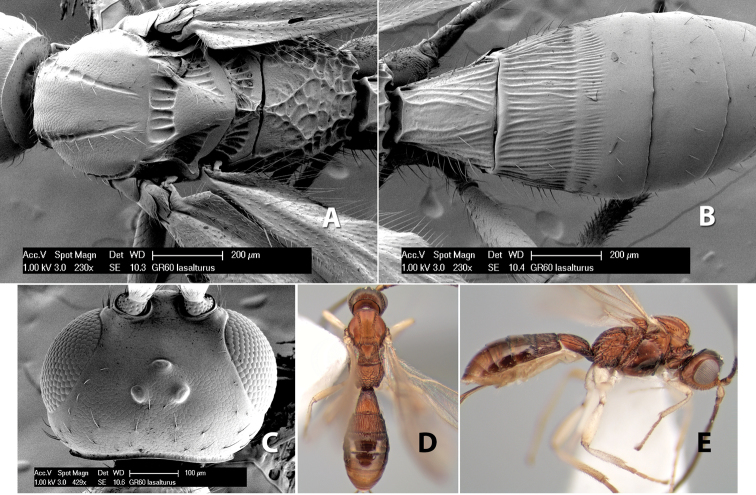
*Heterospilus lasalturus* Marsh, sp. n.: **A–C** paratype **D–E** holotype.

### Heterospilus leenderti

Marsh sp. n.

http://zoobank.org/AF85950E-8ADB-40B8-B946-8E2497897EE6

http://species-id.net/wiki/Heterospilus_leenderti

Figure 167

#### Female.

Body size: 3.5–4.0 mm. Color: head dark brown; scape yellow with lateral longitudinal brown stripe, flagellum brown with white apical annulus, apical 3–5 flagellomeres brown; mesosoma dark brown, mesoscutum and propodeum often lighter brown; metasomal terga dark brown, tergum 2 usually yellow medially, tergum 1 sometimes yellow apically, terga 4–7 rarely lighter brown; wing veins brown, stigma usually yellow, rarely bicolored light brown with yellow at apex or base or along anterior edge; legs yellow. Head: vertex granulate, weak transverse striae or rugae behind ocelli; frons granulate; face granulate-rugose; temple in dorsal view broad but sloping behind eye, width equal to 1/2 eye width; malar space equal to eye height; ocell-ocular distance about 2.5 times diameter of lateral ocellus; 25–30 flagellomeres. Mesosoma: mesoscutal lobes granulate, often weakly so; notauli scrobiculate, meeting at scutellum in triangular costate area; scutellum weakly granulate or smooth; prescutellar furrow with 5 cross carinae; mesopleuron granulate; precoxal sulcus smooth, shorter than mesopleuron; venter granulate; propodeum with basal median areas margined, granulate, basal median carina absent, areola not margined, areolar area areolate-rugose, lateral areas entirely rugose, propodeum with distinct tubercle above hind coxa at base of metasomal tergum 1. Wings: fore wing vein r slightly shorter or equal to length of vein 3RSa, often on same line as 3RSa, vein 1cu-a beyond vein 1M; hind wing vein SC+R absent, vein M+CU shorter than vein 1M. Metasoma: first tergum longitudinally costate, usually rugose medially, length equal to apical width; second tergum longitudinally costate; anterior transverse groove present, straight; posterior transverse groove present; third tergum entirely granulate except for posterior transverse groove, occasionally nearly smooth apically; terga 4–7 granulate; ovipositor equal to half length of metasomal tergum 1.

#### Holotype female.

Top label (white, printed) - COSTA RICA: Puntarenas [;] Rd. to Rincon, 10km W. [;] of Pan-Amer. Hwy, 100m [;] III-V 1989, Hanson & Gauld; second label (red, partially printed and hand written) - HOLOTYPE [;] Heterospilus [;] leenderti [;] P. Marsh. Deposited in ESUW.

#### Paratypes.

6 ♀♀, same data as holotype with additional date of II-III 1989 (ESUW). 6 ♀♀, top label - Costa Rica: Guanacaste [;] Santa Rosa Natl. Park [;] 300m, ex. Malaise trap [;] Site #: BH-10-C and BH-12-C [;] Dates: 4–24.v.1986, 5–26.vii.1986 and 8.ii–2.iii.1986 [;] I.D. Gauld & D. Janzen; second label - [BH] Bosque Humedo [;] mature evergreen dry forest [;] [C] more or less fully [;] shaded as possible (ESUW). 7 ♀♀, top label - Costa Rica: Guanacaste [;] Santa Rosa Natl. Park [;] 300m, ex. Malaise trap [;] Site #: BH-9-O, BH-11-O and 9 [;] Dates: 13.iv–4.v.1986, 4–24.v.1986 and 27.ix–18.x.1986 [;] I.D. Gauld & D. Janzen; second label - [BH] Bosque Humedo [;] mature evergreen dry forest [;] [O] in clearing, fully [;] isolated part of day (ESUW). 4 ♀♀, top label - Costa Rica: Guanacaste [;] Santa Rosa National Pk. [;] 300m, Malaise trap, Ian Gauld [;] 14.vi.1986 and 31.i–21.ii.1987; second label - Bosque Humedo [;] high proportion [;] Evergreen species [;] Sun; third label - BH-11-O [;] 14.Vi.86 and 31.i–21.ii.87 (ESUW). 10 ♀♀, COSTA RICA: Puntarenas [;] Reserva Forestal Golfo Dulce [;] 3km SW of Rincon, 10m [;] Mar-April 1992, November 1992 and July 1991, P. Hanson [;] primary forest, Malaise trap (ESUW). 1 ♀, Costa Rica: Puntarenas [;] R.F. Golfo Dulce, [;] 3km. SW. Rincon, 10m [;] iii.1993 Paul Hanson coll. [;] Malaise, primary forest (ESUW). 1 ♀, Costa Rica: Puntarenas [;] Res. Forestal Golfo Dulce [;] 3km SW Rincon, 10m [;] ii.1993, P. Hanson [;] Malaise, primary forest (ESUW). 4 ♀♀, Costa Rica: Puntarenas [;] R.F. Golfo Dulce, [;] 3km. SW. Rincon, 10m [;] xii.1992, vi.1991 and ii.1992, Paul Hanson (ESUW). 1 ♀, Costa Rica: Puntarenas [;] Golfo Dulce, 24km W. [;] Piedras Blancas, 200m [;] ii.1993, Paul Hanson (ESUW). 4 ♀♀, COSTA RICA: Puntarenas [;] R.F. Golfo Dulce [;] 24km W. Piedras Blancas, [;] 200m [;] Feb. 1992, Paul Hanson (ESUW). 1 ♀, Misc. Doryctinae [;] Costa Rica, Puntarenas [;] R.F. Golfo Dulce, 5km. W. [;] Piedras Blancas, 100m [;] IV-V-1993, P. Hanson (ESUW). 2 ♀♀, COSTA RICA: Puntar [;] Golfo Dulce, 24km W [;] Piedras Blancas [;] 200m, xii 89-iii 1990 [;] Col. Paul Hanson (ESUW). 1 ♀, COSTA RICA: Puntar [;] Golfo Dulce, 10km W [;] Piedras Blancas, 100m [;] VI-VIII 1989, Hanson (ESUW). 2 ♀♀, COSTA RICA: Puntar. [;] R.B. Carara, Estac. [;] Quebrada Bonita, 50m [;] V-VI 1989, P. Hanson (ESUW). 1 ♀, Costa Rica: Puntar. [;] P.N. Corcovado [;] Est. Sirena, 50m [;] x–xii 1990 (ESUW). 1 ♀, Costa Rica, Puntarenas [;] Pen. Osa, Puerto Jimenez [;] 10m, VI-1993, P. Hanson (ESUW). 1 ♀, Costa Rica: Heredia [;] 3km. S. Puerto Viejo, [;] OTS, La Selva, 100m [;] xi.1992, P. Hanson (ESUW). 1 ♀, Costa Rica: San Jose [;] Ciudad Colon [;] 800m, iv-v 1990 [;] Col. Luis Fournier (ESUW). 1 ♀, Costa Rica: San Jose [;] Cerro de la Muerte [;] 19km S 3 W Empalme [;] 2600m, November 1992 [;] P. Hanson, Malaise (ESUW). 1 ♀, Costa Rica: Alajuela ACA [;] San Carlos, R.F. Arenal [;] Sebdero Pilon, 600m, Malaise [;] 14.x–3.xii.1998, G. Carballo [;] L.N.269100-457900 #53365 (ESUW). 1 ♀, Costa Rica: Alajuela [;] 5km W San Ramon [;] 1200m, iv.1997 [;] O. Castro & P. Hanson (ESUW). 2 ♀♀, COSTA RICA: *Punt-* [;] *arenas*. 7km SW Rincon [;] 31.v–7.vi.1998; B. Brown [;] & V. Berezovskiy; Mal. [;] Trp. #5 and #1; 2nd growth (AEIC). 1 ♀, top label - COSTA RICA, Heredia: [;] Est. Biol. La Selva, 50- [;] 150m, 10°26'N, 84°01'W [;] Oct 1995, INBio-OET; second label - 16 Octubre 1995 [;] M/07/476 [;] Bosque primario (INBC). 2 ♀♀, COSTA RICA, Puntar. [;] Golfo Dulce, 24km W. [;] PiedrasBlancas. 200m [;] XII-89-III-90 Hanson (MICR).

#### Comments.

The yellow metasomal tergum 2, tubercles on the propodeum above the hind coxae and the fore wing vein r being nearly as or as long as vein 3RSa, are distinctive for this species.

#### Etymology.

Named for Leendert van der Ent, whose doctoral dissertation at the University of Wyoming on biodiversity of parasitic wasps in Costa Rica included a study on altitudinal diversity of Costa Rican *Heterospilus* with respect to antennal color.

**Figure 167.**
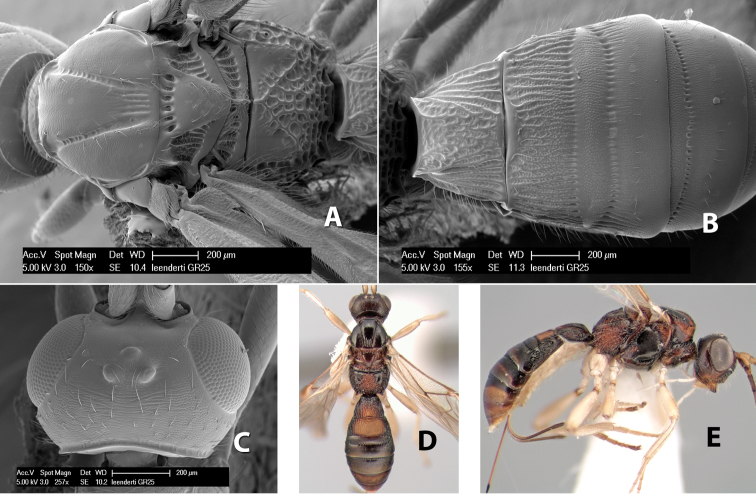
*Heterospilus leenderti* Marsh, sp. n.: **A–C, E** paratype **D** holotype.

### Heterospilus lenca

Marsh sp. n.

http://zoobank.org/DA7F874E-65EC-4F4E-9DEF-59DA856350E0

http://species-id.net/wiki/Heterospilus_lenca

Figure 168

#### Female.

Body size: 2.5 mm. Color: head and mesosoma dark brown, metasomal terga somewhat lighter brown; scape yellow with weak lateral longitudinal brown stripe, flagellum brown with apical white annulus, apical 3–5 flagellomeres brown; wing veins including stigma brown; legs yellow, apical half of femora usually darker brown. Head: vertex granulate; frons granulate; face granulate; temple in dorsal view narrow, sloping behind eye, width less than 1/2 eye width; malar space equal to 1/4 eye height; ocell-ocular distance at least 2.5 times diameter of lateral ocellus; 23 flagellomeres. Mesosoma: mesoscutal lobes weakly granular and partly smooth; notauli weakly scrobiculate anteriorly, smooth posteriorly, meeting at scutellum in unsculptured or partially weakly costate area; scutellum smooth; prescutellar furrow with 5 cross carinae; mesopleuron granulate; precoxal sulcus smooth, shorter than mesopleuron; venter granulate; propodeum with basal median areas margined, granulate, basal median carina absent, areola not margined, areolar area areolate-rugose, lateral areas rugose with small granulate patch anteriorly. Wings: fore wing vein r shorter than vein 3RSa, vein 1cu-a beyond vein 1M; hind wing vein SC+R absent, vein M+CU shorter than vein 1M. Metasoma: first tergum longitudinally costate, length very slightly greater than apical width; second tergum longitudinally costate; anterior transverse groove present, straight; posterior transverse groove present; third tergum costate anteriorly, granulate posteriorly; terga 4–7 weakly granulate; ovipositor as long as metasomal terga 1 and 2 combined.

#### Holotype female.

Top label (white, printed) - Costa Rica: Puntarenas [;] Res. Forestal Golfo Dulce [;] 3km. SW Rincon, 10m [;] iv.1993, P. Hanson [;] Malaise, primary forest; second label (red, partially printed and hand written) - HOLOTYPE [;] Heterospilus [;] lenca [;] P. Marsh. Deposited in ESUW.

#### Paratypes.

Known only from the holotype.

#### Comments.

The absence of hind wing vein SC+R, the granulate metasomal terga 3–6 and the shining but weakly granulate mesoscutum are distinctive for this species.

#### Etymology.

Named for the Lenca, an indigenous people of Honduras and El Salvador.

**Figure 168.**
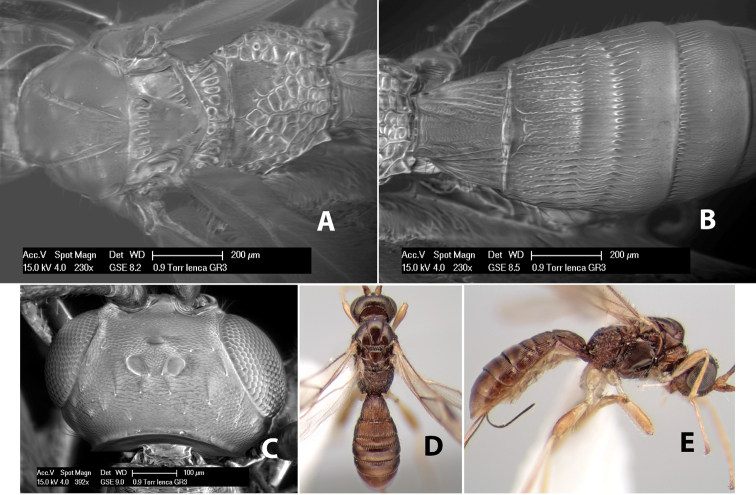
*Heterospilus lenca* Marsh, sp. n., holotype.

### Heterospilus longinoi

Marsh sp. n.

http://zoobank.org/5919EEA3-E942-4D8B-8370-E6C2B1DC2B89

http://species-id.net/wiki/Heterospilus_longinoi

Figure 169

#### Female.

Body size: 3.5 mm. Color: head with vertex and frons brown, face yellow; scape yellow with lateral longitudinal brown stripe, flagellum entirely brown; mesosoma dark brow; metasomal terga brown to dark brown, terga 6–7 yellow at apex; wing vein including stigma brown; legs yellow with apical half of hind femur, apical 1/4 of hind tibia and hind tarsus darker brown. Head: vertex granulate; frons granulate; face granulate; temple in dorsal view narrow, sloping behind eye, width less than 1/2 eye width; malar space greater than 1/4 eye height; ocell-ocular distance about 2.5 times diameter of lateral ocellus; 28 flagellomeres. Mesosoma: mesoscutal lobes granulate, median lobe somewhat depressed medially at junction of notauli; notauli scrobiculate, meeting at scutellum in triangular rugose area; scutellum granulate; prescutellar furrow with 3 cross carinae; mesopleuron granulate; precoxal sulcus scrobiculate, represented by a shallow round depression; venter granulate; propodeum with basal median areas not distinct, obscured by entire rugose propodeum, basal median carina absent, areola not indicated, areolar area rugose, lateral areas entirely rugose, propodeum with distinct tubercle above hind coxa. Wings: fore wing vein r shorter than vein 3RSa, vein 1cu-a beyond vein 1M; hind wing vein SC+R absent, vein M+CU shorter than vein 1M. Metasoma: first tergum longitudinally costate-granulate, raised median area distinctly margined on each side, length greater than apical width; second tergum longitudinally costate-granulate; anterior transverse groove present, straight; posterior transverse groove present; third tergum entirely granulate except for costate transverse groove; terga 4–7 granulate; ovipositor slightly shorter than length of metasomal tergum 1.

#### Holotype female.

Top label (white, printed) - COSTA RICA: La Selva [;] 1.XII.1983 [;] J. Longino [;] (M/13/287); second label (red, partially printed and hand written) - HOLOTYPE [;] Heterospilus [;] longinoi [;] P. Marsh. Deposited in ESUW.

#### Paratypes.

Known only from the holotype.

#### Comments.

The entirely rugose propodeum, deeply sculptured notauli and the granulate metasomal terga 3–6 are distinctive for this species.

#### Etymology.

Named for its collector, J. Longino.

**Figure 169.**
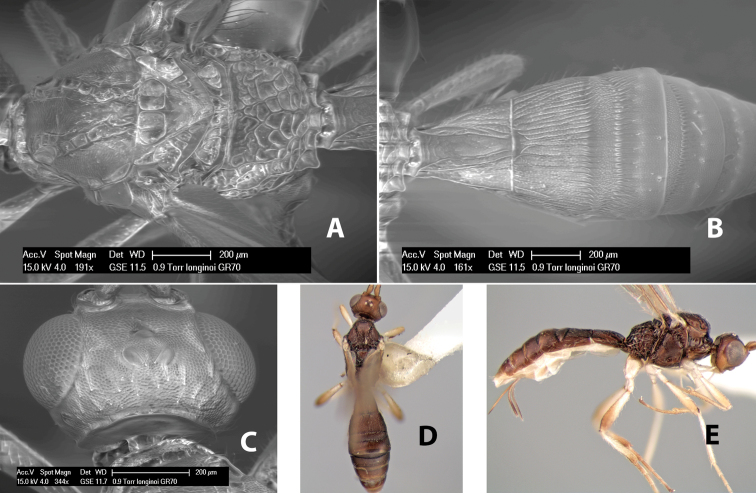
*Heterospilus longinoi* Marsh, sp. n., holotype.

### Heterospilus longisulcus

Marsh sp. n.

http://zoobank.org/4CFDD3AC-A01B-4ABC-A85E-959E3BFF02BB

http://species-id.net/wiki/Heterospilus_longisulcus

Figure 170

#### Female.

Body size: 2.5 mm. Color: body brown to dark brown; scape yellow without lateral brown stripe; flagellum brown; wing veins including stigma brown; legs yellow. Head: vertex granulate; frons granulate; face granulate; temple in dorsal view narrow, sloping behind eye, width less than 1/2 eye width; malar space greater than 1/4 eye height; ocell-ocular distance 2.0–2.5 times diameter of lateral ocellus; 21–23 flagellomeres. Mesosoma: mesoscutal lobes granulate; notauli scrobiculate, meeting at scutellum in triangular rugose area; scutellum granulate; prescutellar furrow with 5 cross carinae rarely with distinct median carina and lateral weaker carinae; mesopleuron granulate; precoxal sulcus scrobiculate, as long as mesopleuron; venter granulate; propodeum with basal median areas margined, granulate, basal median carina absent, areola not margined, areolar area rugose, lateral areas entirely rugose, propodeum with apical lateral corners weakly but distinctly pointed. Wings: fore wing vein r as long as vein 3RSa, vein 1cu-a beyond vein 1M; hind wing vein SC+R absent, vein M+CU shorter than vein 1M. Metasoma: first tergum longitudinally costate, length greater than apical width; second tergum longitudinally costate; anterior transverse groove present, straight; posterior transverse groove present but weak; third tergum costate basally, smooth apically; terga 4–7 smooth; ovipositor half as long as metasoma.

#### Holotype female.

Top label (white, printed) - Costa Rica: Puntarenas [;] San Vito, Las Cruces [;] Wilson Botanical Gardens [;] 18–22.iii.1990, 1150m [;] J.S. Noyes; second label (red, partially printed and hand written) - HOLOTYPE [;] Heterospilus [;] longisulcus [;] P. Marsh. Deposited in ESUW.

#### Paratypes.

1 ♀, same data as holotype (ESUW). 1 ♀, Costa Rica: Puntarenas [;] R.F. Golfo Dulce, [;] 3km. SW. Rincon, 10m [;] x-xii.1990, Paul Hanson (ESUW).

#### Comments.

The precoxal sulcus which is as long as the mesopleuron, the fore wing vein r which is as long as vein 3RSa and the pointed apical lateral corners of the propodeum are distinctive for this species.

#### Etymology.

The specific name refers to the precoxal sulcus that is as long as the mesopleuron.

**Figure 170.**
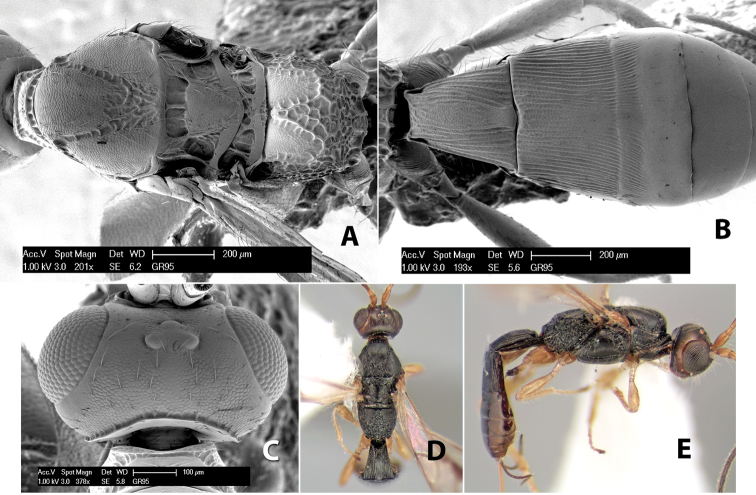
*Heterospilus longisulcus* Marsh, sp. n.: **A–E** paratype **D–E** holotype.

### Heterospilus mam

Marsh sp. n.

http://zoobank.org/BBE0F226-DC94-4EDF-BEC1-B6D8FB733147

http://species-id.net/wiki/Heterospilus_mam

Figure 171

#### Female.

Body size: 2.5 mm. Color: body dark brown, metasomal tergum 1 at apex, tergum 2 medially and terga 5–7 yellow; scape yellow with weak lateral longitudinal brown stripe, flagellum brown with apical white annulus, apical 1–2 flagellomeres brown; wing veins brown, stigma yellow; legs yellow. Head: vertex weakly granulate or partially smooth; frons weakly granulate or smooth; face smooth; temple in dorsal view narrow, sloping behind eye, width less than 1/2 eye width; malar space greater than 1/4 eye height; ocell-ocular distance greater than 2.5 times diameter of lateral ocellus; 19–22 flagellomeres. Mesosoma: mesoscutal lobes smooth; notauli smooth, meeting at scutellum in unsculptured area; scutellum smooth; prescutellar furrow with 1 cross carina, occasionally with weak carinae on each side; mesopleuron granulate; precoxal sulcus smooth, shorter than mesopleuron; venter smooth; propodeum with basal median areas margined, weakly granulate, basal median carina absent, areola not margined, areolar area rugose, lateral areas entirely rugose. Wings: fore wing vein r shorter than vein 3RSa, vein 1cu-a interstitial with vein 1M; hind wing vein SC+R absent, vein M+CU shorter than vein 1M. Metasoma: first tergum longitudinally costate, length equal to apical width; second tergum longitudinally costate; anterior transverse groove present, straight; posterior transverse groove present; third tergum smooth except for costate transverse groove; terga 4–7 smooth; ovipositor as long as metasomal 1.

#### Holotype female.

Top label (white, printed) - Costa Rica, Heredia [;] 3km. S. Puerto Viejo [;] OTS-La Selva. 100m [;] I-II-1993, P. Hanson; second label (red, partially printed and hand written) - HOLOTYPE [;] Heterospilus [;] mam [;] P. Marsh. Deposited in ESUW.

#### Paratypes.

1 ♀, Costa Rica: Puntarenas [;] R. F. Golfo Dulce, [;] 3km. SW. Rincon, 10m [;] ii.1992, Paul Hanson (ESUW).

#### Comments.

The smooth mesoscutal lobes, the short ovipositor and the white annulus at the apex of the flagellum are distinctive for this species.

#### Etymology.

Named for the Mam, a Mayan people of Guatemala.

**Figure 171.**
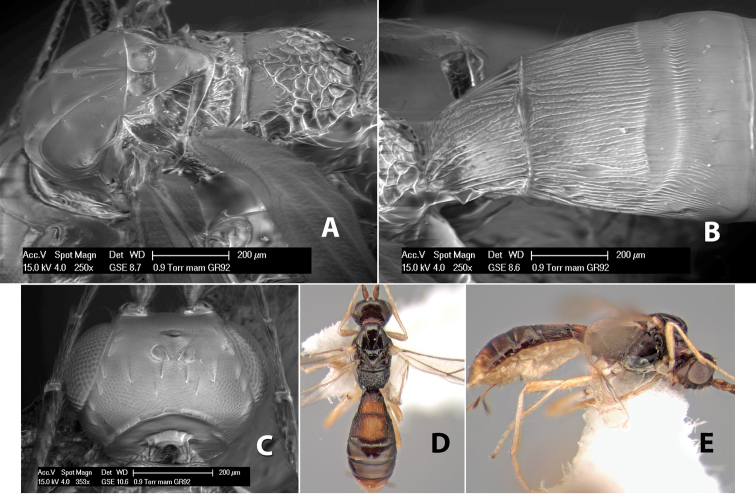
*Heterospilus mam* Marsh, sp. n., holotype.

### Heterospilus microstigmi

Richards

http://species-id.net/wiki/Heterospilus_microstigmi

Figure 172

Heterospilus microstigmi Richards, 1935: 131; Marsh and Melo 1999: 19.

#### Female.

Body size: 2.5–3.0 mm. Color: body yellow or honey yellow, mesoscutum, propodeum and metasomal terga 1–4 sometimes marked with brown; scape yellow without lateral brown stripe, flagellum yellow basally to brown apically; legs yellow. Head: vertex granulate; frons granulate; face granulate; temple in dorsal view narrow, sloping behind eye, width slightly less than 1/2 eye width; malar space greater than 1/4 eye height; ocell-ocular distance slightly greater than 2.5 times diameter of lateral ocellus; 24–28 flagellomeres. Mesosoma: mesoscutal lobes granulate, median lobe with shallow longitudinal depression; notauli scrobiculate, meeting at scutellum in small costate area; scutellum granulate; prescutellar furrow with 1 strong median cross carina and 2 weak carinae on each side; mesopleuron granulate; precoxal sulcus weakly scrobiculate or smooth; venter granulate; propodeum with basal median areas not distinctly margined, granulate-rugose, basal median carina absent, areola not indicated, areolar area rugose, lateral areas entirely rugose, propodeum with small but distinct tubercle above hind coxa. Wings: fore wing vein r slightly shorter than vein 3RSa, vein 1cu-a beyond vein 1M; hind wing vein SC+R absent, vein M+CU shorter than vein 1M. Metasoma: first tergum longitudinally costate, raised median area distinct, length slightly greater than apical width; second tergum longitudinally costate; anterior transverse groove present, straight; posterior transverse groove present; third tergum costate basally, weakly granulate apically; terga 4–7 weakly granulate; ovipositor about 1/2 length of metasoma.

#### Specimens examined.

2 ♀♀, Costa Rica, Puntarenas, Osa Peninsula; 1 ♀, Costa Rica, Puntarenas, Corcovado Nat. Pk. This species also occurs in Trinidad and Brazil.

#### Biology.

Reared from nests of *Microstigmus theridii* Ducke and *Microstigmus comes* Krombein (Hymenoptera: Sphecidae) (Marsh and Melo 1999).

#### Comments.

The yellow color of the body and the rugose propodeum are distinctive for this species.

**Figure 172.**
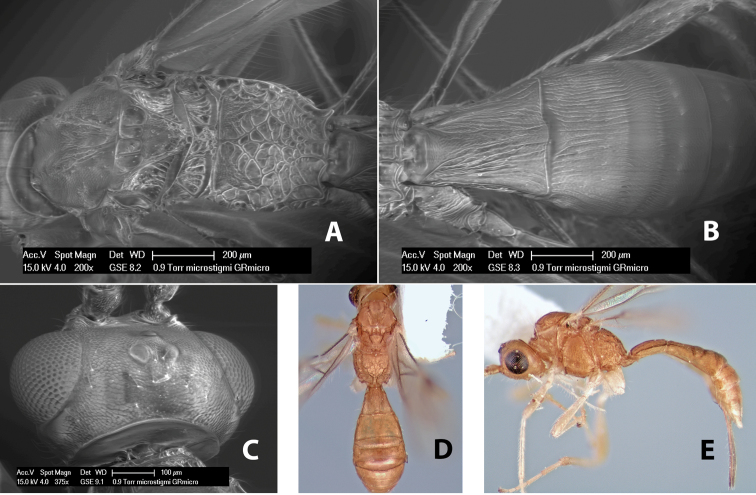
*Heterospilus microstigmi* Richards.

### Heterospilus mopanmaya

Marsh sp. n.

http://zoobank.org/040348F0-28F6-4B49-AAB8-8BB746453F58

http://species-id.net/wiki/Heterospilus_mopanmaya

Figure 173

#### Female.

Body size: 2.5 mm. Color: head brown, mesosoma and metasoma light brown; scape yellow with lateral longitudinal brown stripe, flagellum yellow at base to brown at apex with white apical annulus, apical 3–5 flagellomeres brown; wing veins including stigma light brown; legs yellow. Head: vertex granulate; frons granulate; face granulate; temple in dorsal view broad but not bulging behind eye, width equal to 1/2 eye width; malar space greater than 1/4 eye height; ocell-ocular distance slightly more than 2.5 times diameter of lateral ocellus; 21–23 flagellomeres. Mesosoma: mesoscutal lobes granulate; notauli scrobiculate, meeting at scutellum in triangular costate area; scutellum weakly granulate or smooth; prescutellar furrow with 5 cross carina; mesopleuron granulate; precoxal sulcus smooth, shorter than mesopleuron; venter granulate; propodeum with basal median areas not distinctly margined, granulate, basal median carina absent, areola not margined, areolar area areolate-rugose, lateral areas entirely rugose. Wings: fore wing vein r shorter than vein 3RSa, vein 1cu-a beyond vein 1M; hind wing vein SC+R absent, vein M+CU shorter than vein 1M. Metasoma: first tergum longitudinally costate, apical width equal to length; second tergum longitudinally costate; anterior transverse groove present, straight; posterior transverse groove present; third tergum costate basally, smooth apically; terga 4–7 smooth; ovipositor equal to length of metasomal terga 1 and 2 combined.

#### Holotype female.

Top label (white, partially printed and hand written) - Costa Rica: Guanacaste [;] Santa Rosa Natl. Park [;] 300m, ex. Malaise trap [;] Site #: BH-11-O [;] Dates: 8.ii–2.iii.1986 [;] I. D. Gauld & D. Janzen; second label (white, printed) - [BH] Bosque Humedo [;] mature evergreen dry forest [;] [O] in clearing, fully [;] isolated part of day; third label (red, partially printed and hand written) - HOLOTYPE [;] Heterospilus [;] mopanmaya [;] P. Marsh. Deposited in ESUW.

#### Paratypes.

1 ♀, top label - Costa Rica: Guanacaste [;] Santa Rosa Natl. Park [;] 300m, ex. Malaise trap [;] Site #: SE-8-C [;] Dates: 2–23.iii.1986 [;] I.D. Gauld & D. Janzen; second label - [SE] Bosque San Emilio [;] 50yr old deciduous forest [;] [C] more or less fully [;] shaded as possible (ESUW). 1 ♀, S.RosaPark, Guan. [;] C. Rica 17 Nov 77 [;] D.H. Janzen [;] Dry Hill (AEIC).

#### Comments.

The absence of hind wing vein SC+R, the smooth apical metasomal terga and the lateral brown stripe on the scape are distinctive for this species.

#### Etymology.

Named for the Mopan Maya, a Mayan people of Guatemala and Belize.

**Figure 173.**
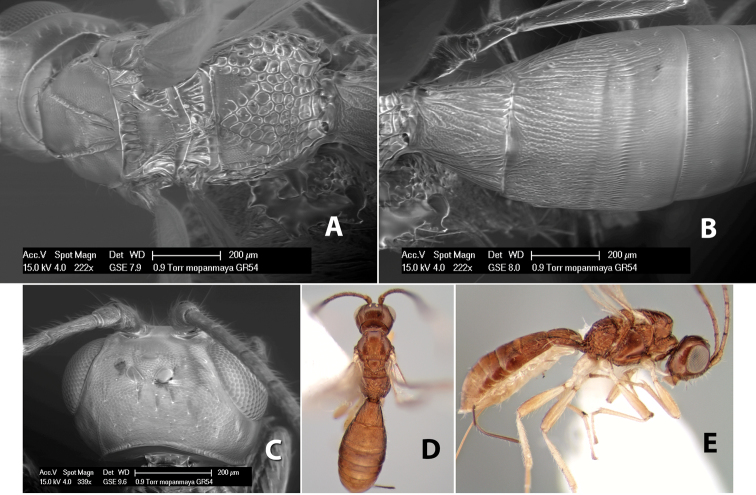
*Heterospilus mopanmaya* Marsh, sp. n., holotype.

### Heterospilus nahua

Marsh sp. n.

http://zoobank.org/65335184-3AD3-4D77-9264-A67E8A585B38

http://species-id.net/wiki/Heterospilus_nahua

Figure 174

#### Female.

Body size: 2.0 mm. Color: head brown, face honey yellow; scape yellow without lateral brown stripe, flagellum brown with apical 3–5 flagellomeres white; mesosoma brown; metasoma brown, terga 1 and 2 medially lighter brown; wing veins including stigma brown; legs yellow, hind femora brown on apical 3/4. Head: vertex very weakly granulate, appearing smooth at lower magnifications; frons smooth; face smooth; temple in dorsal view narrow, sloping behind eye, width less than 1/2 eye width; malar space greater than 1/4 eye height; ocell-ocular distance greater than 2.5 times diameter of lateral ocellus; 16 flagellomeres. Mesosoma: mesoscutal lobes weakly granulate or smooth; notauli weakly scrobiculate or smooth, meeting at scutellum in unsculptured area; scutellum smooth; prescutellar furrow with 1 cross carina; mesopleuron granulate; precoxal sulcus smooth, shorter than mesopleuron; venter granulate; propodeum with basal median areas margined, granulate, basal median carina absent, areola weakly margined, areolar area rugose, lateral areas entirely rugose. Wings: fore wing vein r shorter than vein 3RSa, vein 1cu-a interstitial with vein 1M; hind wing vein SC+R absent, vein M+CU shorter than vein 1M. Metasoma: first tergum longitudinally costate-granulate, length nearly twice apical width; second tergum longitudinally costate-granulate; anterior transverse groove present, straight; posterior transverse groove present; third tergum smooth; terga 4–7 smooth; ovipositor shorter than metasomal tergum 1.

#### Holotype female.

Top label (white, printed) - Costa Rica: Limon, ACLAC [;] Central Res. Biol. Hitoy Cerere [;] Est. Hitoy Cerere, Send. Espavel [;] 300m, 17.iv–17.v.1999, F. Umana [;] L.S. 401500-570200 #52777 Mal.; second label (red, partially printed and hand written) - HOLOTYPE [;] Heterospilus [;] nahua [;] P. Marsh. Deposited in ESUW.

#### Paratypes.

1 ♀, Costa Rica: Puntarenas [;] ACO, Golfito, R.F. Golfo Dulce [;] Est. Agujas, 250–300m, Malaise [;] 15.ix–10.x.1999, J. Azofeifa [;] L.S. 276750-526550 #53486 (ESUW). 1 ♀, Sirena, Osa Pen. [;] VII, 77 Cos. Rica [;] D. H. Janzen (AEIC). 2 ♀♀, Costa Rica: Heredia [;] Braulio Carrillo N.P. [;] 250–500m IV.10.85 [;] Henri Goulet (AEIC). 1 ♀, Costa Rica: Heredia [;] Braulio Carrillo N.P. [;] 400m IV.10–11.85 [;] Henri Goulet (AEIC). 1 ♀, top label - COSTA RICA, Heredia: [;] Est. Biol. La Selva, 50- [;] 150m, 10°26'N, 84°01'W [;] Jan 1996, INBio-OET; second label - 02 Enero 1996 [;] M/04/534 [;] Bosque primario (INBC). 2 ♀♀, top label - COSTA RICA, Heredia: [;] Est. Biol. La Selva, 50- [;] 150m, 10°26'N, 84°01'W [;] Mar 1996, INBio-OET; second label - 15 Marzo 1996 [;] Bosque primario [;] M/03/593 (INBC). 3 ♀♀, COSTA RICA, Puntarenas [;] San Vito, Jardin Bot. [;] Las Cruces, VI-VII/98 and XII/1988 [;] 1200m, Col. P Hanson (MICR). 2 ♀♀, COSTA RICA, SanJosé [;] P. N. Braulio Carillo [;] 9.5km E tunel, 1000m [;] VI/1989 and V-VI-90, col. P. Hanson (MICR).

#### Comments.

The very short ovipositor, the unsculptured area where the notauli meet and the very weakly granulate vertex are distinctive for this species.

#### Etymology.

Named for the Nahua, an indigenous people of Mexico.

**Figure 174.**
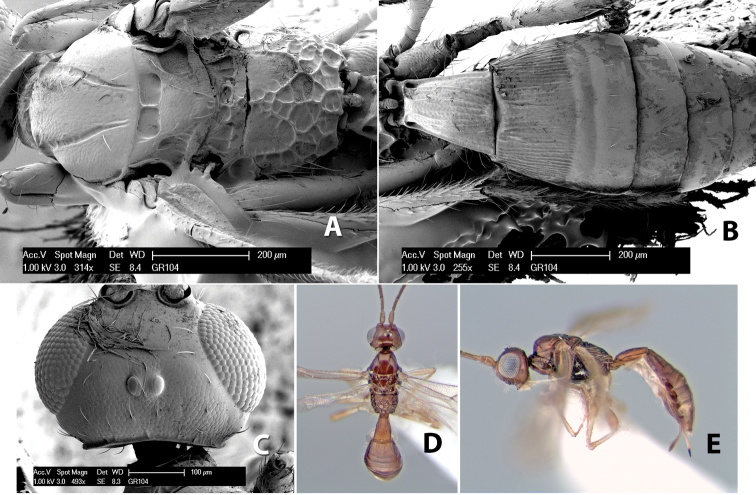
*Heterospilus nahua* Marsh, sp. n.: **A–C** paratype **D–E** holotype.

### Heterospilus nemestrinus

Marsh sp. n.

http://zoobank.org/251ECEEC-5A4C-490D-8788-EF3C8CEB931C

http://species-id.net/wiki/Heterospilus_nemestrinus

Figure 175

#### Female.

Body size: 3.0 mm. Color: head with vertex dark brown, face and eye orbits yellow; mesosoma dark brown; metasoma dark brown, tergum 7 yellow; scape yellow with weak lateral longitudinal brown stripe, flagellum brown; wing veins including stigma brown; legs yellow. Head: vertex granulate with transverse rugae behind ocelli; frons granulate; face rugose; temple in dorsal view narrow, sloping behind eye, width less than 1/2 eye width; malar space equal to 1/4 eye height; ocell-ocular distance about 1.5 times diameter of lateral ocellus; 20 flagellomeres. Mesosoma: mesoscutal lobes granulate, somewhat rugose along notauli; notauli scrobiculate, meeting at scutellum in broad rugose area; scutellum granulate; prescutellar furrow with 5 cross carinae; mesopleuron granulate; precoxal sulcus scrobiculate, shorter than mesopleuron; venter granulate; propodeum with basal median areas not distinct, rugose, basal median carina absent, areola not distinct, areolar area areolate-rugose, lateral areas entirely rugose. Wings: fore wing vein r shorter than vein 3RSa, vein 1cu-a beyond vein 1M; hind wing vein SC+R present, vein M+CU as long as vein 1M. Metasoma: first tergum longitudinally costate, length equal to apical width; second tergum longitudinally costate; anterior transverse groove present, straight; posterior transverse groove present; third tergum costate basally, smooth apically; terga 4–7 smooth; ovipositor half as long as metasoma.

#### Holotype female.

Top label (white, printed) - COSTA RICA-Heredia [;] La Selva Biological Station [;] 10°26'N, 84°01'W, 100m [;] Canopy fogging 19 [;] 8.x.1994 [;] Project ALAS(FVK19); second label (red, partially printed and hand written) - HOLOTYPE [;] Heterospilus [;] nemestrinus [;] P. Marsh. Deposited in ESUW.

#### Paratypes.

Known only from the holotype.

#### Comments.

The rugose propodeum and mesoscutum are distinctive for this species.

#### Etymology.

Named for Nemestrinus, the Roman god of forests, woods and groves.

**Figure 175.**
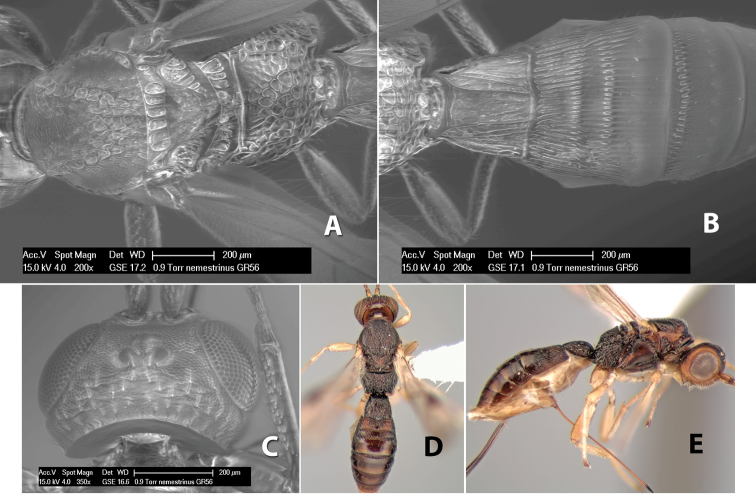
*Heterospilus nemestrinus* Marsh, sp. n., holotype.

### Heterospilus orosi

Marsh sp. n.

http://zoobank.org/B8D2E170-B49C-4062-B4E8-BA6EF4BD156E

http://species-id.net/wiki/Heterospilus_orosi

Figure 176

#### Female.

Body size: 2.0–2.5 mm. Color: head bicolored, vertex dark brown, face honey yellow; scape yellow with lateral longitudinal brown stripe, flagellum brown with apical white annulus, apical 3–5 flagellomeres brown; mesosoma honey yellow, mesopleuron and mesoscutal lobes dark brown; metasomal dark brown, terga 1 and 2 often honey yellow, apical terga sometimes lighter; wing veins including stigma brown; legs yellow. Head: vertex granulate; frons granulate; face granulate; temple in dorsal view narrow, sloping behind eye, width equal to 1/2 eye width; malar space equal to 1/4 eye height; ocell-ocular distance about 2.5 times diameter of lateral ocellus; 19–22 flagellomeres. Mesosoma: mesoscutal lobes granulate; notauli scrobiculate, meeting at scutellum in short costate area; scutellum granulate; prescutellar furrow with 3 cross carinae; mesopleuron granulate; precoxal sulcus weakly scrobiculate or smooth; venter granulate; propodeum with basal median areas margined, sometimes weakly or indistinctly so, areas granulate, basal median carina absent, areola usually indistinct, occasionally distinctly margined, areolar area areolate, lateral areas entirely rugose. Wings: fore wing vein r shorter than vein 3RSa, vein 1cu-a beyond vein 1M; hind wing vein SC+R present, vein M+CU shorter than vein 1M. Metasoma: first tergum longitudinally costate, often rugose medially, length equal to apical width; second tergum longitudinally costate; anterior transverse groove present, straight; posterior transverse groove present; third tergum costate basally, smooth apically; terga 4–7 smooth; ovipositor as long as metasomal tergum 1.

#### Holotype female.

Top label (white, printed) - Costa Rica: Guanacaste [;] Est. Biol. Maritza, 600m [;] xi.1996, C. Zuniga, Malaise [;] L.N. 326900-373000 #47554; second label (red, partially printed and hand written) - HOLOTYPE [;] Heterospilus [;] orosi [;] P. Marsh. Deposited in ESUW.

#### Paratypes.

6 ♀♀, top label - Costa Rica: Guanacaste [;] W. side Volcan Orosi [;] Estac. Maritza, 600m; second label - GNP Biodiversity Survey [;] 1989, Malaise trap [;] L-N-326900-373000 #6834 (ESUW). 3 ♀♀, Costa Rica: Guanacaste [;] Est. Cacao, 1000–1150m [;] viii.1996, M. Pereira [;] L.N. 323150-375500 347561 [;] Malaise trap (ESUW). 2 ♀♀, Costa Rica: Puntarenas [;] Est. Cacao, 1000–1400m [;] 2km. SW del Cerro Cacao [;] vii.1996, J. A. Ugalde [;] L.N. 323100-375800 #8220 [;] Malaise trap (ESUW). 2 ♀♀, Costa Rica: Guanacaste [;] Est. Cacao, 1000–1150m [;] ix.1996, I. Villegas, Malaise [;] L.N. 323150-375500 #47559 (ESUW). 1 ♀, COSTA RICA: Puntar [;] R.B. Carara, Estac. [;] Bijagoal, 500m [;] X 1989, P. Hanson (ESUW).

#### Comments.

The bicolored dark brown to honey yellow body is distinctive for this species.

#### Etymology.

Name for the Volcano Orosi where many of the type series were collected at the Maritza Biological Station.

**Figure 176.**
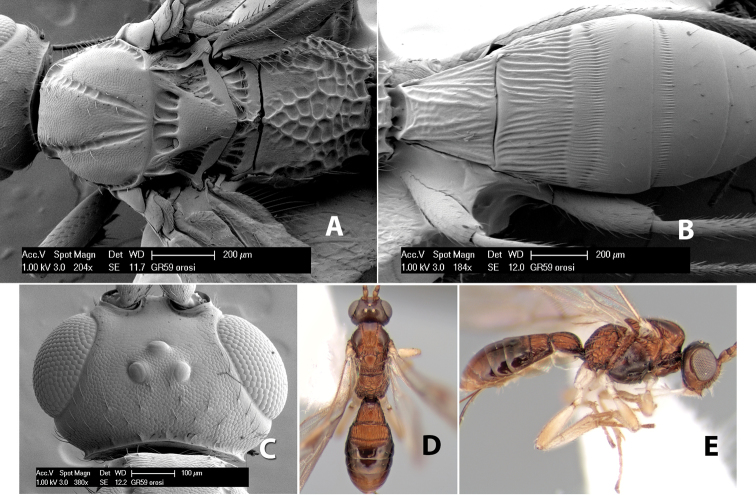
*Heterospilus orosi* Marsh, sp. n.: **A–C** paratype **D–E** holotype.

### Heterospilus parkeri

Marsh sp. n.

http://zoobank.org/61EC2F6C-06C1-4C8A-B821-3A58E6A3C4B6

http://species-id.net/wiki/Heterospilus_parkeri

Figure 177

#### Female.

Body size: 3.0 mm. Color: body brown to dark brown; scape yellow with lateral longitudinal brown stripe, flagellum brown with apical 8–10 flagellomeres white, apical one often darker; wing veins including stigma brown; legs yellow, coxae and trochanters whitish-yellow, hind femur light brown on apical 3/4. Head: vertex granulate; frons granulate; face granulate; temple in dorsal view narrow, sloping behind eye, width less than 1/2 eye width; malar space equal to 1/4 eye height; ocell-ocular distance about twice diameter of lateral ocellus; 23–24 flagellomeres. Mesosoma: mesoscutal lobes granulate; notauli scrobiculate, meeting at scutellum in triangular costate area; scutellum weakly granulate or smooth; prescutellar furrow with 1 cross carina; mesopleuron granulate; precoxal sulcus smooth, shorter than mesopleuron; venter granulate; propodeum with basal median areas distinct but not distinctly margined, granulate, basal median carina absent, areola not margined, areolar area rugose, often with median longitudinal carina, lateral areas rugose posteriorly, granulate anteriorly. Wings: fore wing vein r shorter than vein 3RSa, vein 1cu-a beyond vein 1M; hind wing vein SC+R absent, vein M+CU shorter than vein 1M. Metasoma: first tergum longitudinally costate, length equal to apical width; second tergum longitudinally costate; anterior transverse groove present, straight; posterior transverse groove present; third tergum weakly costate basally, smooth apically; terga 4–7 smooth; ovipositor as long as metasomal tergum 1.

#### Holotype female.

Top label (white, printed) - COSTA RICA-Heredia Prov. [;] La Selva Biological Station [;] 10°26'N, 84°01'W, 100m [;] Malaise trap 06, #291 [;] 2.xii.1993 [;] Project ALAS(M.06.291); second label (red, partially printed and hand written) - HOLOTYPE [;] Heterospilus [;] parkeri [;] P. Marsh. Deposited in ESUW.

#### Paratypes.

1 ♀, same data as holotype with Malaise trap 08, #272 and date of 15.xi.1993 (ESUW). 1 ♀, Costa Rica: Puntarenas [;] ACO, Golfito, PN Corcovado [;] Est. Agujas, Charcos, 600m [;] 17.iv–16.v.1999, J. Azofeifa [;] L.S. 276350-523500 #52776 (ESUW).

#### Comments.

The absence of hind wing vein SC+R, the smooth apical metasomal terga, the short ovipositor and the indistinctly margined basal median areas of the propodeum are distinctive for this species.

#### Etymology.

Named for Frank Parker, in remembrance of our days as graduate students at U. C. Davis and in recognition of the many braconids he collected in Costa Rica.

**Figure 177.**
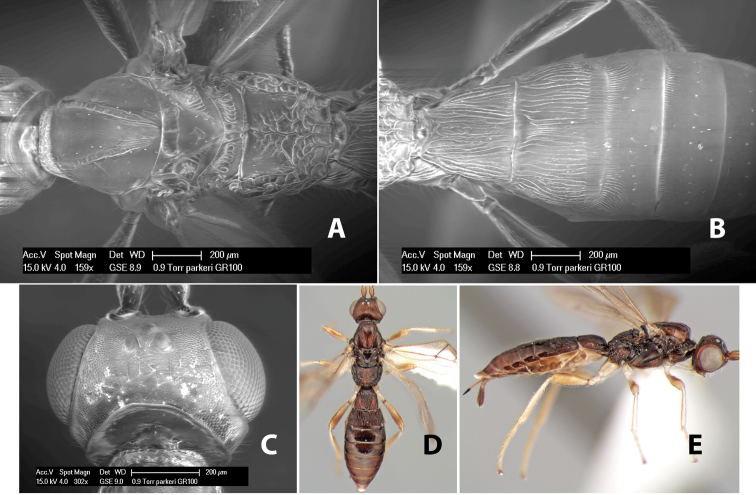
*Heterospilus parkeri* Marsh, sp. n., holotype.

### Heterospilus pech

Marsh sp. n.

http://zoobank.org/70F36A46-B635-46F2-9ADF-93D77E8054F3

http://species-id.net/wiki/Heterospilus_pech

Figure 178

#### Female.

Body size: 3.5 mm. Color: head and mesosoma dark brown; scape yellow with lateral longitudinal brown stripe, flagellum brown with white annulus, apical 3–5 flagellomeres brown; metasomal tergum 1 dark brown on basal half, yellow on apical half, tergum 2 yellow medially, dark brown laterally, tergum 3 yellow basally, dark brown laterally and light brown apically, terga 4–7 light brown; wing veins brown, stigma yellow; legs yellow. Head: vertex granulate; frons granulate; face rugose; temple in dorsal view broad but not sloping behind eye, width less than 1/2 eye width; malar space equal to 1/4 eye height; ocell-ocular distance slightly greater than 2.5 times diameter of lateral ocellus; 28 flagellomeres. Mesosoma: mesoscutal lobes granulate; notauli scrobiculate, meeting at scutellum in triangular costate area; scutellum granulate; prescutellar furrow with 5 cross carinae; mesopleuron granulate; precoxal sulcus smooth, shorter than mesopleuron; venter granulate; propodeum with basal median areas not distinctly margined, granulate, basal median carina absent, areola not distinct, areolar area areolate, lateral areas entirely rugose, propodeum with weak but distinct tubercle above hind coxa at base of petiole. Wings: fore wing vein r shorter than vein 3RSa, vein 1cu-a beyond vein 1M; hind wing vein SC+R present, vein M+CU shorter than vein 1M. Metasoma: first tergum longitudinally costate, apical width equal to length; second tergum longitudinally costate; anterior transverse groove present, straight; posterior transverse groove present; third tergum costate basally, very weakly granulate or appearing smooth apically; terga 4–7 very weakly granulate or appearing smooth; ovipositor equal to length of metasomal terga 1 and 2 combined.

#### Holotype female.

Top label (white, partially printed and hand written) - Costa Rica: Guanacaste [;] Santa Rosa Natl. Park [;] 300m, ex. Malaise trap [;] Site #: H-1-O [;] Dates: 14.viii–6.ix.1986 [;] I.D. Gauld & D. Janzen; second label (white, printed) - [H] open regenerating [;] woodland 10 years old [;] [O] in clearing, fully [;] isolated part of day; third label (red, partially printed and hand written) - HOLOTYPE [;] Heterospilus [;] pech [;] P. Marsh. Deposited in ESUW.

#### Paratypes.

Known only from the holotype.

#### Comments.

The yellow stigma, yellow basal metasomal terga and rugose face are distinctive for this species.

#### Etymology.

Named for the Pech, an indigenous people of Honduras.

**Figure 178.**
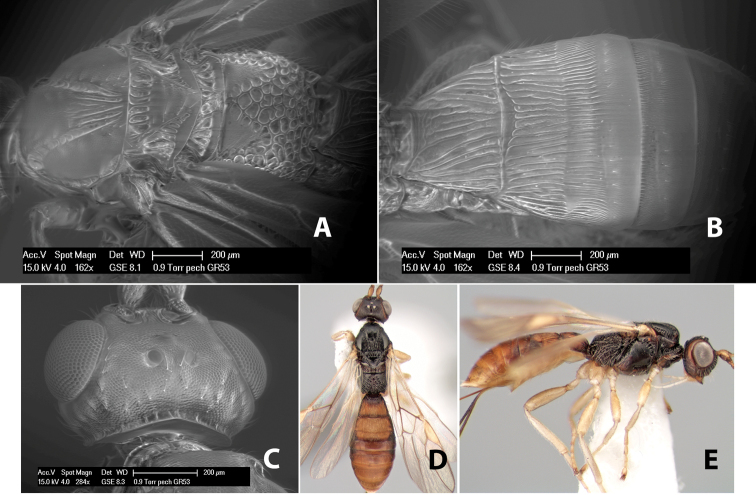
*Heterospilus pech* Marsh, sp. n., holotype.

### Heterospilus phaeocoxus

Marsh sp. n.

http://zoobank.org/2BC2062B-0682-4881-8DF3-93A2A8C5F381

http://species-id.net/wiki/Heterospilus_phaeocoxus

Figure 179

#### Female.

Body size: 3.5 mm. Color: body dark brown, apical metasomal terga yellow; scape dark brown, flagellum brown with apical 9 flagellomeres white, apical flagellomere partially brown; wing veins including stigma brown; legs bicolored yellow and brown, fore and mid coxae and trochanters light yellow, femora brown with yellow at extreme base, tibiae and tarsi brown, hind coxa brown, trochanters and base of femur yellow, rest of femur, tibia and tarsus brown. Head: vertex weakly granulate; frons weakly granulate; face weakly granulate-striate; temple in dorsal view narrow, sloping behind eye, width less than 1/2 eye width; malar space greater than 1/4 eye height; ocell-ocular distance about 2.5 times diameter of lateral ocellus; 27 flagellomeres. Mesosoma: mesoscutal lobes granulate; notauli weakly scrobiculate, meeting at scutellum in triangular costate area; scutellum granulate; prescutellar furrow with 1 cross carinae; mesopleuron granulate; precoxal sulcus smooth, shorter than mesopleuron; venter granulate; propodeum with basal median areas margined, granulate, basal median carina absent, areola not margined, areolar area rugose, lateral areas rugose posteriorly, granulate anteriorly, propodeum with distinct tubercle just above hind coxa. Wings: fore wing vein r shorter than vein 3RSa, vein 1cu-a beyond vein 1M; hind wing vein SC+R present, vein M+CU equal to length of vein 1M. Metasoma: first tergum longitudinally costate, length greater than apical width; second tergum longitudinally costate; anterior transverse groove present, straight; posterior transverse groove present; third tergum smooth except for costate posterior transverse groove; terga 4–7 smooth; ovipositor equal to length of metasomal terga 1 and 2 combined.

#### Holotype female.

Top label (white, printed) - Costa Rica: Alajuela, ACA [;] San Carlos, R.F. Arenal, 600m [;] Send. Pilon, Malaise trap [;]\ 26.x–22.xi.1999, G. Carballo [;] L.N. 269100-457900 #54376; second label (red, partially printed and hand written) - HOLOTYPE [;] Heterospilus [;] phaeocoxus [;] P. Marsh. Deposited in ESUW.

#### Paratypes.

Known only from the holotype.

#### Comments.

The distinctly bicolored brown and yellow legs and the single cross carina in the prescutellar furrow are distinctive for this species.

#### Etymology.

The specific name is from the Greek *phaios*, meaning brown, in reference to the brown hind coxa.

**Figure 179.**
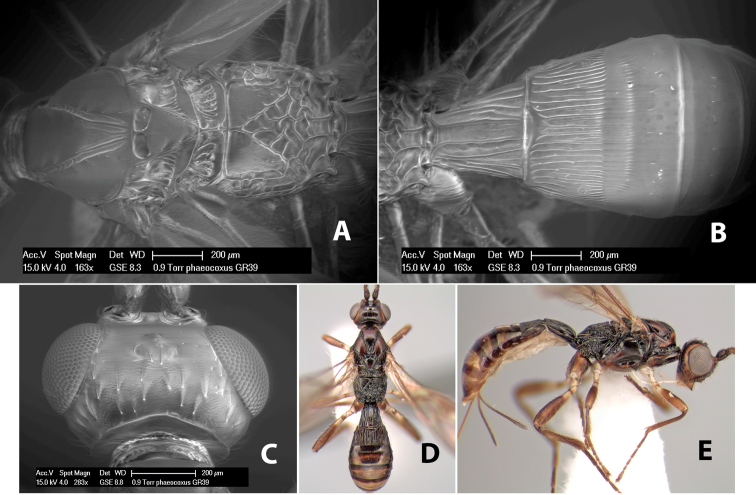
*Heterospilus phaeocoxus* Marsh, sp. n., holotype.

### Heterospilus phytorius

Marsh sp. n.

http://zoobank.org/8263D6B3-2FFE-40C4-B8D8-2A04FF941EF4

http://species-id.net/wiki/Heterospilus_phytorius

Figure 180

#### Female.

Body size: mm. Color: body dark brown, metasomal tergum 1 apically and 2 medially yellow; scape yellow with lateral longitudinal brown stripe, flagellum brown with apical white annulus, apical 5–7 flagellomeres brown; wing veins including stigma light brown; legs yellow. Head: vertex granulate; frons granulate; face granulate; temple in dorsal view broad but sloping behind eye, width equal to 1/2 eye width; malar space greater than 1/4 eye height; ocell-ocular distance about 2.5 times diameter of lateral ocellus; 25–26 flagellomeres. Mesosoma: mesoscutal lobes granulate; notauli scrobiculate, meeting at scutellum in triangular costate area; scutellum granulate; prescutellar furrow with 3–5 cross carinae; mesopleuron granulate; precoxal sulcus smooth, shorter than mesopleuron; venter granulate; propodeum with basal median areas not distinctly margined, granulate, basal median carina absent, areola not margined, areolar area areolate-rugose, lateral areas entirely rugose, propodeum with weak but distinct tubercle above hind coxa at base of petiole. Wings: fore wing vein r shorter than vein 3RSa, vein 1cu-a beyond vein 1M; hind wing vein SC+R absent, vein M+CU shorter than vein 1M. Metasoma: first tergum longitudinally costate, length equal to apical width; second tergum longitudinally costate; anterior transverse groove present, straight; posterior transverse groove present; third tergum costate basally, granulate apically; terga 4–7 granulate; ovipositor equal to length of metasomal terga 1 and 2 combined.

#### Holotype female.

Top label (white, printed) - Costa Rica: Puntarenas [;] San Vito, Las Cruces [;] Wilson Botanical Gardens [;] 18–22.iii.1990, 1150m [;] J.S. Noyes; second label (red, partially printed and hand written) - HOLOTYPE [;] Heterospilus [;] phytorius [;] P. Marsh. Deposited in ESUW.

#### Paratypes.

3 ♀♀, same data as holotype (ESUW). 1 ♀, Costa Rica: Puntarenas [;] Golfo Dulce, 24km W. [;] Piedras Blancas, 200m [;] ii.1993, Paul Hanson (ESUW). 1 ♀, Sirena, Osa Pen. [;] VII. 77 Cos. Rica [;] D. H. Janzen (AEIC). 1 ♀, COSTA RICA: *Punt-* [;] *arenas*, 7km SW Rincon [;] 31.v–7.vi.1998; B. Brown [;] & V. Berezovskiy; Mal. [;] Trp. #5; 2nd growth (AEIC).

#### Comments.

The absence of hind wing vein SC+R, the granulate metasomal terga 4–7 and the small but distinct tubercle on the propodeum above the hind coxa are distinctive for this species.

#### Etymology.

The specific name is from the Greek *pyhtorion*, meaning nursery, in reference to the type series being collected in the Wilson Botanical Gardens in Puntarenas Province.

**Figure 180.**
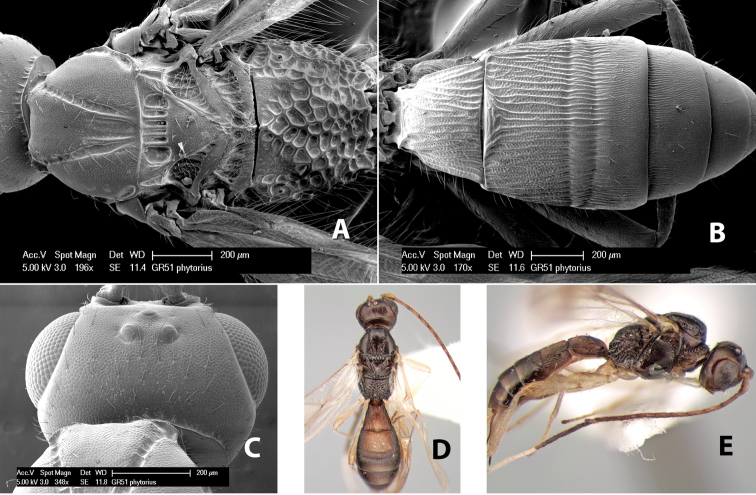
*Heterospilus phytorius* Marsh, n. s.: **A–C** paratype **D–E** holotype.

### Heterospilus pitillaensis

Marsh sp. n.

http://zoobank.org/1D7900F4-CCC1-419E-8C4A-9111D524C241

http://species-id.net/wiki/Heterospilus_pitillaensis

Figure 181

#### Female.

Body size: 2.0–2.5 mm. Color: body light to medium brown; scape yellow without lateral brown stripe, flagellum brown with apical white annulus, apical 3–5 flagellomeres brown; wing veins light brown, stigma yellow; legs yellow. Head: vertex weakly granulate or smooth; frons weakly granulate or smooth; face weakly granulate or partially smooth; temple in dorsal view broad but sloping behind eye, width equal to 1/2 eye width; malar space greater than 1/4 eye height; ocell-ocular distance greater than 2.5 times diameter of lateral ocellus; 22–23 flagellomeres. Mesosoma: mesoscutal lobes weakly granulate, often smooth near prescutellar furrow; notauli smooth or weakly scrobiculate anteriorly, meeting at scutellum in weak costate or often smooth area; scutellum smooth; prescutellar furrow with 3–5 cross carinae; mesopleuron granulate; precoxal sulcus smooth, shorter than mesopleuron; venter smooth; propodeum with basal median areas distinctly margined, granulate, basal median carina absent or rarely very short, areola indistinctly margined, areolar area areolate-rugose, lateral areas entirely rugose. Wings: fore wing vein r shorter than vein 3RSa, vein 1cu-a interstitial with vein 1M; hind wing vein SC+R present, vein M+CU shorter than vein 1M. Metasoma: first tergum longitudinally costate, length equal to or sometimes slightly greater than apical width; second tergum longitudinally costate; anterior transverse groove present, straight; posterior transverse groove present; third tergum costate basally, smooth apically; terga 4–7 smooth rarely weakly granulate at extreme base; ovipositor half as long as metasoma.

#### Holotype female.

Top label (white, printed) - COSTA RICA: [;] Guanacaste Province [;] P. N. Guanacaste [;] below Pitilla, 500m [;] 7–8.iii.1990, J. S. Noyes; second label (red, partially printed and hand written) - HOLOTYPE [;] Heterospilus [;] pitillaensis [;] P. Marsh. Deposited in ESUW.

#### Paratypes.

3 ♀♀, same data as holotype (ESUW). 1 ♀, Costa Rica: Puntarenas [;] San Vito, Las Cruces [;] Wilson Botanical Gardens [;] 18–22.iii.1990, 1150m [;] J.S. Noyes (ESUW). 1 ♀, first label, Costa Rica: Guanacaste [;] Santa Rosa Natl. Park [;] 300m, ex. Malaise trap [;] Site #: H-3-O [;] Dates: 26.vii–14.viii.1986 [;] I.D. Gauld & D. Janzen; second label, [H] open regenerating [;] woodland <10 years old [;] [O] in clearing, fully [;] isolated part of day (ESUW). 2 ♀♀, Bataan, C.R. [;] IV-24–1959 [;] RDShenefelt [;] RDS 57–1959 (AEIC).

#### Comments.

The weakly granulate head and mesosoma and the light brown color of the body are distinctive for this species.

#### Etymology.

Named for the type locality, near the Pitilla (Pitiya) Biological Station in the Guanacaste National Park.

**Figure 181.**
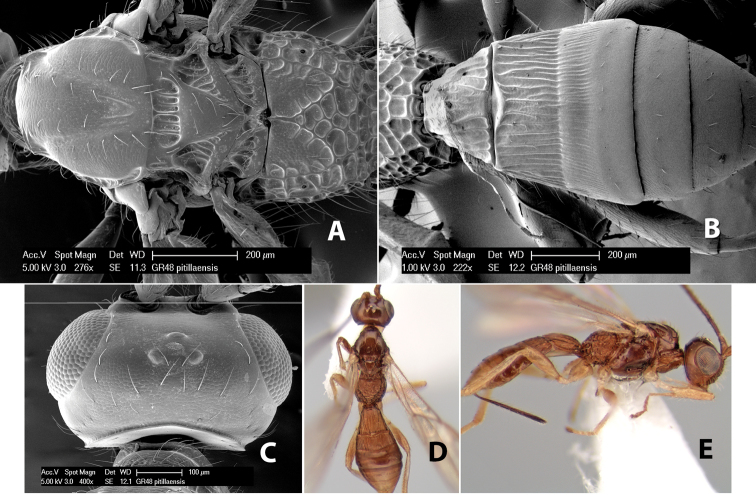
*Heterospilus pitillaensis* Marsh, sp. n.: **A–C** paratype **D–E** holotype.

### Heterospilus poqomam

Marsh sp. n.

http://zoobank.org/CE25DA84-231D-4A64-8B7E-EA1CF49855F5

http://species-id.net/wiki/Heterospilus_poqomam

Figure 182

#### Female.

Body size: 2.5 mm. Color: head with vertex and frons brown, face yellow; scape yellow without lateral brown stripe, flagellum brown with apical 3–5 flagellomeres white, apical most flagellomere sometimes brown, basal 3–4 flagellomeres honey yellow; mesosoma and metasoma dark brown, apical terga lighter brown; wing veins including stigma brown; legs yellow. Head: vertex granulate; frons granulate; face granulate; temple in dorsal view narrow, width less than 1/2 eye width; malar space greater than 1/4 eye height; ocell-ocular distance greater than 2.5 times diameter of lateral ocellus; 18–20 flagellomeres. Mesosoma: mesoscutal lobes granulate; notauli scrobiculate, meeting at scutellum in triangular rugose area; scutellum granulate; prescutellar furrow with 5 cross carinae; mesopleuron granulate; precoxal sulcus scrobiculate, shorter than mesopleuron; venter granulate; propodeum with basal median areas margined, granulate, basal median carina absent, areola not distinctly margined, areolar area rugose, lateral areas entirely rugose. Wings: fore wing vein r shorter than vein 3RSa, vein 1cu-a beyond vein 1M; hind wing vein SC+R absent, vein M+CU shorter than vein 1M. Metasoma: first tergum longitudinally costate, length equal to apical width; second tergum longitudinally costate; anterior transverse groove present, straight; posterior transverse groove present; third tergum costate basally before transverse groove, granulate beyond transverse groove, smooth apically; terga 4–7 weakly smooth apically, weakly granulate basally; ovipositor shorter than metasomal tergum 1.

#### Holotype female.

Top label (white, printed) - COSTA RICA: Puntar [;] Golfo Dulce 24km W [;] Piedras Blancas [;] 200m, xii 89-iii 1990 [;] Col. Paul Hanson; second label (red, partially printed and hand written) - HOLOTYPE [;] Heterospilus [;] poqomam [;] P. Marsh. Deposited in ESUW.

#### Paratypes.

1 ♀, COSTA RICA: Puntarenas [;] Rd. to Rincon, 24km W. [;] Pan-Amer. Hwy, 200m [;] III-V 1989, Hanson & Gauld (ESUW).

#### Comments.

The lighter colored head, smooth metasomal terga 4–7 and white annulus at apex of flagellum are distinctive for this species.

#### Etymology.

Named for the Poqomam, a Mayan people of Guatemala.

**Figure 182.**
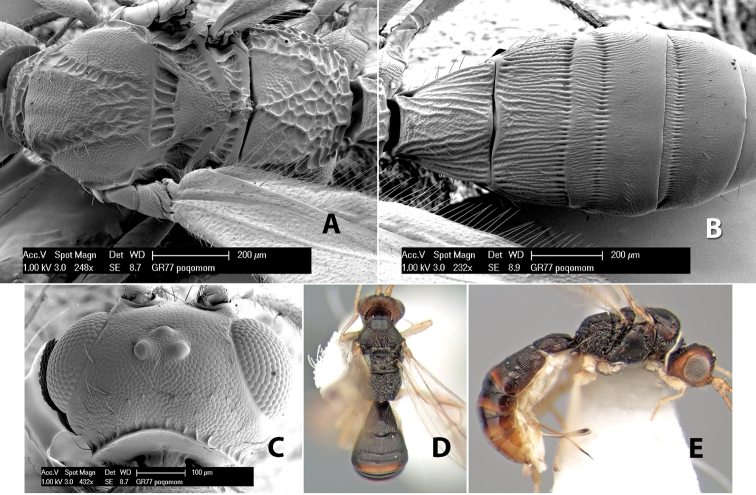
*Heterospilus poqomam* Marsh, sp. n.: **A–C** paratype **D–E** holotype.

### Heterospilus puertoviejoensis

Marsh sp. n.

http://zoobank.org/4871CB1E-AF7A-434E-9F75-F86EC2CE8B9A

http://species-id.net/wiki/Heterospilus_puertoviejoensis

Figure 183

#### Female.

Body size: 2.5–3.0 mm. Color: body dark brown, apical metasomal terga lighter brown; scape brown, flagellum brown with apical white annulus, apical 3–5 flagellomeres brown; legs yellow. Head: vertex granulate; frons granulate; face granulate or granulate-rugose; temple in dorsal view broad but sloping behind eye, width equal to 1/2 eye width; malar space greater than 1/4 eye height; ocell-ocular distance at least 2.5 times diameter of lateral ocellus; 22–27 flagellomeres. Mesosoma: mesoscutal lobes weakly granulate, often partially smooth; notauli scrobiculate, meeting at scutellum in triangular costate area; scutellum weakly granulate; prescutellar furrow with 3–5 cross carinae; mesopleuron granulate; precoxal sulcus smooth, shorter than mesopleuron; venter smooth; propodeum with basal median areas distinct but not always distinctly margined, granulate, basal median carina absent, areola not margined, areolar area areolate-rugose, lateral areas entirely rugose. Wings: fore wing vein r slightly shorter or equal to vein 3RSa, vein 1cu-a beyond vein 1M; hind wing vein SC+R present, vein M+CU shorter than vein 1M. Metasoma: first tergum longitudinally costate, length equal to apical width; second tergum longitudinally costate; anterior transverse groove present, straight; posterior transverse groove present; third tergum costate at base, smooth at apex; terga 4–7 smooth; ovipositor equal to length of metasomal terga 1 and 2 combined.

#### Holotype female.

Top Label (white, printed) - Costa Rica: Heredia [;] 3km. S. Puerto Viejo [;] OTS - La Selva, 100m [;] 16–30 IX.1992 [;] P. Hanson; second label (red, partially printed and hand written) - HOLOTYPE [;] Heterospilus [;] puertoviejoensis [;] P. Marsh. Deposited in ESUW.

#### Paratypes.

8 ♀♀, same data as holotype (ESUW). 9 ♀♀, Costa Rica: Heredia [;] 3km S. Puerto Viejo [;] OTS, La Selva, 100m [;] 1–15 ix 1992, P. Hanson [;] huertos Malaise trap [;] set by G. Wright (ESUW). 18 ♀♀, Costa Rica: Heredia [;] 3km. S. Puerto Viejo, [;] OTS, La Selva, 100m [;] xii.1992, P. Hanson (ESUW). 8 ♀♀, Costa Rica: Heredia [;] Est. Biol. La Selva [;] 50–150m, 10.26 N [;] 84.01 W, Aug. 1992 (ESUW). 1 ♀, COSTA RICA-Heredia Prov. [;] La Selva Biological Station [;] 10°26'N, 84°01'W, 100m [;] Malaise trap 11, #369 [;] 1.iii.1994 [;] Project ALAS(M.11.369) (ESUW). 2 ♀♀, COSTA RICA, Limon [;] Los Diamantes, Guapiles [;] 200m, 20/V/1988 [;] Col. Paul Hanson (ESUW). 1 ♀, Costa Rica: Puntarenas [;] Pen. Osa, Puerto [;] Jimenez, 10m, December [;] 1990, P. Hanson, Malaise (ESUW).

#### Comments.

The yellow stigma and areolate-rugose propodeum are distinctive for this species.

#### Etymology.

Named for the town of Puerto Viejo near the La Selva Biological Station where most of the type series was collected.

**Figure 183.**
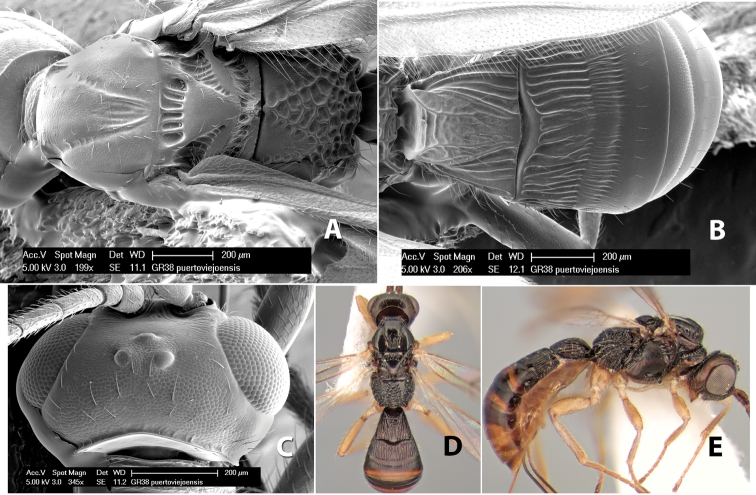
*Heterospilus puertoviejoensis* Marsh, sp. n.: **A–C** paratype **D–E** holotype.

### Heterospilus quitirrisi

Marsh sp. n.

http://zoobank.org/7B38EAB2-5A85-43D9-8224-3CFC7F559AA8

http://species-id.net/wiki/Heterospilus_quitirrisi

Figure 184

#### Female.

Body size: 2.5 mm. Color: head brown, face usually and frons sometimes honey yellow; scape yellow with weak lateral longitudinal brown stripe, flagellum yellow basally to brown apically, with white annulus near tip, apical most 1–2 flagellomeres brown; mesosoma brown, mesoscutum usually lighter brown; metasomal terga brown, tergum 2 yellow medially, terga 5–7 yellow; wing veins including stigma brown; legs yellow. Head: vertex granulate; frons granulate; face granulate; temple in dorsal view narrow, sloping behind eye, width about equal to 1/2 eye width; malar space greater than 1/4 eye height; ocell-ocular distance about twice diameter of lateral ocellus; 19–21 flagellomeres. Mesosoma: mesoscutal lobes granulate; notauli scrobiculate, meeting posteriorly in small rugose area; scutellum granulate; prescutellar furrow with 3–5 cross carinae; mesopleuron granulate; precoxal sulcus weakly scrobiculate, shorter than mesopleuron but often with carinae extending to posterior edge of mesopleuron; venter granulate; propodeum with basal median areas distinct but not always distinctly margined, granulate, basal median carina absent, areola not distinctly margined, areolar area rugose-areolate, lateral areas entirely rugose. Wings: fore wing vein r slightly shorter than vein 3RSa and nearly on same plane as vein 3RSa, vein 1cu-a beyond vein 1M; hind wing vein SC+R present, vein M+CU shorter than vein 1M. Metasoma: first tergum longitudinally costate, apical width equal to length; second tergum longitudinally costate; anterior transverse groove present, straight; posterior transverse groove present; third tergum costate basally, granulate apically; terga 4–7 granulate; ovipositor slightly shorter than metasomal tergum 1.

#### Holotype female.

Top label (white, printed) - COSTA RICA: Puntarenas [;] Rd. to Rincon, 10km W. [;] of Pan-Amer. Hwy. 100m [;] III-V 1989, Hanson & Gauld; second label (red, partially printed and hand written) - HOLOTYPE [;] Heterospilus [;] quitirrisi [;] P. Marsh. Deposited in ESUW.

#### Paratypes.

1 ♀, Costa Rica: Puntarenas [;] R.F. Golfo Dulce, [;] 3km SW. Rincon, 10m, [;] vi.1991, Paul Hanson (ESUW). 1 ♀, COSTA RICA: [;] Puntar [;] Golfo Dulce, 3km [;] SW Rincon, 10m, [;] VI-VII 1989, Paul Hanson (ESUW). 1 ♀, COSTA RICA: Puntarenas [;] R.F. Golfo Dulce, [;] 24km W. Piedras Blancas, [;] 200m [;] Feb. 1992, Paul Hanson (ESUW). 1 ♀, COSTA RICA-Heredia Prov. [;] La Selva Biological Station [;] 10°26'N, 84°01'W, 100m [;] Malaise trap 12, #390 [;] 4.iv.1994 [;] Project ALAS (M.12.390) (ESUW). 1 ♀, COSTA RICA, Puntar. [;] Golfo Dulce, 24km W. [;] PiedrasBlancas, 200m [;] III-VI-90 (MICR).

#### Comments.

The bicolored body and the flagellum with a white annulus at the tip are distinctive for this species.

#### Etymology.

Named for the Quitirrisi, an indigenous people of Costa Rica.

**Figure 184.**
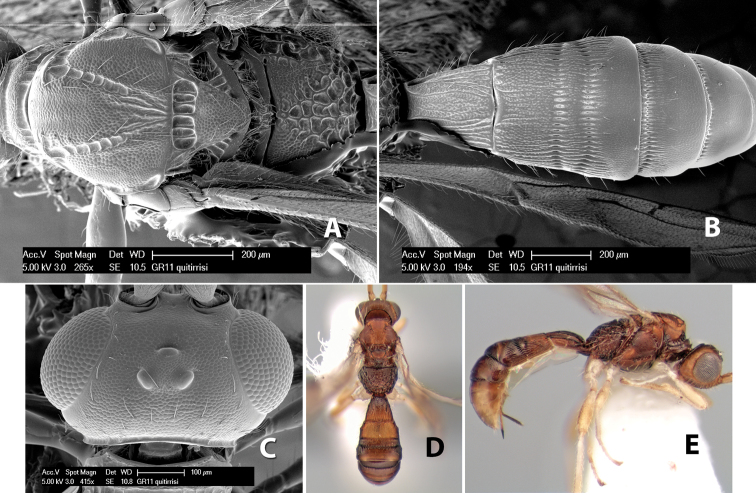
*Heterospilus quitirrisi* Marsh, sp. n.: **A–C** paratype **D–E** holotype.

### Heterospilus rama

Marsh sp. n.

http://zoobank.org/1E4051BC-3247-44E6-9AB0-05EE8A43766B

http://species-id.net/wiki/Heterospilus_rama

Figure 185

#### Female.

Body size: 2.5–3.0 mm. Color: head dark brown, occasionally face lighter brown; scape yellow without lateral longitudinal brown stripe, flagellum brown with apical white annulus, apical 3–5 flagellomeres brown; mesosoma and metasomal terga dark brown; wing veins including stigma brown; legs yellow. Head: vertex granulate, rarely with transverse weak rugae behind ocelli; frons granulate; face granulate; temple in dorsal view narrow, sloping behind eye, width less that 1/2 eye width; malar space greater than 1/4 eye height; ocell-ocular distance 2–2.5 times diameter of lateral ocellus; 20–22 flagellomeres. Mesosoma: mesoscutal lobes granulate; notauli scrobiculate, meeting at scutellum in triangular rugose area; scutellum granulate; prescutellar furrow with 3–5 cross carinae; mesopleuron granulate; precoxal sulcus weakly scrobiculate or smooth, shorter than mesopleuron; venter granulate; propodeum with basal median areas margined, granulate, basal median carina absent, areola not margined, areolar area rugose, lateral areas entirely rugose. Wings: fore wing vein r shorter than vein 3RSa, vein 1cu-a beyond vein 1M; hind wing vein SC+R absent or rarely weakly present, vein M+CU shorter than vein 1M. Metasoma: first tergum longitudinally costate, length equal to apical width; second tergum longitudinally costate; anterior transverse groove present, straight; posterior transverse groove present; third tergum costate anteriorly, granulate posteriorly; terga 4–7 granulate; ovipositor as long as 1/2 length of metasoma.

#### Holotype female.

Top label (white, printed) - Costa Rica: Heredia [;] Puerto Viejo [;] OTS, La Selva, 100m [;] iv.1991, P. Hanson; second label (red, partially printed and hand written) - HOLOTYPE [;] Heterospilus [;] rama [;] P. Marsh. Deposited in ESUW.

#### Paratypes.

1 ♀, Costa Rica: Puntarenas, ACO [;] Golfito, R.F. Golfo Dulce [;] Est. Agujas, 250–350m [;] 4–20.vi.1999, J. Azofeifa [;] L.S. 276750-526550 #52746 [;] Amarilla (ESUW). 2 ♀♀, Costa Rica: Limon, ACLAC [;] Central, R.B. Hitoy Cerere [;] Send. Espavel, 560m [;] 19.v–19.vi.1998, E. Rojas [;] L.S. 400702-570120 #52200 [;] Malaise trap (ESUW). 9 ♀♀, Costa Rica: Puntarenas [;] San Vito, Estac. Biol. [;] Las Alturas, 1500m [;] xii.1991, i.1992, ii.1992 and 15–31 Oct. 1991, Paul Hanson (ESUW). 1 ♀, Costa Rica, Puntarenas [;] San Vito, 1200m café [;] III-IV-1996, P. Hanson (ESUW). 1 ♀, COSTA RICA: [;] Puntar [;] Golfo Dulce, 3km [;] SW. Rincon, 10m [;] VI-VII 1989, Hanson (ESUW). 1 ♀, COSTA RICA: Puntarenas [;] R.F. Golfo Dulce, [;] 24km W. Piedras Blancas, [;] 200m, [;] Feb. 1992, Paul Hanson (ESUW). 1 ♀, COSTA RICA: [;] Puntar. Golfo Dulce [;] 24km W Piedras Blancas [;] 200m, vi-viii 1989 [;] Hanson (ESUW). 1 ♀, COSTA RICA: Puntarenas [;] Reserva Forestal Golfo Dulce [;] 3km SW of Rincon, 10m [;] Mar-April 1992, P. Hanson [;] primary forest, Malaise trap (ESUW). 1 ♀, COSTA RICA: Puntarenas [;] RF Golfo Dulce el 200m [;] 24km W Piedras Blancas [;] P. Hanson xi.1992 (TAMU).

#### Comments.

The absence of hind wing vein SC+R, the strongly granulate and dull mesoscutum and the granulate apical metasomal terga are distinctive for this species.

#### Etymology.

Named for the Rama, an indigenous people of Nicaragua.

**Figure 185.**
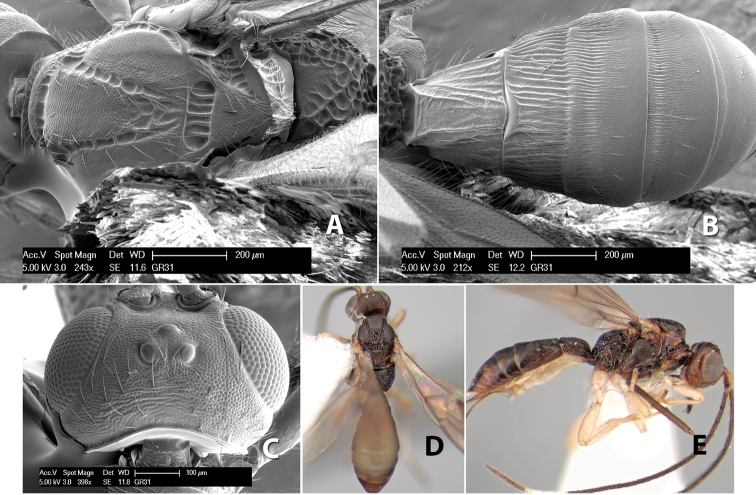
*Heterospilus rama* Marsh, sp. n.: **A–C** paratype **D–E** holotype.

### Heterospilus richardsi

Marsh & Melo

http://species-id.net/wiki/Heterospilus_richardsi

Figure 186

Heterospilus richardsi Marsh & Melo, 1999: 20.

#### Female.

Body size: 2.5–3.0 mm. Color: body brown, head slightly lighter; scape yellow without lateral brown stripe, flagellum brown; wing veins including stigma brown; legs yellow. Head: vertex granulate; frons granulate; face weakly granulate or smooth; temple in dorsal view narrow, sloping behind eye, width less than 1/2 eye width; malar space greater than 1/4 eye height; ocell-ocular distance 2.5 times diameter of lateral ocellus; 21–24 flagellomeres. Mesosoma: mesoscutal lobes granulate; notauli scrobiculate, meeting at scutellum in small triangular costate area; scutellum weakly granulate or smooth; prescutellar furrow with 3 cross carinae; mesopleuron granulate; precoxal sulcus weakly scrobiculate, shorter than mesopleuron; venter granulate; propodeum with basal median areas margined, granulate, basal median carina absent, areola not distinctly margined, areolar area areolate-rugose, lateral areas entirely rugose. Wings: fore wing vein r shorter than vein 3RSa, vein 1cu-a beyond vein 1M; hind wing vein SC+R absent, vein M+CU shorter than vein 1M. Metasoma: first tergum longitudinally costate, length slightly greater than apical width; second tergum longitudinally costate, with raised smooth semicircular area medially at base; anterior transverse groove present, straight; posterior transverse groove present; third tergum costate-granulate basally, weakly granulate apically; terga 4–7 very weakly granulate and shining, appearing smooth in lower magnification; ovipositor as long as or longer than metasoma.

#### Specimens examined.

2 ♀♀, Costa Rica, Heredia, Estacion Biol. La Selva. Also recorded from Brazil.

#### Biology.

Reared from nests of *Spilonema alini* Antropov in Brazil and *Microstigmus* sp. in Costa Rica (Hymenoptera: Sphecidae) (Marsh and Melo 1999).

#### Comments.

The brown body color, brown flagellum and the raised smooth semicircular area at base of metasomal tergum 2 are distinctive for this species.

**Figure 186.**
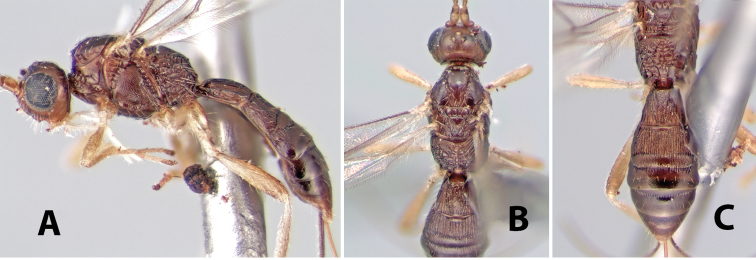
*Heterospilus richardsi* Marsh and Melo.

### Heterospilus robbieae

Marsh sp. n.

http://zoobank.org/F75DF079-A852-42B1-A530-387CAC92C57C

http://species-id.net/wiki/Heterospilus_robbieae

Figure 187

#### Female.

Body size: 2.5 mm. Color: head dark brown; scape yellow with lateral longitudinal brown stripe, flagellum brown with apical white annulus, apical 5–7 flagellomeres brown; mesosoma dark brown, honey yellow along notauli; metasomal terga brown, apical terga usually lighter brown; wing veins including stigma brown; legs yellow, femora darker brown on apical half. Head: vertex granulate; frons weakly granulate; face weakly granulate or smooth; temple in dorsal view narrow, sloping behind eye, width less than 1/2 eye width; malar space greater than 1/4 eye height; ocell-ocular distance about 2.5 times diameter of lateral ocellus; 21–23 flagellomeres. Mesosoma: mesoscutal lobes granulate; notauli smooth, meeting at scutellum in triangular costate area; scutellum granulate; prescutellar furrow with 3–5 cross carinae; mesopleuron granulate; precoxal sulcus smooth, shorter than mesopleuron; venter weakly granulate or smooth; propodeum with basal median areas margined, granulate, basal median carina absent, areola not margined, areolar area areolate-rugose, lateral areas entirely rugose, propodeum with small but distinct tubercle near base of petiole above hind coxa. Wings: fore wing vein r shorter than vein 3RSa, vein 1cu-a beyond vein 1M; hind wing vein SC+R absent, vein M+CU shorter than vein 1M. Metasoma: first tergum longitudinally costate, length greater than apical width; second tergum longitudinally costate; anterior transverse groove present, straight; posterior transverse groove present; third tergum weakly costate or smooth basally, smooth apically; terga 4–7 smooth; ovipositor as long as metasomal terga 1 and 2 combined.

#### Holotype female.

Top label (white, printed) - Costa Rica: Alajuela [;] 5km. W San Ramon [;] 1200m, April 1997 [;] O. Castro & P. Hanson; second label (red, partially printed and hand written) - HOLOTYPE [;] Heterospilus [;] robbieae [;] P. Marsh. Deposited in ESUW.

#### Paratypes.

5 ♀♀, same data as holotype (ESUW). 1 ♀, Costa Rica: San Jose [;] San Antonio de Escazu [;] 1700m, September 1996 [;] P. Hanson & C. Flores [;] Malaise trap (ESUW). 1 ♀, COSTA RICA-San Jose [;] Zurqui de Moravia [;] 1600m, ii.1995 [;] P. Hanson (ESUW).

#### Comments.

The absence of hind wing vein SC+R, the bicolored mesoscutum and the strongly sloping temple behind the eye are distinctive for this species.

#### Etymology.

Named for a dear friend in Christ, Robbie Litzman.

**Figure 187.**
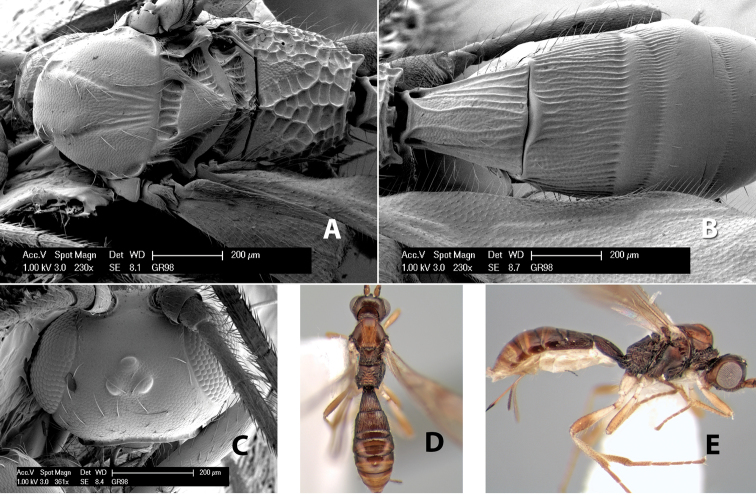
*Heterospilus robbieae* Marsh, sp. n.: **A–C** paratype **D–E** holotype.

### Heterospilus rojasi

Marsh sp. n.

http://zoobank.org/52D497D1-BE21-4EB8-B006-117BD68B6567

http://species-id.net/wiki/Heterospilus_rojasi

Figure 188

#### Female.

Body size: 3.0 mm. Color: head dark brown with eye orbits, lower face and malar space often yellow; scape yellow with lateral longitudinal brown stripe; flagellum brown, preapical 3–5 flagellomeres white, apical 3–5 brown; mesosoma and metasoma dark brown; wing veins including stigma brown; legs yellow. Head: vertex granulate with weak striae or rugae behind ocelli; frons granulate-striate; face rugose; temple in dorsal view broad but sloping behind eye, not bulging, width equal to 1/2 eye width; malar space greater than 1/4 eye height; ocell-ocular distance 2.0–2.5 times diameter of lateral ocellus; 25 flagellomeres. Mesosoma: mesoscutal lobes granulate; notauli scrobiculate, meeting at scutellum in triangular rugose area; scutellum granulate; prescutellar furrow with 3–5 cross carinae; mesopleuron granulate; precoxal sulcus weakly scrobiculate, shorter than mesopleuron; venter granulate; propodeum with basal median areas margined, granulate, basal median carina absent, areola weakly margined, areolar area areolate, lateral areas entirely rugose, propodeum with distinct tubercle just above hind coxae. Wings: fore wing vein r shorter than vein 3RSa, vein 1cu-a beyond vein 1M; hind wing vein SC+R present, vein M+CU shorter than vein 1M. Metasoma: first tergum longitudinally costate, length greater than apical width; second tergum longitudinally costate, width nearly 4 times length; anterior transverse groove present, straight; posterior transverse groove present; third tergum costate at base, granulate at apex; terga 4–7 granulate; ovipositor equal to 1/2 length of metasoma.

#### Holotype female.

Top label (white, printed) - Costa Rica: Limon [;] Sector Cocori, 100m [;] 30km N Cariari, i.1995 [;] E. Rojas, Malaise #4526 [;] L.N. 286000-567500; second label (red, partially printed and hand written) - HOLOTYPE [;] Heterospilus [;] rojasi [;] P. Marsh. Deposited in ESUW.

#### Paratypes.

1 ♀, Costa Rica: Limon [;] 30km N. Cariari, 100m [;] Sector Cocori, Malaise [;] iii.1995, E. Rojas #4524 [;] L.N. 286000-567500 (ESUW). 1 ♀, top label - COSTA RICA, Heredia: [;] Est. Biol. La Selva, 50- [;] 150m, 10°26'N, 84°01'W [;] Apr. 1996, INBio-OET; second label - 1 Abril 1996 [;] Bosque primario [;] M/12/613 (INBC).

#### Comments.

The bicolored head and white annulus on the flagellum are distinctive for this species.

#### Etymology.

Named for the collector of the type series, E. Rojas.

**Figure 188.**
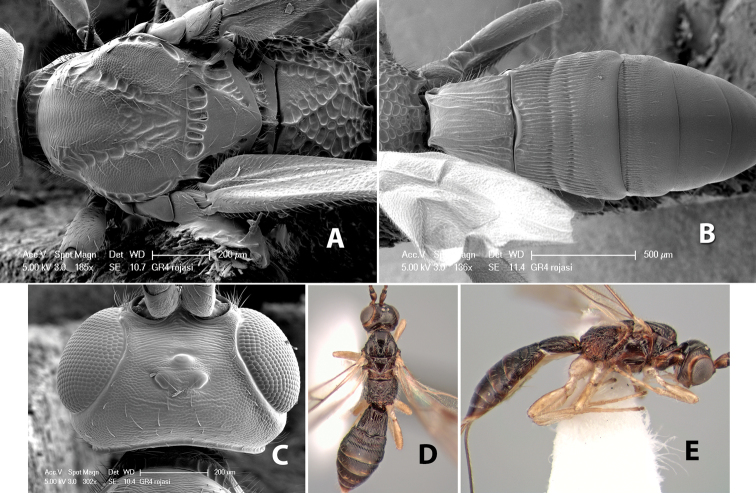
*Heterospilus rojasi* Marsh, sp. n.: **A–C** paratype **D–E** holotype.

### Heterospilus sabrinae

Marsh sp. n.

http://zoobank.org/21381368-5732-42FC-A441-5501318270A7

http://species-id.net/wiki/Heterospilus_sabrinae

Figure 189

#### Female.

Body size: 2.5–3.0 mm. Color: body dark brown, metasomal tergum 2 usually yellow medially, terga 5–7 yellow or lighter brown; scape yellow with lateral longitudinal brown stripe, flagellum brown with apical white annulus, apical 5–7 flagellomeres brown; wing veins including stigma brown; legs yellow with hind femur brown on apical half. Head: vertex granulate; frons granulate; face granulate; temple in dorsal view narrow, sloping behind eye, width lass than 1/2 eye width; malar space greater than 1/4 eye height; ocell-ocular distance twice diameter of lateral ocellus; 21–25 flagellomeres. Mesosoma: mesoscutal lobes granulate; notauli scrobiculate, meeting at scutellum in small indistinct costate area; scutellum granulate; prescutellar furrow with 5 cross carinae; mesopleuron granulate; precoxal sulcus scrobiculate, shorter than mesopleuron; venter granulate; propodeum with basal median areas distinct but not margined, long and somewhat narrow, granulate, basal median carina absent, areola not margined, areolar area areolate-rugose, lateral areas entirely rugose, propodeum with distinct tubercle above hind coxa. Wings: fore wing vein r shorter than vein 3RSa, vein 1cu-a beyond vein 1M; hind wing vein SC+R absent, vein M+CU shorter than vein 1M. Metasoma: first tergum longitudinally costate, occasionally granulate medially at base, length equal to apical width; second tergum longitudinally costate-granulate; anterior transverse groove present, straight or very weakly sinuate; posterior transverse groove present; third tergum entirely granulate except for costate transverse groove, or granulate-costate at base, granulate apically; terga 4–7 weakly granulate, occasionally smooth at extreme base; ovipositor as long as metasomal tergum 1.

#### Holotype female.

Top label (white, printed) - Costa Rica: Puntarenas [;] R.F. Golfo Dulce [;] 3km SW. Rincon, 10m, [;] vi.1991, Paul Hanson; second label (red, partially printed and hand written) - HOLOTYPE [;] Heterospilus [;] sabrinae [;] P. Marsh. Deposited in ESUW.

#### Paratypes.

7 ♀♀, same data as holotype with additional date of Oct. 1991 (ESUW). 1 ♀, Costa Rica: Puntarenas [;] ACO, Golfito, PN Corcovado [;] Est. Agujas. Las Quebraditas [;] 640m, 8–9.ix.1999, J. Azofeifa [;] L.S. 275200-520100 #53263 [;] Amarilla (ESUW). 2 ♀♀, Costa Rica: Puntarenas [;] ACO. Golfito, RF Golfo Dulce [;] Est. Agujas, 250–300m [;] 3–24.vi.1999, J. Azofeifa [;] L.S. 276750-526550 #52840 [;] Red de Golpe (ESUW). 1 ♀, Costa Rica: Puntarenas [;] Pen. Osa, Puerto Jimenez [;] 10m, January 1991, full sun, [;] grassy & weedy site [;] P. Hanson, ex. Malaise (ESUW). 1 ♀, Costa Rica: Puntarenas [;] Golfo Dulce, 24km W. [;] Piedras Blancas, 200m [;] ii.1993, Paul Hanson (ESUW). 1 ♀, Costa Rica: Puntarenas [;] Pen. Osa, 23km. N. Pto. [;] Jimenez, La Palma, 10m [;] viii-ix.1991, P. Hanson [;] Malaise, in large trees (ESUW). 1 ♀, Costa Rica: Puntarenas [;] San Vito, Las Cruces [;] Wilson Botanical Gardens [;] 18–22.iii.1990, 1150m [;] J.S. Noyes (ESUW). 1 ♀, Costa Rica: Puntarenas [;] Peninsula Osa, 10 meters [;] 5km NW Puerto Jimenez, [;] xi-xii.1990, Paul Hanson [;] abandoned cacao orchard (ESUW). 2 ♀♀, Costa Rica, Puntarenas [;] Pe. Osa, Puerto Jimenez [;] 10m, VI-1993, P. Hanson (ESUW). 1 ♀, Costa Rica: Puntarenas [;] Peninsula Osa Puerto [;] Jimenez, 10m, x-xi.1991 [;] P. Hanson, Malaise trap [;] grassy, disturbed site (ESUW). 1 ♀, COSTA RICA: [;] Puntar, Golfo Dulce [;] 24km W Piedras Blancas [;] 200m, vi-viii 1989 [;] Hanson (ESUW). 1 ♀, Costa Rica, Puntarenas [;] Pen. Osa, 23km. N. Pro. [;] Jimenez, La Palma, 10m [;] VI-VIII-1993, P. Hanson (ESUW). 1 ♀, Misc. Doryctinae [;] Costa Rica, Puntarenas [;] R. F. Golfo Dulce, 5km. W. [;] Piedras Blancas, 100m [;] IV-V-1993, P. Hanson (ESUW). 2 ♀♀, COSTA RICA: Puntar. [;] R.B. Carara, Estac. [;] Quebrada Bonita, 50m [;] V-VI 1989, P. Hanson (ESUW). 1 ♀, Costa Rica: Puntarenas, ACO [;] Golfito, Est. Agujas, 250–350m [;] Res. Ftal. Golfo Dulce, Amarilla [;] 3–24.vii.1999, J. Azofeifa [;] L.S. 276750-526550 #52839 (ESUW). 2 ♀♀, COSTA RICA-Heredia Prov. [;] La Selva Biological Station [;] 10°26'N, 84°01'W, 100m [;] Malaise trap 1, #248 and Canopy fogging 28 [;] 1.xi.1993 and 22.x.1994 [;] Project ALAS (M.01.248) and (FPM28) (ESUW). 1 ♀, top label - COSTA RICA: Prov. [;] Heredia, F. La Selva [;] 3km S Pto. Viejo [;] 10°26'N, 84°01'W; second label - 21–23.iv.1989 [;] H.A. Hespenheide: third label - Malaise trap - 1–2 year [;] Second Growth-Forest edge (ESUW). 1 ♀, COSTA RICA: [;] Heredia, Chilamate [;] 75m, xii 89-iii 1990 [;] Hanson & Godoy (ESUW). 1 ♀, Costa Rica: Heredia [;] Est. Biol. La Selva [;] 50–150m, 10°26'N [;] 84°01'W, Aug. 1992 (ESUW). 1 ♀, Costa Rica: Heredia [;] 3km. S. Puerto Viejo [;] OTS - La Selva, 100m [;] 16–30 IX.1992 [;] P. Hanson (ESUW). 4 ♀♀, Costa Rica: Heredia [;] 3km. S. Puerto Viejo [;] OTS, La Selva, 100m [;] xii.1992, xi.1992, I-II-1993 and III-IV-1993, P. Hanson (ESUW). 3 ♀♀, Costa Rica: Heredia [;] 3km S. Puerto Viejo [;] OTS, La Selva, 100m [;] 1–15 ix 1992 and X.1992, P. Hanson [;] huertos Malaise trap [;] set by G. Wright (ESUW). 1 ♀, Costa Rica: Heredia [;] Est. Biol. La Selva [;] 50–150m, 10°26'N [;] 84°01'W [;] ii-iv 1993, P. Hanson [;] huertos Malaise trap [;] set by G. Wright (ESUW). 2 ♀♀, Costa Rica: Cartago [;] Braulio Carillo N.P. [;] 600m, 25.iii.1990 [;] J. S. Noyes, coll. (ESUW). 1 ♀, COSTA RICA: San Jose [;] P.N. Braulio Carillo [;] 9.5km E tunnel, 1000m [;] vii-ix 1989, P.Hanson (ESUW). 1 ♀, Costa Rica: San Jose [;] San Antonio de Escazu [;] 1300m, vi-vii.1998 [;] W. Eberhard (ESUW). 1 ♀, Costa Rica: Limon, 640m [;] Res. Biol. Hitoy Cerere [;] Est. H.C., Send. Bobocara [;] 17.vi–17.vii.1999, F. Umana [;] L.N. 184250-640500 #52859 [;] ex. Malaise trap (ESUW). 1 ♀, COSTA RICA: Limon [;] 16km W. Guapiles [;] 400m, III 1989 [;] col. Paul Hanson (ESUW). 2 ♀♀, Costa Rica: Alajuela, ACA [;] R.B.San Ramon, 900m [;] Malaise, 16–30.vii.1998 [;] P. Hanson (ESUW). 2 ♀♀, top label - Costa Rica: Guanacaste [;] Santa Rosa Natl. Park [;] 300m, ex. Malaise trap [;] Site #: SE-6-C and blank [;] Dates: 18.x–8.xi.1986 and 8–29.xi.1986 [;] I.D. Gauld & D. Janzen; second label - [SE] Bosque San Emilio [;] 50yr old deciduous forest [;] [C] more or less fully [;] shaded as possible (ESUW). 1 ♀, top label - Costa Rica: Guanacaste [;] Santa Rosa Natl. Park [;] 300m, ex. Malaise trap [;] Site #: blank [;] Dates: 18.x–8.xi.1986 [;] I.D. Gauld & D. Janzen; second label - [SE] Bosque San Emilio [;] 50yr old deciduous forest [;] [O] in clearing, fully [;] isolated part of day (ESUW). 1 ♀, top label - Costa Rica: Guanacaste [;] Santa Rosa Natl. Park [;] 300m, ex. Malaise trap [;] Site #: H-1-O [;] Dates: 18.x–8.xi.1986 [;] I.D. Gauld & D. Janzen; second label - [H] open regenerating [;] woodland <10 year old [;] [O] in clearing, fully [;] isolated part of day (ESUW). 2 ♀♀, top label - Costa Rica: Guanacaste [;] Santa Rosa National Pk. [;] 300m, Malaise, Ian Gauld [;] 27.ix–18.x.1986; second label - Bosque San Emilio [;] 50 yr. old deciduous [;] forest, Full Shade; third label - SE-8-C [;] 27.ix–18.x.86 (ESUW). 1 ♀, COSTA RICA: [;] Guanacaste [;] Estac. Mengo [;] SW Volcan Cacao [;] 1100m, 1988–1989 (ESUW). 1 ♀, Costa Rica: Guanacaste [;] Arenales, W. side of [;] Volcan Cacao, 900m [;] xi-xii 1990, P. Hanson (ESUW). 1 ♀, Costa Rica: Guanacaste [;] Est. Cacao, 1000–1150m [;] vii.1996, A. Masis, Malaise [;] L.N. 323150-375500 #47555 (ESUW). 1 ♀, Costa Rica: Guanacaste, Bagaces [;] Pque. Ntl. Palo Verde, Sct. P. Verde [;] Cerro, Guayacan, 212m, Malaise [;] 13.x–11.xi.1999, I. Jimenez [;] L.N. 259350-389600 #54006 (ESUW). 1 ♀, Costa Rica: Guanacaste [;] Est. Pitilia, 700m [;] 9km. S de Santa Cecilia [;] viii-ix.1996, P. Rios & [;] C. Moraga, Malaise [;] L.N. 329950-380450 #47563 (ESUW). 2 ♀♀, Costa Rica: Guanacaste [;] Bagaces, P.N. Palo Verde [;] Sec. Catalina, Fila Catalina [;] 250m, 11 Oct–9 Nov 1999 [;] I. Jimenez, Malaise trap [;] L.N. 257400-400000 #54003 (ESUW). 1 ♀, Costa Rica: Guanacaste, ACT [;] Bagaces, P.N. Palo Verde [;] Sector Palo Verde [;] 0–50m, Red de Golpe [;] 5–12.x.1999, I. Jimenez [;] L.N. 260952-386020 #53602 (ESUW). 1 ♀, Costa Rica: Guanacaste, ACT [;] Bagaces, P.N. Palo Verde, 212m [;] Sec. Palo Verde, Cerro Guayacan [;] 13.ix–13.x.1999, I. Jimenez, Malaise [;] L.N. 259350-389600 #53499 (ESUW). 1 ♀, Costa Rica: Guanacaste, ACT [;] Bagaces, PN Palo Verde [;] Sec. Palo Verde, 250m [;] Amarilla, #53297 [;] L.N. 257400-400000 (ESUW). 2 ♀♀, Costa Rica: Guanacaste [;] Bagaces, P.N. Palo Verde [;] Sect. Palo Verde, Cerro Guayacan [;] 212m, Malaise trap [;] 13.ix–13.x.1999, I. Jimenez [;] L.N. 259350-389000 #53499 (ESUW). 2 ♀♀, COSTA RICA: Puntarenas [;] RF Golfo Dulce el 200m [;] 24km W Piedras Blancas [;] P. Hanson vii.1992 and xii.1992 (TAMU).

#### Comments.

The short ovipositor, bicolored metasomal tergum 2 and the tubercles on the propodeum above the hind coxa are distinctive for this species.

#### Etymology.

Named for my daughter-in-law, Sabrina Marsh.

**Figure 189.**
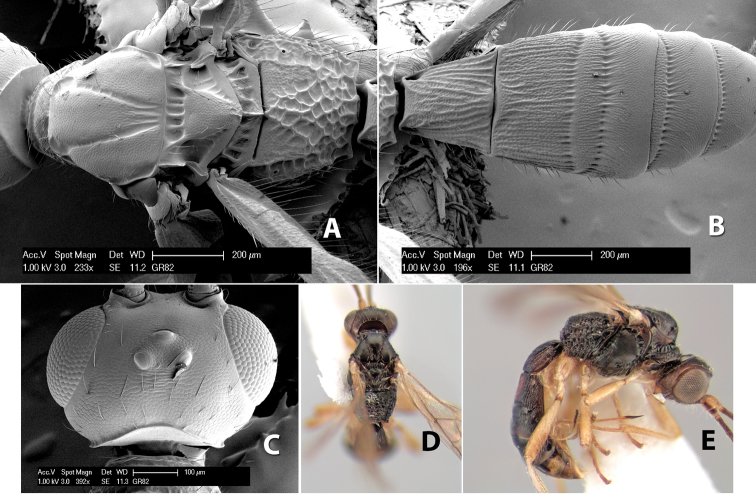
*Heterospilus sabrinae* Marsh, sp. n.: **A–C** paratype **D–E** holotype.

### Heterospilus saminae

Marsh sp. n.

http://zoobank.org/57BC5922-FA20-46F7-AC24-3BFD06AFDDFC

http://species-id.net/wiki/Heterospilus_saminae

Figure 190

#### Female.

Body size: 3.0 mm. Color: body dark brown, metasomal tergum 2 usually yellow medially, terga 5–7 yellow; scape yellow or light brown with lateral longitudinal dark brown stripe, flagellum brown with apical white annulus, apical 5–7 flagellomeres brown; wing veins including stigma brown; legs yellow. Head: vertex granulate; frons weakly granulate, occasionally partially smooth; face smooth; temple in dorsal view narrow, sloping behind eye, width equal to 1/2 eye width; malar space slightly greater than 1/4 eye height; ocell-ocular distance slightly greater than 2.5 times diameter of lateral ocellus; 24–26 flagellomeres. Mesosoma: mesoscutal lobes granulate; notauli usually smooth, occasionally weakly partially scrobiculate, meeting at scutellum in unsculptured area; scutellum granulate; prescutellar furrow with 3–5 cross carinae; mesopleuron granulate; precoxal sulcus scrobiculate, shorter than mesopleuron; venter granulate; propodeum with basal median areas margined, granulate, basal median carina absent, areola not margined, areolar area areolate-rugose, lateral areas entirely rugose and with 2 scrobiculate grooves dorsally, propodeum with small but distinct tubercle above hind coxa. Wings: fore wing vein r shorter than vein 3RSa, vein 1cu-a usually beyond vein 1M, occasionally interstitial; hind wing vein SC+R absent, vein M+CU shorter than vein 1M. Metasoma: first tergum longitudinally costate, often rugose medially, length greater than apical width; second tergum longitudinally costate-granulate; anterior transverse groove present, straight or very slightly sinuate; posterior transverse groove present; third tergum entirely granulate except costate transverse groove; terga 4–7 granulate; ovipositor shorter than metasomal tergum 1.

#### Holotype female.

Top label (white, printed) - Costa Rica: Alajuela Prov. [;] Area Conservation de Arenal [;] Est. San Ramon, Malaise #3 [;] in veg. on Sendero W.F. [;] 5 June to 15 July 1998 [;] N. Zitani, S. Dadelahi, [;] K. Krenzelok, R. Fenoff; second label (red, partially printed and hand written) - HOLOTYPE [;] Heterospilus [;] saminae [;] P. Marsh. Deposited in ESUW.

#### Paratypes.

1 ♀, same data as holotype (ESUW). 4 ♀♀, Costa Rica: Alajuela [;] R.B. San Ramon [;] 800m, xi-xii.1998 [;] P. Hanson (ESUW). 2 ♀♀, Costa Rica: Puntarenas [;] R.F. Golfo Dulce, [;] 24km W. Piedras Blancas [;] 200m, [;] Feb. 1992 and xii.1991, Paul Hanson (ESUW). 1 ♀, Costa Rica: Puntarenas [;] San Vito, Las Cruces [;] Wilson Botanical Gardens [;] 18–22.iii.1990, 1150m [;] J.S. Noyes (ESUW). 1 ♀, Costa Rica: Alajuela [;] 5km W San Ramon [;] 1200m, ii.1997 [;] O.Castro & P.Hanson (ESUW). 1 ♀, Costa Rica: Alajuela [;] Est. San Ramon, Light trap [;] UV/F - in vegetation [;] 10:30–11:30 p.m. [;] 24.vi.1998 [;] Dadelahi & Zitani (ESUW). 1 ♀, Costa Rica: Puntarenas [;] ACO, Golfito, RF Golfo Dulce [;] Est. Agujas, 250–300m [;] 2–22.x.1999, J. Azofeifa [;] L.S. 276750-526550 #53490 [;] Amarilla (ESUW).

#### Comments.

The unsculptured area where the notauli meet, the bicolored metasomal terga and the propodeal sculpture are distinctive for this species.

#### Etymology.

Named for Samin Dadelahi, former student at the University of Wyoming who collected and sorted many braconids from Costa Rica.

**Figure 190.**
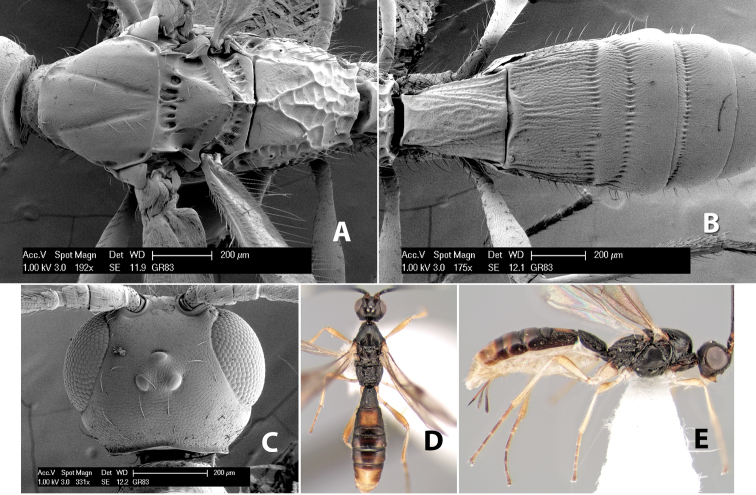
*Heterospilus saminae* Marsh, sp. n.: **A–C** paratype **D–E** holotype.

### Heterospilus sergeyi

Marsh sp. n.

http://zoobank.org/3776CC74-DB01-4DE5-AEA6-4A997F677DCF

http://species-id.net/wiki/Heterospilus_sergeyi

Figure 191

#### Female.

Body size: 3.5 mm. Color: body dark brown or black, mesoscutum and propodeum often lighter brown, apical metasomal terga honey yellow; scape yellow with lateral longitudinal brown stripe, flagellum brown with apical white annulus, apical 5–7 flagellomeres brown; wing veins including stigma brown; legs yellow, apical 1/4 of femora light to medium brown. Head: vertex granulate, usually with transverse rugae behind ocelli; frons granulate; face granulate or granulate-rugose; temple in dorsal view narrow, sloping behind eye, width less than 1/2 eye width; malar space equal to 1/4 eye height; ocell-ocular distance 1.5–2.0 times diameter of lateral ocellus; 27–29 flagellomeres. Mesosoma: mesoscutal lobes granulate; notauli scrobiculate, meeting at scutellum in triangular costate area; scutellum weakly granulate or smooth; prescutellar furrow with 3–6 cross carinae; mesopleuron granulate; precoxal sulcus smooth, shorter than mesopleuron; venter granulate; propodeum with basal median areas margined, granulate, basal median carina absent, areola not margined, areolar area areolate-rugose, lateral areas entirely rugose, propodeum with distinct tubercle above hind coxa at base of petiole. Wings: fore wing vein r shorter than vein 3RSa, vein 1cu-a beyond vein 1M; hind wing vein SC+R absent, vein M+CU shorter than vein 1M. Metasoma: first tergum longitudinally costate, length greater than apical width; second tergum longitudinally costate; anterior transverse groove present, straight; posterior transverse groove present; third tergum smooth at least medially, occasionally weakly costate laterally; terga 4–7 smooth; ovipositor 1/2–3/4 length of metasoma.

#### Holotype female.

Top label (white, printed) - COSTA RICA: Limon [;] 4km NE Bribri [;] 50m, IX–XI 1989 [;] col. Paul Hanson; second label (red, partially printed and hand written) - HOLOTYPE [;] Heterospilus [;] sergeyi [;] P. Marsh. Deposited in ESUW.

#### Paratypes.

2 ♀♀, same data as holotype with additional date of iv-vi 1990 (ESUW). 7 ♀♀, COSTA RICA: Limon [;] 16km W. Guapiles [;] 400m, III 1989, vii-ix 1990, v-vi 1990, II/1989 and iii-v 1990 [;] col. Paul Hanson (ESUW). 1 ♀, Costa Rica: Limon, ACLAC [;] Central, R.B. Hitoy Cerere [;] Send. Espavel, 560m [;] 19.v–19.vi.1998, E. Rojas [;] L.S. 400702-570120 #52200 [;] Malaise Trap (ESUW). 1 ♀, Costa Rica: Limon [;] 30km N Cariari, 100m [;] Sector Cocori, Malaise [;] iii.1995, E. Rojas #4524 [;] L.N. 286000-567500 (ESUW). 1 ♀, COSTA RICA, Limon [;] 4km NE Bribri [;] 50m, IX-XI 1989 [;] col. Paul Hanson (ESUW). 1 ♀, COSTA RICA: Limon [;] P.N. Tortuguero [;] Est. 4-esquinas, 0m. [;] IX-X 1989, J. Solano (ESUW). 1 ♀, Costa Rica: Alajuela, ACA [;] R.B. San Ramon, 900m [;] Malaise, 16–30.vii.1998 [;] P. Hanson (ESUW). 2 ♀♀, Costa Rica: Alajuela [;] 5km. W San Ramon [;] 1200m, July 1997 and ii.1997 [;] O. Castro & P. Hanson (ESUW). 2 ♀♀, Costa Rica: Alajuela [;] San Carlos, R.F. Arenal [;] Send Pilon, 600m, Malaise [;] 9.ix–1.x.1999 and 26.viii–22.ix.1999, G. Carballo [;] L.N. 269100-457900 #53917 and L.N. 269200-458050 #54374 (ESUW). 1 ♀, Costa Rica: Alajuela [;] ACA, R.B.San Ramon [;] 875m, Malaise near [;] station, 10–24.viii.1998 [;] L.J. van der Ent (ESUW). 3 ♀♀, Costa Rica, Alajuela Prov. [;] ACA, Res. Bio. San Ramon [;] Malaise #2, 6 June–15 July [;] 1998, S. Dadelahi, R. Fenoff [;] K. Krenzelok, N. Zitani (ESUW). 1 ♀, Costa Rica, Alajuela Prov. [;] Area de Conservacion Arenal [;] Res. San Ramon, Malaise #18 [;] in open area next to main [;] road, VI-26 to VII-14 1998 [;] S. Dadelahi and N. Zitani (ESUW). 1 ♀, Costa Rica: Alajuela, [;] ACA Arenal, R.B. San [;] Ramon, Est. San Ramon [;] 900m, viii.16–30.1998 [;] Malaise, L.J. van der Ent (ESUW). 1 ♀, COSTA RICA: [;] Heredia, Chilamate [;] 75m, xi 1989 [;] Hanson & Godoy (ESUW). 3 ♀♀, Costa Rica: Heredia [;] 3km. S. Puerto Viejo, [;] OTS, La Selva, 100m [;] xii.1992, III-IV-1993 and IV-V-1993, P. Hanson (ESUW). 2 ♀♀, top label - COSTA RICA, Heredia [;] Est. Biol. La Selva, 50- [;] 150m, 10°26'N, 85°01W [;] Jul and Mar 1993, INBio-OET; second label - 1 julio and 02 Marzo 1993 [;] Bosque primario [;] M/07/141 and M/04/018 (ESUW). 4 ♀♀, COSTA RICA-Heredia Prov. [;] La Selva Biological Station [;] 10°26'N, 85°01W, 100m [;] Malaise trap 03, #385, 05, #324, 11, #357 and 06, #347 [;] 4.iv.1994, 15.i.1994, 15.ii.1994 and 3.ii.1994 [;] Project ALAS (ESUW). 1 ♀, Costa Rica: San Jose [;] San Antonio de Escazu [;] 1300m, ix.1998 [;] W. Eberhard (ESUW). 1 ♀, Costa Rica: San Jose [;] Zurqui de Moravia [;] 1600m, February 1996 [;] P. Hanson, Malaise (ESUW). 1 ♀, Costa Rica: Cartago [;] Turrialba CATIE [;] 14–15.iii.1990 [;] 700m, J. S. Noyes (ESUW). 3 ♀♀, Costa Rica: Puntarenas [;] Pen. Osa, Cerro Rincon [;] 200 meters S. del hito [;] 745m el., virgin forest [;] i.1991, Hanson & Quiros [;] ex. Malaise trap (ESUW). 1 ♀, COSTA RICA: [;] Puntar, Golfo Dulce [;] 24km W Piedras Blancas [;] 200m, vi-viii 1989 [;] Hanson (ESUW). 2 ♀♀, Costa Rica: Puntar. [;] P.N. Corcovado [;] Est. Sirena, 50m [;] x-xii 1990 and IV-VIII 1989 (ESUW). 14 ♀♀, Costa Rica: Puntarenas [;] San Vito, Estac. Biol. [;] Las Alturas, 1500m [;] ii.1992, xi.1991, xi.1991, i.1992, and ii-iv.1993 Paul Hanson (ESUW). 2 ♀♀, Costa Rica: Puntarenas [;] San Vito, Estac. Biol. [;] Las Alturas, 1500m [;] v.1992, Forest border, [;] Malaise, Paul Hanson (ESUW). 3 ♀♀, COSTA RICA: Puntar [;] Cerro Rincon, 200m [;] S. hito, 745m, ii. [;] 1991, Hanson/Godoy (ESUW). 3 ♀♀, COSTA RICA: Puntarenas [;] San Vito, Las Cruces [;] 1200msnm, VII-IX 1988 [;] Coll. P. Hanson (ESUW). 1 ♀, Costa Rica, Puntarenas [;] Pen. Osa, 23km. N. Pto. [;] Jimenez, La Pilma, 10m [;] VI-VIII-1993, P. Hanson (ESUW). 3 ♀♀, Costa Rica: Puntarenas, ACO [;] Golfito, Est. Agujas, 250–350m [;] 15.viii–15.ix.1999, J. Azofeifa [;] L.S. 276750-526550 #53264 [;] Malaise trap (ESUW). 2 ♀♀, Costa Rica: Puntarenas [;] Res. Forestal Golfo Dulce [;] 3km SW Rincon, 10m [;] ii.1993, P. Hanson [;] Malaise, primary forest (ESUW). 1 ♀, COSTA RICA: Puntarenas [;] Rd. to Rincon, 10km W. [;] of Pan-Amer. Hwy. 100m [;] III-V 1989, Hanson & Gauld (ESUW). 1 ♀, COSTA RICA: Puntar [;] R.B. Carara, Estac. [;] Bijagoal, 500m [;] X 1989, P. Hanson (ESUW). 2 ♀♀, COSTA RICA: Puntar. [;] R.B. Carara, Estac. [;] Quebrada Bonita, 50m [;] V-VI 1989, P. Hanson (ESUW). 1 ♀, COSTA RICA, Puntarenas [;] San Vito, Jardin Bot. [;] Las Cruces, VII-VIII/88 [;] 1200m, Col. P. Hanson (ESUW). 1 ♀, Costa Rica: Puntarenas, Finca [;] Marco Morales, 600m NE de la [;] Plaza del Progresso, 110–1200m [;] 8.v–28.vi.1995, M. Segura, Malaise [;] L.N. 317750-594800 #5489 (ESUW). 1 ♀, COSTA RICA: Puntarenas [;] Reserva Forestal Golfo Dulce [;] 3km SW of Rincon, 10m [;] November 1992, P. Hanson [;] primary forest, Malaise trap (ESUW). 1 ♀, Costa Rica: Puntarenas [;] ACO, Golfito, RF Golfo Dulce [;] Est. Agujas, 250–300m [;] 15.vii–15.viii.1999, J. Azofeifa [;] L.S. 276750-526550 #53003 (ESUW). 1 ♀, Costa Rica: Puntarenas [;] Est. Sirena, 1–100m [;] i-iii.1990, G. Fonesca [;] L.S. 270500-508300 [;] Malaise trap #7450 (ESIUW). 1 ♀, Costa Rica: Puntarenas [;] R.F.Golfo Dulce, 3km [;] SW Rincon, 10m [;] Malaise-primary forest [;] viii.1991, P. Hanson (ESUW). 1 ♀, COSTA RICA: La Selva [;] 15.XII.1993 [;] J. Longino [;] (M/04/272) (ESUW). 1 ♀, top label - Costa Rica: Guanacaste [;] W. side Volcan Orosi [;] Estac. Maritza, 600m; second label - GNP Biodiversity Survey [;] 1989, Malaise trap [;] L-N-326900-373000 #6834 (ESUW). 2 ♀♀, Costa Rica: Guanacaste [;] Est. Cacao, 100–1150m [;] ix.1996 and vii.1996, I. Villegas and A. Masis, Malaise [;] L.N. 323150-375500 #47559 and 47555 (ESUW). 1 ♀, Sirena, Osa Pen. [;] VII. 77 Cos. Rica [;] D. H. Janzen (AEIC). 1 ♀, S.RosaPark, Guan. [;] C. Rica 16 Sep 77 [;] D.H. Janzen [;] Dry Hill (AEIC). 1 ♀, Costa Rica: Puntarenas [;] San Vito - Las Cruces [;] 5-VI-1988 1200m [;] P. Hanson (TAMU). 1 ♀, Costa Rica: Limon [;] Cahuita Natl Park [;] 29-V-1988 (TAMU). 1 ♀, Costa Rica [;] San Vito, Puntarenas [;] 9 January 1975 [;] M. Palmer (TAMU). 1 ♀, COSTA RICA: Puntarenas [;] San Vito, Estac. Biol. [;] Los Altures 1500m [;] iv.1992 P. Hanson (TAMU). 1 ♀, COSTA RICA: Puntarenas [;] RF Golfo Dulce el 200m [;] 24km W Piedras Blancas [;] P. Hanson xii.1992 (TAMU). 4 ♀♀, COSTA RICA: [;] 11 mi. from Turrialba [;] “Los Esperales”, C.A.T.I.E. [;] 5-II-1985 [;] P. Stanley (TAMU).

#### Comments.

This species is very similar to *Heterospilus spiloheterus* but is distinguished by the granulate vertex with distinct transverse rugae behind the ocelli. Included in the type series are many specimens with a lighter colored mesoscutum which may be a separate species after more study.

#### Etymology.

Named for my colleague and eminent Russian braconidologist, Sergey Belokobylskij.

**Figure 191.**
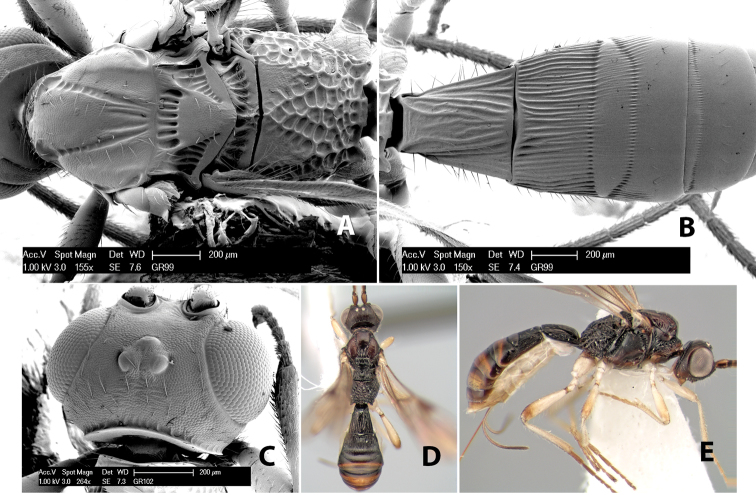
*Heterospilus sergeyi* Marsh, sp. n.: **A–C** paratype **D–E** holotype.

### Heterospilus sharkeyi

Marsh sp. n.

http://zoobank.org/CEC0A0AC-55EA-4B25-B51E-2E783E7FBEFF

http://species-id.net/wiki/Heterospilus_sharkeyi

Figure 192

#### Female.

Body size: 3.0–3.5 mm. Color: head dark brown; scape dark brown, flagellum brown with apical white annulus, apical 5–7 flagellomeres brown; mesosoma dark brown, propodeum, mesopleuron and venter often lighter brown; metasomal terga dark brown; wing veins including stigma brown; legs yellow. Head: vertex granulate, usually with distinct transverse rugae behind antennae; frons granulate; face granulate-rugose; temple in dorsal view narrow, sloping behind eye, width less than 1/2 eye width; malar space equal to 1/4 eye height; ocell-ocular distance about 1.5 times diameter of lateral ocellus; 25–29 flagellomeres. Mesosoma: mesoscutal lobes granulate; notauli scrobiculate, meeting at scutellum in triangular rugose area; scutellum granulate; prescutellar furrow with 3 cross carinae; mesopleuron granulate; precoxal sulcus smooth, shorter than mesopleuron; venter granulate; propodeum with basal median areas margined, granulate, basal median carina absent, areola not distinctly margined, areolar area rugose, lateral areas entirely rugose, propodeum often with small tubercle above hind coxa near base of petiole. Wings: fore wing vein r shorter than vein 3RSa, vein 1cu-a beyond vein 1M; hind wing vein SC+R present, vein M+CU shorter than vein 1M. Metasoma: first tergum longitudinally costate, length equal to apical width; second tergum longitudinally costate, with distinct raised semi-circular smooth area medially at base; anterior transverse groove present, straight; posterior transverse groove present; third tergum entirely smooth except for costate posterior transverse groove; terga 4–7 smooth; ovipositor as long as metasomal tergum 1.

#### Holotype female.

Top label (white, partially printed and hand written) - Costa Rica: Guanacaste [;] Santa Rosa Natl. Park [;] 300m, ex. Malaise trap [;] Site #: (blank) [;] Dates: 18.x–8.xi.1986 [;] I.D. Gauld & D. Janzen; second label (white, printed) - [SE] Bosque Emilio [;] 50yr old deciduous forest [;] [O] in clearing, fully [;] isolated part of day; third label (red, partially printed and hand written) - HOLOTYPE [;] Heterospilus [;] sharkeyi [;] P. Marsh. Deposited in ESUW.

#### Paratypes.

2 ♀♀, same data as holotype with additional dates of 26.x–16.xi.1985 and 6–27.x.1986 (ESUW). 1 ♀, top label - Costa Rica: Guanacaste, Santa [;] Rosa Nat’l Park, Bosque San [;] Emilio, trap #5 in clearing, 300m. [;] XI/8–29/1986, I. Gauld; second label - [SE] Bosque Emilio [;] 50yr old deciduous forest [;] [O] in clearing, fully [;] isolated part of day (ESUW). 2 ♀♀, top label - Costa Rica: Guanacaste [;] Santa Rosa Natl. Park [;] 300m, ex. Malaise trap [;] Site #: H-2-C [;] Dates: 5–26.vii.1986 and 6–27.ix.1986 [;] I.D. Gauld & D. Janzen; second label - [H] open regenerating [;] woodland <10 years old [;] [C] more or less fully [;] shaded as possible (ESUW). 1 ♀, top label - Costa Rica: Guanacaste [;] Santa Rosa Natl. Park [;] 300m, ex. Malaise trap [;] Site #: blank [;] Dates: 27.ix–18.x.1986 [;] I.D. Gauld & D. Janzen; second label - [H] open regenerating [;] woodland <10 years old [;] [O] in clearing, fully [;] isolated pert of day (ESUW). 1 ♀, Costa Rica: Guanacaste [;] Est. Cacao, 1000–1150m [;] viii.1996, M. Pereira [;] L.N. 323150-375500 347561 [;] Malaise trap (ESUW). 1 ♀, top label - Costa Rica: Guanacaste [;] Santa Rosa National Pk. [;] 300m, Malaise, Ian Gauld [;] 10–31.i.1987; second label - Bosque Humedo [;] Mature dry forest [;] high proportion [;] Evergreen species [;] Sun; third label - BH-11-O [;] 10–31.i.87 (ESUW). 1 ♀, Costa Rica: Guanacaste, Bagaces [;] Pque. Ntl. Palo verde, Sct. P. Verde [;] Cerro, Guayacan, 212m, Malaise [;] 13.x–11.xi.1999, I. Jimenez [;] L.N. 259350-389600 #54006 (ESUW). 6 ♀♀, Costa Rica: Heredia [;] 3km. S. Puerto Viejo, [;] OTS, La Selva, 100m [;] xi.1992 and xii.1992, P. Hanson (ESUW). 1 ♀, Costa Rica: Heredia [;] Puerto Viejo, [;] OTS. La Selva, 100m [;] iv.1991, P. Hanson (ESUW). 1 ♀, Costa Rica, Heredia [;] 3km. S. Pto. Viejo [;] OTS-LaSelva, 100m [;] X-1992, P. Hanson (ESUW). 1 ♀, Costa Rica, Heredia Prov. [;] OTS, La Selva, 100m [;] 1993 II-III, P. Hanson (ESUW). 2 ♀♀, COSTA RICA-Heredia Prov. [;] La Selva Biological Station [;] 10°26'N, 84°01'W, 100m [;] Malaise trap 01, #320 [;] 15.i.1994 and 1.xi.1993 (ESUW). 3 ♀♀, COSTA RICA: Puntarenas [;] Rd. to Rincon, 10km W. [;] of Pan-Amer. Hwy, 100m [;] III-V 1989, Hanson & Gauld (ESUW). 1 ♀, Costa Rica: Puntarenas [;] Golfo Dulce, 24km W. [;] Piedras Blancas, 200m [;] iv.1993, Paul Hanson (ESUW). 1 ♀, COSTA RICA: Puntar [;] Golfo Dulce, 10km W [;] Piedras Blancas, 100m [;] VI-VIII 1989, Hanson (ESUW). 1 ♀, COSTA RICA, Alajuela [;] Inst. Tec. SantaClara [;] 150m, 24/III/1989 [;] col. Hanson & Godoy (ESUW). 1 ♀, Costa Rica: Limon [;] Sector Cocori, 100m [;] 30km N Cariari, i.1995 [;] E. Rojas, Malaise #4526 [;] L.N. 286000-567500 (ESUW).

#### Comments.

The distinctly granulate head, white annulus on the flagellum, bicolored mesosoma and the smooth raised area medially at the base of metasomal tergum 2 are distinctive for this species.

#### Etymology.

Named for my long time friend, colleague and fellow braconidologist, Michael Sharkey.

**Figure 192.**
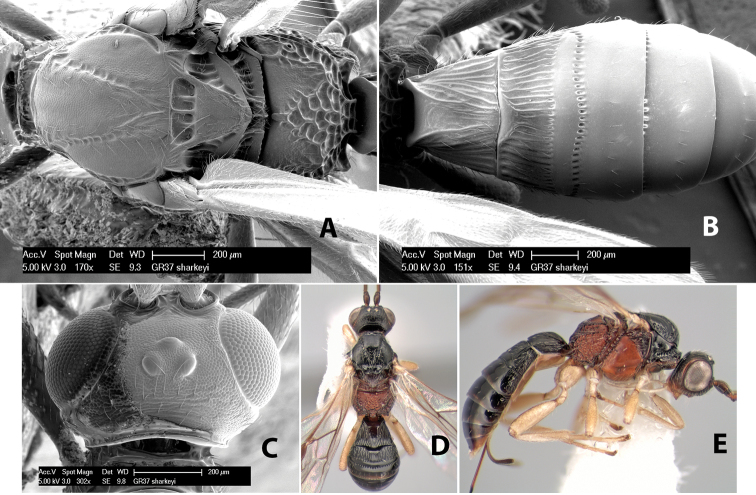
*Heterospilus sharkeyi* Marsh, sp. n.: **A–C** paratype **D–E** holotype.

### Heterospilus spiloheterus

Marsh sp. n.

http://zoobank.org/8A9527ED-3AD3-42D0-9F6A-80F4631B407A

http://species-id.net/wiki/Heterospilus_spiloheterus

Figure 193

#### Female.

Body size: 3.0–3.5 mm. Color: body dark brown or black, apical metasomal terga lighter brown or yellow; scape yellow with lateral longitudinal brown stripe, flagellum brown with apical white annulus, apical 5–7 flagellomeres brown; wing veins including stigma brown; legs yellow, apical ⅓ of femora light to medium brown. Head: vertex granulate; frons granulate; face granulate; temple in dorsal view narrow, sloping behind eye, width less than 1/2 eye width; malar space equal to 1/4 eye height; ocell-ocular distance 2.0–2.5 times diameter of lateral ocellus; 23–25 flagellomeres. Mesosoma: mesoscutal lobes granulate; notauli weakly scrobiculate, often smooth posteriorly; scutellum granulate; prescutellar furrow with 5 cross carinae; mesopleuron granulate; precoxal sulcus smooth, shorter than mesopleuron; venter granulate; propodeum with basal median areas margined, granulate, basal median carina absent, areola not margined, areolar area areolate-rugose, lateral areas entirely rugose, propodeum with distinct tubercle above hind coxa at base of petiole. Wings: fore wing vein r shorter than vein 3RSa, vein 1cu-a beyond vein 1M; hind wing vein SC+R absent, vein M+CU shorter than vein 1M. Metasoma: first tergum longitudinally costate, length greater than apical width; second tergum longitudinally costate; anterior transverse groove present, straight; posterior transverse groove present; third tergum weakly costate basally, smooth apically, occasionally entirely smooth; terga 4–7 smooth; ovipositor half as long as metasoma.

#### Holotype female.

Top label (white, printed) - COSTA RICA: Puntarenas [;] Rd. to Rincon, 10km W. [;] of Pan-Amer. Hwy. 100m [;] III-V 1989, Hanson & Gauld; second label (red, partially printed and hand written) - HOLOTYPE [;] Heterospilus [;] spiloheterus [;] P. Marsh. Deposited in ESUW.

#### Paratypes.

1 ♀, same data as holotype except, 25km W of pan-Amer. Hwy, 200m (ESUW). 13 ♀♀, Costa Rica, Puntarenas [;] R.F. Golfo Dulce, [;] 3km SW. Rincon, 10m, [;] vi.1991, Oct. 1991 and xii.1992 (ESUW). 2 ♀♀, COSTA RICA: Puntar [;] Golfo Dulce, 3km [;] S.W. Rincon, 10m [;] IX-XI 1989, Hanson (ESUW). 2 ♀♀, COSTA RICA: Puntar [;] Golfo Dulce, 10km W [;] Piedras Blancas, 100m [;] VI-VIII 1989, Hanson (ESUW). 1 ♀, Costa Rica: Puntarenas [;] Golfo Dulce, 24km W. [;] Piedras Blancas, 200m [;] ii.1993, Paul Hanson (ESUW). 1 ♀, COSTA RICA: Puntar [;] Golfo Dulce, 24km W [;] Piedras Blancas [;] 200m, vii-ix 1990 [;] Col. Paul Hanson (ESUW). 1 ♀, COSTA RICA: [;] Puntar. Golfo Dulce [;] 24km W Piedras Blancas [;] 200m, vi-viii 1989 [;] Hanson (ESUW). 1 ♀, COSTA RICA: Puntar [;] Golfo Dulce 3km SW [;] Rincon [;] 10m, vii-ix 1990 [;] Col. Paul Hanson (ESUW). 1 ♀, COSTA RICA: Puntarenas [;] 3km S. Rincon, 10m [;] II-III 1989 [;] P. Hanson & I. Gauld (ESUW). 1 ♀, COSTA RICA: [;] Puntar [;] Golfo Dulce, 3km [;] SW. Rincon, 10m [;] VI-VIII 1989, Hanson (ESUW). 2 ♀♀, COSTA RICA: Puntarenas [;] Reserva Forestal Golfo Dulce [;] 3kmSW Rincon, 10m, primary [;] forest, xii.1992, P. Hanson (ESUW). 1 ♀, COSTA RICA: Puntar [;] P.N. Corcovado, Est [;] Sirena, 50m [;] IV-VIII 1989 (ESUW). 1 ♀, Costa Rica: Puntarenas [;] Res. Forestal Golfo Dulce [;] 3km. SW Rincon, 10m [;] xii.1992, P. Hanson [;] Malaise, primary forest (ESUW). 2 ♀♀, COSTA RICA: Puntar [;] R.B. Carara, Estac. [;] Quebrada Bonita, 50m [;] vii-ix 1989, Hanson (ESUW). 2 ♀♀, Costa Rica, Heredia Prov. [;] OTS, La Selva, 100m [;] 1993 II-III P. Hanson (ESUW). 5 ♀♀, Costa Rica, Heredia [;] 3km. S. Puerto Viejo [;] OTS-La Selva, 100m [;] I-II-1993, P. Hanson (ESUW). 1 ♀, Costa Rica: Heredia [;] 3km. S. Puerto Viejo, [;] OTS, La Selva, 100m [;] xii.1992, P. Hanson (ESUW). 1 ♀, COSTA RICA, Heredia [;] Chilamate, 75m [;] 25/III/1989 [;] col. Hanson & Godoy (ESUW). 1 ♀, COSTA RICA: San Jose [;] P.N. Braulio Carillo [;] 9.5km E tunnel, 1000m [;] x-xii 1989, P. Hanson (ESUW). 3 ♀♀, COSTA RICA: Limon [;] P.N. Tortuguero [;] Est. 4-esquinas, 0m [;] iv-v 1989 and VI-VIII 1989, Solano (ESUW). 8 ♀♀, COSTA RICA: Limon [;] 16km W. Guapiles [;] 400m, i-iv.1991, vii-ix 1990, v-vi 1990, III/1989, II/1989 and iii-v 1990 [;] col. Paul Hanson (ESUW). 1 ♀, COSTA RICA: Limón [;] 7km SW Bribri, 50m [;] I-II 1990 [;] Col. Paul Hanson (ESUW). 1 ♀, COSTA RICA: Limon [;] 4km NE Bribri [;] 50m, IX-XI 1989 [;] col. Paul Hanson (ESUW). 1 ♀, Sirena, Osa Pen. [;] VII.77 Cos. Rica [;] D. H. Janzen (AEIC). 1 ♀, COSTA RICA: *Punt-* [;] *arenas*. 7km SW Rincon [;] 31.v–7.vi.1998; B. Brown [;] & V. Berezovskiy; Mal. [;] Trp. #5:2nd growth (AEIC). 1 ♀, top label - COSTA RICA, Heredia: [;] Est. Biol. La Selva, 50- [;] 150m, 10°26'N, 84°01'W [;] Oct 1995, INBio-OET; second label - 16 Octubre 1995 [;] M/07/476 [;] Bosque primario (INBC). 1 ♀, top label - COSTA RICA, Heredia: [;] Est. Biol. La Selva, 50- [;] 150m, 10°26'N, 84°01'W [;] Feb 1998, INBio-OET; second label - 18 Febrero 1998 [;] M/18/702 [;] Borde suampo (INBC). 2 ♀♀, COSTA RICA, Limon [;] sue de Iriquois [;] 300m, 23/V/1987 [;] Col. Paul Hanson (MICR). 2 ♀♀, COSTA RICA, Puntar. [;] Golfo Dulce, 24km W. [;] PiedrasBlancas, 200m [;] XII-89-III-90 and III-VI-90 Hanson (MICR). 1 ♀, COSTA RICA, Puntar [;] San Vito, Las Tables [;] 1600m 10-III 89 (MICR). 1 ♀, Costa Rica [;] San Vito, Puntarenas [;] 19 December 1974 [;] M. Palmer (TAMU). 1 ♀, COSTA RICA: [;] 11 mi. from Turrialba [;] “Los Esperales”, C.A.T.I.E. [;] 5-II-1985 [;] P. Stansly (TAMU). 1 ♀, Costa Rica: Puntarenas [;] San Vito - Las Cruces [;] 5-VI-1988 1200m [;] P. Hanson (TAMU).

#### Comments.

This species is very similar to *Heterospilus sergeyi* but is distinguished by the entirely granulate vertex.

#### Etymology.

The specific name is an anagram of *Heterospilus*.

**Figure 193.**
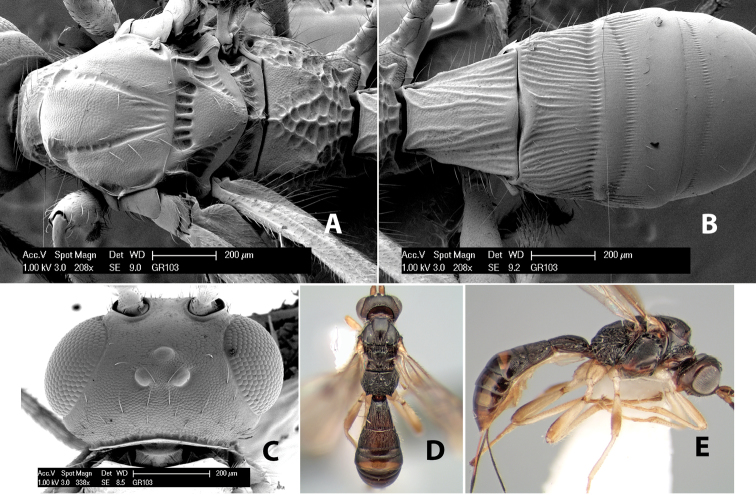
*Heterospilus spiloheterus* Marsh, sp. n.: **A–C** paratype **D–E** holotype.

### Heterospilus strazanaci

Marsh sp. n.

http://zoobank.org/60089A60-68C3-4D8C-81B8-7F32E0A545B0

http://species-id.net/wiki/Heterospilus_strazanaci

Figure 194

#### Female.

Body size: 2.5 mm. Color: body dark brown, metasomal terga 4–7 light brown to yellow; scape yellow with lateral longitudinal brown stripe, flagellum brown with apical white annulus, apical 2–3 flagellomeres brown; wing veins including stigma brown; legs yellow, femora usually brown on apical half. Head: vertex granulate; frons granulate; face weakly granulate or smooth; temple in dorsal view narrow, sloping behind eye, width less than 1/2 eye width; malar space slightly greater than 1/4 eye height; ocell-ocular distance 2.0–2.5 times diameter of lateral ocellus; 18–20 flagellomeres. Mesosoma: mesoscutal lobes granulate; notauli weakly scrobiculate anteriorly, smooth posteriorly; scutellum granulate; prescutellar furrow with 3–5 cross carinae; mesopleuron granulate; precoxal sulcus smooth, shorter than mesopleuron; venter granulate; propodeum with basal median areas margined, granulate, basal median carina absent, areola not margined, areolar area rugose, lateral areas entirely rugose, propodeum with small tubercle above hind coxa near base of petiole. Wings: fore wing vein r shorter than vein 3RSa, vein 1cu-a beyond vein 1M; hind wing vein SC+R absent, vein M+CU shorter than vein 1M. Metasoma: first tergum longitudinally costate, granulate medially, length nearly twice apical width; second tergum longitudinally costate; anterior transverse groove present, straight; posterior transverse groove present, slightly curved anteriorly at sides; third tergum entirely smooth except for costate transverse groove; terga 4–7 smooth; ovipositor as long as metasomal tergum 1.

#### Holotype female.

Top label (white, printed) - COSTA RICA: Limon [;] 16km W. Guapiles [;] 400m, i-iv.1991 [;] col. Paul Hanson; second label (red, partially printed and hand written) - HOLOTYPE [;] Heterospilus [;] strazanaci [;] P. Marsh. Deposited in ESUW.

#### Paratypes.

1 ♀, Costa Rica: Puntarenas, ACO [;] Golfito, P.N. Corcovado [;] Est. Agujas, Cerro Rincon [;] 600–745m, Malaise trap [;] 15.viii–15.ix.1999, J. Azofeifa [;] L.S. 275500-521950 #53268 (ESUW). 1 ♀, top label - Costa Rica: Guanacaste [;] 9km S. Santa Cecilia [;] Estacion Pitilia, 700m [;] vi.1996, Malaise trap; second label - C. Moraga & P. Rios [;] L.N. 330200-380200 [;] #47562 (ESUW). 1 ♀, Costa Rica: Heredia [;] Braulio Carrillo N.P. [;] 250–500m iv.10.85 [;] Henri Goulet (AEIC). 1 ♀, COSTA RICA, Limon [;] sue de Iriquois [;] 300m, 23/V/1987 [;] Col. Paul Hanson (MICR). 1 ♀, COSTA RICA, Puntarenas [;] San Vito, Jardin Bot. [;] Las Cruces, VI-VII/88 [;] 1200m, col. P. Hanson (MICR). 1 ♀, COSTA RICA, San Jose [;] P.N. Braulio Carrillo [;] 9km NE tunnel, 1100m [;] 15/V/89, Col. Hanson (MICR). 1 ♀, COSTA RICA, Guanac [;] Est. Pitilia, 9km S [;] SantaCecilia, 700m [;] IX 1988 P. Hanson (MICR). 2 ♀♀, Costa Rica: San Jose [;] Braulio Carillo N. P. [;] 8.2km E tunnel [;] 15-V-1988 P. Hanson (TAMU).

#### Comments.

The absence of hind wing vein SC+R, the long and narrow first metasomal tergum and the short ovipositor are distinctive for this species.

#### Etymology.

Named for my long time friend and colleague, John Strazanac.

**Figure 194.**
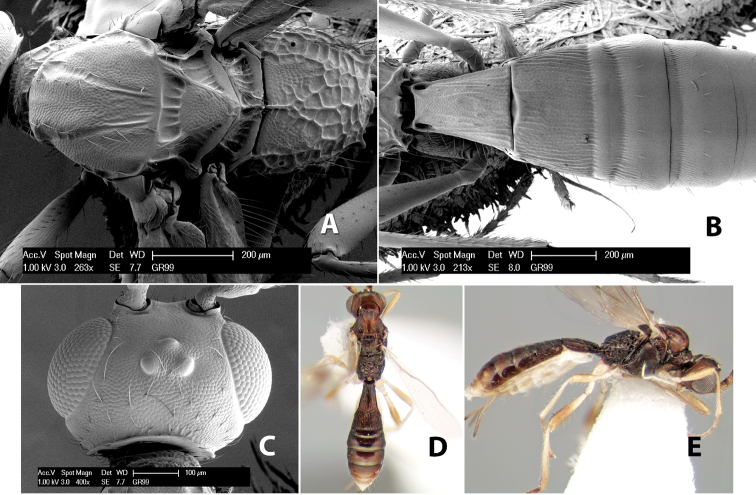
*Heterospilus strazanaci* Marsh, sp. n.: **A–C** paratype **D–E** holotype.

### Heterospilus sumo

Marsh sp. n.

http://zoobank.org/1308312F-6719-4B0E-8185-E576ABE0AD4E

http://species-id.net/wiki/Heterospilus_sumo

Figure 195

#### Female.

Body size: 4.0 mm. Color: body brown; scape brown, flagellum brown with white apical annulus, apical 10–12 flagellomeres white; wing veins including stigma light brown; legs yellow. Head: vertex granulate with transverse rugae behind ocelli; frons granulate; face granulate-rugose; temple in dorsal view narrow, width less than 1/2 eye width; malar space slightly greater than 1/4 eye height; ocell-ocular distance twice diameter of lateral ocellus; 32 flagellomeres. Mesosoma: mesoscutal lobes granulate; notauli strongly scrobiculate, meeting at scutellum in triangular rugose area, rugosity extending anteriorly onto median lobe; scutellum weakly granulate or smooth; prescutellar furrow with 3 cross carinae; mesopleuron granulate; precoxal sulcus smooth, shorter than mesopleuron; venter smooth; propodeum with basal median areas not distinct from rest of propodeum, basal median carina absent, areola not margined, areolar area areolate, lateral areas entirely rugose, propodeum with tubercle just above hind coxa at base of petiole. Wings: fore wing vein r shorter than vein 3RSa, vein 1cu-a beyond vein 1M; hind wing vein SC+R present, vein M+CU shorter than vein 1M. Metasoma: first tergum longitudinally costate-rugose, length equal to apical width; second tergum longitudinally costate, costae angled toward median line, base of tergum with median raised semicircular granulate area; anterior transverse groove present, straight; posterior transverse groove present; third tergum costate basally, smooth apically; terga 4–7 granulate or costate at base, smooth at apex; ovipositor about 1/2 length of metasoma.

#### Holotype female.

Top label (white, printed) - Costa Rica: Heredia [;] Puerto Viejo [;] OTS, La Selva, 100m [;] iv.1991, P. Hanson; second label (red, partially printed and hand written) - HOLOTYPE [;] Heterospilus [;] sumo [;] P. Marsh. Deposited in ESUW.

#### Paratypes.

Known only from the holotype.

#### Comments.

This species is similar to *Heterospilus villegasi* but distinguished by the sculpturing on metasomal tergum 2 and the slightly weaker granulation of the mesoscutum.

#### Etymology.

Named for the Sumo, an indigenous people of Nicaragua.

**Figure 195.**
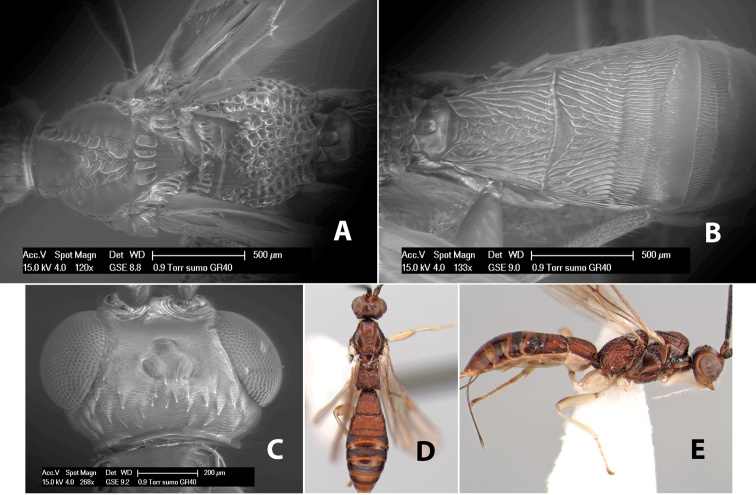
*Heterospilus sumo* Marsh, sp. n., holotype.

### Heterospilus tricolor

Marsh sp. n.

http://zoobank.org/0DB10186-8AC1-4B71-8B27-953AA1090458

http://species-id.net/wiki/Heterospilus_tricolor

Figure 196

#### Female.

Body size: 3.0–3.5 mm. Color: head dark brown, yellow spots on face just below antennae; scape yellow, brown laterally, flagellum brown with apical white annulus, apical 3–5 flagellomeres brown; mesosoma dark brown, mesoscutum lighter brown; metasoma dark brown, terga 2 and 5–7 yellow; wing veins brown, stigma light brown or honey yellow; legs yellow. Head: vertex granulate; frons granulate; face granulate; temple in dorsal view narrow, sloping behind eye, width lass than 1/2 eye width; malar space greater than 1/4 eye height; ocell-ocular distance 2–2.5 times diameter of lateral ocellus; 28–29 flagellomeres. Mesosoma: mesoscutal lobes granulate; notauli scrobiculate, meeting at scutellum in triangular costate area; scutellum granulate; prescutellar furrow with 5–7 cross carinae; mesopleuron granulate; precoxal sulcus smooth, shorter than mesopleuron; venter granulate; propodeum with basal median areas distinct and margined, sometimes indistinctly margined, granulate, basal median carina absent, areola not distinct, areolar area areolate-rugose, lateral areas entirely rugose, propodeum with weak but distinct tubercle above hind coxa, sometimes very weakly indicated. Wings: fore wing vein r shorter than vein 3RSa, vein 1cu-a beyond vein 1M; hind wing vein SC+R absent, vein M+CU shorter than vein 1M. Metasoma: first tergum longitudinally costate laterally, rugose medially, length equal to or slightly greater than apical width; second tergum longitudinally costate; anterior transverse groove present, straight; posterior transverse groove present; third tergum entirely granulate except for costate transverse groove; terga 4–7 granulate; ovipositor shorter than first tergum.

#### Holotype female.

Top label (white, printed) - Costa Rica: Puntarenas [;] Pen. Osa, Cerro Rincon [;] 200 meters S. del hito [;] 745m el., virgin forest [;] i.1991, Hanson & Quiros [;] ex. Malaise trap; second label (red, partially printed and hand written) - HOLOTYPE [;] Heterospilus [;] tricolor [;] P. Marsh. Deposited in ESUW.

#### Paratypes.

1 ♀, Costa Rica: Puntarenas [;] Est. Altmira, 1300–1450m [;] 1km S del Cerro Biolley [;] 23.viii–13.ix.1996, Malaise [;] L.S. 331700-572100 #44870 [;] R. Vollalobos (ESUW). 2 ♀♀, Costa Rica: Puntarenas [;] San Vito, Estac. Biol. [;] Las Alturas, 1500m [;] xii.1991, Paul Hanson (ESUW).

#### Comments.

The dark brown body with the brown mesoscutum and the yellow metasomal terga 2 and 5–7 are distinctive for this species.

#### Etymology.

The specific name is in reference to the tricolored dark brown body with brown mesoscutum and yellow metasomal terga 2 and 5–7.

**Figure 196.**
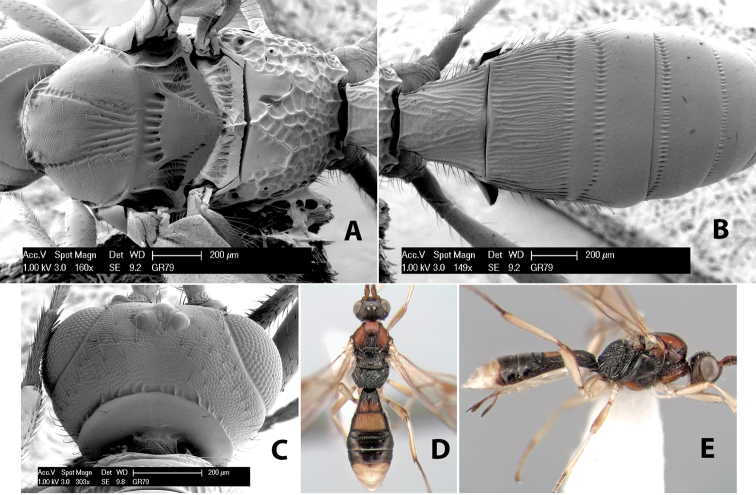
*Heterospilus tricolor* Marsh, sp. n.: **A–C** paratype **D–E** holotype.

### Heterospilus tzutujil

Marsh sp. n.

http://zoobank.org/829248BB-0CF6-48B0-8BA2-EB0B8D5C5A07

http://species-id.net/wiki/Heterospilus_tzutujil

Figure 197

#### Female.

Body size: 3.5 mm. Color: head dark brown; scape yellow with lateral longitudinal brown stripe, flagellum brown with apical white annulus, apical 3–5 flagellomeres brown; mesosoma with mesoscutum, pronotum and mesopleuron dark brown, propleuron and propodeum lighter brown; metasoma brown, terga 2 and 4–7 lighter brown; wing veins including stigma brown; legs yellow. Head: vertex granulate; frons granulate; face granulate; temple in dorsal view narrow, sloping behind eye, width less than 1/2 eye width; malar space equal to 1/4 eye height; ocell-ocular distance about twice diameter of lateral ocellus; 29–31 flagellomeres. Mesosoma: mesoscutal lobes granulate; notauli scrobiculate anteriorly, smooth posteriorly, meeting at scutellum in triangular costate area; scutellum weakly granulate; prescutellar furrow with 5 cross carinae; mesopleuron granulate; precoxal sulcus smooth, shorter than mesopleuron; venter granulate; propodeum with basal median areas margined, granulate, basal median carina absent, areola distinctly margined, areolar area with numerous distinct cross carinae, lateral areas rugose, propodeum with distinct tubercle above hind coxa at base of petiole. Wings: fore wing vein r shorter than vein 3RSa, vein 1cu-a beyond vein 1M; hind wing vein SC+R absent, vein M+CU shorter than vein 1M. Metasoma: first tergum longitudinally costate, length slightly greater than apical width; second tergum longitudinally costate; anterior transverse groove present, straight; posterior transverse groove present; third tergum smooth except for costate transverse groove; terga 4–7 smooth; ovipositor equal to half length of metasoma.

#### Holotype female.

Top Label (white, printed) - Costa Rica: Puntarenas [;] San Vito, Las Cruces [;] Wilson Botanical Gardens [;] 18–22.iii.1990, 1150m [;] J.S. Noyes; second label (red, partially printed and hand written) - HOLOTYPE [;] Heterospilus [;] tzutujil [;] P. Marsh. Deposited in ESUW.

#### Paratypes.

Known only from the holotype.

#### Comments.

The propodeal areola with strong cross carinae and the tubercles on the propodeum near the base of the petiole are distinctive for this species.

#### Etymology.

Named for the Tz’utujil, an indigenous Mayan people of Guatemala.

**Figure 197.**
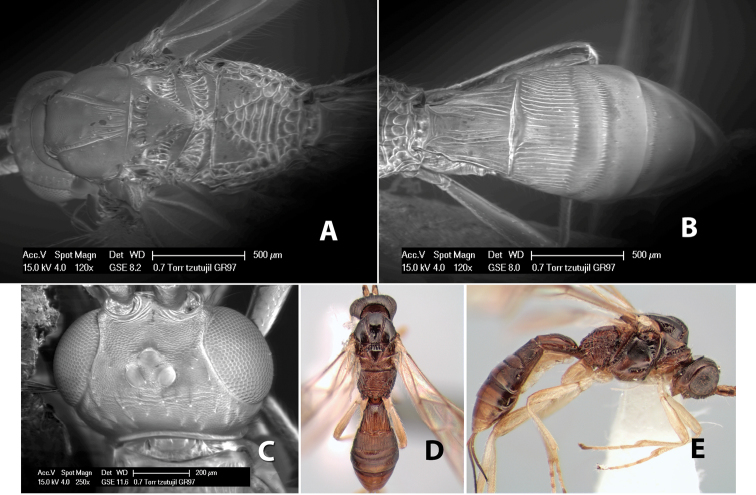
*Heterospilus tzutujil* Marsh, sp. n., holotype.

### Heterospilus ugaldei

Marsh sp. n.

http://zoobank.org/1E19BDE8-1A58-4E0F-B02C-98B314801E13

http://species-id.net/wiki/Heterospilus_ugaldei

Figure 198

#### Female.

Body size: 3.0–3.5 mm. Color: head yellow, sometimes darker along occipital carina; scape yellow with lateral longitudinal brown stripe, flagellum brown with apical white annulus, apical 3–5 flagellomeres brown; mesosoma dark brown, propodeum occasionally lighter brown; metasomal terga dark brown entirely or terga 1, 2 and 4–7 lighter brown; wing veins brown, stigma bicolored brown with yellow at base; legs yellow. Head: vertex granulate, often with transverse rugae behind ocelli; frons granulate-striate; face granulate-rugose; temple in dorsal view narrow, sloping behind eye, width less than 1/2 eye width; malar space greater than 1/4 eye height; ocell-ocular distance about twice diameter of lateral ocellus; 23–25 flagellomeres. Mesosoma: mesoscutal lobes granulate; notauli scrobiculate, meeting at scutellum in triangular costate area; scutellum granulate; prescutellar furrow with 3–5 cross carinae; mesopleuron granulate; precoxal sulcus scrobiculate, shorter than mesopleuron; venter granulate; propodeum with basal median areas margined, granulate, basal median carina absent, areola not margined, areolar area rugose, lateral areas rugose with small granulate area anteriorly, propodeum with small but distinct tubercle above hind coxa at base of petiole. Wings: fore wing vein r nearly as long as vein 3RSa, vein 1cu-a beyond vein 1M; hind wing vein SC+R present, vein M+CU shorter than vein 1M. Metasoma: first tergum longitudinally costate, length equal to apical width; second tergum longitudinally costate; anterior transverse groove present, straight; posterior transverse groove present; third tergum costate basally, smooth apically; terga 4–7 smooth; ovipositor as long as metasomal terga 1 and 2 combined.

#### Holotype female.

Top label (white, partially printed and hand written) - Costa Rica: Guanacaste [;] Santa Rosa National Pk. [;] 300m, Malaise, Ian Gauld [;] 10–31.i.1987; second label (white, partially printed and hand written) - Bosque Humedo [;] Mature dry forest [;] high proportion [;] Evergreen species [;] SUN; third label (white, printed) - BH-9-O [;] 10–31.i.87; fourth label (red, partially printed and hand written) - HOLOTYPE [;] Heterospilus [;] ugaldei [;] P. Marsh. Deposited in ESUW.

#### Paratypes.

1 ♀, same data as holotype (ESUW).

#### Comments.

The yellow head and white annulus on the flagellum are distinctive for this species.

#### Etymology.

Named for Jesus Ugalde in recognition for his hospitality during my several visits to INBio.

**Figure 198.**
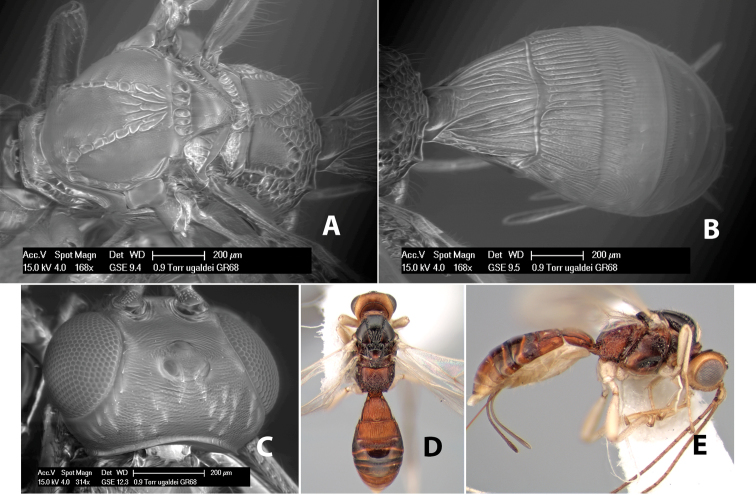
*Heterospilus ugaldei* Marsh, sp. n., holotype.

### Heterospilus villegasi

Marsh sp. n.

http://zoobank.org/5EF2A225-CD99-4CDD-9EA9-B5F775C57D4F

http://species-id.net/wiki/Heterospilus_villegasi

Figure 199

#### Female.

Body size: 3.0 mm. Color: head light brown to brown; scape brown, flagellum brown with white annulus apically, apical 3–5 flagellomeres brown; mesosoma brown, lighter along notauli and upper portion of mesopleuron; metasomal terga brown to dark brown; wing veins including stigma brown; legs yellow, hind femur darker brown on apical half. Head: vertex granulate with weak transverse rugae behind ocelli; frons granulate; face granulate; temple in dorsal view narrow, sloping behind eye, width less than 1/2 eye width; malar space greater than 1/4 eye height; ocell-ocular distance about twice diameter of lateral ocellus; 21–14 flagellomeres. Mesosoma: mesoscutal lobes granulate; notauli scrobiculate, meeting at scutellum in wide nearly rectangular rugose area; scutellum granulate; prescutellar furrow with 3–5 cross carinae; mesopleuron granulate; precoxal sulcus smooth, shorter than mesopleuron; venter granulate; propodeum with basal median areas small but not distinctly margined, granulate, basal median carina absent, areola not distinct, areolar area areolate-rugose, lateral areas entirely rugose. Wings: fore wing vein r shorter than vein 3RSa, vein 1cu-a beyond vein 1M; hind wing vein SC+R present, vein M+CU shorter than vein 1M. Metasoma: first tergum longitudinally costate, length equal to or slightly greater than apical width; second tergum longitudinally costate; anterior transverse groove present, straight; posterior transverse groove present; third tergum costate at base, smooth apically; terga 4–7 smooth; ovipositor equal to half length of metasoma.

#### Holotype female.

Top label (white, printed) - Costa Rica: Guanacaste [;] Est, Cacao, 1000–1150m [;] ix.1996, I. Villegas, Malaise [;] L.N. 323150-375500 #47559; second label (red, partially printed and hand written) - HOLOTYPE [;] Heterospilus [;] villegasi [;] P. Marsh. Deposited in ESUW.

#### Paratypes.

2 ♀♀, same data as holotype except dates of vii.1996, collector A. Masis (ESUW). 1 ♀, Costa Rica: Guanacaste Prov. [;] Guanacaste Conservation Area [;] below Cacao, 400–600m el. [;] 3 March 1990. J.S. Noyes (ESUW).

#### Comments.

This species is similar to *Heterospilus sumo* but differs in the smaller size, sculpturing on metasomal tergum 2 and the coarser granulate mesoscutum.

#### Etymology.

Named for the collector of some of the type series, I. Villegas.

**Figure 199.**
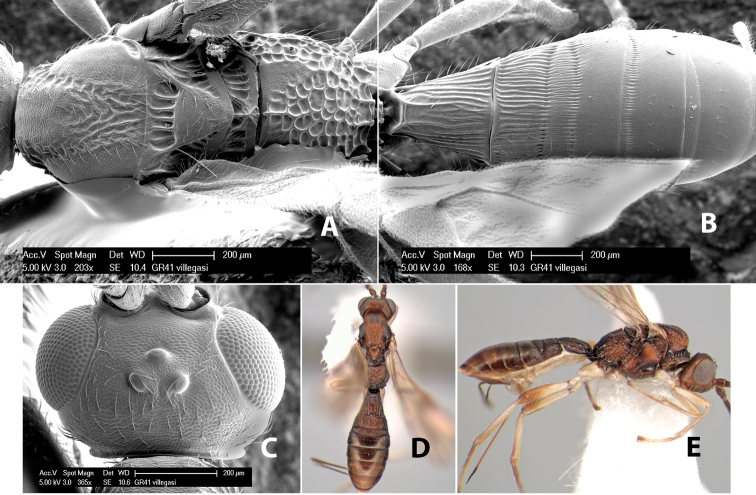
*Heterospilus villegasi* Marsh, sp. n.: **A–C** paratype **D–E** holotype.

### Heterospilus vulcanus

Marsh sp. n.

http://zoobank.org/5F078736-592B-4013-BE6D-2B40DF5E3B37

http://species-id.net/wiki/Heterospilus_vulcanus

Figure 200

#### Female.

Body size: 3.0–3.5 mm. Color: body dark brown, metasomal terga 2 medially and terga 5–7 yellow; scape yellow with lateral longitudinal brown stripe, flagellum brown with apical whiter annulus, apical 5–7 flagellomeres brown; wing veins including stigma brown; legs yellow, apical half of fore and middle femora and apical ⅓ of hind femur brown. Head: vertex granulate; frons granulate; face granulate; temple in dorsal view narrow, sloping behind eye, width less than 1/2 eye width; malar space equal to or slightly greater than 1/4 eye height; ocell-ocular distance about 2.5 times diameter of lateral ocellus; 22–25 flagellomeres. Mesosoma: mesoscutal lobes granulate; notauli weakly scrobiculate or partially smooth, meeting at scutellum in triangular costate area; scutellum granulate; prescutellar furrow with 3–5 cross carinae; mesopleuron granulate; precoxal sulcus scrobiculate, shorter than mesopleuron; venter granulate; propodeum with basal median areas distinct but not margined, granulate, basal median carina absent, areola not margined, areolar area rugose, lateral areas entirely rugose, propodeum with distinct tubercle above hind coxa. Wings: fore wing vein r shorter than vein 3RSa, vein 1cu-a interstitial with or very slightly beyond vein 1M; hind wing vein SC+R absent, vein M+CU shorter than vein 1M. Metasoma: first tergum longitudinally costate often granulate medially, length greater than apical width; second tergum longitudinally costate, lateral costae distinctly angled toward mid-line; anterior transverse groove present, straight; posterior transverse groove present; third tergum entirely smooth except for costate transverse groove; terga 4–7 smooth; ovipositor equal to length of metasomal tergum 1.

#### Holotype female.

Top label (white, printed) - COSTA RICA: Guanac [;] Arenales, W. side [;] Volcan Cacao, 900m [;] 1988–1989; second label (red, partially printed and hand written) - HOLOTYPE [;] Heterospilus [;] vulcanus [;] P. Marsh. Deposited in ESUW.

#### Paratypes.

2 ♀♀, same data as holotype (ESUW). 4 ♀♀, Costa Rica: Guanacaste [;] Est. Cacao, 1000–1150m [;] ix.1996, I. Villegas, Malaise [;] L.N. 323150-375500 #47559 (ESUW). 1 ♀, Costa Rica: Puntarenas [;] Est. Cacao, 1000–1400m [;] 2km. SW del Cerro Cacao [;] vii.1996, J. A. Ugalde [;] L.N. 323100-375800 #8220 [;] Malaise trap (ESUW). 2 ♀♀, COSTA RICA: [;] Guanacaste [;] Estac. Mengo [;] SW Volcan Cacao [;] 1100m, 1988–1989 (ESUW). 1 ♀, Costa Rica: Heredia [;] Braulio Carrillo N.P. [;] 250–500m IV.10.85 [;] Henri Goulet (AEIC). 1 ♀, COSTA RICA: Puntarenas [;] San Vito, Estac. Biol. [;] Los Altures 1500m [;] iv.1992 P. Hanson (TAMU).

#### Comments.

The second metasomal tergum with lateral costae angled toward mid-line, the short ovipositor and bicolored flagellum are distinctive for this species.

#### Etymology.

Named for Vulcan, the Roman god of fire, in reference to most of the type series being collected at Volcan Cacao.

**Figure 200.**
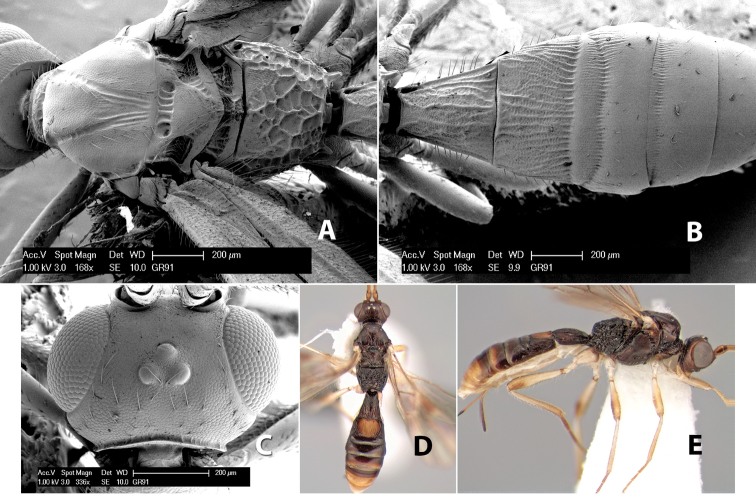
*Heterospilus vulcanus* Marsh, sp. n.: **A–C** paratype **D–E** holotype.

### Heterospilus warreni

Marsh sp. n.

http://zoobank.org/EB2822D4-6844-4ED8-9C80-86ADF518FDBB

http://species-id.net/wiki/Heterospilus_warreni

Figure 201

#### Female.

Body size: 3.0–3.5 mm. Color: body entirely honey yellow, metasomal tergum 2 laterally and lower portion of mesopleuron often darker; scape yellow without lateral brown stripe; flagellum yellow basally to brown apically; wing veins brown, stigma bicolored brown, yellow at apex and along anterior margin; legs yellow. Head: vertex granulate; frons granulate; face rugose; temple in dorsal view broad, slightly bulging behind eye, width slightly less than 1/2 eye width; malar space equal to 1/4 eye height; ocell-ocular distance about 1.5 times diameter of lateral ocellus; 17–22 flagellomeres. Mesosoma: mesoscutal lobes granulate; notauli scrobiculate, meeting at scutellum in triangular rugose area; scutellum granulate; prescutellar furrow with one median cross carinae and occasionally weak carinae on each side; mesopleuron granulate; precoxal sulcus weakly scrobiculate or smooth, shorter than mesopleuron; venter granulate; propodeum with basal median areas distinctly margined, granulate, basal median carina absent, areola not distinctly margined, areolar area rugose, lateral areas entirely rugose. Wings: fore wing vein r longer than vein 3RSa, vein 1cu-a beyond vein 1M; hind wing vein SC+R absent, vein M+CU nearly equal in length to vein 1M. Metasoma: first tergum longitudinally costate, length equal to apical width; second tergum longitudinally costate; anterior transverse groove present, straight; posterior transverse groove weak or absent; third tergum costate basally, smooth apically; terga 4–7 smooth; ovipositor equal to half length of metasoma.

#### Holotype female.

Top label (white, printed) - Costa Rica, Guanacaste Pr. [;] Guan. Conservation Area [;] Santa Rosa hdq., 200m [;] lighttrap, 7-VII 1997 [;] L.J. van der Ent; second label (red, partially printed and hand written) - HOLOTYPE [;] Heterospilus [;] warreni [;] P. Marsh. Deposited in ESUW.

#### Paratypes.

4 ♀♀, same data as holotype with additional date of 6-VII 1997 (ESUW). 1 ♀, Costa Rica: Guanacaste Pr. [;] Guanacaste National Park [;] near Playa Naranja [;] 11 March 1990, J.S. Noyes (ESUW). 1 ♀, top label - Costa Rica: Guanacaste [;] Santa Rosa Natl. Park [;] 300m, ex. Malaise trap [;] Site #: 10 [;] Dates: 8–29.xi.1986 [;] I.D. Gauld & D. Janzen; second label - [BH] Bosque Humedo [;] mature evergreen dry forest [;] [C] more or less fully [;] shaded as possible (ESUW). 1 ♀, top label - Costa Rica: Guanacaste [;] Santa Rosa Natl. Park [;] 300m, ex. Malaise trap [;] Site #: (blank) [;] Dates: 31-I-21-II-1987 [;] I.D. Gauld & D. Janzen; second label - [SE] Bosque San Emilio [;] 50yr old deciduous forest [;] [C] more or less fully [;] shaded as possible (ESUW).

#### Comments.

The honey yellow body and fore wing vein r longer than vein 3RSa are distinctive for this species.

#### Etymology.

Named for my long time friend and mentor in Christ, Warren Litzman.

**Figure 201.**
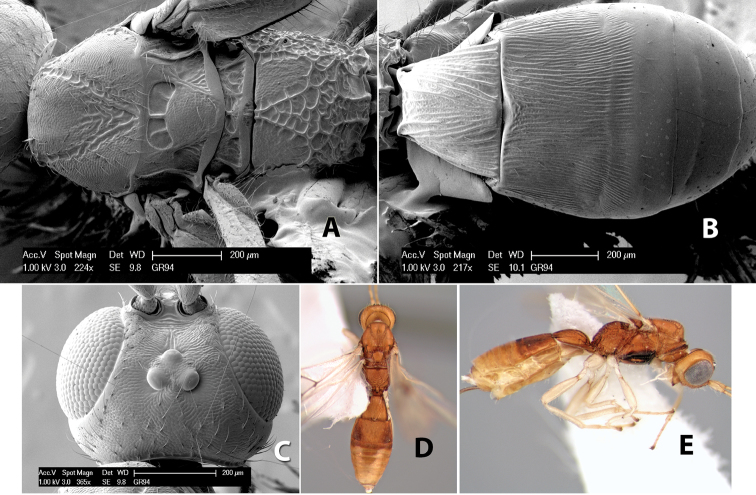
*Heterospilus warreni* Marsh, sp. n.: **A–C** paratype **D–E** holotype.

### Heterospilus wrightae

Marsh sp. n.

http://zoobank.org/FC685F3B-9BFF-46AF-B60E-09DACD8263C8

http://species-id.net/wiki/Heterospilus_wrightae

Figure 202

#### Female.

Body size: 2.5–3.0 mm. Color: body entirely dark brown; scape brown, flagellum entirely brown; wing veins including stigma brown; legs yellow. Head: vertex granulate; frons granulate; face granulate; temple in dorsal view narrow, sloping behind eye, width slightly less than 1/2 eye width; malar space greater than 1/4 eye height; ocell-ocular distance slightly greater than 2.5 times diameter of lateral ocellus; 23–28 flagellomeres. Mesosoma: mesoscutal lobes granulate; notauli scrobiculate, meeting posteriorly in triangular rugose area and often with dimple-like depression at this junction; scutellum granulate; prescutellar furrow with 3–5 cross carinae; mesopleuron granulate; precoxal sulcus smooth, shorter than mesopleuron; venter granulate; propodeum with basal median areas small and not distinctly margined, granulate, basal median carina absent, areola not margined, areolar area areolate, lateral areas entirely rugose. Wings: fore wing vein r shorter than vein 3RSa, vein 1cu-a beyond vein 1M; hind wing vein SC+R present, vein M+CU shorter than vein 1M. Metasoma: first tergum longitudinally costate, occasionally rugose medially; second tergum longitudinally costate; anterior transverse groove present, straight; posterior transverse groove present; third tergum costate basally, granulate apically; terga 4–7 granulate; ovipositor about 3/4 length of metasoma.

#### Holotype female.

First label (white, printed) - Costa Rica: Heredia [;] Est. Biol. La Selva [;] 50–150m, 10.26N, 84.01W [;] ii-iv 1993, P. Hanson [;] huertos Malaise trap [;] set by G. Wright; second label (red, partially printed and hand written) - HOLOTYPE [;] Heterospilus [;] wrightae [;] P. Marsh. Deposited in ESUW.

#### Paratypes.

1 ♀, Costa Rica: Heredia [;] 3km S. Puerto Viejo [;] OTS, La Selva, 100m [;] X.1992, P. Hanson [;] huertos Malaise trap [;] set by G. Wright (ESUW). 1 ♀, Costa Rica: Heredia [;] Est. Biol. La Selva [;] 50–150m, 10.26 N [;] 84.01 W, Aug. 1992 (ESUW). 1 ♀, COSTA RICA-Heredia Prov. [;] La Selva Biological Station [;] 10°26'N, 84°01'W, 100m [;] Canopy fogging 32 [;] 3.xi.1994 [;] Project ALAS (FVK32) (ESUW). 1 ♀, top label - COSTA RICA: Prov. [;] Heredia, F. La Selva [;] 3km S Pto. Viejo [;] 10°26'N, 84°01'W; second label - 28.vii.1982 [;] H. A. Hespenheide; third label - At foliar nectarines [;] of Soc....(?) [;] 2–3 yr. plot (ESUW). 1 ♀, COSTA RICA: Limon [;] 4km NE Bribri [;] 50m, IX-XI 1989 [;] col. Paul Hanson (ESUW). 1 ♀, Costa Rica: Puntarenas [;] Pen. Osa. 5km. N. Pto. [;] Jimenez, 10m, iii-iv. [;] 1991, P. Hanson, Malaise (ESUW). 1 ♀, Costa Rica: Puntarenas [;] San Vito, Estac. Biol. Las Alturas, 1500m [;] iii.1992, Paul Hanson (ESUW). 1 ♀, Costa Rica: Puntarenas [;] Peninsula Osa, Puerto [;] Jimenez, 10m, x-xi.1991 [;] P. Hanson, Malaise trap [;] grassy, disturbed site (ESUW). 2 ♀♀, Costa Rica, Puntarenas [;] R.F. Golfo Dulce, 5km. [;] W. Piedras Blancas, 100m [;] I-1993, P. Hanson (ESUW). 1 ♀, COSTA RICA: Puntarenas [;] San Vito, Estac. Biol [;] Los Altures 1500m [;] iv.1992 P. Hanson (TAMU).

#### Comments.

The brown flagellum, absence of propodeal tubercles above hind coxae, and the dimple-like depression where the notauli meet are distinctive for this species.

#### Etymology.

Named for Geraldine “Jeri” Wright, who, as a student at the University of Wyoming, spent time in Costa Rica collecting parasitic wasps, setting Malaise traps and sorting many specimens of Doryctinae.

**Figure 202.**
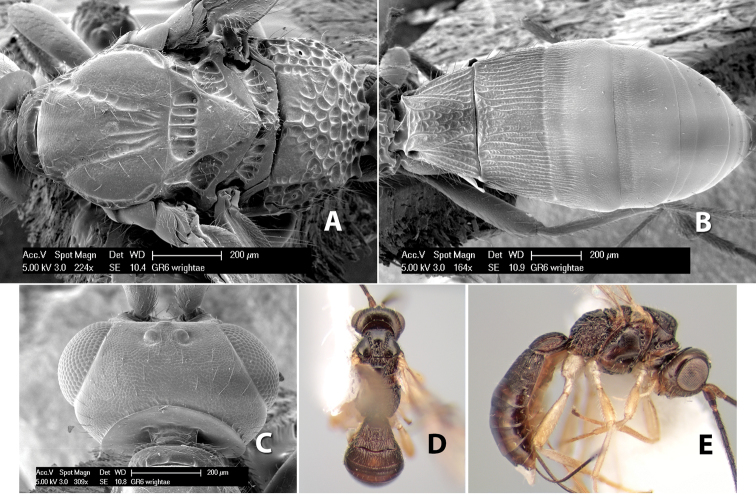
*Heterospilus wrightae* Marsh, sp. n.: **A–C** paratype **D–E** holotype.

### Heterospilus xinca

Marsh sp. n.

http://zoobank.org/33750197-AADB-4B42-A332-6DB79420DF82

http://species-id.net/wiki/Heterospilus_xinca

Figure 203

#### Female.

Body size: 3.0 mm. Color: head brown; scape yellow with lateral longitudinal brown stripe, flagellum brown with apical white annulus, apical 3–5 flagellomeres brown; mesosoma brown, area along notauli and propodeum often lighter brown; metasomal terga light brown to honey yellow, laterally terga are often dark brown; wing veins brown, stigma yellow; legs yellow. Head: vertex granulate; frons granulate; face granulate; temple in dorsal view sloping behind eye, width equal to 1/2 eye width; malar space greater than 1/4 eye height; ocell-ocular distance at least 2.5 times diameter of lateral ocellus; 21–14 flagellomeres. Mesosoma: mesoscutal lobes granulate; notauli scrobiculate, meeting at scutellum in triangular costate area; scutellum weakly granulate; prescutellar furrow with 5 cross carinae; mesopleuron granulate; precoxal sulcus smooth, shorter than mesopleuron; venter granulate; propodeum with basal median areas margined, granulate, basal median carina absent, areola not margined, areolar area rugose, lateral areas entirely rugose. Wings: fore wing vein r shorter than vein 3RSa, vein 1cu-a beyond vein 1M; hind wing vein SC+R absent, vein M+CU shorter than vein 1M. Metasoma: first tergum longitudinally costate, length equal to apical width; second tergum longitudinally costate; anterior transverse groove present, straight; posterior transverse groove present; third tergum costate basally, weakly granulate apically; terga 4–7 weakly granulate; ovipositor as long as metasomal terga 1 and 2 combined.

#### Holotype female.

Top label (white, partially printed and hand written) - Costa Rica: Guanacaste [;] Santa Rosa Natl. Park [;] 300m, ex. Malaise trap [;] Site #: (blank) [;] Dates: 18.i–8.ii.1986 [;] I.D. Gauld & D. Janzen; second label (white, printed) - [BH] Bosque Humedo [;] mature evergreen dry forest [;] [O] in clearing, fully [;] isolated part of day; third label (red, partially printed and hand written) - HOLOTYPE [;] Heterospilus [;] xinca [;] P. Marsh. Deposited in ESUW.

#### Paratypes.

3 ♀♀, same data as holotype except: Site #: BH-9-O; Dates: 14.viii–6.ix.1986, 13.iv–4.v.1986 and 8.ii–2.iii.1986 (ESUW). 7 ♀♀, top label - Costa Rica: Guanacaste [;] Santa Rosa Natl. Park [;] 300m, ex. Malaise trap [;] Site #: BH-12-C and BH-10-C [;] Dates: 8.ii–2.iii.1986, 22.iii–13.iv.1986, 4–24.v.1986, 14.viii–6.ix.1986 and 26.vii–14.viii.1986 [;] I.D. Gauld & D. Janzen; second label - [BH] Bosque Humedo [;] mature evergreen dry forest [;] [C] more or less fully [;] shaded as possible (ESUW). 3 ♀♀, Costa Rica: Guanacaste [;] Santa Rosa Natl. Park [;] 300m, ex. Malaise trap [;] Site #: H-4-C [;] Dates: 4–24.v.1986 and 8–29.xi.1986 [;] I.D. Gauld & D. Janzen; second label - [H] open regenerating [;] woodland <10 years old [;] [C] more or less fully [;] shaded as possible (ESUW).

#### Comments.

The light brown color of the body and the absence of hind wing vein SC+R are distinctive for this species.

#### Etymology.

Named for the Xinca, an indigenous people of Guatemala.

**Figure 203.**
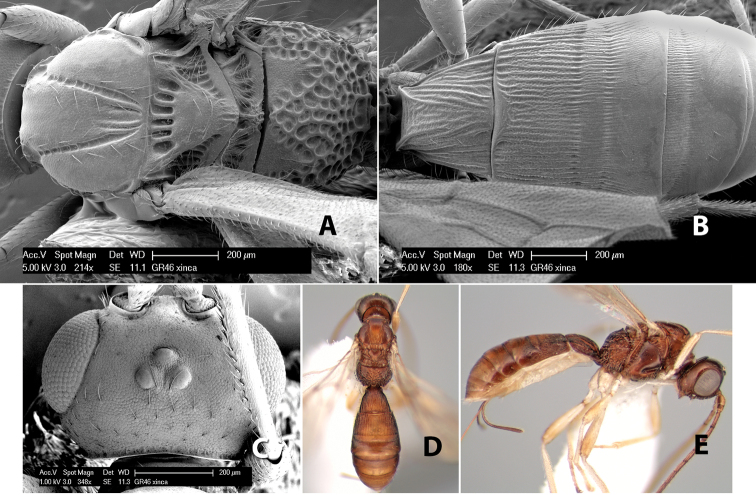
*Heterospilus xinca* Marsh, sp. n.: **A–C** paratype **D–E** holotype.

### Heterospilus zapotec

Marsh sp. n.

http://zoobank.org/D5EA2F85-B626-4437-BD3B-3FF0B98B7947

http://species-id.net/wiki/Heterospilus_zapotec

Figure 204

#### Female.

Body size: 3.5–4.0 mm. Color: body dark brown, metasomal terga 5–7 yellow; scape yellow with lateral longitudinal brown stripe, flagellum brown with apical white annulus, apical 5–7 flagellomeres brown; wing veins including stigma brown; legs yellow. Head: vertex granulate; frons granulate; face granulate; temple in dorsal view narrow and sloping behind eye width 1/2 eye width; malar space equal to 1/4 eye height; ocell-ocular distance about 2.5 times diameter of lateral ocellus; 25–27 flagellomeres. Mesosoma: mesoscutal lobes granulate; notauli scrobiculate, meeting at scutellum in triangle costate area; scutellum weakly granulate or smooth; prescutellar furrow with 3–5 cross carinae; mesopleuron granulate; precoxal sulcus smooth, shorter than mesopleuron; venter weakly granulate or smooth; propodeum with basal median areas weakly but distinctly margined, granular or rugose, basal median carina absent, areola not margined, areolar area areolate-rugose, lateral areas entirely rugose. Wings: fore wing vein r shorter than vein 3RSa, vein 1cu-a beyond vein 1M; hind wing vein SC+R present, vein M+CU shorter than vein 1M. Metasoma: first tergum longitudinal costate, length greater than apical width; second tergum longitudinally costate; anterior transverse groove present, straight; posterior transverse groove present; third tergum costate basally, weakly granulate or smooth apically; terga 4–7 weakly granulate or smooth; ovipositor about 3/4 length of metasoma.

#### Holotype female.

Top label (white, printed) - Costa Rica: Puntarenas, ACO [;] Golfito, R.F. Golfo Dulce [;] Est. Agujas, 250–350m [;] 15.vi–15.vii.1999, J. Azofeifa [;] L.S. 276750-526550 #52838 [;] Malaise trap; second label (red, partially printed and hand written) - HOLOTYPE [;] Heterospilus [;] zapotec [;] P. Marsh. Deposited in ESUW.

#### Paratypes.

1 ♀, COSTA RICA: Limon [;] P.N. Tortuguero [;] Est. 4-esquinas, 0m [;] iv-v.1989, J. Solano (ESUW). 1 ♀, COSTA RICA, Heredia [;] Chilamate, 75m [;] 25/III/1989 [;] col. Hanson & Godoy (ESUW). 1 ♀, Sirena, Osa Pen. [;] VII 77 Cos.Rica [;] D.H. Janzen (AEIC). 1 ♀, S.RosaPark, Guan. [;] C. Rica 4 Nov 77 [;] D,H. Janzen [;] Dry Hill (AEIC). 1 ♀, COSTA RICA, San Jose [;] Zurqui de Moravia [;] 1600m, 24-XII 1988 [;] Col. P. Hanson (MICR).

#### Comments.

This species is similar to *Heterospilus carolinae* but is distinguished by the yellow apical metasomal terga.

#### Etymology.

Named for the Zapotec, an indigenous people of Mexico.

**Figure 204.**
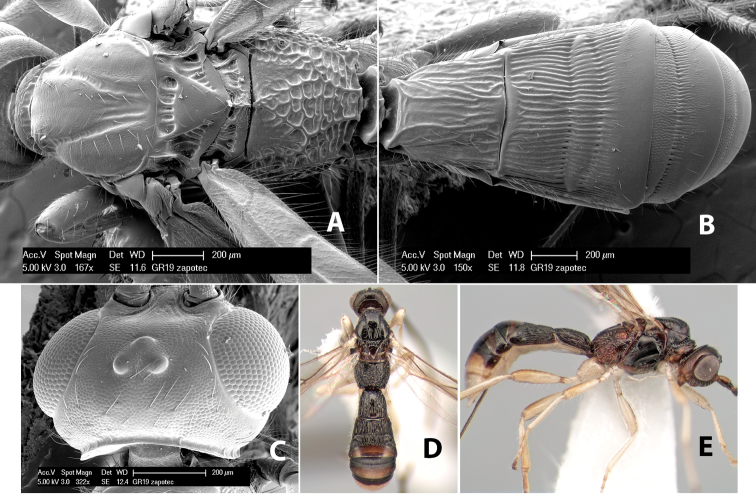
*Heterospilus zapotec* Marsh, sp. n.: **A–D** paratype **E** holotype.

### Heterospilus zitaniae

Marsh sp. n.

http://zoobank.org/B9C88B47-CB7E-4E89-A837-F455D28F3681

http://species-id.net/wiki/Heterospilus_zitaniae

Figure 205

#### Female.

Body size: 3.0 mm. Color: head, mesosoma and metasomal terga 1 and 2 dark brown, metasomal terga 3–7 slightly lighter brown; scape yellow without lateral brown stripe, flagellum brown with apical 3–5 flagellomeres white; wing veins including stigma brown; legs yellow. Head: vertex granulate, nearly smooth near eyes; frons granulate; face weakly granulate; temple in dorsal view narrow, sloping behind eye, width less than 1/2 eye width; malar space greater than 1/4 eye height; ocell-ocular distance at least 2.5 times diameter of lateral ocellus; 24 flagellomeres. Mesosoma: mesoscutal lobes granulate; notauli scrobiculate, meeting at scutellum in triangular costate area; scutellum granulate; prescutellar furrow with 3 cross carinae; mesopleuron granulate; precoxal sulcus granulate, extending to posterior edge of mesopleuron by distinct groove or carinae; venter granulate; propodeum with basal median areas margined, granulate, basal median carina present, areola not distinctly margined, areolar area rugose, lateral areas entirely rugose. Wings: fore wing vein r nearly as long as vein 3RSa, vein 1cu-a beyond vein 1M; hind wing vein SC+R present, vein M+CU shorter than vein 1M. Metasoma: first tergum longitudinally costate, length longer than apical width; second tergum longitudinally costate; anterior transverse groove present, straight; posterior transverse groove present; third tergum smooth except for posterior transverse groove; terga 4–7 smooth; ovipositor equal to length of metasomal terga 1 and 2 combined.

#### Holotype female.

Top label (white, printed) - Costa Rica: Alajuela Prov. [;] Area de Conservation Arenal [;] R. San Ramon, 8–10.vi.1998 [;] N. side San Lorencito near [;] station, ex Malaise trap [;] N. Zitani, S. Dadelahi, [;] R. Fenoff, K. Krenzalok; second label (red, partially printed and hand written) - HOLOTYPE [;] Heterospilus [;] zitaniae [;] P. Marsh. Deposited in ESUW.

#### Paratypes.

Known only from the holotype.

#### Comments.

The long fore wing vein r (nearly as long as vein 3RSa) and the small ocelli are distinctive for this species.

#### Etymology.

Named for Nina Zitani, who helped collect this species and in honor of her interest in the braconid fauna of Costa Rica.

**Figure 205.**
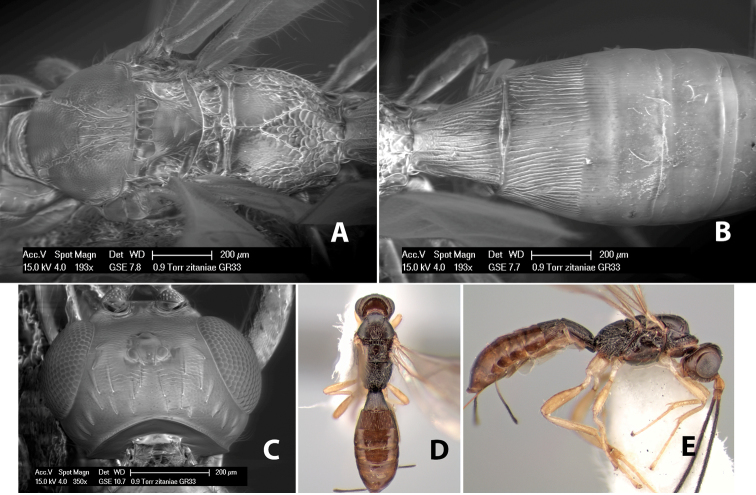
*Heterospilus zitaniae* Marsh, sp. n., holotype.

### Heterospilus zoque

Marsh sp. n.

http://zoobank.org/3B1137DE-AA89-4DE0-8E9F-D053D0462661

http://species-id.net/wiki/Heterospilus_zoque

Figure 206

#### Female.

Body size: 4.0–4.5 mm. Color: body dark brown, metasomal tergum 2 with yellow spots on each side of median brown spot, terga 5–7 yellow; scape yellow with lateral longitudinal brown stripe, flagellum brown with apical white annulus, apical 3–5 flagellomeres brown; wing veins brown, stigma bicolored with yellow at apex and base; legs yellow. Head: vertex weakly granulate, smooth near eyes; frons weakly granulate or smooth; face weakly granulate; temple in dorsal view narrow, width less than 1/2 eye width; malar space greater than 1/4 eye height; ocell-ocular distance about 2.5 times diameter of lateral ocellus; 26–28 flagellomeres. Mesosoma: mesoscutal lobes weakly granulate, sometimes partially smooth on median lobe; notauli scrobiculate, meeting at scutellum in triangular costate area; scutellum weakly granulate or smooth; prescutellar furrow with 3 cross carinae; mesopleuron weakly granulate, smooth just above precoxal sulcus; precoxal sulcus smooth, shorter than mesopleuron; venter granulate; propodeum with basal median areas margined, granulate or granulate-rugose, basal median carina absent, areola not margined, areolar area rugose, lateral areas rugose posteriorly, granulate anteriorly, propodeum with tubercle just above hind coxa at base of petiole. Wings: fore wing vein r shorter than vein 3RSa, vein 1cu-a beyond vein 1M; hind wing vein SC+R present, vein M+CU shorter than vein 1M. Metasoma: first tergum longitudinally costate, length greater than apical width; second tergum longitudinally costate; anterior transverse groove present, straight; posterior transverse groove present; third tergum smooth except for costate posterior transverse groove; terga 4–7 smooth; ovipositor half as long as metasoma.

#### Holotype female.

Top label (white, printed) - Costa Rica: Puntarenas [;] San Vito, Estac. Biol. [;] Las Alturas, 1500m [;] xii.1991, Paul Hanson; second label (red, partially printed and hand written) - HOLOTYPE [;] Heterospilus [;] zoque [;] P. Marsh. Deposited in ESUW.

#### Paratypes.

1♀, COSTA RICA: [;] Puntar. Golfo Dulce [;] 24km W Piedras Blancas [;] 200m, vi-viii 1989 [;] Hanson (ESUW).

#### Comments.

The weakly granulate head and the bicolored stigma and metasomal tergum 2 are distinctive for this species.

#### Etymology.

Named for the Zoque, an indigenous people of Mexico.

**Figure 206.**
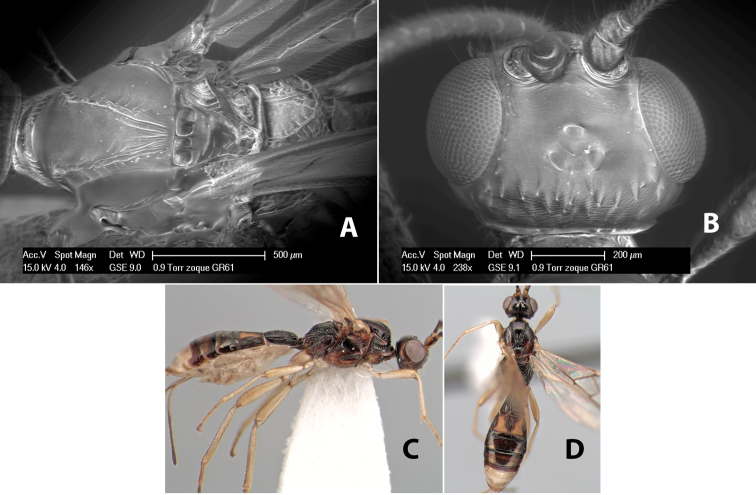
*Heterospilus zoque* Marsh, sp. n., holotype

### Key to species of Costa Rican *Heterospilus* with smooth vertex

**Table:** 

1	Hind wing vein SC+R absent	2
–	Hind wing vein SC+R present	28
2(1)	Mesopleuron smooth, rarely smooth just above pre coxal sulcus only	3
–	Mesopleuron granulate	16
3(2)	Basal median carina of propodeum absent, areola meeting anterior margin of propodeum	4
–	Basal median carina of propodeum present, usually as long or longer than prescutellar furrow cross carinae, rarely shorter and nearly absent	7
4(3)	Ovipositor as long as metasoma	*Heterospilus hespenheidei* Marsh, sp. n.
–	Ovipositor half or less as long as metasoma	5
5(4)	Mesoscutum and scutellum smooth; antenna with 18 or more flagellomeres, apical 3–5 flagellomeres white	*Heterospilus ricacosta* Marsh, sp. n.
–	Mesoscutum and scutellum weakly granulate; antenna with 15 or less flagellomeres, flagellum entirely brown	6
6(5)	Prescutellar furrow with 1 distinct cross carina; mesosoma not flattened dorso-ventrally	*Heterospilus sanjosensis* Marsh, sp. n.
–	Prescutellar furrow with 3 cross carinae; mesosoma somewhat flattened dorso-ventrally	*Heterospilus brevicornus* Marsh, sp. n.
7(3)	Body entirely yellow	*Heterospilus luteus* Marsh, sp. n.
–	Body brown or dark brown, some metasomal terga often lighter	8
8(7)	Mesoscutal lobes smooth	9
–	Mesoscutal lobes granulate	13
9(8)	Notauli incomplete or absent	*Heterospilus careonotaulus* Marsh, sp. n.
–	Notauli complete and distinct	10
10(9)	Antenna with 15 or less flagellomeres, basal flagellomere yellow	*Heterospilus poqomchi* Marsh, sp. n.
–	Antenna with 18 or more flagellomeres, basal flagellomere brown	11
11(10)	Length of first metasomal tergum at most 1.5 times apical width, often nearly equal, all terga brown or dark brown	*Heterospilus wildi* Marsh, sp. n.
–	Length of first metasomal tergum 2 or more times apical width, apical terga usually lighter brown or yellow	12
12(11)	Notauli meeting posteriorly in small triangular rugose area; stigma bright yellow	*Heterospilus penosa* Marsh, sp. n.
–	Notauli meeting posteriorly in 2 converging carinae; stigma usually light brown	*Heterospilus petiolatus* Marsh, sp. n.
13(8)	Second metasomal tergum yellow, lighter than first tergum	14
–	Second metasomal tergum dark brow, concolorous with first tergum	15
14(13)	Precoxal sulcus strongly scrobiculate, as long as mesopleuron	*Heterospilus qanjobal* Marsh, sp. n.
–	Precoxal sulcus weakly scrobiculate, shorter than mesopleuron	*Heterospilus flavostigmus* Marsh, sp. n.
15(13)	Notauli meeting posteriorly in triangular rugose area; length of first metasomal tergum equal to or slightly greater than apical width	*Heterospilus angelicae* Marsh, sp. n.
–	Notauli meeting posteriorly in nearly rectangular distinctly costate area; length of first metasomal tergum at least 1.5 times greater than apical width	*Heterospilus phaeoskelus* Marsh, sp. n.
16(2)	Prescutellar sulcus with 3–5 equally distinct cross carinae	17
–	Prescutellar furrow with 1 distinct median cross carina, rarely with very weak indistinct carinae on each side	22
17(16)	Ovipositor as long as or longer than metasoma	18
–	Ovipositor shorter than metasoma	19
18(17)	Antenna with 13 flagellomeres; basal median carina of propodeum absent, areola meeting anterior edge of propodeum; ovipositor longer than body	*Heterospilus hypermekus* Marsh, sp. n.
–	Antenna with 18–22 flagellomeres; basal median carina of propodeum present; ovipositor slightly longer than metasoma	*Heterospilus smithi* Marsh, sp. n.
19(17)	Apico-lateral corners of propodeum each produced into distinct tubercle	20
–	Apico-lateral corners of propodeum not produced into tubercle	21
20(19)	Metasomal tergum 2 smooth at apex, weakly costate at base; apical 3–5 flagellomeres white	*Heterospilus tuberculatus* Marsh, sp. n.
–	Metasomal tergum 2 entirely costate; flagellomeres entirely brown	*Heterospilus longius* Marsh, sp. n. (in part)
21(19)	Length of metasomal tergum 1 equal to apical width; flagellomeres brown	*Heterospilus thereospilus* Marsh, sp. n.
–	Length of metasomal tergum 1 nearly twice apical width; apical flagellomeres white	*Heterospilus zurquiensis* Marsh, sp. n.
22(16)	Apico-lateral corners of propodeum produced into more or less distinct tubercle	*Heterospilus alejandroi* Marsh, sp. n.
–	Apico-lateral corners of propodeum not produced into tubercle	23
23(22)	Precoxal sulcus nearly as long as mesopleuron, at least extending to posterior margin of mesopleuron by short carinae	24
–	Precoxal sulcus distinctly shorter than mesopleuron	25
24(23)	Apical 3–5 flagellomeres short, length 2.0–2.5 width	*Heterospilus shonan* Marsh, sp. n.
–	Apical flagellomeres longer, length 4–5 times width	*Heterospilus parvus* Marsh, sp. n.
25(23)	Scape with lateral longitudinal light brown stripe	*Heterospilus retheospilus* Marsh, sp. n.
–	Scape entirely yellow, without lateral brown stripe	26
26(25)	Fore wing vein r shorter than vein 3RSa; length of metasomal tergum 1 slightly greater than apical width	*Heterospilus wahli* Marsh, sp. n.
–	Fore wing vein r equal to or longer than vein 3RSa; length of metasomal tergum 1 equal to apical width	27
27(26)	Head yellow, mesosoma dark brown	*Heterospilus conservatus* Marsh, sp. n.
–	Head and mesosoma concolorous brown	*Heterospilus attraholucus* Marsh, sp. n.
28(1)	Mesopleuron granulate, rarely weakly granulate dorsally and more or less smooth above precoxal sulcus	29
–	Mesopleuron smooth and polished	49
29(28)	Ovipositor longer than metasoma	30
–	Ovipositor as long as or shorter than metasoma	34
30(209)	Prescutellar furrow with 3–5 cross carinae	*Heterospilus dos* Marsh, sp. n.
–	Prescutellar furrow with 1 cross carina	31
31(30)	Propodeum with more or less distinct tubercle (formed by raised carina) apically on each side of metasomal base just above hind coxa; flagellum with apical 5–7 flagellomeres white	32
–	Propodeum without such distinct tubercle; flagellum entirely brown	33
32(31)	Median basal carina of propodeum absent, areola meeting anterior margin of propodeum; mesoscutal lobes yellow	*Heterospilus cuatro* Marsh, sp. n.
–	Median basal carina of propodeum present, about as long as cross carina of prescutellar furrow; mesoscutal lobes dark brown	*Heterospilus malaisei* Marsh, sp. n.
33(31)	Metasomal terga 2 and 3 smooth; body mostly light brown	*Heterospilus colonensis* Marsh, sp. n.
–	Metasomal terga 2 and 3 costate; at least mesosoma dark brown	*Heterospilus uno* Marsh, sp. n.
34(29)	Prescutellar furrow with 1 cross carina	35
–	Prescutellar furrow with 3–5 cross carinae	38
35(34)	Metasomal tergum 2 narrow, at least 4 times as wide as long, metasomal terga 3 (at apex), 4 and 5 weakly granulate	*Heterospilus tres* Marsh, sp. n.
–	Metasomal tergum 2 broad, at most 3 times as wide as long, metasomal terga 3 (at apex), 4 and 5 smooth	36
36(35)	Length of first metasomal tergum twice or more apical width; metasomal terga 2–7 smooth	*Heterospilus levis* Marsh, sp. n. (in part)
–	Length of first metasomal tergum at most slightly greater than apical width; metasomal terga 2–3 costate	37
37(36)	Ovipositor at most as long as metasomal tergum 1	*Heterospilus cabecares* Marsh, sp. n.
–	Ovipositor about 3/4 length of metasoma	*Heterospilus nueve* Marsh, sp. n.
38(34)	Mesosoma and metasoma yellow, head dark brown	*Heterospilus nigracapitus* Marsh, sp. n.
–	Body entirely brown or dark brown, rarely mesosoma bicolored brown and light brown with head light brown or yellow	39
39(38)	Scape yellow with lateral longitudinal brown stripe	40
–	Scape entirely yellow or brown	41
40(39)	Ovipositor as long as metasomal terga 1 and 2 combined; stigma entirely light brown	*Heterospilus diez* Marsh, sp. n.
–	Ovipositor as long as metasoma; stigma brown with yellow spots at base and apex	*Heterospilus cinco* Marsh, sp. n.
41(39)	Ovipositor as long as metasoma	42
–	Ovipositor at most half as long as metasoma, usually shorter than metasomal terga 1 and 2 combined	43
42(41)	Anterior transverse groove of metasomal tergum 2 sinuate	*Heterospilus chorti* Marsh, sp. n.
–	Anterior transverse groove of metasomal tergum 2 straight	*Heterospilus cero* Marsh, sp. n.
43(41)	Metasomal terga 4 and 5 longitudinally costate on basal 3/4, smooth on apical 1/4	*Heterospilus hachaensis* Marsh, sp. n.
–	Metasomal terga 4 and 5 entirely smooth	44
44(43)	Length of metasomal tergum 1 nearly twice apical width	*Heterospilus racostica* Marsh, sp. n.
–	Length of metasomal tergum 1 equal to apical width, at most very slightly greater	45
45(44)	Temple in dorsal view bulging behind eye, width equal to 1/2 eye width	*Heterospilus siete* Marsh, sp. n.
–	Temple in dorsal view sloping inward behind eye, width less than 1/2 eye width	46
46(44)	Face smooth	*Heterospilus nunesi* Marsh, sp. n.
–	Face weakly striate, rarely partially smooth near eye	47
47(46)	Median basal carina of propodeum absent or very short, distinctly shorter than cross carina of prescutellar furrow; flagellum with apical white annulus	*Heterospilus masneri* Marsh, sp. n.
–	Median basal carina of propodeum present, slightly longer than cross carina of prescutellar furrow; flagellum brown	48
48(47)	Apical-lateral corners of propodeum produced into small tubercles	*Heterospilus fischeri* Marsh, sp. n. (in part)
–	Propodeum without apical-lateral corners produced into small tubercles	*Heterospilus seis* Marsh, sp. n.
49(28)	Ovipositor longer than metasoma	50
–	Ovipositor equal to or shorter than metasoma	63
50(49)	Face rugose or striate, at least partially	51
–	Face smooth	54
51(50)	Body entirely yellow	*Heterospilus washingtoni* Marsh, sp. n.
–	At least head and mesosoma dark brown	52
52(51)	Basal median carina of propodeum present and longer than prescutellar cross carinae, basal median areas not margined	*Heterospilus nephilim* Marsh, sp. n.
–	Basal median carina of propodeum absent, areola reaching anterior margin of propodeum, basal median areas distinctly margined	53
53(52)	Legs yellow, trochanters often lighter yellow	*Heterospilus veintiuno* Marsh, sp. n.
–	Hind femur bicolored, yellow on basal 1/2 and brown on apical 1/2	*Heterospilus zeus* Marsh, sp. n.
54(50)	Mesoscutum with distinct transverse carinae on lateral lobes along notauli and laterally on median lobe	*Heterospilus pharkidodus* Marsh, sp. n.
–	Mesoscutal lobes smooth or granulate	55
55(54)	Mesoscutal lobes smooth	56
–	Mesoscutal lobes granulate, rarely weakly so	59
56(55)	Temple in dorsal view broad, width greater than 1/2 eye width; antenna with less than 15 flagellomeres, all flagellomeres brown	57
–	Temple in dorsal view narrow, sloping behind eye, width less than 1/2 eye width; antenna with more than 15 flagellomeres, apical flagellomeres white	58
57(56)	Notauli not complete, absent posteriorly	*Heterospilus leiponotaulus* Marsh, sp. n.
–	Notauli complete	*Heterospilus diecisiete* Marsh, sp. n.
58(56)	Propodeum laterally rugose posteriorly, smooth anteriorly; propodeum with tubercle (raised carina) apically on each side of metasomal attachment just above hind coxa	*Heterospilus trece* Marsh, sp. n.
–	Propodeum entirely rugose laterally; propodeum without distinct tubercle apically at base of metasoma	*Heterospilus macrocaudatus* Marsh, sp. n.
59(55)	Anterior transverse groove of metasomal tergum 2 sinuate	*Heterospilus luteoscutum* Marsh, sp. n.
–	Anterior transverse groove of metasomal tergum 2 straight	60
60(59)	Length of metasomal tergum 1 nearly twice apical width	*Heterospilus catiensis* Marsh, sp. n.
–	Length of metasomal tergum 1 equal to or slightly greater than apical width	61
61(60)	Legs brown, trochanters and extreme base of femur lighter	*Heterospilus veinte* Marsh, sp. n.
–	Legs entirely yellow	62
62(61)	Head brown, mesosoma and metasoma dark brown	*Heterospilus terrabas* Marsh, sp. n.
–	Head, mesosoma and metasoma dark brown	*Heterospilus huetares* Marsh, sp. n.
63(49)	Face sculptured, striate or rugose, rarely weakly so	64
–	Face smooth	69
64(63)	Metasomal terga 3–5 nearly completely longitudinally costate	*Heterospilus holleyae* Marsh, sp. n.
–	Metasomal terga 4–5 smooth	65
65(64)	Prescutellar furrow with 1 cross carina	*Heterospilus aphrodite* Marsh, sp. n.
–	Prescutellar furrow with 3–5 cross carinae	66
66(65)	Mesoscutal lobes smooth and shining	67
–	Mesoscutal lobes granulate	68
67(66)	Fore wing vein r nearly as long as vein 3RSa; flagellum brown	*Heterospilus gauldi* Marsh, sp. n. (in part)
–	Fore wing vein r half or less length of vein 3RSa; flagellum brown with apical white annulus	*Heterospilus reagani* Marsh, sp. n.
68(66)	Metasomal terga dark brown or black	*Heterospilus trienta* Marsh, sp. n.
–	Metasomal terga beyond 1 yellow or light brown, tergum 2 often darker medially and laterally	*Heterospilus saturn* Marsh, sp. n.
69(63)	Prescutellar furrow with 1 distinct median cross carina, rarely with weak, short carinae on each side of median distinct carina	70
–	Prescutellar furrow with 3–5 distinct strong cross carinae	87
70(69)	Mesoscutal lobes granulate, often weakly so	71
–	Mesoscutal lobes smooth	79
71(70)	Body entirely yellow	*Heterospilus jupiter* Marsh, sp. n.
–	Body brown, dark brown or bicolored brown and yellow	72
72(71)	Metasomal tergum 3 distinctly costate basally, usually somewhat beyond posterior transverse groove	73
–	Metasomal tergum 3 smooth, at least smooth beyond posterior transverse groove	75
73(72)	Metasoma beyond tergum 1 yellow-orange, mesosoma and head dark brown; stigma yellow	*Heterospilus luteogaster* Marsh, sp. n.
–	Metasoma dark brown, concolorous with mesosoma and head; stigma brown	74
74(73)	Temple in dorsal view narrow, sloping behind eye, width less than 1/2 eye width	*Heterospilus demeter* Marsh, sp. n.
–	Temple in dorsal view broader, bulging behind eye, width about equal to 1/2 eye width	*Heterospilus colliletus* Marsh, sp. n.
75(72)	Length of metasomal tergum 1 at least twice as long as apical width, terga 2 and 3 entirely smooth	*Heterospilus dieciseis* Marsh, sp. n.
–	Length of metasomal tergum 1 at most slightly greater than apical width, tergum 2 distinctly costate	76
76(75)	Antenna with 15 or less flagellomeres	77
–	Antenna with more than 15 flagellomeres	78
77(76)	Mesoscutal lobes densely hairy over most of surface; precoxal sulcus without carinae extending to posterior margin of mesopleuron	*Heterospilus athena* Marsh, sp. n.
–	Mesoscutal lobes sparsely hairy along notauli; precoxal sulcus with carinae extending to posterior margin of mesopleuron	*Heterospilus breviarius* Marsh, sp. n.
78(76)	Temple in dorsal view broad, width greater than 1/2 eye width	*Heterospilus empalmensis* Marsh, sp. n.
–	Temple in dorsal view narrow, sloping behind eye, width less than 1/2 eye width	*Heterospilus dieciocho* Marsh, sp. n.
79(70)	Metasomal terga 3–5 with white unsclerotized area across posterior borders; tergum 2 narrow, width about 4 times median length	80
–	Metasomal terga 3–5 entirely sclerotized; tergum 2 broader width usually less than 3 timed median length	81
80(79)	Apical 3–5 flagellomeres white	*Heterospilus hansonorum* Marsh, sp. n.
–	Flagellomeres entirely brown	*Heterospilus once* Marsh, sp. n.
81(79)	Length of metasomal tergum 1 at least twice apical width; metasomal tergum 2 weakly costate at base only	82
–	Length of metasomal tergum 1 equal to or slightly greater than apical width; metasomal tergum 2 entirely coarsely costate	83
82(81)	Fore wing vein r nearly as long as vein 3RSa; flagellum entirely brown	*Heterospilus artemis* Marsh, sp. n.
–	Fore wing vein 1/2 or less as long as vein 3RSa; apical 3–5 flagellomeres white	*Heterospilus levitergum* Marsh, sp. n.
83(81)	Metasomal terga 3 and 4 granulate apically	*Heterospilus veintitres* Marsh, sp. n.
–	Metasomal terga 3 and 4 smooth apically	84
84(83)	Stigma dark brown	*Heterospilus dulcus* Marsh, sp. n.
–	Stigma yellow	85
85(84)	Posterior transverse groove of metasomal tergum 3 slightly sinuate	*Heterospilus diecinueve* Marsh, sp. n.
–	Posterior transverse groove of metasomal tergum 3 straight	86
86(85)	Apical 5–7 flagellomeres white	*Heterospilus leviscutum* Marsh, sp. n.
–	Flagellum entirely brown	*Heterospilus mercury* Marsh, sp. n.
87(69)	Mesoscutal lobes granulate, sometimes weakly so and partially smooth	88
–	Mesoscutal lobes smooth and shining	89
88(87)	Basal median areas of propodeum smooth; stigma bicolored, brown with light yellow apex	*Heterospilus ypsilon* Marsh, sp. n.
–	Basal median areas of propodeum granulate, sometimes weakly so; stigma entirely brown	*Heterospilus xerxes* Marsh, sp. n.
89(87)	Scape yellow with lateral longitudinal brown stripe	90
–	Scape entirely yellow or brown, without lateral longitudinal brown stripe	91
90(89)	Anterior transverse groove of metasomal tergum 2 slightly sinuate	*Heterospilus veintidos* Marsh, sp. n.
–	Anterior transverse groove of metasomal tergum 2 straight	*Heterospilus catorce* Marsh, sp. n.
91(89)	Basal median carina of propodeum absent, areola meeting apical border of propodeum	*Heterospilus doce* Marsh, sp. n.
–	Basal median carina present, at least as long as prescutellar cross carinae	92
92(91)	Length of metasomal tergum 1 twice apical width; apical 3–7 flagellomeres white	93
–	Length of metasomal tergum 1 equal to or only slighter greater than apical width; flagellum entirely brown	94
93(92)	Mesoscutum honey yellow, remainder of mesosoma dark brown	*Heterospilus apollo* Marsh, sp. n.
–	Mesosoma entirely dark brown	*Heterospilus hera* Marsh, sp. n.
94(92)	Stigma yellow	*Heterospilus mars* Marsh, sp. n.
–	Stigma brown	*Heterospilus borucas* Marsh, sp. n.

### Heterospilus alejandroi

Marsh sp. n.

http://zoobank.org/C3F3DDA0-36BB-424A-906B-4B89FD6E7FA2

http://species-id.net/wiki/Heterospilus_alejandroi

Figure 207

#### Female.

Body size: 2.5 mm. Color: body dark brown; scape yellow without lateral brown stripe, flagellum brown; wing veins including stigma brown; legs yellow. Head: vertex smooth; frons smooth; face smooth; temple in dorsal view broad but sloping behind eye, width slightly greater than 1/2 eye width; malar space greater than 1/4 eye height; ocell-ocular distance greater than 2.5 times diameter of lateral ocellus; 14–16 flagellomeres. Mesosoma: mesoscutal lobes weakly granulate; notauli smooth posteriorly, meeting at scutellum in small triangular costate area, rarely this area unsculptured; scutellum weakly granulate; prescutellar furrow with 1 cross carina; mesopleuron weakly granulate or smooth; precoxal sulcus scrobiculate, shorter than mesopleuron; venter weakly granulate or smooth; propodeum with basal median areas usually margined but rarely not distinctly margined, granulate, basal median carina absent, areola not margined, areolar area areolate-rugose, lateral areas entirely rugose, apical-lateral corners rarely with small tubercle. Wings: fore wing vein r equal to or longer than vein 3RSa, vein 1cu-a beyond vein 1M; hind wing vein SC+R absent, vein M+CU shorter than vein 1M. Metasoma: first tergum longitudinally costate, length greater than apical width; second tergum longitudinally granulate-costate; anterior transverse groove weakly present, straight; posterior transverse groove weakly present; third tergum smooth; terga 4–7 smooth; ovipositor half as long as metasomal tergum 1.

#### Holotype female.

Top label (white, printed) - Costa Rica, Puntarenas [;] San Vito, Estac. Biol. [;] Las Alturas, 200m [;] I-II-1995, P. Hanson; second label (red, partially printed and hand written) - HOLOTYPE [;] Heterospilus [;] alejandroi [;] P. Marsh. Deposited in ESUW.

#### Paratypes.

1 ♀, same data as holotype (ESUW). 4 ♀♀, Costa Rica: San Jose [;] Zurqui de Moravia [;] 1600m, iii.1992 [;] Col. Paul Hanson (ESUW). 3 ♀♀, Costa Rica: San Jose [;] Zurqui De Moravia [;] 1600m. VII-1996 [;] P. Hanson (ESUW). 1 ♀, Costa Rica: San Jose [;] Zurqui de Moravia [;] 1600m, February 1996 [;] P. Hanson, Malaise (ESUW). 1 ♀, Costa Rica: San Jose [;] Zurqui de Moravia [;] 1600m, P. Hanson [;] ix.1995 (ESUW). 2 ♀♀, Costa Rica: Cartago [;] 4km NE Cañon [;] Genesis II, 2350m [;] viii.1995, P. Hanson (ESUW). 1 ♀, Costa Rica: San Jose [;] Cerro de la Muerte [;] 6km. N. San Gerardo [;] 2800m, August 1992 [;] P. Hanson, Malaise (ESUW). 1 ♀, Costa Rica: San Jose [;] 26km. N. San Isidro [;] just S. of Division [;] 2100m, ix-x.1992 [;] P. Hanson, Malaise [;] secondary growth (ESUW). 1 ♀, Costa Rica: Alajuela [;] ACA R.B. San Ramon [;] 900m, Malaise near [;] road, 14–21.vii.1998 [;] L. J. van der Ent (ESUW). 1 ♀, Costa Rica: Puntarenas [;] San Vito, Las Cruces [;] Wilson Botanical Gardens [;] 18–22.iii.1990, 1150m [;] J.S. Noyes (ESUW). 1 ♀, COSTA RICA, Puntar [;] San Vito, Los Fablos [;] 1600m 10-III-89 (MICR).

#### Comments.

The dark brown body, weakly granulate mesoscutum and smooth metasomal terga 3–7 are distinctive for this species.

#### Etymology.

Named for my friend and fellow student of the Doryctinae, Alejandro Zaldivar-Riverón.

**Figure 207.**
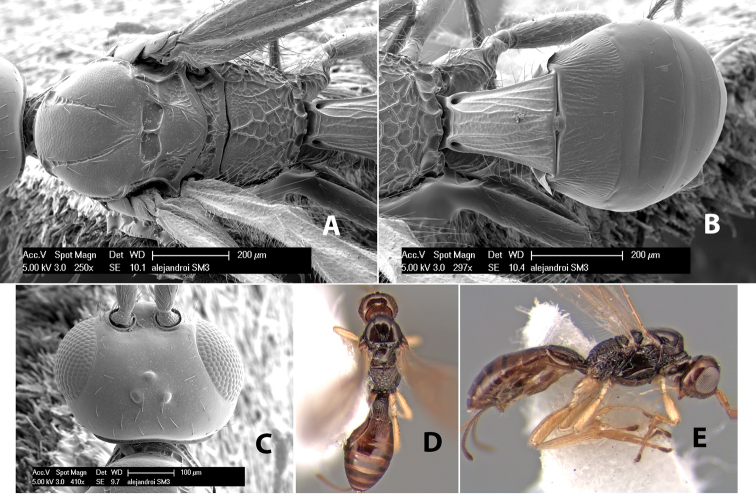
*Heterospilus alejandroi* Marsh, sp. n.: **A–C** paratype **D–E** holotype.

### Heterospilus angelicae

Marsh sp. n.

http://zoobank.org/875FFEC6-50E5-45A1-84EF-279231D85E79

http://species-id.net/wiki/Heterospilus_angelicae

Figure 208

#### Female.

Body size: 3.0–3.5 mm. Color: body dark brown, apical metasomal terga lighter brown to yellow; scape light brown without lateral brown stripe, flagellum brown; wing veins including stigma brown; legs yellow. Head: vertex smooth; frons smooth; face smooth; temple in dorsal view broad, slightly bulging behind eye, width equal to 1/2 eye width; malar space greater than 1/4 eye height; ocell-ocular distance about 2.5 times diameter of lateral ocellus; 23–27 flagellomeres. Mesosoma: mesoscutal lobes granulate; notauli scrobiculate, meeting posteriorly in triangular rugose-costate area; scutellum weakly granulate; prescutellar furrow with 3 cross carinae, often with median carina strongest; mesopleuron smooth; precoxal sulcus scrobiculate, nearly as long as mesopleuron, at least with short carinae extending to posterior margin of mesopleuron; venter smooth; propodeum with basal median areas margined, granulate, basal median carina present, areola not margined, areolar area rugose, lateral areas entirely rugose. Wings: fore wing vein r about as long as vein 3RSa, vein 1cu-a beyond vein 1M; hind wing vein SC+R absent, vein M+CU longer than vein 1M. Metasoma: first tergum longitudinally costate, rugose medially; second tergum longitudinally costate; anterior transverse groove present, straight; posterior transverse groove present; third tergum entirely smooth; terga 4–7 smooth; ovipositor as long as metasoma.

#### Holotype female.

Top label (white, printed) - Costa Rica: San Jose [;] Zurqui de Moravia [;] 1600m, v 1992 [;] Col. Paul Hanson; second label (red, partially printed and hand written) - HOLOTYPE [;] Heterospilus [;] angelicae [;] P. Marsh. Deposited in ESUW.

#### Paratypes.

3 ♀♀, same data as holotype with additional date of III-1995 (ESUW). 1 ♀, COSTA RICA: [;] Guanacaste [;] Estac. Mengo [;] SW Volcan Cacao [;] 1100m, 1988–1989 (ESUW). 1 ♀, Costa Rica: Limon, ACLAC [;] Central, R.B. Hitoy Cerere [;] Estac. Hitoy Cerere, #52757 [;] Send, Tomade Agua, 100m [;] 17.iv–8.v.1999, F. Umana [;] L.N. 184600-643400 [;] Malaise trap (ESUW). 1 ♀, Costa Rica: San Jose [;] Cerro de la Muerte [;] 2km W Empalme [;] 2300m, February 1995 [;] P. Hanson, Malaise (ESUW). 1 ♀, COSTA RICA: San Jose [;] Zurqui de Moravia [;] ix-x.1993 1600m [;] P. Hanson (TAMU).

#### Comments.

The smooth mesopleuron, longer ovipositor, broad temple and brown flagellum are distinctive for this species.

#### Etymology.

Named for my friend and fellow braconidologist, Angelica Penteado-Dias.

**Figure 208.**
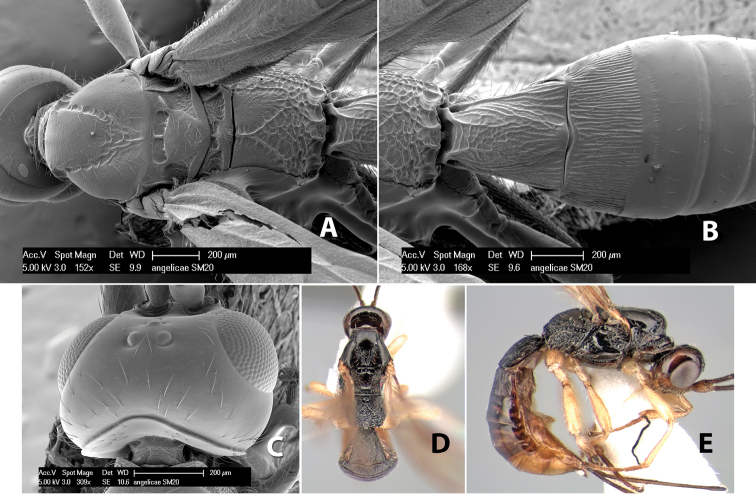
*Heterospilus angelicae* Marsh, sp. n.: **A–C** paratype **D–E** holotype.

### Heterospilus aphrodite

Marsh sp. n.

http://zoobank.org/4054EC85-837C-41D7-88F3-5BC892A1145E

http://species-id.net/wiki/Heterospilus_aphrodite

Figure 209

#### Female.

Body size: 3.0–3.5 mm. Color: head yellow; scape yellow without lateral brown stripe; flagellum brown with basal flagellomeres lighter; mesosoma bicolored, mesoscutum, scutellum and mesopleuron dark brown, propleuron, pronotum, propodeum and venter honey yellow; wing veins including stigma brown; legs yellow; metasomal terga light brown, tergum 3 darker apically. Head: vertex smooth; frons smooth; face weakly rugose and partially smooth; temple in dorsal view narrow but slightly bulging behind eye, width less than 1/2 eye width; malar space equal to 1/4 eye height; ocell-ocular distance about twice diameter of lateral ocellus; 25 flagellomeres. Mesosoma: mesoscutal lobes granulate; notauli scrobiculate, meeting posteriorly in triangular rugose area; scutellum granulate; prescutellar furrow with 1 cross carina; mesopleuron smooth; precoxal sulcus weakly scrobiculate or smooth; venter smooth; propodeum with basal median areas margined, weakly granulate or partially smooth, basal median carina present but short, areola not completely margined, areolar area rugose, lateral areas entirely rugose. Wings: fore wing vein r shorter than vein 3RSa, vein 1cu-a beyond vein 1M; hind wing vein SC+R present, vein M+CU about as long as vein 1M. Metasoma: first tergum longitudinally costate-granulate, length equal to apical width; second tergum longitudinally costate-granulate; anterior transverse groove present, sinuate; posterior transverse groove present; third tergum costate basally, smooth apically; terga 4–7 smooth; ovipositor as long as metasomal terga 1 and 2 combined.

#### Holotype female.

Top label (white, partially printed and hand written) - Costa Rica: Guanacaste [;] Santa Rosa Natl. Park [;] 300m, ex. Malaise trap [;] Site #: (blank) [;] Dates: 10–31.i.1987 [;] I.D. Gauld & D. Janzen; second label (white, printed) - [H] open regenerating [;] woodland <10 years old [;] [C] more or less fully [;] isolated as possible; third label (red, partially printed and hand written) - HOLOTYPE [;] Heterospilus [;] aphrodite [;] P. Marsh. Deposited in ESUW.

#### Paratypes.

1 ♀, Costa Rica: Guanacaste [;] PN Guanacaste, 7km E HQ [;] near “small house” [;] 9.iii.1990, J. S. Noyes (ESUW). 1 ♀, S.RosaPark, Guan. [;] C. Rica 4 Jan 78 [;] D.H. Janzen [;] Dry Hill (AEIC).

#### Comments.

The bicolored body, the brown flagellum, large ocelli and short ovipositor are distinctive for this species.

#### Etymology.

Named for the Greek goddess of love, Aphrodite.

**Figure 209.**
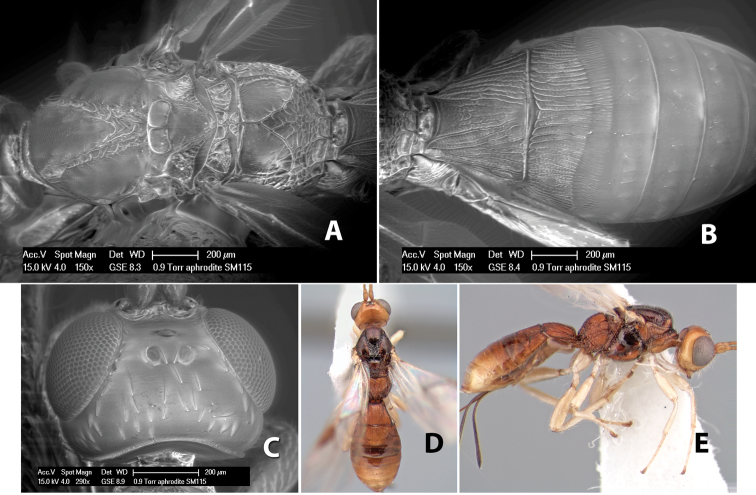
*Heterospilus aphrodite* Marsh, sp. n., holotype.

### Heterospilus apollo

Marsh sp. n.

http://zoobank.org/C4E16627-46FB-40A0-B10B-0F9571A11D53

http://species-id.net/wiki/Heterospilus_apollo

Figure 210

#### Female.

Body size: 3.0 mm. Color: head dark brown, face somewhat lighter; scape light brown without lateral brown stripe; flagellum brown with apical 5–7 flagellomeres white; mesosoma dark brown with propleuron, mesoscutum and venter honey yellow; wing veins including stigma brown; coxae, trochanters, basal 1/5 of femora yellow, remainder of legs brown; metasomal terga 1 and 2 dark brown, remainder of terga honey yellow. Head: vertex smooth; frons smooth; face smooth; temple in dorsal view narrow, sloping behind eye, width less than 1/2 eye width; malar space equal to 1/4 eye height; ocell-ocular distance greater than 2.5 times diameter of lateral ocellus; 20 flagellomeres. Mesosoma: mesoscutal lobes smooth; notauli smooth, meeting posteriorly in unsculptured area except for 2 converging carinae; scutellum smooth; prescutellar furrow with 3–5 cross carinae; mesopleuron smooth; precoxal sulcus smooth, shorter than mesopleuron; venter smooth; propodeum with basal median areas margined, smooth, basal median carina present, short, areola distinctly margined, areolar area rugose, lateral areas entirely rugose. Wings: fore wing vein r shorter than vein 3RSa, vein 1cu-a interstitial with vein 1M; hind wing vein SC+R present, vein M+CU shorter than vein 1M. Metasoma: first tergum longitudinally costate, length nearly 3 times apical width; second tergum longitudinally costate; anterior transverse groove present, straight; posterior transverse groove weak or absent; third tergum entirely smooth; terga 4–7 smooth; ovipositor 3/4 length of metasoma.

#### Holotype female.

Top label (white, printed) - Costa Rica: Puntarenas [;] San Vito, Estac. Biol. [;] Las Alturas, 1500m [;] xi.1991, Paul Hanson; second label (red, partially printed and hand written) - HOLOTYPE [;] Heterospilus [;] apollo [;] P. Marsh. Deposited in ESUW.

#### Paratypes.

Known only from the holotype.

#### Comments.

The long and narrow metasomal tergum 1, the bicolored mesosoma and the white apical annulus of the flagellum are distinctive for this species.

#### Etymology.

Named for the Greek god Apollo.

**Figure 210.**
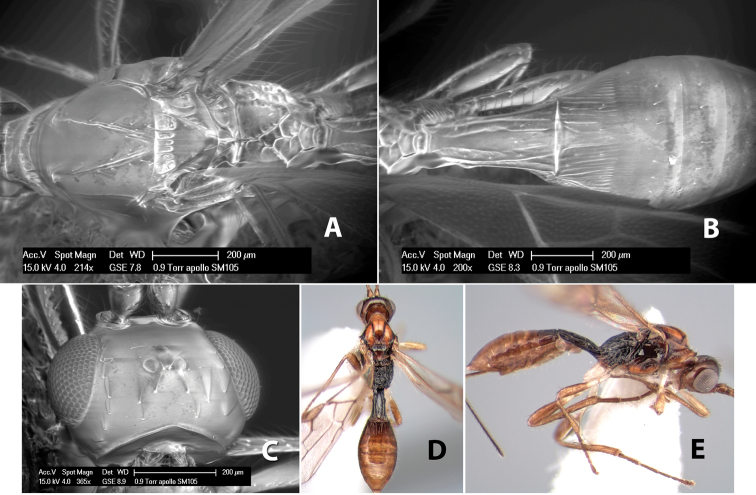
*Heterospilus apollo* Marsh, sp. n., holotype.

### Heterospilus artemis

Marsh sp. n.

http://zoobank.org/7FB01990-2772-408D-91DF-D6504D6320A0

http://species-id.net/wiki/Heterospilus_artemis

Figure 211

#### Female.

Body size: 2.0–2.5 mm. Color: body dark brown, terga 1–3 sometimes lighter brown or yellow; scape yellow without lateral brown stripe; flagellum brown; wing veins brown, stigma yellow; legs yellow or bicolored, fore and mid coxae and trochanters yellow, femora, tibiae and tarsi light brown, hind coxa brown, trochanters yellow, femur (except at extreme base), tibia and tarsus brown. Head: vertex smooth; frons smooth; face smooth; temple in dorsal view broad, bulging behind eye, width nearly equal to eye width; malar space greater than 1/4 eye height; ocell-ocular distance greater than 2.5 times diameter of lateral ocellus; 14 flagellomeres. Mesosoma: mesoscutal lobes smooth or very weakly granulate; notauli scrobiculate, meeting before prescutellar furrow in medial longitudinal groove; scutellum smooth; prescutellar furrow with 1 cross carina; mesopleuron smooth; precoxal sulcus scrobiculate, shorter than mesopleuron; venter smooth; propodeum with basal median areas margined, smooth, basal median carina present, areola margined, areolar area rugose, lateral areas entirely rugose. Wings: fore wing vein r slightly shorter or as long as vein 3RSa and slightly longer than width of stigma, vein 1cu-a beyond vein 1M; hind wing vein SC+R present, vein M+CU shorter than vein 1M. Metasoma: first tergum longitudinally costate, length greater than apical width; second tergum weakly costate basally, smooth apically; anterior transverse groove weak or absent; posterior transverse groove weak or absent; third tergum entirely smooth; terga 4–7 smooth; ovipositor about 3/4 length of metasoma.

#### Holotype female.

Top label (white, printed) - Costa Rica: San Jose [;] Cerro de la Muerte, 19km [;] S 3km W Empalme, 2600m [;] ix.1992, P. Hanson, Malaise; second label (red, partially printed and hand written) - HOLOTYPE [;] Heterospilus [;] artemis [;] P. Marsh. Deposited in ESUW.

#### Paratypes.

1 ♀, Costa Rica: San Jose [;] Cerro de la Muerte [;] 2km W. Empalme [;] 2300m, June 1995 [;] P. Hanson, Malaise (ESUW).

#### Comments.

The median apical grove on the mesoscutum where the notauli meet, the short antenna and the wide temple are distinctive for this species.

#### Etymology.

Named for the Greek goddess of the hunt, Artemis.

**Figure 211.**
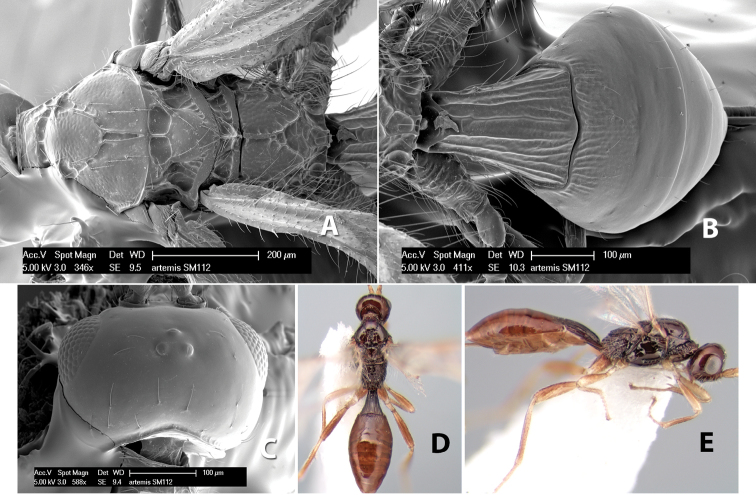
*Heterospilus artemis* Marsh, sp. n.: **A–C** paratype **D–E** holotype.

### Heterospilus athena

Marsh sp. n.

http://zoobank.org/66CD7347-2B44-4257-B4E9-D2BD64A74525

http://species-id.net/wiki/Heterospilus_athena

Figure 212

#### Female.

Body size: 2.0 mm. Color: body brown to dark brown; scape yellow; flagellum brown; wing veins including stigma brown; legs bicolored yellow with all femora brown on apical 4/5, yellow on basal 1/5. Head: vertex smooth, rarely with weak striations around ocelli; frons smooth; face smooth; temple in dorsal view broad, somewhat bulging behind eye, width greater than 1/2 eye width; malar space greater than 1/4 eye height, nearly equal to 1/2 eye height; ocell-ocular distance greater than 2.5 times diameter of lateral ocellus; 13–14 flagellomeres. Mesosoma: mesoscutal lobes weakly granulate and partially smooth, lateral lobes entirely hairy; notauli scrobiculate, meeting posteriorly in triangular costate area; scutellum weakly granulate or smooth; prescutellar furrow with 1 cross carina; mesopleuron smooth; precoxal sulcus smooth, shorter than mesopleuron; venter smooth; propodeum with basal median areas margined, weakly granulate or smooth, basal median carina present, areola distinctly margined, areolar area rugose, lateral areas entirely rugose. Wings: fore wing vein r nearly as long as vein 3RSa and nearly equal to width of stigma, vein 1cu-a beyond vein 1M; hind wing vein SC+R present, vein M+CU shorter than vein 1M. Metasoma: first tergum longitudinally costate, rugose medially at base, length equal to apical width; second tergum longitudinally costate; anterior transverse groove weak or absent; posterior transverse groove absent; third tergum entirely smooth; terga 4–7 smooth; ovipositor 3/4 length of metasoma.

#### Holotype female.

Top label (white, printed) - Costa Rica: San Jose [;] Zurqui de Moravia [;] 1600m. iii.1992 [;] Col. Paul Hanson; second label (red, partially printed and hand written) - HOLOTYPE [;] Heterospilus [;] athena [;] P. Marsh. Deposited in ESUW.

#### Paratypes.

1 ♀, same data as holotype with date of vi.1992 (ESUW).

#### Comments.

The shore antenna, densely hairy mesoscutum, single cross carina in prescutellar furrow and the distinctly margined areola on the propodeum are distinctive for this species.

#### Etymology.

Named for Athena, the Greek goddess of wisdom.

**Figure 212.**
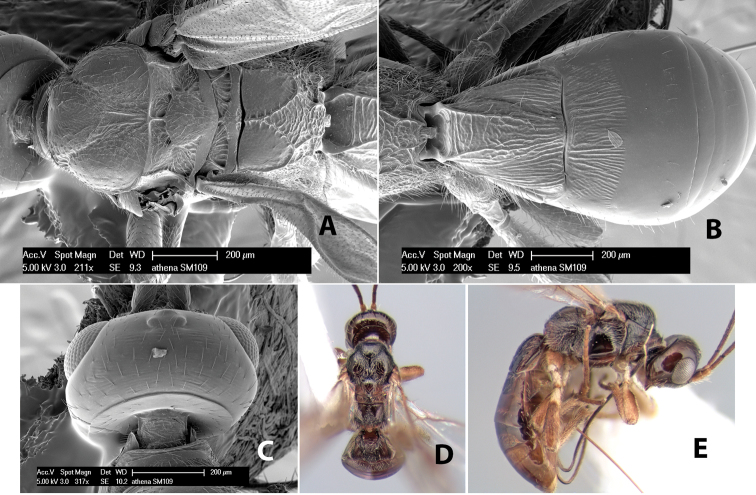
*Heterospilus athena* Marsh, sp. n.: **A–C** paratype **D–E** holotype.

### Heterospilus attraholucus

Marsh sp. n.

http://zoobank.org/EAF84589-86D5-4FFB-A21A-18C04F75C26C

http://species-id.net/wiki/Heterospilus_attraholucus

Figure 213

#### Female.

Body size: 2.25 mm. Color: head with vertex and temple dark brown, face light brown; mesosoma dark brown; metasomal terga honey yellow to light brown, tergum 2 yellow; scape yellow without lateral brown stripe; flagellum brown, basal flagellomeres yellow; wing veins including stigma brown; legs yellow. Head: vertex smooth; frons smooth; face smooth; temple in dorsal view narrow, sloping behind eye, width less than 1/2 eye width; malar space greater than 1/4 eye height; ocell-ocular distance about 2.5 times diameter of lateral ocellus; 19 flagellomeres. Mesosoma: mesoscutal lobes granulate; notauli scrobiculate, meeting posteriorly in triangular rugose area; scutellum granulate; prescutellar furrow with 1 cross carina; mesopleuron granulate; precoxal sulcus scrobiculate, shorter than mesopleuron; venter granulate; propodeum with basal median areas margined, granulate, basal median carina very short but distinct, areola not margined, areolar area rugose, lateral areas entirely rugose. Wings: fore wing vein r slightly longer than vein 3RSa, vein 1cu-a beyond vein 1M; hind wing vein SC+R absent, vein M+CU shorter than vein 1M. Metasoma: first tergum longitudinally costate, raised median area smooth or weakly granulate between carinae, length equal to apical width; second tergum longitudinally costate; anterior transverse groove present, straight; posterior transverse groove present; third tergum smooth, except for costate transverse groove; terga 4–7 smooth; ovipositor about half as long as metasoma.

#### Holotype female.

Top label (white, printed) - Costa Rica, Guanacaste Pr. [;] Guan. Conservation Area [;] Santa Rosa hdq., 200m [;] lighttrap, 6-VII 1997 [;] L.J. van der Ent; second label (red, partially printed and hand written) - HOLOTYPE [;] Heterospilus [;] attraholucus [;] P. Marsh. Deposited in ESUW.

#### Paratypes.

1 ♀, top label - Costa Rica: Guanacaste [;] Santa Rosa Natl. Park [;] 300m, ex. Malaise trap [;] Site #: SE-C-6 [;] Dates: 29.xi–20.xii.1986 [;] I.D. Gauld & D. Janzen; second label - [SE] Bosque San Emilio [;] 50yr old deciduous forest [;] [C] more or less fully [;] shaded as possible (ESUW). 3 ♀♀, S.RosaPark, Guan. [;] C. Rica 11 Sep 77, 29 Oct 77 and 6 Nov 77 [;] D.H. Janzen [;] Dry Hill and Riparian (AEIC).

#### Comments.

The longer fore wing vein r, the absence of hind wing vein SC+R and the lighter colored metasomal terga are distinctive for this species.

#### Etymology.

The specific name is from the Latin *attraho*, meaning draw to, and the Latin *lucis*, meaning light, in reference to the holotype being attracted to and caught in a light trap.

**Figure 213.**
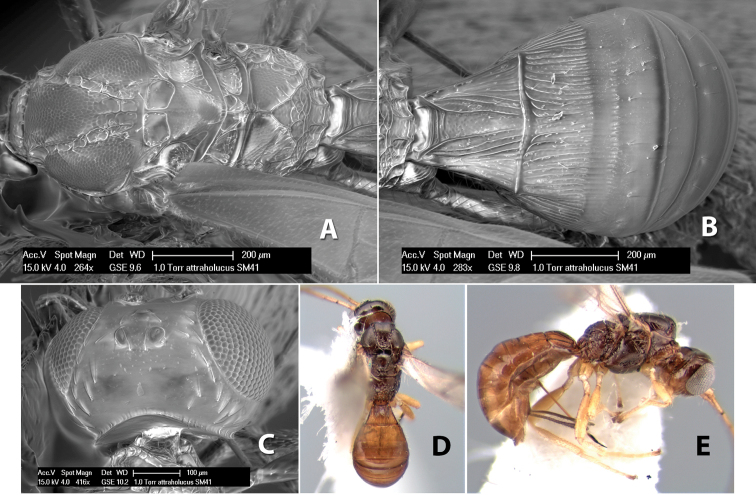
*Heterospilus attraholucus* Marsh, sp. n., holotype.

### Heterospilus borucas

Marsh sp. n.

http://zoobank.org/3AB93B2E-F6B3-4A09-97B5-1D602EB3DA03

http://species-id.net/wiki/Heterospilus_borucas

Figure 214

#### Female.

Body size: 3.0 mm. Color: head with vertex and frons brown, face honey yellow; scape yellow without lateral brown stripe; flagellum brown; mesosoma brown; metasomal terga 1–3 brown, terga 4–7 yellow; wing veins including stigma brown; legs yellow, apical half of hind femur, tibia and tarsus light brown. Head: vertex smooth; frons smooth or very weakly striate; face smooth; temple in dorsal view narrow, sloping behind eye, width less than 1/2 eye width; malar space greater than 1/4 eye height; ocell-ocular distance twice diameter of lateral ocellus; 22 flagellomeres. Mesosoma: mesoscutal lobes smooth; notauli scrobiculate, meeting posteriorly in triangular rugose area; scutellum smooth; prescutellar furrow with 3 cross carinae; mesopleuron smooth; precoxal sulcus smooth, shorter than mesopleuron; venter smooth; propodeum with basal median areas margined, smooth, basal median carina present but short, about as long as prescutellar cross carinae, areola weakly margined, areolar area rugose, lateral areas entirely rugose. Wings: fore wing vein r slightly shorter than vein 3RSa, vein 1cu-a beyond vein 1M; hind wing vein SC+R present, vein M+CU shorter than vein 1M. Metasoma: first tergum longitudinally costate, rugose medially at base, length greater than apical width; second tergum longitudinally costate; anterior transverse groove present, straight; posterior transverse groove weakly impressed or absent; third tergum costate basally, smooth apically; terga 4–7 smooth; ovipositor as long as metasoma.

#### Holotype female.

Top label (white, partially printed and hand written) - Costa Rica: Guanacaste [;] Santa Rosa Natl. Park [;] 300m, ex. Malaise trap [;] Site #: BH-12-C [;] Dates: 8.ii–2.iii 1986 [;] I.D. Gauld & D. Janzen; second label (white, printed) - [BH] Bosque Humedo [;] mature evergreen dry forest [;] [C] more or less fully [;] shaded as possible; third label (red, partially printed and hand written) - HOLOTYPE [;] Heterospilus [;] borucas [;] P. Marsh. Deposited in ESUW.

#### Paratypes.

1 ♀, COSTA RICA: [;] San Jose [;] Ciudad Colon, 800m [;] xii 1989 - i 1990 [;] Luis Fournier (ESUW).

#### Comments.

The smooth mesoscutum, long ovipositor, brown flagellum and bicolored hind leg are distinctive for this species.

#### Etymology.

Named for the Borucas, an indigenous people of Costa Rica.

**Figure 214.**
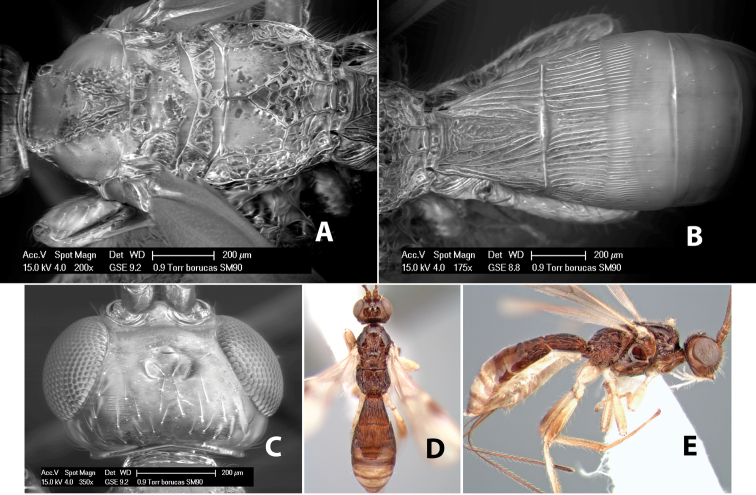
*Heterospilus borucas* Marsh, sp. n., holotype.

### Heterospilus breviarius

Marsh sp. n.

http://zoobank.org/0476D647-719E-40B4-8C95-92B868C59B3A

http://species-id.net/wiki/Heterospilus_breviarius

Figure 215

#### Female.

Body size: 2.0 mm. Color: head with vertex and frons brown, face yellow; scape yellow without lateral brown stripe; flagellum brown, basal 1–3 flagellomeres yellow; mesosoma and metasoma brown; wing veins including stigma light brown; legs yellow except femora light brown. Head: vertex smooth; frons smooth; face smooth but weakly striate medially; temple in dorsal view broad, width equal to 1/2 eye width; malar space greater than 1/4 eye height; ocell-ocular distance slightly greater than 2.5 times diameter of lateral ocellus; 13 flagellomeres. Mesosoma: mesoscutal lobes weakly granulate or smooth; notauli scrobiculate, meeting posteriorly in triangular costate area; scutellum smooth; prescutellar furrow with 1 cross carina; mesopleuron smooth; precoxal sulcus scrobiculate, extending to posterior margin of mesopleuron by distinct striae; venter smooth; propodeum with basal median areas margined, granulate, basal median carina absent, areola not margined, areolar area rugose, lateral areas entirely rugose. Wings: fore wing vein r shorter than vein 3RSa, vein 1cu-a slightly beyond vein 1M; hind wing vein SC+R present, vein M+CU shorter than vein 1M. Metasoma: first tergum longitudinally costate-granulate, length greater than apical width; second tergum longitudinally costate-granulate; anterior transverse groove weak, straight; posterior transverse groove absent; third tergum entirely smooth; terga 4–7 smooth; ovipositor as long as metasoma.

#### Holotype female.

Top label (white, partially printed and hand written) - Costa Rica: Guanacaste [;] Santa Rosa Natl. Park [;] 300m, ex. Malaise trap [;] Site #: H-2-O [;] Dates: 20 XII 86-10 I 1987 [;] I.D. Gauld & D. Janzen; second label (white, printed) - [H] open regenerating [;] woodland <10 years old [;] [O] in clearing, fully [;] isolated part of day; third label (red, partially printed and hand written) - HOLOTYPE [;] Heterospilus [;] breviarius [;] P. Marsh. Deposited in ESUW.

#### Paratypes.

Known only from the holotype.

#### Comments.

The short antennae, long ovipositor and single cross carina in the prescutellar furrow are distinctive for this species.

#### Etymology.

The specific name is from the Latin *breviarius*, meaning shortened, in reference to the short antennae.

**Figure 215.**
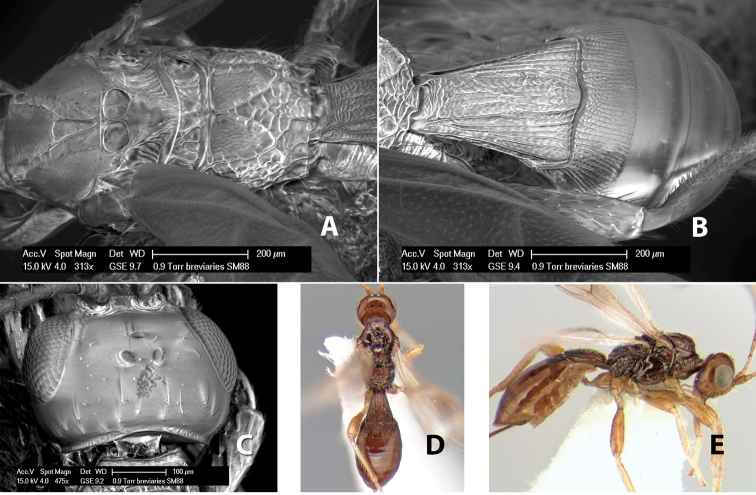
*Heterospilus breviarius* Marsh, sp. n., holotype.

### Heterospilus brevicornus

Marsh sp. n.

http://zoobank.org/FEDF7DD0-C56D-455A-BE05-034CEA312AE0

http://species-id.net/wiki/Heterospilus_brevicornus

Figure 216

#### Female.

Body size: 1.5–2.0 mm. Color: body light to medium brown, metasomal tergum 2 often yellow; scape yellow without lateral brown stripe, flagellum brown, basal flagellomeres usually lighter; wing veins light brown or translucent, stigma brown; legs yellow. Head: vertex smooth; frons smooth; face smooth; temple in dorsal view broad, slightly bulging behind eye, width equal to 1/2 eye width; malar space equal to 1/4 eye height; ocell-ocular distance greater than 2.5 times diameter of lateral ocellus; 12–15, rarely 17, flagellomeres. Mesosoma: often flattened dorso-ventrally; mesoscutal lobes weakly granulate; notauli weakly scrobiculate, meeting posteriorly in small triangular weakly rugose area; scutellum weakly granulate; prescutellar furrow usually with 3 cross carinae often median carina stronger than lateral carinae; mesopleuron smooth; precoxal sulcus smooth, shorter than mesopleuron; venter smooth; propodeum with basal median areas distinct but often not margined, granulate, basal median carina absent, areola not margined, areolar area rugose, lateral areas entirely rugose. Wings: fore wing vein r as long as vein 3RSa, vein 1cu-a beyond vein 1M; hind wing vein SC+R absent, vein M+CU as long as vein 1M. Metasoma: first tergum longitudinally costate, length equal to apical width; second tergum longitudinally costate; anterior transverse groove weakly indicated or absent, straight medially, often curved forward laterally; posterior transverse groove weakly indicated or absent; third tergum smooth; terga 4–7 smooth; ovipositor half as long as metasoma.

#### Holotype female.

Top label (white, partially printed and hand written) - Costa Rica: Guanacaste [;] Santa Rosa Natl. Park [;] 300m, ex. Malaise trap [;] Site #: H-1-O [;] Dates: 29.xi–20.xii.1986 [;] I.D. Gauld & D. Janzen; second label (white, printed) - [H] open regenerating [;] woodland <10 years old [;] [O] in clearing fully [;] isolated part of day; third label (red, partially printed and hand written) - HOLOTYPE [;] Heterospilus [;] brevicornus [;] P. Marsh. Deposited in ESUW.

#### Paratypes.

4 ♀♀, same data as holotype with additional site # H-3-O, and dates 21.ii–14.iii.1987 and 10–31.i.1987 (ESUW). 1 ♀, top label - Costa Rica: Guanacaste [;] Santa Rosa Natl. Park [;] 300m, ex. Malaise trap [;] Site #: H-2-C [;] Dates: 8–29.xi.1986 [;] I.D. Gauld & D. Janzen; second label - [H] open regenerating [;] woodland <10 years old [;] [C] more or less fully [;] shaded as possible (ESUW). 1 ♀, top label - Costa Rica: Guanacaste [;] Santa Rosa Natl. Park [;] 300m, ex. Malaise trap [;] Site #: blank [;] Dates: 18.x–8.xi.1986 [;] I.D. Gauld & D. Janzen; second label - [BH] Bosque Humedo [;] mature evergreen dry forest [;] [C] more or less fully [;] shaded as possible (ESUW). 1 ♀, top label - Costa Rica: Guanacaste [;] Santa Rosa Natl. Park [;] 300m, ex. Malaise trap [;] Site #: SE-6-C [;] Dates: 20.xii.86–10.i.1987 [;] I.D. Gauld & D. Janzen; second label - [SE] Bosque San Emilio [;] 50yr old deciduous forest [;] [C] more or less fully [;] shaded as possible (ESUW). 2 ♀♀, top label - Costa Rica: Guanacaste [;] Santa Rosa Natl. Park [;] 300m, ex. Malaise trap [;] Site #: SE-5-O [;] Dates: 6–27.ix.1986 [;] I.D. Gauld & D. Janzen; second label - [SE] Bosque San Emilio [;] 50yr old deciduous forest [;] [O] in clearing, fully [;] isolated part of day (ESUW). 1 ♀, top label - Costa Rica: Guanacaste, Santa [;] Rosa Nat’l. Park, Bosque San [;] Emilio, trap #7 in clearing, 300m. [;] II/8-III/2/1986, I. Gauld; second label - [SE] Bosque San Emilio [;] 59yr old deciduous forest [;] [O] in clearing, fully [;] isolated part of day (ESUW). 1 ♀, top label - Costa Rica: Guanacaste, Santa [;] Rosa Nat’l. Park, Bosque San [;] Emilio, trap #9 in clearing, 300m. [;] XII/7–28/1985, I. Gauld; second label - [BH] Bosque Humedo [;] mature evergreen dry forest [;] [O] in clearing, fully [;] isolated part of day (ESUW). 1 ♀, Costa Rica: Guanacaste [;] Santa Rosa Natl. Park [;] 300m, ex. Malaise trap [;] Site #: blank [;] Dates: 23.iii–13.iv.1986 [;] I.D. Gauld & D. Janzen (ESUW). 1 ♀, Costa Rica, Guanacaste Pr. [;] Guan. Conservation Area [;] Santa Rosa Hdq., 200m [;] Malaise trap 22–26 VII 1997 [;] 3x night L.J. van der Ent (ESUW). 1 ♀, COSTA RICA: San Jose, [;] Cerro de la Muerte, [;] 26km N San Isidro, 2100m, [;] ii-v 1992 [;] Paul Hanson (ESUW). 1 ♀, Costa Rica: San Jose [;] Cerro de la Muerte [;] 6Km. N. San Gerado [;] 2800m, xii.1992-ii.1993 [;] Paul Hanson (ESUW). 1 ♀ top label - COSTA RICA: Heredia [;] Pr: La Selva Biol. Sta. [;] 3km S Pto. Viejo [;] 10°26'N, 84°01'W; second label - 19.v.1990 [;] H.A. Hespenheide [;] on dead Citrus (ESUW). 1 ♀, Costa Rica: Puntarenas [;] Est. Altmira, 1300–1450m [;] 1km S del Cerro Biolley [;] 23.viii–13.ix.1996, Malaise [;] L.S. 331700-572100 #44870 [;] R. Villalobos (ESUW). 1 ♀, Costa Rica: Puntarenas [;] Pen. Osa, Puerto Jimenez [;] 10m, August 1991, full sun, [;] grassy & weedy site [;] P. Hanson, ex. Malaise (ESUW). 1 ♀, S.RosaPark, Guan. [;] C. Rica 25 Jul 77 [;] D.H. Janzen [;] Dry Hill (AEIC).

#### Comments.

The short antennae, small body and flattened mesosoma are distinctive for this species.

#### Etymology.

The specific name is from the Latin *brevis*, meaning short, and the Latin *cornu*, meaning horn, in reference to the short antennae.

**Figure 216.**
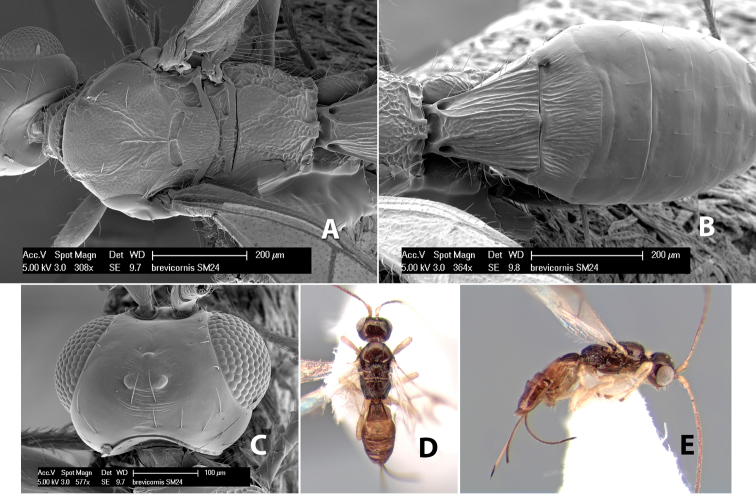
*Heterospilus brevicornus* Marsh, sp. n.: **A–C** paratype **D–E** holotype.3

### Heterospilus cabecares

Marsh sp. n.

http://zoobank.org/5CA08593-299B-4F77-8941-F2052B0DB2FA

http://species-id.net/wiki/Heterospilus_cabecares

Figure 217

#### Female.

Body size: 2.0–2.5 mm. Color: head and mesosoma dark brown, metasoma brown to dark brown, tergum 2 yellow with lateral edges dark brown; scape yellow without lateral brown stripe; flagellum brown with apical 3–5 flagellomeres white, apical one sometimes dark; wing veins including stigma brown; legs yellow. Head: vertex smooth; frons smooth; face granulate; temple in dorsal view narrow, width less than 1/2 eye width; malar space equal to 1/4 eye height; ocell-ocular distance greater than 2.5 times diameter of lateral ocellus; 18–19 flagellomeres. Mesosoma: mesoscutal lobes granulate; notauli scrobiculate, meeting posteriorly in triangular rugose-costate area; scutellum granulate; prescutellar furrow with 1 cross carina; mesopleuron granulate; precoxal sulcus weakly scrobiculate, shorter than mesopleuron; venter granulate; propodeum with basal median areas margined, granulate, basal median carina present, short, areola not margined, areolar area rugose, lateral areas rugose apically, granulate basally. Wings: fore wing vein r shorter than vein 3RSa, vein 1cu-a beyond vein 1M; hind wing vein SC+R present, vein M+CU shorter than vein 1M. Metasoma: first tergum longitudinally costate, length equal to apical width; second tergum longitudinally costate; anterior transverse groove present, straight; posterior transverse groove weak or absent; third tergum smooth; terga 4–7 smooth; ovipositor as long as metasomal tergum 1.

#### Holotype female.

Top label (white, partially printed and hand written) - Costa Rica: Guanacaste [;] Santa Rosa Natl. Park [;] 300m, ex. Malaise trap [;] Site #BH-9-O [;] Dates: 8.ii–2.iii 1986 [;] I.D. Gauld & D. Janzen; second label (white, printed) - [BH] Bosque Humedo [;] mature evergreen dry forest [;] [O] in clearing, fully [;] isolated part of day; third label (red, partially printed and hand written) - HOLOTYPE [;] Heterospilus [;] cabecares [;] P. Marsh. Deposited in ESUW.

#### Paratypes.

2 ♀♀, same data as holotype with additional date of 18.i–8.ii 1986 (ESUW). 1 ♀, top label - Costa Rica: Guanacaste [;] Santa Rosa Natl. Park [;] 300m, ex. Malaise trap [;] Site #: (blank) [;] Dates: 1–28.vii 1985 [;] I.D. Gauld & D. Janzen; second label - [SE] Bosque San Emilio [;] 50yr old deciduous forest [;] [C] more or less fully [;] shaded as possible (ESUW). 1 ♀, top label - Costa Rica: Guanacaste [;] Santa Rosa Natl. Park [;] 300m, ex. Malaise trap [;] Site #: (blank) [;] Dates: 18.i–8.ii.1986 [;] I.D. Gauld & D. Janzen; second label - [BH] Bosque Humedo [;] mature evergreen dry forest [;] [C] more or less fully [;] shaded as possible (ESUW). 1 ♀, top label - Costa Rica: Guanacaste [;] Santa Rosa National Pk. [;] 300m, Malaise trap, Ian Gauld [;] 31.i–21.ii 1987; second label - Bosque Humedo [;] mature dry forest [;] high proportion evergreen species [;] Full shade (ESUW). 1 ♀, top label - Costa Rica: Guanacaste [;] Santa Rosa National Pk. [;] 300m, Malaise trap, Ian Gauld [;] 31.i–21.ii 1987; second label - Bosque San Emilio [;] 50 yr. Old deciduous [;] Forest, Sun (ESUW). 3 ♀♀, S.RosaPark, Guan. [;] C. Rica 12 Dec. 76, 8 Jan. 77 and 16 May 77 [;] D. H. Janzen [;] Riparian and Dry Hill (AEIC). 1 ♀, top label - LaLola,C.R. [;] VI-5 1957 [;] MJStelzer [;] MJS 57-235; second label - On cacao (AEIC).

#### Comments.

The short ovipositor, smooth vertex and yellow metasomal tergum 2 are distinctive for this species.

#### Etymology.

Named for the Cabecares, an indigenous people of Costa Rica.

**Figure 217.**
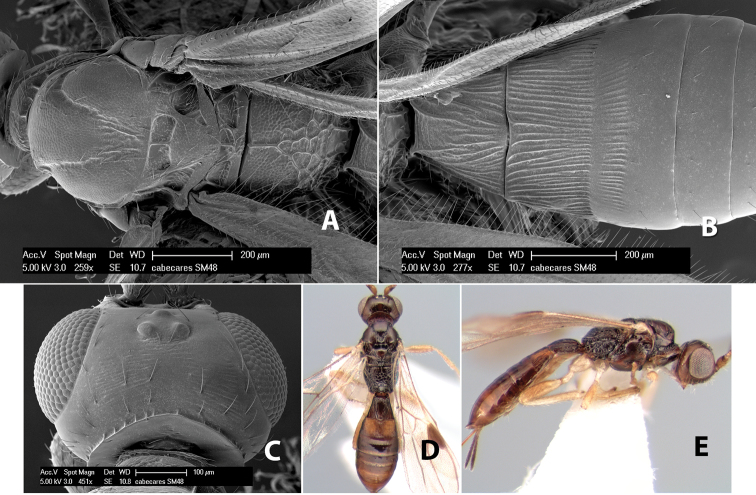
*Heterospilus cabecares* Marsh, sp. n.: **A–C** paratype **D–E** holotype.

### Heterospilus careonotaulus

Marsh sp. n.

http://zoobank.org/E2A75673-69A5-4236-A516-19003206EFE6

http://species-id.net/wiki/Heterospilus_careonotaulus

Figure 218

#### Female.

Body size: 1.0–1.5 mm. Color: body dark brown, metasomal tergum 1 usually yellow; scape yellow without lateral brown stripe; flagellum brown, basal flagellomeres yellow, apical 3–5 flagellomeres white; wing veins including stigma brown; legs yellow. Head: vertex smooth; frons smooth; face smooth; temple in dorsal view narrow, sloping behind eye, width equal to 1/2 eye width; malar space greater than 1/4 eye height; ocell-ocular distance greater than 2.5 times diameter of lateral ocellus; 11–12 flagellomeres. Mesosoma: mesoscutal lobes weakly granulate or smooth; notauli absent posteriorly, weakly present and smooth anteriorly; scutellum weakly granulate or smooth; prescutellar furrow with 1 cross carina; mesopleuron smooth; precoxal sulcus scrobiculate, shorter than mesopleuron; venter smooth; propodeum with basal median areas margined, smooth, basal median carina present, areola narrow, distinctly margined, areolar area broadly rugose, lateral areas entirely rugose. Wings: fore wing vein r slightly shorter than vein 3RSa and longer than vein r-m, vein 1cu-a interstitial with vein 1M; hind wing vein SC+R absent, vein M+CU shorter than vein 1M. Metasoma: first tergum weakly costate, often partially smooth, length greater than apical width; second tergum smooth; anterior transverse groove absent; posterior transverse groove absent; third tergum smooth; terga 4–7 smooth; ovipositor as long as metasomal terga 1 and 2 combined.

#### Holotype female.

Top label (white, printed) - Costa Rica, San Jose [;] Zurqui de Moravia [;] 1600m, ix.1995 [;] P. Hanson; second label (red, partially printed and hand written) - HOLOTYPE [;] Heterospilus [;] careonotaulus [;] P. Marsh. Deposited in ESUW.

#### Paratypes.

2 ♀♀, same data as holotype with additional dates of IV/1989 and vi-vii 1989 (ESUW). 1 ♀, COSTA RICA, San Jose [;] P.N.BraulioCarillo [;] 9.5Km E tunnel, 1000m [;] VI/1989, col. Hanson (ESUW).

#### Comments.

The small size, incomplete notauli, smooth metasomal tergum 1 and the white apex of the flagellum are distinctive for this species.

#### Etymology.

The specific name is from the Latin *careo*, meaning to be without, in reference to the incomplete or absent notauli.

**Figure 218.**
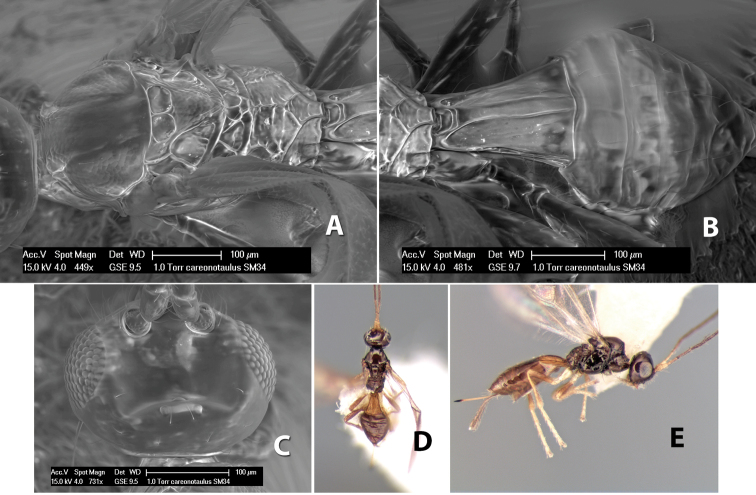
*Heterospilus careonotaulus* Marsh, sp. n.: **A–C, E** paratype **D** holotype.

### Heterospilus catiensis

Marsh sp. n.

http://zoobank.org/41CA6F62-91F2-4161-AEA6-FE18619E4BDD

http://species-id.net/wiki/Heterospilus_catiensis

Figure 219

#### Female.

Body size: 3.0 mm. Color: head, mesosoma and metasomal tergum brown, metasomal terga 2–7 light brown; scape yellow without lateral brown stripe; flagellum brown with apical 5–7 flagellomeres white, apical one sometimes dark; wing veins light brown, stigma light brown or yellow; legs yellow. Head: vertex smooth; frons smooth; face smooth; temple in dorsal view narrow, sloping behind eye, width slightly less than 1/2 eye width; malar space greater than 1/4 eye height; ocell-ocular distance greater than 2.5 times diameter of lateral ocellus; 26 flagellomeres. Mesosoma: mesoscutal lobes very weakly granulate or smooth; notauli scrobiculate, meeting posteriorly in triangular costate area; scutellum smooth; prescutellar furrow with 3 cross carinae; mesopleuron smooth; precoxal sulcus smooth, shorter than mesopleuron; venter smooth; propodeum with basal median areas margined, smooth, basal median carina present, areola distinctly margined, areolar area broadly rugose, lateral areas entirely rugose. Wings: fore wing vein r shorter than vein 3RSa, vein 1cu-a beyond vein 1M; hind wing vein SC+R present, vein M+CU shorter than vein 1M. Metasoma: first tergum longitudinally costate, length twice apical width; second tergum longitudinally costate; anterior transverse groove present, straight; posterior transverse groove present; third tergum costate basally, smooth apically; terga 4–7 smooth; ovipositor longer than metasoma.

#### Holotype female.

Top label (white, printed) - Costa Rica: Cartago [;] Turrialba, CATIE [;] 14–15.iii.1990 [;] 700m, J.S. Noyes; second label (red, partially printed and hand written) - HOLOTYPE [;] Heterospilus [;] catiensis [;] P. Marsh. Deposited in ESUW.

#### Paratypes.

Known only from the holotype.

#### Comments.

This species is distinguished by the costate metasomal tergum 3 and the 3 cross carinae in the prescutellar furrow.

#### Etymology.

Named for CATIE, the Centro Agronómico Tropical de Investigación y Enseñanza (Tropical Agriculture Research and Higher Education Center) in Turrialba, Cartago Province.

**Figure 219.**
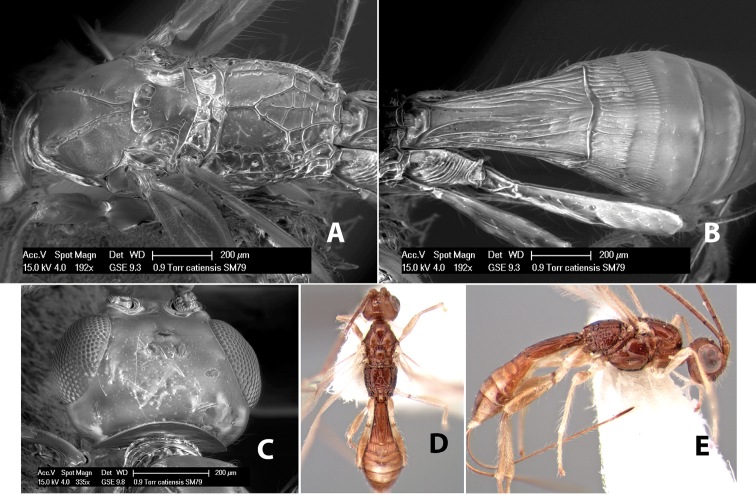
*Heterospilus catiensis* Marsh, sp. n., holotype.

### Heterospilus catorce

Marsh sp. n.

http://zoobank.org/A3874FFE-0219-46B4-869B-1FD40271AE6D

http://species-id.net/wiki/Heterospilus_catorce

Figure 220

#### Female.

Body size: 3.0 mm. Color: head with vertex and frons dark brown, face honey yellow; mesosoma dark brown; metasomal terga 1 and 2 dark brown, remainder of terga dark brown basally, dark yellow apically; scape yellow with lateral longitudinal brown stripe; flagellum brown with apical white annulus, apical 3–5 flagellomeres brown; legs yellow; wing veins including stigma brown. Head: vertex smooth; frons smooth; face smooth; temple in dorsal view narrow, sloping behind eye, width less than 1/2 eye width; malar space about equal to 1/4 eye height; ocell-ocular distance 2.5 times diameter of lateral ocellus; 26 flagellomeres. Mesosoma: mesoscutal lobes smooth; notauli weakly scrobiculate anteriorly, smooth posteriorly, meeting posteriorly in rectangular costate area; scutellum smooth; prescutellar furrow with 3 cross carinae; mesopleuron smooth; precoxal sulcus smooth, shorter than mesopleuron; venter smooth; propodeum with basal median areas margined, smooth, basal median carina present, areola distinctly margined, areolar area rugose, lateral areas rugose apically, smooth basally. Wings: fore wing vein r shorter than vein 3RSa, vein 1cu-a beyond vein 1m; hind wing vein SC+R present, vein M+CU shorter than vein 1M. Metasoma: first tergum longitudinally costate, length equal to apical width; second tergum longitudinally costate, narrow, width about 4 times length; anterior transverse groove present, very slightly sinuate; posterior transverse groove present; third tergum costate basally, weakly granulate apically; terga 4–7 weakly granulate basally, nearly smooth apically; ovipositor about 3/4 length of metasoma.

#### Holotype female.

Top label (white, partially printed and hand written) - Costa Rica: Guanacaste [;] Santa Rosa National Pk. [;] 300m, Malaise trap, Ian Gauld [;] 26.vii–14.viii 1986; second label (white, partially printed and hand written) - Bosque San Emelio [;] 50yr. old deciduous [;] forest, [;] Full Shade; third label (white, printed) - SE-8-C [;] 26.vii–14.viii.86; fourth label (red, partially printed and hand written) - HOLOTYPE [;] Heterospilus [;] catorce [;] P. Marsh. Deposited in ESUW.

#### Paratypes.

Known only from the holotype.

#### Comments.

The smooth mesoscutum, rectangular costate area where notauli meet, the narrow metasomal tergum 2 and the white annulus on the flagellum are distinctive for this species.

#### Etymology.

The specific name is an arbitrary combination of letters.

**Figure 220.**
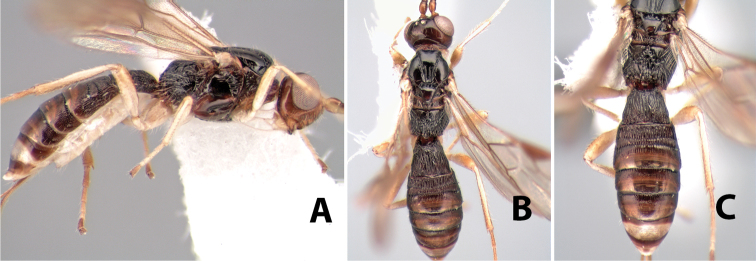
*Heterospilus catorce* Marsh, sp. n., holotype.

### Heterospilus cero

Marsh sp. n.

http://zoobank.org/47AD3EDD-53C4-4A1A-90E5-C2D1F68ED03E

http://species-id.net/wiki/Heterospilus_cero

Figure 221

#### Female.

Body size: 2.0–2.5 mm. Color: body dark brown; scape yellow without lateral brown stripe; flagellum brown; wing veins including stigma brown; legs yellow. Head: vertex smooth; frons smooth; face smooth; temple in dorsal view broad but sloping behind eye, width equal to 1/2 eye width; malar space greater than 1/4 eye height; ocell-ocular distance slightly greater than 2.5 times diameter of lateral ocellus; 18–21 flagellomeres. Mesosoma: mesoscutal lobes weakly granulate; notauli weakly scrobiculate, meeting posteriorly in triangular costate area; scutellum smooth; prescutellar furrow with 3 cross carinae; mesopleuron granulate; precoxal sulcus smooth, shorter than mesopleuron; venter smooth; propodeum with basal median areas margined, granulate, basal median carina present, short, areola distinctly margined, areolar area rugose, lateral areas entirely rugose. Wings: fore wing vein r slightly shorter than vein 3RSa, vein 1cu-a beyond vein 1M; hind wing vein SC+R present, vein M+CU shorter than vein 1M. Metasoma: first tergum longitudinally costate-granulate, length equal to apical width; second tergum longitudinally costate; anterior transverse groove weak or apparently absent, straight; posterior transverse groove weak or apparently absent; third tergum costate basally, smooth apically; terga 4–7 smooth; ovipositor as long as metasoma.

#### Holotype female.

Top label (white, printed) - Costa Rica: Guanacaste, ACT [;] Bagaces, P.N. Palo Verde [;] Sect. Palo Verde, Cerro Guaycan [;] 160m, Malaise trap [;] 15.vi–15.vii.1999, I. Jimenez [;] L.N. 259350-259350 #52850; second label (red, partially printed and hand written) - HOLOTYPE [;] Heterospilus [;] cero [;] P. Marsh. Deposited in ESUW.

#### Paratypes.

1 ♀, same data as holotype (ESUW). 1 ♀, Costa Rica: Guanacaste, ACT [;] Bagaces, P.N. Palo Verde [;] Sec. P. Verde 0–50m [;] Extremo E. Campo Aterrizaje [;] Malaise trap, #53260 [;] 17.viii–13.ix.1999, I. Jimenez [;] L.N. 260952-385020 (ESUW). 1 ♀, S.RosaPark, Guan. [;] C. Rica 9 Nov 77 [;] D.H. Janzen [;] Riparian (AEIC).

#### Comments.

The distinctly margined areola on the propodeum and the ovipositor as long as the metasoma are distinctive for this species.

#### Etymology.

The specific name is an arbitrary combination of letters.

**Figure 221.**
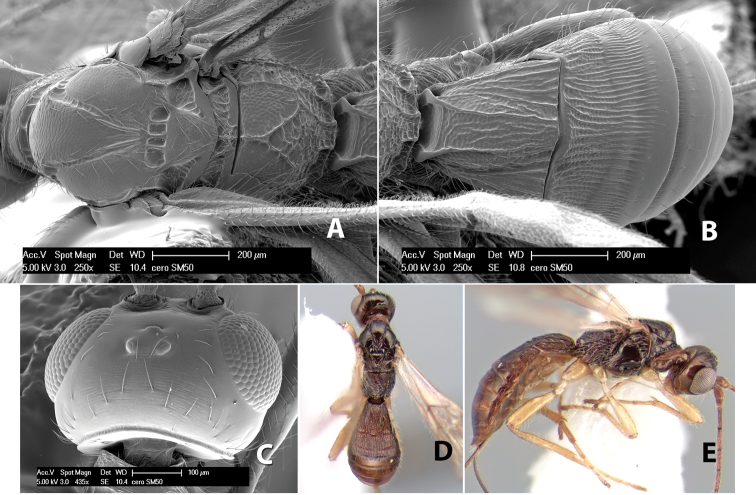
*Heterospilus cero* Marsh, sp. n.: **A–C** paratype **D–E** holotype.

### Heterospilus chorti

Marsh sp. n.

http://zoobank.org/0437B68E-DA77-4DE4-BD6D-781FBFD38300

http://species-id.net/wiki/Heterospilus_chorti

Figure 222

#### Female.

Body size: 2.0 mm. Color: head brown; scape yellow without lateral brown stripe, flagellum brown; mesosoma dark brown, mesoscutum light brown; metasomal tergum 1 dark brown, tergum 2 yellow, terga 3–6 light brown; wing veins brown, stigma bicolored brown with yellow apex; legs yellow. Head: vertex smooth; frons smooth; face smooth; temple in dorsal view broad, width equal to 1/2 eye width; malar space greater than 1/4 eye height; ocell-ocular distance about 2.5 times diameter of lateral ocellus; 17 flagellomeres. Mesosoma: mesoscutal lobes weakly granulate; notauli scrobiculate, meeting posteriorly in triangular costate area; scutellum smooth; prescutellar furrow with 1 distinct cross carina and rarely 2 very weak carinae laterally; mesopleuron granulate; precoxal sulcus smooth, shorter than mesopleuron; venter weakly granulate; propodeum with basal median areas margined, weakly granulate or smooth, basal median carina present, long, areola distinctly margined, areolar area weakly rugose, lateral areas weakly rugose. Wings: fore wing vein r shorter than vein 3RSa, vein 1cu-a interstitial with vein 1M; hind wing vein SC+R present, vein M+CU shorter than vein 1M. Metasoma: first tergum longitudinally costate, length greater than apical width; second tergum longitudinally costate; anterior transverse groove present, sinuate; posterior transverse groove absent; third tergum weakly costate at base or entirely smooth; terga 4–7 smooth; ovipositor as long as metasoma.

#### Holotype female.

Top label (white, printed) - COSTA RICA-Heredia Prov. [;] La Selva Biological Station [;] 10°26'N, 84°01'W, 100m [;] Canopy fogging 31 [;] 2.xi.1994 [;] Project ALAS (FPM31); second label (red, partially printed and hand written) - HOLOTYPE [;] Heterospilus [;] chorti [;] P. Marsh. Deposited in ESUW.

#### Paratypes.

Known only from the holotype.

#### Comments.

The distinctly margined areola on the propodeum, the sinuate anterior transverse groove on metasomal tergum 2 and the bicolored body are distinctive for this species.

#### Etymology.

Named for the Ch’orti’, a Mayan people of El Salvador.

**Figure 222.**
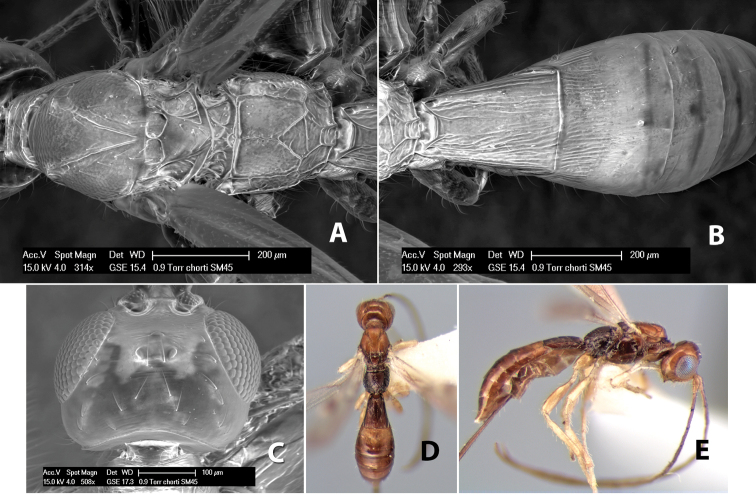
*Heterospilus chorti* Marsh, sp. n., holotype.

### Heterospilus cinco

Marsh sp. n.

http://zoobank.org/D0A64C11-6E02-40D6-A742-8D9E0FE08349

http://species-id.net/wiki/Heterospilus_cinco

Figure 223

#### Female.

Body size: 2.5 mm. Color: body dark brown, apical metasomal terga slightly lighter; scape yellow with lateral longitudinal brown stripe; flagellum brown with apical white annulus but with apical 3–5 flagellomeres brown; wing veins brown, stigma bicolored brown with yellow spot at base; legs yellow. Head: vertex smooth; frons smooth; face smooth; temple in dorsal view narrow, width slightly less than 1/2 eye width; malar space equal to 1/4 eye height; ocell-ocular distance slightly greater than 2.5 times diameter of lateral ocellus; 20 flagellomeres. Mesosoma: mesoscutal lobes granulate; notauli scrobiculate, meeting posteriorly in triangular costate area; scutellum weakly granulate; prescutellar furrow with 3 cross carinae; mesopleuron weakly granulate; precoxal sulcus smooth, shorter than mesopleuron; venter smooth; propodeum with basal median areas margined, granulate, basal median carina present but very short, areola not margined, areolar area rugose, lateral areas rugose apically, weakly granulate basally. Wings: fore wing vein r shorter than vein 3RSa, vein 1cu-a beyond vein 1M; hind wing vein SC+R present, vein M+CU shorter than vein 1M. Metasoma: first tergum longitudinally costate, length slightly greater than apical width; second tergum longitudinally costate; anterior transverse groove present, straight; posterior transverse groove present; third tergum smooth except for costate transverse groove; terga 4–7 smooth; ovipositor as long as metasoma.

#### Holotype female.

Top label (white, partially printed and hand written) - Costa Rica: Guanacaste [;] Santa Rosa Natl. Park [;] 300m, ex. Malaise trap [;] Site #: SE-C-6 [;] Dates: 29.xi–20.xii.1986 [;] I.D. Gauld & D. Janzen; second label (white, printed) - [SE] Bosque San Emilio [;] 50yr old deciduous forest [;] [C] more or less fully [;] shaded as possible; third label (red, partially printed and hand written) - HOLOTYPE [;] Heterospilus [;] cinco [;] P. Marsh. Deposited in ESUW.

#### Paratypes.

Known only from the holotype.

#### Comments.

The flagellum with a white apical annulus, the lateral brown stripe on the scape and the bicolored stigma are distinctive for this species.

#### Etymology.

The specific name is an arbitrary combination of letters.

**Figure 223.**
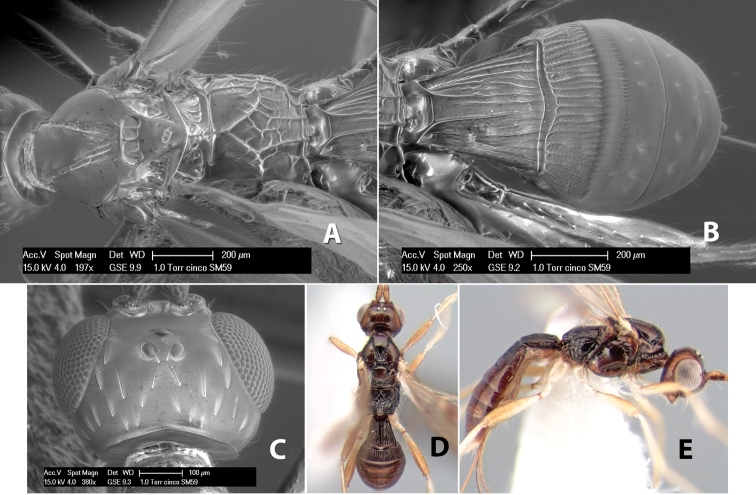
*Heterospilus cinco* Marsh, sp. n., holotype.

### Heterospilus colliletus

Marsh sp. n.

http://zoobank.org/4E57E271-1E8B-44F1-A8D4-8ED00282AAEA

http://species-id.net/wiki/Heterospilus_colliletus

Figure 224

#### Female.

Body size: 2.5 mm. Color: body dark brown, apical metasomal terga lighter; scape light brown without lateral brown stripe; flagellum brown; wing vein including stigma brown; legs yellow. Head: vertex smooth; frons smooth; face smooth; temple in dorsal view broad, slightly bulging eye, width equal to 1/2 eye width; malar space equal to 1/4 eye height; ocell-ocular distance slightly greater than 2.5 times diameter of lateral ocellus; 19 flagellomeres. Mesosoma: mesoscutal lobes granulate; notauli scrobiculate, meeting posteriorly in triangular costate area; scutellum smooth; prescutellar furrow with 1 cross carina; mesopleuron smooth; precoxal sulcus smooth, shorter than mesopleuron; venter smooth; propodeum with basal median areas margined, smooth, basal median carina present but short, areola not margined, areolar area rugose, lateral areas entirely rugose. Wings: fore wing vein r shorter than vein 3RSa, vein 1cu-a beyond vein 1M; hind wing vein SC+R present, vein M+CU slightly longer than vein 1M. Metasoma: first tergum longitudinally costate, length greater than apical width; second tergum longitudinally costate; anterior transverse groove present, straight; posterior transverse groove weakly indicated; third tergum costate basally, smooth apically; terga 4–7 smooth; ovipositor half as long as metasoma.

#### Holotype female.

Top label (white, printed) - Costa Rica: San Jose [;] Cerro de la Muerte [;] 6km. N. San Gerardo [;] 2800m, August 1992 [;] P. Hanson, Malaise; second label (red, partially printed and hand written) - HOLOTYPE [;] Heterospilus [;] colliletus [;] P. Marsh. Deposited in ESUW.

#### Paratypes.

Known only from the holotype.

#### Comments.

The single cross carina in the prescutellar furrow, granulate mesoscutal lobes, the longer hind wing vein M+CU and the dark brown body are distinctive for this species.

#### Etymology.

The specific name is from the Latin *collis*, meaning hill, and the Latin *letum*, meaning death, in reference to the type locality of Cerro de la Muerte, Hill of Death.

**Figure 224.**
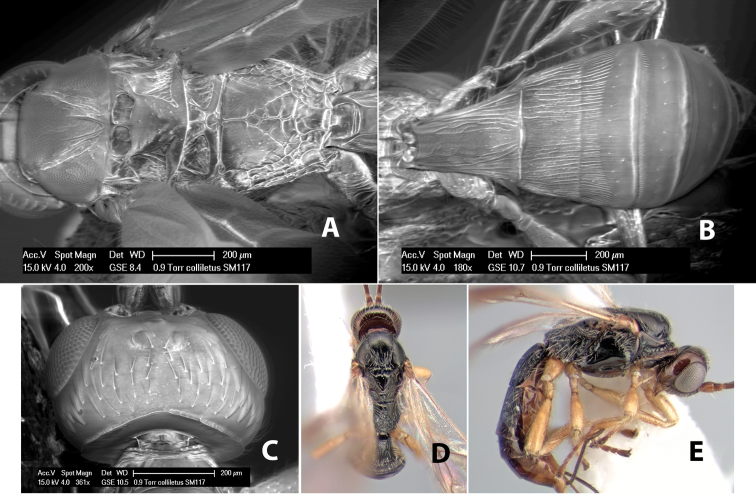
*Heterospilus colliletus* Marsh, sp. n., holotype.

### Heterospilus colonensis

Marsh sp. n.

http://zoobank.org/DA103927-40D2-47D1-8A7B-56FD573E28C7

http://species-id.net/wiki/Heterospilus_colonensis

Figure 225

#### Female.

Body size: 1.5 mm. Color: head light to medium brown; scape yellow without lateral brown stripe; flagellum brown; mesosoma light to medium brown, usually yellow along notauli; metasomal terga 1–2 yellow, remainder of terga light brown; wing veins very light brown, stigma bicolored light brown with apex and base white; legs yellow. Head: vertex smooth; frons smooth; face smooth; temple in dorsal view broad, width about 1/2 eye width; malar space greater than 1/4 eye height; ocell-ocular distance greater than 2.5 times diameter of lateral ocellus; 17–18 flagellomeres. Mesosoma: mesoscutal lobes weakly granulate; notauli weakly scrobiculate, meeting posteriorly in triangular costate area; scutellum smooth; prescutellar furrow with 1 cross carina; mesopleuron weakly granulate; precoxal sulcus smooth, shorter than mesopleuron; venter smooth; propodeum with basal median areas margined, weakly granulate or smooth, basal median carina present, areola distinctly margined, areolar area rugose, lateral areas entirely rugose. Wings: fore wing vein r shorter than vein 3RSa, vein 1cu-a beyond vein 1M; hind wing vein SC+R present, vein M+CU shorter than vein 1M. Metasoma: first tergum weakly longitudinally costate, length greater than apical width; second tergum smooth; anterior transverse groove weak or rarely absent; posterior transverse groove weak or rarely absent; third tergum smooth; terga 4–7 smooth; ovipositor longer than metasoma.

#### Holotype female.

Top label (white, printed) - COSTA RICA: [;] San Jose [;] Ciudad Colon, 800m [;] xii 1989 - i 1990 [;] Luis Fournier; second label (red, partially printed and hand written) - HOLOTYPE [;] Heterospilus [;] colonensis [;] P. Marsh. Deposited in ESUW.

#### Paratypes.

1 ♀, same data as holotype (ESUW).

#### Comments.

The long ovipositor, light colored body and bicolored stigma are distinctive for this species.

#### Etymology.

Named for the city of Colon where the type series was collected.

**Figure 225.**
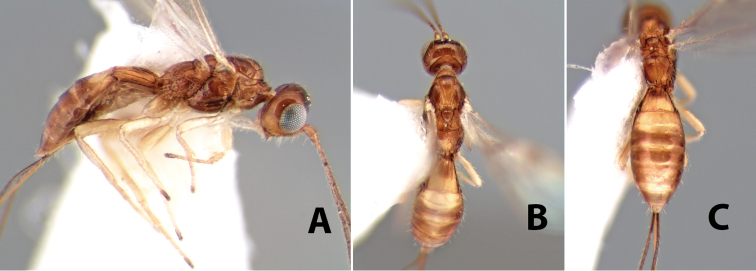
*Heterospilus colonensis* Marsh, sp. n., holotype.

### Heterospilus conservatus

Marsh sp. n.

http://zoobank.org/B42C08E0-A284-44FD-8D19-052F2172A83E

http://species-id.net/wiki/Heterospilus_conservatus

Figure 226

#### Female.

Body size: 3.0 mm. Color: head light brown; scape yellow without lateral brown stripe, flagellum brown; mesosoma dark brown, pronotum and propleuron lighter brown; metasomal tergum 1 dark brown, remainder of terga light brown; wing veins including stigma brown; legs yellow. Head: vertex smooth; frons smooth; face rugose; temple in dorsal view broad, somewhat bulging behind eye, width slightly less than 1/2 eye width; malar space about 1/4 eye height; ocell-ocular distance twice diameter of lateral ocellus; 22 flagellomeres. Mesosoma: mesoscutal lobes granulate; notauli scrobiculate, meeting posteriorly in triangular rugose area; scutellum granulate; prescutellar furrow with 1 cross carina; mesopleuron granulate; precoxal sulcus weakly scrobiculate, shorter than mesopleuron; venter granulate; propodeum with basal median areas margined, granulate, basal median carina present but very short, areola not margined, areolar area rugose, lateral areas entirely rugose. Wings: fore wing vein r longer than vein 3RSa, vein 1cu-a beyond vein 1M; hind wing vein SC+R absent, vein M+CU slightly shorter than vein 1M. Metasoma: first tergum costate-rugose, length equal to apical width; second tergum longitudinally costate; anterior transverse groove present, straight; posterior transverse groove present; third tergum weakly costate basally, smooth apically; terga 4–7 smooth; ovipositor as long as metasomal tergum 1.

#### Holotype female.

Top label (white, printed) - Costa Rica, Guanacaste Pr. [;] Guan. Conservation Area [;] Santa Rosa hdq., 200m [;] lighttrap, 6-VII 1007(1997) [;] L.J. van der Ent; second label (red, partially printed and hand written) - HOLOTYPE [;] Heterospilus [;] conservatus [;] P. Marsh. Deposited in ESUW.

#### Paratypes.

Known only from the holotype.

#### Comments.

The longer fore wing vein r, the rugose face and the light brown head are distinctive for this species.

#### Etymology.

The specific name is from the Latin *conservatus*, meaning conservation, in reference to the holotype locality of the Guanacaste Conservation Area.

**Figure 226.**
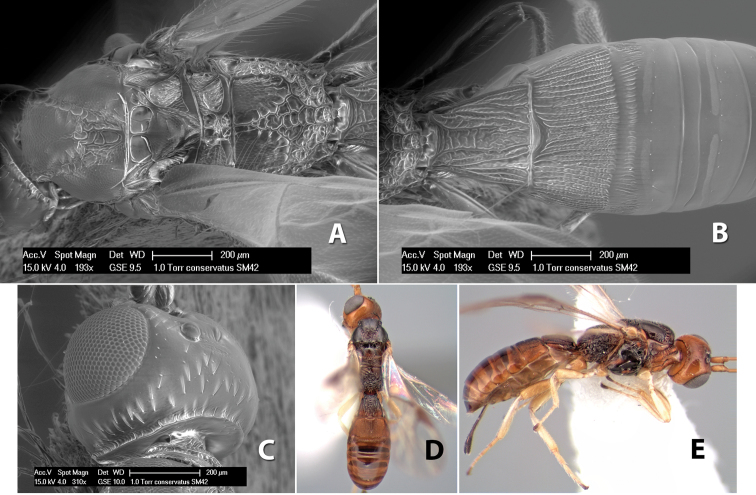
*Heterospilus conservatus* Marsh, sp. n., holotype.

### Heterospilus cuatro

Marsh sp. n.

http://zoobank.org/21A60518-E0AF-44FE-A7ED-3C75A302DA95

http://species-id.net/wiki/Heterospilus_cuatro

Figure 227

#### Female.

Body size: 2.5 mm. Color: head with vertex and temples brown, face honey yellow; scape yellow without lateral brown stripe; flagellum brown with apical 5–7 flagellomeres white; mesosoma dark brown, mesoscutal lobes yellow; metasomal terga dark brown, terga 6–7 yellow; wing veins including stigma brown; legs yellow or light brown. Head: vertex smooth; frons smooth; face smooth; temple in dorsal view narrow, width less than 1/2 eye width; malar space greater than 1/4 eye height; ocell-ocular distance about 2.5 times diameter of lateral ocellus; 24 flagellomeres. Mesosoma: mesoscutal lobes granulate; notauli scrobiculate, meeting posteriorly in triangular costate area; scutellum granulate; prescutellar furrow with 1 cross carina; mesopleuron granulate; precoxal sulcus weakly scrobiculate, shorter than mesopleuron; venter granulate; propodeum with basal median areas margined, granulate, basal median carina absent, areola not margined, areolar area rugose, lateral areas granulate basally, rugose apically; propodeum with small but distinct tubercle above hind coxa. Wings: fore wing vein r shorter than vein 3RSa, vein 1cu-a interstitial with vein 1M; hind wing vein SC+R present, vein M+CU shorter than vein 1M. Metasoma: first tergum longitudinally costate, length about twice apical width; second tergum longitudinally costate; anterior transverse groove present, straight; posterior transverse groove present; third tergum costate basally, smooth apically; terga 4–7 smooth; ovipositor longer than metasoma.

#### Holotype female.

Top label (white, partially printed and hand written) - COSTA RICA, Guanac, [;] Est. Pitilla, 9km S [;] SantaCecilia, 700m [;] IX 1988 P. Hanson; second label (red, partially printed and hand written) - HOLOTYPE [;] Heterospilus [;] cuatro [;] P. Marsh. Deposited in ESUW.

#### Paratypes.

Known only from the holotype.

#### Comments.

The yellow mesoscutal lobes, apical flagellomeres being white and the long metasomal tergum are distinctive for this species.

#### Etymology.

The specific name is an arbitrary combination of letters.

**Figure 227.**
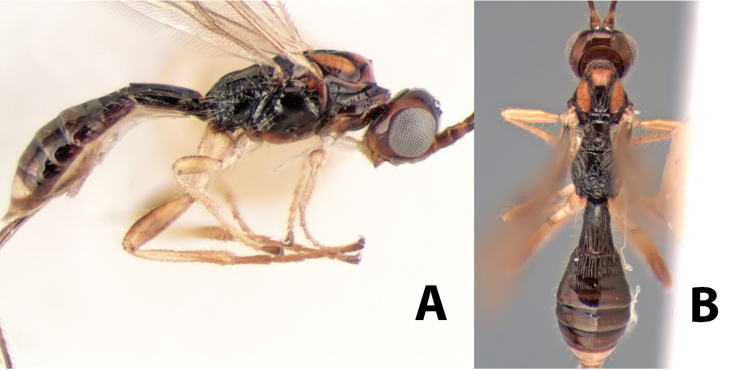
*Heterospilus cuatro* Marsh, sp. n., holotype.

### Heterospilus demeter

Marsh sp. n.

http://zoobank.org/D16E6AE9-8876-4433-A546-23AA6D70A254

http://species-id.net/wiki/Heterospilus_demeter

Figure 228

#### Female.

Body size: 2.0–2.5 mm. Color: body brown to dark brown, apical metasomal terga slightly lighter brown than terga 1 and 2; scape yellow without lateral brown stripe; flagellum brown; wing veins including stigma brown; legs yellow. Head: vertex smooth; frons smooth; face smooth; temple in dorsal view narrow, sloping behind eye, width less than 1/2 eye width; malar space equal to 1/2 eye height; ocell-ocular distance 2–2.5 times diameter of lateral ocellus; 15–19 flagellomeres. Mesosoma: mesoscutal lobes granulate; notauli scrobiculate, meeting posteriorly in triangular costate area; scutellum smooth; prescutellar furrow with 1 cross carina; mesopleuron smooth; precoxal sulcus weakly scrobiculate, shorter than mesopleuron; venter smooth; propodeum with basal median areas margined, granulate, basal median carina present, areola not margined, areolar area rugose, lateral areas entirely rugose. Wings: fore wing vein r shorter than vein 3RSa, vein 1cu-a slightly beyond vein 1M; hind wing vein SC+R present, vein M+CU shorter than vein 1M. Metasoma: first tergum longitudinally costate, length equal to apical width; second tergum longitudinally costate; anterior transverse groove present, straight; posterior transverse groove present; third tergum costate basally, smooth apically; terga 4–7 smooth; ovipositor equal to length of metasomal terga 1 and 2 combined.

#### Holotype female.

Top label (white, partially printed and hand written) - COSTA RICA: Limón [;] 7km SW Bribri, 50m [;] I-II (or 1–11?) 1990 [;] Col. Paul Hanson; second label (red, partially printed and hand written) - HOLOTYPE [;] Heterospilus [;] demeter [;] P. Marsh. Deposited in ESUW.

#### Paratypes.

1 ♀, Costa Rica, Alajuela [;] 5km. W. San Ramon [;] 1200m, I-1997 [;] O Castro & P. Hanson (ESUW). 1 ♀, Costa Rica: Puntarenas [;] Res. Forestal Golfo Dulce [;] 3km SW Rincon, 10m [;] ii.1993, P. Hanson [;] Malaise, primary forest (ESUW). 1 ♀, Costa Rica: Puntarenas [;] Peninsula Osa [;] Puerto Jimenez, 10m [;] i-ii–1992, Paul Hanson [;] grassy, weedy site (ESUW). 1 ♀, Costa Rica: San Jose [;] Zurqui de Moravia [;] 1600m, P. Hanson [;] ix.1995 (ESUW). 1 ♀, Costa Rica: Alajuela [;] Res. Biol. San Ramon [;] 800m, iv-v.1999 [;] P. Hanson, Malaise (ESUW). 2 ♀♀, top label - Costa Rica: San José [;] San Pedro de Montes de Oca [;] 1100 m8nm 25-IV-90 [;] Col. Hanson; second label - Ficus [;] costar. (MICR).

#### Comments.

The granulate mesoscutum, single cross carina in the prescutellar furrow and the brown flagellum are distinctive for this species.

#### Etymology.

Named for the Greek goddess of agriculture, Demeter.

**Figure 228.**
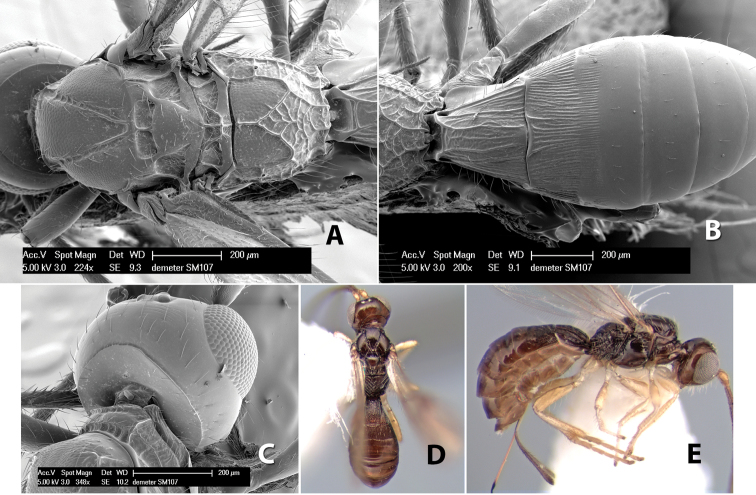
*Heterospilus demeter* Marsh, sp. n.: **A–C** paratype **D–E** holotype.

### Heterospilus diecinueve

Marsh sp. n.

http://zoobank.org/59F608A4-18B3-4E30-8AF6-1BE4E308F834

http://species-id.net/wiki/Heterospilus_diecinueve

Figure 229

#### Female.

Body size: mm. Color: body dark brown, apical metasomal terga yellow; scape brown; flagellum brown; wing veins brown, stigma yellow; legs yellow. Head: vertex smooth; frons smooth; face smooth; temple in dorsal view narrow, sloping behind eye, width about equal to 1/2 eye width; malar space greater than 1/4 eye height; ocell-ocular distance slightly greater than 2.5 times diameter of lateral ocellus; 29 flagellomeres. Mesosoma: mesoscutal lobes smooth, shining; notauli scrobiculate, meeting posteriorly in triangular costate area; scutellum smooth; prescutellar furrow with 3–5 cross carinae; mesopleuron smooth; precoxal sulcus smooth, shorter than mesopleuron; venter smooth; propodeum with basal median areas weakly margined, partially smooth and rugose, basal median carina absent, areola not margined, areolar area rugose, lateral areas entirely rugose. Wings: fore wing vein r shorter than vein 3RSa, vein 1cu-a beyond vein 1M; hind wing vein SC+R present, vein M+CU slightly shorter than vein 1M. Metasoma: first tergum longitudinally costate, length equal to apical width; second tergum longitudinally costate, width about 3.5 times length; anterior transverse groove present, slightly sinuate; posterior transverse groove present; third tergum costate basally, smooth apically; terga 4–7 smooth; ovipositor as long as metasoma.

#### Holotype female.

Top label (white, printed) - Costa Rica: Guanacaste, ACT [;] Bagaces, P.N. Palo Verde, 212m [;] Sec. Palo Verde, Cerro Guayacan [;] 13.ix–13.x.1999, I. Jimenez, Malaise [;] L.N. 259350-389600 #53499; second label (red, partially printed and hand written) - HOLOTYPE [;] Heterospilus [;] diecinueve [;] P. Marsh. Deposited in ESUW.

#### Paratypes.

Known only from the holotype.

#### Comments.

The dark brown body, the smooth mesoscutum and the slightly sinuate anterior transverse groove of the metasoma are distinctive for this species.

#### Etymology.

The specific name is an arbitrary combination of letters.

**Figure 229.**
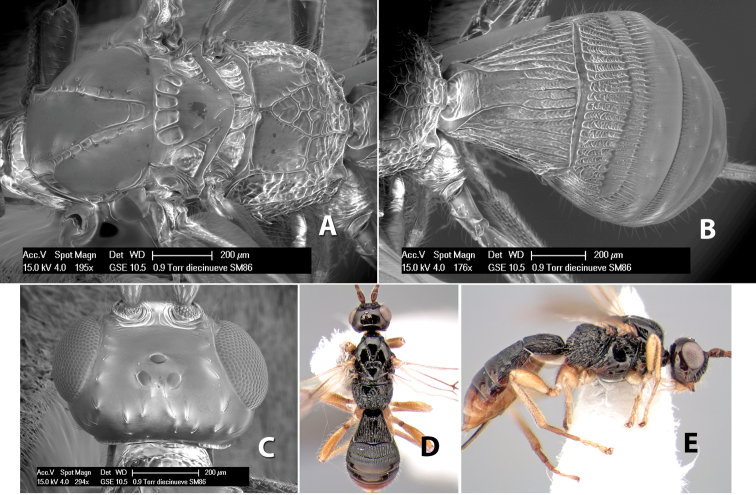
*Heterospilus diecinueve* Marsh, sp. n., holotype.

### Heterospilus dieciocho

Marsh sp. n.

http://zoobank.org/DEB6CC36-74FD-42A8-BFEE-8A45EA212429

http://species-id.net/wiki/Heterospilus_dieciocho

Figure 230

#### Female.

Body size: 3.5 mm. Color: head with vertex and frons brown, face, eye orbits and temple honey yellow; scape light brown without lateral brown stripe; flagellum dark brown; mesosoma dark brown; wing veins including stigma brown; legs yellow; metasomal terga 1, 2 and base of 3 dark brown, remainder light brown. Head: vertex smooth; frons mostly smooth, transversely striate near antennal bases; face smooth; temple in dorsal view narrow, width less than 1/2 eye width; malar space equal to 1/4 eye height; ocell-ocular distance about twice diameter of lateral ocellus; 26 flagellomeres. Mesosoma: mesoscutal lobes granulate; notauli scrobiculate, meeting posteriorly in triangular costate area; scutellum smooth; prescutellar furrow with 1 cross carina; mesopleuron smooth; precoxal sulcus smooth, shorter than mesopleuron; venter smooth; propodeum with basal median areas margined, weakly granulate or smooth, basal median carina present, areola distinctly margined, areolar area rugose, lateral areas rugose apically, smooth basally. Wings: fore wing vein r shorter than vein 3RSa, vein 1cu-a beyond vein 1M; hind wing vein SC+R present, vein M+CU as long as vein 1M. Metasoma: first tergum longitudinally costate, length equal to apical width; second tergum longitudinally costate; anterior transverse groove present straight or slightly sinuate laterally; posterior transverse groove present; third tergum costate at base, smooth at apex; terga 4–7 smooth; ovipositor as long as metasoma.

#### Holotype female.

Top label (white, partially printed and hand written) - COSTA RICA: Limon [;] 16km West Guapiles [;] 400m, April 1989 [;] P. Hanson IV-V; second label (red, partially printed and hand written) - HOLOTYPE [;] Heterospilus [;] dieciocho [;] P. Marsh. Deposited in ESUW.

#### Paratypes.

Known only from the holotype.

#### Comments.

The prescutellar furrow with one cross carina, granulate mesoscutum, brown flagellum and honey yellow face are distinctive for this species.

#### Etymology.

The specific name is an arbitrary combination of letters.

**Figure 230.**
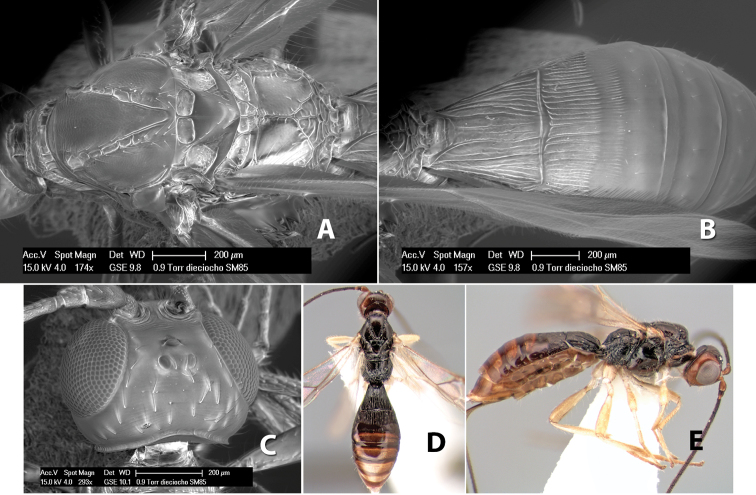
*Heterospilus dieciocho* Marsh, sp. n., holotype.

### Heterospilus dieciseis

Marsh sp. n.

http://zoobank.org/293F8E56-1D3C-4858-9270-934463280DA0

http://species-id.net/wiki/Heterospilus_dieciseis

Figure 231

#### Female.

Body size: 2.5 mm. Color: body brown, apical metasomal tergum yellow; scape yellow without lateral brown stripe; flagellum brown, apical 5–7 flagellomere sometimes dark; wing veins light brown, stigma light brown or yellow; legs yellow. Head: vertex smooth; frons smooth; face smooth; temple in dorsal view narrow, sloping behind eye, width less than 1/2 eye width; malar space greater than ⅓ eye height; ocell-ocular distance greater than 2.5 times diameter of lateral ocellus; 22 flagellomeres. Mesosoma: mesoscutal lobes weakly granulate or smooth; notauli weakly scrobiculate or pertly smooth, meeting posteriorly in triangular costate area; scutellum smooth; prescutellar furrow with 1 cross carina; mesopleuron smooth; precoxal sulcus smooth, shorter than mesopleuron; venter smooth; propodeum with basal median areas margined, smooth, basal median carina present, areola distinctly margined, areolar area rugose, lateral areas rugose apically, smooth basally. Wings: fore wing vein r shorter than vein 3RSa, vein 1cu-a slightly beyond vein 1M; hind wing vein SC+R present, vein M+CU shorter than vein 1M. Metasoma: first tergum longitudinally costate, length greater than twice apical width; second tergum smooth; anterior transverse groove weakly present, straight; posterior transverse groove weakly present; third tergum smooth entirely; terga 4–7 smooth; ovipositor about 3/4 length of metasoma.

#### Holotype female.

Top label (white, printed) - Costa Rica: Cartago [;] Turrialba, CATIE [;] 14–15.iii.1990 [;] 700m, J.S. Noyes; second label (red, partially printed and hand written) - HOLOTYPE [;] Heterospilus [;] dieciseis [;] P. Marsh. Deposited in ESUW.

#### Paratypes.

1 ♀, Costa Rica: San Jose [;] Braulio Carillo N. P. [;] 8.2km E tunnel [;] 15-V-1988 P. Hanson (TAMU).

#### Comments.

This species is similar to *Heterospilus catiensis* but is distinguished by the smooth metasomal terga 2–7 and the shorter ovipositor.

#### Etymology.

The specific name is an arbitrary combination of letters.

**Figure 231.**
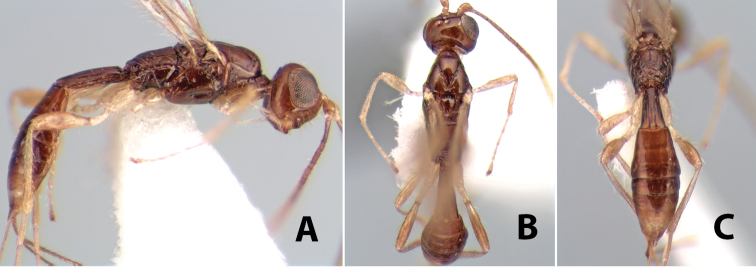
*Heterospilus dieciseis* Marsh, sp. n., holotype.

### Heterospilus diecisiete

Marsh sp. n.

http://zoobank.org/186F878E-0412-4E8F-8EC1-84AAEE29AFBD

http://species-id.net/wiki/Heterospilus_diecisiete

Figure 232

#### Female.

Body size: 2.0 mm. Color: head and mesosoma dark brown, metasoma brown; scape light brown without lateral brown stripe; flagellum brown; wing veins including stigma brown; legs bicolored, tibiae and tarsi yellow, femora and coxae light brown, trochanters yellow. Head: eyes small; vertex smooth; frons smooth; face smooth, width greater than height, oral opening unusually large; temple in dorsal view broad, width greater than 1/2 eye width; malar space greater than 1/4 eye height; ocell-ocular distance about 4 times diameter of lateral ocellus; 13 flagellomeres. Mesosoma: mesoscutal lobes smooth; notauli scrobiculate, meeting posteriorly in small triangular costate area; scutellum smooth; prescutellar furrow with 3 cross carinae; mesopleuron smooth; precoxal sulcus scrobiculate, as long as mesopleuron; venter smooth; propodeum with basal median areas margined, smooth, basal median carina present and long, areola not margined, areolar area rugose, lateral areas entirely rugose, propodeum with apical-lateral corners pointed. Wings: fore wing vein r shorter than vein 3RSa, vein 1cu-a slightly beyond vein 1M; hind wing vein SC+R present, vein M+CU shorter than vein 1M. Metasoma: first tergum longitudinally costate, length greater than apical width; second tergum smooth except for weak short costae at basal corners; anterior transverse groove weak but present; posterior transverse groove weak but present; third tergum smooth entirely; terga 4–7 smooth; ovipositor about as long as entire body.

#### Holotype female.

Top label (white, printed) - Costa Rica: Heredia [;] Est. Biol. La Selva [;] 50–150m, 10.26N, 84.01W [;] ii-iv 1993, P. Hanson [;] huertos Malaise trap [;] set by G. Wright; second label (red, partially printed and hand written) - HOLOTYPE [;] Heterospilus [;] diecisiete [;] P. Marsh. Deposited in ESUW

#### Paratypes.

Known only from the holotype.

#### Comments.

This unusual species is distinguished by the small eyes and short face, short flagellum, long precoxal sulcus, pointed apical-lateral corners of the propodeum and the unusually long ovipositor.

#### Etymology.

The specific name is an arbitrary combination of letters.

**Figure 232.**
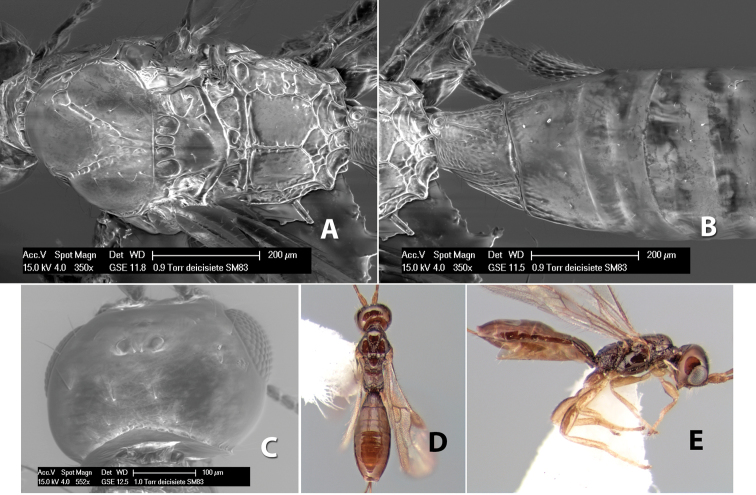
*Heterospilus diecisiete* Marsh, sp. n., holotype.

### Heterospilus diez

Marsh sp. n.

http://zoobank.org/E77EBBF3-06C6-40AD-97FC-C40D15191EA5

http://species-id.net/wiki/Heterospilus_diez

Figure 233

#### Female.

Body size: 2.0 mm. Color: head and mesosoma dark brown; metasomal terga 1, 3 and 4 dark brown, tergum 2 honey yellow medially, terga 5–7 yellow; scape light brown with lateral longitudinal darker brown stripe; flagellum brown with white apical annulus, apical 3–4 flagellomeres brown; wing veins brown, stigma bicolored brown with extreme base lighter; legs yellow. Head: vertex smooth; frons smooth; face smooth; temple in dorsal view broad but sloping behind eye, width about equal to 1/2 eye width; malar space greater than 1/4 eye height; ocell-ocular distance greater than 2.5 times diameter of lateral ocellus; 20 flagellomeres. Mesosoma: mesoscutal lobes weakly granulate; notauli scrobiculate, meeting posteriorly is small triangular costate area; scutellum granulate; prescutellar furrow with 3–5 cross carinae; mesopleuron granulate; precoxal sulcus weakly scrobiculate or smooth, shorter than mesopleuron; venter granulate; propodeum with basal median areas margined, granulate, basal median carina present but short, areola not margined, areolar area rugose, lateral areas rugose apically, granulate basally. Wings: fore wing vein r shorter than vein 3RSa, vein 1cu-a beyond vein 1M; hind wing vein SC+R present, vein M+CU shorter than vein 1M. Metasoma: first tergum longitudinally costate, length greater than apical width; second tergum longitudinally costate; anterior transverse groove present, straight; posterior transverse groove present; third tergum costate basally, smooth apically; terga 4–7 smooth; ovipositor as long as metasomal terga 1 and 2 combined.

#### Holotype female.

Top label (white, partially printed and hand written) - Costa Rica: Guanacaste [;] Santa Rosa Natl. Park [;] 300m, ex. Malaise trap [;] Site #: H-2-C [;] Dates: 8–29.xi.1986 [;] I.D. Gauld & D. Janzen; second label (white, printed) - [H] open regenerating [;] woodland <10 years old [;] [C] more or less fully [;] shaded as possible; third label (red, partially printed and hand written) - HOLOTYPE [;] Heterospilus [;] diez [;] P. Marsh. Deposited in ESUW.

#### Paratypes.

Known only from the holotype.

#### Comments.

The white annulus on the flagellum, the weak but visible lateral brown stripe on the scape and the bicolored metasomal terga are distinctive for this species.

#### Etymology.

The specific name is an arbitrary combination of letters.

**Figure 233.**
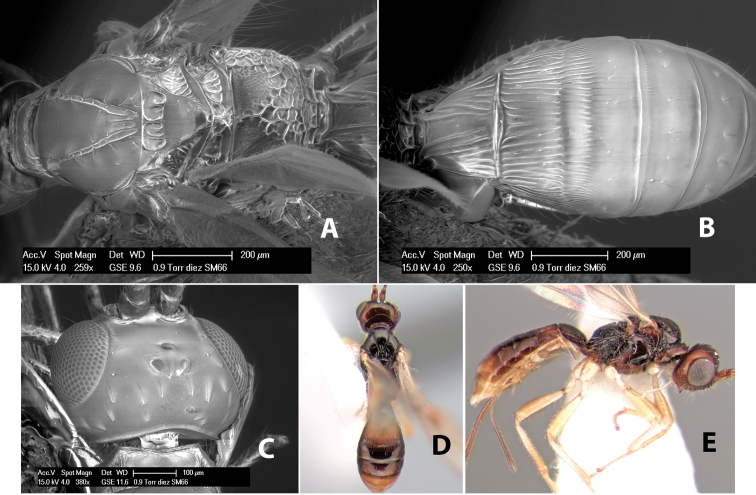
*Heterospilus diez* Marsh, sp. n., holotype.

### Heterospilus doce

Marsh sp. n.

http://zoobank.org/B8E4DD6C-3AC9-4B55-A403-3F56D1A1B2F0

http://species-id.net/wiki/Heterospilus_doce

Figure 234

#### Female.

Body size: 3.0 mm. Color: body dark brown, apical metasomal terga yellow; scape brown without lateral brown stripe; flagellum brown; wing veins brown, stigma yellow; legs yellow. Head: vertex smooth; frons smooth; face smooth; temple in dorsal view broad but sloping behind eye, width equal to 1/2 eye width; malar space greater than 1/4 eye height; ocell-ocular distance greater than 2.5 times diameter of lateral ocellus; 22–24 flagellomeres. Mesosoma: mesoscutal lobes smooth; notauli scrobiculate, bordered by 2 strong carinae at their posterior junction; scutellum smooth; prescutellar furrow with 3 cross carinae; mesopleuron smooth; precoxal sulcus weakly scrobiculate or smooth, shorter than mesopleuron; venter smooth; propodeum with basal median areas indistinctly margined, rugose, basal median carina absent, areola not margined, areolar area areolate-rugose, lateral areas entirely rugose. Wings: fore wing vein r nearly as long as vein 3RSa, vein 1cu-a beyond vein 1M; hind wing vein SC+R present, vein M+CU nearly as long as vein 1M. Metasoma: first tergum longitudinally costate or porcate, length equal to apical width; second tergum longitudinally costate; anterior transverse groove present, straight; posterior transverse groove absent; third tergum costate at base, smooth at apex; terga 4–7 smooth; ovipositor as long as metasomal 1 and 2 combined.

#### Holotype female.

Top label (white, printed) - Costa Rica: San Jose [;] San Antonio de Escazu [;] 1300m, iii-iv.1998 [;] W.Eberhard & P.Hanson; second label (red, partially printed and hand written) - HOLOTYPE [;] Heterospilus [;] doce [;] P. Marsh. Deposited in ESUW.

#### Paratypes.

1 ♀, Costa Rica: Guanacaste [;] Santa Rosa Natl. Park [;] 300m, ex. Malaise trap [;] Site #: 11 [;] Dates: 13.iv–4.v.1986 [;] I.D. Gauld & D. Janzen (ESUW). 2 ♀♀, top label - Costa Rica: Guanacaste [;] Santa Rosa Natl. Park [;] 300m, ex. Malaise trap [;] Site #; blank [;] Dates: 4–14.v.1986 and 8–24.xi.1986 [;] I.D. Gauld & D. Janzen; second label - [SE] Bosque San Emilio [;] 50yr old deciduous forest [;] [C] more or less fully [;] shaded as possible (ESUW). 1 ♀, top label - Costa Rica: Guanacaste [;] Santa Rosa Natl. Park [;] 300m, ex. Malaise trap [;] Site #; 4 [;] Dates 18.x–8.xi.1986 [;] I.D. Gauld & D. Janzen; [H] open regenerating [;] woodland <10 years old [;] [O] in clearing, fully [;] isolated part of day (ESUW). 1 ♀, Costa Rica: Guanacaste [;] Est. Biol. Maritza, 600m [;] i.1997, C. Zuniga, Malaise [;] L.N. 326900-373000 #47557 (ESUW). 1 ♀, Costa Rica: Puntarenas [;] San Vito - Las Cruces [;] 5-VI-1988 1200m [;] P. Hanson (TAMU). 2 ♀♀, S.RosaPark, Guan. [;] C. Rica 28 Oct 77 and 6 Dec 77 [;] D.H. Janzen [;] Dry Hill (AEIC).

#### Comments.

The distinct carinae along the notauli where they meet posteriorly, the yellow stigma and rugose propodeum are distinctive for this species.

#### Etymology.

The specific name is an arbitrary combination of letters.

**Figure 234.**
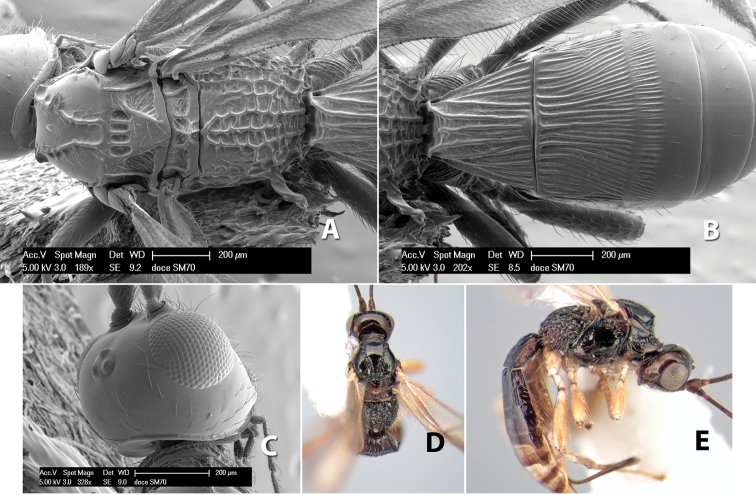
*Heterospilus doce* Marsh, sp. n.: **A–C** paratype **D–E** holotype.

### Heterospilus dos

Marsh sp. n.

http://zoobank.org/01DE5AB4-7989-41E9-BC1C-4F664EE804DB

http://species-id.net/wiki/Heterospilus_dos

Figure 235

#### Female.

Body size: 3.5–4.0 mm. Color: head with vertex dark brown, face and eye orbits honey yellow; scape yellow without lateral brown stripe; flagellum brown; mesosoma dark brown; metasoma dark brown, apical terga lighter brown; wing veins including stigma brown; legs yellow. Head: vertex smooth; frons smooth; face rugose; temple in dorsal view narrow, sloping behind eye, width less than 1/2 eye width; malar space about equal to 1/4 eye height; ocell-ocular distance 1.5 times diameter of lateral ocellus; 29–31 flagellomeres. Mesosoma: mesoscutal lobes granulate; notauli scrobiculate, meeting posteriorly in rectangular rugose area; scutellum smooth; prescutellar furrow with 3–5 cross carinae; mesopleuron weakly granulate, rarely smooth above precoxal sulcus; precoxal sulcus weakly scrobiculate, shorter than mesopleuron; venter weakly granulate or partially smooth; propodeum with basal median areas margined, granulate, basal median carina absent, areola not margined, areolar area areolate-rugose, lateral areas rugose posteriorly, smooth anteriorly. Wings: fore wing vein r slightly shorter or equal to vein 3RSa, vein 1cu-a beyond vein 1M; hind wing vein SC+R present, vein M+CU shorter than vein 1M. Metasoma: first tergum longitudinally costate-rugose, length equal to or slightly greater than apical width; second tergum longitudinally costate-granulate; anterior transverse groove present, straight; posterior transverse groove present; third tergum costate basally, smooth apically; terga 4–7 smooth; ovipositor longer than metasoma.

#### Holotype female.

Top label (white, printed) - Costa Rica: Heredia [;] Est. Biol. La Selva [;] 50–150m, 10.26N, 84.01W [;] ii-iv 1993, P. Hanson [;] huertos Malaise trap [;] set by G. Wright; second label (red, partially printed and hand written) - HOLOTYPE [;] Heterospilus [;] dos [;] P. Marsh. Deposited in ESUW.

#### Paratypes.

2 ♀♀, COSTA RICA, Limon [;] 4km NE Bribri [;] 50m, IX-XI 1989 [;] col. Paul Hanson (ESUW).

#### Comments.

The long ovipositor, rugose area where notauli meet on mesoscutum and the large ocelli and short ocell-ocular distance are distinctive for this species.

#### Etymology.

The specific name is an arbitrary combination of letters.

**Figure 235.**
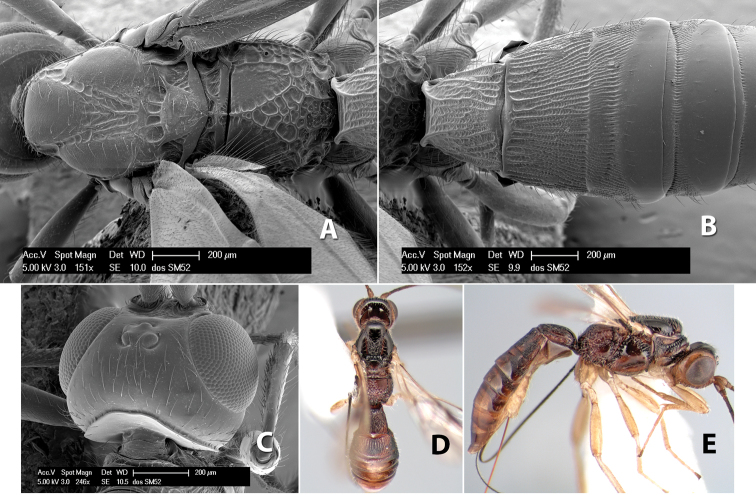
*Heterospilus dos* Marsh, sp. n.: **A–C** paratype **D–E** holotype.

### Heterospilus dulcus

Marsh sp. n.

http://zoobank.org/AE368D52-FA6F-48BB-A701-59B727AC3448

http://species-id.net/wiki/Heterospilus_dulcus

Figure 236

#### Female.

Body size: 3.5 mm. Color: body dark brown, apical metasomal terga somewhat lighter brown; scape brown; flagellum brown with white apical annulus, apical 3–5 flagellomeres brown; wing veins including stigma brown; legs yellow. Head: vertex smooth; frons smooth; face smooth; temple in dorsal view narrow, sloping behind eye, width less than 1/2 eye height; malar space equal to 1/4 eye height; ocell-ocular distance slightly greater than twice diameter of lateral ocellus; 26–29 flagellomeres. Mesosoma: mesoscutal lobes smooth; notauli weakly scrobiculate or smooth, meeting posteriorly in triangular costate area; scutellum smooth; prescutellar furrow with 1 cross carina; mesopleuron smooth; precoxal sulcus smooth, shorter than mesopleuron; venter smooth; propodeum with basal median areas margined, smooth, basal median carina present, areola distinctly margined, areolar area rugose, lateral areas rugose apically, smooth basally. Wings: fore wing vein r shorter than vein 3RSa, vein 1cu-a beyond vein 1M; hind wing vein SC+R present, vein M+CU as long as vein 1M. Metasoma: first tergum longitudinally costate, length slightly greater than apical width; second tergum longitudinally costate, width nearly 4 times length; anterior transverse groove present, straight; posterior transverse groove present; third tergum costate basally, smooth apically; terga 4–7 smooth; ovipositor half as long as metasoma.

#### Holotype female.

Top label (white, printed) - Costa Rica: Puntarenas [;] Golfo Dulce, 24km W. [;] Piedras Blancas, 200m [;] ii.1993, Paul Hanson; second label (red, partially printed and hand written) - HOLOTYPE [;] Heterospilus [;] dulcus [;] P. Marsh. Deposited in ESUW.

#### Paratypes.

1 ♀, Costa Rica: Puntarenas [;] R.F. Golfo Dulce, 24km. [;] W. Piedras Blancas, 200m [;] VIII-IX-1993, P. Hanson (ESUW). 1 ♀, Costa Rica: Puntarenas, ACO [;] Golfito, Est. Agujas, 250–350m [;] Res. Ftal. Golfo Dulce, Amarilla [;] 3–24.vii.1999, J. Azofeifa [;] L.S. 276750-526550 #52839 (ESUW). 1 ♀, Costa Rica: Puntarenas [;] R.F. Golfo Dulce, [;] 3km. SW. Rincon, 10m [;] Oct. 1991, Paul Hanson (ESUW). 1 ♀, Costa Rica: Cartago [;] Turrialba, CATIE [;] 14–15 March 1990 [;] 700m, J.S. Noyes (ESUW). 2 ♀♀, Sirena, Osa Pen. [;] VII. 77 Cos. Rica [;] D. H. Janzen (AEIC). 1 ♀, C. Rica:Escazú [;] May 20, 1987 [;] H.&M.Townes (AEIC).

#### Comments.

The single cross carina in the prescutellar furrow, the flagellum with the apical white annulus and the shorter ovipositor are distinctive for this species.

#### Etymology.

The specific name is from the Latin *dulcis*, meaning sweet, in reference to most of the type series being from the area of Golfo Dulce, the Sweet Gulf.

**Figure 236.**
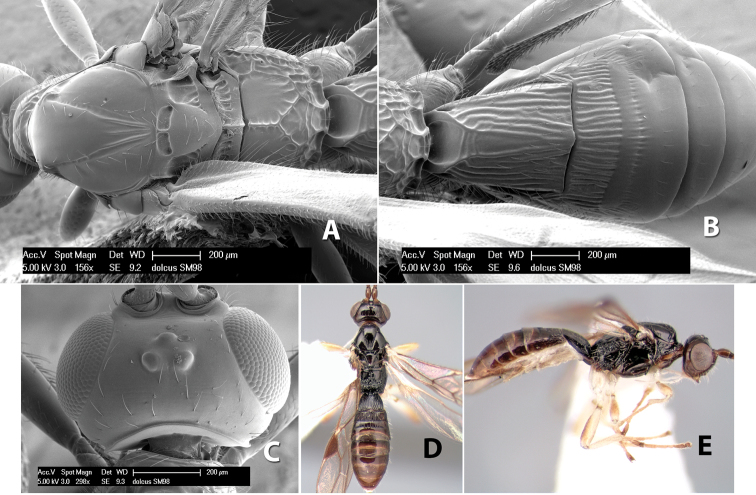
*Heterospilus dulcus* Marsh, sp. n.: **A–C** paratype **D–E** holotype.

### Heterospilus empalmensis

Marsh sp. n.

http://zoobank.org/7FCD4A18-2A6B-4F86-AA42-AB8697A28A63

http://species-id.net/wiki/Heterospilus_empalmensis

Figure 237

#### Female.

Body size: 3.0 mm. Color: head brown; scape honey yellow; flagellum brown, basal flagellomeres often honey yellow; mesosoma with lateral mesoscutal lobes honey yellow, medial lobes brown, pronotum, mesopleuron, and propodeum dark brown, lower portion of mesopleuron along precoxal sulcus often honey yellow; wing veins brown, stigma yellow; legs honey yellow; metasomal terga 1 and 2 brown, remainder of terga honey yellow. Head: vertex smooth; frons smooth; face smooth; temple in dorsal view broad, slightly bulging behind eye, width greater than 1/2 eye width; malar space greater than 1/4 eye height, nearly ⅔ eye height; ocell-ocular distance nearly 5 times diameter of lateral ocellus; 19–22 flagellomeres. Mesosoma: mesoscutal lobes weakly granulate or smooth; notauli scrobiculate, meeting posteriorly in triangular costate area; scutellum smooth; prescutellar furrow with 1 cross carina; mesopleuron smooth; precoxal sulcus weakly scrobiculate or smooth, shorter than mesopleuron; venter smooth; propodeum with basal median areas margined, weakly granulate or smooth, basal median carina present, areola not margined, areolar area rugose, lateral areas entirely rugose. Wings: fore wing vein r shorter than vein 3RSa and nearly as long as stigma width, vein 1cu-a beyond vein 1M; hind wing vein SC+R present, straight, vein M+CU equal to vein 1M. Metasoma: first tergum longitudinally costate, length greater than apical width; second tergum longitudinally costate; anterior transverse groove weak or absent; posterior transverse groove weak or absent; third tergum smooth entirely; terga 4–7 smooth; ovipositor equal to half length of metasoma.

#### Holotype female.

Top label (white, printed) - Costa Rica: San Jose [;] Cerro de la Muerte [;] 20Km. S. Empalme [;] 2800m, vii-viii 1989 [;] Paul Hanson; second label (red, partially printed and hand written) - HOLOTYPE [;] Heterospilus [;] empalmensis [;] P. Marsh. Deposited in ESUW.

#### Paratypes.

2 ♀♀, same data as holotype (ESUW). 2 ♀♀, Costa Rica: San Jose [;] 16Km S. Empalme [;] 2600m, i-ii 1989 [;] P. Hanson & I. Gauld [sic] (ESUW). 5 ♀♀, Costa Rica: San Jose [;] Cerro de la Muerte [;] 19km. S. 3km. W. Empalme [;] 2600m, IV-VII.1992 [;] P. Hanson (ESUW). 2 ♀♀, COSTA RICA: San Jose, Cerro Muerte [;] 20km S Empalme, 2800m [;] xi 88-I 1989, P. Hanson (ESUW). 2 ♀♀, Costa Rica: San Jose [;] Cerro de la Muerte, 19km [;] S3km W Empalme, 2600m [;] ix.1992 and x-xii.1993, P. Hanson, Malaise (ESUW). 1 ♀, Costa Rica: San Jose [;] Cerro de la Muerte, 2800m [;] 6km. N. San Gerrado [;] vi.1992, P Hanson coll. (ESUW). 1 ♀, COSTA RICA: San Jose [;] P.N. Braulio Carillo [;] 9.5km E tunnel, 1000m [;] vii-ix 1989, P. Hanson (ESUW). 1 ♀, top label - Costa Rica: Guanacaste [;] Santa Rosa Natl. Park [;] 300m, ex. Malaise trap [;] Site #: H-1-O [;] Dates: 20.xii.86–10.i.1987 [;] I.D. Gauld & D. Janzen; second label - [H] open regenerating [;] woodland <10 years old [;] [O] in clearing, fully [;] isolated part of day (ESUW). 1 ♀, Costa Rica: Puntarenas, ACLAC [;] PILA, Est. Altamira, 2279m [;] Cerro Quemado, R. Villalobos [;] 23.iv–22.vi.1999, Malaise trap [;] L.S. 336200-575560 #52790 (ESUW). 1 ♀, Costa Rica: Cartago [;] 4km NE Cañon [;] Genesis II, 2350m [;] x.1996, P. Hanson (ESUW). 1 ♀, COSTA RICA: Cartago [;] Cerro de la Muerte [;] Villa Mills, 3000m [;] vii-viii 1989, Hanson (ESUW). 3 ♀♀, COSTA RICA, SanJosé [;] Cerro Muerte, 20Km S [;] Empalme, 2800m [;] I-II/1989, P. Hanson (MICR). 6 ♀♀, COSTA RICA, Cartago- [;] San Jose, 20km SE [;] Empalme, 2800m [;] VIII to XI/ 1988, Col. Hanson (MICR).

#### Comments.

The long malar space, bicolored mesosoma, single cross carina in the prescutellar furrow and the narrow stigma are distinctive for this species.

#### Etymology.

The specific name is from the locality of Empalme near where many of the type series were collected.

**Figure 237.**
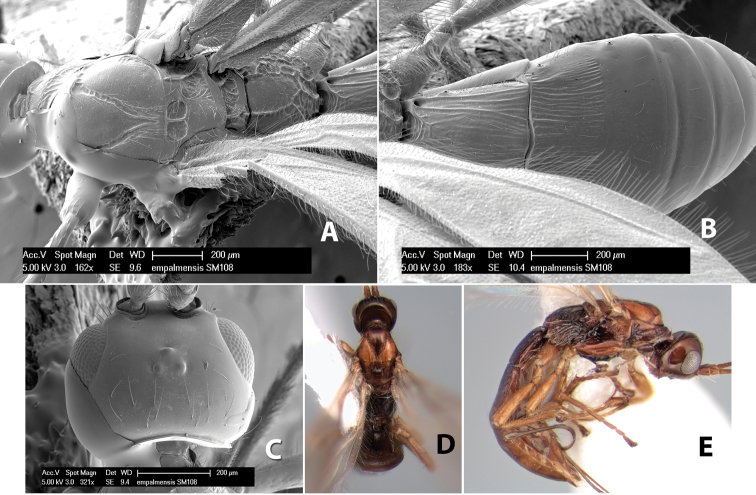
*Heterospilus empalmensis* Marsh, sp. n.: **A–C** paratype **D–E** holotype.

### Heterospilus flavostigmus

Marsh sp. n.

http://zoobank.org/D5207705-FEF4-42CA-B28B-C2DB218B22AF

http://species-id.net/wiki/Heterospilus_flavostigmus

Figure 238

#### Female.

Body size: 3.0 mm. Color: head and mesosoma dark brown or black, metasoma honey yellow or light brown, tergum 1 often darker; scape yellow without lateral brown stripe, flagellum brown, basal flagellomere often yellow; wing veins brown, stigma yellow; legs yellow. Head: vertex smooth; frons smooth; face smooth; temple in dorsal view broad, somewhat bulging behind eye, width equal to 1/2 eye width; malar space greater than 1/4 eye height; ocell-ocular distance greater than 2.5 times diameter of lateral ocellus; 24 flagellomeres. Mesosoma: mesoscutal lobes granulate; notauli scrobiculate, meeting at scutellum in triangular costate area; scutellum smooth; prescutellar furrow with 3 cross carinae; mesopleuron smooth above precoxal sulcus, weakly granulate dorsally; precoxal sulcus weakly scrobiculate, shorter than mesopleuron; venter smooth; propodeum with basal median areas margined, granulate, basal median carina present, areola somewhat distinctly margined, areolar area rugose, lateral areas entirely rugose. Wings: fore wing vein r shorter than vein 3RSa, vein 1cu-a slightly beyond vein 1M; hind wing vein SC+R absent, vein M+CU shorter than vein 1M. Metasoma: first tergum longitudinally costate, length slightly greater than apical width; second tergum longitudinally costate-rugose; anterior transverse groove present, straight; posterior transverse groove present; third tergum costate basally, smooth or weakly granulate apically; terga 4–7 smooth or weakly granulate; ovipositor half as long as metasoma.

#### Holotype female.

Top label (white, partially printed and hand written) - Costa Rica: Guanacaste [;] Santa Rosa Natl. Park [;] 300m, ex. Malaise trap [;] Site #: SE-7-O [;] Dates: 8–29.xi.1986 [;] I.D. Gauld & D. Janzen; second label (white, printed) - [SE] Bosque San Emilio [;] 50yr old deciduous forest [;] [O] in clearing, fully [;] isolated part of day; third label (red, partially printed and hand written) - HOLOTYPE [;] Heterospilus [;] flavostigmus [;] P. Marsh. Deposited in ESUW.

#### Paratypes.

1 ♀, same data as holotype with date of 14.vii–6.ix.1986 (ESUW). 1 ♀, Costa Rica: Alajuela, ACA [;] San Carlos, R.F. Arenal, 600m [;] Sec. la Penisula, Amarilla [;] 26–29.xi.1999, G. Carballo [;] L.N. 271500-453800 #54375 (ESUW).

#### Comments.

The yellow stigma and light brown or honey yellow metasoma are distinctive for this species.

#### Etymology.

The specific name is from the Latin *flavus*, meaning yellow, in reference to the yellow stigma.

**Figure 238.**
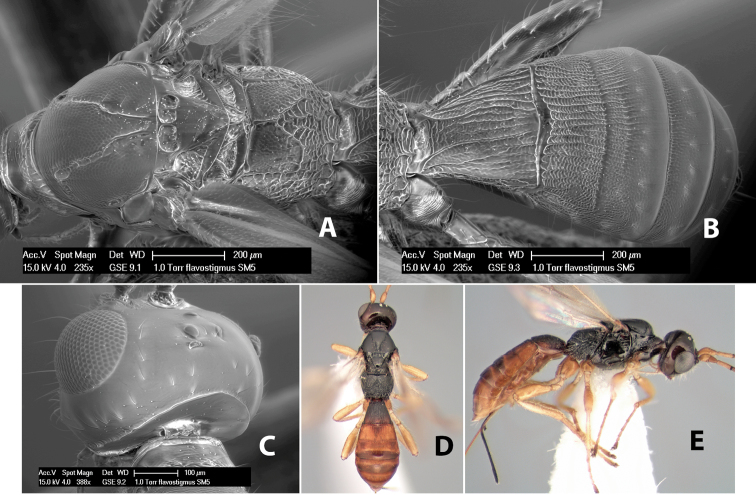
*Heterospilus flavostigmus* Marsh, sp. n., holotype.

### Heterospilus hachaensis

Marsh sp. n.

http://zoobank.org/01275539-377D-440F-98C9-71DB4DFB88C3

http://species-id.net/wiki/Heterospilus_hachaensis

Figure 239

#### Female.

Body size: 3.5–4.0 mm. Color: body dark brown or black; scape yellow without lateral brown stripe; flagellum brown, basal flagellomeres often lighter; wing veins brown, stigma yellow; legs honey yellow. Head: vertex smooth; frons weakly transversely striate; face weakly striate or smooth; temple in dorsal view broad, somewhat bulging behind eye, width equal to 1/2 eye width; malar space greater than 1/4 eye height; ocell-ocular distance slightly greater than 2.5 times diameter of lateral ocellus; 27–34 flagellomeres. Mesosoma: mesoscutal lobes weakly granulate or partially smooth; notauli scrobiculate, meeting posteriorly in triangular rugose area; scutellum smooth; prescutellar furrow with 3–5 cross carinae; mesopleuron granulate; precoxal sulcus weakly scrobiculate, shorter than mesopleuron; venter weakly granulate or partially smooth; propodeum with basal median areas margined, granulate, basal median carina absent, areola not margined, areolar area rugose, lateral areas entirely rugose. Wings: fore wing vein r shorter than vein 3RSa, vein 1cu-a beyond vein 1M; hind wing vein SC+R present, vein M+CU shorter than vein 1M. Metasoma: first tergum longitudinally costate, length equal to apical width; second tergum longitudinally costate; anterior transverse groove present, straight; posterior transverse groove present; third tergum nearly completely costate, smooth at extreme posterior border; terga 4–5 costate at base, smooth at apex, tergum 6 granulate at base, smooth at apex; ovipositor as long as metasomal tergum 1.

#### Holotype female.

Top label (white, printed) - COSTA RICA: [;] Guanacaste Prov. [;] Cerro el Hacha [;] NW Volcan Orosi [;] 300m, 1988; second label (red, partially printed and hand written) - HOLOTYPE [;] Heterospilus [;] hachaensis [;] P. Marsh. Deposited in ESUW.

#### Paratypes.

5 ♀♀, same data as holotype (ESUW).

#### Comments.

The yellow stigma, costate metasomal terga 4–5 and dark brown or black body are distinctive for this species.

#### Etymology.

Named for the type locality of Cerro el Hacha in Guanacaste Province.

**Figure 239.**
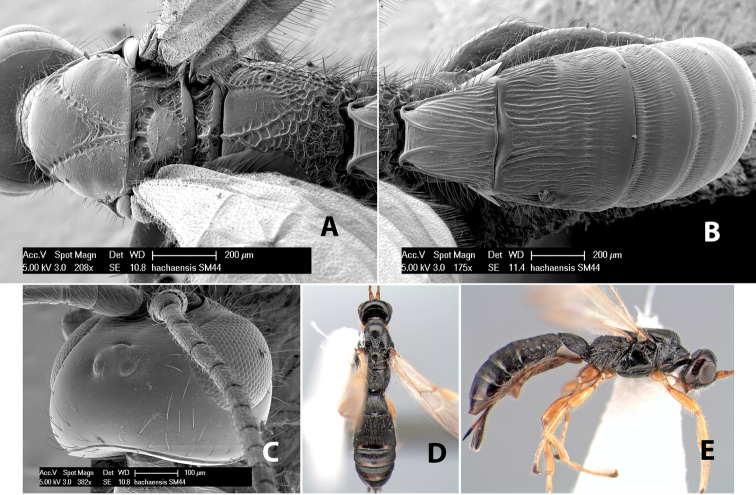
*Heterospilus hachaensis* Marsh, sp. n.: **A–C** paratype **D–E** holotype.

### Heterospilus hansonorum

Marsh sp. n.

http://zoobank.org/DF438B3F-E91B-45AA-A546-4D23709FE8AB

http://species-id.net/wiki/Heterospilus_hansonorum

Figure 240

#### Female.

Body size: 2.5–3.0 mm. Color: body dark brown, metasomal terga 3–5 with basal weakly sclerotized area often lighter colored; scape brown without lateral brown stripe; flagellum brown with apical 5–7 flagellomeres white, apical flagellomere often darker; wing veins including stigma brown; legs yellow or light brown. Head: vertex smooth; frons smooth; face smooth; temple in dorsal view narrow, sloping behind eye, width less than 1/2 eye width; malar space slightly greater than 1/4 eye height; ocell-ocular distance slightly greater than 2.5 times diameter of lateral ocellus; 17–23 flagellomeres. Mesosoma: mesoscutal lobes smooth; notauli smooth, meeting posteriorly in triangular rugose area; scutellum smooth; prescutellar furrow usually with 1 cross carina, rarely with weaker carinae on each side; mesopleuron smooth; precoxal sulcus smooth, shorter than mesopleuron; venter smooth; propodeum with basal median areas margined, smooth, basal median carina present, often very short, areola distinctly margined, areolar area rugose, lateral areas nearly entirely smooth, some weakly rugose at extreme apex. Wings: fore wing vein r shorter than vein 3RSa, vein 1cu-a slightly beyond or nearly interstitial with vein 1M; hind wing vein SC+R present, vein M+CU shorter than vein 1M. Metasoma: first tergum longitudinally costate-granulate, length greater than apical width, median raised area margined by distinct scrobiculate grooves; second tergum costate, width nearly 4 times length; anterior transverse groove present, straight or slightly sinuate; posterior transverse groove weak or absent; terga 3–5 smooth and with basal areas weakly sclerotized and usually white; terga 6–7 smooth; ovipositor 1/2 to 3/4 length of metasoma.

#### Holotype female.

Top label (white, printed) Costa Rica, Carthago Pr. [;] La Cangreja, 1960m [;] 1991: xi, P. Hanson; second label (red, partially printed and hand written) - HOLOTYPE [;] Heterospilus [;] hansonorum [;] P. Marsh. Deposited in ESUW.

#### Paratypes.

2 ♀♀, same data as holotype with additional dates of xii.1991 and vi-vii.1992 (ESUW). 1 ♀, Costa Rica, Carthago Pr. [;] Dulce Nombre, Vivero [;] Linda Vista, 1300m [;] 1993: viii-x, P. Hanson (ESUW). 3 ♀♀, Costa Rica: Cartago [;] Turrialba, CATIE [;] 14–15 March 1990 [;] 700m, J.S. Noyes (ESUW). 1 ♀, Costa Rica: Cartago [;] 4km NE Cañon [;] Genesis II, 2350m [;] v.1995, P. Hanson (ESUW). 1 ♀, Costa Rica: Cartago [;] Braulio Carillo N.P. [;] 600m, 25.iii.1990 [;] J. S. Boyes, coll. (ESUW). 1 ♀, COSTA RICA: Puntar [;] Golfo Dulce 3km SW [;] Rincon [;] 10m, xii 1989-iii 1990 [;] Col. Paul Hanson (ESUW). 1 ♀, Costa Rica: Puntarenas [;] Buenos Aires, Est. Altamira [;] Send. Los Gigante, 1450m [;] 4.i–3.ii.2000, D.Rubi, Malaise [;] L.S. 331700-572200 #54810 (ESUW). 2 ♀♀, COSTA RICA: Puntar [;] R.B. Carara, Estac. [;] Bijagoal, 500m [;] X 1989, P. Hanson (ESUW). 2 ♀♀, Costa Rica: Puntarenas, ACO [;] Golfito, PN Corcovado [;] Est. Agujas, Los Charcos [;] 600–745m, 15.vi–15.vii.1999 [;] Malaise, J. Azofeifa #52836 [;] L.S. 275500-523500 (ESUW). 1 ♀, Costa Rica: Puntarenas [;] Pen. Osa, Cerro Rincon [;] 200 meters S. del hito [;] 745m el., virgin forest [;] i.1991, Hanson & Quiros [;] ex. Malaise trap (ESUW). 1 ♀, COSTA RICA, Puntarenas [;] San Vito, Jardin Bot. [;] Las Cruces, VII-VIII/88 [l] 1200m, Col. P. Hanson (ESUW). 1 ♀, Costa Rica, Puntarenas [;] Pe. Osa, 5km. N. [;] Puerto Jimenez, 10m [;] I-II-1993 P. Hanson (ESUW). 9 ♀♀, Costa Rica: Puntarenas [;] Golfo Dulce, 24km W. [;] Piedras Blamcas, 200m [;] vi.1991, xii.1991, I.1993, VI-VII-1993, xi-xii.1992, ix-xi 1989 and III.1993, Paul Hanson (ESUW). 2 ♀♀, Costa Rica: Puntarenas [;] R.(eserva) F.(orestal) Golfo Dulce [;] 3km. SW Rincon, 10m [;] iii.1993 and July 1991, Paul Hanson coll. [;] Malaise, primary forest (ESUW). 2 ♀♀, Costa Rica: Puntarenas [;] R.F. Golfo Dulce [;] 3km SW. Rincon, 10m [;] vi.1991, Paul Hanson (ESUW). 3 ♀♀, COSTA RICA: Puntarenas [;] Rd. to Rincon, 24km W. [;] Pan-Amer. Hwy, 200m [;] III-V 1989, Hanson & Gauld (ESUW). 7 ♀♀, Costa Rica: Puntarenas [;] San Vito, Estac. Biol. [;] Las Alturas, 1500m [;] xii.1991, ii.1992, 15–31 Oct. 1991, ii-iv.1993 and iii-v.1995, Paul Hanson (ESUW). 1 ♀, Costa Rica: Puntarenas [;] Send. ac. Pittier, 1800-2000m [;] 1Km N. de la Est., Malaise [;] 13.ix–13.x.1996, A. M. Maroto [;] L.S. 331800-577400 #44868 (ESUW). 1 ♀, Costa Rica: Puntarenas [;] Zona Protectora Las Tablas [;] 1Km NE de Sitio Portones [;] Camino a Tablas, 1530m [;] 30.viii–5.ix.1995, M. Cinchilla [;] L.S. 320100-596800 #7458 [;] Malaise trap (ESUW). 1 ♀, Costa Rica: Puntarenas [;] Golfito, R.F. Golfo Dulce [;] Est. Agujas, 250–350m [;] 4–22.v.1999, J. Azofeifa [;] L.S. 276750-526550 #52779 [;] Red de Golpe (ESUW). 2 ♀♀, Costa Rica: Puntarenas [;] San Vito, Las Cruces [;] Wilson Botanical Gardens [;] 18–22.iii.1990, 1150m [;] J.S. Noyes (ESUW). 1 ♀, COSTA RICA, Puntarenas [;] Parrita, palmar [;] 0m, I 1989 [;] Col. P. Hanson (ESUW). 2 ♀♀, Costa Rica: Alajuela, ACA [;] San Carlos, R.F. Arenal [;] Sendero Pilon, 600m, Malaise [;] 14.x–3.xii.1998 and 17–18.v.1999, G. Carballo [;] L.N. 269100-457900 #53365 (ESUW). 4 ♀♀, Costa Rica: Alajuela [;] 5km. W San Ramon [;] 1200m, December 1996, November 1996 and I-1997 [;] O. Castro & P. Hanson (ESUW). 1 ♀, Costa Rica: Alajuela, San Carlos [;] R.F. Arenal, Sector de la Peninsula [;] 600m, 6–12.i.2000, G. Carballo [;] L.N. 271500-453800 #54382 [;] Amarilla (ESUW). 1 ♀, COSTA RICA: Limon [;] 4km NE Bribri [;] 50m, IX-XI 1989 [;] col. Paul Hanson (ESUW). 3 ♀♀, COSTA RICA: Limon [;] 16km West Guapiles [;] 400m, April 1989, III 1989 and II/1989 [;] P. Hanson (ESUW). 1 ♀, COSTA RICA, Limon [;] sur de Iriquois [;] 300m, 23/V/1987 [;] Col. Paul Hanson (ESUW). 1 ♀, COSTA RICA, Limon [;] Parque Nac. Cahuita [;] 0m, 29/IV/1988 [;] Col. Paul Hanson (ESUW). 1 ♀, Costa Rica: Limon, Central [;] R.B. Hitoy Cerere, Est. Hitoy [;] Cerere, Send. Toma de Agua, [;] 100–140m, Malaise trap [;] 11.x–11.xi.1999, F. Umana [;] L.N. 184600-643400 #54013 (ESUW). 2 ♀♀, Costa Rica: San Jose [;] 26km. N. San Isidro [;] just S. of Division [;] 2100m, vi-viii.1992 and xi.1992-i.1993 [;] P. Hanson, Malaise [;] secondary growth (ESUW). 5 ♀♀, COSTA RICA: San Jose [;] Zurqui de Moravia [;] 1600m, I-II 1989, 24.XII/1988, IV/1989 and III-1996 [;] P. Hanson & I. Gauld (ESUW). 1 ♀, COSTA RICA, San Jose [;] 6 NE San Jeronimo de Moravia, Carr. Carillo [;] 1500m, 12/V/88, Hanson (ESUW). 4 ♀♀, COSTA RICA: [;] San Jose [;] Ciudad Colon, 800m [;] xii 1989 - i 1990 [;] Luis Fournier (ESUW). 1 ♀, Costa Rica: San Jose [;] San Antonio de Escazu [;] 1300m, ix.1997 [;] W. Eberhard (ESUW). 1 ♀, COSTA RICA, Hered[ia] [;] Chilamate, 75m [;] 25.III.1989 [;] Hanson & Godoy (ESUW). 4 ♀♀, COSTA RICA-Heredia Prov. [;] La Selva Biological Station [;] 10°26'N, 84°01'W, 100m [;] Malaise trap 08, #366, 11, #408, 01, #305 and 05, #352 [;] 1.iii.1994, 17.vii.1995, 3.i.1994 and 15.ii.1994 [;] Project ALAS (ESUW). 2 ♀♀, Costa Rica: Heredia, ACCVC [;] Sarapiqui, Zona Prot. La Selva [;] Est. Biol. La Selva, Finca La Selva [;] 3km. S. de Sarapiqui, 50–100m [;] 18.iv.1988m, H. A. Hespenheide [;] manual (red, libre), #54835 [;] L.N. 268800-535300 (ESUW). 4 ♀♀, top label - Costa Rica: Guanacaste [;] Santa Rosa Natl. Park [;] 300m, ex. Malaise trap [;] Site #: BH-9-O and blank [;] Dates: 26.VII-14.VIII.1986, 18.x–8.xi.1986 and 20.xi.86–10.i.1987 [;] I.D. Gauld & D. Janzen; second label - [BH] Bosque Humedo [;] mature evergreen dry forest [;] [O] in clearing, fully [;] isolated part of year (ESUW). 2 ♀♀, top label - Costa Rica: Guanacaste [;] Santa Rosa Natl. Park [;] 300m, ex. Malaise trap [;] Site #: blank [;] Dates: 20.xii.86–10.i.1987 [;] I.D. Gauld & D. Janzen; second label - [BH] Bosque Humedo [;] mature evergreen dry forest [;] [C] more or less fully [;] shaded as possible (ESUW). 3 ♀♀, top label - Costa Rica: Guanacaste [;] Santa Rosa Natl. Park [;] 300m, ex. Malaise trap [;] Site #: SE-O-5 and SE-7-O [;] Dates: 18.x–8.xi.1986 [;] I.D. Gauld & D. Janzen; second label - [SE] Bosque San Emilio [;] 50yr old deciduous forest [;] [O] in clearing, fully [;] isolated part of year (ESUW). 1 ♀, top label - Costa Rica: Guanacaste [;] Santa Rosa Natl. Park [;] 300m, ex. Malaise trap [;] Site #: SE-6-C [;] Dates: 18.x–8.xi.1986 [;] I.D. Gauld & D. Janzen; second label - [SE] Bosque San Emilio [;] 50yr old deciduous forest [;] [C] more or less fully [;] shaded as possible (ESUW). 1 ♀, Costa Rica: Guanacaste, ACT [;] Bagaces, P.N. Palo Verde [;] Sec. P. Verde, 0–50m [;] 2–12.xii.1999, I. Jimenez [;] L.N. 260932-385020 #54246 [;] Red de Golpe (ESUW). 4 ♀♀, Costa Rica: Guanacaste [;] P. N. Guanacaste [;] below Pitilla, 500m [;] 7–8.iii.1990, J. S. Noyes (ESUW). 1 ♀, Costa Rica, Guanacaste Pr. [;] Guanac. Conserv. Area [;] Estacion Pitilla, 680m [;] M.trap, 10–21 vii 1997 [;] 2x night, L.J. van der Ent (ESUW). 1 ♀, COSTA RICA: [;] Guanacaste [;] Estac. Mengo [;] SW Volcan Cacao [;] 1100m, 1988–1989 (ESUW). 1 ♀, Costa Rica: Guanacaste Pr. [;] Guanacaste National Park [;] near Headquarters [;] 1–10 March 1990, J.S. Noyes (ESUW). 1 ♀, COSTA RICA, Puntar [;] Golfo Dulce, 3km [;] SW. Rincón, 10m [;] III-VI-1990, Hanson (MICR). 1 ♀, Costa Rica [;] San Vito, Puntarenas [;] 1 January 1975 [;] M. Palmer (TAMU). 1 ♀, COSTA RICA: Puntarenas [;] RF Golfo Dulce, el 200m [;] 24km W Piedras Blancas [;] P. Hanson x.1992 (TAMU). 1 ♀, COSTA RICA: Puntarenas [;] San Vito, Estac. Biol. [;] Los Alturas 1500m [;] iv.1992 P. Hanson (TAMU). 1 ♀, S.RosaPark, Guan. [;] C. Rica 19–20.Jan.77 [;] D. H. Janzen [;] Riparian (AEIC). 1 ♀, C. Rica:Escazú [;] May 25, 1987 [;] H.&M.Townes (AEIC).

#### Comments.

This species, along with *Heterospilus once*, is distinguished by the weakly sclerotized base of metasomal terga 3–5. It can be distinguished from *Heterospilus once* by the white apical flagellomeres.

#### Etymology.

This species is named for Paul Hanson and his wife, Carolina Godoy, in recognition of their many years of collecting wasps in Costa Rica and in appreciation for their hospitality during my visits to Costa Rica.

**Figure 240.**
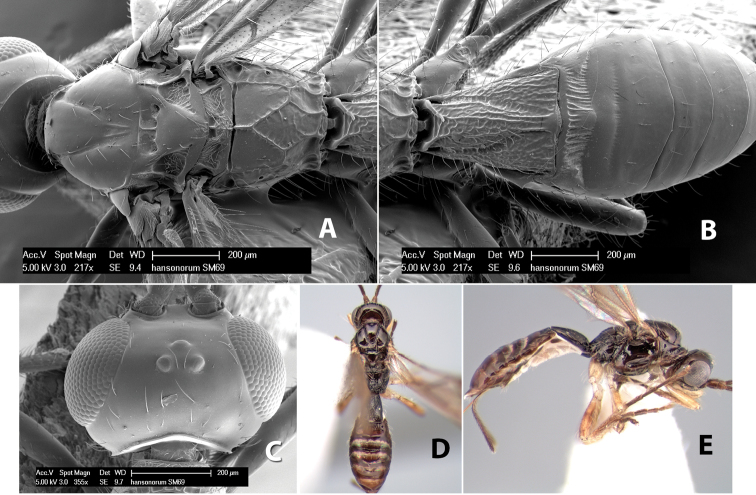
*Heterospilus hansonorum* Marsh, sp. n.: **A–C** paratype **D–E** holotype.

### Heterospilus hera

Marsh sp. n.

http://zoobank.org/02D5B413-D8FB-4C14-A77E-842A04276B32

http://species-id.net/wiki/Heterospilus_hera

Figure 241

#### Female.

Body size: 3.0–3.5 mm. Color: body dark brown, apical metasomal tergum yellow; scape honey yellow without lateral brown stripe; flagellum brown with apical 7–10 flagellomeres white, apical one sometimes darker; wing veins including stigma brown; legs yellow. Head: vertex smooth; frons smooth; face smooth; temple in dorsal view narrow, sloping behind eye, width less than 1/2 eye width; malar space greater than 1/4 eye height; ocell-ocular distance about 2.5 times diameter of lateral ocellus; 22–29 flagellomeres. Mesosoma: mesoscutal lobes smooth; notauli scrobiculate, meeting posteriorly in triangular costate area; scutellum smooth; prescutellar furrow with 3 cross carinae; mesopleuron smooth; precoxal sulcus smooth, shorter than mesopleuron; venter smooth; propodeum with basal median areas margined, smooth, basal median carina present, areola not distinctly margined, areolar area rugose, lateral areas rugose apically, smooth basally. Wings: fore wing vein r shorter than vein 3RSa, vein 1cu-a interstitial with or slightly beyond vein 1M; hind wing vein SC+R present, vein M+CU shorter than vein 1M. Metasoma: first tergum longitudinally costate, length nearly twice apical width; second tergum longitudinally costate; anterior transverse groove present but weak, sometimes absent; posterior transverse groove absent; third tergum entirely smooth; terga 4–7 smooth; ovipositor about 3/4 length of metasoma.

#### Holotype female.

Top label (white, printed) - Costa Rica: Golfo Dulce, 24km W. [;] Piedras Blancas, 200m [;] xii.1991, Paul Hanson; second label (red, partially printed and hand written) - HOLOTYPE [;] Heterospilus [;] hera [;] P. Marsh. Deposited in ESUW.

#### Paratypes.

1 ♀, Costa Rica: Puntarenas [;] Res. Forestal Golfo Dulce, 200m [;] xi.1990, P. Hanson [;] Malaise, Primary forest (ESUW). 1 ♀, Costa Rica: Puntarenas [;] Est. Cacao, 1000–1400m [;] 2km. SW del Cerro Cacao [;] vii.1996, J. A. Ugalde [;] L.N. 323100-375800 #8220 [;] Malaise trap (ESUW). 1 ♀, COSTA RICA, Guanac. [;] Estac, Pitilla, 9km S Santa Cecilia, 700m [;] IX/1988, I. Gauld (ESUW).

#### Comments.

The absent posterior transverse groove on metasomal tergum 3, the white apical annulus on the flagellum and the 3 cross carinae in the prescutellar furrow are distinctive for this species.

#### Etymology.

Named for the Greek goddess Hera.

**Figure 241.**
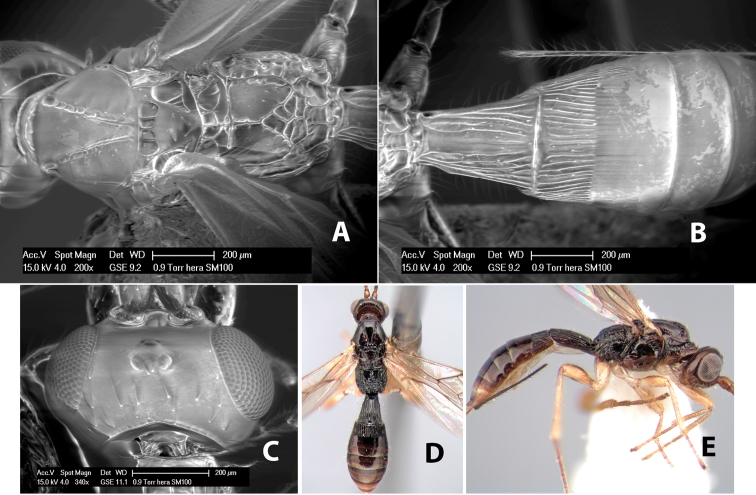
*Heterospilus hera* Marsh, sp. n.: **A–D** paratype **E** holotype.

### Heterospilus hespenheidei

Marsh sp. n.

http://zoobank.org/BAB29BEA-A1F3-4243-AE83-F9644EABE31A

http://species-id.net/wiki/Heterospilus_hespenheidei

Figure 242

#### Female.

Body size: 1.5 mm. Color: body dark brown, metasomal tergum 2 usually yellow; scape yellow without lateral brown stripe, flagellum brown, basal flagellomere often lighter; wing veins brown, stigma usually brown, occasionally lighter; legs yellow or light brown. Head: vertex smooth; frons smooth; face smooth; temple in dorsal view broad, slightly bulging behind eye, width equal to 1/2 eye width; malar space equal to 1/4 eye height; ocell-ocular distance slightly greater than 2.5 times diameter of lateral ocellus; 12–16 flagellomeres. Mesosoma: mesoscutal lobes weakly granulate; notauli weakly indicated or absent, usually smooth, meeting posteriorly in unsculptured area; scutellum weakly granulate; prescutellar furrow with 1 cross carina; mesopleuron smooth; precoxal sulcus scrobiculate, shorter than mesopleuron; venter smooth; propodeum with basal median areas margined, smooth, basal median carina absent, areola not margined, areolar area rugose, lateral areas entirely rugose. Wings: fore wing vein r slightly shorter than vein 3RSa, vein 1cu-a beyond vein 1M; hind wing vein SC+R absent, vein M+CU shorter than vein 1M. Metasoma: first tergum longitudinally costate, length equal to apical width; second tergum weakly longitudinally costate; anterior transverse groove weak, straight; posterior transverse groove weak or absent; third tergum entirely smooth; terga 4–7 smooth; ovipositor as long as metasoma.

#### Holotype female.

Top label (white, printed) - COSTA RICA: Heredia [;] Pr: La Selva Biol. Sta. [;] 3km S Pto. Viejo [;] 10°26'N, 84°01'W; second label (white, partially printed and hand written) - 19.v.1990 [;] H.A. Hespenheide [;] on dead citrus; third label (red, partially printed and hand written) - HOLOTYPE [;] Heterospilus [;] hespenheidei [;] P. Marsh. Deposited in ESUW.

#### Paratypes.

3 ♀♀, same data as holotype with additional dates of 18 and 20.v.1990 (ESUW).

#### Comments.

The long ovipositor, short body and weak notauli are distinctive for this species.

#### Etymology.

Named for the collector of the type series, Henry Hespenheide.

**Figure 242.**
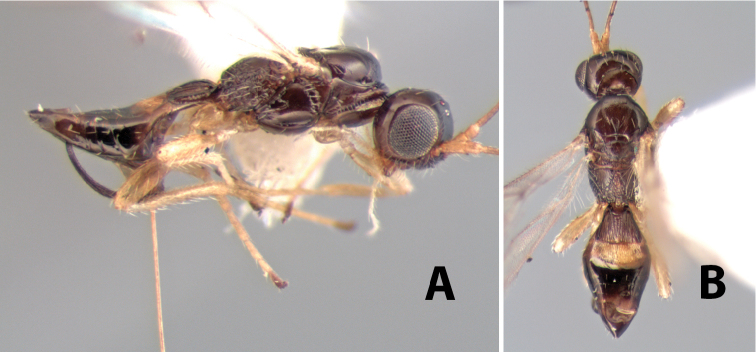
*Heterospilus hespenheidei* Marsh, sp. n., holotype.

### Heterospilus holleyae

Marsh sp. n.

http://zoobank.org/6DB85BCB-6BBB-4B8A-BCC9-837FC888DB2D

http://species-id.net/wiki/Heterospilus_holleyae

Figure 243

#### Female.

Body size: 4.0–4.5 mm. Color: entire body dark brown or black; scape brown, flagellum entirely dark brown; legs honey yellow, hind coxa brown anteriorly; wing veins including stigma dark brown. Head: vertex smooth; frons smooth, rarely weakly striate at antennal base; face rugose; temple in dorsal view somewhat narrow and sloping behind eye, width less than 1/2 eye width; malar space about 1/5 eye height; ocell-ocular distance about 2.5 times diameter of lateral ocellus; 34–36 flagellomeres. Mesosoma: mesoscutal lobes smooth; notauli smooth, meeting posteriorly in unsculptured area; scutellum smooth; prescutellar furrow with 3 cross carinae, middle carina stronger than lateral ones; mesopleuron smooth; precoxal sulcus smooth, shorter than mesopleuron; venter smooth; propodeum with basal median areas small, smooth and not distinctly margined, basal median carina absent or very short, areola not distinctly margined, areolar area rugose, lateral areas entirely rugose. Wings: fore wing vein r less than 1/2 length of vein 3RSa, vein 1cu-a beyond vein 1M; hind wing vein SC+R present, vein M+CU nearly as long as vein 1M. Metasoma: first tergum longitudinally costate, length greater than apical width; second tergum longitudinally costate; anterior transverse groove present, straight; posterior transverse groove present; third tergum longitudinally costate, smooth at extreme apex; terga 4 and 5 longitudinally costate, smooth at extreme apex; terga 6 and 7 smooth; ovipositor about as long as metasomal tergum 1.

#### Holotype female.

Top label (white, printed) - S.RosaPark, Guan. [;] C. Rica 8 Oct 77 [;] D.H. Janzen [;] Riparian; second label (red, printed) - HOLOTYPE [;] *Heterospilus* [;] *holleyae* Marsh. Deposited in AEIC.

#### Paratypes.

6 ♀♀, same data as holotype except: dates of 14. Dec. 76 to6 Oct 77; Dry Hill in addition to Riparian (AEIC).

#### Comments.

The costate metasomal terga 3–5, smooth notauli and brown coxa are distinctive for this species.

#### Etymology.

Named for Jo-Anne Holley, wife of Alex Wild.

**Figure 243.**
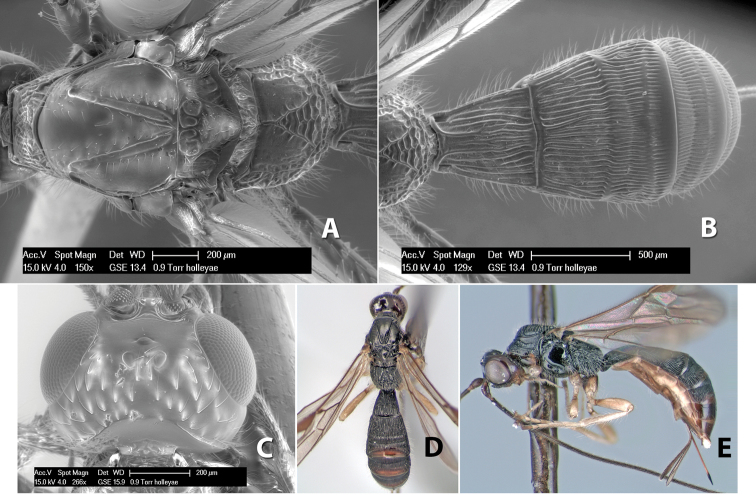
*Heterospilus holleyae* Marsh, sp. n., holotype.

### Heterospilus huetares

Marsh sp. n.

http://zoobank.org/CB303AC6-A8CF-49FA-BDBC-AA345EA4BCC5

http://species-id.net/wiki/Heterospilus_huetares

Figure 244

#### Female.

Body size: 2.5–3.0 mm. Color: body dark brown, propleuron yellow, apical metasomal terga yellow; scape yellow or light brown without lateral brown stripe; flagellum entirely brown; wing veins brown, stigma light brown or yellow; legs yellow. Head: vertex smooth; frons smooth; face smooth; temple in dorsal view broad but sloping behind eye, width equal to 1/2 eye width; malar space greater than 1/4 eye height; ocell-ocular distance about 2.5 times diameter of lateral ocellus; 26–31 flagellomeres. Mesosoma: mesoscutal lobes granulate; notauli scrobiculate, meeting posteriorly in triangular rugose-costate area; scutellum smooth; prescutellar furrow usually with 3 cross carinae, rarely with only one distinct carina; mesopleuron smooth; precoxal sulcus smooth, shorter than mesopleuron; venter smooth; propodeum with basal median areas margined granulate, rarely smooth, basal median carina present but short, areola not distinctly margined, areolar area rugose, lateral areas entirely rugose. Wings: fore wing vein r shorter than vein 3RSa, vein 1cu-a interstitial with or slightly beyond vein 1M; hind wing vein SC+R present, vein M+CU shorter than vein 1M. Metasoma: first tergum longitudinally costate, length slightly greater than apical width; second tergum longitudinally costate-granulate; anterior transverse groove present, straight; posterior transverse groove present; third tergum costate basally, smooth apically; terga 4–5 costate or granulate basally, smooth apically; terga 6–7 smooth; ovipositor longer than metasoma.

#### Holotype female.

Top label (white, printed) - Costa Rica: Heredia [;] 3km S. Puerto Viejo [;] OTS, La Selva, 100m [;] 1–15 ix 1992, P. Hanson [;] huertos Malaise trap [;] set by G. Wright; second label (red, partially printed and hand written) - HOLOTYPE [;] Heterospilus [;] huetares [;] P. Marsh. Deposited in ESUW.

#### Paratypes.

3 ♀♀, same data as holotype (ESUW). 2 ♀♀, Costa Rica: Heredia [;] Est. Biol. La Selva [;] 50–150m, 10.26 N [;] 84.01 W, Aug. 1992 (ESUW). 1 ♀, Costa Rica, Heredia [;] 3km. S. Puerto Viejo [;] OTS-La Selva, 100m [;] IV-V-1993, P. Hanson (ESUW). 1 ♀, Costa Rica: Heredia [;] 3km. S. Puerto Viejo [;] OTS - La Selva, 100m [;] 16–30 IX.1992 [;] P. Hanson (ESUW). 1 ♀, Costa Rica, Puntarenas [;] Pen. Osa, Puerto Jimenez [;] 10m, VI-1993, P. Hanson (ESUW).

#### Comments.

The brown flagellum and scape and the ovipositor being longer than the metasoma are distinctive for this species.

#### Etymology.

Named for the Huetares, an indigenous people of Costa Rica.

**Figure 244.**
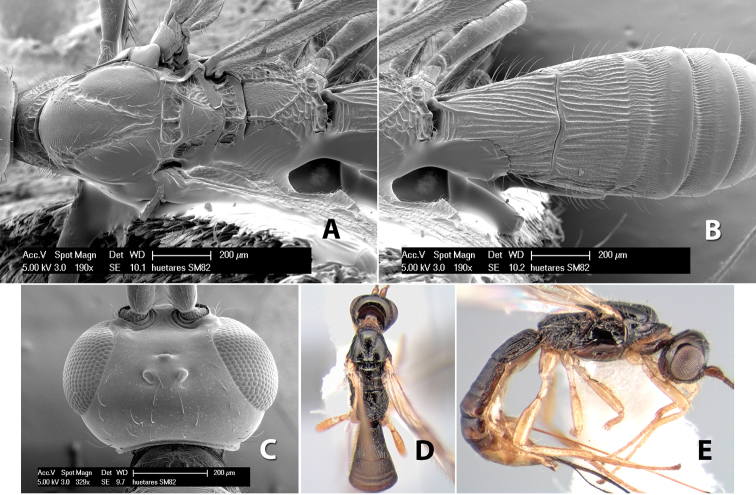
*Heterospilus huetares* Marsh, sp. n.: **A–C** paratype **D–E** holotype.

### Heterospilus hypermekus

Marsh sp. n.

http://zoobank.org/7E0A01D2-DF51-4AEA-858B-B978931F9B98

http://species-id.net/wiki/Heterospilus_hypermekus

Figure 245

#### Female.

Body size: 1.5 mm. Color: body dark brown, apical metasomal terga lighter brown; scape yellow without lateral brown stripe, flagellum brown; wing veins including stigma brown; fore legs yellow; mid and hind coxae and femora brown, trochanters and tarsi yellow, tibiae yellow on basal half, brown on apical half. Head: vertex smooth; frons smooth; face smooth; temple in dorsal view broad, bulging behind eye, width greater than 1/2 eye width; malar space greater than 1/4 eye height; ocell-ocular distance greater than 2.5 times diameter of lateral ocellus; 13 flagellomeres. Mesosoma: mesoscutal lobes granulate; notauli scrobiculate, meeting at scutellum in unsculptured area; scutellum granulate; prescutellar furrow with 3 cross carinae; mesopleuron granulate; precoxal sulcus smooth, shorter than mesopleuron; venter granulate; propodeum with basal median areas distinct but not margined, granulate, basal median carina absent, areola not margined, areolar area rugose, lateral areas entirely rugose. Wings: fore wing vein r shorter than vein 3RSa, vein 1cu-a beyond vein 1M; hind wing vein SC+R absent, vein M+CU slightly longer than vein 1M. Metasoma: first tergum longitudinally costate, length slightly greater than apical width; second tergum longitudinally costate; anterior transverse groove present, straight; posterior transverse groove present; third tergum smooth entirely; terga 4–7 smooth; ovipositor as long as entire body.

#### Holotype female.

Top label (white, partially printed and hand written) - Costa Rica: Guanacaste [;] Santa Rosa Natl. Park [;] 300m, ex. Malaise trap [;] Site #: (blank) [;] Dates: 13.iv–4.v.1986 [;] I.D. Gauld & D. Janzen; second label (white, printed) - [H] open regenerating [;] woodland <10 years old [;] [C] more or less fully [;] shaded as possible; third label (red, partially printed and hand written) - HOLOTYPE [;] Heterospilus [;] hypermekus [;] P. Marsh. Deposited in ESUW.

#### Paratypes.

1 ♀, same data as holotype, dates of 4–24.v.1986, second label, [SE] Bosque San Emilio [;] 50yr old deciduous forest [;] [C] more or less fully [;] shaded as possible (ESUW).

#### Comments.

The very long ovipositor, broad temple and brown flagellum are distinctive for this species.

#### Etymology.

The specific name is from the Greek *hypermekes*, meaning very long, in reference to the unusually long ovipositor.

**Figure 245.**
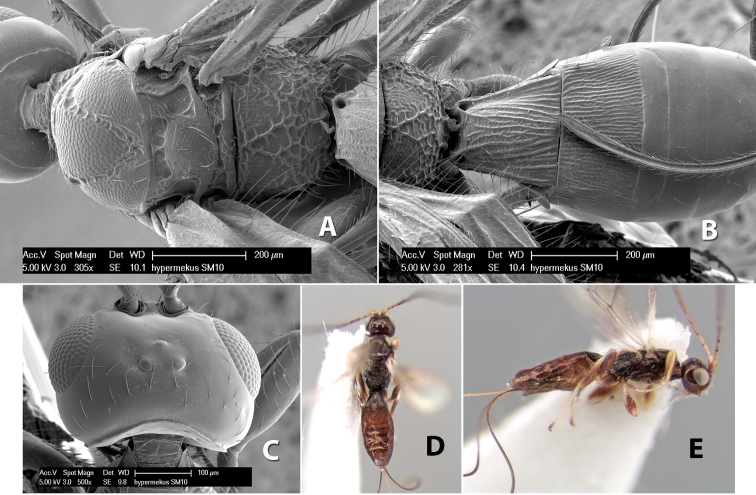
*Heterospilus hypermekus* Marsh, sp. n.: **A–C** paratype **D–E** holotype.

### Heterospilus jupiter

Marsh sp. n.

http://zoobank.org/67227EE2-972E-4A4F-B720-F0ED4274A9BB

http://species-id.net/wiki/Heterospilus_jupiter

Figure 246

#### Female.

Body size: 2.0 mm. Color: body including legs entirely yellow; scape yellow without lateral brown stripe; flagellum brown with basal flagellomeres lighter; wing veins light brown, stigma yellow. Head: vertex smooth; frons smooth, rarely with few weak striations; face smooth; temple in dorsal view broad, width equal to 1/2 eye width; malar space greater than 1/4 eye height; ocell-ocular distance about 2.5 times diameter of lateral ocellus; 15–19 flagellomeres. Mesosoma: mesoscutal lobes granulate, rarely partially smooth; notauli scrobiculate, meeting posteriorly in triangular costate area; scutellum smooth or rarely weakly granulate; prescutellar furrow with 1 cross carina; mesopleuron smooth; precoxal sulcus smooth, shorter than mesopleuron; venter smooth; propodeum with basal median areas margined, smooth or rarely weakly granulate, basal median carina present and long, areola not completely margined, areolar area rugose, lateral areas rugose apically, smooth basally. Wings: fore wing vein r shorter than vein 3RSa, vein 1cu-a beyond vein 1M; hind wing vein SC+R present, vein M+CU shorter than vein 1M. Metasoma: first tergum longitudinally costate, length slightly greater than apical width; second tergum weakly longitudinally costate; anterior transverse groove weak or absent; posterior transverse groove weak or absent; third tergum entirely smooth; terga 4–7 smooth; ovipositor half as long as metasoma.

#### Holotype female.

Top label (white, partially printed and hand written) - Costa Rica: Guanacaste [;] Santa Rosa Natl. Park [;] 300m, ex. Malaise trap [;] Site #: (blank) [;] Dates: 31.I-21.II.1987 [;] I.D. Gauld & D. Janzen; second label (white, printed) - [SE] Bosque San Emilio [;] 50yr old deciduous forest [;] [C] more or less fully [;] shaded as possible; third label (red, partially printed and hand written) - HOLOTYPE [;] Heterospilus [;] jupiter [;] P. Marsh. Deposited in ESUW.

#### Paratypes.

1 ♀, same data as holotype (ESUW). 2 ♀♀, COSTA RICA, San Jose [;] San Antonio de Escazu [;] 1300m, III 1989 [;] Col. W. Eberhard (ESUW). 1 ♀, Costa Rica, San Jose [;] Zurqui de Moravia [;] 1600m III-1996 [;] P. Hanson (ESUW). 2 ♀♀, S.RosaPark, Guan. [;] C. Rica 3–4 Dec., 76 [;] D. H. Janzen [;] Dry Hill (AEIC).

#### Comments.

The yellow body, single cross carina in the prescutellar furrow and the long median basal carina on the propodeum are distinctive for this species.

#### Etymology.

Named for the Roman god Jupiter.

**Figure 246.**
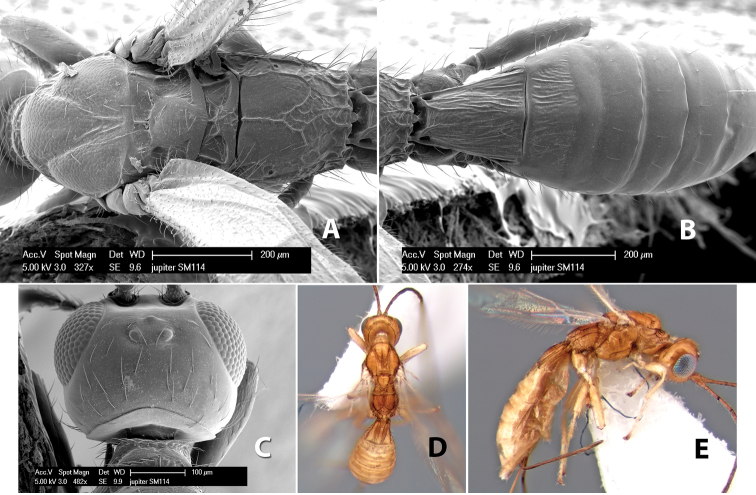
*Heterospilus jupiter* Marsh, sp. n.: **A–C** paratype **D–E** holotype.

### Heterospilus leiponotaulus

Marsh sp. n.

http://zoobank.org/01990EEF-7F33-4A35-8809-E6151F7708E1

http://species-id.net/wiki/Heterospilus_leiponotaulus

Figure 247

#### Female.

Body size: 1.5 mm. Color: body brown to light brown, propodeum, metasomal terga 1 and 2 often yellow; scape yellow without lateral brown stripe; flagellum brown; legs yellow. Head: vertex smooth; frons smooth; face smooth; temple in dorsal view broad, bulging behind eye, width greater than 1/2 eye width; malar space greater than 1/4 eye height; ocell-ocular distance greater than 2.5 times diameter of lateral ocellus; 12 flagellomeres. Mesosoma: mesoscutal lobes smooth; notauli present only anteriorly or absent completely, area where they would meet at apex of median mesoscutal lobe represented by short shallow groove; scutellum smooth; prescutellar furrow with 3 cross carinae; mesopleuron smooth; precoxal sulcus weakly scrobiculate or nearly smooth, shorter than mesopleuron; venter smooth; propodeum with basal median areas not distinctly margined, granulate, basal median carina absent, areola not margined, areolar area rugose, lateral areas entirely rugose. Wings: fore wing vein r shorter than vein 3RSa, vein 1cu-a beyond vein 1M; hind wing vein SC+R present, vein M+CU shorter than vein 1M. Metasoma: first tergum longitudinally costate, length greater than apical width; second tergum smooth; anterior transverse groove absent; posterior transverse groove absent; third tergum smooth; terga 4–7 smooth; ovipositor longer than metasoma.

#### Holotype female.

Top label (white, printed) - Costa Rica: Cartago [;] 4km NE Cañon [;] Genesis II, 2350m [;] vii.1995, P. Hanson; second label (red, partially printed and hand written) - HOLOTYPE [;] Heterospilus [;] leiponotaulus [;] P. Marsh. Deposited in ESUW.

#### Paratypes.

1 ♀, top label - Costa Rica: Guanacaste [;] Santa Rosa Natl. Park [;] 300m, ex. Malaise trap [;] Site #: (blank) [;] Dates: 31-I-21-II-1987 [;] I.D. Gauld & D. Janzen; second label - [SE] Bosque San Emilio [;] 50yr old deciduous forest [;] [C] more or less fully [;] shaded as possible (ESUW). 1 ♀, Costa Rica, Carthago Pr. [;] La Cangreja, 1950m [;] I 1992, P. Hanson (ESUW).

#### Comments.

This species is distinguished by the weak or absent notauli, the smooth metasomal terga 2–3, the short antennae and the wide temple.

#### Etymology.

The specific name is from the Greek *leipo*, meaning be wanting or without, in reference to the absent notauli.

**Figure 247.**
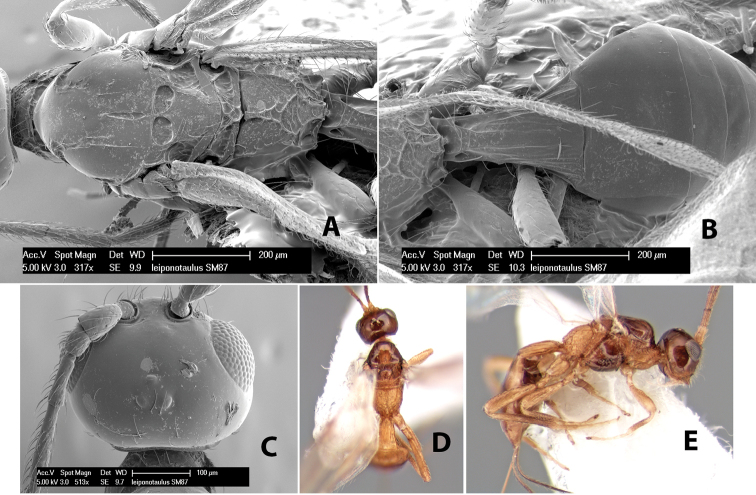
*Heterospilus leiponotaulus* Marsh, sp. n.: **A–C** paratype **D–E** holotype.

### Heterospilus leviscutum

Marsh sp. n.

http://zoobank.org/FF84B04A-D6E8-4A1E-BD24-03903485C597

http://species-id.net/wiki/Heterospilus_leviscutum

Figure 248

#### Female.

Body size: 2.5–3.0 mm. Color: body brown to dark brown, metasomal terga 3–5 honey yellow at apex, terga 6–7 honey yellow; scape brown; flagellum brown with apical 5–7 flagellomeres white, apical one often darker; wing veins brown, stigma yellow or light brown; legs yellow, femora with brown swelling dorsally, mid and hind femora brown on apical ⅓. Head: vertex smooth; frons smooth; face smooth; temple in dorsal view narrow, width less than 1/2 eye width; malar space greater than 1/4 eye height; ocell-ocular distance greater than 2.5 times diameter of lateral ocellus; 18–22 flagellomeres. Mesosoma: mesoscutal lobes smooth; notauli weakly scrobiculate, meeting posteriorly in unsculptured area but with two short longitudinal carinae; scutellum smooth; prescutellar furrow with 3 cross carinae; mesopleuron smooth; precoxal sulcus smooth, shorter than mesopleuron; venter smooth; propodeum with basal median areas margined, smooth, basal median carina present but short, areola not margined, areolar area rugose, lateral areas entirely rugose. Wings: fore wing vein r shorter than vein 3RSa, vein 1cu-a interstitial with vein 1M; hind wing vein SC+R present, vein M+CU shorter than vein 1M. Metasoma: first tergum longitudinally costate, length slightly greater than apical width; second tergum longitudinally costate; anterior transverse groove present, straight; posterior transverse groove weak or absent; third tergum costate basally, smooth apically; terga 4–7 smooth; ovipositor as long as metasomal terga 1–2 combined.

#### Holotype female.

Top label (white, printed) - Costa Rica: Puntarenas [;] R.F. Golfo Dulce, 3km. [;] S.W. Rincon, 10m [;] I.1992, P. Hanson; second label (red, partially printed and hand written) - HOLOTYPE [;] Heterospilus [;] leviscutum [;] P. Marsh. Deposited in ESUW.

#### Paratypes.

1 ♀, Costa Rica, Puntarenas [;] San Vito, Estac. Biol. [;] Las Alturas, 1500m [;] 15–31 Oct. 1991 P. Hanson (ESUW). 1 ♀, Costa Rica: Puntarenas, ACO [;] Golfito, R.F. Golfo Dulce [;] Est. Agujas, 250–350m [;] 4–20.vi.1999, J. Azofeifa [;] L.S. 276750-526550 #52746 [;] Amarilla (ESUW). 1 ♀, COSTA RICA: Puntar. [;] R.B. Carara, Estac. Quebrada Bonita, 50m [;] V-VI 1989, P. Hanson (ESUW). 1 ♀, Costa Rica: Puntarenas [;] San Vito, Las Cruces [;] Wilson Botanical Gardens [;] 18–22.iii.1990, 1150m [;] J.S. Noyes (ESUW). 6 ♀♀, Costa Rica: Alajuela [;] 5km. W. San Ramon [;] 1200m, X-1996, April 1997, ii.1997 and iv.1997 [;] O. Castro & P. Hanson (ESUW). 1 ♀, Costa Rica: Alajuela [;] San Carlos, R.F. Arenal [;] Sendero Pilon, 600m [;] 17–18.v.1999, G. Carballo [;] L.N.269100-457900 #53363 [;] Malaise trap (ESUW). 1 ♀, COSTA RICA: Guanac. [;] Sotobosque, W side [;] Volcan Cacao, 1100m [;] II 1989, I. Gauld (ESUW). 1 ♀, Costa Rica: Guanacaste [;] Est. Biol. Maritza, 600m [;] xi.1996, C. Zuniga, Malaise [;] L.N. 326900-373000 #47554 (ESUW). 1 ♀, COSTA RICA: [;] Guanacaste [;] Estac. Mengo [;] SW Volcan Cacao [;] 1100m, 1988–1989 (ESUW). 1 ♀, COSTA RICA: Guanac. [;] Arenales, W. side [;] Volcan Cacao, 900m [;] 1988–1989 (ESUW). 1 ♀, COSTA RICA: [;] San Jose [;] Ciudad Colon [;] 800m, iii-iv 1990 [;] Col. Luis Fournier (ESUW). 2 ♀♀, Costa Rica: San Jose [;] San Antonia de Escazu [;] 1300m, vi-vii.1998 [;] W. Eberhard (ESUW). 1 ♀, COSTA RICA: San Jose, [;] Cerro de la Muerte, [;] 26km N San Isidro, 2100m, [;] ii-v.1992 [;] Paul Hanson (ESUW). 1 ♀, Costa Rica: Cartago [;] Braulio Carillo N.P. [;] 600m, 25.iii.1990 [;] J. S. Noyes, coll. (ESUW). 1 ♀, Costa Rica: Cartago [;] Turrialba, CATIE [;] 14–15 March 1990 [;] 700m, J.S. Noyes (ESUW). 1 ♀, COSTA RICA, Limon [;] sur de Iriquois [;] 300m, 23/V/1987 [;] Col. Paul Hanson (MICR). 2 ♀♀, Est. Cacao, 1000–1400m, [;] Lado SO Vol. Cacao, [;] P.N.G., Prov. Guan. [;] COSTA RICA, C. [;] Chaves, Ago 1991, [;] L-N-323300,375700 (INBC). 1 ♀, COSTA RICA. Prov. Puntarenas. R.F. [;] Golfo Dulce. Est. Agujas. 300m. 12 [;] MAY 2001. J. Azofeifa. Libre [;] L_S_276750_526550 #63266 (INBC).

#### Comments.

The smooth and polished mesoscutum, flagellum with white apical flagellomeres, unsculptured area where notauli meet posteriorly and the short ovipositor are distinctive for this species.

#### Etymology.

The specific name is from the Latin *levis*, meaning smooth, polished, in reference to the smooth and polished mesoscutum.

**Figure 248.**
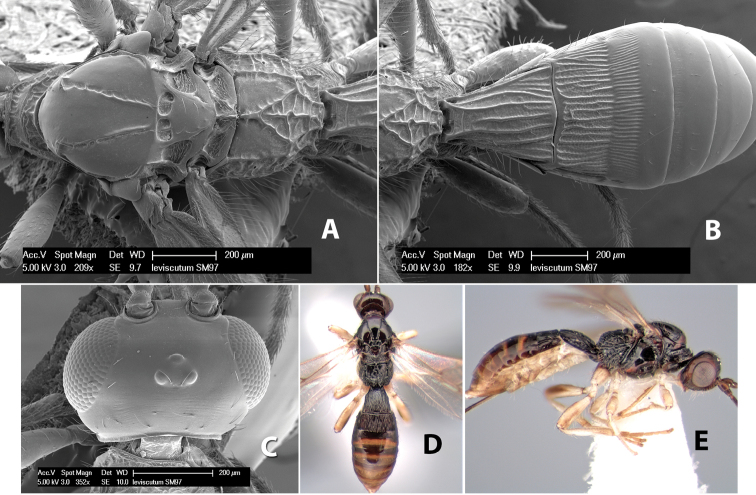
*Heterospilus leviscutum* Marsh, sp. n.: **A–C** paratype **D–E** holotype.

### Heterospilus levitergum

Marsh sp. n.

http://zoobank.org/ADFFE21F-A7D6-480D-857C-416D1F832B30

http://species-id.net/wiki/Heterospilus_levitergum

Figure 249

#### Female.

Body size: 2.0–2.5 mm. Color: head dark brown; scape yellow without lateral brown stripe; flagellum brown with apical 3–5 flagellomeres white; mesosoma dark brown; wing veins including stigma brown; legs bicolored, coxae and trochanters nearly white, femora, tibiae and tarsi yellow or light brown; metasomal tergum 1 dark brown, tergum 2 yellow, terga 3–7 brown. Head: vertex smooth; frons smooth; face smooth; temple in dorsal view narrow, sloping behind eye, width less than 1/2 eye width; malar space greater than 1/4 eye height; ocell-ocular distance slightly greater than 2.5 times diameter of lateral ocellus; 13–17 flagellomeres. Mesosoma: mesoscutal lobes smooth, rarely weakly granulate; notauli smooth, meeting posteriorly in triangular costate area; scutellum smooth; prescutellar furrow with 1 cross carina; mesopleuron smooth; precoxal sulcus smooth, rarely weakly scrobiculate, extending to posterior margin of mesopleuron by distinct carinae; venter smooth; propodeum with basal median areas weakly granulate or smooth, basal median carina present, areola distinctly margined, areolar area rugose, lateral areas entirely rugose. Wings: fore wing vein r shorter than vein 3RSa, vein 1cu-a interstitial with vein 1M; hind wing vein SC+R present, vein M+CU shorter than vein 1M. Metasoma: first tergum longitudinally costate, length nearly twice apical width; second tergum mostly smooth with few costae medially at base; anterior transverse groove present, straight; posterior transverse groove weakly present or absent; third tergum entirely smooth; terga 4–7 smooth; ovipositor 3/4 length of metasoma.

#### Holotype female.

Top label (white, printed) - Costa Rica: Heredia [;] 3km. S. Puerto Viejo, [;] OTS, La Selva, 100m [;] xii.1992, P. Hanson; second label (red, partially printed and hand written) - HOLOTYPE [;] Heterospilus [;] levitergum [;] P. Marsh. Deposited in ESUW.

#### Paratypes.

1 ♀, Costa Rica, Carthago Pr. [;] Dulce Nombre, Vivero [;] Linda Vista, 1300m [;] 1993: viii-x, P. Hanson (ESUW). 1 ♀, Costa Rica: Cartago [;] Turrialba, CATIE [;] 14–15.iii.1990 [;] 700m, J. S. Noyes (ESUW).

#### Comments.

The smooth metasomal terga 2–7, the single cross carina in the prescutellar furrow and the apical white annulus on the flagellum are distinctive for this species.

#### Etymology.

The specific name is from the Latin *levis*, meaning smooth, polished, in reference to the smooth metasomal terga.

**Figure 249.**
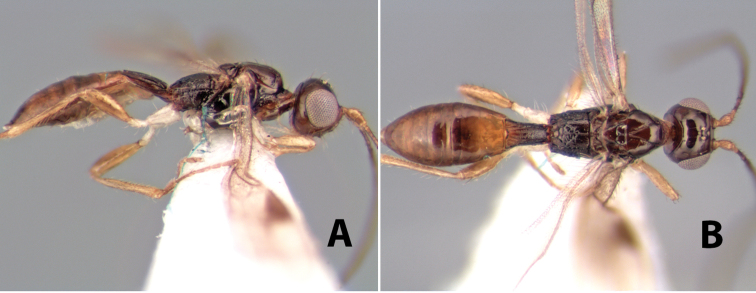
*Heterospilus levitergum* Marsh, sp. n., holotype.

### Heterospilus longius

Marsh sp. n.

http://zoobank.org/40CF9025-AE97-4786-BCF3-1B69F802EB3B

http://species-id.net/wiki/Heterospilus_longius

Figures 250 , 251

#### Female.

Body size: 2.5–3.0 mm. Color: head honey yellow except vertex brown; scape yellow without lateral brown stripe, flagellum yellow basally to brown apically; mesosoma dark brown; metasomal terga dark brown, tergum 2 yellow; wing veins including stigma brown; legs yellow. Head: vertex smooth or rarely weakly costate behind ocelli; frons smooth or weakly costate; face smooth, weakly costate or rugose; temple in dorsal view narrow, width less than 1/2 eye width; malar space equal to 1/4 eye height; ocell-ocular distance about 1.5 times diameter of lateral ocellus; 16–21 flagellomeres. Mesosoma: mesoscutal lobes granulate; notauli scrobiculate, meeting at scutellum in triangular costate-rugose area; scutellum granulate; prescutellar furrow with 3 cross carinae; mesopleuron granulate; precoxal sulcus scrobiculate, shorter than mesopleuron; venter granulate; propodeum with basal median areas not distinctly margined, granulate, basal median carina absent, areola not margined, areolar area areolate-rugose, lateral areas entirely rugose, apical lateral corners produced into distinct small tubercle. Wings: fore wing vein r longer than vein 3RSa, rarely equal, vein 1cu-a beyond vein 1M; hind wing vein SC+R absent, vein M+CU shorter than vein 1M. Metasoma: first tergum longitudinally costate, length equal to apical width; second tergum longitudinally costate; anterior transverse groove absent or rarely weakly indicated; posterior transverse groove absent or rarely weakly present; third tergum smooth; terga 4–7 smooth; ovipositor as long as metasomal terga 1 and 2 combined.

#### Holotype female.

Top label (white, printed) - COSTA RICA, San Jose [;] San Antonio de Escazu [;] 1300, IV/1987 [;] Col. W. Eberhard; second label (red, partially printed and hand written) - HOLOTYPE [;] Heterospilus [;] longius [;] P. Marsh. Deposited in ESUW.

#### Paratypes.

1 ♀, top label - Costa Rica: Guanacaste [;] Santa Rosa Natl. Park [;] 300m, ex. Malaise trap [;] Site #: SE-6-C [;] Dates: 2–23.iii.1986 [;] I.D. Gauld & D. Janzen; second label - [SE] Bosque San Emilio [;] 50yr old deciduous forest [;] [C] more or less fully [;] shaded as possible (ESUW). 2 ♀♀, top label - Costa Rica: Guanacaste [;] Santa Rosa Natl. Park [;] 300m, ex. Malaise trap [;] Site #: 6 [;] Dates: 8.ii–2.ii.1986 [;] I.D. Gauld & D. Janzen; second label - [SE] Bosque San Emilio [;] 50yr old deciduous forest [;] [C] more or less fully [;] shaded as possible (ESUW). 1 ♀, top label - Costa Rica: Guanacaste [;] Santa Rosa Natl. Park [;] 300m, ex. Malaise trap [;] Site #: 8 [;] Dates: 28.xii.85–18.i.1986 [;] I.D. Gauld & D. Janzen; second label - [SE] Bosque San Emilio [;] 50yr old deciduous forest [;] [C] more or less fully [;] shaded as possible (ESUW). 1 ♀, top label - Costa Rica: Guanacaste [;] Santa Rosa Natl. Park [;] 300m, ex. Malaise trap [;] Site #: SE.6.C [;] Dates: 18.x–8.xi.1986 [;] I.D. Gauld & D. Janzen; second label - [SE] Bosque San Emilio [;] 50yr old deciduous forest [;] [C] more or less fully [;] shaded as possible (ESUW). 1 ♀, top label - Costa Rica: Guanacaste [;] Santa Rosa Natl. Park [;] 300m, ex. Malaise trap [;] Site #: 7 [;] Dates: 26.vii–14,viii.1986 [;] I.D. Gauld & D. Janzen; second label - [SE] Bosque San Emilio [;] 50yr old deciduous forest [;] [O] in clearing, fully [;] isolated part of day (ESUW). 1 ♀, top label - Costa Rica: Guanacaste [;] Santa Rosa Natl. Park [;] 300m, ex. Malaise trap [;] Site #: blank [;] Dates: 8.ii–2.iii.1986 [;] I.D. Gauld & D. Janzen; second label - [SE] Bosque San Emilio [;] 50yr old deciduous forest [;] [O] in clearing, fully [;] isolated part of day (ESUW). 1 ♀, top label - Costa Rica: Guanacaste [;] Santa Rosa Natl. Park [;] 300m, ex. Malaise trap [;] Site #: SE-6-C [;] Dates: 2–23.iii.1986 [;] I.D. Gauld & D. Janzen; second label - [SE] Bosque San Emilio [;] 50yr old deciduous forest [;] [O] in clearing, fully [;] isolated part of day (ESUW). 1 ♀, top label - Costa Rica: Guanacaste, Santa [;] Rosa Nat’l. Park, Bosque San [;] Emilio, trap #7 in clearing, 300m [;] II/8-III/2/1986, I. Gauld; second label - [SE] Bosque San Emilio [;] 50yr old deciduous forest [;] [O] in clearing, fully [;] isolated part of day (ESUW). 1 ♀, top label - Costa Rica: Guanacaste [;] Santa Rosa Natl. Park [;] 300m, ex. Malaise trap [;] Site #: blank [;] Dates: 10–31.i.1987 [;] I.D. Gauld & D. Janzen; second label - [H] open regenerating [;] woodland, 10 years old [;] [O] in clearing, fully [;] isolated part of day (ESUW). 1 ♀, top label - Costa Rica: Guanacaste [;] Santa Rosa Natl. Park [;] 300m, ex. Malaise trap [;] Site #: H-2-O [;] Dates: 20.xii.86–10.i.1987 [;] I.D. Gauld & D. Janzen; second label - [H] open regenerating [;] woodland, 10 years old [;] [O] in clearing, fully [;] isolated part of day (ESUW). 1 ♀, top label - Costa Rica: Guanacaste [;] Santa Rosa Natl. Park [;] 300m, ex. Malaise trap [;] Site #: H-1-O [;] Dates: 20.xii.86–10.i.1987 [;] I.D. Gauld & D. Janzen; second label - [H] open regenerating [;] woodland, 10 years old [;] [O] in clearing, fully [;] isolated part of day (ESUW). 1 ♀, top label - Costa Rica: Guanacaste [;] Santa Rosa Natl. Park [;] 300m, ex. Malaise trap [;] Site #: 11 [;] Dates: 13.iv–4.v.1986 [;] I.D. Gauld & D. Janzen; second label - [H] open regenerating [;] woodland, 10 years old [;] [O] in clearing, fully [;] isolated part of day (ESUW). 1 ♀, top label - Costa Rica: Guanacaste [;] Santa Rosa Natl. Park [;] 300m, ex. Malaise trap [;] Site #: 3 [;] Dates: 7–28.xii.1985 [;] I.D. Gauld & D. Janzen; second label - [H] open regenerating [;] woodland, 10 years old [;] [O] in clearing, fully [;] isolated part of day (ESUW). 1 ♀, Costa Rica: top label - Guanacaste [;] Santa Rosa National Pk. [;] 300m, Malaise, Ian Gauld [;] 10–31.i.1987; second label - Bosque San Emilio [;] 50 yr. old deciduous [;] forest [;] Full Shade; third label - SE-6-O [;] 10–31.i.87 (ESUW). 1 ♀, Costa Rica: top label - Guanacaste [;] Santa Rosa National Pk. [;] 300m, Malaise, Ian Gauld [;] 18.x–8.xi.1986; second label - Open regenerating [;] Woodland less than [;] 10 yrs. old, Sun; third label - H-3-O [;] 18.x–8.xi.86 (ESUW). 3 ♀♀, Costa Rica, Guanacaste Pr. [;] Guan. Conservation Area [;] Santa Rosa hdq., 200m [;] lighttrap, 27-30-VI 1997 and 7-VII 1997 [;] L.J. van der Ent (ESUW). 3 ♀♀, Costa Rica: Limon [;] Sector Cocori, 100m [;] 30km N Cariari, i.1995 and iii.1995 [;] E. Rojas, Malaise #4526 and 4524 [;] L.N. 286000-567500 (ESUW). 1 ♀, Costa Rica: Puntarenas [;] R.F. Golfo Dulce, 5km. [;] W. Piedras Blancas, 100m [;] xi-xii.1991, P. Hanson [;] Malaise nr. second growth (ESUW). 2 ♀♀, Costa Rica: Puntarenas [;] R. F. Golfo Dulce, [;] 3km SW. Rincon, 10m, [;] vi.1991, Paul Hanson (ESUW). 1 ♀, COSTA RICA: Punta. [;] Golfo Dulce, 3km SW [;] Rincon [;] 10m, vii-ix 1990 [;] Col. Paul Hanson (ESUW). 1 ♀, Costa Rica: Puntarenas [;] San Vito, Las Cruces [;] Wilson Botanical Gardens [;] 18–22.iii.1990, 1150m [;] J.S. Noyes (ESUW). 1 ♀, Costa Rica: San Jose [;] 26km. N. San Isidro [;] just S. of Division [;] 2100m, viii-ix.1991 [;] P. Hanson, Malaise [;] secondary growth (ESUW). 1 ♀, COSTA RICA: [;] San Jose [;] Ciudad Colon [;] 800m, iii-iv 1990 [;] Col. Luis Fournier (ESUW). 1 ♀, Costa Rica: San Jose [;] Cerro de la Muerte [;] 6km. N. San Gerardo [;] 2800m, September 1997 [;] P. Hanson, Malaise (ESUW). 1 ♀, Costa Rica: Alajuela [;] 5km. W San Ramon [;] 1200m, April 1997 [;] O. Castro & P. Hanson (ESUW). 1 ♀, Costa Rica: Guanacaste [;] P. N. Guanacaste [;] below Pitilia, 500m [;] 7–8.iii.1990, J. S. Noyes (ESUW). 1 ♀, COSTA RICA: Guanac. [;] Estac. Mengo, S.W. [;] Volcan Cacao, 1100m [;] IX-X 1989 (ESUW). 1 ♀, Costa Rica: Limon, ACLAC [;] Central, R.B. Hitoy Cerere [;] Est. Hitoy Cerere, Send. [;] Catarata, 90m, Red de Golpe [;] 10.vii.1999, F. Umaña [;] L.N. 184600-643400 #52657 (ESUW). 1 ♀, Costa Rica: Heredia [;] 3km. S. Puerto Viejo, [;] OTS, La Salve, 100m [;] xii.1992, P. Hanson (ESUW). 1 ♀, Costa Rica: Cartago [;] Turrialba, CATIE [;] 14–15 March 1990 [;] 700m, J.S. Noyes (ESUW). 1 ♀, COSTA RICA, Limon [;] sur de Iriquois [;] 300m, 23/V/1987 [;] Col. Paul Hanson (MICR). 5 ♀♀, C. Rica:Escazú [;] May 21–26, 1987 [;] H.&M.Townes (AEIC). 7 ♀♀, S.RosaPark, Guan. [;] C. Rica various dates 9 Jan. 77 to 30 Jan 78 [;] D.H Janzen [;] Dry Hill and Riparian (AEIC).

#### Comments.

The long fore wing vein r, smooth metasomal terga 3–7 and the weak or absent transverse grooves on metasomal terga 2 and 3 are distinctive for this species.

#### Etymology.

The specific name is from the Latin *longius*, meaning longer, in reference to the fore wing vein r being longer than vein 3RSa.

**Figure 250.**
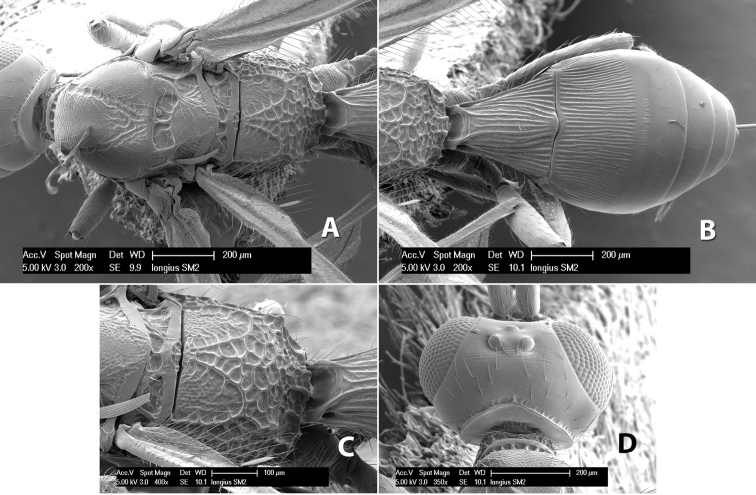
*Heterospilus longius* Marsh, sp. n., paratype.

**Figure 251.**
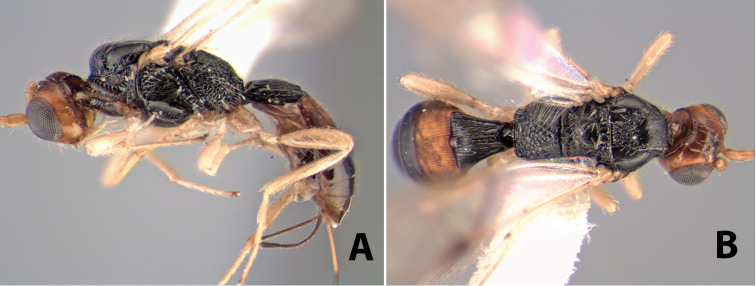
*Heterospilus longius* Marsh, sp. n., holotype.

### Heterospilus luteogaster

Marsh sp. n.

http://zoobank.org/866A9D9F-9B13-44AE-B61B-098FEEADC4D5

http://species-id.net/wiki/Heterospilus_luteogaster

Figure 252

#### Female.

Body size: 2.5–3.0 mm. Color: head and mesosoma dark brown, metasoma honey yellow or light brown, tergum 1 usually dark brown on basal ⅘; scape yellow without lateral brown stripe; flagellum brown; wing veins brown, stigma yellow; legs yellow. Head: vertex smooth; frons smooth, rarely weakly striate near antennal bases; face smooth; temple in dorsal view broad but not distinctly bulging behind eye, width equal to 1/2 eye width; malar space greater than 1/4 eye height; ocell-ocular distance greater than 2.5 times diameter of lateral ocellus; 21–28 flagellomeres. Mesosoma: mesoscutal lobes granulate; notauli scrobiculate, meeting posteriorly in triangular costate area; scutellum weakly granulate or smooth; prescutellar furrow with 3 cross carinae, often with median carina strong and lateral carinae weaker; mesopleuron smooth; precoxal sulcus weakly scrobiculate or smooth; venter smooth; propodeum with basal median areas margined, granulate, basal median carina present and short, areola not distinctly margined, areolar area rugose, lateral areas entirely rugose. Wings: fore wing vein r shorter than vein 3RSa, vein 1cu-a interstitial with vein 1M; hind wing vein SC+R present, vein M+CU as long as vein 1M. Metasoma: first tergum longitudinally costate-granulate, length equal to apical width; second tergum longitudinally costate-granulate; anterior transverse groove present, straight; posterior transverse groove present; third tergum costate basally, smooth at extreme apex; terga 4–7 costate or granulate at base, smooth apically; ovipositor half as long as metasoma.

#### Holotype female.

Top label (white, partially printed and hand written) - Costa Rica: Guanacaste [;] Santa Rosa Natl. Park [;] 300m, ex. Malaise trap [;] Site #: (blank) [;] Dates: 14.VIII-6.IX.1986 [;] I.D. Gauld & D. Janzen; second label (white, printed) - [SE] Bosque San Emilio [;] 50yr old deciduous forest [;] [C] more or less fully [;] shaded as possible; third label (red, partially printed and hand written) - HOLOTYPE [;] Heterospilus [;] luteogaster [;] P. Marsh. Deposited in ESUW.

#### Paratypes.

1 ♀, same data as holotype except Site #: SE-5-O (ESUW). 3 ♀♀, top label - Costa Rica: Guanacaste [;] Santa Rosa National Pk. [;] 300m, Malaise, Ian Gauld [;] 14.vi–5.vii.1986 and 27.ix–18.x.1986; second label - Bosque San Emilio [;] 50yr Old deciduous [;] Forest, SUN; third label - SE-7-O [;] 14.vi–5.vii.86 and 27.ix–18.x.86 (ESUW). 32 ♀♀, S.RosaPark, Guan. [;] C. Rica 25 May to 3 Dec 77 [;] D.H. Janzen [;] Riparian (AEIC). 2 ♀♀, Costa Rica [;] VII.29.77 [;] Riparian (AEIC). 1 ♀, S.RosaPark, Guan. [;] C. Rica 7 Dec., 76 (AEIC). 1 ♀, COSTA RICA: Escazu; [;] Jan. 10 1988; [;] W.T.Wcislo (AEIC).

#### Comments.

The yellow or light brown metasoma, the yellow stigma and the strongly granulate basal median areas of the propodeum are distinctive for this species.

#### Etymology.

The specific name is from the Latin *luteus*, meaning yellow, and the Greek *gaster*, meaning stomach, in reference to the yellow metasoma.

**Figure 252.**
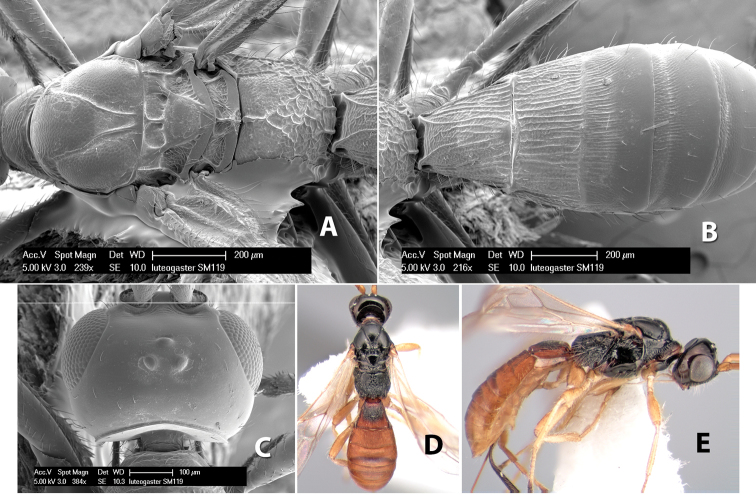
*Heterospilus luteogaster* Marsh, sp. n.: **A–C** paratype **D–E** holotype.

### Heterospilus luteoscutum

Marsh sp. n.

http://zoobank.org/14BC0388-9B20-4CCE-A894-2889D730A501

http://species-id.net/wiki/Heterospilus_luteoscutum

Figures 253 , 254

#### Female.

Body size: 5.0 mm. Color: head yellow; scape and flagellum dark brown; mesosoma with propleuron and median mesoscutal lobe yellow, remainder dark brown, propodeum laterally and mesopleuron dorsally slightly lighter; wing veins including stigma dark brown; legs with coxae, trochanters, femora and tibiae yellow, tarsi brown; metasomal tergum 1 dark brown, tergum 2 dark brown medially, yellow laterally, terga 3–5 dark brown medially at base, light brown apically and yellow laterally, terga 6–7 yellow. Head: vertex smooth; frons weakly transversely striate; face smooth; temple in dorsal view narrow, less than 1/2 eye width; malar space slightly greater than 1/4 eye height; ocell-ocular distance about 1.5 times diameter of lateral ocellus; 34 flagellomeres. Mesosoma: mesoscutal lobes granulate; notauli scrobiculate, meeting posteriorly in triangular rugose area; scutellum weakly granulate; prescutellar furrow with 3 cross carinae; mesopleuron smooth; precoxal sulcus scrobiculate, shorter than mesopleuron; venter smooth; propodeum with basal median areas margined, granulate, basal median carina present, areola not distinctly margined, areolar area rugose, lateral areas rugose apically, smooth basally. Wings: fore wing vein r shorter than vein 3RSa, vein 1cu-a beyond vein 1M; hind wing vein SC+R present, vein M+CU shorter than vein 1M. Metasoma: first tergum longitudinally costate, with several distinct cross carinae medially at base, length greater than apical width; second tergum longitudinally costate; anterior transverse groove present, sinuate; posterior transverse groove present; third tergum granulate basally, smooth apically; terga 4–7 smooth, terga 4 and 5 weakly granulate at base; ovipositor longer than metasoma.

#### Holotype female.

Top label (white, printed) - COSTA RICA: Puntarenas [;] San Vito, Estac. Biol. [;] Las Alturas, 1500m. [;] ii 1992, P. Hanson; second label (red, partially printed and hand written) - HOLOTYPE [;] Heterospilus [;] luteoscutum [;] P. Marsh. Deposited in ESUW.

#### Paratypes.

Known only from the holotype.

#### Comments.

The yellow mesoscutum, long ovipositor, sinuate anterior transverse groove of metasomal tergum 2 and the brown flagellum are distinctive for this species.

#### Etymology.

The specific name is from the Latin *luteus*, meaning yellow, in reference to the yellow median mesoscutal lobe.

**Figure 253.**
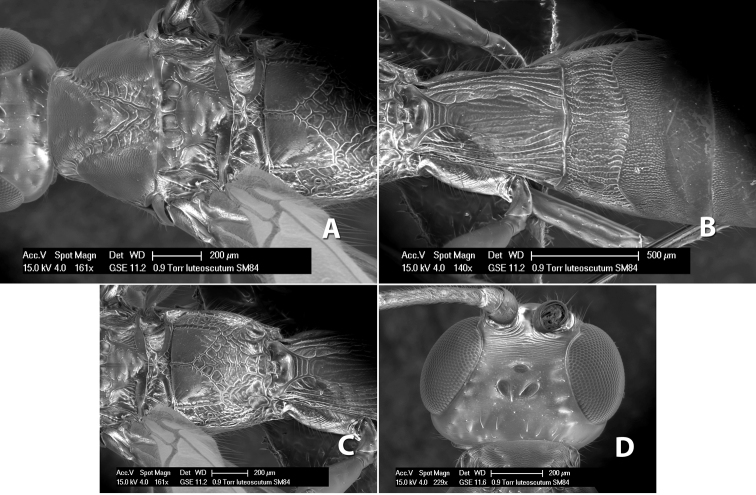
*Heterospilus luteoscutum* Marsh, sp. n., holotype.

**Figure 254.**
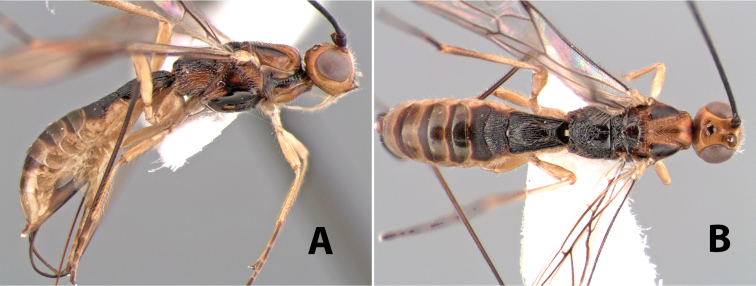
*Heterospilus luteoscutum* Marsh, sp. n., holotype.

### Heterospilus luteus

Marsh sp. n.

http://zoobank.org/1557B128-725D-4866-826B-5B5CF892A88D

http://species-id.net/wiki/Heterospilus_luteus

Figure 255

#### Female.

Body size: 1.5 mm. Color: body entirely yellow; scape yellow without lateral brown stripe, flagellum yellow basally to brown apically; wing veins including stigma light brown; legs yellow. Head: vertex smooth; frons smooth; face smooth; temple in dorsal view narrow, width less than 1/2 eye width; malar space slightly greater than or equal to 1/4 eye height; ocell-ocular distance about 2.5 times diameter of lateral ocellus; 13–15 flagellomeres. Mesosoma: mesoscutal lobes weakly granulate; notauli scrobiculate, meeting posteriorly in triangular rugose area; scutellum smooth; prescutellar furrow with 1 cross carina; mesopleuron smooth; precoxal sulcus weakly scrobiculate, shorter than mesopleuron; venter smooth; propodeum with basal median areas margined, smooth, basal median carina present, areola not margined, areolar area rugose, lateral areas entirely rugose. Wings: fore wing vein r slightly shorter or equal to vein 3RSa, vein 1cu-a slightly beyond vein 1m, rarely interstitial; hind wing vein SC+R absent, vein M+CU shorter than vein 1M. Metasoma: first tergum weakly longitudinally costate, length equal to apical width; second tergum weakly longitudinally costate; anterior transverse groove weakly indicated or absent; posterior transverse groove weakly indicated or absent; third tergum entirely smooth; terga 4–7 smooth; ovipositor as long as metasomal terga 1 and 2 combined.

#### Holotype female.

Top label (white, printed) - Costa Rica: San Jose [;] Cerro de la Muerte [;] 6km. N. San Gerardo [;] 2800m, September 1992 [;] P. Hanson, Malaise; second label (red, partially printed and hand written) - HOLOTYPE [;] Heterospilus [;] luteus [;] P. Marsh. Deposited in ESUW.

#### Paratypes.

1 ♀, top label - Costa Rica: Guanacaste [;] Santa Rosa Natl. Park [;] 300m, ex. Malaise trap [;] Site #: (blank) [;] Dates: 18.i–8.ii.1986 [;] I.D. Gauld & D. Janzen; second label - [H] open regenerating [;] woodland <10yrs old [;] [O] clearing, fully [;] isolated part of day (ESUW). 1 ♀, top label - Costa Rica: Guanacaste [;] Santa Rosa Natl. Park [;] 300m, ex. Malaise trap [;] Site #: H-1-O [;] Dates: 20.xii.86–10.i.1987 [;] I.D. Gauld & D. Janzen; second label - [H] open regenerating [;] woodland <10yrs old [;] [O] clearing, fully [;] isolated part of day (ESUW).

#### Comments.

The small and yellow body, short antennae and smooth vertex are distinctive for this species.

#### Etymology.

The specific name is from the Latin *luteus*, meaning yellow, in reference to the entirely yellow body.

**Figure 255.**
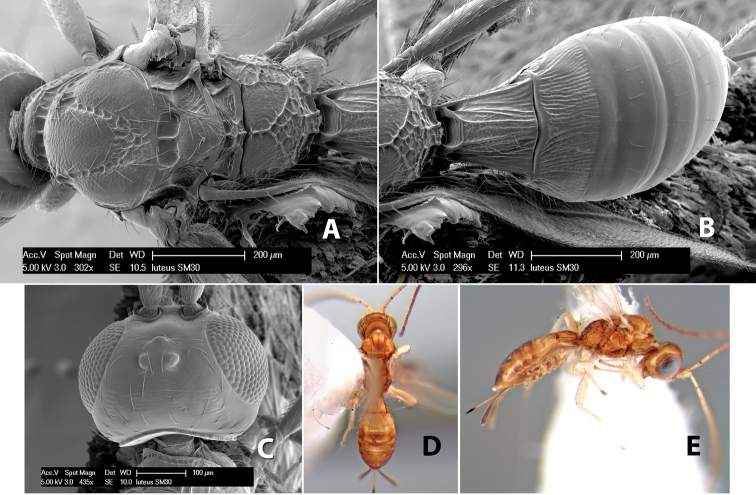
*Heterospilus luteus* Marsh, sp. n.: **A–C** paratype **D–E** holotype.

### Heterospilus macrocaudatus

Marsh sp. n.

http://zoobank.org/45E50B12-23D6-4DF0-AB5E-0BDDB6E7E721

http://species-id.net/wiki/Heterospilus_macrocaudatus

Figure 256

#### Female.

Body size: 3.5 mm. Color: body dark brown, apical metasomal terga lighter; scape brown; flagellum entirely brown or brown with apical 5–7 flagellomeres white; wing veins including stigma brown; legs yellow or light brown. Head: vertex smooth; frons smooth; face smooth; temple in dorsal view narrow, sloping behind eye, width equal to 1/2 eye width; malar space greater than 1/4 eye height; ocell-ocular distance greater than 2.5 times diameter of lateral ocellus; 21–25 flagellomeres. Mesosoma: mesoscutal lobes smooth; notauli scrobiculate, meeting posteriorly with 2 converging distinct carinae; scutellum smooth; prescutellar furrow with 3 cross carinae; mesopleuron smooth; precoxal sulcus smooth, shorter than mesopleuron; venter smooth; propodeum with basal median areas margined, smooth, basal median carina present, short, areola usually weakly margined, areolar area rugose, lateral areas entirely rugose. Wings: fore wing vein r shorter than vein 3RSa, vein 1cu-a interstitial with or slightly beyond vein 1M; hind wing vein SC+R present, vein M+CU shorter than vein 1M. Metasoma: first tergum longitudinally costate, length greater than apical width; second tergum longitudinally costate-granulate; anterior transverse groove present, straight; posterior transverse groove present; third tergum costate basally, smooth apically; terga 4–7 smooth; ovipositor nearly twice as long as metasoma.

#### Holotype female.

Top label (white, partially printed and hand written) - Costa Rica: Guanacaste [;] Santa Rosa Natl. Park [;] 300m, ex. Malaise trap [;] Site #: BH-12-C [;] Dates: 27.ix–18.x.1986 [;] I.D. Gauld & D. Janzen; second label (white, printed) - [BH] Bosque Humedo [;] mature evergreen dry forest [;] [C] more or less fully [;] shaded as possible; third label (red, partially printed and hand written) - HOLOTYPE [;] Heterospilus [;] macrocaudatus [;] P. Marsh. Deposited in ESUW.

#### Paratypes.

2 ♀♀, same data as holotype except: sites # of 6 and H-1-O; dates of 26.x–16.xi.1986 and 20.xii.86–10.i.1987; second label of [SE] Bosque San Emilio [;] 50yr old deciduous forest [;] [C] more or less fully [;] shaded as possible and [H] open regenerating [;] woodland <10 years old [;] [O] in clearing, fully [;] isolated part of day (ESUW). 1 ♀, Costa Rica: Guanacaste, ACT [;] Bagaces, P.N. Palo Verde [;] Sect. Catalina, 0–50m, de Luz [;] 8–12.xi.1999, I. Jimenez [;] L.N. 260952-385020 #53252 (ESUW). 1 ♀, Costa Rica: Guanacaste, ACT [;] Bagaces, P.N. Palo Verde, 212m [;] Sec. Palo Verde, Cerro Guayacan [;] 13.ix–13.x.1999, I. Jimenez, Malaise [;] L.N. 259350-389600 #53499 (ESUW). 1 ♀, Costa Rica, San Jose [;] San Ignacio [;] July 21 1980 [;] Wharton, Coll (TAMU).

#### Comments.

The unusually long ovipositor and the smooth mesoscutum are distinctive for this species. The flagellum varies from entirely brown to brown with apical flagellomeres white.

#### Etymology.

The specific name is from the Greek *macros*, meaning long, and the Latin *cauda*, meaning tail, in reference to the unusually long ovipositor.

**Figure 256.**
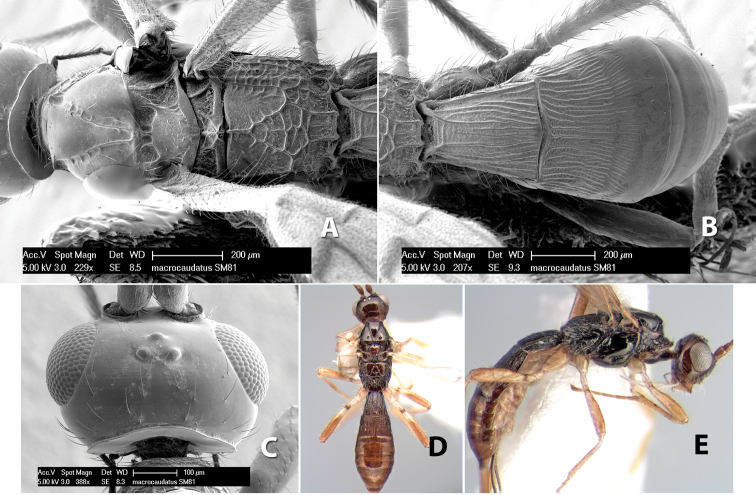
*Heterospilus macrocaudatus* Marsh, sp. n.: **A–C, E** paratype **D** holotype.

### Heterospilus malaisei

Marsh sp. n.

http://zoobank.org/FD21C01F-B1AD-48CD-9B45-C4C2805E7483

http://species-id.net/wiki/Heterospilus_malaisei

Figure 257

#### Female.

Body size: 3.5–4.0 mm. Color: body dark brown, apical metasomal terga somewhat lighter brown; scape yellow without lateral brown stripe; flagellum brown with apical 5–10 flagellomeres white, apical flagellomere occasionally brown; wing veins including stigma brown; legs yellow. Head: vertex smooth; frons smooth; face smooth; temple in dorsal view narrow, width less than 1/2 eye width; malar space slightly greater than 1/4 eye height; ocell-ocular distance about twice diameter of lateral ocellus; 25–29 flagellomeres. Mesosoma: mesoscutal lobes smooth; notauli scrobiculate, meeting posteriorly in triangular costate area; scutellum smooth; prescutellar furrow with 1 cross carina; mesopleuron granulate, occasionally smooth near precoxal sulcus; precoxal sulcus smooth, shorter than mesopleuron; venter smooth; propodeum with basal median areas margined and narrow, smooth, basal median carina present, areola distinctly margined, areolar area rugose, lateral areas rugose apically, granulate basally, propodeum with more or less distinct tubercle or raised carina apically on each side just above hind coxa and on each side of metasomal base. Wings: fore wing vein r shorter than vein 1M, vein 1cu-a beyond vein 1M; hind wing vein SC+R present, vein M+CU shorter than vein 1M. Metasoma: first tergum longitudinally costate, length about twice apical width; second tergum longitudinally costate; anterior transverse groove weak or occasionally absent, straight; posterior transverse groove weak or occasionally absent; third tergum entirely smooth; terga 4–7 smooth; ovipositor longer than metasoma.

#### Holotype female.

Top label (white, printed) - COSTA RICA: Puntarenas [;] San Vito, Las Cruces [;] 1200msnm, VIII-IX 1988 [;] Coll. P. Hanson; second label (red, partially printed and hand written) - HOLOTYPE [;] Heterospilus [;] malaisei [;] P. Marsh. Deposited in ESUW.

#### Paratypes.

2 ♀♀, Costa Rica: Puntarenas [;] R.F. Golfo Dulce, [;] 3km SW. Rincon, 10m [;] vi.1991, Paul Hanson (ESUW). 2 ♀♀, Costa Rica: Guanacaste [;] 2km SW de Cerro Cacao [;] Est. Cacao, 1000–1400m [;] 21–28.v.1992, Curso Biod. [;] L.N. 323300-375700 #6900 (ESUW). 2 ♀♀, Costa Rica: Guanacaste [;] Est. Cacao, 100–1150m [;] ix.1996, I. Villegas, Malaise [;] L.N. 323150-375700 #47559 (ESUW). 1 ♀, Costa Rica: Guanacaste [;] Est. Cacao, 1000–1150m [;] viii.1996, M. Pereira [;] L.N. 323150-375500 #47561 [;] Malaise trap (ESUW). 1 ♀, Costa Rica: Guanacaste [;] Est. Pitilia, 700m [;] 9km. S de Santa Cecilia [;] viii-ix.1996, P. Rios & [;] C. Moraga, Malaise [;] L.N. 329950-380450 #47563 (ESUW). 1 ♀, Costa Rica: San Jose [;] San Antonio de Escazu [;] 1300m, vi.1997 [;] W. Eberhard (ESUW). 1 ♀, COSTA RICA, Alajuela [;] Finca San Gabriel [;] 2 0 dos Rios, 600m [;] VIII/88, Col. Hanson (ESUW).

#### Comments.

The smooth mesoscutum, narrow temple and white apical flagellomeres are distinctive for this species.

#### Etymology.

Named for René Malaise whose invention of the Malaise trap enhanced our knowledge of Hymenoptera diversity in general and parasitic wasps in particular.

**Figure 257.**
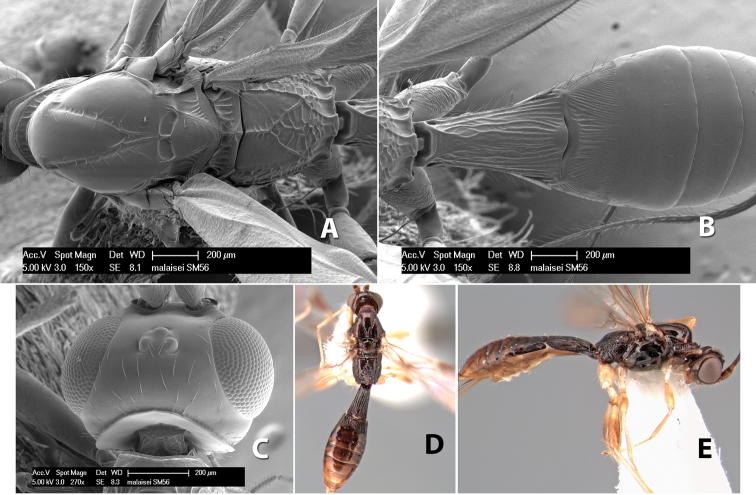
*Heterospilus malaisei* Marsh, sp. n.: **A–C** paratype **D–E** holotype.

### Heterospilus mars

Marsh sp. n.

http://zoobank.org/06547877-7EB0-4389-A25A-3D8B5E234AC9

http://species-id.net/wiki/Heterospilus_mars

Figure 258

#### Female.

Body size: 3.0 mm. Color: body dark brown, apical metasomal terga lighter brown; scape brown without lateral brown stripe; flagellum brown; wing veins brown, stigma yellow; legs yellow. Head: vertex smooth; frons smooth; face smooth; temple in dorsal view broad and slightly bulging behind eye, width equal to 1/2 eye width; malar space slightly greater than 1/4 eye height; ocelli small, ocell-ocular distance nearly 5 times diameter of lateral ocellus; 21–24 flagellomeres. Mesosoma: mesoscutal lobes smooth; notauli scrobiculate, meeting posteriorly in triangular costate area; scutellum smooth; prescutellar furrow with 3–5 cross carinae; mesopleuron smooth; precoxal sulcus smooth, shorter than mesopleuron; venter smooth; propodeum with basal median areas margined, weakly granulate, basal median carina present, areola distinctly margined, areolar area rugose, lateral areas rugose apically, smooth basally. Wings: fore wing vein r shorter than vein 3RSa (in holotype vein r is very short and nearly absent), vein 1cu-a beyond vein 1M; hind wing vein SC+R present, vein M+CU shorter than vein 1M. Metasoma: first tergum longitudinally costate, length equal to apical width; second tergum longitudinally costate, costae converging toward midline; anterior transverse groove present but weak, straight; posterior transverse groove weak or absent; third tergum weakly costate basally, smooth apically; terga 4–7 smooth; ovipositor as long as metasomal terga 1 and 2 combined.

#### Holotype female.

Top label (white, printed) - Costa Rica: Guanacaste [;] Est. Pitilla 9km S de Santa [;] Cecilia, 700m, C. Moraga [;] ii.1995, Amarilla #6998 [;] L.N. 329950-380450; second label (red, partially printed and hand written) - HOLOTYPE [;] Heterospilus [;] mars [;] P. Marsh. Deposited in ESUW.

#### Paratypes.

1 ♀, same data as holotype (ESUW). 1 ♀, Costa Rica: San Jose [;] Braulio Carillo N. P. [;] 8.2km E tunnel [;] 14-V-1988 P. Hanson (TAMU).

#### Comments.

The yellow stigma, the extremely small ocelli and large ocell-ocular distance, and the brown flagellum are distinctive for this species.

#### Etymology.

Named for the Roman god Mars.

**Figure 258.**
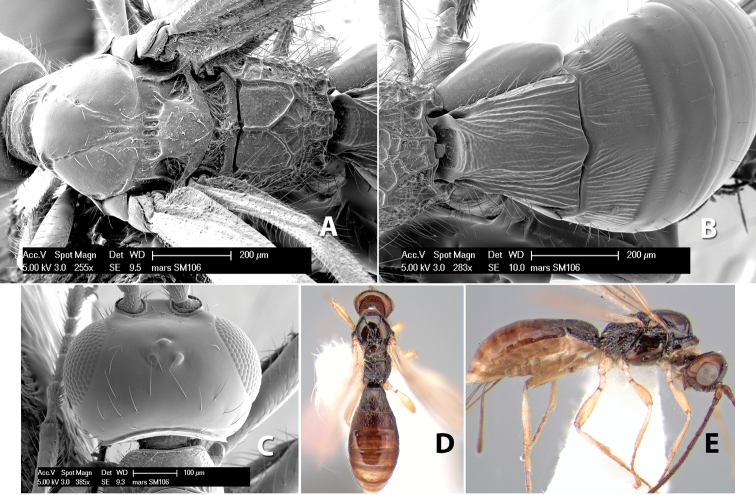
*Heterospilus mars* Marsh, sp. n.: **A–C** paratype **D–E** holotype.

### Heterospilus masneri

Marsh sp. n.

http://zoobank.org/2DBA999F-17E3-4285-AC0E-5F7AF9F4E093

http://species-id.net/wiki/Heterospilus_masneri

Figure 259

#### Female.

Body size: 2.5–3.0 mm. Color: body dark brown, apical metasomal terga lighter brown; scape light brown without lateral brown stripe; flagellum brown with apical white annulus, apical 3–5 flagellomeres brown; wing veins including stigma brown; legs yellow to honey yellow. Head: vertex smooth, rarely with weak granulations near ocelli; frons smooth; face weakly granulate or striate; temple in dorsal view narrow, sloping behind eye, width equal to 1/2 eye width; malar space greater than 1/4 eye height; ocell-ocular distance slightly greater than 2.5 times diameter of lateral ocellus; 20–24 flagellomeres. Mesosoma: mesoscutal lobes weakly, rarely partially smooth; notauli smooth posteriorly, meeting posteriorly in triangular costate area; scutellum smooth; prescutellar furrow with 3–5 cross carinae; mesopleuron granulate; precoxal sulcus smooth, shorter than mesopleuron; venter granulate; propodeum with basal median areas margined, granulate or rarely smooth, basal median carina absent, areola not margined, areolar area areolate-rugose, lateral areas entirely rugose. Wings: fore wing vein r shorter than vein 3RSa, vein 1cu-a slightly beyond or interstitial with vein 1M; hind wing vein SC+R present, vein M+CU shorter than vein 1M. Metasoma: first tergum longitudinally costate, length equal to apical width; second tergum longitudinally costate; anterior transverse groove present, straight; posterior transverse groove present; third tergum costate basally, smooth apically; terga 4–7 smooth; ovipositor as long as metasomal terga 1 and 2 combined or half as long as metasoma.

#### Holotype female.

Top label (white, printed) - COSTA RICA: Puntarenas [;] Reserva Forestal Golfo Dulce [;] 3km SW of Rincon, 10m [;] Mar-April 1992, P. Hanson [;] primary forest, Malaise trap; second label (red, partially printed and hand written) - HOLOTYPE [;] Heterospilus [;] masneri [;] P. Marsh. Deposited in ESUW.

#### Paratypes.

1 ♀, top label - Costa Rica: Guanacaste [;] Santa Rosa National Pk. [;] 300m, Malaise, Ian Gauld [;] 31.i–21.ii.1987; second label - Bosque Humedo [;] mature dry forest [;] high proportion [;] evergreen species [;] sun (ESUW). 3 ♀♀, top labels - Costa Rica: Guanacaste [;] Santa Rosa Natl. Park [;] 300m, ex. Malaise trap [;] Site #: H-3-O, H-2-C and blank [;] Dates: 31.i–21.ii.1987, 6–27.ix.1986 and 21.ii–14.iii.1987 [;] I.D. Gauld & D. Janzen; second labels - [H] open regenerating [;] woodland, 10 years old [;] [O] in clearing, fully [;] isolated pert or day and [C] more or less fully [;] shaded as possible (ESUW). 1 ♀, Costa Rica: Puntarenas [;] R.F. Golfo Dulce, [;] 3km. SW. Rincon, 10m [;] iii.1993 Paul Hanson coll. [;] Malaise, primary forest (ESUW). 1 ♀, Costa Rica, Puntarenas [;] Pen. Osa, 5km N, [;] Puerto Jimenez, 10m [;] I-II-1993 P. Hanson (ESUW). 1 ♀, Costa Rica: Puntarenas [;] R.F. Golfo Dulce, 3km [;] SW Rincon, 10m [;] Malaise-primary forest [;] viii.1991, P. Hanson (ESUW). 1 ♀, Costa Rica: Puntarenas [;] Pen. Osa, Puerto Jimenez [;] 10m, January 1991, full sun, [;] grassy & weedy site [;] P. Hanson, ex. Malaise (ESUW). 1 ♀, COSTA RICA. Prov. Puntarenas. P.N. [;] Corcovado. Sector Tigre. 34m 28 [;] NOV 2002. J. Azofeifa Zuniga. De [;] Golpe. L S 277800 529600 #72455 (INBC).

#### Comments.

The weakly granulate or smooth mesoscutal lobes and the medium length of the ovipositor are distinctive for this species.

#### Etymology.

Named for my long time friend and fellow hymenopterist, Lubomir Masner.

**Figure 259.**
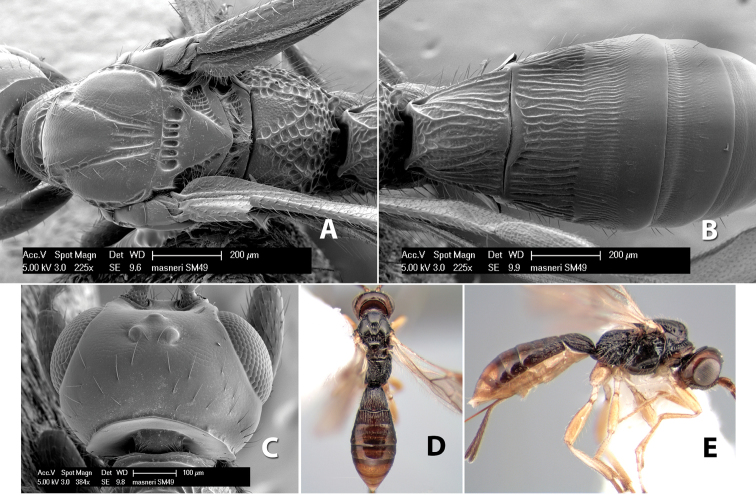
*Heterospilus masneri* Marsh, sp. n.: **A–C** paratype **D–E** holotype.

### Heterospilus mercury

Marsh sp. n.

http://zoobank.org/02B85514-CC53-49BA-B90C-5BFD6DA13497

http://species-id.net/wiki/Heterospilus_mercury

Figure 260

#### Female.

Body size: 2.5 mm. Color: head and mesosoma dark brown, metasoma dark brown with apical terga often lighter; scape light brown; flagellum brown; wing veins brown, stigma yellow; legs yellow. Head: vertex smooth; frons smooth; face smooth; temple in dorsal view narrow, width less than 1/2 eye width; malar space equal to 1/4 eye height; ocell-ocular distance about twice diameter of lateral ocellus; 24–28 flagellomeres. Mesosoma: mesoscutal lobes smooth; notauli scrobiculate, meeting posteriorly in rectangular costate area; scutellum smooth; prescutellar furrow with 1 cross carina; mesopleuron smooth; precoxal sulcus smooth, shorter than mesopleuron; venter smooth; propodeum with basal median areas margined, smooth, basal median carina present, areola not distinctly margined, areolar area rugose, lateral areas entirely rugose. Wings: fore wing vein r shorter than vein 3RSa, vein 1cu-a beyond vein 1M; hind wing vein SC+R present, vein M+CU shorter than vein 1M. Metasoma: first tergum longitudinally costate, length greater than apical width; second tergum longitudinally costate; anterior transverse groove weak or absent; posterior transverse groove weak or absent; third tergum entirely smooth; terga 4–7 smooth; ovipositor as long a metasomal terga 1 and 2 combined.

#### Holotype female.

Top label (white, printed) - Costa Rica: Puntarenas [;] R.F. Golfo Dulce, [;] 3km. SW. Rincon, 10m, [;] x-xii.1990, Paul Hanson; second label (red, partially printed and hand written) - HOLOTYPE [;] Heterospilus [;] mercury [;] P. Marsh. Deposited in ESUW.

#### Paratypes.

1 ♀, same data as holotype except date of ii.1992 (ESUW). 1 ♀, COSTA RICA: [;] Puntarenas [;] Golfo Dulce [;] 15km W. Piedras [;] Blancas, 100m, [;] xii 1990, P. Hanson (ESUW). 1 ♀, Costa Rica: Alajuela, ACA [;] San Carlos, R.F. Arenal [;] Sendero Pilon, 600m, Malaise [;] 26.x–22.xi.1999, G. Carballo [;] L.N. 269100-457900 #54376 (ESUW). 1 ♀, top label - Costa Rica: Limon [;] ACLAC, Central [;] Res. Biol. Hitoy Cerere [;] Est. Hitoy Cerere, 140m; second label - Sendero Toma de Agua [;] 17 Sept. - 10 Oct. 1999 [;] F. Umana, Malaise trap [;] L.N. 184600-643400 #53497 (ESUW). 1 ♀, top label - COSTA RICA, Heredia: [;] Est. Biol. La Selva, 50- [;] 150m, 10°26'N, 84°01'W [;] Oct 1998, INBio-OET; second label - 29 Octubre 1998 [;] Borde suampo [;] M/18/720 (INBC).

#### Comments.

The yellow stigma, smooth mesoscutum and single cross carina in the prescutellar furrow are distinctive for this species.

#### Etymology.

Named for the Roman god Mercury.

**Figure 260.**
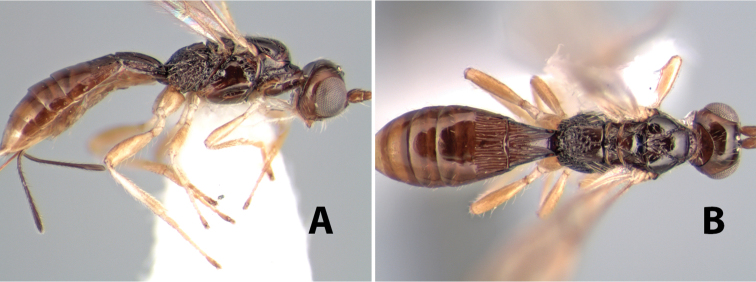
*Heterospilus mercury* Marsh, sp. n., holotype.

### Heterospilus nephilim

Marsh sp. n.

http://zoobank.org/D6CAE569-1379-4FD3-A211-580D9DBF931B

http://species-id.net/wiki/Heterospilus_nephilim

Figure 261

#### Female.

Body size: 5.0–5.25 mm. Color: head and mesosoma dark brown, metasoma honey yellow, tergum 1 often darker medially; scape light brown without lateral brown stripe; flagellum brown; wing veins including stigma brown; legs bicolored, fore and middle legs light brown with lighter trochanters, hind leg dark brown except trochanters light brown. Head: vertex smooth; frons smooth; face smooth, rarely weakly striate below antennal bases; temple in dorsal view broad, bulging behind eye, width greater than 1/2 eye width; malar space slightly greater than 1/4 eye height; ocell-ocular distance slightly greater than 2.5 times diameter of lateral ocellus; 30–33 flagellomeres. Mesosoma: mesoscutal lobes smooth, rarely with some striae near where notauli meet; notauli scrobiculate, meeting posteriorly in triangular costate area; scutellum smooth; prescutellar furrow with 3–5 cross carinae; mesopleuron smooth; precoxal sulcus smooth, shorter than mesopleuron; venter smooth; propodeum with basal median areas not distinctly margined, rugose or partially smooth, basal median carina present, areola not distinct, areolar area rugose, lateral areas rugose apically, smooth basally. Wings: fore wing vein r shorter than vein 3RSa and nearly equal to width of stigma, vein 1cu-a beyond vein 1M; hind wing vein SC+R present, vein M+CU shorter than vein 1M. Metasoma: first tergum longitudinally costate-granulate, distinct cross carinae medially at base between distinct longitudinal carinae, length greater than apical width; second tergum longitudinally costate-granulate; anterior transverse groove present, straight; posterior transverse groove weak or absent; third tergum entirely smooth; terga 4–7 smooth; ovipositor longer than metasoma.

#### Holotype female.

Top label (white, printed) - COSTA RICA, Heredia [;] Est. Biol. La Selva 50- [;] 150m, 10°26N, 84°01W [;] Apr. 1993, INBio-OET; second label (white, printed) - 01 Abril 1993 [;] M/05/052 [;] Bosque primario; third label - INBio bar code; fourth label (red, partially printed and hand written) - HOLOTYPE [;] Heterospilus [;] nephilim [;] P. Marsh. Deposited in ESUW.

#### Paratypes.

7 ♀♀, same data as holotype with additional dates of Mar. 1993 and second label of 15 Marzo 1993 [;] Bosque Primario [;] M/05/036 (ESUW). 1 ♀, COSTA RICA Ala. [;] 20km S Upala [;] 11 Dec. 1990 [;] F. D. Parker (UTAH). 1 ♀, COSTA RICA, Heredia [;] 3km. S. Puerto Viejo [;] OTS-La Selva, 100m [;] ii-iii.1993 P. Hanson (MICR).

#### Comments.

The large size, long ovipositor, dark colored hind legs and the broad and bulging temple are distinctive for this species.

#### Etymology.

The specific name is from the Hebrew, *Nephilim*, which refers to the Biblical giants of the Old Testament and is in reference to the large size of this species.

**Figure 261.**
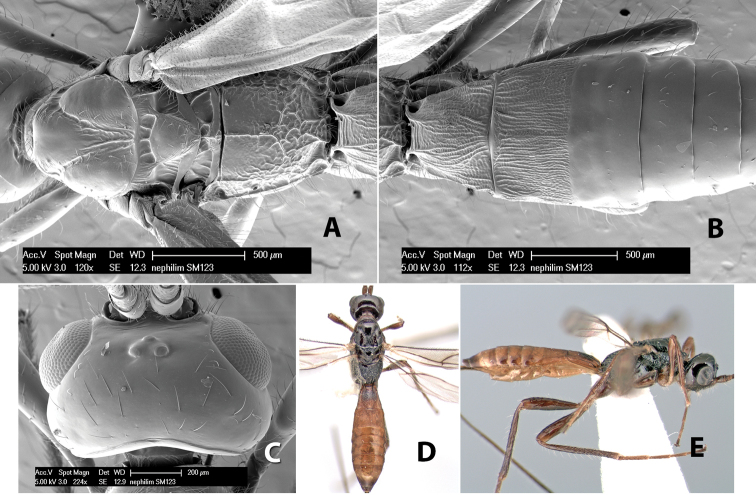
*Heterospilus nephilim* Marsh, sp. n.: **A–C** paratype **D–E** holotype.

### Heterospilus nigracapitus

Marsh sp. n.

http://zoobank.org/530ABC9C-2925-4B8C-95E2-D0D698D02BE4

http://species-id.net/wiki/Heterospilus_nigracapitus

Figure 262

#### Female.

Body size: 2.0–2.5 mm. Color: head dark brown or black, mesosoma and metasoma yellow, mesoscutum and mesopleuron sometimes darker; scape yellow without lateral brown stripe; flagellum brown; legs yellow or light brown. Head: vertex smooth or with few weak striae behind ocelli; frons smooth; face smooth; temple in dorsal view narrow, width equal to 1/2 eye width; malar space greater than 1/4 eye height; ocell-ocular distance greater than 2.5 times diameter of lateral ocellus; 21–22 flagellomeres. Mesosoma: mesoscutal lobes granulate; notauli scrobiculate, meeting posteriorly in triangular costate area; scutellum granulate; prescutellar furrow with 1 distinct median cross carina and 2 lateral weaker carinae; mesopleuron granulate; precoxal sulcus scrobiculate, shorter than mesopleuron; venter granulate; propodeum with basal median areas margined, granulate, basal median carina present, very short, areola indistinctly margined, areolar area rugose, lateral areas entirely rugose. Wings: fore wing vein r shorter than vein 3RSa, vein 1cu-a beyond vein 1M; hind wing vein SC+R present, vein M+CU shorter than vein 1M. Metasoma: first tergum longitudinally costate, length slightly greater than apical width; second tergum longitudinally costate; anterior transverse groove present, straight or very slightly sinuate; posterior transverse groove present; third tergum costate basally, smooth apically; terga 4–7 smooth, often costate at base; ovipositor as long as metasomal terga 1 and 2 combined.

#### Holotype female.

Top label (white, printed) - Costa Rica: Puntarenas [;] Peninsula Osa, Puerto [;] Jimenez, 10m [;] ii-iii.1993, P. Hanson; second label (red, partially printed and hand written) - HOLOTYPE [;] Heterospilus [;] nigracapitus [;] P. Marsh. Deposited in ESUW.

#### Paratypes.

1 ♀, Costa Rica, Puntarenas [;] Pen. Osa, 5km. N. [;] Puerto Jimenez, 10m [;] I-II-1993 P. Hanson (ESUW).

#### Comments.

The dark brown or black head and yellow mesosoma and metasoma and the presence of hind wing vein SC+R are distinctive for this species.

#### Etymology.

The specific name is from the Latin *nigra*, meaning black, and the Latin *capitis*, meaning head, in reference to the dark brown or black head contrasting to the yellow remainder of the body.

**Figure 262.**
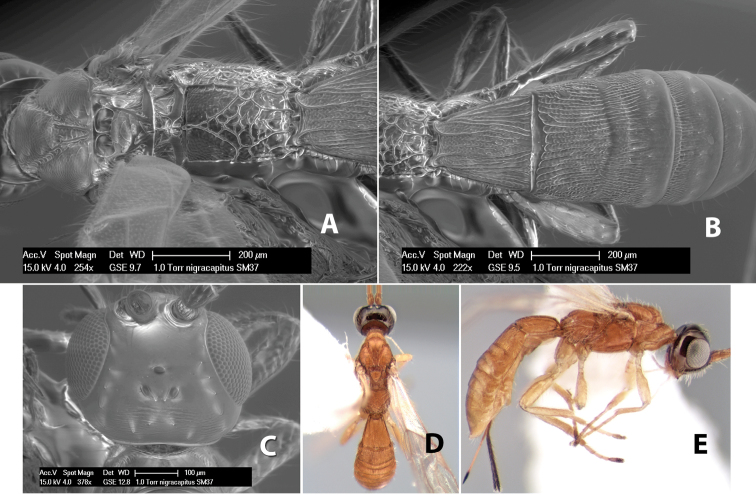
*Heterospilus nigracapitus* Marsh, sp. n., holotype.

### Heterospilus nueve

Marsh sp. n.

http://zoobank.org/5097B1D3-ABC9-41FF-A955-6AC7361D21D8

http://species-id.net/wiki/Heterospilus_nueve

Figure 263

#### Female.

Body size: 2.0 mm. Color: head with vertex and frons brown, face and eye orbits honey yellow; mesosoma dark brown; metasoma dark brown with apical terga lighter brown; scape yellow without lateral brown stripe; flagellum brown; wing veins including stigma brown; legs yellow. Head: vertex smooth; frons smooth; face smooth; temple in dorsal view narrow, sloping behind eye, width less than 1/2 eye width; malar space equal to 1/4 eye height; ocell-ocular distance about 1.5 times diameter of lateral ocellus; 19 flagellomeres. Mesosoma: mesoscutal lobes granulate; notauli scrobiculate, meeting posteriorly in triangular costate-rugose area; scutellum granulate; prescutellar furrow with 1 cross carina; mesopleuron granulate; precoxal sulcus smooth, shorter than mesopleuron; venter weakly granulate or smooth; propodeum with basal median areas margined, granulate, basal median carina present, areola not distinctly margined, areolar area rugose, lateral areas entirely rugose. Wings: fore wing vein r shorter than vein 3RSa, vein 1cu-a slightly beyond vein 1M; hind wing vein SC+R present, vein M+CU shorter than vein 1M. Metasoma: first tergum longitudinally costate, length equal to apical width, median raised area distinctly margined and with cross carina at base; second tergum longitudinally costate; anterior transverse groove present, straight; posterior transverse groove absent; third tergum costate basally, smooth apically; terga 4–7 smooth; ovipositor about 3/4 length of metasoma.

#### Holotype female.

Top label (white, printed) - COSTA RICA-Heredia Prov. [;] La Selva Biological Station [;] 10°26'N, 84°01'W, 100m [;] Canopy fogging 37 [;] 15.xi.1994 [;] Project ALAS (FVK37); second label (red, partially printed and hand written) - HOLOTYPE [;] Heterospilus [;] nueve [;] P. Marsh. Deposited in ESUW.

#### Paratypes.

1 ♀, COSTA RICA, Limon [;] sur de Iriquois [;] 300m, 23/V/1987 [;] Col. Paul Hanson (MICR). 1 ♀, top label - COSTA RICA, Heredia: [;] Est. Biol. La Selva, 50- [;] 150m, 10°26'N, 84°01'W [;] Aug 1998, INBio-OET; second label - 06 Agosto 1998 [;] Borde suampo [;] M/18/714 (INBC).

#### Comments.

The absent posterior transverse groove of metasomal tergum 3, the distinct median basal carina of the propodeum and the brown flagellum are distinctive for this species.

#### Etymology.

The specific name is an arbitrary combination of letters.

**Figure 263.**
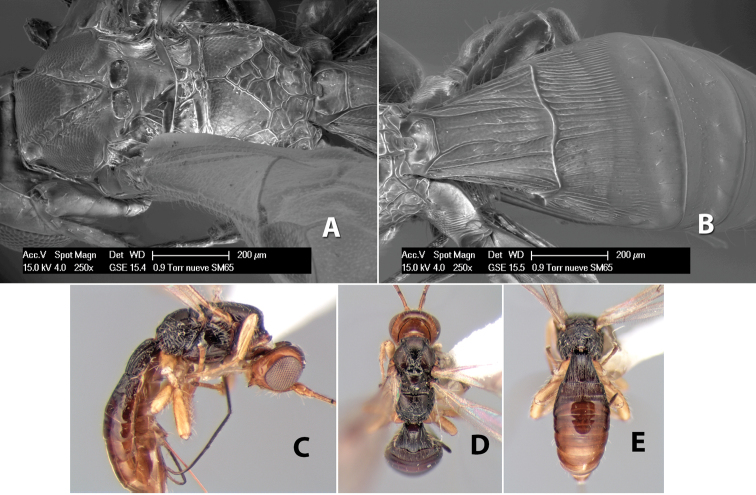
*Heterospilus nueve* Marsh, sp. n., holotype.

### Heterospilus nunesi

Marsh sp. n.

http://zoobank.org/0E911C02-4904-4CE7-A0CE-C271B5EC14F6

http://species-id.net/wiki/Heterospilus_nunesi

Figure 264

#### Female.

Body size: 2.5–3.0 mm. Color: body dark brown, apical metasomal terga usually yellow or light brown; scape yellow without lateral brown stripe; flagellum brown; wing veins brown, stigma light brown or yellow; legs yellow. Head: vertex smooth; frons smooth; face smooth; temple in dorsal view narrow, sloping behind eye, width equal to eye width; malar space greater than 1/4 eye height; ocell-ocular distance slightly greater than 2.5 times diameter of lateral ocellus; 20–25 flagellomeres. Mesosoma: mesoscutal lobes weakly granulate; notauli scrobiculate, meeting posteriorly in triangular costate area; scutellum smooth; prescutellar furrow with 3–5 cross carinae; mesopleuron weakly granulate; precoxal sulcus smooth weakly scrobiculate or smooth, shorter than mesopleuron; venter smooth; propodeum with basal median areas margined, granulate, basal median carina absent or very short, areola distinctly margined, areolar area rugose, lateral areas entirely rugose. Wings: fore wing vein r shorter than vein 3RSa, vein 1cu-a slightly beyond or interstitial with vein 1M; hind wing vein SC+R present, vein M+CU shorter than 1M. Metasoma: first tergum longitudinally costate, length greater than apical width; second tergum longitudinally costate; anterior transverse groove present, straight; posterior transverse groove present; third tergum costate basally, smooth apically; terga 4–7 smooth; ovipositor half as long as metasoma.

#### Holotype female.

Top label (white, partially printed and hand written) - COSTA RICA, Heredia [;] Chilamate, 75m [;] 25-II-1989 [;] Col. P. Hanson; second label (red, partially printed and hand written) - HOLOTYPE [;] Heterospilus [;] nunesi [;] P. Marsh. Deposited in ESUW.

#### Paratypes.

1 ♀, same data as holotype (ESUW). 1 ♀, Costa Rica: Heredia [;] Est. Biol. La Selva [;] 50–150m, 10.26 N [;] 84.01 W, Aug. 1992 (ESUW). 1 ♀, Costa Rica: Heredia [;] 3km. S. Puerto Viejo [;] OTS - La Selva, 100m [;] 16–30 IX.1992 [;] P. Hanson (ESUW). 1 ♀, Costa Rica, Heredia Prov. [;] OTS, La Selva, 100m [;] 1993 II-III P. Hanson (ESUW). 1 ♀, COSTA RICA-Heredia Prov. [;] La Selva Biological Station [;] 10°26'N, 84°01W 100m [;] Malaise trap 01, #248 [;] 1.xi.1993 [;] Project ALAS (M.01.248) (ESUW). 1 ♀, Costa Rica: Heredia [;] 3Km. S. Puerto Viejo [;] OTS - La Selva, 100m [;] v-vi.1993, P. Hanson (ESUW). 1 ♀, COSTA RICA: [;] Guanacaste Prov. [;] Cerro el Hacha [;] NW Volcan Orosi [;] 300m, 1988 (ESUW). 1 ♀, Costa Rica: Puntarenas [;] Pen. Osa, Puerto Jimenez [;] 10m, August 1991, full sun, [;] grassy & weedy site [;] P. Hanson, ex Malaise (ESUW). 1 ♀, top label - Costa Rica: Puntarenas [;] A.C.O., Golfito, Reserva [;] Forestal Golfo Dulce [;] Est. Agujas, 250–350m; second label - 2–22 October 1999 [;] J. Azofeifa, Red de Golpe [;] L-S-276750-526550 #5349 (ESUW). 4 ♀♀, Costa Rica: Puntarenas ACO [;] Golfito, R.F. Golfo Dulce [;] Est Agujas, 250–350m [;] 3–23.ix.1999 and 4–22.v.1999, J. Azofeifa [;] L.S. 276750-526550 #53269 [;] Red de Golpe (ESUW). 1 ♀, Costa Rica: Puntarenas, ACO [;] Golfito, R.F. Golfo Dulce [;] Est. Agujas, 250–350m [;] 1–11.xi.1999, J. Azofeifa [;] Red.de Golpe #54023 [;] L.S.276750-526550 (ESUW). 1 ♀, COSTA RICA, Heredia [;] Chilamate, 75m [;] 25/III/1989 [;] col. Hanson & Godoy (MICR). 2 ♀♀, Costa Rica: Puntarenas [;] San Vito - Las Cruces [;] 5-VI-1988 1200m [;] P. Hanson (TAMU). 3 ♀♀, Costa Rica: San Jose [;] Braulio Carillo N. P. [;] 8.2km E tunnel [;] 15-V-1988 (TAMU). 4 ♀♀, COSTA RICA, Prov. Puntarenas [;] Est. Agujas, Frente a la Estación, [;] 300m, 19–21 MAR 1997. M. Lobo. [;] Red de Golpe. [;] L_S_276750_536550 #45572 (INBC). 1 ♀, CPOSTA RICA, Prov. Puntarenas, [;] Est. Agujas, Send. Ajo. 300m. [;] 3–7 DIC1997. M. Lobo. Red de [;] Golpe. L_S_276750_526550 [;] #48755 (INBC). 2 ♀♀, COSTA RICA. Prov. Puntarenas. P.N. [;] Corcovado, Sector Tigre. 34m. 28 [;] NOV 2002. J. Azofeifa Zuniga. De [;] Golpe. L.S. 277800 529600 #72455 (INBC). 2 ♀♀, Turrialba, C.R. [;] IV-17, 21-1957 [;] RDShenefelt [;] RDS 57-68, 124 (AEIC). 1 ♀, LaLola,C.R. [;] VI-8 1957 [;] MJStelzer [;] [;] MS 57-254 (AEIC).

#### Comments.

The dark brown body, brown flagellum and presence of hind wing vein SC+R are distinctive for this species.

#### Etymology.

Named for Juliano F. Nunes in recognition of his studies of Brazilian Doryctinae.

**Figure 264.**
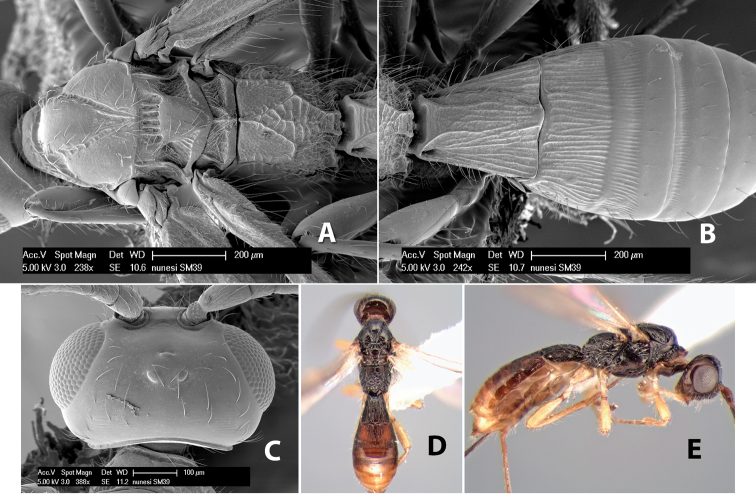
*Heterospilus nunesi* Marsh, sp. n.: **A–C** paratype **D–E** holotype.

### Heterospilus once

Marsh sp. n.

http://zoobank.org/57673638-9C37-44C8-AA71-C65DCA0F64A2

http://species-id.net/wiki/Heterospilus_once

Figure 265

#### Female.

Body size: 2.5 mm. Color: body dark brown, weakly sclerotized areas at base of metasomal terga 3–5 often white; scape yellow without lateral brown stripe; flagellum brown; wing veins including stigma brown; legs yellow. Head: vertex smooth; frons smooth; face smooth; temple in dorsal view sloping behind eye, width less than 1/2 eye width; malar space greater than 1/4 eye height; ocell-ocular distance greater than 2.5 times diameter of lateral ocellus; 20 flagellomeres. Mesosoma: mesoscutal lobes smooth; notauli smooth, meeting posteriorly in triangular rugose area; scutellum smooth; prescutellar furrow with 1 cross carina; mesopleuron smooth; precoxal sulcus smooth, shorter than mesopleuron; venter smooth; propodeum with basal median areas margined, smooth, basal median carina present, areola distinctly margined, areolar area smooth, lateral areas rugose apically, smooth basally. Wings: fore wing vein r shorter than vein 3RSa, vein 1cu-a interstitial with vein 1M; hind wing vein SC+R present, vein M+CU shorter than vein 1M. Metasoma: first tergum longitudinally costate-granulate, length nearly twice apical width, median raised area distinctly margined; second tergum longitudinally costate-granulate, apical border raised and smooth, length about 4 times width; anterior transverse groove present, straight; posterior transverse groove weak or absent; third tergum smooth with basal weakly sclerotized area which is often lighter colored; terga 4–5 smooth with basal weakly sclerotized area which is often lighter colored; terga 6–7 smooth; ovipositor about 3/4 length of metasoma.

#### Holotype female.

Top label (white, printed) - Costa Rica: San Jose [;] 26km. N. San Isidro [;] just S. of Division [;] 2100m, vi-viii.1992 [;] P. Hanson, Malaise [;] secondary growth; second label (red, partially printed and hand written) - HOLOTYPE [;] Heterospilus [;] once [;] P. Marsh. Deposited in ESUW.

#### Paratypes.

2 ♀♀, same data as holotype (ESUW). 1 ♀, COSTTA RICA: San Jose, [;] Cerro de la Muerte, [;] 26km N San Isidro, 2100m, [;] ii-v 1992 [;] Paul Hanson (ESUW).

#### Comments.

The weakly sclerotized basal areas of metasomal terga 3–5 are distinctive for this species. It is similar to *Heterospilus hansonorum* but is distinguished by the brown flagellum.

#### Etymology.

The specific name is an arbitrary combination of letters.

**Figure 265.**
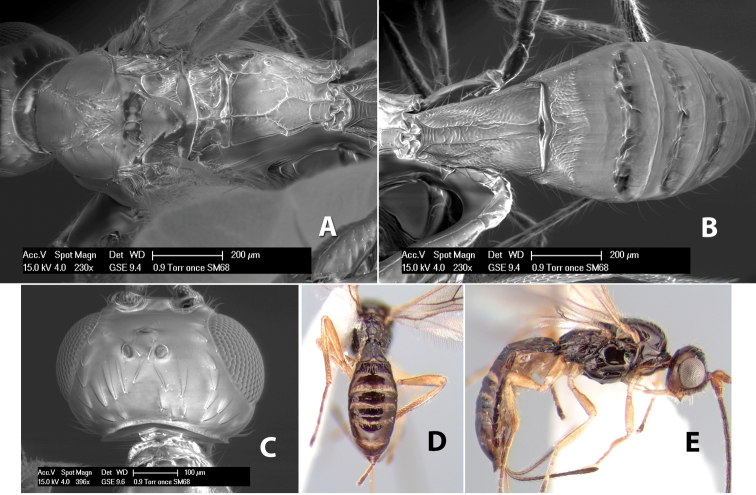
*Heterospilus once* Marsh, sp. n., holotype.

### Heterospilus parvus

Marsh sp. n.

http://zoobank.org/7A7BD095-C4CE-446D-8B53-14DFD847CE11

http://species-id.net/wiki/Heterospilus_parvus

Figures 266 , 267

#### Female.

Body size: 1.5–2.0 mm. Color: head and mesosoma light to medium brown, metasomal terga 1 and 2 usually yellow, terga 3–7 usually light brown, metasoma rarely entirely yellow; scape yellow without lateral brown stripe; flagellum brown, basal flagellomeres sometimes yellow; wing veins including stigma brown; legs yellow. Head: vertex smooth; frons smooth; face smooth; temple in dorsal view narrow but sloping behind eye, width equal to 1/2 eye width; malar space greater than 1/4 eye height; ocell-ocular distance greater than 2.5 times diameter of lateral ocellus; 14–17 flagellomeres. Mesosoma: mesoscutal lobes granulate; notauli scrobiculate, meeting at scutellum in triangular rugose area; scutellum weakly granulate or smooth; prescutellar furrow with 1 cross carina; mesopleuron granulate; precoxal sulcus smooth, shorter than mesopleuron, often with weak striae extending to posterior margin of mesopleuron; venter granulate; propodeum with basal median areas margined, granulate, basal median carina present and very short, rarely absent so areola meets basal margin of propodeum, areola not distinctly margined, areolar area rugose, lateral areas entirely rugose. Wings: fore wing vein r equal to or slightly shorter than vein 3RSa, vein 1cu-a interstitial with vein 1M; hind wing vein SC+R absent, vein M+CU shorter than vein 1M. Metasoma: first tergum weakly longitudinally costate, length equal to apical width; second tergum weakly longitudinally costate; anterior transverse groove present, straight; posterior transverse groove present; third tergum entirely smooth; terga 4–7 smooth; ovipositor equal to length of metasomal terga 1 and 2 combined.

#### Holotype female.

Top label (white, partially printed and hand written) - Costa Rica: Guanacaste [;] Santa Rosa National Pk. [;] 300m, Malaise, Ian Gauld [;] 31.i–21.ii.1987; second label (white, partially printed and hand written) - Bosque Humedo [;] Mature dry forest [;] high proportion [;] Evergreen species [;] Sun; third label (white, printed) - BH-9-O [;] 31.i–21.ii.87; fourth label (red, partially printed and hand written) - HOLOTYPE [;] Heterospilus [;] parvus [;] P. Marsh. Deposited in ESUW.

#### Paratypes.

19 ♀♀, same data as holotype with additional dates of 31.i–4.ii.1987 and 10–31.i.1987 (ESUW). 6 ♀♀, top label - Costa Rica: Guanacaste [;] Santa Rosa Natl. Park [;] 300m, ex. Malaise trap [;] Site #: BH-11-O [;] Dates: 8.ii–2.iii.1986 [;] I.D. Gauld & D. Janzen; second label - [BH] Bosque Humedo [;] mature evergreen dry forest [;] [O] in clearing, fully [;] isolated part of day (ESUW). 4 ♀♀, top label - Costa Rica: Guanacaste [;] Santa Rosa Natl. Park [;] 300m, ex. Malaise trap [;] Site #: BH-9-O [;] Dates: 20.xi.86–10.i.1987 [;] I.D. Gauld & D. Janzen; second label - [BH] Bosque Humedo [;] mature evergreen dry forest [;] [O] in clearing, fully [;] isolated part of day (ESUW). 5 ♀♀, top label - Costa Rica: Guanacaste [;] Santa Rosa Natl. Park [;] 300m, ex. Malaise trap [;] Site #: BH-11-O or blank [;] Dates: 18.i–8.ii.1986 [;] I.D. Gauld & D. Janzen; second label - [BH] Bosque Humedo [;] mature evergreen dry forest [;] [O] in clearing, fully [;] isolated part of day (ESUW). 2 ♀♀, top label - Costa Rica: Guanacaste [;] Santa Rosa Natl. Park [;] 300m, ex. Malaise trap [;] Site #: blank [;] Dates: 28.xii.85–18.i.1986 [;] I.D. Gauld & D. Janzen; second label - [BH] Bosque Humedo [;] mature evergreen dry forest [;] [O] in clearing, fully [;] isolated part of day (ESUW). 2 ♀♀, top label - Costa Rica: Guanacaste [;] Santa Rosa Natl. Park [;] 300m, ex. Malaise trap [;] Site #: blank [;] Dates: 2–23.iii.1986 [;] I.D. Gauld & D. Janzen; second label - [BH] Bosque Humedo [;] mature evergreen dry forest [;] [O] in clearing, fully [;] isolated part of day (ESUW). 1 ♀, top label - Costa Rica: Guanacaste [;] Santa Rosa Natl. Park [;] 300m, ex. Malaise trap [;] Site #: BH-10-C [;] Dates: 16.xi–7.xii.1985 [;] I.D. Gauld & D. Janzen; second label - [BH] Bosque Humedo [;] mature evergreen dry forest [;] [C] more or less fully [;] shaded as possible (ESUW). 1 ♀, top label - Costa Rica: Guanacaste [;] Santa Rosa Natl. Park [;] 300m, ex. Malaise trap [;] Site #: BH-12-C [;] Dates: 18.i–8.ii.1986 [;] I.D. Gauld & D. Janzen; second label - [BH] Bosque Humedo [;] mature evergreen dry forest [;] [C] more or less fully [;] shaded as possible (ESUW). 1 ♀, top label - Costa Rica: Guanacaste [;] Santa Rosa Natl. Park [;] 300m, ex. Malaise trap [;] Site #: BH-12-C [;] Dates: 16.xi–7.xii.1985 [;] I.D. Gauld & D. Janzen; second label - [BH] Bosque Humedo [;] mature evergreen dry forest [;] [C] more or less fully [;] shaded as possible (ESUW). 1 ♀, top label - Costa Rica: Guanacaste [;] Santa Rosa Natl. Park [;] 300m, ex. Malaise trap [;] Site #: blank [;] Dates: 14.vi–5.vii.1986 [;] I.D. Gauld & D. Janzen; second label - [BH] Bosque Humedo [;] mature evergreen dry forest [;] [C] more or less fully [;] shaded as possible (ESUW). 2 ♀♀, top label - Costa Rica: Guanacaste [;] Santa Rosa Natl. Park [;] 300m, ex. Malaise trap [;] Site #: BH-10-C [;] Dates: 8.ii–2.iii.1986 [;] I.D. Gauld & D. Janzen; second label - [BH] Bosque Humedo [;] mature evergreen dry forest [;] [C] more or less fully [;] shaded as possible (ESUW). 2 ♀♀, top label - Costa Rica: Guanacaste [;] Santa Rosa Natl. Park [;] 300m, ex. Malaise trap [;] Site #: blank [;] Dates: 7–28.xii.1985 [;] I.D. Gauld & D. Janzen; second label - [SE] Bosque San Emilio [;] 50yr old deciduous forest [;] [C] more or less fully [;] shaded as possible (ESUW). 1 ♀, top label - Costa Rica: Guanacaste [;] Santa Rosa Natl. Park [;] 300m, ex. Malaise trap [;] Site #: BH-9-O [;] Dates: 2–23.iii.1986 [;] I.D. Gauld & D. Janzen; second label - [SE] Bosque San Emilio [;] 50yr old deciduous forest [;] [C] more or less fully [;] shaded as possible (ESUW). 1 ♀, top label - Costa Rica: Guanacaste [;] Santa Rosa Natl. Park [;] 300m, ex. Malaise trap [;] Site #: blank [;] Dates: 31.i–21.ii.1987 [;] I.D. Gauld & D. Janzen; second label - [SE] Bosque San Emilio [;] 50yr old deciduous forest [;] [C] more or less fully [;] shaded as possible (ESUW). 2 ♀♀, top label - Costa Rica: Guanacaste [;] Santa Rosa Natl. Park [;] 300m, ex. Malaise trap [;] Site #: 8 [;] Dates: 23.iii–13.iv.1986 [;] I.D. Gauld & D. Janzen; second label - [SE] Bosque San Emilio [;] 50yr old deciduous forest [;] [C] more or less fully [;] shaded as possible (ESUW). 3 ♀♀, top label - Costa Rica: Guanacaste [;] Santa Rosa Natl. Park [;] 300m, ex. Malaise trap [;] Site #: blank [;] Dates: 18.i–8.ii.1986 [;] I.D. Gauld & D. Janzen; second label - [SE] Bosque San Emilio [;] 50yr old deciduous forest [;] [C] more or less fully [;] shaded as possible (ESUW). 1 ♀, top label - Costa Rica: Guanacaste [;] Santa Rosa Natl. Park [;] 300m, ex. Malaise trap [;] Site #: 6 [;] Dates: 8–26.x.1985 [;] I.D. Gauld & D. Janzen; second label - [SE] Bosque San Emilio [;] 50yr old deciduous forest [;] [C] more or less fully [;] shaded as possible (ESUW). 1 ♀, top label - Costa Rica: Guanacaste [;] Santa Rosa Natl. Park [;] 300m, ex. Malaise trap [;] Site #: SE-6-C [;] Dates: 20.xii.86–10.i.1987 [;] I.D. Gauld & D. Janzen; second label - [SE] Bosque San Emilio [;] 50yr old deciduous forest [;] [C] more or less fully [;] shaded as possible (ESUW). 2 ♀♀, top label - Costa Rica: Guanacaste [;] Santa Rosa Natl. Park [;] 300m, ex. Malaise trap [;] Site #: H-3-O [;] Dates: 10–31.i.1987 [;] I.D. Gauld & D. Janzen; second label - [H] open regenerating woodland <10 years old [;] [O] in clearing, fully [;] isolated part of day (ESUW). 1 ♀, top label - Costa Rica: Guanacaste [;] Santa Rosa Natl. Park [;] 300m, ex. Malaise trap [;] Site #: blank [;] Dates: 10–31.i.1987 [;] I.D. Gauld & D. Janzen; second label - [H] open regenerating woodland <10 years old [;] [C] more or less fully [;] shaded as possible (ESUW). 1 ♀, top label - Costa Rica: Guanacaste [;] Santa Rosa Natl. Park [;] 300m, ex. Malaise trap [;] Site #: SE-5-O [;] Dates: 31.i–21.ii.1987 [;] I.D. Gauld & D. Janzen; second label - [SE] Bosque San Emilio [;] 50yr old deciduous forest [;] [O] in clearing, fully [;] isolated part of day (ESUW). 1 ♀, top label - Costa Rica: Guanacaste [;] Santa Rosa Natl. Park [;] 300m, ex. Malaise trap [;] Site #: SE-7-O [;] Dates: 7–28.xii.1985 [;] I.D. Gauld & D. Janzen; second label - [SE] Bosque San Emilio [;] 50yr old deciduous forest [;] [O] in clearing, fully [;] isolated part of day (ESUW). 1 ♀, top label - Costa Rica: Guanacaste [;] Santa Rosa Natl. Park [;] 300m, ex. Malaise trap [;] Site #: SE-5-O [;] Dates: 26.vii.1986 [;] I.D. Gauld & D. Janzen; second label - [SE] Bosque San Emilio [;] 50yr old deciduous forest [;] [O] in clearing, fully [;] isolated part of day (ESUW). 2 ♀♀, top label - Costa Rica: Guanacaste [;] Santa Rosa Natl. Park [;] 300m, ex. Malaise trap [;] Site #: SE-7-O [;] Dates: 10–31.i.1987 [;] I.D. Gauld & D. Janzen; second label - [SE] Bosque San Emilio [;] 50yr old deciduous forest [;] [O] in clearing, fully [;] isolated part of day (ESUW). 2 ♀♀, top label - Costa Rica: Guanacaste [;] Santa Rosa Natl. Park [;] 300m, ex. Malaise trap [;] Site #: blank [;] Dates: 29.xi–20.xii.1986 [;] I.D. Gauld & D. Janzen; second label - [SE] Bosque San Emilio [;] 50yr old deciduous forest [;] [O] in clearing, fully [;] isolated part of day (ESUW). 1 ♀, top label - Costa Rica: Guanacaste, Santa [;] Rosa Nat’l Park, Bosque San [;] Emilio, trap #5 in clearing, 300m. [;] XII/28/85-I/18/1986, I. Gauld; second label - [SE] Bosque San Emilio [;] 50yr old deciduous forest [;] [O] in clearing, fully [;] isolated part of day (ESUW). 1 ♀ Costa Rica: Guanacaste Pr. [;] Guanacaste National Park [;] near Headquarters [;] 1–10 March 1990, J.S. Noyes (ESUW). 3 ♀♀, COSTA RICA: [;] San Jose [;] Ciudad Colon, 800m [;] xii 1990 and iv-v 1990, Luis Fournier (ESUW). 1 ♀, COSTA RICA: Alajuela [;] San Pedro de la [;] Tigra Cacao, 200m [;] I-II 1990 R, Cespedes (ESUW). 1♀, Costa Rica, Carthago Pr. [;] Dulce Nombre, Vivero [;] Linda Vista, 1300m [;] 1993: viii-x, P. Hanson (ESUW). 2 ♀♀, COSTA RICA: Puntar [;] Golfo Dulce 3km SW [;] Rincon [;] 10m, xii 1989-iii 1990 [;] Col. Paul Hanson (ESUW). 2 ♀♀, Costa Rica: Puntarenas [;] Pen. Osa, Puerto Jimenez [;] 10m, August 1991, full sun, [;] grassy & weedy site [;] P. Hanson, ex. Malaise (ESUW). 1 ♀, COSTA RICA, SanJosé [;] Ciudad Colón, 800m [;] Hdq. Rodeo, 14.ix.91 [;] col. Paul Hanson (MICR). 1 ♀, COSTA RICA, SanJosé [;] Ciudad Colón, 800m [;] II 1990 [;] Col. Luis Fournier (MICR). 8 ♀♀, S.RosaPark, Guan. [;] C. Rica various dates 4 Dec., 76 to 8 Jan 78 [;] D.H. Janzen [;] Dry Hill and Riparian (AEIC).

#### Comments.

The small size, yellow metasomal terga 1 and 2 and brown flagellum are distinctive for this species.

#### Etymology.

The specific name is from the Latin *parvus*, meaning little, in reference to the small size of this species.

**Figure 266.**
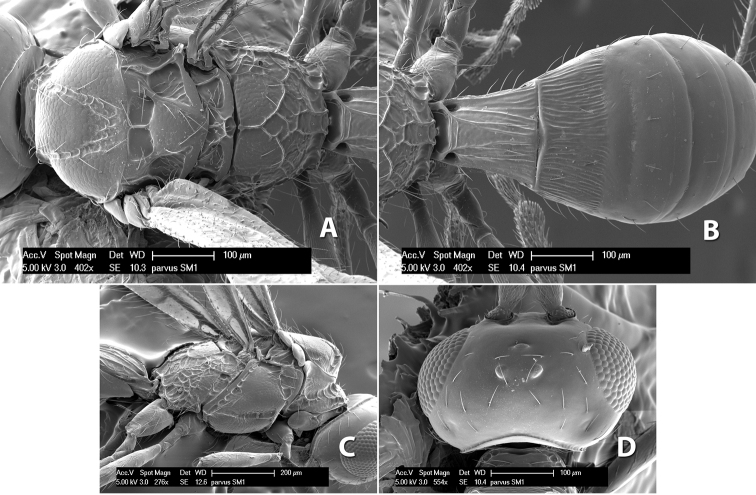
*Heterospilus parvus* Marsh, sp. n., paratype.

**Figure 267.**
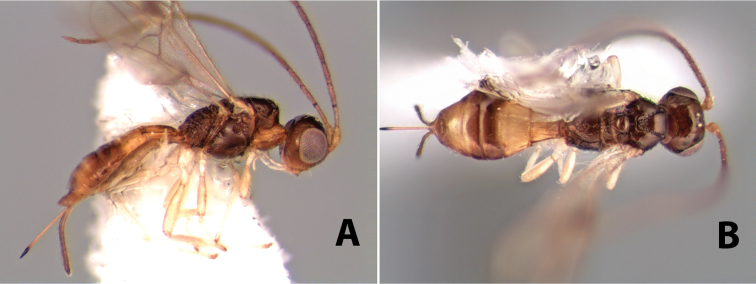
*Heterospilus parvus* Marsh, sp. n., holotype.

### Heterospilus penosa

Marsh sp. n.

http://zoobank.org/C1782CAD-5F40-46B1-97EC-A7378113FFF8

http://species-id.net/wiki/Heterospilus_penosa

Figure 268

#### Female.

Body size: 2.0–2.5 mm. Color: head and mesosoma medium to dark brown, propleuron usually yellow, metasomal terga yellow, tergum 1 usually slightly darker; scape yellow without lateral brown stripe, flagellum brown; wing veins brown, stigma yellow; legs yellow. Head: vertex smooth; frons smooth; face smooth; temple in dorsal view broad but sloping behind eye, width equal to 1/2 eye width; malar space greater than 1/4 eye height; ocell-ocular distance greater than 2.5 times diameter of lateral ocellus; 16–21 flagellomeres. Mesosoma: mesoscutal lobes smooth; notauli smooth, meeting posteriorly in small triangular rugose area; scutellum smooth; prescutellar furrow with 3 cross carinae; mesopleuron smooth; precoxal sulcus smooth, shorter than mesopleuron; venter smooth; propodeum with basal median areas margined, granulate, basal median carina extremely short or appearing absent, areola not margined, areolar area rugose, lateral areas entirely rugose. Wings: fore wing vein r shorter than vein 3RSa, vein 1cu-a interstitial with vein 1M; hind wing vein SC+R absent, vein M+CU shorter than vein 1M. Metasoma: first tergum longitudinally costate, length nearly twice apical width; second tergum longitudinally costate; anterior transverse groove very weak or absent; posterior transverse groove very weak or absent; third tergum entirely smooth; terga 4–7 smooth; ovipositor as long as metasomal terga 1 and 2 combined.

#### Holotype female.

Top label (white, printed) - Costa Rica: Puntarenas [;] Pen. Osa, Puerto Jimenez [;] 10m, September 1991, full [;] sun, grassy & weedy site [;] P. Hanson, ex. Malaise; second label (red, partially printed and hand written) - HOLOTYPE [;] Heterospilus [;] penosa [;] P. Marsh. Deposited in ESUW.

#### Paratypes.

1 ♀, Costa Rica: Puntarenas, ACO [;] Golfito, R.F. Golfo Dulce [;] Est. Agujas, 250–350m [;] 1–11.xi.1999, J. Azofeifa [;] Red de Golpe #54023 [;] L.S. 276750-526550 (ESUW).

#### Comments.

The long and narrow metasomal tergum 1, extremely short or absent basal median carina on the propodeum, the yellow stigma and the smooth mesoscutum and mesopleuron are distinctive for this species.

#### Etymology.

The specific name refers to the type series being collected on or in the vicinity of the Osa Peninsula in Puntarenas Province.

**Figure 268.**
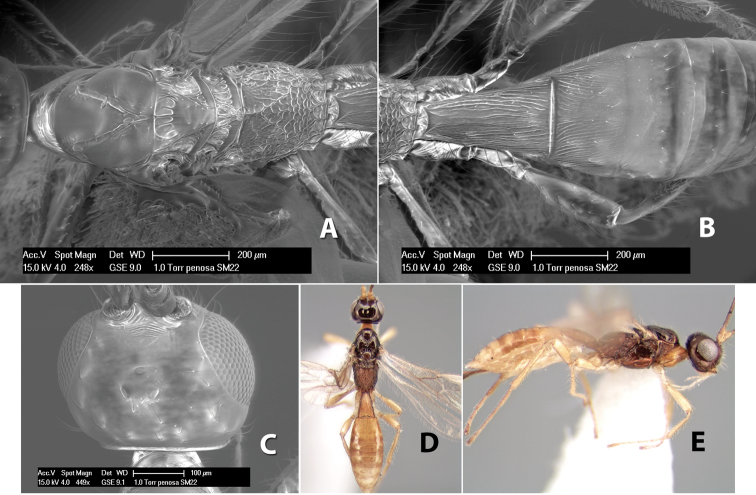
*Heterospilus penosa* Marsh, sp. n., holotype.

### Heterospilus petiolatus

Marsh sp. n.

http://zoobank.org/027C37C8-6E5E-41E7-AE63-9ABD3AA331CA

http://species-id.net/wiki/Heterospilus_petiolatus

Figure 269

#### Female.

Body size: 3.0–3.5 mm. Color: head dark brown; mesosoma dark brown with propodeum usually lighter brown; metasomal terga 1, 2 and base of 3 brown to light brown, apex of 3 and remainder of terga yellow; scape yellow without lateral brown stripe, flagellum brown; wing veins brown, stigma yellow; legs yellow. Head: vertex smooth; frons smooth; face smooth; temple in dorsal view broad but sloping behind eye, width equal to 1/2 eye width; malar space equal to 1/4 eye height; ocell-ocular distance about 2.5 times diameter of lateral ocellus; 23–27 flagellomeres. Mesosoma: mesoscutal lobes smooth; notauli weakly scrobiculate, meeting posteriorly in triangular area with 2 longitudinal costae; scutellum smooth; prescutellar furrow with 3 cross carinae; mesopleuron smooth; precoxal sulcus smooth, shorter than mesopleuron; venter smooth; propodeum with basal median areas margined, smooth, basal median carina present, areola not margined, areolar area areolate-rugose, lateral areas entirely rugose. Wings: fore wing vein r shorter than vein 3RSa, vein 1cu-a interstitial with vein 1M; hind wing vein SC+R absent, vein M+CU as long as vein 1M. Metasoma: first tergum longitudinally costate, length about twice apical width; second tergum longitudinally costate; anterior transverse groove present, straight; posterior transverse groove present; third tergum weakly costate basally, smooth apically; terga 4–7 smooth; ovipositor half as long as metasoma.

#### Holotype female.

Top label (white, printed) - COSTA RICA, Limon [;] sur de Iriquois [;] 200m, 23/V/1987 [;] Col. Paul Hanson; second label (red, partially printed and hand written) - HOLOTYPE [;] Heterospilus [;] petiolatus [;] P. Marsh. Deposited in ESUW.

#### Paratypes.

3 ♀♀, 1 ♂, same data as holotype (ESUW). 1 ♀, Costa Rica: Heredia [;] 3km. S. Puerto Viejo [;] OTS - La Selva, 100m [;] 16–30 IX.1992 [;] P. Hanson (ESUW). 1 ♀, Costa Rica: Puntarenas [;] Peninsula Osa, 10 meters [;] 5km NW Puerto Jimenez, [;] viii-ix.1991, Paul Hanson [;] abandoned cacao orchard (ESUW). 1 ♀, COSTA RICA, Alajuela [;] Finca La Selva [;] NE Dos Rios, 400m [;] 27/III/88, Col. Hanson (ESUW). 1 ♀, Costa Rica, Puntarenas [;] Pen. Osa, 5km. N. [;] Puerto Jimenez, 10m [;] I-II-1993 P. Hanson (ESUW). 3 ♀♀, COSTA RICA, Limon [;] sur de Iriquois [;] 300m, 23/V/1987 [;] Col. Paul Hanson (MICR). 1 ♀, COSTA RICA, SanJosé [;] Cerro Muerte, 20km S [;] Empalme. 2800m [;] IX-X 1989 Hanson (MICR). 1 ♀, COSTA RICA, SanJosé [;] P.N. Braulio Carillo [;] 9.5km E tunnel, 1000m [;] V-VI-90 P. Hanson (MICR). 1 ♀, Costa Rica: Limon [;] Cahuita Natl Park [;] 29-V-1988 P. Hanson (YAMU). 1 ♀, top label - LaLola,C.R. [;] VI-22 1957 [;] MJStelzer [;] MS-57-352; second label - On cacao (AEIC).

#### Comments.

The long and narrow metasomal tergum 1 and the smooth mesosoma are distinctive for this species.

#### Etymology.

The specific name is in reference to the long and narrow petiolate metasomal tergum 1.

**Figure 269.**
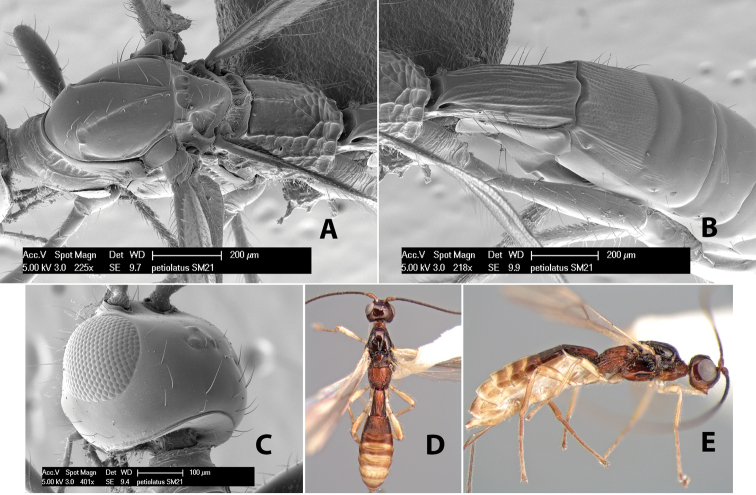
*Heterospilus petiolatus* Marsh, sp. n.: **A–C** paratype **D–E** holotype.

### Heterospilus phaeoskelus

Marsh sp. n.

http://zoobank.org/976BD1A3-F3B1-4C88-8F98-756B582268C7

http://species-id.net/wiki/Heterospilus_phaeoskelus

Figure 270

#### Female.

Body size: 3.0 mm. Color: body dark brown, apical metasomal terga lighter brown; scape light brown without lateral brown stripe; wing veins including stigma brown; legs light brown or honey yellow. Head: vertex smooth; frons smooth; face smooth; temple in dorsal view broad but sloping behind eye, width equal to 1/2 eye width; malar space greater than 1/4 eye height; ocell-ocular distance slightly greater than 2.5 times diameter of lateral ocellus; 23–25 flagellomeres. Mesosoma: mesoscutal lobes granulate; notauli scrobiculate, meeting posteriorly in rectangular costate area; scutellum smooth; prescutellar furrow with 3–5 cross carinae; mesopleuron smooth at least above precoxal sulcus, often granulate or costate dorsally; precoxal sulcus scrobiculate, extending to posterior margin of mesopleuron by distinct carinae; venter granulate; propodeum with basal median areas margined, granulate, basal median carina present, areola not margined, areolar area areolate-rugose, lateral areas entirely rugose. Wings: fore wing vein r shorter than vein 3RSa, vein 1cu-a beyond vein 1M; hind wing vein SC+R absent, vein M+CU longer than vein 1M. Metasoma: first tergum longitudinally costate, length greater than apical width; second tergum longitudinally costate; anterior transverse groove present, weak, straight; posterior transverse groove present; third tergum smooth except for costate transverse groove; terga 4–7 smooth; ovipositor half as long as metasoma.

#### Holotype female.

Top label (white, printed) - Costa Rica, Carthago Pr. [;] La Cangreja, 1960m [;] 1991: x, P. Hanson; second label (red, partially printed and hand written) - HOLOTYPE [;] Heterospilus [;] phaeoskelus [;] P. Marsh. Deposited in ESUW.

#### Paratypes.

2 ♀♀, same data as holotype with additional date of vi-vii.1992 (ESUW).

#### Comments.

The brown legs, smooth mesopleuron and granulate mesoscutum are distinctive for this species.

#### Etymology.

The specific name is from the Greek *phaios*, meaning brown, and the Greek *skelos*, meaning leg, in reference to the brown legs of this species.

**Figure 270.**
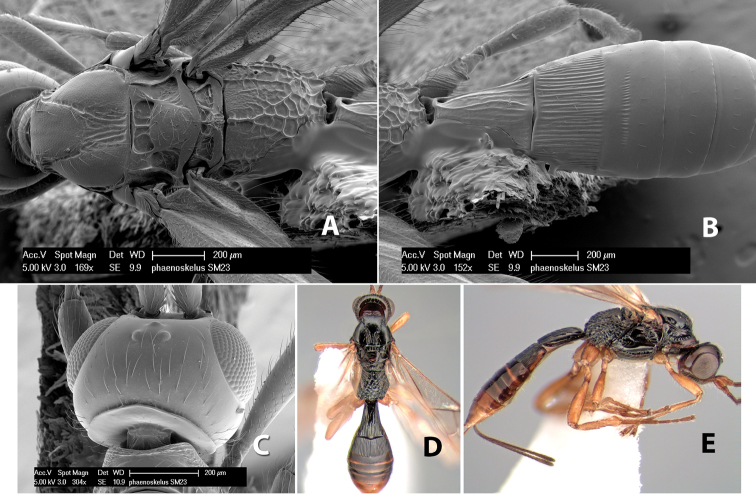
*Heterospilus phaeoskelus* Marsh, sp. n.: **A–C** paratype **D–E** holotype.

### Heterospilus pharkidodus

Marsh sp. n.

http://zoobank.org/8A20C42A-8C7C-4B5C-B226-EBD6B6B6A9C6

http://species-id.net/wiki/Heterospilus_pharkidodus

Figure 271

#### Female.

Body size: 4.0–4.5 mm. Color: body dark brown, apical metasomal terga yellow; scape and flagellum brown, apical 7–10 flagellomeres white; legs yellow except tarsi brown, hind femur light brown, hind tibia dark brown at extreme base; wing veins including stigma brown. Head: vertex smooth; frons weakly transversely costate; face smooth except for numerous setal punctures; temple in dorsal view narrow, sloping behind eye, width less than 1/2 eye width; malar space greater than 1/4 eye height; ocell-ocular distance twice diameter of lateral ocellus; 32 flagellomeres. Mesosoma: mesoscutal lobes weakly granulate, lateral lobes broadly transversely costate along notauli, median lobe transversely costate laterally, apical corners sharply produced; notauli scrobiculate with strong carina on inner side where notauli meet at prescutellar furrow; scutellum smooth; prescutellar furrow with 3 cross carinae; mesopleuron smooth; precoxal sulcus smooth, shorter than mesopleuron; venter smooth; propodeum with basal median areas smooth, distinctly margined, basal median carina absent, areola not margined, areolar area areolate-rugose, lateral areas entirely areolate-rugose. Wings: fore wing vein r less than 1/2 length of vein 3RSa, vein 1cu-a beyond vein 1M; hind wing vein SC+R present, vein M+CU slightly shorter than vein 1M. Metasoma: first tergum longitudinally porcate, length slightly greater than apical width; second tergum longitudinally costate or porcate; anterior transverse groove present, straight; posterior transverse groove present; third tergum costate, smooth at apex; terga 4–7 smooth; ovipositor longer than metasoma.

#### Holotype female

. Top label (white, printed) - COSTA RICA-Heredia Prov. [;] La Selva Biological Station [;] 10°26'N, 84°01'W, 100m [;] Canopy fogging 32 [;] 3.xi.1994 [;] Project ALAS (FVK32); second label (red, printed) - HOLOTYPE [;] *Heterospilus* [;] *pharkidodus* Marsh. Deposited in ESUW.

#### Paratypes

. 1 ♀, top label - COSTA RICA, Heredia: [;] Est. Biol. La Selva, 50- [;] 150m, 10°26'N, 84°01'W [;] Sep 1998, INBio-OET; second label - 03 Setiembre 1998 [;] Borde suampo [;] M/18/716 (INBC).

#### Comments

. The strongly transversely costate mesoscutum, long ovipositor and smooth face are distinctive for this species.

#### Etymology.

The specific name is from the Greek, *pharkidodes*, meaning wrinkled in reference to the costate and wrinkled mesoscutum.

**Figure 271.**
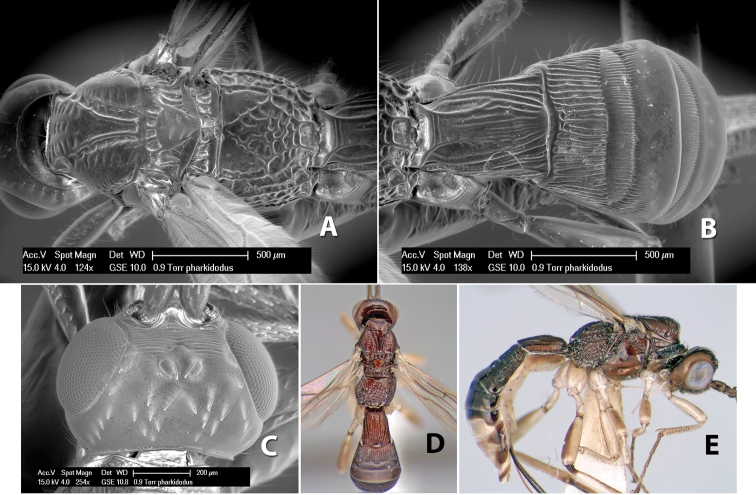
*Heterospilus pharkidodus* Marsh, sp. n., holotype.

### Heterospilus poqomchi

Marsh sp. n.

http://zoobank.org/0492923A-E5F2-4B2C-A775-6A269A84168F

http://species-id.net/wiki/Heterospilus_poqomchi

Figure 272

#### Female.

Body size: 1.5 mm. Color: body entirely brown; scape yellow without lateral brown stripe; flagellum brown, basal flagellomere yellow, apical 3–5 flagellomeres white; wing veins including stigma brown; legs yellow. Head: vertex smooth; frons smooth; face smooth; temple in dorsal view narrow, width greater than 1/2 eye width; malar space greater than 1/4 eye height; ocell-ocular distance greater than 2.5 times diameter of lateral ocellus; 12–14 flagellomeres. Mesosoma: mesoscutal lobes smooth; notauli smooth, meeting posteriorly in weakly costate or unsculptured area; scutellum smooth; prescutellar furrow with 1 cross carina; mesopleuron smooth; precoxal sulcus scrobiculate, shorter than mesopleuron; venter smooth; propodeum with basal median areas margined, smooth, basal median carina present but very short, areola distinctly margined, areolar area broadly rugose, lateral areas entirely rugose. Wings: fore wing vein r shorter than vein 3RSa, vein 1cu-a beyond vein 1M; hind wing vein SC+R absent, vein M+CU shorter than vein 1M. Metasoma: first tergum longitudinally costate, length slightly greater than apical width; second tergum longitudinally costate; anterior transverse groove present, smooth, straight; posterior transverse groove present, smooth; third tergum entirely smooth; terga 4–7 smooth; ovipositor as long as metasomal terga 1 and 2 combined.

#### Holotype female.

Top label (white, printed) - COSTA RICA: Puntar [;] Golfo Dulce 24km W [;] Piedras Blancas [;] 200m, xii 89-iii 1990 [;] Col. Paul Hanson; second label (red, partially printed and hand written) - HOLOTYPE [;] Heterospilus [;] poqomchi [;] P. Marsh. Deposited in ESUW.

#### Paratypes.

3 ♀♀, same data as holotype, additional dates of Feb. 1992, III.1993 and VIII-IX-1993 (ESUW). 1 ♀, Costa Rica: Alajuela, ACA [;] San Carlos, R.F. Arenal [;] Sendero Pilon, 600m, Malaise [;] 14.x–3.xii.1998, G. Carballo [;] L.N. 269100-457900 #53365 (ESUW). 2 ♀♀, COSTA RICA, Puntar. [;] Golfo Dulce, 24km W. [;] PiedrasBlancas, 200m [;] iIII-VI-90 Hanson (MICR).

#### Comments.

The small size, distinctly margined areola on the propodeum and the short tricolored flagellum are distinctive for this species.

#### Etymology.

Named for the Poqomchi’, a Mayan people of Guatemala.

**Figure 272.**
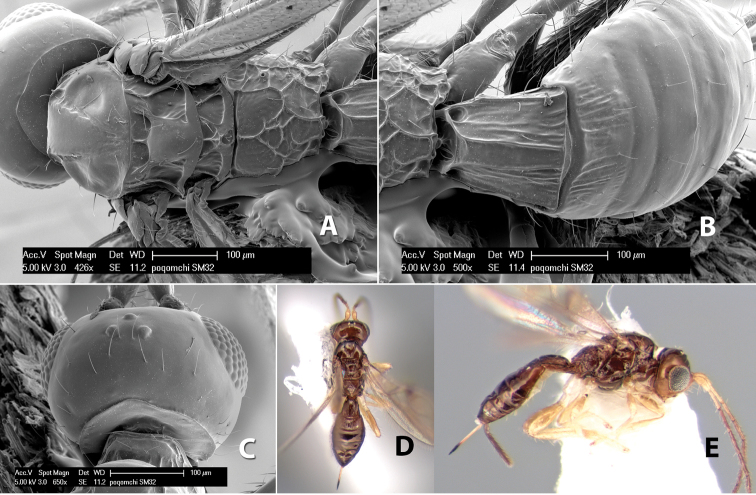
*Heterospilus poqomchi* Marsh, sp. n.: **A–C** paratype **D–E** holotype.

### Heterospilus qanjobal

Marsh sp. n.

http://zoobank.org/070B8E53-9DAF-472F-AE9C-86065E482AFA

http://species-id.net/wiki/Heterospilus_qanjobal

Figure 273

#### Female.

Body size: 3.5–4.0 mm. Color: head and mesosoma brown to dark brown, metasoma brown, terga 1 dark brown; scape yellow without lateral brown stripe, flagellum brown; legs yellow to light brown. Head: vertex smooth; frons smooth; face smooth; temple in dorsal view broad, bulging behind eye, width greater than 1/2 eye width; malar space greater than 1/4 eye height; ocell-ocular distance slightly greater than 2.5 times diameter of lateral ocellus; 24 flagellomeres. Mesosoma: mesoscutal lobes weakly granulate, median lobe with longitudinal median shallow smooth groove; notauli scrobiculate, meeting posteriorly in triangular rugose area; scutellum smooth; prescutellar furrow with 1 cross carina; mesopleuron smooth; precoxal sulcus strongly scrobiculate, as long as mesopleuron; venter smooth; propodeum with basal median areas margined, granulate, basal median carina present, areola distinctly margined, areolar area rugose, lateral areas entirely rugose. Wings: fore wing vein r shorter than vein 3RSa, vein 1cu-a beyond vein 1M; hind wing vein SC+R absent, vein M+CU slightly shorter or equal to vein 1M. Metasoma: first tergum rugose, length greater than apical width, median raised area bordered by distinct margined smooth grooves; second tergum longitudinally costate medially, smooth apically and laterally; anterior transverse groove weakly indicated or absent; posterior transverse groove weakly indicated or absent; third tergum entirely smooth; terga 4–7 smooth; ovipositor as long as metasoma.

#### Holotype female.

Top label (white, printed) - Costa Rica: San Jose [;] Zurqui de Moravia [;] 1600m, ii.1994 [;] Paul Hanson; second label (red, partially printed and hand written) - HOLOTYPE [;] Heterospilus [;] qanjobal [;] P. Marsh. Deposited in ESUW.

#### Paratypes.

1 ♀, same data as holotype with date of III-1995 (ESUW).

#### Comments.

The strongly scrobiculate precoxal sulcus which is as long as the mesopleuron, the large body size and the rugose metasomal tergum 1 are distinctive for this species.

#### Etymology.

Named for the Q’anjob’al, a Mayan people of Guatemala.

**Figure 273.**
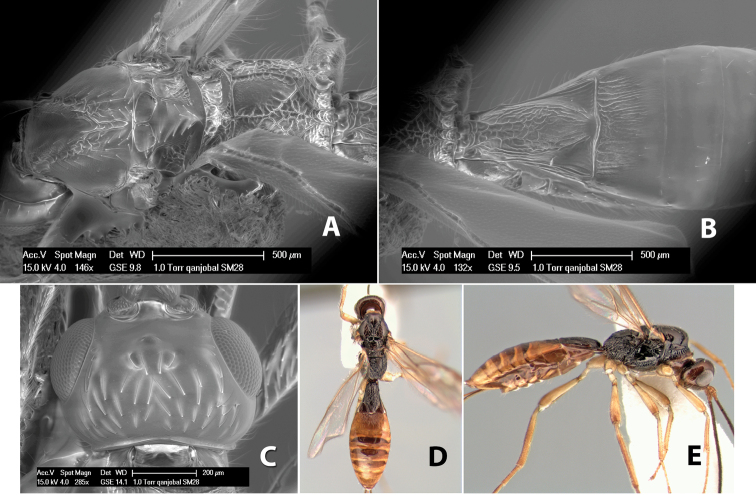
*Heterospilus qanjobal* Marsh, sp. n., holotype.

### Heterospilus racostica

Marsh sp. n.

http://zoobank.org/F2F64555-8033-4281-8468-5098C2F7F105

http://species-id.net/wiki/Heterospilus_racostica

Figure 274

#### Female.

Body size: 2.5 mm. Color: body dark brown, apical metasomal terga slightly lighter; scape yellow without lateral brown stripe; flagellum brown; wing veins including stigma brown; legs yellow or light brown. Head: vertex smooth; frons smooth; face smooth; temple in dorsal view sloping behind eye, width equal to 1/2 eye width; malar space slightly greater than 1/4 eye height; ocell-ocular distance greater than 2.5 times diameter of lateral ocellus; 21 flagellomeres. Mesosoma: mesoscutal lobes weakly granulate or partially smooth; notauli scrobiculate, meeting posteriorly in triangular costate area; scutellum smooth; prescutellar furrow with 3 cross carinae; mesopleuron weakly granulate or partially smooth; precoxal sulcus smooth, shorter than mesopleuron; venter smooth; propodeum with basal median areas margined, granulate, basal median carina absent, areola not margined, areolar area rugose, lateral areas entirely rugose. Wings: fore wing vein r shorter than vein 3RSa, vein 1cu-a interstitial with vein 1M; hind wing vein SC+R present, vein M+CU shorter than vein 1M. Metasoma: first tergum longitudinally costate, length greater than apical width; second tergum longitudinally costate; anterior transverse groove present, straight; posterior transverse groove present; third and fourth terga costate basally, smooth apically; terga 5–7 smooth; ovipositor half as long as metasoma.

#### Holotype female.

Top label (white, printed) - Costa Rica: Puntarenas [;] Peninsula Osa, Puerto [;] Jimenez, 10m, x-xi.1991 [;] P. Hanson, Malaise trap [;] grassy, disturbed site; second label (red, partially printed and hand written) - HOLOTYPE [;] Heterospilus [;] racostica [;] P. Marsh. Deposited in ESUW.

#### Paratypes.

1 ♀, Costa Rica: Puntarenas [;] R.F. Golfo Dulce, [;] 3km. SW. Rincon, 10m [;] Oct. 1991, Paul Hanson (ESUW). 1 ♀, Costa Rica: Puntarenas [;] R.F. Golfo Dulce, 5Km. W [;] Piedras Blancas, 100m [;] xi-xii.1992, P. Hanson (ESUW). 1 ♀, top label - Costa Rica: Guanacaste [;] Santa Rosa Natl. Park [;] 300m, ex. Malaise trap [;] Site #: SE-6-C [;] Dates: 2–23.iii.1986 [;] I.D. Gauld & D. Janzen; second label - [SE] Bosque San Emilio [;] 50yr old deciduous forest [;] [C] more or less fully [;] isolated as possible (ESUW). 1 ♀, Costa Rica: Cartago [;] Turrialba, CATIE [;] 14–15 March 1990 [;] 700m, J.S. Noyes (ESUW). 1 ♀, Costa Rica: Heredia [;] 3km S. Puerto Viejo [;] OTS, La Selva, 100m [;] 1–15 ix 1992, P. Hanson [;] huertos Malaise trap [;] set by G. Wright (ESUW). 1 ♀, COSTA RICA, SanJosé [;] P.N.BraulioCarillo [;] 9.5Km E tunnel, 1000m [;] VI/1989, col. Hanson (MICR). 1 ♀, top label - Turrialba,C.R. [;] IV-21-1957 [;] RDShenefelt [;] RDS 57-128; second label - forest edge of plantation (AEIC).

#### Comments.

The long, narrow metasomal tergum 1 and the brown flagellum are distinctive for this species.

#### Etymology.

The specific name is an anagram of Costa Rica.

**Figure 274.**
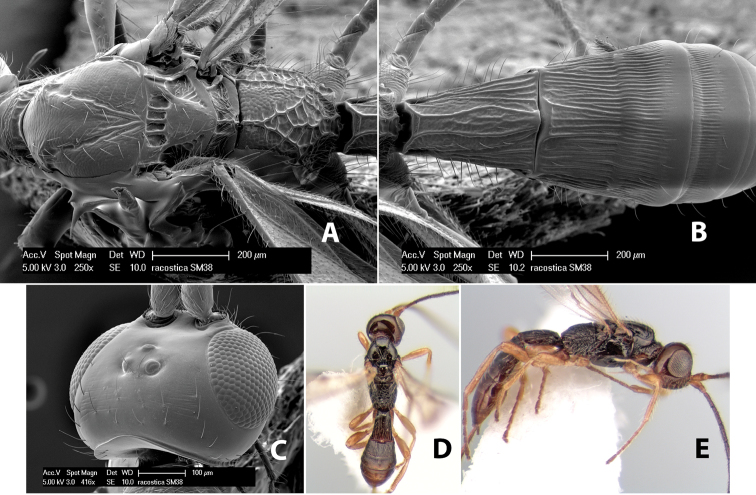
*Heterospilus racostica* Marsh, sp. n.: **A–C** paratype **D–E** holotype.

### Heterospilus reagani

Marsh sp. n.

http://zoobank.org/EFB2477E-1AE0-402F-8F39-3299584B78EF

http://species-id.net/wiki/Heterospilus_reagani

Figure 275

#### Female.

Body size: 4.0–4.5 mm. Color: head dark brown; scape yellow or light brown with lateral longitudinal brown stripe; flagellum brown with apical white annulus, apical 5–8 flagellomeres brown; mesosoma usually brown, occasionally dark brown; metasomal terga dark brown; wing veins including stigma light brown; legs yellow. Head: vertex smooth, rarely with weak striations around antennal bases; frons transversely striate; face rugose; temple in dorsal view narrow but somewhat bulging behind eye, width equal to 1/2 eye width; malar space greater than 1/4 eye height; ocell-ocular distance about twice diameter of lateral ocellus; 30–34 flagellomeres. Mesosoma: mesoscutal lobes smooth; notauli scrobiculate, meeting posteriorly in triangular costate area; scutellum smooth; prescutellar furrow with 5 cross carinae; mesopleuron smooth at least above precoxal sulcus, occasionally weakly granulate dorsally; precoxal sulcus smooth, shorter than mesopleuron; venter smooth; propodeum with basal median areas margined, smooth, basal median carina absent, areola not margined, areolar area areolate, lateral areas mostly rugose with small smooth area basally. Wings: fore wing vein r shorter than vein 3RSa, vein 1cu-a beyond vein 1M; hind wing vein SC+R present, vein M+CU shorter than vein 1M. Metasoma: first tergum longitudinally costate, length greater than apical width; second tergum longitudinally costate, width about 4 times length, with raised smooth median area at basal border; anterior transverse groove present, slightly sinuate; posterior transverse groove present; third tergum costate basally, smooth apically; terga 4–7 smooth; ovipositor as long as metasoma.

#### Holotype female.

Top label (white, printed) - Costa Rica: Alajuela, ACA [;] San Carlos, Res. F. Arenal [;] Sendero Pilon, 600m, Malaise [;] 14.x–3.xii.1998, G. Carballo [;] L.N. 269100-457900 #53365; second label (red, partially printed and hand written) - HOLOTYPE [;] Heterospilus [;] reagani [;] P. Marsh. Deposited in ESUW.

#### Paratypes.

3 ♀♀, same data as holotype (ESUW). 1 ♀, Costa Rica: Puntarenas [;] San Vito - Las Cruces [;] 22-IV to 5-V-1988 [;] P. Hanson (TAMU).

#### Comments.

The large size of the body, the antennae with apical white annulus, the smooth mesoscutum and the raised smooth area at the base of metasomal tergum 2 are distinctive for this species.

#### Etymology.

Named for the 40th president of the United States, Ronald Reagan.

**Figure 275.**
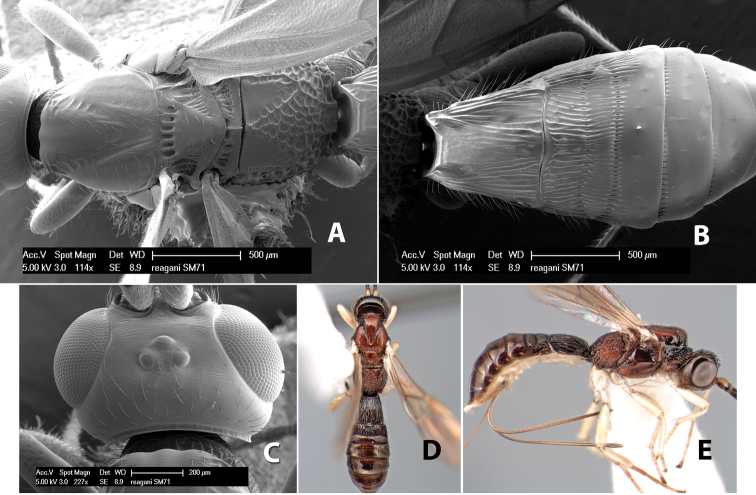
*Heterospilus reagani* Marsh, sp. n.: **A–C** paratype **D–E** holotype.

### Heterospilus retheospilus

Marsh sp. n.

http://zoobank.org/ADEABE1A-8A80-4CA3-BB36-E9956386B566

http://species-id.net/wiki/Heterospilus_retheospilus

Figure 276

#### Female.

Body size: 3.0 mm. Color: body dark brown, apical metasomal terga somewhat lighter brown; scape yellow with weak lateral longitudinal brown stripe, flagellum brown with apical 3–5 flagellomeres white; wing veins including stigma brown; legs light brown. Head: vertex smooth; frons smooth; face smooth; temple in dorsal view narrow, sloping behind eye, width equal to 1/2 eye width; malar space greater than 1/4 eye height; ocell-ocular distance greater than 2.5 times diameter of lateral ocellus; 18 flagellomeres. Mesosoma: mesoscutal lobes weakly granulate; notauli weakly scrobiculate, meeting posteriorly in unsculptured area; scutellum weakly granulate; prescutellar furrow with 1 cross carina; mesopleuron granulate; precoxal sulcus weakly scrobiculate, shorter than mesopleuron; venter granulate; propodeum with basal median areas margined, granulate, basal median carina present, areola distinctly margined, areolar area rugose, lateral areas rugose posteriorly, granulate anteriorly. Wings: fore wing vein r shorter than vein 3RSa, vein 1cu-a interstitial with vein 1M; hind wing vein SC+R absent, vein M+CU shorter than vein 1M. Metasoma: first tergum longitudinally costate, length greater than apical width; second tergum longitudinally costate; anterior transverse groove very weak and nearly absent, straight; posterior transverse groove indicated by very weak impressed line, nearly absent; third tergum smooth; terga 4–7 smooth; ovipositor half as long as metasoma.

#### Holotype female.

Top label (white, printed) - COSTA RICA: [;] San Jose [;] Zurqui de Moravia [;] 1600m, viii-ix 1989 [;] Col. Paul Hanson; second label (red, partially printed and hand written) - HOLOTYPE [;] Heterospilus [;] retheospilus [;] P. Marsh. Deposited in ESUW.

#### Paratypes.

Known only from the holotype.

#### Comments.

The long a narrow metasomal tergum 1 and the white annulus on the flagellum are distinctive for this species.

#### Etymology.

The specific name is an anagram of the generic name *Heterospilus*.

**Figure 276.**
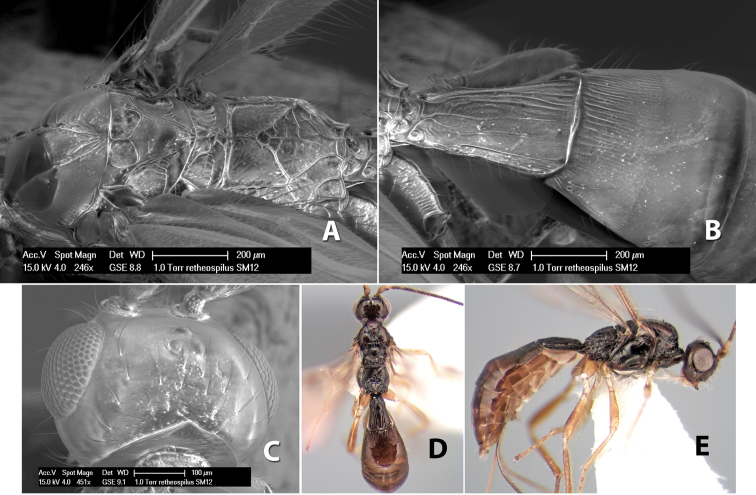
*Heterospilus retheospilus* Marsh, sp. n., holotype.

### Heterospilus ricacosta

Marsh sp. n.

http://zoobank.org/AA7FC364-5DBB-4768-8EEB-8BA7B60ECB16

http://species-id.net/wiki/Heterospilus_ricacosta

Figure 277

#### Female.

Body size: 2.5 mm. Color: body dark brown, metasomal tergum 2 honey yellow medially, tergum 1 at apex sometimes yellow, tergum 3 at base sometimes yellow; scape yellow without lateral brown stripe; flagellum brown with apical 3–5 flagellomeres white, apical one sometimes darker; wing veins including stigma brown; legs yellow. Head: vertex smooth; frons smooth; face smooth; temple in dorsal view narrow, width less than 1/2 eye width; malar space greater than 1/4 eye height; ocell-ocular distance greater than 2.5 times diameter of lateral ocellus; 18–20 flagellomeres. Mesosoma: mesoscutal lobes smooth; notauli weakly scrobiculate, meeting posteriorly in triangular costate area; scutellum smooth; prescutellar furrow with 1 strong median cross carina and often weaker carinae on each side; mesopleuron smooth; precoxal sulcus scrobiculate, shorter than mesopleuron but often with carinae extending to posterior margin of mesopleuron; venter smooth; propodeum with basal median areas margined, rugose, basal median carina absent, areola not margined, areolar area rugose, lateral areas entirely rugose. Wings: fore wing vein r shorter than vein 3RSa, vein 1cu-a slightly beyond or interstitial vein 1M; hind wing vein SC+R absent, vein M+CU shorter than vein 1M. Metasoma: first tergum longitudinally costate, length equal to apical width; second tergum longitudinally costate; anterior transverse groove present, straight; posterior transverse groove present; third tergum smooth; terga 4–7 smooth; ovipositor half as long as metasoma.

#### Holotype female.

Top label (white, printed) - Costa Rica, Puntarenas [;] Pen. Osa, 5km. N, [;] Puerto Jimenez, 10m [;] I-II-1993 P. Hanson; second label (red, partially printed and hand written) - HOLOTYPE [;] Heterospilus [;] ricacosta [;] P. Marsh. Deposited in ESUW.

#### Paratypes.

2 ♀♀, same data as holotype with additional dates of VIII-IX-1993 (ESUW). 1 ♀, Costa Rica: Puntarenas [;] Pen. Osa, Puerto Jimenez [;] 10m, July 1991, full sun, [;] grassy & weedy site [;] P. Hanson, ex. Malaise (ESUW). 1 ♀, Costa Rica: Heredia [;] Est. Biol. La Selva [;] 50–150m, 10.26 N [;] 84.01 W, Aug. 1992 (ESUW). 2 ♀♀, Costa Rica: Alajuela [;] 5km. W San Ramon [;] 1200m, April 1997 [;] O. Castro & P. Hanson (ESUW). 1 ♀, COSTA RICA, Guanac [;] Estac. Maritza, W [;] Volcán Orosi, 600m [;] 1988–1989 (MICR). 1 ♀, COSTA RICA, Heredia [;] Rio Frio, Banano [;] 100m, X 1989 [;] col. Edgar Quirós (MICR).

#### Comments.

The short temple, smooth mesoscutum and honey yellow metasomal tergum 2 are distinctive for this species.

#### Etymology.

The specific name is an anagram of Costa Rica.

**Figure 277.**
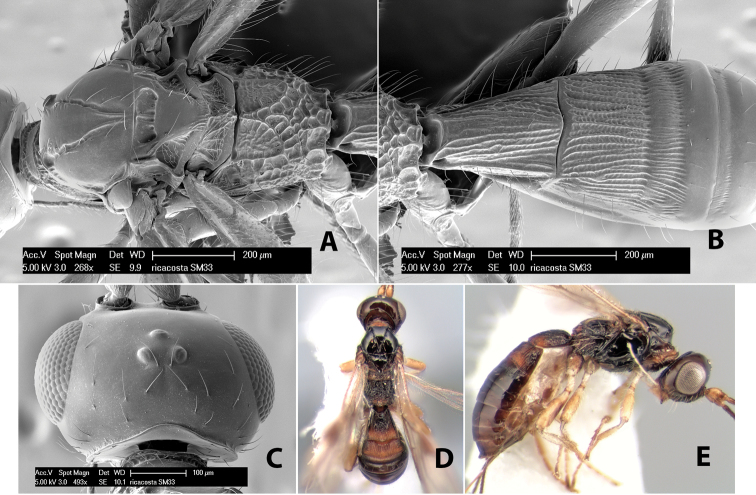
*Heterospilus ricacosta* Marsh, sp. n.: **A–C** paratype **D–E** holotype.

### Heterospilus sanjosensis

Marsh sp. n.

http://zoobank.org/AFCA94CA-3EDA-4021-A86D-F802079B9C62

http://species-id.net/wiki/Heterospilus_sanjosensis

Figure 278

#### Female.

Body size: 2.0–2.5 mm. Color: head and mesosoma dark brown, metasomal terga light brown, tergum 1 often darker brown; scape yellow without lateral brown stripe, flagellum brown; wing veins including stigma brown; legs yellow. Head: vertex smooth; frons smooth; face smooth; temple in dorsal view broad but sloping behind eye, width slightly greater than 1/2 eye width; malar space greater than 1/4 eye height; ocell-ocular distance greater than 2.5 times diameter of lateral ocellus; 14–15 flagellomeres. Mesosoma: mesoscutal lobes weakly granulate and shining; notauli mostly smooth, meeting at scutellum in unsculptured area; scutellum weakly granulate; prescutellar furrow with 1 cross carina; mesopleuron smooth above precoxal sulcus, weakly granulate dorsally; precoxal sulcus weakly scrobiculate, shorter than mesopleuron; venter smooth; propodeum with basal median areas margined, granulate, basal median carina absent, areola not margined, areolar area areolate-rugose, lateral areas entirely rugose. Wings: fore wing vein r equal to or slightly shorter than vein 3RSa, vein 1cu-a slightly beyond vein 1M; hind wing vein SC+R absent, vein M+CU equal in length to vein 1M. Metasoma: first tergum weakly longitudinally costate, length greater than apical width; second tergum weakly costate basally, smooth apically; anterior transverse groove present, weak, straight; posterior transverse groove present, weak; third tergum entirely smooth; terga 4–7 smooth; ovipositor half as long as metasoma.

#### Holotype female.

Top label (white, printed) - Costa Rica: San Jose [;] Cerro de la Muerte [;] 6km. N. San Gerardo [;] 2800m, August 1992 [;] P. Hanson, Malaise; second label (red, partially printed and hand written) - HOLOTYPE [;] Heterospilus [;] sanjosensis [;] P. Marsh. Deposited in ESUW.

#### Paratypes.

1 ♀, Costa Rica: San Jose [;] Zurqui de Moravia [;] 1600m, P. Hanson [;] ix.1995 (ESUW). 2 ♀♀, Costa Rica: San Jose [;] 26km. N. San Isidro [;] just S. of Division [;] 2100m, viii-ix.1991 and vi-viii.1992 [;] P. Hanson, Malaise [;] secondary growth (ESUW). 1 ♀, COSTA RICA: Alajuela [;] San Pedro de la [;] Tigra Cacao, 200m [;] I-II 1990 R. Cespedes (ESUW). 1 ♀, Costa Rica: Alajuela [;] 5km W San Ramon [;] 1200m, iv.1997 [;] O. Castro & P. Hanson (ESUW). 1 ♀, COSTA RICA: San Jose [;] Zurqui de Moravia [;] ix–x.1993 1600m [;] P. Hanson (TAMU). 1 ♀, Costa Rica: Puntarenas [;] San Vito - Las Cruces [;] 5-VI-1988 [;] P. Hanson (TAMU). 1 ♀, COSTA RICA, *Punt-* [;] *arenas*, Las Alturas, [;] 1600m; 10–130. vi.1998; [;] Brown & Berezovskiy; [;] Mal. Trp. #2; for. edge (AEIC).

#### Comments.

The short antennae, the metasomal tergum 2 which is smooth apically and the unsculptured notauli are distinctive for this species.

#### Etymology.

Named for San Jose Province where most of the type series was collected.

**Figure 278.**
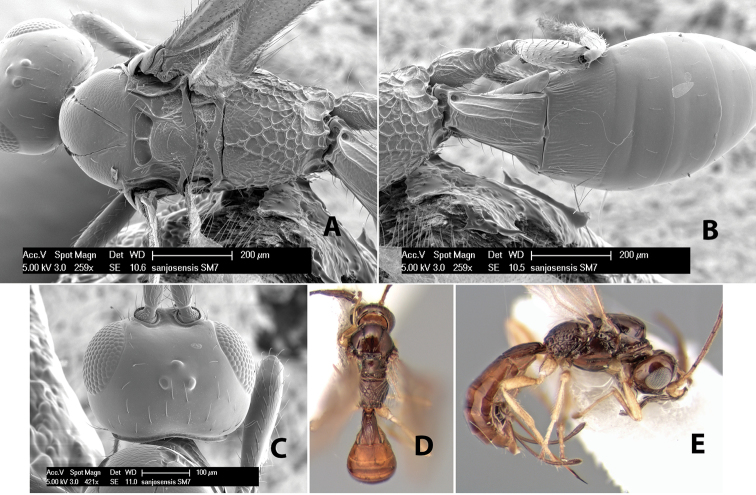
*Heterospilus sanjosensis* Marsh, sp. n.: **A–C** paratype **D–E** holotype.

### Heterospilus saturn

Marsh sp. n.

http://zoobank.org/9C792C78-53DE-4D4E-9D13-DE8A294F39D1

http://species-id.net/wiki/Heterospilus_saturn

Figure 279

#### Female.

Body size: 2.75 mm. Color: head with vertex and frons light brown, face yellow; scape yellow without lateral brown stripe; flagellum brown; mesosoma brown; wing veins including stigma light brown; legs yellow; metasomal tergum 1 brown, terga 2–6 honey yellow, tergum 2 brown medially and laterally, tergum 3 brown laterally. Head: vertex smooth; frons smooth; face weakly striate medially, smooth laterally; temple in dorsal view narrow, width less than 1/2 eye width; malar space slightly greater than 1/4 eye height; ocell-ocular distance about 2.5 times diameter of lateral ocellus; 23–24 flagellomeres. Mesosoma: mesoscutal lobes granulate, with long sparse hairs along notauli giving appearance of lobes being entirely hairy; notauli scrobiculate, meeting posteriorly in rectangular rugose area; scutellum weakly granulate or smooth; prescutellar furrow with 3–5 cross carinae; mesopleuron smooth; precoxal sulcus smooth, shorter than mesopleuron; venter smooth; propodeum with basal median areas margined, granulate, basal median carina absent, areola not margined, areolar area areolate-rugose, lateral areas entirely rugose. Wings: fore wing vein r slightly shorter than vein 3RSa, vein 1cu-a beyond vein 1M; hind wing vein SC+R present, vein M+CU shorter than vein 1M. Metasoma: first tergum longitudinally costate-granulate, length equal to apical width; second tergum longitudinally costate-granulate; anterior transverse groove present, straight; posterior transverse groove present; third tergum costate basally, smooth apically; terga 4–7 smooth; ovipositor half as long as metasoma.

#### Holotype female.

Top label (white, partially printed and hand written) - Costa Rica: Guanacaste [;] Santa Rosa Natl. Park [;] 300m, ex. Malaise trap [;] Site #: H-2-O [;] Dates: 20.xii.86–10.i.1987 [;] I.D. Gauld & D. Janzen; second label (white, printed) - [H] open regenerating [;] woodland, 10 years old [;] [O] in clearing, fully [;] isolated part of day; third label (red, partially printed and hand written) - HOLOTYPE [;] Heterospilus [;] saturn [;] P. Marsh. Deposited in ESUW.

#### Paratypes.

1 ♀, top label - Costa Rica: Guanacaste [;] Santa Rosa Natl. Park [;] 300m, ex. Malaise trap [;] Site #: 8 [;] Dates: 23.iii–13.iv.1986 [;] I.D. Gauld & D. Janzen; second label (white, printed) - [SE] San Emilio [;] 50yr old deciduous forest [;] [C] more or less fully [;] shaded as possible (ESUW). 7 ♀♀, S.RosaPark, Guan. [;] C. Rica 5 Jan 78 to 4 Nov 77 [;] D.H. Janzen [;] Dry Hill and Riparian (AEIC).

#### Comments.

The sparse long hair along the notauli, the bicolored metasomal tergum 2 and the absence of the basal median carina of the propodeum are distinctive for this species.

#### Etymology.

Named for the Roman god, Saturn.

**Figure 279.**
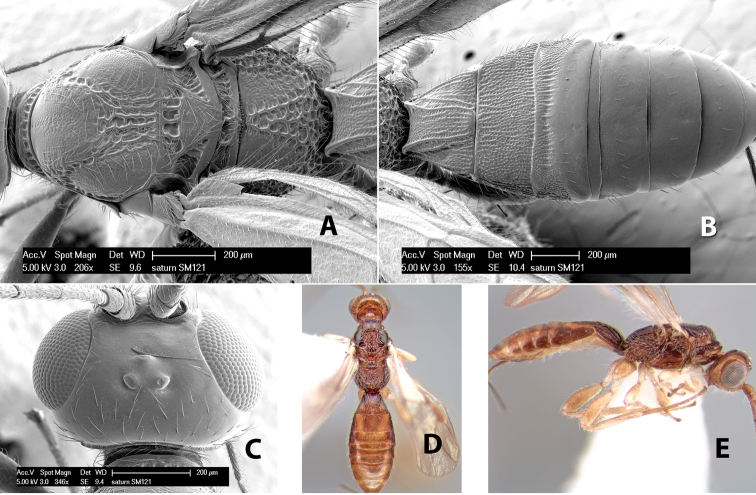
*Heterospilus saturn* Marsh, sp. n.: **A–C** paratype **D–E** holotype.

### Heterospilus seis

Marsh sp. n.

http://zoobank.org/C5EE9217-E9E0-4170-B867-47484CBE8A3C

http://species-id.net/wiki/Heterospilus_seis

Figure 280

#### Female.

Body size: mm. Color: head with vertex and temple dark brown, face light brown; mesosoma and metasoma dark brown, apical metasomal terga slightly lighter brown; scape yellow without lateral brown stripe; flagellum brown; wing veins including stigma brown; legs yellow. Head: vertex smooth; frons smooth; face weakly striate and partially smooth; temple in dorsal view narrow, width less than 1/2 eye width; malar space greater than 1/4 eye height; ocell-ocular distance greater than 2.5 times diameter of lateral ocellus; 18 flagellomeres. Mesosoma: mesoscutal lobes granulate; notauli scrobiculate, meeting posteriorly in triangular costate area; scutellum granulate; prescutellar furrow with 3–5 cross carinae; mesopleuron granulate; precoxal sulcus scrobiculate, shorter than mesopleuron; venter granulate; propodeum with basal median areas margined, granulate, basal median carina present, areola distinctly margined, areolar area rugose, lateral areas entirely rugose. Wings: fore wing vein r shorter than vein 3RSa, vein 1cu-a beyond vein 1M; hind wing vein SC+R present, vein M+CU shorter than vein 1M. Metasoma: first tergum longitudinally costate, length slightly greater than apical width; second tergum longitudinally costate; anterior transverse groove present, straight; posterior transverse groove present; third tergum smooth except for costate transverse groove; terga 4–7 smooth; ovipositor as long as metasomal terga 1 and 2 combined.

#### Holotype female.

Top label (white, partially printed and hand written) - Costa Rica: Guanacaste [;] Santa Rosa National Pk. [;] 300m, Malaise, Ian Gauld [;] 27.ix–18.x.1986; second label (white, partially printed and hand written) - Bosque San Emilio [;] 50yr old deciduous [;] forest. Sun; third label (white, printed) - SE 7-O [;] 27.ix–18.x.86; fourth label (red, partially printed and hand written) - HOLOTYPE [;] Heterospilus [;] seis [;] P. Marsh. Deposited in ESUW.

#### Paratypes.

Known only from the holotype.

#### Comments.

The short ovipositor, brown flagellum and presence of the basal median carina on the propodeum are distinctive for this species.

#### Etymology.

The species name is an arbitrary combination of letters.

**Figure 280.**
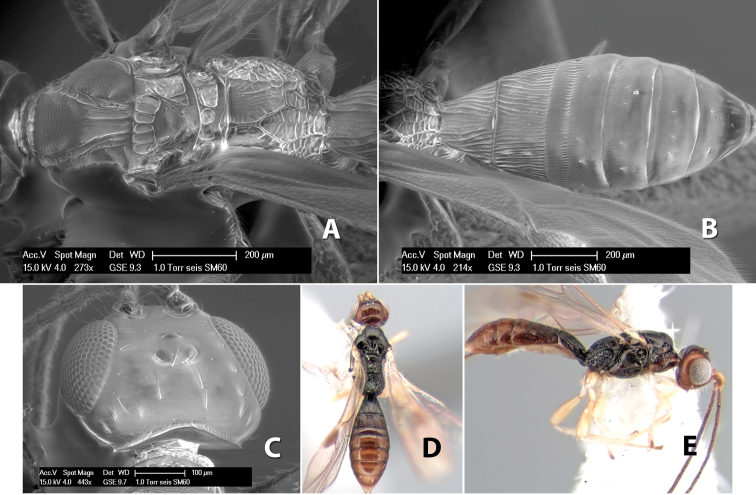
*Heterospilus seis* Marsh, sp. n., holotype.

### Heterospilus shonan

Marsh sp. n.

http://zoobank.org/D2F34716-AB2D-49AF-B44C-E4B81077CBF4

http://species-id.net/wiki/Heterospilus_shonan

Figure 281

#### Female.

Body size: 3.0 mm. Color: head and mesosoma dark brown, metasomal tergum dark brown, remainder of terga lighter brown; scape yellow without lateral brown stripe, flagellum brown; wing veins including stigma brown; legs yellow. Head: vertex smooth; frons smooth; face smooth; temple in dorsal view narrow, sloping behind eye, width equal to 1/2 eye width; malar space greater than 1/4 eye height; ocell-ocular distance about 2.5 times diameter of lateral ocellus; 17 flagellomeres, apical 3–5 flagellomeres short, length 2.0–2.5 times width. Mesosoma: mesoscutal lobes granulate; notauli scrobiculate, meeting at scutellum in triangular costate-rugose area; scutellum smooth; prescutellar furrow with 1 cross carina; mesopleuron smooth above precoxal sulcus, granulate or rugose dorsally; precoxal sulcus scrobiculate, nearly as long as mesopleuron; venter smooth; propodeum with basal median areas margined, granulate, basal median carina present, areola distinctly margined, areolar area rugose, lateral areas entirely rugose. Wings: fore wing vein r shorter than vein 3RSa, vein 1cu-a beyond vein 1M; hind wing vein SC+R absent, vein M+CU shorter than vein 1M. Metasoma: first tergum longitudinally costate, median raised area bordered laterally by distinct smooth grooves; second tergum granulate on basal half, smooth on apical half; anterior transverse groove indicated by weak shallow line, straight; posterior transverse groove indicated by weak shallow line; third tergum smooth entirely; terga 4–7 smooth; ovipositor as long as metasomal terga 1 and 2 combined.

#### Holotype female.

Top label (white, printed) - Costa Rica: San Jose [;] Zurqui de Moravia [;] 1600m, v 1992 [;] Col. Paul Hanson; second label (red, partially printed and hand written) - HOLOTYPE [;] Heterospilus [;] shonan [;] P. Marsh. Deposited in ESUW

#### Paratypes.

Known only from the holotype.

#### Comments.

The partially smooth and granulate metasomal tergum 2, short ovipositor and distinct median basal carina on the propodeum are distinctive for this species.

#### Etymology.

The specific name is an anagram of Hanson, referring to the collector of the holotype, Paul Hanson.

**Figure 281.**
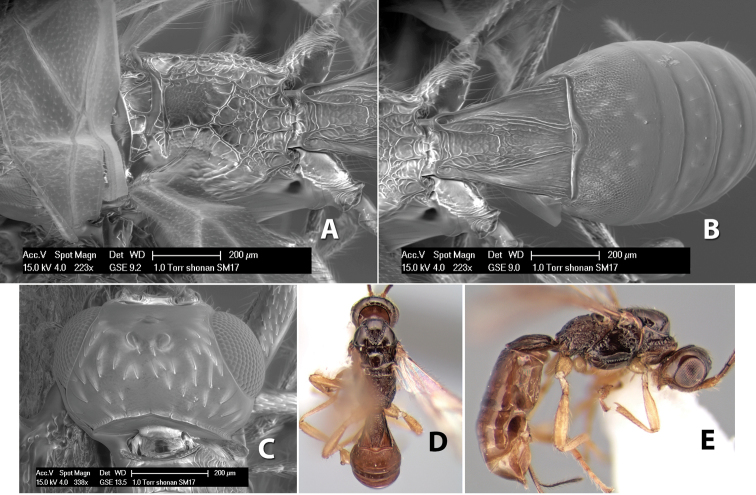
*Heterospilus shonan* Marsh, sp. n., holotype.

### Heterospilus siete

Marsh sp. n.

http://zoobank.org/A0E3370C-6203-485E-8193-28BE25129337

http://species-id.net/wiki/Heterospilus_siete

Figure 282

#### Female.

Body size: 2.5 mm. Color: head with vertex and temple brown, face yellow, mesosoma and metasoma brown, apical metasomal terga lighter brown; scape yellow without lateral brown stripe; flagellum brown; wing veins including stigma brown; legs yellow. Head: vertex smooth; frons smooth; face weakly striate and partially smooth; temple in dorsal view broad, width equal to 1/2 eye width; malar space equal to 1/4 eye height; ocell-ocular distance about 2.5 times diameter of lateral ocellus; 20 flagellomeres. Mesosoma: mesoscutal lobes granulate; notauli scrobiculate, meeting posteriorly in triangular costate-rugose area; scutellum granulate; prescutellar furrow with 1 strong median carina and weaker carinae laterally; mesopleuron granulate; precoxal sulcus smooth, shorter than mesopleuron; venter smooth; propodeum with basal median areas margined, granulate, basal median carina present, long, areola distinctly margined, areolar area rugose, lateral areas entirely rugose. Wings: fore wing vein r shorter than vein 3RSa, vein 1cu-a beyond vein 1M; hind wing vein SC+R present, vein M+CU shorter than vein 1M. Metasoma: first tergum longitudinally costate-granulate, length about equal to apical width; second tergum longitudinally costate; anterior transverse groove weak but present, straight; posterior transverse groove weak but present; third tergum costate basally, smooth apically; terga 4–7 smooth; ovipositor half as long as metasoma.

#### Holotype female.

Top label (white, printed) - Costa Rica: Puntarenas [;] San Vito, Estac. Biol. [;] Las Alturas, 1500m, [;] pasture, V.1992 [;] P. Hanson; second label (red, partially printed and hand written) - HOLOTYPE [;] Heterospilus [;] siete [;] P. Marsh. Deposited in ESUW.

#### Paratypes.

Known only from the holotype.

#### Comments.

The long and distinct median basal carina of the propodeum, the brown flagellum and the ovipositor being half as long as metasoma are distinctive for this species.

#### Etymology.

The specific name is an arbitrary combination of letters.

**Figure 282.**
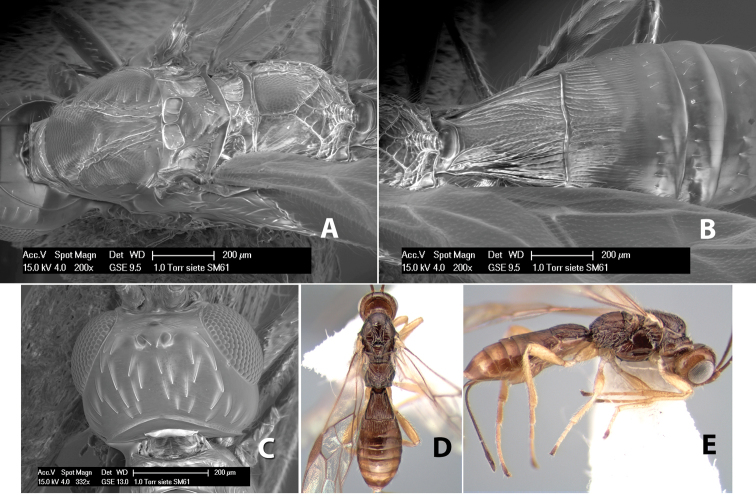
*Heterospilus siete* Marsh, sp. n., holotype.

### Heterospilus smithi

Marsh sp. n.

http://zoobank.org/5EC841DF-51CB-4FC5-A202-7ED9BDA4C4EC

http://species-id.net/wiki/Heterospilus_smithi

Figure 283

#### Female.

Body size: 3.0 mm. Color: body dark brown to black; scape yellow without lateral brown stripe, flagellum brown; wing veins including stigma brown; legs yellow. Head: vertex smooth; frons smooth; face smooth; temple in dorsal view narrow, sloping behind eye, width less than 1/2 eye width; malar space greater than 1/4 eye height; ocell-ocular distance slightly greater than 2.5 times diameter of lateral ocellus; 19–22 flagellomeres. Mesosoma: mesoscutal lobes granulate; notauli scrobiculate, meeting at scutellum in triangular rugose area; scutellum smooth; prescutellar furrow with 3 cross carinae; mesopleuron granulate; precoxal sulcus scrobiculate, shorter than mesopleuron; venter granulate; propodeum with basal median areas margined, granulate, basal median carina present and very short or absent, areola usually margined, areolar area rugose, lateral areas rugose posteriorly, small smooth spot anteriorly. Wings: fore wing vein r shorter than vein 3RSa, vein 1cu-a beyond vein 1M; hind wing vein SC+R absent, vein M+CU slightly longer than vein 1M. Metasoma: first tergum longitudinally costate, median raised area usually distinctly margined by lateral grooves, length greater than apical width; second tergum longitudinally costate; anterior transverse groove present, straight; posterior transverse groove absent; third tergum entirely smooth; terga 4–7 smooth; ovipositor longer than metasoma.

#### Holotype female.

Top label (white, printed) - Costa Rica: Puntarenas [;] San Vito, Estac. Biol. [;] Las Alturas, 2050m [;] ix-xi.1992, Paul Hanson [;] ex. Malaise trap; second label (red, partially printed and hand written) - HOLOTYPE [;] Heterospilus [;] smithi [;] P. Marsh. Deposited in ESUW.

#### Paratypes.

1 ♀, Costa Rica: San Jose [;] Zurqui de Moravia [;] 1600m, February 1996 [;] P. Hanson, Malaise (ESUW). 1 ♀, Costa Rica: Alajuela [;] 5km. W San Ramon [;] 1200m, December 1996 [;] O. Castro & P. Hanson (ESUW). 4 ♀♀, Costa Rica: Puntarenas [;] San Vito, Las Cruces [;] Wilson Botanical Gardens [;] 18–22.iii.1990, 1150m [;] J.S. Noyes (ESUW).

#### Comments.

The long ovipositor, smooth metasomal terga 3–7 and the hind wing vein M+CU longer than vein 1M are distinctive for this species.

#### Etymology.

Named for my friend and long time Systematic Entomology Laboratory colleague, David R. Smith.

**Figure 283.**
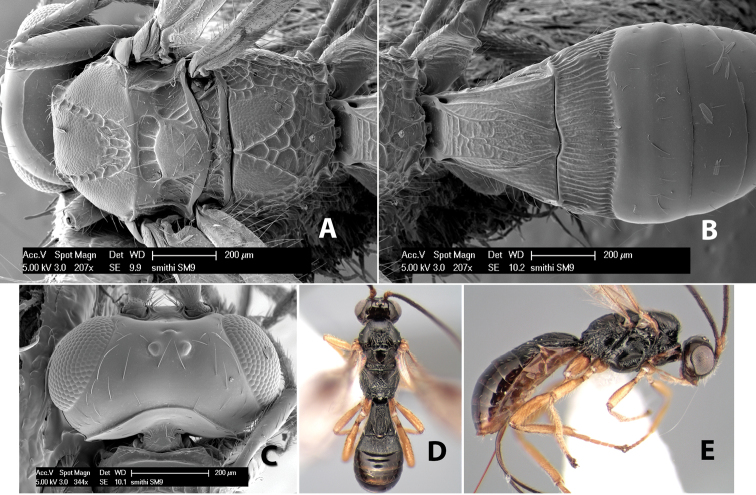
*Heterospilus smithi* Marsh, sp. n.: **A–C** paratype **D–E** holotype.

### Heterospilus terrabas

Marsh sp. n.

http://zoobank.org/58A06AC5-8493-46EB-996C-ABDAD2EC7480

http://species-id.net/wiki/Heterospilus_terrabas

Figure 284

#### Female.

Body size: 2.5–3.0 mm. Color: head with vertex brown, face yellow; scape yellow without lateral brown stripe; flagellum brown; mesosoma and metasoma dark brown; wing veins including stigma brown; legs yellow. Head: vertex smooth; frons smooth; face smooth; temple in dorsal view narrow but slightly bulging behind eye, width less than 1/2 eye width; malar space less than 1/4 eye height; ocell-ocular distance about 1.5 times diameter of lateral ocellus; 21–23 flagellomeres. Mesosoma: mesoscutal lobes granulate; notauli scrobiculate, meeting posteriorly in triangular costate area; scutellum weakly granulate; prescutellar furrow with 1 cross carina; mesopleuron smooth; precoxal sulcus smooth, shorter than mesopleuron; venter smooth; propodeum with basal median areas margined, weakly granulate or partially smooth, basal median carina present, areola distinctly margined, areolar area rugose, lateral areas entirely rugose. Wings: fore wing vein r shorter than vein 3RSa, vein 1cu-a beyond vein 1M; hind wing vein SC+R present, vein M+CU shorter than vein 1M. Metasoma: first tergum longitudinally costate, length greater than apical width; second tergum longitudinally costate; anterior transverse groove present, straight; posterior transverse groove present; third tergum costate basally, smooth apically; terga 4–7 smooth; ovipositor longer than metasoma.

#### Holotype female.

Top label (white, printed) - COSTA RICA: Puntarenas [;] Reserva Forestal Golfo Dulce [;] 3km SW of Rincon, 10m [;] Mar-April 1992, P. Hanson [;] primary forest, Malaise trap; second label (red, partially printed and hand written) - HOLOTYPE [;] Heterospilus [;] terrabas [;] P. Marsh. Deposited in ESUW.

#### Paratypes.

1 ♀, same data as holotype (ESUW).

#### Comments.

The short ocell-ocular distance and short malar space, long ovipositor and single cross carina in the prescutellar furrow are distinctive for this species.

#### Etymology.

Named for the Térrabas, an indigenous people from Puntarenas Province, Costa Rica.

**Figure 284.**
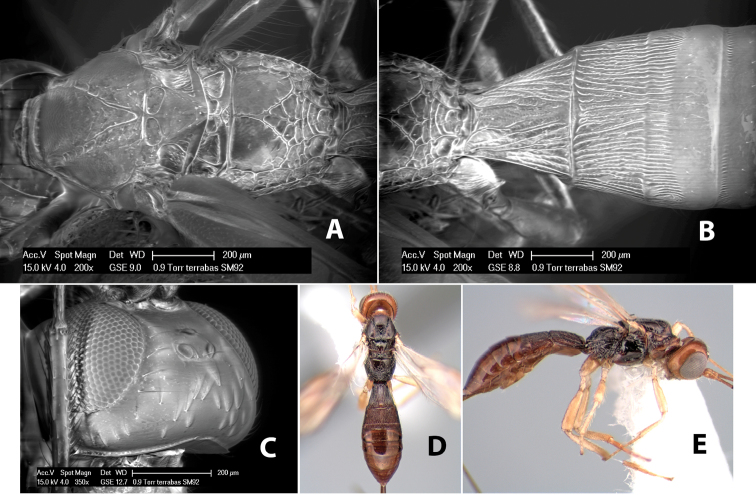
*Heterospilus terrabas* Marsh, sp. n., holotype.

### Heterospilus thereospilus

Marsh sp. n.

http://zoobank.org/94ADC9FC-0AB7-4F3D-B189-B476728D5EAE

http://species-id.net/wiki/Heterospilus_thereospilus

Figure 285

#### Female.

Body size: 2.5 mm. Color: head with vertex brown, face honey yellow; mesosoma and metasoma dark brown, apical metasomal terga somewhat lighter brown; scape yellow without lateral brown stripe; flagellum brown; wing veins including stigma brown; legs yellow. Head: vertex mostly smooth, occasionally weakly costate behind ocelli; frons smooth; face weakly rugose; temple in dorsal view narrow, sloping behind eye, width less than 1/2 eye width; malar space apparently greater than 1/4 eye height (partially hidden in glue); ocell-ocular distance slightly greater than 2.5 times diameter of lateral ocellus; 19 flagellomeres. Mesosoma: mesoscutal lobes granulate; notauli scrobiculate, meeting posteriorly in rectangular rugose area; scutellum granulate; prescutellar furrow with 5 cross carinae; mesopleuron granulate; precoxal sulcus weakly scrobiculate, shorter than mesopleuron; venter possibly granulate (obscured by glue); propodeum with basal median areas margined, granulate, basal median carina absent, areola not margined, areolar area areolate-rugose, lateral areas rugose posteriorly, granulate area anteriorly. Wings: fore wing vein r shorter than vein 3RSa, vein 1cu-a slightly beyond vein 1M; hind wing vein SC+R absent, vein M+CU shorter than vein 1M. Metasoma: first tergum longitudinally costate, length equal to apical width; second tergum longitudinally costate; anterior transverse groove present, straight; posterior transverse groove absent; third tergum costate at extreme base, remainder smooth; terga 4–7 smooth; ovipositor as long as metasomal tergum 1.

#### Holotype female.

Top label (white, printed) - Costa Rica: Puntarenas [;] R.F. Golfo Dulce [;] 3km SW. Rincon, 10m [;] vi.1991, Paul Hanson; second label (red, partially printed and hand written) - HOLOTYPE [;] Heterospilus [;] thereospilus [;] P. Marsh. Deposited in ESUW.

#### Paratypes.

Known only from the holotype.

#### Comments.

The short ovipositor, absence of the posterior transverse groove on metasomal tergum 3 and the rectangular rugose area on the mesoscutum where the notauli meet are distinctive for this species.

#### Etymology.

The specific name is an anagram of the generic name *Heterospilus*.

**Figure 285.**
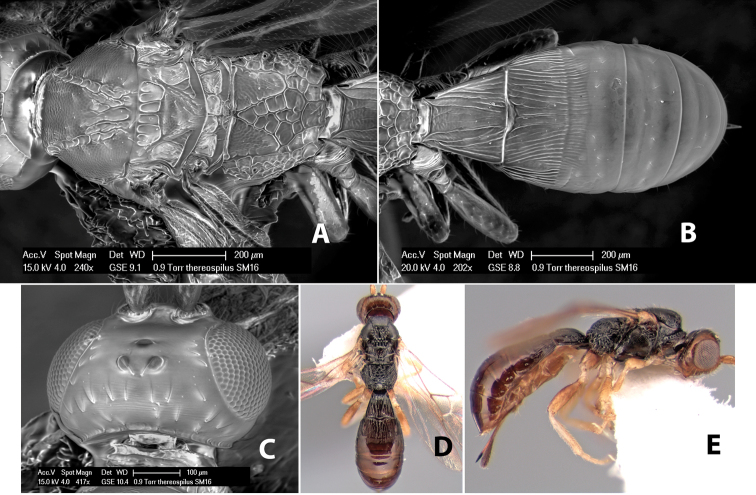
*Heterospilus thereospilus* Marsh, sp. n., holotype.

### Heterospilus trece

Marsh sp. n.

http://zoobank.org/BBEA4D89-969E-4851-8510-3924935E90E1

http://species-id.net/wiki/Heterospilus_trece

Figure 286

#### Female.

Body size: 3.5 mm. Color: body dark brown, apical metasomal terga often lighter brown; scape yellow with lateral longitudinal brown stripe; flagellum brown with apical 10–12 flagellomeres white, apical one sometimes dark; wing veins including stigma brown; legs bicolored brown and yellow, hind femur yellow on basal half, brown on apical half. Head: vertex smooth; frons smooth; face smooth; temple in dorsal view narrow, sloping behind eyes, width less than 1/2 eye width; malar space greater than 1/4 eye height; ocell-ocular distance 2.0–2.5 times diameter of lateral ocellus; 26–31 flagellomeres. Mesosoma: mesoscutal lobes smooth; notauli scrobiculate, meeting posteriorly in triangular costate area; scutellum smooth; prescutellar furrow with 1 distinct median cross carina and, rarely, weaker carinae on each side; mesopleuron smooth; precoxal sulcus smooth, shorter than mesopleuron; venter smooth; propodeum with basal median areas margined, smooth, basal median carina present, often very short, areola not margined, areolar area rugose, lateral areas rugose apically, smooth basally, propodeum with distinct tubercle above hind coxa. Wings: fore wing vein r shorter than vein 3RSa, vein 1cu-a beyond vein 1M; hind wing vein SC+R present, vein M+CU shorter than vein 1M. Metasoma: first tergum longitudinally costate, length greater than apical width; second tergum longitudinally costate, raised smooth area medially at base; anterior transverse groove present or rarely weak or absent, usually straight, rarely slightly sinuate; posterior transverse groove weak or absent; third tergum smooth; terga 4–7 smooth; ovipositor longer than metasoma.

#### Holotype female.

Top label (white, printed) - COSTA RICA: Puntar [;] P.N. Corcovado, Est. [;] Sirena, 50m [;] IV-VIII 1989; second label (red, partially printed and hand written) - HOLOTYPE [;] Heterospilus [;] trece [;] P. Marsh. Deposited in ESUW.

#### Paratypes.

1 ♀, COSTA RICA: Puntarenas [;] Rd, to Rincon, 24km W. [;] Pan-Amer. Hwy, 200m [;] III-V 1989, Hanson & Gauld (ESUW). 2 ♀♀, COSTA RICA: Puntarenas [;] Reserva Forestal Golfo Dulce [;] 3km SW of Rincon, 10m [;] November 1992 and July 1991, P. Hanson [;] primary forest, Malaise trap (ESUW). 1 ♀, top label - Costa Rica: Guanacaste [;] Santa Rosa Natl. Park [;] 300m, ex. Malaise trap [;] Site #: SE-O-5 [;] Dates: 18.x–8.xi.1986 [;] I.D. Gauld & D. Janzen; second label - [SE] Bosque San Emilio [;] 50yr old deciduous forest [;] [O] in clearing, fully isolated part of day (ESUW). 1 ♀, top label - Costa Rica: Guanacaste [;] Santa Rosa Natl. Park [;] 300m, ex. Malaise trap [;] Site #: BH-12-C [;] Dates: 18.x–8.xi.1986 [;] I.D. Gauld & D. Janzen; second label - [BH] Bosque Humedo [;] mature evergreen dry forest [;] [C] more or less fully [;] shaded as possible (ESUW). 1 ♀, top label - Costa Rica: Guanacaste [;] Santa Rosa National Pk. [;] 300m, Malaise trap, Ian Gauld [;] 27.ix–18.x.1986; second label - Bosque San Emilio [;] 50yr Old deciduous [;] forest. Full Shade; third label - SE-8-C [;] 27.ix–18.x.86 (ESUW). 1 ♀, Costa Rica: Guanacaste [;] Est. Cacao, 1000–1150m [;] ix.1996, I. Villegas, Malaise [;] L.N. 323150-375500 #47559 (ESUW). 1 ♀, Costa Rica: Alajuela Prov. [;] Area Conservation de Arena [;] Est. San Ramon, Malaise #3 [;] in veg. on Sendero W.F. [;] 5 June to 15 July 1998 [;] N. Zitani, S. Dadelahi [;] K. Krenzelok, R. Fenoff (ESUW). 1 ♀, Sirena, Osa Pen. [;] VII. 77 Cos. Rica [;] D. H. Janzen (AEIC).

#### Comments.

The smooth mesoscutum and mesopleuron, the white annulus at apex of flagellum, the tubercle on the propodeum just above the hind coxa and the distinct median basal carina of the propodeum are distinctive for this species.

#### Etymology.

The specific name is an arbitrary combination of letters.

**Figure 286.**
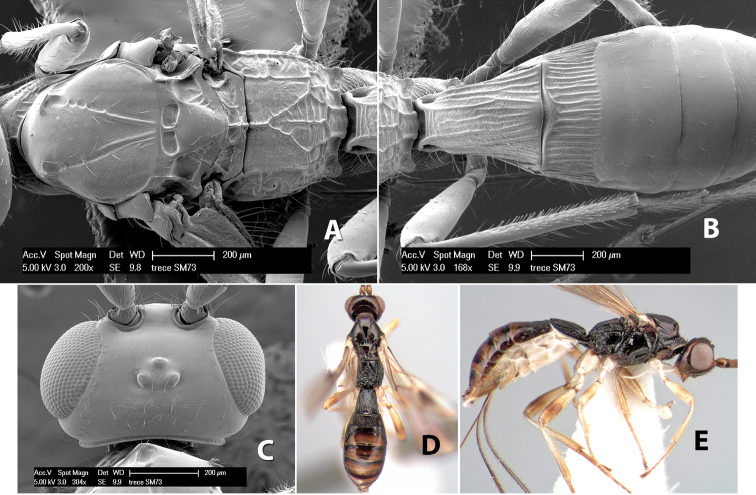
*Heterospilus trece* Marsh, sp. n.: **A–C** paratype **D–E** holotype.

### Heterospilus tres

Marsh sp. n.

http://zoobank.org/11B07656-5A07-4B5B-8159-83522F2EDC27

http://species-id.net/wiki/Heterospilus_tres

Figure 287

#### Female.

Body size: 2.0 mm. Color: head with face and temples honey yellow, frons and vertex light to medium brown; scape yellow without lateral brown stripe; flagellum brown; mesosoma brown, mesoscutum sometimes lighter; metasomal tergum 1 dark brown, remainder of terga light to medium brown; wing veins including stigma brown; legs yellow. Head: vertex smooth; frons smooth; face smooth; temple in dorsal view narrow, width less than 1/2 eye width; malar space equal to 1/4 eye height; ocell-ocular distance twice diameter of lateral ocellus; 21 flagellomeres. Mesosoma: mesoscutal lobes granulate; notauli scrobiculate, meeting posteriorly in triangular costate area; scutellum granulate; prescutellar furrow with 1 cross carina; mesopleuron granulate; precoxal sulcus weakly granulate or smooth, shorter than mesopleuron; venter granulate; propodeum with basal median areas margined, granulate, basal median carina present, areola not distinctly margined, areolar area rugose, lateral areas rugose at extreme apex, smooth over remainder of area. Wings: fore wing vein r shorter than vein 3RSa, vein 1cu-a beyond vein 1M; hind wing vein SC+R present, vein M+CU shorter than vein 1M. Metasoma: first tergum longitudinally costate, length greater than apical width; second tergum longitudinally costate; anterior transverse groove present, sinuate; posterior transverse groove present; third tergum costate basally, smooth or rarely granulate apically; terga 4–7 smooth, rarely weakly granulate; ovipositor as long as metasoma.

#### Holotype female.

Top label (white, printed) - COSTA RICA-Heredia Prov. [;] La Selva Biological Station [;] 10°26'N, 84°01'W, 100m [;] Canopy fogging 32 [;] 3.xi.1994 [;] Project ALAS (FVK32); second label (red, partially printed and hand written) - HOLOTYPE [;] Heterospilus [;] tres [;] P. Marsh. Deposited in ESUW.

#### Paratypes.

2 ♀♀, same data as holotype with dates of 20.x.1994 and 19.x.1994 and Canopy fogging 26 and 27 (ESUW).

#### Comments.

The long ovipositor, the single cross carina in the prescutellar furrow and the sinuate anterior transverse groove of metasomal tergum 2 are distinctive for this species.

#### Etymology.

The specific name is an arbitrary combination of letters.

**Figure 287.**
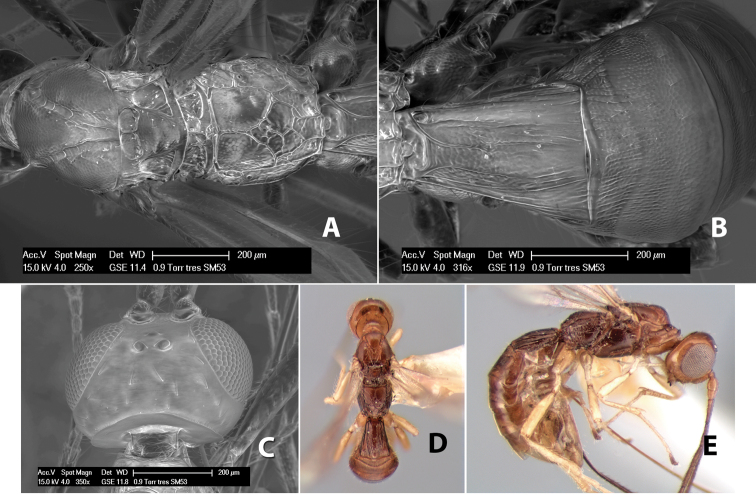
*Heterospilus tres* Marsh, sp. n., holotype.

### Heterospilus trienta

Marsh sp. n.

http://zoobank.org/C6207A8B-98D6-4E30-B647-F3F03195E848

http://species-id.net/wiki/Heterospilus_trienta

Figure 288

#### Female.

Body size: 3.0 mm. Color: head brown, mesosoma and metasoma dark brown; scape yellow without lateral brown stripe; flagellum brown; wing veins including stigma brown; legs yellow. Head: vertex smooth; frons smooth; face striate at least medially; temple in dorsal view narrow, sloping behind eye, width less than 1/2 eye width; malar space equal to 1/4 eye height; ocell-ocular distance greater than 2.5 times diameter of lateral ocellus; 22–23 flagellomeres. Mesosoma: mesoscutal lobes granulate; notauli scrobiculate, meeting posteriorly in wide rectangular costate-rugose area; scutellum weakly granulate or smooth; prescutellar furrow with 3–5 cross carinae; mesopleuron granulate, smooth just above precoxal sulcus; precoxal sulcus scrobiculate, shorter than mesopleuron; venter granulate; propodeum with basal median areas margined, granulate, basal median carina present, short, areola distinctly margined, areolar area rugose, lateral areas entirely rugose. Wings: fore wing vein r shorter than vein 3RSa, vein 1cu-a beyond vein 1M; hind wing vein SC+R present, vein M+CU slightly shorter than vein 1M. Metasoma: first tergum longitudinally costate, length equal to apical width; second tergum longitudinally costate; anterior transverse groove present, straight; posterior transverse groove present; third tergum costate basally, smooth apically; terga 4–7 smooth; ovipositor about 3/4 length of metasoma.

#### Holotype female.

Top label (white, printed) - Costa Rica: Puntarenas [;] Res. Forestal Golfo Dulce [;] 3km. SW Rincon, 10m [;] xii.1992, P. Hanson [;] Malaise, primary forest; second label (red, partially printed and hand written) - HOLOTYPE [;] Heterospilus [;] trienta [;] P. Marsh. Deposited in ESUW.

#### Paratypes.

2 ♀♀, Costa Rica: Puntarenas [;] San Vito, Las Cruces [;] Wilson Botanical Gardens [;] 18–22.iii.1990, 1150m [;] J.S. Noyes (ESUW).

#### Comments.

The rectangular costate-rugose area on the mesoscutum, the narrow temple and the striate face are distinctive for this species.

#### Etymology.

The specific name is an arbitrary combination of letters.

**Figure 288.**
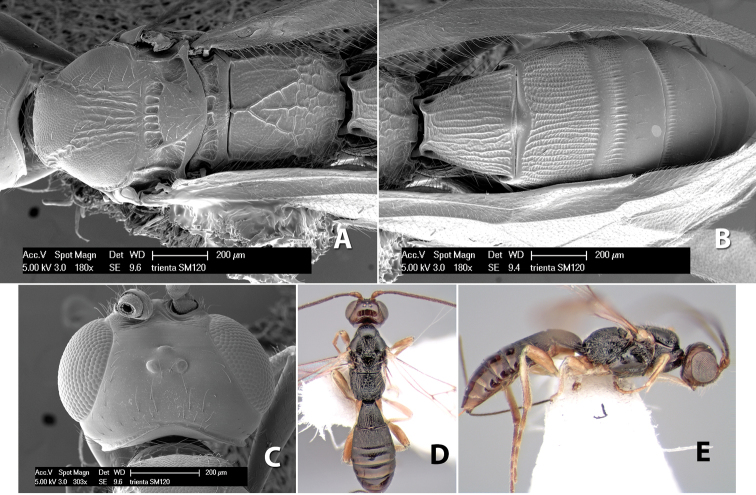
*Heterospilus trienta* Marsh, sp. n.: **A–C** paratype **D–E** holotype.

### Heterospilus tuberculatus

Marsh sp. n.

http://zoobank.org/BAD71B07-E3E7-46CE-97B1-3AC074859F8E

http://species-id.net/wiki/Heterospilus_tuberculatus

Figure 289

#### Female.

Body size: 3.0 mm. Color: head and mesosoma brown, metasomal terga light brown, tergum 2 yellow; scape yellow without lateral brown stripe, flagellum brown with apical 3–5 flagellomeres white; wing veins including stigma brown; legs yellow. Head: vertex smooth; frons smooth; face smooth; temple in dorsal view narrow, sloping behind eye, width less than 1/2 eye width; malar space greater than 1/4 eye height; ocell-ocular distance slightly greater than 2.5 times diameter of lateral ocellus; 19 flagellomeres. Mesosoma: mesoscutal lobes granulate; notauli scrobiculate, meeting posteriorly in triangular costate area; scutellum granulate; prescutellar furrow with 3 cross carinae; mesopleuron granulate; precoxal sulcus scrobiculate, as long as mesopleuron; venter granulate; propodeum with basal median areas margined, granulate, basal median carina present but short, areola not margined, areolar area rugose, lateral areas entirely rugose, apical lateral corners of propodeum distinctly produced into tubercles. Wings: fore wing vein r slightly shorter than vein 3RSa, vein 1cu-a interstitial with vein 1M; hind wing vein SC+R absent, vein M+CU slightly shorter than vein 1M. Metasoma: first tergum longitudinally costate; second tergum weakly costate at base, remainder smooth; anterior transverse groove absent; posterior transverse groove absent; third tergum entirely smooth; terga 4–7 smooth; ovipositor as long as metasomal terga 1 and 2 combined.

#### Holotype female.

Top label (white, printed) - COSTA RICA, Limon [;] 16km W Guápiles [;] 400m, II/1989 [;] col. Paul Hanson; second label (red, partially printed and hand written) - HOLOTYPE [;] Heterospilus [;] tuberculatus [;] P. Marsh. Deposited in ESUW.

#### Paratypes.

Known only from the holotype.

#### Comments.

The distinct tubercles at the apical lateral corners of the propodeum and the precoxal sulcus which is as long as the mesopleuron are distinctive for this species.

#### Etymology.

The specific name is in reference to the distinct tubercles on the propodeum.

**Figure 289.**
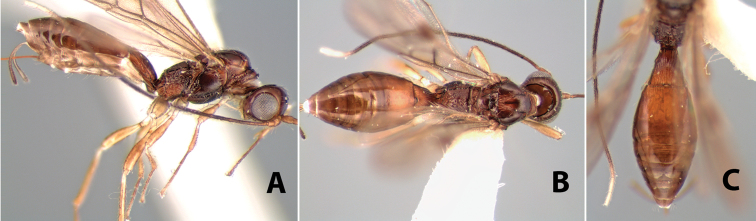
*Heterospilus tuberculatus* Marsh, sp. n., holotype.

### Heterospilus uno

Marsh sp. n.

http://zoobank.org/CEDD13A2-7198-46EF-85DC-8E7376625D8D

http://species-id.net/wiki/Heterospilus_uno

Figure 290

#### Female.

Body size: 3.0 mm. Color: head honey yellow; scape yellow without lateral brown stripe; flagellum brown; mesosoma dark brown; metasomal tergum 1 dark brown, tergum 2 yellow, dark brown medially and laterally, tergum 3 dark brown, yellow medially at base, tergum 4 brown, terga 5–7 honey yellow; wing veins including stigma brown; legs yellow. Head: vertex smooth; frons smooth; face weakly granulate or partially smooth; temple in dorsal view narrow, width less than 1/2 eye width; malar space greater than 1/4 eye height; ocell-ocular distance about 2.5 times diameter of lateral ocellus; 22–24 flagellomeres. Mesosoma: mesoscutal lobes granulate; notauli scrobiculate, meeting posteriorly in triangular rugose or rugose-costate area; scutellum smooth; prescutellar furrow with 1 cross carina; mesopleuron granulate; precoxal sulcus scrobiculate, shorter than mesopleuron; venter granulate; propodeum with basal median areas margined, granulate, basal median carina present, areola distinctly margined, areolar area rugose, lateral areas rugose apically, smooth or granulate basally. Wings: fore wing vein r shorter than vein 3RSa, vein 1cu-a beyond vein 1M; hind wing vein SC+R present, vein M+CU shorter than vein 1M. Metasoma: first tergum longitudinally costate-granulate, length slightly greater than apical width; second tergum longitudinally costate-granulate; anterior transverse groove present, straight; posterior transverse groove absent or weakly present; third tergum costate basally, smooth apically; terga 4–7 smooth; ovipositor longer than metasoma.

#### Holotype female.

Top label (white, printed) - Costa Rica: Puntarenas [;] San Vito, Estac. Biol. [;] Las Alturas, 1500m [;] iii.1992, Paul Hanson; second label (red, partially printed and hand written) - HOLOTYPE [;] Heterospilus [;] uno [;] P. Marsh. Deposited in ESUW.

#### Paratypes.

2 ♀♀, same data as holotype (ESUW).

#### Comments.

The long ovipositor, longer than the metasoma, the single cross carina in the prescutellar furrow and the honey yellow head are distinctive for this species.

#### Etymology.

The specific name is an arbitrary combination of letters.

**Figure 290.**
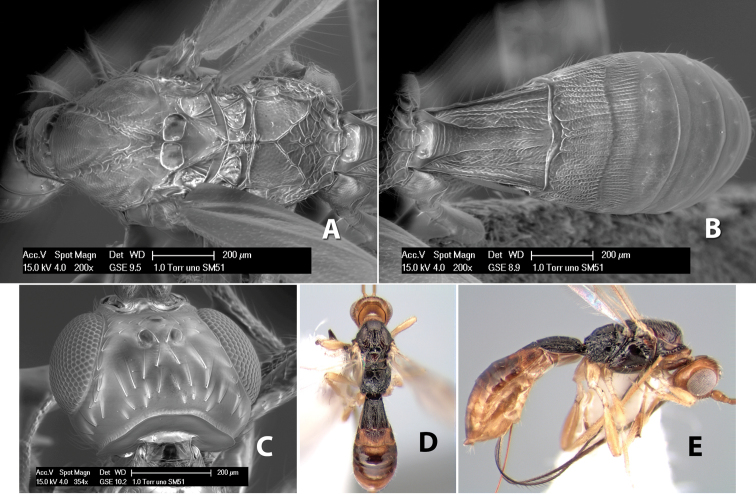
*Heterospilus uno* Marsh, sp. n., holotype.

### Heterospilus veinte

Marsh sp. n.

http://zoobank.org/A656E971-BD1A-47D2-9ED0-929768D1A5B4

http://species-id.net/wiki/Heterospilus_veinte

Figure 291

#### Female.

Body size: 3.5 mm. Color: body brown to dark brown; scape light brown without lateral brown stripe; flagellum brown; wing veins including stigma brown; fore and mid coxae, trochanters and basal 1/5 of femora yellow, apical 4/5 of femora, tibiae and tarsi brown, hind trochanters and basal 1/5 of femur yellow, hind coxa, apical 4/5 of femur, tibia and tarsus brown. Head: vertex smooth; frons smooth; face smooth; temple in dorsal view narrow, sloping behind eye, width less than 1/2 eye width; malar space greater than 1/4 eye height; ocell-ocular distance about 2.25 times diameter of lateral ocellus; 24 flagellomeres. Mesosoma: mesoscutal lobes granulate; notauli scrobiculate, meeting posteriorly in triangular costate area; scutellum smooth; prescutellar furrow with 1 distinct median cross carina and weaker carinae on each side; mesopleuron smooth; precoxal sulcus scrobiculate, shorter than mesopleuron; venter smooth; propodeum with basal median areas margined, granulate, basal median carina present but short, areola not distinctly margined, areolar area rugose, lateral areas entirely rugose. Wings: fore wing vein r shorter than vein 3RSa, vein 1cu-a beyond vein 1M; hind wing vein SC+R present, vein M+CU shorter than vein 1M. Metasoma: first tergum longitudinally costate, length greater than width; second tergum longitudinally costate; anterior transverse groove present, straight; posterior transverse groove weak but present; third tergum costate basally, smooth apically; terga 4–7 smooth; ovipositor about as long as metasoma and half of mesosoma combined.

#### Holotype female.

Top label (white, printed) - Costa Rica: Alajuela [;] R.B. San Ramon [;] 800m, xi-xii.1998 [l] P. Hanson; second label (red, partially printed and hand written) - HOLOTYPE [;] Heterospilus [;] veinte [;] P. Marsh. Deposited in ESUW.

#### Paratypes.

1 ♀, top label - COSTA RICA, Heredia: [;] Est. Biol. La Selva, 50- [;] 150m, 10°26'N, 84°01'W [;] Aug 1995, INBio-OET; second label - 16 Agosto 1995 [;] M/13/434 [;] Bosque secundario (INBC).

#### Comments.

The long ovipositor, the narrow temple and the bicolored legs are distinctive for this species.

#### Etymology.

The specific name is an arbitrary combination of letters.

**Figure 291.**
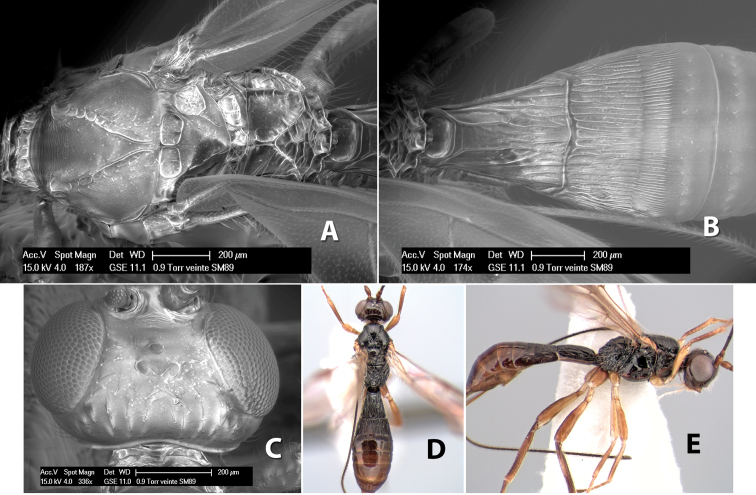
*Heterospilus veinte* Marsh, sp. n., holotype.

### Heterospilus veintidos

Marsh sp. n.

http://zoobank.org/ED046074-9FC1-45E3-A2F1-2B453A67DECB

http://species-id.net/wiki/Heterospilus_veintidos

Figure 292

#### Female.

Body size: 3.0 mm. Color: body dark brown, metasomal tergum 2 somewhat lighter brown, terga 6 yellow; scape honey yellow with darker lateral longitudinal brown stripe; flagellum brown with apical white annulus, apical 3–5 flagellomeres brown; wing veins including stigma brown; legs mostly yellow, coxae and trochanters lighter, hind femur with brown area laterally on apical half. Head: vertex smooth; frons smooth; face smooth; temple in dorsal view narrow, sloping behind eye, width less than 1/2 eye width; malar space equal to 1/4 eye height; ocell-ocular distance about twice diameter of lateral ocellus; 28 flagellomeres. Mesosoma: mesoscutal lobes smooth and polished; notauli scrobiculate, meeting posteriorly in triangular costate area; scutellum smooth; prescutellar furrow with 3 cross carinae; mesopleuron smooth; precoxal sulcus smooth, shorter than mesopleuron; venter smooth; propodeum with basal median areas margined, smooth, basal median carina present, areola distinctly margined, areolar area rugose, lateral areas rugose apically, smooth basally. Wings: fore wing vein r shorter than vein 3RSa, vein 1cu-a beyond vein 1M; hind wing vein SC+R present, vein M+CU shorter than vein 1M. Metasoma: first tergum longitudinally costate, length equal to apical width; second tergum longitudinally costate, width about 4 times length; anterior transverse groove present, slightly sinuate; posterior transverse groove present; third tergum costate on basal ⅓, smooth on apical ⅔; terga 4–7 smooth; ovipositor half as long as metasoma.

#### Holotype female.

Top label (white, printed) - Costa Rica: Puntarenas [;] R.F. Golfo Dulce, 3km. [;] S.W. Rincon, 10m [;] I.1992, P. Hanson; second label (red, partially printed and hand written) - HOLOTYPE [;] Heterospilus [;] veintidos [;] P. Marsh. Deposited in ESUW.

#### Paratypes.

3 ♀♀, S.RosaPark, Guan. [;] C. Rica 5, 12 14 Sep 77 [;] D.H. Janzen [;] Riparian (AEIC). 1 ♀, S.RosaPark, Guan. [;] C. Rica 8 Dec., 76 [;] D. H. Janzen [;] Riparian (AEIC).

#### Comments.

The short metasomal tergum 1, the sinuate anterior transverse groove on tergum 2, the smooth and polished mesoscutum and the flagellum with apical white annulus are distinctive for this species.

#### Etymology.

The specific name is an arbitrary combination of letters.

**Figure 292.**
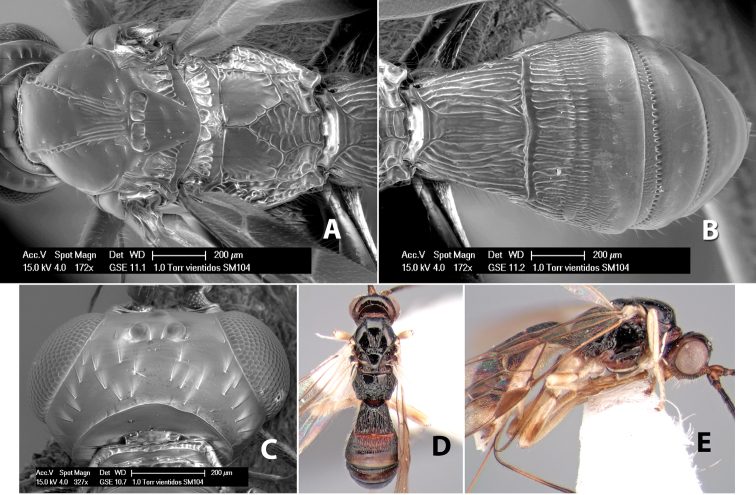
*Heterospilus veintidos* Marsh, sp. n., holotype.

### Heterospilus veintitres

Marsh sp. n.

http://zoobank.org/46E7CF19-5855-4F46-A307-1AEAE06FF76D

http://species-id.net/wiki/Heterospilus_veintitres

Figure 293

#### Female.

Body size: 3.5 mm. Color: body dark brown; scape and flagellum brown; wing veins including stigma brown; legs yellow. Head: vertex smooth; frons smooth; face smooth; temple in dorsal view narrow, sloping behind eye, width less than 1/2 eye width; malar space greater than 1/4 eye height; ocell-ocular distance greater than 2.5 times diameter of lateral ocellus; 28 flagellomeres. Mesosoma: mesoscutal lobes smooth; notauli weakly scrobiculate anteriorly, smooth posteriorly, meeting posteriorly in unsculptured area except for 2 converging carinae; scutellum smooth; prescutellar furrow with 1 cross carina; mesopleuron smooth; precoxal sulcus weakly scrobiculate, shorter than mesopleuron; venter smooth; propodeum with basal median areas margined, smooth, basal median carina present, short, areola distinctly margined, areolar area rugose, lateral areas entirely rugose. Wings: fore wing vein r shorter than vein 3RSa, vein 1cu-a beyond vein 1M; hind wing vein SC+R present, vein M+CU shorter than vein 1M. Metasoma: first tergum longitudinally costate, length greater than apical width; second tergum longitudinally costate; anterior transverse groove present, straight; posterior transverse groove present; third tergum costate basally, granulate apically; terga 4–7 weakly granulate; ovipositor equal to half length of metasoma.

#### Holotype female.

Top label (white, printed) - COSTA RICA: Guanac. [;] Estac. Mengo, S.W. [;] Volcan Cacao, 1100m [;] IX-X 1989; second label (red, partially printed and hand written) - HOLOTYPE [;] Heterospilus [;] veintitres [;] P. Marsh. Deposited in ESUW.

#### Paratypes.

1 ♀, Costa Rica: Heredia [;] Braulio Carrillo N.P. [;] 250–500m IV.10.85 [;] Henri Goulet (AEIC).

#### Comments.

The granulate metasomal terga 4–6, the two carinae where notauli meet and the single cross carina in the prescutellar furrow are distinctive for this species.

#### Etymology.

The specific name is an arbitrary combination of letters.

**Figure 293.**
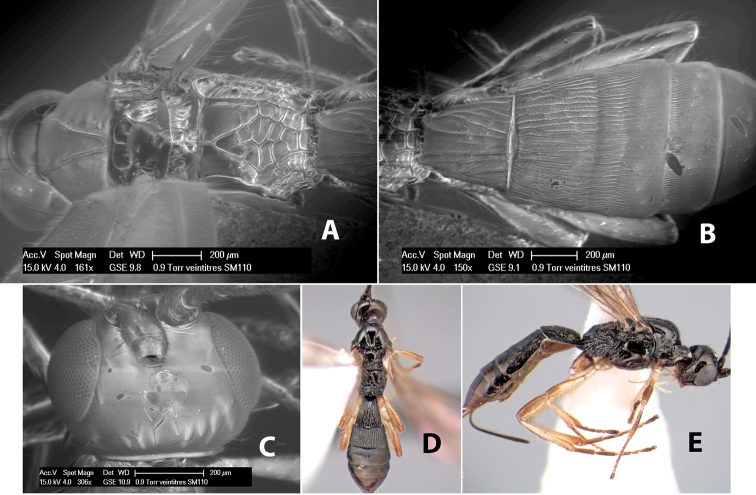
*Heterospilus veintitres* Marsh, sp. n., holotype.

### Heterospilus veintiuno

Marsh sp. n.

http://zoobank.org/4CE19719-874E-4217-8DEE-CF14A50FD6AD

http://species-id.net/wiki/Heterospilus_veintiuno

Figure 294

#### Female.

Body size: 3.5–4.0 mm. Color: head with vertex and frons dark brown, face, temple and eye orbits light brown or honey yellow; scape light brown without lateral brown stripe; flagellum brown; mesosoma brown, pronotum occasionally lighter; wing veins including stigma brown; legs yellow; metasoma dark brown, apical terga lighter brown or honey yellow. Head: vertex smooth; frons weakly striate or smooth; face rugose; temple in dorsal view narrow but slightly bulging behind eye, width less than 1/2 eye width; malar space equal to 1/4 eye height; ocell-ocular distance 1.5 times diameter of lateral ocellus; 27 flagellomeres. Mesosoma: mesoscutal lobes granulate; notauli scrobiculate, meeting posteriorly in triangular rugose-costate area; scutellum weakly granulate; prescutellar furrow with 3 cross carinae; mesopleuron smooth; precoxal sulcus weakly scrobiculate, shorter than mesopleuron; venter smooth; propodeum with basal median areas margined, weakly granulate, basal median carina absent, areola not margined, areolar area areolate-rugose, lateral areas entirely rugose. Wings: fore wing vein r shorter than vein 3RSa, vein 1cu-a beyond vein 1M; hind wing vein SC+R present, vein M+CU as long as vein 1M. Metasoma: first tergum longitudinally costate, length greater than apical width; second tergum longitudinally costate; anterior transverse groove present, straight; posterior transverse groove present; third tergum costate basally, smooth apically; terga 4–7 smooth; ovipositor longer than metasoma.

#### Holotype female.

Top label (white, printed) - COSTA RICA-Heredia Prov. [;] Las Selva Biological Station [;] 10°26'N, 84°01'W, 100m [;] Canopy fogging 28 [;] 22.x.1994 [;] Project ALAS (FPM28); second label (red, partially printed and hand written) - HOLOTYPE [;] Heterospilus [;] veintiuno [;] P. Marsh. Deposited in ESUW.

#### Paratypes.

2 ♀♀, same data as holotype except: Canopy fogging 20 and 29; dates of 23.x.1994, 9.x.1994 (ESUW).

#### Comments.

The rugose face, short ocell-ocular distance and the long ovipositor are distinctive for this species.

#### Etymology.

The specific name is an arbitrary combination of letters.

**Figure 294.**
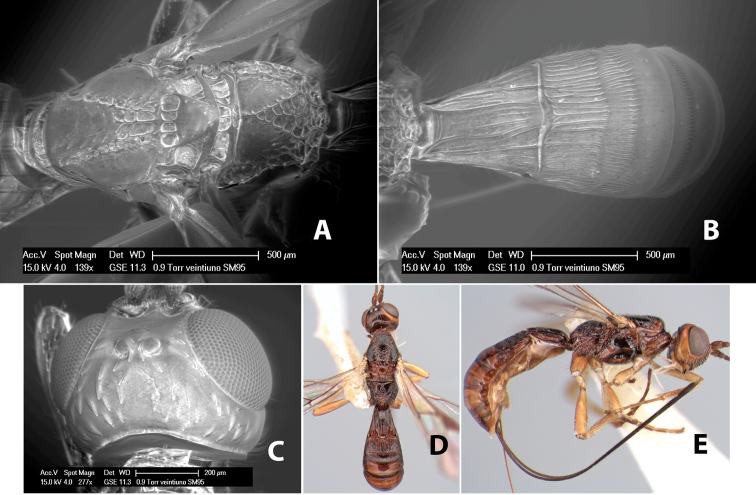
*Heterospilus veintiuno* Marsh, sp. n., holotype.

### Heterospilus wahli

Marsh sp. n.

http://zoobank.org/5CB44550-FC2A-4351-8C93-BC41662B0C93

http://species-id.net/wiki/Heterospilus_wahli

Figure 295

#### Female.

Body size: 2.0 mm. Color: head with vertex brown, face honey yellow, mesosoma and metasoma brown to dark brown; scape yellow without lateral brown stripe, flagellum brown with apical 5–7 flagellomeres white; wing veins including stigma brown; legs yellow. Head: vertex smooth; frons smooth; face smooth; temple in dorsal view narrow, sloping behind eye, width less than 1/2 eye width; malar space greater than 1/4 eye height; ocell-ocular distance greater than 2.5 times diameter of lateral ocellus; 14–16 flagellomeres. Mesosoma: mesoscutal lobes granulate; notauli scrobiculate, meeting at scutellum in triangular costate area; scutellum weakly granulate; prescutellar furrow with 1 cross carina; mesopleuron granulate; precoxal sulcus weakly scrobiculate, shorter than mesopleuron; venter granulate; propodeum with basal median areas margined, granulate, basal median carina present, areola distinctly margined, areolar area rugose, lateral areas rugose posteriorly, small granulate area anteriorly. Wings: fore wing vein r slightly shorter than vein 3RSa, vein 1cu-a slightly beyond vein 1M; hind wing vein SC+R absent, vein M+CU equal in length to vein 1M. Metasoma: first tergum longitudinally costate, length greater than apical width; second tergum longitudinally costate; anterior transverse groove present, straight; posterior transverse groove present; third tergum entirely smooth; terga 4–7 smooth; ovipositor as long as metasomal terga 1 and 2 combined.

#### Holotype female.

Top label (white, printed) - COSTA RICA: [;] Puntar. Golfo Dulce [;] 24km W Piedras Blancas [;] 200m, vi-viii 1989 [;] Hanson; second label (red, partially printed and hand written) - HOLOTYPE [;] Heterospilus [;] wahli [;] P. Marsh. Deposited in ESUW.

#### Paratypes.

1 ♀, same data as holotype (ESUW). 2 ♀♀, Costa Rica: Puntarenas [;] R. F. Golfo Dulce, 24km. [;] W. Piedras Blancas, 200m [;] I.1993, P. Hanson (ESUW). 2 ♀♀, COSTA RICA: Puntarenas [;] RF Golfo Dulce el 200m [;] 24km W Piedras Blancas [;] P. Hanson ix.1992 and vi.1993 (TAMU).

#### Comments.

The flagellum with an apical white annulus, the smooth metasomal tergum 3 and hind wing vein M+CU being equal in length to vein 1M are distinctive for this species.

#### Etymology.

Named for my friend and colleague, David Wahl.

**Figure 295.**
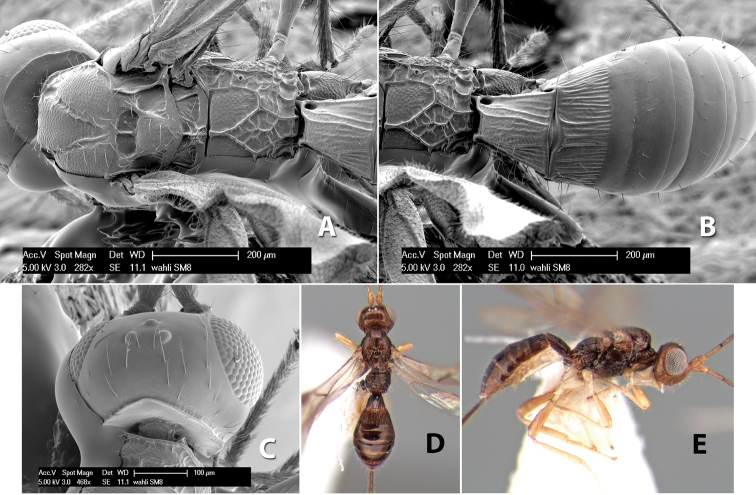
*Heterospilus wahli* Marsh, sp. n.: **A–C** paratype **D–E** holotype.3

### Heterospilus washingtoni

Marsh sp. n.

http://zoobank.org/2F0B383C-CE09-4E48-A216-48F1666F8ED3

http://species-id.net/wiki/Heterospilus_washingtoni

Figures 296 , 297

#### Female.

Body size: 4.0–5.0 mm. Color: body entirely yellow; scape brown; flagellum brown; wing veins including stigma brown; legs yellow. Head: vertex smooth; frons smooth; face rugose, at least medially; temple in dorsal view narrow, width equal to 1/2 eye width; malar space greater than 1/4 eye height; ocell-ocular distance slightly greater than twice diameter of lateral ocellus; 33–37 flagellomeres. Mesosoma: mesoscutal lobes smooth; notauli scrobiculate, meeting posteriorly in costate-rugose rectangular area; scutellum smooth; prescutellar furrow with 1 cross carina; mesopleuron smooth; precoxal sulcus smooth, shorter than mesopleuron; venter smooth; propodeum with basal median areas margined, smooth, basal median carina absent, areola weakly margined or not margined, areolar area rugose, lateral areas entirely rugose. Wings: fore wing vein r shorter than vein 3RSa, vein 1cu-a beyond vein 1M; hind wing vein SC+R present, vein M+CU shorter than vein 1M. Metasoma: first tergum longitudinally costate, distinct transverse carina medially at base, length equal to apical width; second tergum longitudinally costate; anterior transverse groove present, straight; posterior transverse groove present; third tergum costate basally, smooth apically; terga 4–7 smooth; ovipositor longer than metasoma.

#### Holotype female.

Top label (white, printed) - COSTA RICA-Heredia Prov. [;] La Selva Biological Station [;] 10°26'N, 84°01'W, 100m [;] Canopy fogging 19 [;] 8.x.1994 [;] Project ALAS(FVK19); second label (red, partially printed and hand written) - HOLOTYPE [;] Heterospilus [;] washingtoni [;] P. Marsh. Deposited in ESUW.

#### Paratypes.

1 ♀, same data as holotype except: Canopy fogging 20; date of 9.x.1994 (ESUW). 1 ♀, top label - Costa Rica: Guanacaste [;] Santa Rosa Natl. Park [;] 300m, ex. Malaise trap [;] Site #: H-1-O [;] Dates: 29.xi–20.xii 1996 [;] I.D. Gauld & D. Janzen; second label - [H] open regenerating [;] woodland <10 years old [;] [O] in clearing, fully [;] isolated part of day (ESUW). 1 ♀, S.RosaPark, Guan. [;] C. Rica 5 Nov 77 [;] D.H. Janzen [;] Dry Hill (AEIC).

#### Comments.

The large yellow body, smooth mesosoma, single cross carina in prescutellar furrow and the long ovipositor are distinctive for this species.

#### Etymology.

Named for the father of the United States and our first president, George Washington.

**Figure 296.**
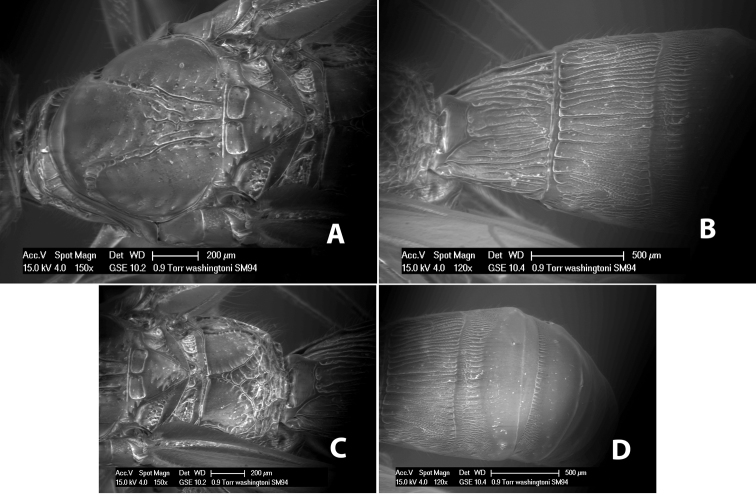
*Heterospilus washingtoni* Marsh, sp. n., holotype.

**Figure 297.**
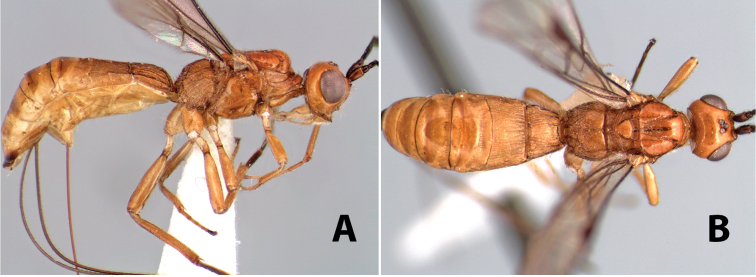
*Heterospilus washingtoni* Marsh, sp. n., holotype.

### Heterospilus wildi

Marsh sp. n.

http://zoobank.org/D2298422-857C-4AFC-9E7F-017937B4D3C5

http://species-id.net/wiki/Heterospilus_wildi

Figure 298

#### Female.

Body size: 2.0–2.5 mm. Color: body dark brown; scape yellow without lateral brown stripe; flagellum brown, usually with apical white annulus of 5–7 flagellomeres, occasionally apical 3–5 flagellomeres brown; wing veins including stigma brown; legs yellow. Head: vertex smooth and shining; frons smooth; face smooth; temple in dorsal view narrow, width equal to 1/2 eye width; malar space greater than 1/4 eye height; ocell-ocular distance greater than 2.5 times diameter of lateral ocellus; 18–22 flagellomeres. Mesosoma: mesoscutal lobes smooth and shining; notauli weakly scrobiculate, meeting posteriorly in triangular costate area; scutellum smooth; prescutellar furrow with 3–5 cross carinae; mesopleuron smooth; precoxal sulcus weakly scrobiculate and very short, often represented by single large pit; venter smooth; propodeum with basal median areas margined, granulate or rugose, basal median carina present but very short and often nearly absent, areola not margined, areolar area areolate-rugose, lateral areas entirely rugose. Wings: fore wing vein r shorter than vein 3RSa, vein 1cu-a slightly beyond vein 1m or occasionally interstitial; hind wing vein SC+R absent, vein M+CU shorter than vein 1M. Metasoma: first tergum longitudinally costate, length greater than apical width, rarely nearly equal to apical width; second tergum longitudinally costate; anterior transverse groove present, straight; posterior transverse groove present; third tergum entirely smooth except for costate transverse groove; terga 4–7 smooth; ovipositor half as long as metasoma.

#### Holotype female.

Top label (white, partially printed and hand written) - Costa Rica: Guanacaste [;] Santa Rosa Natl. Park [;] 300m, ex. Malaise trap [;] Site #: BH-9-O [;] Dates: 26.vii–14.viii.1986 [;] I.D. Gauld & D. Janzen; second label (white, printed) - [BH] Bosque Humedo [;] mature evergreen dry forest [;] [O] in clearing, fully [;] isolated part of day; third label (red, partially printed and hand written) - HOLOTYPE [;] Heterospilus [;] wildi [;] P. Marsh. Deposited in ESUW.

#### Paratypes.

5 ♀♀, same data as holotype with additional site # BH-11-O and dates of 18.x–8.xi.1986, 14.viii–6.ix.1986, 6–27.ix.1986, 24.v–14.vi.1986 (ESUW). 4 ♀♀, top label - Costa Rica: Guanacaste [;] Santa Rosa Natl. Park [;] 300m, ex. Malaise trap [;] Site: BH-12-C and blank [;] Dates: 14.vi–5.vii.1986, 5–26.vii.1986 and 16.xi–7.xii.1985 [;] I.D. Gauld & D. Janzen; second label - [BH] Bosque Humedo [;] mature evergreen dry forest [;] [C] more or less fully [;] shaded as possible (ESUW). 1 ♀, top label - Costa Rica: Guanacaste [;] Santa Rosa Natl. Park [;] 300m, ex. Malaise trap [;] Site: blank [;] Dates: 16.xi–7.xii.1985 [;] I.D. Gauld & D. Janzen; second label - [SE] Bosque San Emilio [;] 50yr old deciduous forest [;] [C] more or less fully [;] shaded as possible (ESUW). 1 ♀, top label - Costa Rica: Guanacaste [;] Santa Rosa Natl. Park [;] 300m, ex. Malaise trap [;] Site: blank [;] Dates: 26.x–16.xi.1985 [;] I.D. Gauld & D. Janzen; second label - [SE] Bosque San Emilio [;] 50yr old deciduous forest [;] [O] in clearing, fully [;] isolated part of day (ESUW). 2 ♀♀, top label - Costa Rica: Guanacaste [;] Santa Rosa Natl. Park [;] 300m, ex. Malaise trap [;] Site: H-1-O and H-2-O [;] Dates: 14.vi–5.vii.1986 and 20.xii.86–10.i.1987 [;] I.D. Gauld & D. Janzen; second label - [H] open regenerating [;] woodland <10 years old [;] [O] in clearing, fully [;] isolated part of day (ESUW). 1 ♀, Costa Rica: Guanacaste [;] Santa Rosa Natl. Park [;] regenerating woodland [;] >10yr. old, 300 meters [;] 6–27.ix.1986, I.D.Gauld [;] ex. Townes Malaise H3-O [;] direct sun daily, wet (ESUW). 1 ♀, Costa Rica: Guanacaste [;] Santa Rosa Natl. Park [;] 300m, ex. Malaise trap [;] Site: blank [;] Dates: 23.iii–13.iv.1986 [;] I.D. Gauld & D. Janzen (ESUW). 1 ♀, top label - Costa Rica: Guanacaste [;] Santa Rosa National Pk. [;] 300m, Malaise, Ian Gauld [;] 27,ix–18.x.1986; second label - Bosque San Emilio [;] 50 yr. Old deciduous [;] Forest. Full Shade; third label - SE-8-C [;] 27.ix–18.x.86 (ESUW). 1 ♀, Costa Rica: Puntarenas [;] R.F. Golfo Dulce, 5km. [;] w. Piedras Blancas, 100m [;] vi–vii.1991, P. Hanson [;] Malaise, second growth (ESUW). 1 ♀, Costa Rica, Puntarenas [;] Pen. Osa, 5km. N. [;] Puerto Jimenez, 10m [;] V-VII-1991, P. Hanson (ESUW). 1 ♀, COSTA RICA: Puntarenas [;] Reserva Forestal Golfo Dulce [;] 3km southwest of Rincon [;] 10m, July 1991, P. Hanson [;] primary forest, Malaise trap (ESUW). 3 ♀♀, Costa Rica: Puntarenas, ACO [;] Golfito, R.F. Golfo Dulce [;] Est. Agujas, 250–350m [;] 4–20.vi.1999, J. Azofeifa [;] L.S.276750-526550 #52746 [;] Amarilla (ESUW). 1 ♀, Costa Rica: Puntarenas [;] Golfo Dulce, 24km W. [;] Piedras Blancas, 200m [;] xii.1991, Paul Hanson (ESUW). 2 ♀♀, Costa Rica: Puntarenas [;] R.F.Golfo Dulce, 3km [;] SW Rincon, 10m [;] Malaise-primary forest [;] viii.1991, P. Hanson (ESUW). 1 ♀, Costa Rica: Cartago [;] Turrialba la Isabel [;] 650m, Café, ix.1994 [;] M. Cerda & P. Hanson (ESUW). 2 ♀♀, Costa Rica, Cartago [;] Turrialba, CATIE [;] 14–15 March 1990 [;] 700m, J.S. Noyes (ESUW). 1 ♀, Costa Rica: Limon, ACLAC [;] Central, R.B. Hitoy Cerere [;] Est. Hitoy Cerera, Send. Bobocara [;] 300m, Malaise trap [;] 17.ix–10.x.1999, F. Umana [;] L.N. 184250-641800 #53495 (ESUW). 1 ♀, Costa Rica: Heredia [;] Est. Biol. La Selva [;] 50–150m, 10.26 N [;] 84.01 W, Aug. 1992 (ESUW). 1 ♀, Costa Rica: Heredia [;] 3km. S. Puerto Viejo [l] OTS - La Selva, 100m [;] 16–30 IX. 1992 [;] P. Hanson (ESUW).

#### Comments.

The smooth and shining vertex, the short precoxal sulcus and the flagellum with an apical white annulus are distinctive for this species.

#### Etymology.

Named for Alex Wild, whose head is as bald and shining as this species, in recognition of his work on the molecular analyses and imaging of the species in this study.

**Figure 298.**
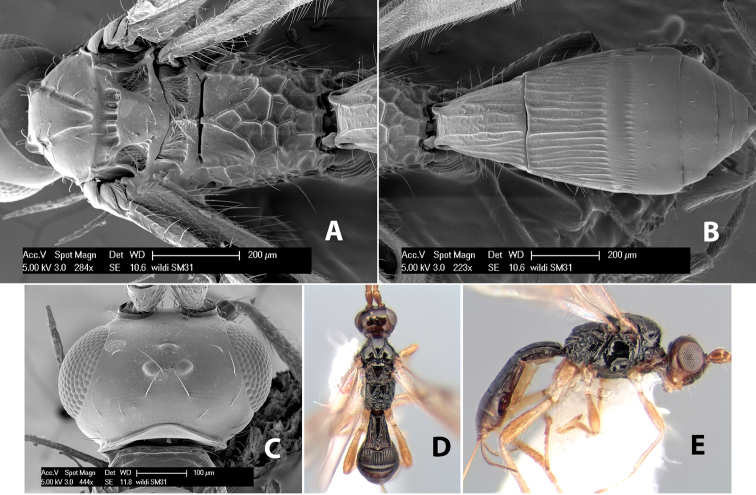
*Heterospilus wildi* Marsh, sp. n.: **A–C** paratype **D–E** holotype.

### Heterospilus xerxes

Marsh sp. n.

http://zoobank.org/FED89683-3A4C-445F-8A9C-2F00FD15D803

http://species-id.net/wiki/Heterospilus_xerxes

Figure 299

#### Female.

Body size: 2.0–2.5 mm. Color: body brown to dark brown, metasomal tergum 2 honey yellow, terga 3–5 brown, tergum 6 yellow; scape yellow without lateral brown stripe; flagellum brown with basal flagellomeres yellow, apical 3–5 flagellomeres white; wing veins including stigma brown; legs yellow. Head: vertex smooth; frons smooth; face smooth; temple in dorsal view narrow, sloping behind eye, width about equal to 1/2 eye width; malar space greater than 1/4 eye height; ocell-ocular distance slightly greater than 2.5 times diameter of lateral ocellus; 14–21 flagellomeres. Mesosoma: mesoscutal lobes granulate; notauli scrobiculate, meeting posteriorly in triangular costate area; scutellum smooth; prescutellar furrow with 3 cross carinae; mesopleuron smooth; precoxal sulcus weakly scrobiculate or smooth, shorter than mesopleuron; venter smooth; propodeum with basal median areas margined, weakly granulate or partially smooth, basal median carina present, areola distinctly margined, areolar area rugose, lateral areas entirely rugose. Wings: fore wing vein r shorter than vein 3RSa, vein 1cu-a slightly beyond vein 1M or rarely interstitial; hind wing vein SC+R present, vein M+CU shorter than vein 1M. Metasoma: first tergum longitudinally costate, length greater than apical width; second tergum longitudinally costate; anterior transverse groove weak or nearly absent; posterior transverse groove weak or absent; third tergum entirely smooth; terga 4–7 smooth; ovipositor half as long as metasoma.

#### Holotype female.

Top label (white, printed) - Costa Rica: Cartago [;] La Cangreja, 1950m [;] ix-xii.1992 [;] P. Hanson; second label (red, partially printed and hand written) - HOLOTYPE [;] Heterospilus [;] xerxes [;] P. Marsh. Deposited in ESUW.

#### Paratypes.

3 ♀♀, Costa Rica: Puntarenas [;] San Vito, Estac. Biol. [;] Las Alturas, 1500m [;] xii.1991, ii.1992 and iii.1992, Paul Hanson (ESUW). 1 ♀, Costa Rica: Guanacaste [;] Est. Cacao, 1000–1150m [;] viii.1996, M. Pereira [;] L.N. 323150-375500 #47561 [;] Malaise trap (ESUW). 1 ♀, Costa Rica: Guanacaste, ACT [;] Bagaces, P.N. Palo Verde [;] Sec. P. Verde, 0–50m, Amarilla [;] 200m N de Estacion [;] 4–11.xi.1999, J. Jimenez [;] L.N. 323150-38020 #54000 (ESUW). 1 ♀, top label - Costa Rica: Guanacaste [;] Santa Rosa Natl. Park [;] 300m, ex Malaise trap [;] Site #: (blank) [;] Dates: 18.x–8.xi.1986 [;] I.D. Gauld & D. Janzen; second label - [H] open regenerating [;] woodland <10 years old [;] [C] more or less fully [;] shaded as possible (ESUW).

#### Comments.

The flagellum with apical white flagellomeres, the granulate mesoscutum and the smooth metasomal tergum 3 are distinctive for this species.

#### Etymology.

Named for Xerxes, a king of ancient Persia.

**Figure 299.**
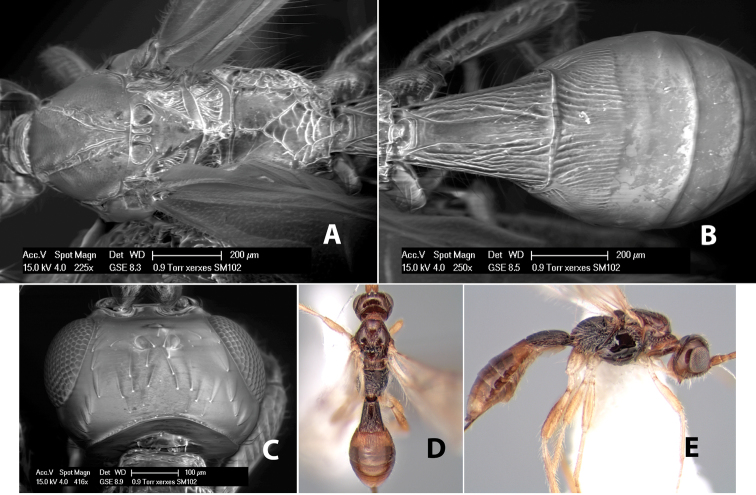
*Heterospilus xerxes* Marsh, sp. n., holotype.

### Heterospilus ypsilon

Marsh sp. n.

http://zoobank.org/5FA85BB8-F454-4088-8519-A7785613267B

http://species-id.net/wiki/Heterospilus_ypsilon

Figure 300

#### Female.

Body size: 3.5 mm. Color: head dark brown; scape honey yellow without lateral brown stripe; flagellum brown with apical 5–7 flagellomeres white; mesosoma dark brown; wing veins brown, stigma brown with apex yellow; legs yellow; metasomal tergum 1 dark brown, tergum 2 usually yellow, rarely brown, terga 3–4 brown, remainder of terga yellow. Head: vertex smooth; frons smooth; face smooth; temple in dorsal view narrow, sloping behind eye, width less than 1/2 eye width; malar space greater than 1/4 eye height; ocell-ocular distance slightly greater than twice diameter of lateral ocellus; 25–28 flagellomeres. Mesosoma: mesoscutal lobes weakly granulate or partially smooth; notauli scrobiculate, meeting posteriorly in rectangular costate area; scutellum smooth; prescutellar furrow with 5 cross carina; mesopleuron smooth; precoxal sulcus scrobiculate, extending to posterior margin of mesopleuron by weak carinae; venter smooth; propodeum with basal median areas margined, weakly granulate or smooth, basal median carina present but short, areola distinctly margined, areolar area rugose, lateral areas entirely rugose. Wings: fore wing vein r shorter than vein 3RSa, vein 1cu-a beyond vein 1M; hind wing vein SC+R present, vein M+CU shorter than vein 1M. Metasoma: first tergum longitudinally costate, length greater than apical width; second tergum longitudinally costate; anterior transverse groove present, straight; posterior transverse groove weak or absent; third tergum smooth entirely; terga 4–7 smooth; ovipositor about 3/4 length of metasoma.

#### Holotype female.

Top label (white, printed) - COSTA RICA: Puntar [;] Golfo Dulce. 10km W [;] Piedras Blancas, 100m [;] VI-VIII 1989, Hanson; second label (red, partially printed and hand written) - HOLOTYPE [;] Heterospilus [;] ypsilon [;] P. Marsh. Deposited in ESUW.

#### Paratypes.

1 ♀, Costa Rica: Heredia [;] 3km S. Puerto Viejo [;] OTS, La Selva, 100m [;] 1–15 ix 1992, P. Hanson [;] huertos Malaise trap [;] set by G. Wright (ESUW).

#### Comments.

The smooth metasomal tergum 3, the white apical annulus on the flagellum and the 5 cross carinae in the prescutellar furrow are distinctive for this species.

#### Etymology.

The specific name is the 20th letter of the Greek alphabet.

**Figure 300.**
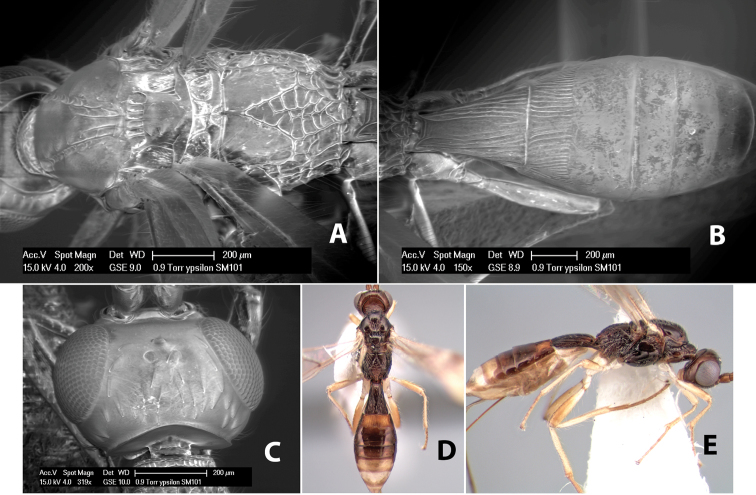
*Heterospilus ypsilon* Marsh, sp. n., holotype.

### Heterospilus zeus

Marsh sp. n.

http://zoobank.org/65B3D74E-9208-4DA9-9D7A-6D7D938817E8

http://species-id.net/wiki/Heterospilus_zeus

Figure 301

#### Female.

Body size: 3.5–5.0 mm. Color: head yellow except vertex brown to dark brown; scape brown without lateral brown stripe; flagellum brown; mesosoma dark brown; wing veins including stigma brown; fore and mid legs yellow, hind coxa and trochanters yellow, hind femur and tibia yellow on basal half and brown on apical half, hind tarsus brown; metasomal terga dark brown, terga 3–7 yellow laterally. Head: vertex smooth; frons smooth posteriorly, depressed and striate anteriorly behind antennal sockets; face rugose with median raised smooth area; temple in dorsal view narrow, width less than 1/2 eye width; malar space equal to 1/4 eye height; ocell-ocular distance about twice diameter of lateral ocellus; 35–42 flagellomeres. Mesosoma: mesoscutal lobes granulate; notauli scrobiculate, meeting posteriorly in triangular costate-rugose area; scutellum weakly granulate; prescutellar furrow with 5 cross carinae; mesopleuron smooth; precoxal sulcus smooth, shorter than mesopleuron; venter smooth; propodeum with basal median areas margined, granulate, basal median carina absent, areola not distinctly margined, areolar area rugose, lateral areas rugose apically, smooth or weakly granulate basally. Wings: fore wing vein r shorter than vein 3RSa, vein 1cu-a beyond vein 1M; hind wing vein SC+R present, vein M+CU shorter than vein 1M. Metasoma: first tergum longitudinally costate, somewhat rugose medially, with several cross carinae medially at base, length greater than apical width; second tergum longitudinally costate; anterior transverse groove present, sinuate; posterior transverse groove present; third tergum costate basally, weakly granulate apically; terga 4–6 weakly granulate or smooth apically, granulate basally; ovipositor longer than metasoma.

#### Holotype female.

Top label (white, printed) - COSTA RICA-Heredia Prov. [;] Las Selva Biological Station [;] 10°26'N, 84°01'W, 100m [;] Canopy fogging 29 [;] 23.x.1994 [;] Project ALAS (FPM29); second label (red, partially printed and hand written) - HOLOTYPE [;] Heterospilus [;] zeus [;] P. Marsh. Deposited in ESUW.

#### Paratypes.

1 ♀, COSTA RICA: Puntar [;] Golfo Dulce, 10km W [;] Piedras Blancas, 100m [;] VI-VIII 1989, Hanson (ESUW).

#### Comments.

The long antennae and ovipositor, the sinuate anterior transverse groove on metasomal tergum 2 and the weakly granulate metasomal terga 4–6 are distinctive for this species.

#### Etymology.

Named for the Greek god, Zeus.

**Figure 301.**
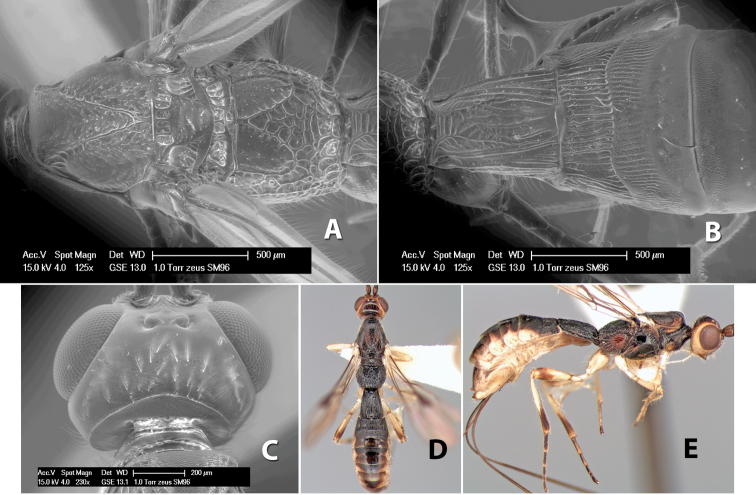
*Heterospilus zeus* Marsh, sp. n., holotype.

### Heterospilus zurquiensis

Marsh sp. n.

http://zoobank.org/3643BA17-22CE-42E6-B7B6-BA6FDFC35FF3

http://species-id.net/wiki/Heterospilus_zurquiensis

Figure 302

#### Female.

Body size: 2.0 mm. Color: head dark brown; scape yellow with weak, lateral longitudinal brown stripe, flagellum brown with apical 4–6 flagellomeres white, apical one often dark; mesosoma dark brown, propodeum somewhat lighter; metasomal terga brown, tergum 1 usually and tergum often yellow; wing veins including stigma brown; legs yellow. Head: vertex smooth; frons smooth; face smooth; temple in dorsal view narrow, sloping behind eye, width equal to 1/2 eye width; malar space greater than 1/4 eye height; ocell-ocular distance greater than 2.5 times diameter of lateral ocellus; 16–17 flagellomeres. Mesosoma: mesoscutal lobes weakly granulate or smooth, at least partially; notauli smooth, meeting posteriorly in unsculptured area; scutellum smooth; prescutellar furrow with 3 cross carinae; mesopleuron granulate; precoxal sulcus smooth, shorter than mesopleuron; venter weakly granulate; propodeum with basal median areas margined, granulate, basal median carina present, areola not margined, areolar area broadly rugose, lateral areas entirely rugose. Wings: fore wing vein r shorter than vein 1M, vein 1cu-a beyond vein 1M; hind wing vein SC+R absent, vein M+CU shorter than vein 1M. Metasoma: first tergum longitudinally costate, length greater than apical width; second tergum longitudinally costate; anterior transverse groove present, straight; posterior transverse groove present; third tergum entirely smooth; terga 4–7 smooth; ovipositor shorter than metasomal tergum 1.

#### Holotype female.

Top label (white, printed) - COSTA RICA: San Jose [;] Zurqui de Moravia [;] 1600m, I-II 1989 [;] P. Hanson & I. Gauld; second label (red, partially printed and hand written) - HOLOTYPE [;] Heterospilus [;] zurquiensis [;] P. Marsh. Deposited in ESUW.

#### Paratypes.

1 ♀, top label - Costa Rica: Guanacaste [;] 9km S. Santa Cecilia [;] Estacion Pitilia, 700m [;] vi.1996, Malaise trap; second label - C. Moraga & P. Rios [;] L.N. 330200-380200 [;] #47562 (ESUW). 2 ♀♀, Costa Rica: San Jose [;] Zurqui de Moravia [;] 1600m, vi.1992 and III-1996, col. P. Hanson (ESUW). 1 ♀, COSTA RICA: San Jose [;] P.N. Braulio Carillo [;] 9.5km E tunnel, 1000m [;] iii-iv 1990 P. Hanson (ESUW). 1 ♀, COSTA RICA: [;] Guanacaste [;] Estac. Mengo [;] SW Volcan Cacao [;] 1100m, 1988–1989 (ESUW). 1 ♀, COSTA RICA: San Jose [;] Ciudad Colon, 800m [;] xii 1989-i 1990 [;] Louis Fournier (ESUW). 1 ♀, Costa Rica: Guanacaste [;] Est. Cacao, 1000–1150m [;] viii.1996, M. Pereira [;] L.N. 323150-375500 #47561 [;] Malaise trap (ESUW).

#### Comments.

The white annulus on the flagellum, the longer and narrow metasomal tergum 1 and the nearly smooth mesoscutal lobes are distinctive for this species.

#### Etymology.

Named for the locality where part of the type series was collected, Zurqui de Moravia in San Jose Province.

**Figure 302.**
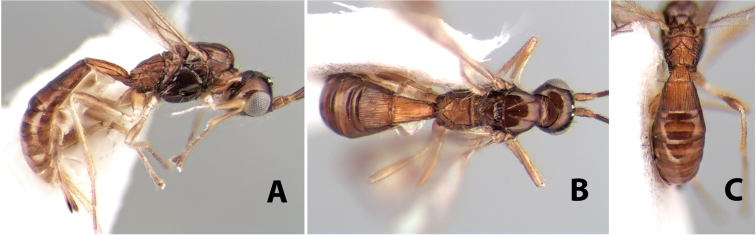
*Heterospilus zurquiensis* Marsh, sp. n., holotype.
